# Randomized controlled studies on the efficacy of antiarthritic agents in inhibiting cartilage degeneration and pain associated with progression of osteoarthritis in the rat

**DOI:** 10.1186/s13075-016-0921-5

**Published:** 2016-01-21

**Authors:** Erica M. TenBroek, Laurie Yunker, Mae Foster Nies, Alison M. Bendele

**Affiliations:** Medtronic Inc., 710 Medtronic Parkway, Minneapolis, MN 55432 USA; Bolder BioPATH, Inc., 5541 Central Avenue, Suite 160, Boulder, CO 80301 USA

**Keywords:** Osteoarthritis, Arthritis, Monoiodoacetic, Meniscal, Therapeutics, Degeneration, Pain, NF-κB, RANKL, Bone remodeling

## Abstract

**Background:**

As an initial step in the development of a local therapeutic to treat osteoarthritis (OA), a number of agents were tested for their ability to block activation of inflammation through nuclear factor κ-light-chain-enhancer of activated B cells (NF-κB), subchondral bone changes through receptor activator of nuclear factor κB ligand (RANKL)-mediated osteoclastogenesis, and proteolytic degradation through matrix metalloproteinase (MMP)-13 activity. Candidates with low toxicity and predicted efficacy were further examined using either of two widely accepted models of OA joint degeneration in the rat: the monoiodoacetic acid (MIA) model or the medial meniscal tear/medial collateral ligament tear (MMT/MCLT) model.

**Methods:**

Potential therapeutics were assessed for their effects on the activation of nuclear factor (NF)-κB, RANKL-mediated osteoclastogenesis, and MMP-13 activity in vitro using previously established assays. Toxicity was measured using HeLa cells, a synovial cell line, or primary human chondrocytes. Drugs predicted to perform well in vivo were tested either systemically or via intraarticular injection in the MIA or the MMT/MCLT model of OA. Pain behavior was measured by mechanical hyperalgesia using the digital Randall-Selitto test (dRS) or by incapacitance with weight bearing (WB). Joint degeneration was evaluated using micro computed tomography and a comprehensive semiquantitative scoring of cartilage, subchondral bone, and synovial histopathology.

**Results:**

Several agents were effective both in vitro and in vivo. With regard to pain behavior, systemically delivered clonidine was superior in treating MIA-induced changes in WB or dRS, while systemic clonidine, curcumin, tacrolimus, and fluocinolone were all somewhat effective in modifying MMT/MCLT-induced changes in WB. Systemic tacrolimus was the most effective in slowing disease progression as measured by histopathology in the MMT/MCLT model.

**Conclusions:**

All of the agents that demonstrated highest benefit in vivo, excepting clonidine, were found to inhibit MMP-13, NF-κB, and bone matrix remodeling in vitro. The MIA and MMT/MCLT models of OA, previously shown to possess inflammatory characteristics and to display associated pain behavior, were affected to different degrees by the same drugs. Although no therapeutic was remarkable across all measures, the several which showed the most promise in either model merit continued study with alternative dosing and therapeutic strategies.

**Electronic supplementary material:**

The online version of this article (doi:10.1186/s13075-016-0921-5) contains supplementary material, which is available to authorized users.

## Background

Osteoarthritis (OA) is a chronic degenerative disease that negatively impacts the lives of more than 27 million individuals in the United States [1]. It has been predicted that OA will be the fourth leading cause of disability by 2020 [2]. The early pain and inflammation of OA are typically treated with oral analgesics or anti-inflammatories, therapies that may be accompanied by significant side effects in a small percentage of patients [3]. As the disease progresses, intraarticular (IA) injections of steroids and hyaluronic acid (HA) offer temporary relief but generally fail to address the underlying degeneration or to consistently block disease progression. In the case of HA, the lack of efficacy may be due at least in part to limited persistence in the joint space [4] and short half-life [5]. Eventually, when the pain and degeneration become intractable, patients have few options other than joint replacement. Further revision or replacement surgeries may be required 15–20 years after the first replacement if the artificial joint fails [6]. Locally delivered therapeutics with the ability to inhibit disease progression and also block chronic pain might significantly delay the need for joint replacement. With the goal of developing an IA therapeutic, agents were chosen on the basis of their predicted ability to inhibit key processes involved in OA disease progression. Several targets were considered, including pivotal points within inflammatory pathways, proteinase production, and osteoclastogenesis.

Complex interactions involving joint inflammation, synovitis, secretion of mediators, cartilage degeneration, and subsequent subchondral bone remodeling have all been identified as playing a role in the development of chronic OA [7–10]. Inflammation is primarily related to activation of the classical nuclear factor κ-light-chain-enhancer of activated B cells (NF-κB) pathway and the synthesis of compounds that amplify the inflammatory process, which then may trigger degradation or remodeling of the cartilaginous matrix [11–13]. Evidence indicates that chemokines and cytokines secreted into the synovial fluid activate chondrocytes and trigger not only the synthesis of extracellular matrix but also additional synthesis of proinflammatory molecules [9]. In preclinical models, the inhibition of NF-κB or active proteinases has been shown to slow joint degeneration [11, 12]. Proteinases include but are not limited to matrix metalloproteinases (MMPs), particularly MMP-13 [14, 15], and aggrecanases, such as a disintegrin and metalloproteinase with thrombospondin motifs 4 and 5 (ADAMTS4 and ADAMTS5, respectively) [12, 16].

Subchondral bone changes associated with OA are driven largely by the nonclassical NF-κB-related receptor activator of nuclear factor κB ligand (RANKL) pathway [17, 18], activation of which may lead to both inflammation [10] and pain [19]. RANKL, a member of the tumor necrosis factor (TNF) superfamily, is produced by synovial tissue and binds to the receptor activator of NF-κB found on immune cells and osteoclasts [20]. Inflammation of synovial tissues within the joint attracts monocytes and macrophages, which, in the presence of RANKL and other signals produced by synovial fibroblasts and activated T cells, become osteoclasts. Such osteoclastogenesis, coupled with the activity of the osteoclasts and other inflammatory cells, may then trigger remodeling of subchondral bone adjacent to the synovium, neurovascular invasion, and formation of potentially painful osteophytes [21]. In addition to the RANKL pathway, increased transforming growth factor (TGF)-β activity in the subchondral bone stimulated by inappropriate mechanical loading may contribute to these boney changes [22].

The following studies were performed in an effort to determine whether the ability of a compound to block activation of NF-κB, synthesis of MMP-13, or activation of RANKL-mediated osteoclastogenesis might predict in vivo efficacy. The toxicity of the compounds was measured in vitro using cartilage, synovial cells, and/or HeLa cells. The most promising candidates were then tested using either of two widely accepted models of OA joint degeneration: the monoiodoacetic acid (MIA) model or the unilateral medial meniscal tear/medial collateral ligament tear (MMT/MCLT) rat model [23, 24]. Both models have been shown by others to recapitulate different aspects of degenerative joint disease [25, 26]. Primary endpoints in these studies included semiquantitative histopathological analysis of the affected joints and quantitative analysis of pain behavior.

## Methods

### Compound selection

Over 30 compounds either known to have or alleged to have therapeutic effects on any form of arthritis were considered for screening in vitro, with the ultimate goal of developing a local delivery formulation (Table 1 and data not shown). A multifaceted numerical ranking system was used to prioritize compounds, with higher values given to compounds with previously established anti-inflammatory characteristics, the ability to block MMPs associated with cartilage degeneration, and/or the ability to block bone remodeling. Those agents with at least one of these known characteristics and an ability to block a target within a pathway associated with pain were considered particularly attractive. Agents with regulatory approval in at least one country received priority over those not approved for clinical use. Compounds with demonstrated efficacy in clinical trials were ranked more highly than those with only in vitro or animal testing data. A number of agents were screened that are not discussed here, owing to their proprietary nature. The manufacturer, chemical structure, primary effects, published half-lives, and clinical use for the various compounds evaluated are provided in Table 1. Considerations for testing included cost or availability of the purified compound, stability or shelf life, solubility, required formulation, and the ability to partner or to license the particular compound for clinical use. In some cases, drugs were tested in vitro but were later discovered to be incompatible with delivery formulation for animal studies and so were not tested in vivo.

**Table 1:** Therapeutic candidates that were screened in vitro

Therapeutic candidates	MW	Primary effects	Structure	Notes on preclinical or clinical use
Alendronate sodium C_4_H_12_NaNO_7_P_2_ ∙ 3H_2_O Sigma A4978 10 mM stock in H_2_O	325.12	Bisphosphonate that targets farnesyl pyrophosphate synthase and inhibits osteoclast activity [68] Used as a positive control for bone changes in osteoclast assays and animal trials *t*½ >10 years		Approved for treatment of bone loss in osteoporosis and associated with a reduced prevalence of subchondral bone lesions in knee OA [69]
Ascomycin (FK520) C_43_H_69_NO_12_ A3835 (Sigma-Aldrich, St. Louis, MO, USA) 10 mM stock in DMSO	792	Analog of FK506 with strong immunosuppressant properties Acts by binding to immunophilins, especially macrophilin-12 Inhibits production of Th1 (interferon and IL-2) and Th2 (IL-4 and IL-10) Inhibits activation of mast cells [70]		The related compound, Pimicrolimus, is effective for treating atopic dermatitis and may also be effective for treating the same condition in psoriasis [71]
BAY-11-7082 C_10_H_9_NO_2_S 196870 (Calbiochem, San Diego, CA, USA) 48 mM stock in DMSO	207.25	Bay 11-7082 is an inhibitor of cytokine-induced IκBα phosphorylation (Calbiochem)		Not clinically approved
BMS-345541 C_14_H_17_N_5_ 401480 (Calbiochem) 3.9 mM stock in DMSO	255.3	Cell-permeable, allosteric site-binding inhibitor of IKK-2 (reported IC_50_ 300 nM) with tenfold higher selectivity for IKK-2 over IKK-1 (IC_50_ = 4 μM) [72, 73] *t*½ = 2.2 h		Blocks inflammation and joint destruction in murine arthritis model and blocked MMPs in arthritis model [72, 73] Not clinically approved
Acetyl-11-keto-β-boswellic acid, (*Boswellia serrata*) 110123 (Calbiochem) 9.75 mM in DMSO	512.7	Blocked TNF-stimulated MMP expression and protected against experimental arthritis [74] Binds to and inhibits IKKα and IKKβ to inhibit NF-κB signaling [75]		Clinically tested in an Ayurvedic formulation RA-11 (ARTREX, MENDAR; AyurCore, San Jose, CA, USA) with other nutraceuticals [76]
Clonidine C_9_H_9_Cl_2_N_3_ ∙ HCl Lot CTM-723 (AAIPharma, Wilmington, NC, USA) 8.69 mM stock (2 mg/ml)	266.55	α_2_-Receptor agonist and antihypertensive agent Possible induction of iNOS through NF-κB [77] *t*_1/2_ = 6–20 h		Used clinically to treat hypertension Clinically tested and found to be effective postoperative analgesic for knee arthroscopy [53]
CORM-2 (tricarbonyldichlororuthenium(II) dimer; CO-releasing molecule) [Ru(CO)_3_Cl_2_]_2_ Aldrich-288144 (Sigma-Aldrich) 10 mM stock in DMSO	512	Decreases oxidative stress in chondrocytes Inhibits IL-1β-induced TNF-α and downregulates NOS-2 and mPGES-1, and COX-2 expression Inhibits p65 NF-κB and HIF-1α DNA-binding activity Reduces IκBα phosphorylation [78] Downregulates MMP-1, MMP-3, MMP-10, MMP-13, and ADAMTS-5 in OA chondrocytes [79]		Not clinically approved
Curcumin Diferuloylmethane (*Curcuma longa*; turmeric) C_21_H_20_O_6_ C1386 (Sigma-Aldrich) 13.5 mM stock in EtOH	368.4	Reportedly inhibits both NF-κB activation and osteoclastogenesis induced by RANKL [80] Modulates genes involved in oxidative stress, apoptosis, inflammation, regulation of transcription, DNA replication, and cellular morphogenesis [81] *t*_1/2_ = 10 min intestinal *t*_1/2_ = 30–50 min plasma		Nutraceutical in clinical trials for treatment of colitis, colorectal cancer, and early Alzheimer’s disease Ayurvedic therapeutic and component of RA-11 (nutraceutical mixture) tested in clinical trial for OA [76] Reportedly disease-modifying while blocking pain [76]
Curcumin-14 Curcumin analog EF24 or 3,5-bis(2-flurobenzylidene)piperidin-4-one [82] Synthesized at Medtronic (Minneapolis, MN, USA) 10 mM stock in DMSO	311	Reportedly 10 times more potent than curcumin Inhibits NF-κB by inhibiting IκB kinase (IKK) [82] Potent anticarcinogenic activity inducing death of lung, breast, ovarian, and cervical cancer cells [82]		Novel monoketone analog of curcumin Not yet tested clinically
Diacerein C_19_H_12_O_8_ Nutraceutical that is enriched in rhubarb Breaks down to active metabolite rhein D9302 (Sigma-Aldrich) 27.15 mM stock in DMSO	368.3	Reportedly reduces IL-1β, caspase-3, inducible nitric oxide synthase (iNOS), and phosphorylation of c-Jun and c-Jun N-terminal kinase (JNK) Enhances expression of TGF-β1 and TGF-β2 [83] Reportedly inhibits osteoclast bone destruction Reportedly increases chondrocyte production of GAGs and collagen in vitro [83, 84]		Claimed disease-modifying OA drug that may slow joint space narrowing [85, 86] Reportedly better than NSAIDs for knee and hip OA with a carryover effect after discontinuation [87] ECHODIAH (3-year placebo-controlled trial on hip OA) showed some positive improvement [85, 86]
Epigallocatechin-3-gallate C_22_H_18_O_11_ 324880 (Calbiochem) 11 mM stock in DMSO	458.4	Catechin inhibitor of osteoclastogenesis and NF-κB found in green tea [88] Potent antioxidant that may inhibit cartilage degradation by suppressing AGE-mediated activation and the catabolic response in human chondrocytes [89, 90] *t*_1/2_ = 3–5 h		Nutraceutical tested in numerous clinical trials for efficacy in several different diseases [91]
Fluocinolone Acetonide C_24_H_30_F_2_O_6_ F3132 (Sigma-Aldrich) 50 mM stock in EtOH	452.5	Corticosteroid (potent) Blocks IL-1 and TNF production and TNF-induced apoptosis [92] *t*_1/2_ = 1.3 – 1.7 h		FDA-approved as sustained-release intraocular implants for the treatment of diabetic macular edema and uveitis [52]
GM6001 (generic names galardin, ilomostat) C_20_H_28_N_4_O_4_ 364205 (Calbiochem) 5 mM stock in DMSO	388.5	Cell-permeable, broad-spectrum inhibitor of matrix metalloproteinases (MMPs) Prevents the release of TNF-α and blocks endotoxin-induced death in mice (per Calbiochem)		In clinical testing for eye disease and COPD (Glycomed, San Diego, CA, USA; Arriva Pharmaceuticals, Alameda, CA, USA; Quick-Med Technologies, Gainesville, FL, USA)
IKK-2 inhibitor IV [5-(p-Fluorophenyl)-2-ureido]thiophene-3-carboxamide (TPCA-1) Positive control for IKK-2-mediated inflammation Calbiochem	279.3	Reportedly a potent cell-permeable inhibitor of IKK-2 (IC_50_ = 18 nM) with selectivity over IKK-1, JNK, and p38MAPK Inhibits TNF-α in human monocytes (IC_50_ = 0.15–2.5 μM) Blocks IL-8 and IL-6 in synovial fibroblasts (IC_50_ = 100 nM) [93] Reduced paw edema in rat inflammatory arthritis model (per Calbiochem) (about 100 % at 30 mg/kg)		Not clinically approved Efficacy of TPCA-1 was similar to that of etanercept [93] Inhibitor was tested as potential therapeutic for rheumatoid arthritis (e.g., GSK, London, UK)
NF-κB activation inhibitor IV [resveratrol derivative (E)-2-fluoro-4′-methoxystilbene] 481412 (Calbiochem)	228.1	Experimentally used as an anti-inflammatory but not an antioxidant 130-fold more potent than resveratrol at inhibiting NF-κB [94]		Not clinically approved
IKK-2 inhibitor V IMD-0354 *N*-(3,5-bis-trifluoromethyl-phenyl)-5-chloro-2-hydroxy-benzamide C_15_H_8_ClF_6_NO_2_ 3.8 mM stock in EtOH 401482 (EMD Millipore, Billerica, MA, USA)	383.7	Cell-permeable IKK-2 inhibitor and established inhibitor of NF-κB pathway Reported IC_50_ = 250 nM for block of IκBα phosphorylation [95]		Approved for atopic dermatitis (Institute for Medicinal Molecular Design, Tokyo, Japan)
IKK-2 inhibitor VI (5-Phenyl-2-ureido)thiophene-3-carboxamide C_12_H_11_N_3_O_2_S 401483 (Calbiochem) 3.8 mM stock in DMSO	261.3	Reported cell-permeable, reversible inhibitor of IKK-2 (IC50 = 13–18 nM) Orally bioavailable		Not clinically approved
IKK-2 inhibitor VIII (ACHP) C_21_H_24_N_4_O_2_ 401487 (Calbiochem) 2 mM stock in DMSO	364.4	A cell-permeable piperidinyl-pyridine compound and selective inhibitor of IKK-2 (IC_50_ = 8.5 and 250 nM for IKK-2 and IKK-1, respectively) Orally bioavailable in both rats and mice and effectively inhibited arachidonic acid–induced swelling in murine model (per Calbiochem)		Not clinically approved
Meloxicam (Mobic; Boehringer Ingelheim Pharmaceuticals, Ridgefield, CT, USA) C_14_H_12_N_3_NaO_4_S_2_ M3935 (Sigma-Aldrich) 2 mM stock in DMSO (saline for in vivo)	351.4	Nonsteroidal anti-inflammatory drug (NSAID) that inhibits prostaglandin synthetase (cyclooxygenase) and prostaglandin synthesis Inhibits NF-κB in activated macrophages [96] *t*_1/2_ = 15–20 h		Approved for relief of the symptoms of arthritis, primary dysmenorrhea, and fever and also as an analgesic, especially where there is an inflammatory component
Pimecrolimus C_43_H_68_ClNO_11_ S5004 (Selleck Chemicals, Houston, TX, USA) 10 mM stock in DMSO	810.5	Ascomycin macrolactam derivative Like tacrolimus, a calcineurin inhibitor that inhibits release of inflammatory cytokines [97] *t*_1/2_ = 30–100 h		Approved for atopic dermatitis (ELIDEL; Meda Pharma, Luxembourg)
Resveratrol C_14_H_12_O_3_ 554325 (Calbiochem) 50 mM stock in DMSO	228.2	Suppresses IL-1β signaling and IL-1β-stimulated apoptosis in osteoarthritis [98] A natural inhibitor of NF-κB [99] *t*_1/2_ = 1–3 h		Nutraceutical said to protect against neuronal cell death; interferes with the stages of initiation, promotion, and progression of cancer; normalizes blood glucose levels; and acts as an anti-inflammatory The focus of many clinical trials [100]
Rhein (diacerein derivative) C_15_H_8_O_6_ R7269 (Sigma-Aldrich) 10 mM stock in DMSO	284.2	Anthraquinone-active metabolite of diacerein Reduces proliferation of chondrocytes and synoviocytes; inhibits NF-κB activation in vitro [101] *t*_1/2_ = 4.3 h		Orally administered diacerein is completely converted to rhein before reaching the systemic circulation
SC514 C_9_H_8_N_2_OS_2_ 401479 (Calbiochem) 2 mM stock in DMSO	224.3	Selective inhibitor of IKK-2 *t*_1/2_ = 12 min		Experimental use only
Sulfasalazine (Azulfidine; Pfizer, New York, NY, USA) C_18_H_14_N_4_O_5_S S0883 (Sigma-Aldrich) 200 mM stock in DMSO	398.4	NSAID *t*_1/2_ = 5 – 10 h		Approved for use in RA and OA Generic
Sulindac C_20_H_17_FO_3_S S8139 (Sigma-Aldrich) 70 mM stock in DMSO	356.4	NSAID *t*_1/2_ = 7.8 h		Approved for use in RA and OA Generic
Tacrolimus (FK506) C_44_H_69_NO_12_ Anhydrous F4679 (Sigma-Aldrich) or Prograf (Astellas Pharma, Tokyo, Japan) for clinical use 12.4 mM stock in EtOH	804	Immunosuppressant Blocks TNF-α and IL-1β production [64] May be disease-modifying while blocking pain Blocks calcineurin pathway and bone remodeling in RA [65] *t*_1/2_ = 11.3 h		Approved for RA in Japan in 2005 (Astellas Pharma) Approved immunosuppressant in United States (1994) Janus drug-eluting stent (Sorin Biomedica, Milan, Italy) Polymer elution tested in models of uveitis [60]
Tranilast (*N*-(3′,4′-dimethoxycinnamoyl)anthranilic acid; brand name Rizaben) C_18_H_17_NO_5_ Calbiochem 616400 2 mM stock in DMSO (1 % sodium bicarbonate in vivo)	327.3	Anti-inflammatory and analgesic properties in collagen-induced arthritis (RA model) [34]. Suppresses COX-2 and iNOS expression, reduces PGE_2_ and iNOS-derived NO production in stimulated macrophages. Diminishes TNF-α and IL-1β production [35]. T_1/2_ = 7.4 h		In testing for restenosis (SmithKline Beecham, London, UK; Nuon Therapeutics, San Mateo, CA, USA) Also used and/or tested in Japan (Kissei Pharmaceutical, Matsumoto, Japan) as an antiallergic, antiasthmatic, ophthalmic agent
Triamcinolone acetonide C_24_H_31_FO_6_ T6501 or clinical grade (Sigma-Aldrich) 10 mM stock in EtOH	434.5	Steroid *t*_1/2_ = 88 min		Approved for IA injection in OA
Triamcinolone hexacetonide C_21_H_27_FO_6_ Aristospan (Sandoz, Princeton, NJ, USA) 37.5 mM stock (20 mg/ml)	532.7	Steroid *t*_1/2_ = 88 min		Approved for IA injection in OA
Withaferin A (withanolide) C_28_H_38_O_6_ ALX-350-153 (Axxora, Farmingdale, NY, USA) 2.125 mM in EtOH	470.6	Reportedly inhibits NF-κB activation by preventing the TNF-α-induced activation of IKK-β (IKK-2) [102] Blocks osteoclastogenesis of bone remodeling [103] *t*_1/2_ = 1.36 h		Medicinal plant derivative (nutraceutical) for RA [104] and other inflammatory disorders, especially in India RA-11 (Ayurvedic therapeutic containing Withaferin A) tested in clinical trial for OA (ARTREX; AyurCore) [76]

ACHP, 2-amino-6-(2-(cyclopropylmethoxy)-6-hydroxyphenyl)-4-(4-piperidinyl)-3-pyridinecarbonitrile; AGE, advanced glycation end product; CORM-2, carbon monoxide-releasing molecule 2; COX-2, cyclooxygenase-2; DMSO, dimethyl sulfoxide; ECHODIAH, Evaluation of the Chondromodulating Effect of Diacerein in Osteoarthritis of the Hip; EGCG, epigallocatechin gallate; FDA, U.S. Food and Drug Administration; FK506, tacrolimus; FK520, ascomycin; GAG, glycosaminoglycan; HIF-1α, hypoxia-inducible factor 1α; IA, intraarticular; IC_50_, concentration at which the response is reduced by half; IκΒα, inhibitor of nuclear factor κB; IKK, inhibitor of nuclear factor κB kinase; IL, interleukin; iNOS, inducible nitric oxide synthase; JNK, c-Jun N-terminal kinase; MAPK, mitogen-activated protein kinase; MMP, matrix metalloproteinase; MW, molecular weight; NF-κB, nuclear factor κ-light-chain-enhancer of activated B cells; NSAID, nonsteroidal anti-inflammatory drug; OA, osteoarthritis; PGE, prostaglandin E; RA, rheumatoid arthritis; RANKL, receptor activator of nuclear factor κB ligand; SC514, selective reversible inhibitor of inhibitor of nuclear factor κB kinase 2; *t*_1/2_, half-life; TGF-β, transforming growth factor β; Th, helper T immune response-related cell; TNF-α, tumor necrosis factor α; TPCA-1, 5-(p-fluorophenyl)-2-ureido]thiophene-3-carboxamide

Basic information is provided about the source and structure of the chemical, the stock solution, and previous in vitro and in vivo studies [68–104]

### NF-κB

NF-κB activity was assessed using a previously described dual plasmid reporter system [27]. HeLa cells were transfected with NF-κB luciferase (Stratagene Products Division, Agilent Technologies, La Jolla, CA, USA) and pRLuc-N3 (PerkinElmer, Waltham, MA, USA) at an 8:1 ratio, respectively, in triplicate. After 48 h, drugs were applied with or without TNF-α (20 ng/ml, PH C3016; BioSource, San Diego, CA, USA). NF-κB activity was measured 5 h later with a Dual-Glo Luciferase Assay (Promega, Madison, WI, USA) and an Omni plate reader (BMG Labtech, Cary, NC, USA). The ratio of firefly luciferase luminescence (NF-κB reporter) to Renilla luciferase luminescence (control) was normalized for the number of viable transfected cells. Data are reported as a ratio of activity of viable treated cells over the activity of cells not exposed to TNF-α or drug (fold stimulation above basal untreated levels of activity). The viability of HeLa cells with and without drug was also measured using a CellTiter-Glo luminescent viability assay (Promega). See Additional file 1: Table S3 for results.

### Osteoclastogenesis

RAW 264.7 cells (American Type Culture Collection [ATCC], Manassas, VA, USA), a murine monocytic cell line that differentiates in the presence of RANKL, was used for measurements of osteoclastogenesis and osteoclast activity. Cells were amplified on nonadherent culture substratum in high-glucose Dulbecco’s modified Eagle’s medium (DMEM) containing 10 % fetal bovine serum (FBS) and penicillin-streptomycin in a 37 °C, 5 % CO_2_, humidified incubator and were passaged by manual dissociation. Cells were seeded on 16-well osteologic slides (BD BioCoat; BD Biosciences Discovery Labware, Billerica, MA, USA) at 0.125 × 10^4^ cells/0.25 ml/well in triplicate and were allowed to adhere for 24 h, then stimulated to differentiate (30 mg/ml RANKL; PeproTech, Rocky Hill, NJ, USA) with or without drug for 7 days. Plates were subsequently rinsed, bleached, dried, and analyzed for matrix degradation using a Nikon COOLSCOPE (Nikon Instruments, Melville, NY, USA). Matrix resorption was quantified using EclipseNet (Nikon Instruments)/Visiopharm (Visiopharm, Hørsholm, Denmark) software (see Additional file 1).

### MMP-13 activity and toxicity

Differentiated chondrogenic pellets were used both to test the ability of drugs to block MMP-13 activity and to test for drug toxicity. Normal human articular chondrocytes (Clonetics CC-2550, adult male, lot 5 F1452; Lonza, Walkersville, MD, USA) were maintained in complete growth medium (CC-4409, R3-insulin-like growth factor [IGF]-1, human recombinant fibroblast growth factor β, transferrin, insulin, FBS, gentamicin/amphotericin-B and basal medium; Lonza) in a humidified incubator at 37 °C with 5 % CO_2_. Cells were trypsinized at approximately 85 % confluence and washed, and 2.2–2.5 × 10^5^ cells/75 μl were allowed to settle in sterilized, V-bottomed, nonadherent 96-well plates (Thermo Scientific, Waltham, MA, USA). Plates were centrifuged at 600 rpm to form aggregates, and cell pellets were fed into chondrocyte differentiation medium (CC-3225; Lonza) three times per week. By 28 days, the pellets expressed markers of mature cartilage (e.g., type II collagen and aggrecan; data not shown). To mimic osteoarthritic cartilage, pellets were treated with or without TNF-α for 24 h before application of drug with or without TNF for another 24 h. Supernatants were collected and analyzed for MMP-13 using an enzyme-linked immunosorbent assay (GE Healthcare Life Sciences, Little Chalfont, UK). Cytotoxicity to pellets was assessed using CellTiter-Blue (Promega).

### Synovial toxicity

SW982 (ATCC HTB-93), a human synovial sarcoma cell line, was grown in low-glucose DMEM with 10 % FBS in a 37 °C, 5 % CO_2_, humidified incubator. Cells were trypsinized and plated at 1–2 × 10^4^ cells/well in Optilux 96-well microtiter plates (BD Falcon; BD Biosciences Discovery Labware) and were exposed to drug for approximately 24 h. Viability was measured with the CellTiter-Glo luminescent viability assay (Promega). A range was first identified in which the drugs might be both effective and nontoxic. To more accurately predict the effects of the drugs on osteoarthritic synovium, cells were tested with or without TNF-α (20 ng/ml; BioSource).

### Ethics and compliance

The human chondrocytes used in toxicity and MMP studies were procured from Lonza in accordance with U.S. Food and Drug Administration regulations (21 CFR part 1271: Human Cells, Tissues, and Cellular and Tissue-Based Products) that govern tissue banking. Lonza is registered under the identifier FEI: 0001114298 and holds a permit to operate a tissue bank in the State of Maryland with licenses in tissue banking in the states of New York and California. Records of informed consent were required for all human tissue.

### Functional testing

#### Digital Randall-Selitto test

The digital Randall-Selitto (dRS) test is said to be a reliable and repeatable measure of neuropathic, bone, and inflammatory pain behavior in several different models [28–30], including those of arthritic or joint pain [29, 31, 32]. Baseline and posttreatment values were evaluated using a dRS test device (IITC Life Science, Woodland Hills, CA, USA). Animals were allowed to acclimate to the testing room before all experimentation and for a minimum of 30 minutes before testing. To ensure that their hind limbs were accessible, the animals were gently suspended in a restraint sling. The joint compression threshold was measured once at each time point for the ipsilateral and contralateral knee joints. Pressure was applied gradually over approximately 10 seconds to the medial and lateral aspects of the knee joint. Measurements were taken from the first observed behavior of vocalization, struggle, or withdrawal. A cutoff value of 600 g was used to prevent injury to the animal.

#### Weight bearing

Weight bearing (WB; incapacitance) was measured five times per day, once per week, using a Linton incapacitance meter (Stoelting Co., Wood Dale, IL, USA). Rats were acclimated to the meter before study initiation, and, similarly, immediately before WB, the animals were placed in the meter and allowed to acclimate for 2–5 minutes. Each hind paw was placed on a separate force plate so that the force exerted by each side could be averaged over a 5-second interval, and the mean of three readings was taken for each data point. Right paw force was compared with that on the left side for each group to confirm that animals were displaying incapacitance and/or pain behavior. Differences in force (left minus right) and right paw force as a percentage of the total force exerted by both paws were determined and compared between groups.

#### Monosodium iodoacetic acid

Sprague Dawley rats (100–125 g; Harlan Laboratories, Indianapolis, IN, USA) were allowed food and water and maintained on a 12:12-h light/dark schedule. Following isoflurane anesthesia, 50 μl (2 mg) of MIA was injected into the synovial space of the left knee. Animals were randomly assigned to groups, and pain behavior was assessed 7, 14, 21, and 28 days following MIA injection and intervention using WB with the Stoelting incapacitance meter (trial 1) or the dRS test (trials 2 and 3). The dRS test was adopted for the second and third MIA trials after its sensitivity in the MIA model was validated (ALGOS 171.3; 11/16/2008SFN; Algos Therapeutics, St. Paul, MN, USA). Animals received MIA treatment and then saline control or therapeutic. WB or dRS measurements were done immediately before treatments and at 1, 3, 5, and 24 h posttreatment as described further below. Histopathology was performed at each time point and at study termination. All studies were conducted in accordance with the International Association for the Study of Pain Guidelines and were approved by the Algos Therapeutics, Inc. Institutional Animal Care and Use Committee (IACUC) before initiation. Those running the studies were blind to the treatments. See Additional file 1 for further details.

#### Medial meniscal tear

Male Lewis rats (260–295 g; Charles River Laboratories, Wilmington, MA, USA) were allowed food and water and maintained on a 12:12-h light/dark schedule. Following isoflurane anesthesia, the medial collateral ligament was transected just below its attachment to the meniscus in the right knee. The meniscus was cut at its narrowest point away from the ossicles so as not to damage the tibial surface and to ensure that the anterior and posterior meniscus halves were freely movable [33]. In trial 1, agents were administered subcutaneously daily for 3 weeks beginning 1 day before surgery. In trial 2, drugs were administered IA weekly beginning 1 week following surgery. Three weeks postsurgery, animals were humanely killed and their right knees were collected for histopathology. All studies were conducted in accordance with the International Association for the Study of Pain Guidelines and were approved by the Bolder BioPATH IACUC (Boulder, CO, USA) in compliance with regulations before study initiation (IACUC protocol BBP03-006). Those running the studies were blinded to the treatments. See Additional file 1 for further details.

### Histopathology

Dissected knee joints were fixed in 10 % formaldehyde, decalcified for 2 days in 10 % formic acid, trimmed into two equal frontal halves, and processed and embedded using conventional methods. Sections were stained with either hematoxylin and eosin or toluidine blue stain, or both stains. The histopathologist was blinded to all treatments.

#### MIA model

Following functional testing in the first and second MIA trials, femurs were fixed in paraformaldehyde, processed, sectioned, stained with hematoxylin and eosin or Safranin O at Premier Laboratory (Longmont, CO, USA), and analyzed by a veterinary pathologist. For MIA trial 1, sections were evaluated for the percentage area of chondrocyte necrosis and/or proteolytic degeneration using a 5-point scale with 0 = none; 1 being <10 %; 2 = 10–30 %; 3 = 30–60 %; 4 = 60–90 %; and 5 being >90 %. Other characteristics of inflammation, proliferation, and integrity of the synovium, subchondral bone, and articular cartilage were scored as normal, minimal, mild, moderate, marked, or increased or decreased when severity was not graded. MIA trial 2 was measured similarly and then cross-checked according to the methods used for MIA trial 3 (see text below).

For MIA trial 3, joints were fixed and toluidine blue–stained sections of the rat knees were comprehensively analyzed from test and control subjects as detailed in Additional file 1. Briefly, a 5-point cartilage matrix score was derived based upon (1) approximate percentage of total loss of articular chondrocytes for each of four articular surfaces, (2) estimated proteoglycan loss (via Safranin O staining), and (3) loss of interstitial matrix. Chondrocyte necrosis and proteolytic degeneration of the femurs were scored on a 5-point scale as in trial 1. A femoral cartilage degeneration score and a three-zone sum of the tibial cartilage degeneration scores (mean of three levels) were also summed to create a total cartilage degeneration score. The mean osteophyte score for each joint was added to this value to create a total joint score with matrix. Other parameters assessed included synovial membrane inflammation and proliferation. Representative images from each group were also collected (see, e.g., Fig. 4 and Additional file 1: Fig. S55, Trial 3).

#### MMT/MCLT model

For MMT/MCLT rat knee joints, sections were cut in 200-μm steps; stained with toluidine blue (also with a right and left half per section); and similarly analyzed for cartilage degeneration, proteoglycan loss, collagen damage, and osteophyte formation. Results were averaged across the three sections for an overall semiquantitative score. Regional differences across the tibial plateau were taken into consideration by dividing each section into three zones delineated using an ocular micrometer (outside, middle, and inside). Scores were based on the percentage of area affected within the zone.

Cartilage degeneration in the tibia was scored on a 5-point scale as detailed in the Methods section of Additional file 1. The total extent of degeneration of the tibial plateau was measured (in micrometers) and included an analysis of cell loss, proteoglycan loss, and collagen damage. Significant cartilage degeneration reflecting any degeneration extending through more than 50 % of the cartilage thickness was also measured. Collagen damage across the medial tibial plateau (the most severely affected section of the two halves) was also quantified.

Osteophytes and femoral cartilage degeneration were analyzed as detailed in Additional file 1. Scoring of the osteophytes and categorization into small, medium, and large was performed using an ocular micrometer. The actual osteophyte measurement (tidemark to farthest distance point extending toward the synovium) was also recorded. The femoral cartilage degeneration score and the three-zone sum of the tibial cartilage degeneration scores (mean of three levels) were summed to create a total cartilage degeneration score. The mean osteophyte score for each joint was added to this value to create a total joint score.

Overall findings, including synovial health, were also assessed and documented (see, e.g., Additional file 1: Table S1), and representative images were acquired for each animal (see, e.g., Additional file 1: Figs. S57–S59).

### μCT

At the end of MIA trial 2, five knees from each group were fixed in formalin and scanned at 55 kV, 145 μA, and 300-ms image acquisition time (μCT40; SCANCO Medical AG, Brüttisellen, Switzerland). Slice thickness was 16 μm with isotropic voxels, and 511 projections were taken for each 360-degree rotation. To correct for beam-hardening artifacts, a μ-law scaling algorithm was applied at a level of 200 mg/cm^3^ hydroxyapatite. Scans were reformatted from transverse to sagittal planes before analysis. The tibial plateau was identified, and the slice numbers corresponding to the medial side were recorded. One hundred slices from the middle of the region were selected, and a contour was applied to the subchondral bone that was 50 pixels (800 μm) in diameter. The final size of the region of interest (ROI) was 800 μm × 1600 μm before Gaussian filtering. Care was taken to exclude cortical bone and include only trabecular bone in the ROI. A morphometric analysis on the ROI of each sample was completed, with the same size Gaussian filter and threshold applied throughout the entire study. Every effort was made to minimize variability, including consistently using the same methods and equipment. One specially trained and skilled scientist performed all μCT measurements. The output of this analysis included total volume analyzed, bone volume, trabecular bone measurements, and a structural model index. See Additional file 1 for further details.

### Statistics

Statistical analyses were conducted using Prism 5.01 (GraphPad Software, La Jolla, CA, USA) or MS Excel software (Microsoft, Redmond, WA, USA) with the Bio-biomedical statistical package designed at Medtronic plugin. For in vitro studies, one- or two-way analysis of variance (ANOVA) was used to look for differences; if significant, then further analysis was done with Dunnett’s or pairwise *t* tests. In general, results of pairwise comparisons with controls using a standard two-tailed *t* test are shown in the figures with asterisks indicating significance of *p* ≤ 0.05. Samples were tested at least in triplicate, and all tests were repeated. Further details are included in the text and figure legends.

The mean and standard error of the mean (SEM) were determined for each animal treatment group. For the MIA dRS studies, one-way ANOVA was used to compare joint compression thresholds of experimental time points with the pretreatment value on any given testing day. OA-related pain in the vehicle group was estimated at each time point on each testing day by comparing ipsilateral (injured) with contralateral (normal) joint compression thresholds using paired *t* tests. The progression of OA-related pain for other treatments was estimated by comparing pretreatment joint compression thresholds on each testing day with pre-MIA measurements using repeated measures one-way ANOVA and by comparing pretreatment joint compression thresholds with vehicle-treated animals on each testing day using an unpaired *t* test or one-way ANOVA.

For histopathological comparisons, nonqualitative scales were used for scoring, and a treatment group mean ± SEM for each score and measurement was determined as previously recommended by Gerwin et al. for the Osteoarthritis Research Society International histopathology initiative [25]. Statistical analyses were then performed using parametric ANOVA methods. When several treatment groups were compared, multiple comparison procedures such as the Bonferroni or Tukey correction were used. Dunnett’s test was applied for comparisons with vehicle. Scored parameters were analyzed using a Kruskal-Wallis test with Dunn’s posttest.

To analyze data from the MMT/MCLT WB studies, a repeated measures one-way ANOVA comparing pre- and posttreatment WB measurements with the vehicle-treated animal or pain behavior control treatment was used as indicated. Similarly, for the MIA WB studies, a repeated measures one-way ANOVA with *p* ≤ 0.05 was used, and Dunnett’s multiple comparisons post hoc test was performed when appropriate.

For analysis of μCT data, one-way ANOVA was used to look for differences between groups; if *p* < 0.05, then a Bonferroni post hoc test was performed to identify any significant differences between groups.

## Results

### In vitro studies

**Fig. 1:**
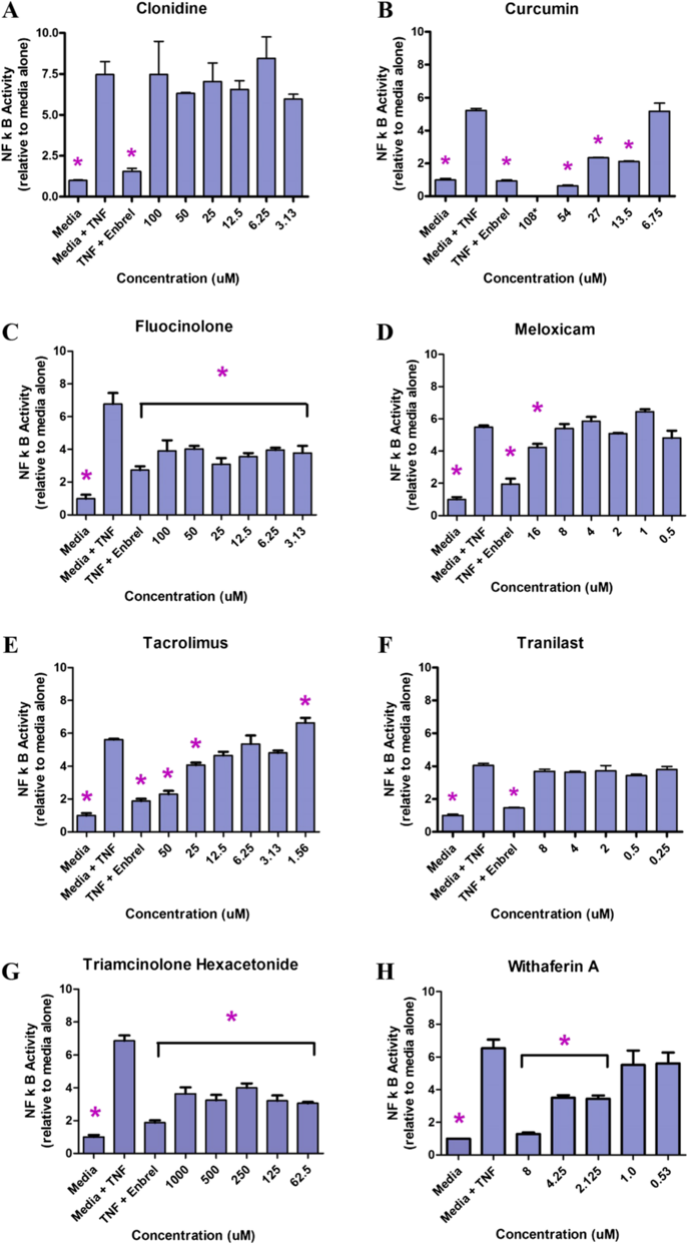
**a**–**h** Tumor necrosis factor (TNF)-stimulated nuclear factor κ-light-chain-enhancer of activated B cells (NF-κB) activity relative to media alone, with or without drug. The ratio of the NF-κB activity of viable cells to the activity detected without exposure to TNF-α (fold stimulation above basal untreated levels of activity with or without drug). Controls: Media = untreated HeLa cells; Media + TNF = cells treated with TNF-α; Media + Enbrel = cells treated with TNF-α in the presence of Enbrel, a known TNF inhibitor; Test = cells treated with TNF-α in the presence of different concentrations of drug. Shown are resultsFollowing one-way analysis of variance with the drugs tested in vivo (i.e., clonidine, curcumin, fluocinolone, meloxicam, tacrolimus, tranilast, triamcinolone hexacetonide, and withaferin). Following one-way analysis of variance, pairwise comparisons with the media TNF control were made using a standard two-tailed *t* test. *p ≤ 0.05. Additional data is provided in Additional file 1

Cells typically found in the joint space, such as chondrocytes, synovial cells, and macrophages, proved difficult to transfect with the reporter constructs, so the NF-κB assay was performed in a HeLa cell line previously shown to respond to stimulants of NF-κB in this dual reporter assay [27]. Etanercept (Enbrel; Amgen, Thousand Oaks, CA, USA), a known inhibitor of TNF and NF-κB, was used as a control and inhibited NF-κB at expected concentrations (Fig. 1).

**Fig. 2:**
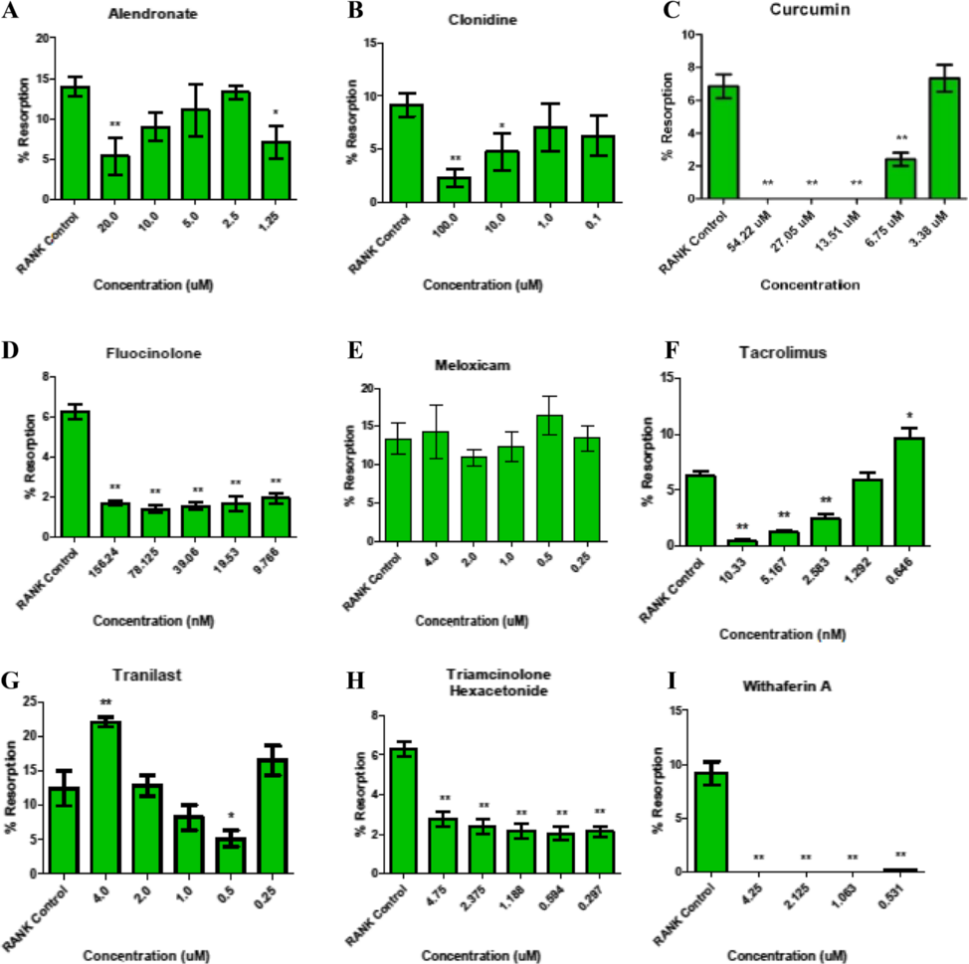
**a**–**i** The effect of tested drugs (alendronate, clonidine, curcumin, fluocinolone, meloxicam, tacrolimus, tranilast, triamcinolone hexacetonide, and withaferin) on osteoclast differentiation and resorption. The lowest concentrations tested are shown; additional data is provided in Additional file 1. Following one-way analysis of variance, pairwise comparisons with the tumor necrosis factor control were made using a standard two-tailed *t* test. *p ≤ 0.05, **p ≤ 0.001. *RANK* receptor activator of NF-κB

**Fig. 3:**
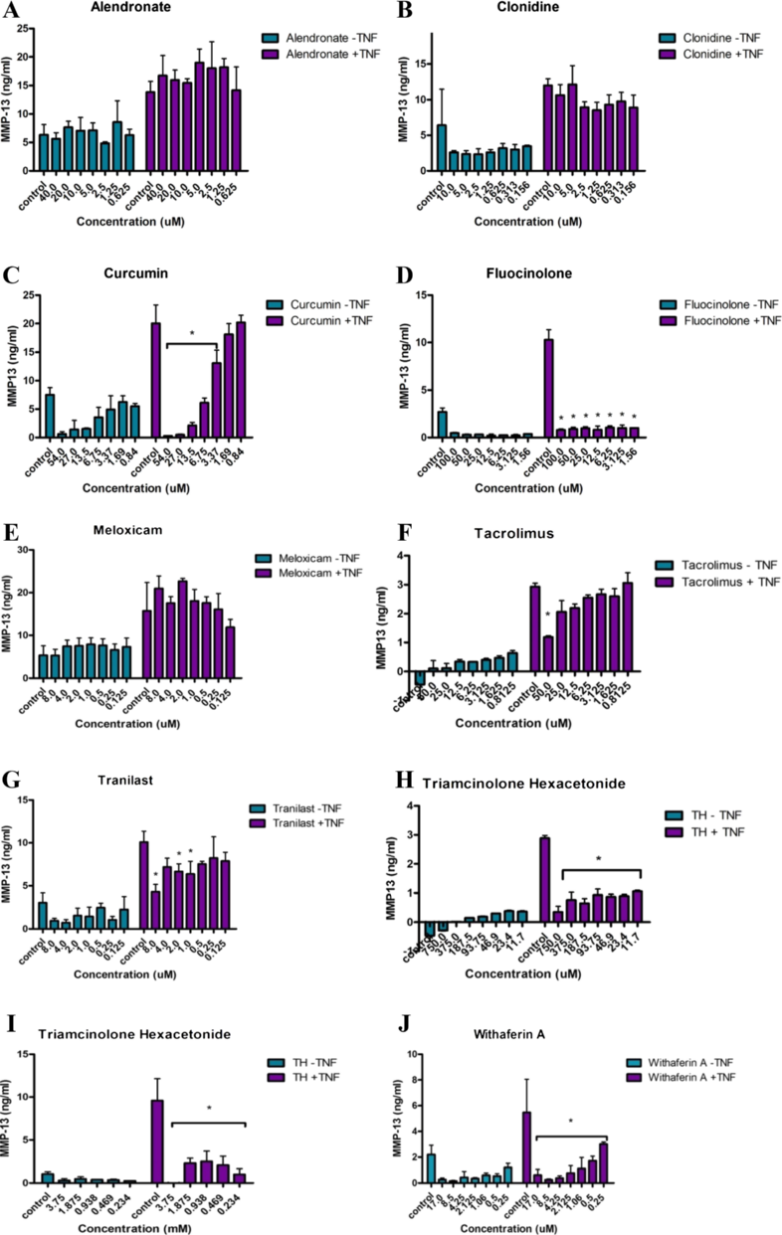
**a**–**d** The effect of tested drugs (alendronate, clonidine, curcumin, fluocinolone, meloxicam, tacrolimus, tranilast, triamcinolone hexacetonide, and withaferin) on matrix metalloproteinase (MMP)-13 activity of the chondrogenic pellets. The lowest concentrations tested are shown. Additional data is provided in Additional file 1. Following one-way analysis of variance, pairwise comparisons with the tumor necrosis factor (TNF) control were made using a standard two-tailed *t* test. *p ≤ 0.05

Agents tested in vitro are shown in Table 1. Many of these compounds were found to block NF-κB and MMP-13 activity as well as RANKL-mediated osteoclastogenesis and at a range of concentrations (Figs. 1, 2 and 3, Table 2, and Additional file 1: Figs. S3–S18). The drug IMMD-0354 (Institute for Medicinal Molecular Design, Tokyo, Japan), an inhibitor of nuclear factor κB kinase (IKK)-2 inhibitor, displayed fairly effective inhibition of the three targets and was relatively nontoxic to chondrocytes. However, IMMD-0354 was somewhat toxic to synoviocytes and HeLa cells (Table 3 and Additional file 1: Figs. S19–S28), so it was not prioritized for in vivo testing. Fluocinolone was notably nontoxic and effective in all assays over a broad range of concentrations (Figs. 1, 2 and 3). Curcumin was also relatively nontoxic and effective in all assays (Figs. 1, 2 and 3 and Additional file 1). Tacrolimus inhibited NF-κB and MMP-13 at micromolar concentrations but was more effective in inhibiting the nonclassical (osteologic) NF-κB pathway (2.6 nM). Pimecrolimus and ascomycin, structurally related to tacrolimus, inhibited matrix resorption and NF-κB over a broad and nontoxic range, but they failed to inhibit MMP-13 at the concentrations tested (to 40 μM) (Table 2 and Additional file 1: Fig. S18). Tacrolimus inhibited MMP-13 at 50 μM (Additional file 1: Fig. S10). Triamcinolone acetonide (TA) was more effective in the NF-κB assay than in the bone and MMP-13 assays (Additional file 1: Fig. S4 vs. Figs. S8 and S18). Triamcinolone hexacetonide (TH) was consistently more effective than TA in vitro, possibly because of its formulation (Figs. 1, 2 and 3 and Additional file 1), so this clinical formulation was used in vivo. Withaferin was highly effective in the bone assay (Fig. 2i) and also inhibited NF-κB and MMP-13 at a range of concentrations (Figs. 1 and 3). On the basis of in vitro testing, fluocinolone, curcumin, withaferin, tacrolimus, and TH were chosen for initial testing in the MIA model.

**Table 2:** Efficacy of drugs in NF-κB, MMP-13, and bone remodeling assays

Drug	Test range (μM)	Overlapping effective and nontoxic range^a^ (μM)	NF-κB	MMP-13	Bone	Tested in vivo
Alendronate	0.625–40	Bone control	N/A	NE	1.25 and 20 μΜ^b^	MMT/MCLT
Amrinone	0.625–80	80	NE	80	NE	No
Ascomycin	0.01–40	1.25–40	E	NE	E	TBD
BAY-117082	0.188–192	6–192	NE	E	E	Proprietary
BMS-345541	0.24–15.6	0.244	NE	E	E	Proprietary
Boswellic acid	0.61–680	40–680	E	E	E	Purity an issue
Clonidine	0.156–100	10–100	NE	NE	E	MMT/MCLT and MIA
CORM-2	0.625–40	20–40+	N/A	E	E	Proprietary
Curcumin	0.84–108	13.5–54	E	E	E	MMT/MCLT and MIA
Curcumin-14	1.25–80	2.5–10	E	E	NE	TBD
Diacerein	1.5–217	Toxic to chondrocytes	E	E	E	Toxicity and purity issues
EGCG	0.688–44	5.5–44	E	E	NE	Purity an issue
Fluocinolone^c^	0.010–100	3.1–100	E	E	E	MMT/MCLT and MIA
GM6001	0.33–25	NA	NA	25	NE	No
NF-κB activation inhibitor IV	0.156–10	NA	7.7	NE	N/A	No
IKK inhibitor V^d^ (IMMD-0354)	0.025–26	1.625–26		E	E	Cost-prohibitive/proprietary
IKK inhibitor VI	0.028–15.2	0.24–15.2	E	E	E	Cost-prohibitive/proprietary
IKK inhibitor VIII (ACHP)	0.25–16	4 (toxicity to synovial cells)	N/A	E	E	Cost-prohibitive/proprietary
Meloxicam	0.125–32	16–32	E	NE	NE	MIA
Pimecrolimus (ELIDEL; Meda Pharma, Luxembourg)	0.625–40	2.5–20	E	NE	E	TBD
Resveratrol	3.125–200	6.25–200	NE	E	E	TBD
Rhein	0.625– 40	N/A	NE	E	NE	Lack of potency
SC514	0.125–32	NE in nontoxic range	NE	NE	NE	Proprietary
Sulfasalazine^d^	12.5–1600	150–1600		E	E	Lack of potency
Sulindac	4.35–280	280	E	E	E	Lack of potency
Tacrolimus^e^ (FK506)	0.813–100	50	E	E	E	MMT/MCLT and MIA
Tranilast	0.125–8	1–8	NE	E	E	MIA
Triamcinolone acetonide	0.7–2000	250–500	E	E	E	Aristospan (TH) tested instead
Triamcinolone hexacetonide^e^	3.125–3750	62.5–3750	E	E	E	MMT/MCLT and MIA
Withaferin A^f^	0.25–17	4.25–17	E	E	E	MMT/MCLT model

*ACHP* 2-amino-6-(2-(cyclopropylmethoxy)-6-hydroxyphenyl)-4-(4-piperidinyl)-3-pyridinecarbonitrile, *E* effective nontoxic concentration that overlaps with other tested agents, *EGCG* epigallocatechin gallate, *FK506* tacrolimus, *IKK* inhibitor of nuclear factor κB kinase, *MIA* monoiodoacetic acid, *MMP* matrix metalloproteinase, *MMT/MCLT* medial meniscal tear/medial collateral ligament tear, *N/A* not applicable, *NF-κB* nuclear factor κ-light-chain-enhancer of activated B cells, *NE* not effective and nontoxic within the effective/nontoxic range for the other tested drugs, *SC514* selective reversible inhibitor of inhibitor of nuclear factor κB kinase 2, *TBD* to be determined, *TH* triamcinolone hexacetonide

The overlapping dose range that was effective and nontoxic is also shown. If effective in vitro, it is noted whether in vivo testing occurred and in which models. See Figs. 1, 2 and 3 and Additional file 1 for results in the specific assays

^a^Dose found to inhibit with minimal or no toxicity to synovium or cartilage. Values reflect overlapping range if agent was effective in more than one assay

^b^Used as a bone remodeling control; not tested <1.25 μM in the bone assay

^c^Fluocinolone was effective at much lower doses in both the bone and MMP-13 assays

^d^Effective in the MMP-13 and bone assays; at higher concentrations, this compound blocked NF-κB activity in the HeLa assay but also blocked expression of the Renilla plasmid luciferase. At lower concentrations, it was not effective against NF-κB

^e^Tacrolimus and TH were effective at much lower doses in the bone assay. For TH, lower doses may have been effective in the NF-κB assay but were not tested

^f^Because of the promising results, especially in the bone and MMP-13 assays, and in spite of its slight toxicity in the synovial and chondrocyte assays, Withaferin A was tested in the MMT/MCLT model

**Table 3:** Summary of the in vivo results using the MIA rat model

MIA model				Results					
	Trial 1: systemic delivery			Weight bearing (significant differences; h)				Histopathology (*n* = 3)^a^	
	Drug (daily)	Dose (s.c.) (mg/kg)	Number of animals	7 days	14 days	21 days	28 days	Cartilage	Bone
1	Vehicle saline	5	10				Term	4.7	Severe
2	Clonidine	0.1	10	1, 5, 24	1	1	1, 3	4.7	Severe
3	Fluocinolone	0.002	10			BL^b^, 1		3.0	Minimal
4	Morphine	6	10	1, 3, 5	1	BL, 1, 3	1, 3	N/A	N/A
1H	Vehicle Saline	5 ml/kg	3	Term	N/A	N/A	N/A	4.7	Severe
2H	Vehicle Saline	5 ml/kg	3	N/A	Term	N/A	N/A	5.0	Severe
3H	Vehicle Saline	5 ml/kg	3	N/A	N/A	Term	N/A	4.7	Severe
	Trial 2: systemic delivery (*n* = 10)			Digital Randall-Selitto (significant differences; h)				Histopathology (*n* = 3 or 4)	
	Drug (daily, unless indicated)	Dose (mg/kg)	Route (ml)	7 days	14 days	21 days	28 days	Cartilage matrix	Total joints
1	Vehicle saline	N/A	s.c. (5)			1, −5		4.5 ± 0.5	14.3 ± 1.1
2	Clonidine (weekly)^c^	0.1	s.c. (5)	1, 3, 5	1, 3, 5	3	1, 3, 5	3.7 ± 0.3	11.7 ± 0.9
3	Tacrolimus	0.3	i.p. (1)			Pretrt^d^		3.8 ± 0.6	11.5 ± 1.7
4	Tacrolimus	0.6	i.p. (1)			1		3.8 ± 0.6	10.3 ± 2.1
5	Curcumin	50	p.o. (5)	1		Pretrt^d^		5.0 ± 0.0	14.5 ± 0.3
6	Fluocinolone	0.01	s.c. (5)		5^e^			3.3 ± 0.8	9.3 ± 2.5
	Trial 3: articular delivery (*n* = 10)			Digital Randall-Selitto test (significant differences; h)				Histopathology (*n* = 3)	
	Drug (weekly)	Dose (μg)	Route (30 μl for i.a.)	7 days	14 days	21 days	28 days	Cartilage matrix	Total joints
1	Vehicle saline	N/A	i.a.					2.7 ± 1.5	5.7 ± 3.8
2	Clonidine	100 μg/kg	s.c.	1, 3, 5	1, 3	1, 3	1, 3	3.7 ± 0.9	10.7 ± 2.8
3	Clonidine	4.5	i.a.	1				3.0 ± 1.1	8.7 ± 3.3
4	Tacrolimus	0.03	i.a.					5.0 ± 0.0	13.3 ± 1.7
5	Fluocinolone	0.015	i.a.	1			−24	3.3 ± 1.2	10.3 ± 2.7
6	Meloxicam	100	i.a.	1	3			1.0 ± 0.0	3.0 ± 1.2
7	Tranilast	0.5	i.a.	1, 3	1	1		5.0 ± 0.0	14.3 ± 0.3
8	Triamcinolone H	150	i.a.	3	1	Pretrt^f^	−24	3.3 ± 0.9	8.3 ± 2.6

*BL* baseline, *i.a.* intraarticular, *i.p.* intraperitoneal, *p.o.*, per oral, *s.c.* subcutaneous delivery

The details of the related studies and results are provided in Additional file 1. Shown are hours after drug delivery where a statistically measurable effect (*p* ≤ 0.05) was observed on weight bearing or mechanical hyperalgesia compared with the pretreatment baseline of that day, unless noted otherwise. Negative values indicate decreasing of threshold (e.g., −24 = worse at 24 h). “Pretrt” refers to an effect on pain that was measurable before the dosing for that particular day

For histopathology, scores approach 0 with improvement. The femoral cartilage degeneration score and the three-zone sum of the tibial cartilage degeneration scores (mean of three levels) were summed to create a total cartilage degeneration score (shown). The mean osteophyte score for each joint was added to this value to create a total joint score with matrix. Additional measures of tibial cartilage, bone and synovial changes, and details of statistical analysis are provided in Additional file 1

^a^Groups 1–3 necropsy on day 29; group 1H necropsy on day 7, group 2H on day 14, and group 3H on day 21

^b^Significant difference in weight-bearing score on day 21 compared with vehicle control–treated rats

^c^Weekly clonidine showed significant effects on the pretreatment joint compression threshold compared with pretreatment vehicle alone, observed on days 7, 14, 21, and 28

^d^Treatment resulted in a significant increase in pre-treatment joint compression thresholds compared with pretreatment on day 7

^e^Significant decrease in joint compression threshold compared with vehicle controls; no effect compared with day 7 pretreatment baseline

^f^Significant decrease in joint compression threshold compared with day 7 pretreatment baseline

### MIA model

The first trial in the MIA model was used to test clonidine, fluocinolone, and morphine as well as to establish the histopathological progression of the model (Table 3; Additional file 1: Figs. S29–S39 and Tables S5–S7). Fluocinolone was found to be the most promising of the three agents in this trial. Not only did it inhibit disease progression, with animals showing a lower percentage of chondrocyte necrosis and proteoglycan degeneration across the tibial and femoral cartilage with minimal bone changes (Table 3; Additional file 1: Tables S5–S7), but it also significantly improved baseline and 1-h WB by 21 days (Additional file 1: Fig. S39). Clonidine, with its known characteristics as an analgesic, was found to be similar in efficacy to morphine when administered systemically daily (Additional file 1: Figs. S30–S37). It was thus used as a control for all subsequent trials.

**Fig. 4:**
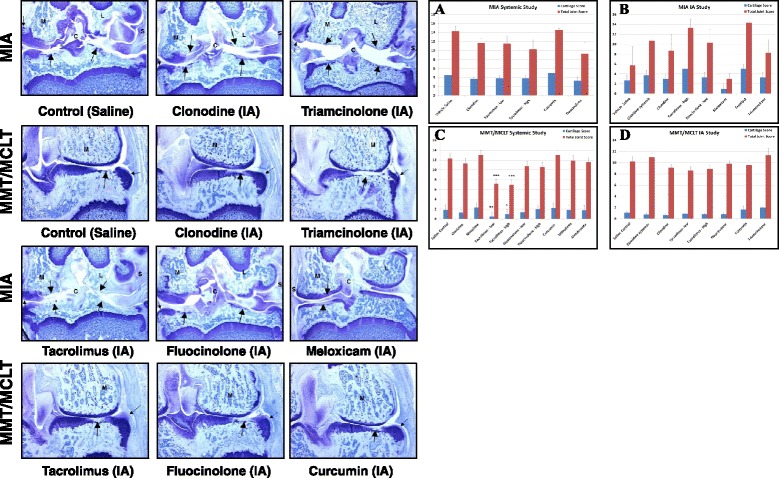
Representative frontal images from the intraarticular (IA) delivery studies in the monoiodoacetic acid (MIA; original magnification × 16) and medial meniscal tear/medial collateral ligament tear (MMT/MCLT; original magnification × 25) rat models (28 and 21 days postinjury, respectively). Shown are images from animals treated with saline, 4.5 mg of clonidine, 150 mg of triamcinolone, 30 ng of tacrolimus, 15 ng of fluocinolone, 100 mg of meloxicam, or 30 mg of curcumin. MIA: *M* medial, *L* lateral, *S* synovium, *large arrows* affected cartilage surface, *small arrows* osteophyte. MMT/MCLT: *M* marrow, *large arrows* affected cartilage surface, *small arrows* osteophyte. *Top right*: Comparisons of mean cartilage matrix damage and total joint scores with matrix (**a**–**d**). **a** Trial 2 MIA systemic study (*n* = 10 per group). **b** Trial 3 MIA IA study (*n* = 10 per group). **c** Trial 1 MMT/MCLT systemic study (*n* = 8). **d** Trial 2 MMT/MCLT IA study (*n* = 10). Toluidine blue–stained sections from knees of animals treated with test compounds were analyzed for proteoglycan and cartilage matrix loss, subchondral bone resorption, sclerosis, and osteophyte formation as well as synovitis (see Additional file 1). Femoral and tibial cartilage degeneration scores were summed for total cartilage scores (*blue*). Total joint scores also included osteophyte analysis (*red*)

In trial 2 (Table 3 and Fig. 4a; Additional file 1: Figs. S41–S49 and Tables S8–S19), fluocinolone was compared with clonidine, tacrolimus, and curcumin and mechanical hyperalgesia was used to measure differences in pain perception. Drug administration began on day 7. Owing to limited oral bioavailability, curcumin was administered by gavage. Unless otherwise noted, the hours shown in Table 3 (dRS column) were those when significant improvement in comparison with baseline was observed. As opposed to trial 1, daily subcutaneous administration of clonidine was better at treating mechanical hyperalgesia, and over a period of at least 5 h (Additional file 1: Figs. S41–S45), in spite of being administered immediately before testing. Clonidine also showed significant effects on the pretreatment joint compression thresholds (Additional file 1: Fig. S41), indicating a persistent effect. Tacrolimus, curcumin, and fluocinolone inconsistently treated mechanical hyperalgesia on days 7, 14, and 21, respectively (Table 3; Additional file 1: Figs. S46–S49). Rats treated with tacrolimus appeared healthy but consistently showed blood in the stool. When all time measures were considered, the cumulative pretreatment effects on pain behavior noted at day 21 for tacrolimus and fluocinolone were not observed on day 28. Histopathological analysis and comparison of total joint scores failed to show significant differences in joint deterioration (Table 3 and Fig. 4b; Additional file 1: Tables S8–S19). However, although cartilage matrix and total joint scores were not found to be significantly different, fluocinolone did block several measures of cartilage degeneration and bone resorption at 0.01 mg/kg (*p* < 0.03) (Additional file 1 Tables S16 and S17), consistent with its effects in vitro.

In the third MIA trial (Table 3 and Fig. 4; Additional file 1: Figs. S50–S54 and Tables S20–S35), IA clonidine, tacrolimus, fluocinolone, meloxicam, tranilast, and TH were compared with systemically delivered clonidine. Tranilast was included because it fared well in two of the in vitro assays and because it was shown to have anti-inflammatory and analgesic properties in a rheumatoid arthritis (RA) model [34]. It was also shown to suppress cyclooxygenase (COX)-2 and inducible nitric oxide synthase (iNOS) expression as well as to reduce prostaglandin E_2_ and iNOS-derived NO production in stimulated macrophages [35]. Doses were chosen on the basis of historical data and an estimation of the volume of the bursa–joint space. While none of the agents significantly impacted histopathological progression (Table 3; Additional file 1: Tables S20–S35), meloxicam, which was included because of its efficacy against NF-κB and approval for treating OA, performed best overall with relatively low cartilage matrix and total joint scores (Table 3 and Fig. 4; Additional file 1: Tables S28 and S29). Meloxicam significantly inhibited mechanical hyperalgesia, both at 7 and 14 days (Table 3; Additional file 1: Figs. S51 and S52). Dosing for meloxicam was based upon previous studies using an IA COX-2 inhibitor, parecoxib, in a rat anterior collateral ligament tear (ACLT) model [36]. Triamcinolone and tranilast also both significantly inhibited pain behavior at 7 and 14 days, but TH was, if anything, negatively effective at 21 and 28 days. Tranilast failed to positively impact histopathological measures (Additional file 1: Tables S30 and S31), so it was not tested in the MMT/MCLT model. Controls showed no to subacute synovitis with generally moderate fibrosis and variable findings following therapy (Additional file 1: Table S21). In this trial, only three animals from each group were analyzed histologically, which likely influenced the ability to discern differences. Representative images from six of the groups are shown in Fig. 4 and are compared with IA delivery in the MMT/MCLT model.

### MMT/MCLT model

In MMT/MCLT trial 1 (Table 4 and Fig. 4c; Additional file 1: Fig. S56 and Appendix), several agents were effective when delivered systemically, with morphine and clonidine most positively modifying WB asymmetry. The lower dose of tacrolimus, the highest dose of fluocinolone, and curcumin also significantly alleviated pain behavior (Table 4). Progression in this model has been described previously [25, 33]. Representative images for trial 1 are shown in the Additional file 1: Fig. S57. Tacrolimus (Table 4; Additional file 1: Tables S38 and S39) and fluocinolone (Table 5) both significantly inhibited changes in various measures of cartilage and bone, with tacrolimus demonstrating the most consistent and profound effects across all measures. Tacrolimus unfortunately led to blood in the stool of rats as well as loss of bone trabeculae, effects that could be deleterious with prolonged systemic administration. Both curcumin and withaferin were effective at alleviating WB asymmetry but were without positive effects on joint degeneration. For at least withaferin, this may in part be explained by the cell toxicity noted in the screening.

**Table 4:** Summary of in vivo results using the MMT/MCLT rat model

	MMT/MCLT model			Results					
	Trial 1: systemic delivery (*n* = 8)			Weight bearing (significant effects; h; *p* < 0.05)			Histopathology (*n* = 8)		
	Drug (daily)	Dose	Route (1 ml.)	7 days	14 days	21 days	Cartilage degeneration score		Total joint score
1	Saline control	n.a.	i.p.				1.83 ± 0.31		12.29 ± 0.62
2	Morphine	10 mg/kg	i.p.	1	1,3,5	3,5	2.33 ± 0.66		12.96 ± 0.89
3	Tacrolimus	0.3 mg/kg	i.p.	3	0,1,3,5,24	3	0.5 ± 0.15^a^		7.13 ± 0.69^b^
4	Tacrolimus	0.6 mg/kg	i.p.	–	1	–	0.96 ± 0.27^c^		6.96 ± 0.65^b^
5	Fluocinolone	0.005 mg/kg	i.p.	5	1,3	1	1.33 ± 0.14		10.75 ± 0.61
6	Fluocinolone	0.010 mg/kg	i.p.	1, 24	1,3,5,24	–	1.96 ± 0.77		10.54 ± 0.88
7	Clonidine	0.100 mg/kg	i.p.	1	1,3,5	3,5,24	1.29 ± 0.20		11.33 ± 0.55
8	Alendronate	10 μg/kg	i.p.	5	1	–	1.79 ± 0.60		11.55 ± 0.72
9	Curcumin	50 mg/kg	p.o.	–	0,1,3,5,24	0, 3	2.21 ± 0.60		12.96 ± 0.62
10	Withaferin	50 mg/kg	p.o.	1	3	–	1.83 ± 0.52		11.83 ± 0.61
	Trial 2: intraarticular delivery (*n* = 10)			Weight bearing (significant effects)			Histopathology (*n* = 10)		
	Drug (weekly)	Dose	Route (30 μl)	7 days	14 days	21 days	Synovium	Cartilage degeneration score	Total joint score
1	Saline control	n.a.	i.a.			–	–	1.07 ± 0.24	10.23 ± 0.85
2	Clonidine	100 μg/kg	s.c.	+/−	+/−	+/−	+/−	0.77 ± 0.28	11.03 ± 0.61
3	Clonidine	4.5 μg	i.a.	–	–	–	–	0.67 ± 0.23	9.13 ± 0.54
4	Triamcinolone H	0.15 mg	i.a.	–	–	–	+	2.03 ± 0.52	11.30 ± 1.21
5	Tacrolimus	15 ng	i.a.	–	–	–	–	0.87 ± 0.36	8.60 ± 0.63
6	Tacrolimus	30 ng	i.a.	–	–	–	–	0.8 ± 0.19	8.90 ± 0.66
7	Fluocinolone	15 ng	i.a.	–	–	–	–	0.8 ± 0.25	9.80 ± 0.51
8	Curcumin	30 μg	i.a.	–	–	–	–	1.63 ± 0.61	9.57 ± 0.91

*n.a.* not applicable, *i.a.* intraarticular, *s.c.* subcutaneous, *MMT/MCLT* medial meniscal tear/medial collateral ligament tear. −24 = worse at 24 h; “Pretrt” refers to an effect on pain that is measurable before dosing

The details of the related studies and results are provided in Additional file 1. In trial 1, drug administration was prophylactic in that test articles were administered subcutaneously daily for 3 weeks beginning 1 day before surgery. In trial 2, drug administration was therapeutic in that drugs were administered weekly beginning 1 week after surgery. For weight bearing, shown are hours after drug delivery when a statistically measurable effect (*p* ≤ 0.05) was observed compared with the pretreatment baseline of that day, unless noted otherwise. With regard to histopathological measurements, medial femur cartilage degeneration and total joint score are noted. The scores approach 0 with improvement. The mean osteophyte score for each joint was added to the total cartilage degeneration score to create a total joint score. Additional measures of tibial cartilage, bone and synovial changes as well as details of statistical analysis are provided in Additional file 1

^a^ *p* ≤ 0.005 compared with vehicle alone

^b^ *p* ≤ 0.001 compared with vehicle alone

^c^ *p* ≤ 0.05 compared with vehicle alone

**Table 5:** Histological analysis in the MMT/MCLT studies (fluocinolone)

Animal	Knee	Medial tibial cartilage degeneration score^a^				Tibial cartilage degeneration width		Depth ratio, any matrix change^b^		Medial tibial osteophytes		Medial femoral cartilage degeneration score^a^	Bone score	Total joint score without femur	Total joint score
		Three-zone total	Zone 1 (Outside)	Zone 2 (Middle)	Zone 3 (Inside)	Total^c^ (μm)	Sig^d^ (μm)	Mean	Zone 2	Score^e^	Measure (μm)				
1	R	6.33	4.33	2.00	0.00	1133.33	633.33	0.34	0.05	3.00	413.33	2.00	4.00	9.33	11.33
2	R	4.67	3.33	1.33	0.00	1000.00	433.33	0.28	0.06	1.33	280.00	3.00	1.00	6.00	9.00
3	R	7.00	4.67	2.33	0.00	1133.33	766.67	0.39	0.16	3.00	426.67	2.33	2.00	10.00	12.33
4	R	7.67	4.67	2.67	0.33	1600.00	800.00	0.42	0.31	4.67	586.67	3.00	3.00	12.33	15.33
5	R	5.33	4.00	1.33	0.00	1266.67	566.67	0.33	0.05	3.00	400.00	1.33	3.00	8.33	9.67
6	R	6.00	4.00	2.00	0.00	1100.00	666.67	0.35	0.10	2.67	380.00	2.00	3.00	8.67	10.67
7	R	4.67	3.00	1.67	0.00	1400.00	466.67	0.29	0.13	2.00	333.33	1.00	3.00	6.67	7.67
8	R	5.00	3.00	2.00	0.00	1066.67	433.33	0.32	0.03	2.33	333.33	1.00	2.00	7.33	8.33
Mean		5.83	3.88	1.92	0.04	1212.50	595.83	0.34	0.11	2.75	394.17	1.96	2.63	8.58	10.54
SE		0.39	0.24	0.16	0.04	70.69	51.35	0.02	0.03	0.34	32.50	0.28	0.32	0.71	0.88
*t* test to G1		0.19	0.23	0.23	1.00	0.27	**0.05**	0.46	0.18	**0.02**	**0.01**	0.77	0.11	**0.04**	0.12
Percentage		0.11	0.10	0.13	0.00	0.08	0.18	0.06	0.41	0.30	0.23	−0.07	0.19	0.18	0.14

SE, standard error; Sig, significant; G1, group 1

Shown are results of the analysis of toluidine-stained sections from three levels within the joints of a group of eight animals treated systemically with 10 μg/kg fluocinolone for 21 days. Note that the cartilage degeneration and total joint scores depicted in Table 4 were not statistically different compared with the control group treated with saline (not shown). Several other measures were significantly different (*t* test results in boldface type). To view similar data for all the groups in both models, see Additional file 1

^a^Cartilage degeneration score = depth (0–5) for each of three zones, then summed (mean of three-step section)

^b^Mean lesion depth in micrometers versus depth to tidemark in center of zone in the tibial plateau (mean of three-step section)

^c^Width of any cartilage lesion (mean of three-step section)

^d^Width of cartilage degeneration extending >50 % of total thickness (mean of three-step section)

^e^Osteophyte scores 1 = small up to 299 μm, 2 = medium 300–399 μm, 3 = large 400–499 μm, 4 = very large 500–599 μm, 5 = very large >600 μm

Several drugs were tested via IA injection in MMT/MCLT trial 2 (Table 4; Additional file 1: Figs. S58–S59, Tables S46–S53). Only systemic clonidine was found to be effective via WB. Clonidine was also effective at modifying WB when given intraperitoneally (Table 4), but it was not effective as a subcutaneous bolus dosed once weekly before incapacitance testing (s.c.) (Table 4). Similarly, IA administration of 4.5 μg of clonidine once weekly also failed to modify WB asymmetry. In spite of this, histopathological analysis revealed a positive effect on at least one measure of cartilage degeneration (Additional file 1: Fig. S58 and Table S48) with IA clonidine and on medial tibial osteophytes when delivered systemically (Additional file 1: Table S47).

In this same trial with the MMT/MCLT model, tacrolimus and curcumin did not positively affect total joint and cartilage scores, but they had a significantly positive impact on some measures of cartilage degeneration compared with vehicle alone (Additional file 1: Table S50–S51 and S53). For example, animals treated with 15 ng of IA tacrolimus once weekly showed significantly decreased cartilage degeneration scores for zone 2 of the medial tibia (42 %). The width of total cartilage degeneration was also significantly decreased (12 %). Animals treated with 30 μg of IA curcumin once weekly also had significantly decreased cartilage degeneration scores in zone 2 of the medial tibia (40 %). The depth ratio of any matrix change in zone 2 was similarly significantly decreased (69 %). In contrast, TH-treated animals showed very severe cartilage loss over several measures compared with vehicle alone (Fig. 4). The significantly lower osteophyte scores served to offset this loss, yielding nonsignificant changes in total joint scores (Additional file 1: Table S49). Representative histological images from six of the groups are shown in Fig. 4, and images of the remaining groups are provided in Additional file 1: Fig. S58. The synovium of MMT/MCLT animals at 21 days showed normal fibrous repair with minimal to mild synovitis that consistently improved with TH treatment (Additional file 1: Fig. S59).

### μCT analysis

**Fig. 5:**
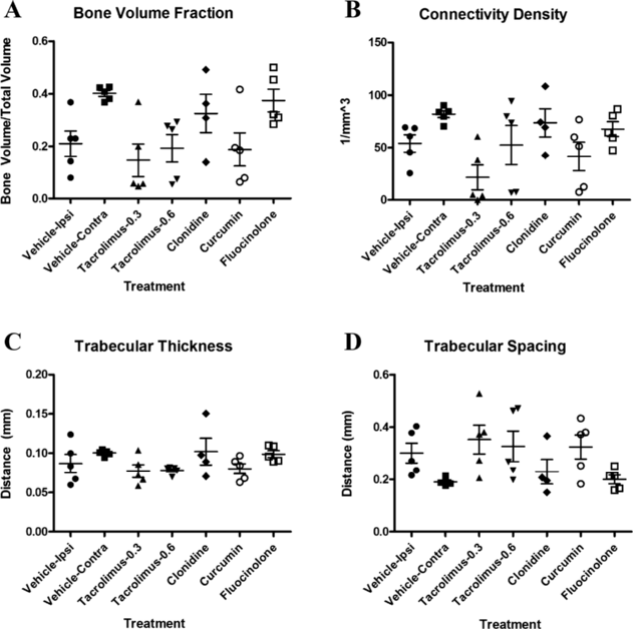
Micro computed tomographic (μCT) analysis of knee joints from rats treated with monoiodoacetic acid (MIA) in trial 2 (*n* = 5). Vehicle ipsilateral is the injured joint, and vehicle contralateral is the untreated control joint, of the vehicle-treated animal. Comparison of joints analyzed by μCT from five animals of each group in MIA trial 2. Note that only four knees from the clonidine group were analyzed. **a** Relative bone volume fraction (bone volume/total volume). **b** Connectivity density (1/mm^3^). **c** Trabecular thickness (distance in millimeters). **d** Trabecular spacing (distance in millimeters). One-way analysis of variance was used to look for differences between groups. If *p* ≤ 0.05, then Bonferroni’s post hoc test was performed to identify significant differences between groups

The drug treatments did not significantly affect the parameters analyzed by μCT, although the trends were somewhat consistent with the histopathological findings (Fig. 5) and could potentially prove to be significant in a larger study.

## Discussion

Chronic OA is depicted by a self-perpetuating cycle whereby loss of cartilage leads to altered joint biomechanics and an instability that furthers nerve damage, inflammation, boney changes, and resultant pain [10]. The development of pain is strongly correlated with formation of osteophytes, changes in subchondral bone, joint effusion, and inflammation [37, 38]. Both models used in this set of studies showed significant osteophyte formation that likely contributed to pain (Fig. 4, *small arrows*). Although normal articular cartilage is avascular and aneural, sensory nerves found in vascular channels within the cartilage in mild and severe OA may contribute to tibiofemoral pain [21]. Perivascular and free nerve fibers, as well as nerve trunks, are also observed in subchondral bone and osteophytes [21, 39].

Although it is not clear which targets are most important in OA, NF-κB, bone remodeling, and MMP-13 have been identified to play key roles in progression [11, 40–42], and all continue to be of interest in the pathogenesis of OA [7, 12, 43]. We chose to test existing, well-characterized drugs and nutraceuticals [43] proposed to be potential OA therapeutics (Table 1).

In the relatively severe MIA model, rapid degeneration of joint cartilage and disruption of the underlying subchondral bone ensue from the MIA-induced death of chondrocytes, and cells in the outer margins proliferate, often forming large osteophytes [23, 44]. In the presence of normal load-bearing, there is a progressive loss of proteoglycan, fibrillation, collapse of collagenous matrix, and resorption of subchondral bone [45], with concomitant increases in aggrecanases, MMPs, and inflammatory mediators [46]. IA MIA may also significantly injure dorsal root ganglion cells, including those that innervate targets outside the knee joint, such as hind paw skin [47].

In contrast to the MIA model, transection of the medial collateral ligament and medial meniscus of the femorotibial joint in the MMT/MCLT rat [20] results in rapidly progressive degeneration characterized by chondrocyte and proteoglycan loss, fibrillation, osteophyte formation, and chondrocyte cloning [25, 33, 48]. Damage in this model may best mirror posttraumatic human OA [26]. Its more severe and rapidly progressing phenotype compared with the ACLT model potentially allows for detection of smaller differences between treatments [20]. Broad-spectrum MMP inhibitors are effective in this model [48], which also has relevance to human OA, where MMPs play an active role [49]. While broad-spectrum MMP inhibitors have failed in clinical trials because of their dose-limiting musculoskeletal side effects [50], attention to MMP-13 has been revived by the development of targeted inhibitors [15, 51]. MMP-knockout mice have further demonstrated the importance of MMP-13 in cartilage remodeling [49].

Owing to the complex nature of OA, one model does not fully recapitulate all its characteristics [12, 25, 26]. It seems evident that therapeutics are needed that address such complexity. Indeed, the combination of the selective COX-2 inhibitor meloxicam with pregabalin (a calcium channel α_2_δ ligand developed to manage neuropathic pain) was found to most effectively treat knee pain in patients with OA [19]. The MIA and MMT/MCLT models differ significantly, although both display synovial inflammation, chondropathy, and osteophytosis [26]. Inflammation and osteophyte scores appear to be more pronounced in the MMT/MCLT model [26] (see also Fig. 4). In the MIA model, both inflammatory and protease mediator gene clusters are active, including interleukin (IL)-1β, TNF-α, IL-15, IL-12, chemokines, and NF-κB, and all have also been identified as controlling the progression of cartilage destruction [46]. Upregulation of asporin and downregulation of transforming growth factor β, Sry-related high-mobility group box 9 (SOX-9), IGF, and connective tissue growth factor have been shown to be critical for the suppression of matrix synthesis and chondrocytic anabolic activities, which collectively contribute to the progression of cartilage destruction in the MIA model [46].

Fluocinolone was fairly effective overall, inhibiting all three targets in vitro, mildly retarding degeneration in vivo in both models, and consistently demonstrating positive effects on pain behavior in the MMT/MCLT model. Fluocinolone had a significant impact on several measures of cartilage damage and bone resorption in both MIA trial 2 and MMT/MCLT trial 1, although it did not significantly affect total joint scores (Tables 3 and 4; Additional file 1). Owing to the long-term side effects associated with systemic use in humans, fluocinolone would have clinical potential only via local delivery similar to that used with intraocular delivery devices [52]. In our initial local delivery studies, with the doses and frequency of dosing used here, fluocinolone did not prove as effective.

Triamcinolone, also a corticosteroid, provides some clinical benefit in OA and other arthritis patients for up to 6 months following IA delivery [53, 54]. TH was more effective than the acetonide at inhibiting NF-κB, but a clear dose–response relationship was not always observed, potentially due to solubility limitations or to an insufficient sensitivity of the assay. In spite of efficacy in humans, IA TH did not result in improvement in WB in the MMT/MCLT model. It is possible that this was related to the dose used. IA TA attenuated WB asymmetry and distal allodynia to control levels in the MMT/MCLT model in a study by Mapp et al. [26] when the treatment was initiated at 14 days; however, the dose per knee was higher than our 150 μg at 1 mg. In contrast, distal allodynia was unaltered in their MIA model [26], while sporadically positive effects on dRS were seen in our MIA studies (Table 3). In our hands, TH negatively affected several of the cartilage and bone scores measured histologically (Additional file 1: Table S49). TH showed efficacy against in vitro targets, so it is possible that these negative findings were due to a block of pain feedback resulting in an increased use of the joint and worsening of the disease (Fig. 4). It is also possible that TH affected joint biology, leading to increased deterioration. This could be further tested by treating control joints with TH. As in our study with TH, TA has been shown to reduce synovial inflammation in both the MIA and MMT/MCLT models [26].

When delivered via IA injection following arthroscopy, clonidine has been found to offer longer-lasting analgesia than morphine [53, 55, 56]. In our studies, systemic clonidine performed as well as, and often better than, morphine in treating pain behavior. Unexpectedly, IA clonidine, similar to IA TH, did not consistently relieve pain behavior in spite of efficacy in humans [29, 37]. IA clonidine in the MIA model had significant effects on dRS at 1 h on day 7 and no significant positive effects in the MMT/MCLT model. However, higher IA concentrations of clonidine may be required to inhibit pain targets in the rat [57]. Because clonidine elicits its antinociceptive effect on the central nervous system, the decreased systemic exposure with local delivery may explain the failure of IA clonidine to inhibit pain in these studies. Although systemically delivered clonidine was effective as an analgesic, it was not consistent in slowing joint degeneration and inhibited only some measures of histological progression in the MMT/MCLT model, including development of osteophytes (see Additional file 1). Clonidine also inhibited osteoclastogenesis in vitro at high concentrations (Fig. 2b).

In the MMT/MCLT model, none of the IA agents significantly affected WB, and all sporadically increased pain behavior compared with the controls. It is possible that the injection itself caused pain, although the average response of dRS and WB of animals that received IA saline did not show this. It has been shown, in fact, that IA saline injections decrease lesion severity in the MMT/MCLT model, and the degree is directly related to frequency and timing of injections postsurgery [58]. (Joint lavage in humans has shown mixed results, however [59].) Doses were chosen on the basis of other studies [52, 60] or were scaled down from concentrations used clinically [53, 55]. For some drugs, such as clonidine, central pain targets exist outside the synovial space [56], and the blood levels associated with IA delivery may not have been sufficient to affect these targets.

In spite of the fact that inhibition of COX-2 has been demonstrated to block TNF activation of NF-κB [61], meloxicam was relatively ineffective in our in vitro assay. This may be the result of NF-κB pathway differences between cell types. In the MIA model, however, IA meloxicam significantly increased joint compression thresholds on days 7 and 14 and had minor, albeit insignificant, positive effects on histopathology (Fig. 4). Several NSAIDs have been shown to block mechanical hyperalgesia in the MIA model (ALGOS 171.3; 11/16/2008SFN), and one of these, rofecoxib, also blocks both nociceptive and neuropathic pain behavior in the MMT/MCLT model [62]. Although not tested in the MMT/MCLT rats, IA meloxicam (0.25 mg) has been shown to inhibit cartilage degeneration and improve nociception in an ACLT model [63]. Interestingly, in a recent clinical study reported by Ohtori et al. [19], IA meloxicam failed to relieve pain unless delivered with pregabalin.

Tacrolimus, a calcineurin inhibitor and RA therapeutic, was tested because of its effects on NF-κB [64] and its ability to inhibit bone remodeling related to RANKL activation of osteoclasts [65]. Tacrolimus was a top inhibitor of bone matrix resorption in vitro and significantly inhibited histological progression in the MMT/MCLT model (Additional file 1: Tables S38 and S39), although there were only minor improvements with IA delivery (Additional file 1: Tables S50 and S51). At the lower dose, tacrolimus also positively influenced WB asymmetry in the MMT/MCLT model. Tacrolimus had a slightly negative impact on bone and cartilage measures in the MIA model when delivered intraarticularly (Additional file 1: Tables S24 and S25), although not on total bone and joint scores, and this was not seen when it was given systemically (Additional file 1: Tables S10–S13). Tacrolimus had minor positive effects on mechanical hyperalgesia in the MIA model when delivered systemically (Additional file 1: Figs. S46 and S47). It remains possible that tacrolimus might be a useful therapeutic in clinical OA. However, the intestinal side effect noted and the immunosuppressive effects previously described clinically [65] suggest that locally delivered tacrolimus might be necessary, possibly with supplementary pain therapy.

Analysis of MIA joints by μCT showed no statistically significant differences between groups, although positive trends were observed with clonidine and fluocinolone (Fig. 5). As noted here, in severe human OA, changes in subchondral bone are observed in the medial tibial compartment, where the relative bone volume and trabecular thickness are less than in controls, and the structural model index and trabecular spacing are greater. These changes typically indicate an increase in bone turnover [66]. Increased subchondral plate thickness, trabecular thickness, and separation have also been documented in the MIA model, with trabecular number decreased compared with control tibiae [67].

## Conclusions

Not surprisingly, our results varied between the MIA and the MMT/MCLT models, underscoring differences in mechanisms of degeneration and pain that have been confirmed by others [25, 26]. It is challenging to measure pain in small animals and unclear whether the pain measured is similar to human OA pain. For this reason, histopathological analysis is considered the gold standard for measurement of disease progression [25]. Several agents were at least moderately effective at modifying histopathological progression, including tacrolimus and fluocinolone. Considering pain relief and improved histopathology, systemically delivered tacrolimus performed best in the MMT/MCLT model. Clonidine, used as a pain control in place of morphine, performed fairly effectively in treating pain in both models.

## Additional file

Additional file 1: **Supplementary material includes additional in vitro and in vivo methods, additional in vitro and in vivo results, and individual animal data.** A table of contents is presented at the beginning of the document for ease of navigation. (PDF 11370 kb)

## Abbreviations

ACHP: 2-amino-6-(2-(cyclopropylmethoxy)-6-hydroxyphenyl)-4-(4-piperidinyl)-3-pyridinecarbonitrile
ACLT: anterior collateral ligament tear
ADAMTS: a disintegrin and metalloproteinase with thrombospondin motif
AGE: advanced glycation end product
ANOVA: analysis of variance
ATCC: American Type Culture Collection
BL: baseline
CORM-2: carbon monoxide-releasing molecule 2
COX-2: cyclooxygenase-2
DMEM: Dulbecco’s minimal Eagle’s medium
DMSO: dimethyl sulfoxide
dRS: digital Randall-Selitto test
E: effective nontoxic concentration that overlaps with other tested agents
ECHODIAH: Evaluation of the Chondromodulating Effect of Diacerein in Osteoarthritis of the Hip
EGCG: epigallocatechin gallate
FBS: fetal bovine serum
FDA: U.S. Food and Drug Administration
FK506: tacrolimus
FK520: ascomycin
GAG: glycosaminoglycan
HA: hyaluronic acid
HIF-1α: hypoxia-inducible factor 1α
IA: intraarticular
IACUC: Institutional Animal Care and Use Committee
IC_50_: concentration at which the response is reduced by half
IGF: insulin-like growth factor
IκΒ-α: inhibitor of nuclear factor κB
IKK: inhibitor of nuclear factor κB kinase
IL: interleukin
iNOS: inducible nitric oxide synthase
i.p.: intraperitoneal
JNK: c-Jun N-terminal kinase
MAPK: mitogen-activated protein kinase
MIA: monoiodoacetic acid
micro-CT or μCT: micro computed tomography
MMP: matrix metalloproteinase
MMT/MCLT: medial meniscal tear/medial collateral ligament tear
M_r_: molecular weight
N/A: not applicable
NF-κB: nuclear factor κ-light-chain-enhancer of activated B cells
NE: not effective and nontoxic within the effective/nontoxic range for the other tested drugs
NSAID: nonsteroidal anti-inflammatory drug
OA: osteoarthritis
PGE: prostaglandin E
Pretrt: pretreatment (measurable before dosing)
RA: rheumatoid arthritis
RANK: receptor activator of NF-κB RANKL, receptor activator of nuclear factor κB ligand
ROI: region of interest
SC514: selective reversible inhibitor of inhibitor of nuclear factor κB kinase 2
SEM: standard error of the mean
SOX-9: Sry-related high-mobility group box 9
*t*_1/2_: half-life
TA: triamcinolone acetonide
TBD: to be determined
TGF-β: transforming growth factor β
TH: triamcinolone hexacetonide
Th1 or Th2: Th, helper T immune response-related cell
TNF-α: tumor necrosis factor α
TPCA: 5-(p-fluorophenyl)-2-ureido]thiophene-3-carboxamide
WB: Weight bearing
