# Supplementary material for: Randomized controlled studies on the efficacy of antiarthritic agents in inhibiting cartilage degeneration and pain associated with progression of osteoarthritis in the rat
Source: Arthritis Res Ther. 2016 Jan 21;18:24. doi: 10.1186/s13075-016-0921-5 (PMC4721142; doi:10.1186/s13075-016-0921-5)
Supplement: Supplementary file 1 — Supplementary material includes additional in vitro and in vivo methods, additional in vitro and in vivo results, and individual animal data. A table of contents is presented at the beginning of the document for ease of navigation. (PDF 11370 kb) [file 13075_2016_921_MOESM1_ESM.pdf]

SUPPLEMENT

Randomized and controlled studies on the efficacy of anti-arthritic agents in inhibiting cartilage degeneration and pain associated with progression of osteoarthritis in the rat.

Erica M. TenBroek[1*], Laurie Yunker[1], Mae Foster Nies[1], and Alison M. Bendele[2]

[1]These studies were conducted at Medtronic Inc., Ventures and New Therapies, Therapy, Research and Development 710 Medtronic Parkway, Fridley, MN 55432; [2]Bolder BioPATH, Inc. 5541 Central Ave Suite 160, Boulder, Colorado, 80301.

*Author may be contacted through email at tenbre1@comcast.net

Table of Contents

I. Supplementary Methods 7

In Vivo Functional Testing 7

Monoiodoacetoacetate Model Trial 1 7

Monoiodoacetoacetate Model Trial 2 8

Monoiodoacetoacetate Model Trial 3 9

Meniscal Tear Model Trial 1 9

Meniscal Tear Model Trial 2 9

Histopathology 10

Monoiodoacetoacetate Model 10

Medial Meniscal Tear Model 11

Micro CT 13

II. Supplementary Results 16

In Vitro NF-κB Activity 17

In Vitro Osteoclastogenesis 17

In Vitro MMP-13 Activity 27

In Vitro Synovial Toxicity 36

In Vitro Chondrocyte Toxicity 45

In Vitro Hela Cell Drug Toxicity Testing 47

Toxicity and Efficacy: Comparison of Test Results 48

In Vivo Testing of Osteoarthritic Animals 52

MIA Model Functional Assessment 55

MIA Model Histopathology Results 70

MMT/MCLT Model Functional Assessment 98

MMT/MCLT Model Histopathology Results 104

Appendix – Individual Animal Data……………………………………………………………… ..…. 118

Tables

Table 1 – Example of a histopathology report for an individual animal. 15

Table 2 – Example of calculated histopathological degeneration for an individual animal 15

Table 3- Drug toxicity measured using a CTG assay and HeLa cells. 50

Table 4. Comparison of screening results for various drugs. 51

Table 5. Percent area chondrocyte necrosis / proteoglycan degeneration MIA Trial 1 70

Table 6. Average percent area chondrocyte necrosis / proteoglycan degeneration MIA Trial 1 70

Table 7. Unmasked individual animal histopathological data for each treatment in MIA Trial 1 71

Table 8. Histopathological analysis of proteoglycan and matrix loss in Vehicle Controls in MIA Trial 2. 79

Table 9. Histopathological analysis of cartilage, bone, and synovium in Vehicle Controls in MIA Trial 2. 79

Table 10. Histopathological analysis of proteoglycan and matrix loss in Tacrolimus-treated animals in MIA Trial 2. 79

Table 11. Histopathological analysis of cartilage, bone, and synovium in Tacrolimus-treated animals in MIA Trial 2. 80

Table 12. Histopathological analysis of proteoglycan and matrix loss in Tacrolimus-treated animals in MIA Trial 2. 80

Table 13. Histopathological analysis of cartilage, bone, and synovium in Tacrolimus-treated animals in MIA Trial 2. 80

Table 14. Histopathological analysis of proteoglycan and matrix loss in Curcumin-treated animals in MIA Trial 2. 80

Table 15. Histopathological analysis of cartilage, bone, and synovium in Curcumin-treated animals in MIA Trial 2. 81

Table 16. Histopathological analysis of proteoglycan and matrix loss in Fluocinolone-treated animals in MIA Trial 2. 81

Table 17. Histopathological analysis of cartilage, bone, and synovium in Fluocinolone-treated animals in MIA Trial 2. 81

Table 18. Histopathological analysis of proteoglycan and matrix loss in Clonidine-treated animals in MIA Trial 2. 82

Table 19. Histopathological analysis of cartilage, bone, and synovium in Clonidine-treated animals in MIA Trial 2 82

Table 20. Histopathological analysis of proteoglycan and matrix loss in Vehicle Controls. 82

Table 21. Histopathological analysis of cartilage, bone, and synovium in Vehicle Controls 83

Table 22. Histopathological analysis of proteoglycan and matrix loss in Clonidine-treated animals. 83

Table 23. Histopathological analysis of cartilage, bone, and synovium in Clonidine-treated animals. 84

Table 24. Histopathological analysis of proteoglycan and matrix loss in Tacrolimus-treated animals. 84

Table 25. Histopathological analysis of cartilage, bone, and synovium in Tacrolimus-treated animals. 85

Table 26. Histopathological analysis of proteoglycan and matrix loss in Fluocinolone-treated animals. 85

Table 27. Histopathological analysis of cartilage, bone, & synovium in Fluocinolone-treated animals. 86

Table 28. Histopathological analysis of proteoglycan and matrix loss in Meloxicam-treated animals. 86

Table 29. Histopathological analysis of cartilage, bone, and synovium in Meloxicam-treated animals. 86

Table 30. Histopathological analysis of proteoglycan and matrix loss in Tranilast-treated animals. 87

Table 31. Histopathological analysis of cartilage, bone, and synovium in Tranilast-treated animals. 87

Table 32. Histopathological analysis of proteoglycan and matrix loss in Triamcinolone Hexacetonide-treated animals. 88

Table 33. Histopathological analysis of cartilage, bone, and synovium in Triamcinolone Hexacetonide-treated animals. 88

Table 34. Histopathological analysis of proteoglycan and matrix loss in Clonidine-treated animals. 88

Table 35. Histopathological analysis of cartilage, bone, and synovium in Clonidine-treated animals. 89

Table 36. Histopathology of animals treated with vehicle alone (Group 1; Systemic delivery) 105

Table 37. Histopathology of animals treated with morphine (Group 2; Systemic delivery) 106

Table 38. Histopathology of animals treated with Tacrolimus (Group 3; Systemic delivery low dose) 106

Table 39. Histopathology of animals treated with Tacrolimus (Group 4; Systemic delivery high dose) 107

Table 40. Histopathology of animals treated with fluocinolone (Group 5; Systemic delivery low dose) 107

Table 41. Histopathology of animals treated with fluocinolone (Group 6; Systemic delivery high dose) 108

Table 42. Histopathology of animals treated with clonidine (Group 7; Systemic delivery) 108

Table 43. Histopathology of animals treated with Alendronate (Group 8; Systemic delivery) 109

Table 44. Histopathology of animals treated with Curcumin (Group 9; Oral delivery) 109
Table 45. Histopathology of animals treated with Withaferin (Group 10; Oral delivery) 110
Table 46. Histopathology of animals treated with saline (Group 1; IA delivery) 113
Table 47. Histopathology of animals treated with clonidine (Group 2; SC delivery) 113
Table 48. Histopathology of animals treated with clonidine (Group 3; IA delivery) 114
Table 49. Histopathology of animals treated with Triamcinolone Hexacetonide (Group 4; IA delivery) 114
Table 50. Histopathology of animals treated with low dose Tacrolimus (Group 5; IA delivery) 115
Table 51. Histopathology of animals treated with high dose Tacrolimus (Group 6; IA delivery) 115
Table 52. Histopathology of animals treated with Fluocinolone (Group 7; IA delivery) 116
Table 53. Histopathology of animals treated with Curcumin (Group 8; IA delivery) 116
Table 54. Experimental Design and Key for MIA Trial 1. 118
Table 55. Experimental Design and Key for MIA Trial 2. 118
Table 56. Experimental Design and Key for MIA Trial 3. 118
Table 57. Experimental Design and Key for MMT/MCLT Trial 1. 119
Table 58. Experimental Design and Key for MMT/MCLT Trial 2. 119
Table 59. Individual animal WB score data for MIA Trial 1 Day 7. Scores calculated as indicated in the methods. 120
Table 60. Individual animal WB score data for MIA Trial 1 Day 14. 121
Table 61. Individual animal WB score data for MIA Trial 1 Day 21. 122
Table 62. Individual animal WB score data for MIA Trial 1 Day 28. 123
Table 63. Individual animal joint compression data for MIA 2 Day 7. 124
Table 64. Individual animal joint compression data for MIA 2 Day 14. 125
Table 65. Individual animal joint compression data for MIA 2 Day 21. 126
Table 66. Individual animal joint compression data for MIA 2 Day 28. 127
Table 67. Individual joint compression data for MIA Trial 3 Day 7. 128
Table 68. Individual joint compression data for MIA Trial 3 Day 14. 129
Table 69. Individual joint compression data for MIA Trial 3 Day 21. 130
Table 70. Individual joint compression data for MIA Trial 3 Day 28. 131
Table 71. Individual animal data for MMT/MCLT Trial 1 Baseline and Day 7 data 132
Table 72. Individual animal data for MMT/MCLT Trial 1 Day 14 data 135
Table 73. Individual animal data for MMT/MCLT Trial 1 Day 21 data 138
Table 74. Individual animal weight bearing data for the MMT/MCLT Trial 2 Day 7 Data 142
Table 75. Individual animal data for MMT/MCLT IA Trial 2 Day 14 data 146
Table 76. Individual animal data for MMT/MCLT IA Trial 2 Day 21 data 150

Figures

Figure 1. MIA Trial Timeline 7
Figure 2. MMT/MCLT Trial Timeline 10
Figure 3. Sulfasalazine effects on the different reporter plasmids 18
Figure 4. NFkB activity in HeLa cells using the dual reporter system. 19

Figure 5 - Measurement of drug effects on osteoclastogenesis and osteoclast activity. 22

Figure 6. Effect of sulfasalazine on osteoclast differentiation vs. mature activity. 23

Figure 7 – Effects of different drugs on osteoclastogenesis using the Osteologic assay. 24

Figure 8 - Effects of different drugs on osteoclastogenesis using the Osteologic assay. 25

Figure 9 - Effects of different drugs on osteoclastogenesis using the Osteologic assay. 26

Figure 10 - Effects of Epigallocatechin Gallate (EGCG), Tacrolimus, Triamcinolone Hexacetonide and 3-O-Acetyl-11-keto-β-Boswellic Acid (Boswellia) on MMP-13 levels. 27

Figure 11 - Effects of Epigallocatechin Gallate (EGCG), Dimethyl Sulfoxide (DMSO), Clonidine, Fluocinolone and 3-O-Acetyl-11-keto-β-Boswellic Acid (Boswellia) on MMP-13 levels 28

Figure 12 - Effects of BAY 11-7082, BMS-345541, Alendronate and Meloxicam on MMP-13 levels. 29

Figure 13 - Effects of IKK-2 Inhibitor VI, IKK-2 Inhibitor VIII, CORM-2, Rhein, GM6001, Curcumin and SC514 on MMP-13 levels. 30

Figure 14 - Effects of Diacerin, IKK-2 Inhibitor V, Withaferin A, Triamcinolone Hexacetonide, Curcumin 14 and Amrinone on MMP-13 levels. 31

Figure 15 - Effects of Triamcinolone Acetonide, NFkB Activation Inhibitor IV, Sulindac, Sulfasalazine, Tranilast and Resveratrol on MMP-13 levels. 32

Figure 16 - Effects of BAY 11-7082, Ketorolac, Clonidine, Resveratrol, and Fluocinolone on MMP-13 levels. 33

Figure 17 - Effects of BMS-345541 (IKK-2 Inhibitor III), IKK-2 Inhibitor VI and CORM-2 on MMP-13 levels. 34

Figure 18 - Effects of Pimecrolimus, Ethanol, Ascomycin, Boswellia, SC514 and Triamcinolone Acetonide on MMP-13 levels. 35

Figure 19 – Effects of Triamcinolone Hexacetonide, Sulindac, Meloxicam, Alendronate, Amrinone and Sulfasalazine on viability of synovial sarcoma cells (SW982) +/-TNF-alpha 36

Figure 20 – Effects of BMS-345541, BAY 11-7082, GM6001, Tranilast, CORM-2, Triamcinolone Acetonide, and IKK2 Inhibitor V on the viability of synovial sarcoma cells (SW982) +/-TNF-alpha. 37

Figure 21 – Effects of Tranilast, Resveratrol, Verapamil, CORM-2, Sulindac, and Sulfasalazine on the viability of SW982 cells +/- TNF alpha. 38

Figure 22 - Effects of GM6001, NFkB Activation Inhibitor IV, BMS-345541 and Triamcinolone Acetonide on the viability of SW982 cells +/- TNF-alpha. 39

Figure 23 – The effects of Tacrolimus, Boswellia, EGCG, Fluocinolone, IKK2 Inhibitor VI, Withaferin A and Curcumin on the viability of SW982 cells +/- TNF-alpha. 40

Figure 24 – The effects of Curcumin-14, Clonidine, IKK2 Inhibitor VIII, Amrinone and DMSO on the viability of SW982 cells +/- TNF alpha. 41

Figure 25 – The effects of Rhein, SC514, Ketorolac, Pimecrolimus, Resveratrol and Ascomycin on the viability of SW982 cells +/- TNF- alpha. 42

Figure 26 – The effects of Triamcinolone Acetonide, Meloxicam, SC514, and Diacerin on the viability of SW982 cells +/- TNF alpha. 43

Figure 27 – The effects of Traimcinolone Hexacetonide, Clonidine, Curcumin-14, Fluocinolone, NFkB Activation Inhibitor, Curcumin and Tacrolimus on the viability of SW982 +/- TNF-alpha. 44

Figure 28. Drug toxicity testing with chondrogenic pellets for agents tested in vivo. 45

Figure 29. Drug toxicity testing with chondrogenic pellets for agents not tested in vivo. 47

Figure 30 – Effect of vehicle on weight bearing on Days 7, 14, 21, and 28 55

Figure 31 – Effect of morphine (s.c.) weight bearing on Day 7 56

Figure 32 - Effect of morphine (s.c.) on weight bearing on Day 14

56
Figure 33 - Effect of morphine (s.c.) on weight bearing on Day 21 57
Figure 34 - Effect of morphine (s.c.) on weight bearing on Day 28 57
Figure 35 - Effect of clonidine (s.c.) on weight bearing on Day 7 58
Figure 36 - Effect of clonidine (s.c.) on weight bearing on Day 14 58
Figure 37- Effect of clonidine (s.c.) on weight bearing on Day 21 59
Figure 38 - Effect of clonidine (s.c.) on weight bearing on Day 28 59
Figure 39–Pre-dose effect of daily fluocinolone administration (s.c.) on weight bearing 60
Figure 40 - Effect of fluocinolone (s.c.) on weight bearing on Day 21 60
Figure 41 – Pre-dose effect of weekly clonidine (s.c.) administration on MIA-induced mechanical hyperalgesia 61
Figure 42 - Effect of clonidine (s.c.) on MIA-induced mechanical hyperalgesia on Day 7 62
Figure 43 - Effect of clonidine (s.c.) on MIA-induced mechanical hyperalgesia on Day 14 62
Figure 44- Effect of clonidine (s.c.) on MIA-induced mechanical hyperalgesia on Day 21 63
Figure 45 - Effect of clonidine (s.c.) on MIA-induced mechanical hyperalgesia on Day 28 63
Figure 46 - Effect of daily Tacrolimus administration on pre-dosing MIA-induced mechanical hyperalgesia 64
Figure 47 - Effect of Tacrolimus on MIA-induced mechanical hyperalgesia on Day 21 64
Figure 48– Effect of Curcumin on MIA-induced mechanical hyperalgesia on Day 7 65
Figure 49 - Effect of Fluocinolone on MIA-induced mechanical hyperalgesia on Day 14 65
Figure 50 - Effect of weekly intra-articular Test Compound administration on MIA-induced mechanical hyperalgesia in the rat measured prior to dosing compared to day 7 pretreatment baseline values 68
Figure 51 - Effect of the intra-articular Test Compounds on MIA-induced mechanical hyperalgesia on Day 7 68
Figure 52 - Effect of the intra-articular Test Compounds on MIA-induced mechanical hyperalgesia on Day 14 68
Figure 53 - Effect of the intra-articular Test Compounds on MIA-induced mechanical hyperalgesia on Day 21 69
Figure 54 - Effect of the intra-articular Test Compounds on MIA-induced mechanical hyperalgesia on Day 28 69
Figure 55- Representative images from Trial 3 MIA Model 90
Figure 56 - Day 7, 14, and 21 incapacitance results displayed as a ratio of injured to non-injured and compared with baseline control (shown as a line). 101
Figure 57 - Representative images from Trial 1 MMT/MCLT. **Error! Bookmark not defined.**
Figure 58 - Representative cross-sectional images from vehicle-treated MCLT/MMT animals (Trial 2).**Error! Bookmark not defined.**
Figure 59- Cross section of the joint space for Triamcinolone Hexacetonide treated rats (Trial 2). 111

I. Supplementary Methods

In Vivo Functional Testing

All studies, although non-GLP, were conducted according to Good Laboratory Practices defined in 21 CFR Part 58 with prior Institutional Animal Use Committee approval.

See Appendix for individual animal data. Experimental design is shown in the Appendix of this Supplement. All investigators and study personnel were blind to the groups and treatments through statistical analysis for each study except Trial 1 of the MIA model. In this study, only persons conducting weight bearing and the Pathologist scoring the histopathology slides were blinded to the treatments and groups.

<u>Monoiodoacetoacetate (MIA) Model</u>

All studies using the MIA model were conducted at ALGOS Therapeutics. Figure 1 depicts the typical timeline for the MIA trials.

Figure 1. MIA Trial Timeline

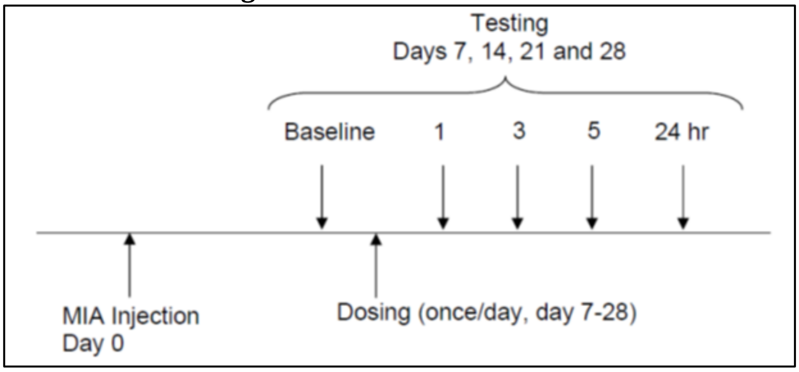

MIA Trial 1

Male Wistar rats (Harlan, Indianapolis, Indiana, U.S.A.) weighing 207 ± 2 grams on the first day of dosing were housed three per cage. Animals had free access to food and water and were maintained on a 12:12h light/dark schedule for the duration of the study. The animal colony was maintained at approximately 21ºC. All experiments were conducted in accordance with the International Association for the Study of Pain Guidelines.

MIA was administered into the left knee as previously described at day 0. Drugs and other compounds were solubilized and stored as recommended by the suppliers. All therapeutic agents and vehicle tested were administered via subcutaneous injection in a 5 ml/kg dose volume once a day. On each test day, WB was measured prior to dosing and 1,3,5, and 24 hours after dosing. Body weight was measured pre-dose.

As previously described, limb WB was measured using a Linton Incapacitance Tester (Stoelting Co., Wood Dale, IL). For each time point tested, rats were allowed to acclimate to the test chamber. Three readings were taken, with each reading measuring the average force exerted over a three second interval. The percent WB for the injured leg was calculated using the following formula:

$$\% \text{ WB Score} = 100 \text{ x } \left[\frac{\text{WB on left leg}}{\text{WB on left leg + WB on right leg}} \right]$$

Treatment groups were assigned based on pre-treatment WB score on Day 7. For each time point tested, the mean of the three WB tests and standard error of the mean (SEM) were determined for each treatment group.

Statistical analyses were conducted using Prism 5.01 (GraphPad, San Diego, CA, USA). The stability of the vehicle group over time was assessed on each testing day (Days 7, 14, 21, and 28) using repeated measures one-way analysis of variance (ANOVA) comparing WB scores (WB) at each experimental time point on a given testing day versus that testing day's pre-treatment measurement. Osteoarthritis-related pain in the vehicle group was estimated by comparing pre-treatment WB on each testing day versus pre-MIA measurements using repeated measures one-way ANOVA. The acute effects of morphine, clonidine, and fluocinolone were analyzed by comparing WB at each experimental time point on a given testing day with the corresponding vehicle measurements using an unpaired t-test. The chronic effects of morphine, clonidine, and fluocinolone treatment were analyzed by comparing pre-treatment WB on each testing day versus the appropriate Day 7 pre-treatment measurements using repeated measures one-way ANOVA and by comparing pre-treatment WB with vehicle pre-treatment WB on each testing day using an unpaired t-test. Pre-MIA and Day 7 pre-treatment WB between all groups was assessed using one-way ANOVA. The Dunnett's multiple comparison post hoc test was used when appropriate.

MIA Trial 2

Male Wistar rats (Harlan, Indianapolis, Indiana, U.S.A.) weighing 178 ± 1 grams (167-207 g) on the first day of dosing were housed three per cage. Animals had free access to food and water and were maintained on a 12:12h light/dark schedule for the duration of the study. Starting on Day 8 after MIA injection, animals receiving Compound C were fed diets consisting of Harlan Teklad 2018, water, sweetened condensed milk and approximately 30 g per rat Bio-Serve cherry flavor nutra-gel (cat# S4798-cup, lot# 109533) in addition to Bio-Serve liquid rat diet diluted 1:4 with water and approximately 40 ml of sweetened condensed milk (cat# F1259SP, lot# 109751). The animal colony was maintained at approximately 21ºC. All experiments were conducted in accordance with the International Association for the Study of Pain Guidelines and were approved by the Algos Therapeutics, Inc. animal care and use committee.

MIA was administered into the left knee as previously described, and all dosing and testing occurred according to the timeline in Figure 1.

Clonidine HCL (CTM-723, 2 mg/ml) was administered weekly, while all other test agents were administered daily. All drugs except curcumin were diluted from stock solutions on the day of administration. Curcumin was weighed each day of dosing and suspended in 0.3% methyl cellulose prior to gavage. Clonidine, Fluocinolone and vehicle were administered via subcutaneous injection in a 5 ml/kg dose volume. Tacrolimus was administered via intraperitoneal injection in a 1 ml/kg dose volume. Curcumin was administered via oral gavage in a dose volume of 5 ml/kg.

Baseline and post-treatment values for mechanical sensitivity were evaluated using a digital Randall-Selitto (dRS) test device (IITC Life Sciences, Woodland Hills, CA). Animals were allowed to acclimate to the testing room for a minimum of 30 minutes before testing. Animals were placed in a restraint sling that suspended the animal, leaving the hind limbs available for testing. Joint compression threshold was measured once at each time point for the ipsilateral and contralateral knee joints. Pressure was applied gradually over approximately 10 seconds to the medial and lateral aspects of the knee joint. Measurements were taken from the first observed behavior of vocalization,

struggle or withdrawal. A cut-off value of 600 g was used to prevent injury to the animal. The mean and standard error of the mean (SEM) were determined for each treatment group.

As with Trial 1, statistical analyses were conducted using Prism™ 5.01. Statistical significance was assigned as $p \leq 0.05$. The stability of the vehicle group over time was assessed on each testing day (Days 7, 14, 21, and 28) using repeated measures one-way analysis of variance (ANOVA) comparing joint compression thresholds at each experimental time point on a given testing day versus that testing day's pre-treatment value. Osteoarthritis-related pain in the vehicle group was estimated at each time point on each testing day by comparing ipsilateral (injured) to contralateral (normal) joint compression thresholds using paired t-tests. The progression of osteoarthritis-related pain in the vehicle group was estimated by comparing pre-treatment joint compression thresholds on each testing day versus pre-MIA measurements using repeated measures one-way ANOVA.

The acute effects of clonidine and Fluocinolone were analyzed by comparing joint compression thresholds at each experimental time point on a given testing day with vehicle treated animals using an unpaired t-test. The acute effect of Tacrolimus was analyzed by comparing joint compression thresholds at each time point on a given testing day with the corresponding pre-treatment baseline values using repeated measures one-way ANOVA and compared to vehicle treated animals using one-way ANOVA. The acute effect of Curcumin was analyzed by comparing joint compression thresholds at each time point on a given testing day with the corresponding pre-treatment baseline values using repeated measures one-way ANOVA. Chronic effects were analyzed by comparing pre-treatment joint compression thresholds on each testing day versus the appropriate Day 7 pre-treatment values using repeated measures one-way ANOVA and by comparing pre-treatment joint compression thresholds with vehicle treated animals on each testing day using an unpaired t-test or one-way ANOVA. Day 7 pre-treatment joint compression thresholds between all groups were assessed using one-way ANOVA. The Dunnett's multiple comparison post hoc test was used when appropriate.

MIA Trial 3

Male Wistar rats (Harlan, Indianapolis, Indiana, U.S.A.) weighing 207 ± 2 grams on the first day of dosing were housed three per cage. Animals had free access to food and water and were maintained on a 12:12h light/dark schedule for the duration of the study. The animal colony was maintained at approximately 21ºC. All experiments were conducted in accordance with the International Association for the Study of Pain Guidelines.

Vehicle and compounds were administered locally into the injured knee joint (intra-articular; i.a.) on Days 7, 14, 21, and 28. Clonidine (0.1 mg/kg) served as the positive control and was administered subcutaneously on Days 7, 14, 21, and 28. All injections were done with a 27 gauge needle. As described for Trial 2, mechanical hyperalgesia was measured by determining the force, applied to either the ipsilateral (injured) or contralateral knee, required to elicit a nocifensive response to joint compression using a digital Randall-Selitto test device. Joint compression thresholds were measured 7, 14, 21 and 28 days after MIA injection. On all testing days after MIA injection, joint compression thresholds were measured prior to dosing and 1, 3, 5, and 24 hours after dosing. Individual animal data is shown in the Appendix.

<u>Meniscal Tear (MMT/MCLT) Model</u>

All MMT/MCLT trials and associated analyses were conducted at BolderBioPath. The general timecourse for each study is depicted in Figure 2. Animals (8 or 10/group), were housed 2-3/cage and kept on a 12 hour light/dark schedule. To initiate the model, animals were anesthetized with

Isoflurane and the right knee area prepared for surgery. A skin incision was made over the medial aspect of the knee and the medial collateral ligament was exposed by blunt dissection and then transected. The medial meniscus was cut through the full thickness to simulate a complete tear and the skin was then closed with suture.

All pharmaceutical agents were prepared and stored as recommended by the supplier. Daily drug preparations were made fresh from stock solutions. In Trial 1, test articles were administered subcutaneously daily for three weeks beginning one day prior to surgery. In Trial 2 drugs were administered IA weekly beginning one week following surgery. Both Curcumin and Withaferin were delivered by gavage and were prepared in 0.3% Methyl Cellulose.

WB was measured five times a day, once a week, using a Linton incapacitance meter (Stoelting Co.) to assess pain behavior. Rats were acclimated to the incapacitance meter prior to study initiation. Similarly, immediately prior to WB animals were placed in the incapacitance meter and allowed to acclimate for 2-5 minutes. Each hind paw was placed on a separate force plate so that the force exerted by each side could be averaged over a 5 second interval and the mean of three readings taken for each data point. Right paw force was compared to left for each group to confirm that animals were displaying incapacitance / pain behavior. Difference in force (left minus right) and right paw force as a percentage of the total force exerted by both paws were determined and compared between groups. Three weeks after surgery, animals were humanely euthanized and right knees collected for histopathology evaluation.

Figure 2. MMT/MCLT Trial Timeline

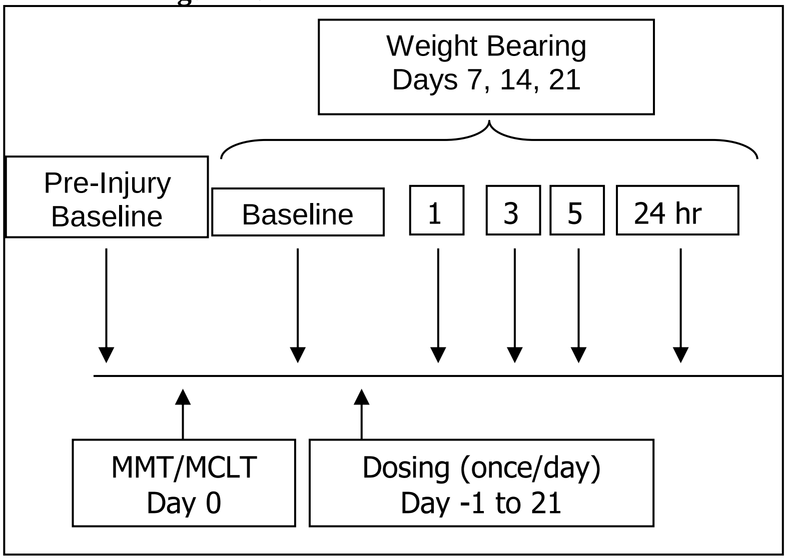

MMT/MCLT Trial 1

The study design and animal usage was approved by Bolder BioPATH's Institutional Animal Care and Use Committee (IACUC) for compliance with regulations prior to study initiation (IACUC Protocol #BBP03-006).

Male Lewis rats weighing 261-291 grams (mean 277) on day -1 were obtained from Charles River Labs. Animals were identified by a distinct mark at the base of the tail delineating group and animal number. After randomization, all cages were labeled with protocol number, group numbers, and animal numbers with appropriate color-coding (see protocol).

Upon arrival, animals were housed 4/cage in shoe-box polycarbonate cages with wire tops, wood chip bedding and suspended food and water bottles. The cages conformed to the guidelines cited in the Guide for the Care and Use of Laboratory Animals (4). Animals were acclimated for 7 days prior to being randomized into groups. An attending veterinarian was on site or on call during the live phase of the study, and no concurrent medications were given. During the acclimation and study periods, animals were housed in a laboratory environment with temperatures ranging between 67-76°F and relative humidity between 30-70%. Automatic timers provided 12 hours of light and 12 hours of dark. Animals were allowed access ad libitum to Harlan Teklad Rodent Chow and fresh municipal tap water.

Dosing by the intraperitoneal route (qd at 24 hr intervals) was initiated on day -1 before surgery and was continued for 3 weeks except group 2 (morphine) was dosed on days 7, 14, 21 only for purposes of pain testing. Both Withaferin and Curcumin were delivered by gavage qd at 24 hr intervals. WB was measured pretreatment, and at 1,3, 5 and 24 hours post dosing.

MMT/MCLT Trial 2

This basic study design and animal usage was approved by Bolder BioPATH's Institutional Animal Care and Use Committee (IACUC) for compliance with regulations prior to study initiation (IACUC Protocol #BBP03-006).

Male Lewis rats weighing 260-295 grams (mean 283) on day -5 were obtained from Charles River Labs. Animals were identified by a distinct mark at the base of the tail delineating group and animal number. After randomization, all cages were labeled with protocol number, group numbers, and animal numbers with appropriate color-coding (see protocol).

Upon arrival, animals were housed 4/cage in shoe-box polycarbonate cages with wire tops, wood chip bedding and suspended food and water bottles. The cages conformed to the guidelines cited in the Guide for the Care and Use of Laboratory Animals. Animals were acclimated for 7 days prior to being randomized into groups. An attending veterinarian was on site or on call during the live phase of the study, and no concurrent medications were given. During the acclimation and study periods, animals were housed in a laboratory environment with temperatures ranging between 67-76°F and relative humidity between 30-70%. Automatic timers provided 12 hours of light and 12 hours of dark. Animals were allowed access ad libitum to Harlan Teklad Rodent Chow and fresh municipal tap water.

Dosing by the intra-articular route was initiated on day 7 after surgery and was continued for 3 weeks (days 7, 14, 21) in all groups except group 2 (clonidine) which was dosed subcutaneously (systemically) on days 7, 14, 21. WB was measured pretreatment, and at 1,3, and 5 hours post dosing.

Histopathology

Dissected joints were fixed in 10% formaldehyde and decalcified for two days in 10% formic acid. Knees were trimmed into approximately two equal frontal halves, processed through graded alcohol to dehydrate, cleared and paraffin embedded by conventional methods. Blocks were sectioned and stained with either or both hematoxylin and eosin or toluidine blue.

<u>Histopathology MIA Model</u>

The histopathology for the first MIA trial was performed by Premier Laboratories (Dr. Michael Hawes, APS, Pathologist). Treatments were masked during the assessment. Chondrocyte necrosis and proteolytic degeneration of the femurs were scored on a 5 point scale with 0 = None; 1 = <10%; 2 = 10-30%; 3 = 30-60%; 4 = 60-90%; 5 =>90%.. Other parameters analyzed included proteoglycan content (safranin O staining), synovial membrane inflammation and proliferation, subchondral bone, and articular cartilage (as indicated in Supplementary methods). Characteristics within these categories were scored as normal, minimal, mild, moderate, marked, or +/- when severity was not graded. Slides from Trial 2 were further reviewed by Dr. Bendele using the analytical method described below for the MMT/MCLT model to confirm any conclusions.

For the histopathology of the MIA model in Trial 3, completed by Bolder BioPATH (and Dr. Bendele), a tolouidine blue stained slide containing both halves of the joint was comprehensively analyzed from test and control subjects. Approximate % of total loss of articular chondrocytes, loss of proteoglycan and loss of collagenous matrix were determined for each articular surface (medial tibial plateau (MTP), lateral tibial plateau (LTP), medial femoral condyle (MFC), and lateral femoral condyle (LFC). Chondrocyte loss was quantified by estimating the area of cartilage in which there were no apparently viable chondrocytes and proteoglycan loss was quantified by estimating differences in intensity of toluidine blue matrix staining. Loss of interstitial matrix was an indication of disruption and diminished type II collagen. Based on the mean % loss for the four surfaces in these parameters, a five point matrix score was utilized with the subchondral bone resorption, subchondral bone sclerosis and osteophyte scores to achieve a total joint score.

<u>Histopathology MMT/MCLT Model</u>

Degenerative changes occur by 3-6 weeks post-surgery in the MMT/MCLT model and are of greatest severity on the outer 1/3 of the tibial cartilage. Less severe degenerative changes are observed in the middle and inner 1/3 of the tibial cartilage. Osteophytes progressively increase in size and are ultimately quite large (medial tibia). By 12 months nearly all rats show total cartilage loss. Due to the rapid progression of cartilage degeneration, protective effects of therapeutics are not always apparent in the outer 1/3 of the tibial cartilage, although zonal analysis may reveal effects of treatment in the middle and inner 1/3. Substantial subchondral and epiphyseal bone changes occur in the medial tibia subjacent to the areas of greatest lesion severity. This model offered an opportunity to evaluate both the chondroprotective effects and bone preserving activities of the various agents tested.

The histopathological analysis of the MMT/MCLT model was also performed by Bolder BioPATH and Dr. Bendele. To accomplish this, three sections were cut from each paraffin embedded knee at 200 uM steps, stained with toluidine blue (with a right and left halve per section), and analyzed for cartilage degeneration, proteoglycan loss, collagen damage, and osteophyte formation. Results were averaged across the 3 sections for an overall semi-quantitative score. Regional differences across the tibial plateau were taken into consideration by dividing each section into 3 zones delineated using an ocular micrometer (outside, middle, and inside). Scores were based on the percentage of area affected within the zone.

Cartilage degeneration in the tibia was scored on a five point scale for each zone, collagen damage across the medial tibial plateau was measured by total width for each grade damage. Cartilage degeneration in the tibia was scored none to severe (numerical values 0-5) for each zone (area defined by micrometer) using the following criteria:

0=no degeneration

1=minimal degeneration, chondrocyte and proteoglycan loss, generally without fibrillation involving the mainly the superficial zone or extending into the upper 10% of the cartilage thickness (50% or greater width of the zone), or at least 5% but not more than 10% overall total proteoglycan and cell loss in the zone if lesion is focal and deeper in some areas

2=mild degeneration, chondrocyte and proteoglycan loss with fibrillation involving mainly the upper 25% of cartilage thickness (50% or greater width of the zone), fibrillation generally superficial (upper 10%) while chondrocyte and PG loss extend into approximately 25% of the cartilage depth, or 10-25% overall chondrocyte and proteoglycan loss in the zone if lesion is focal and deeper in some areas

3=moderate degeneration, chondrocyte and proteoglycan loss with fibrillation extending well into the midzone and generally affecting " (50%) of the total cartilage thickness (50% or greater width of the zone), fibrillation/collagen damage generally extends into upper 25% while chondrocyte and PG loss extend through 50% thickness, or 25-50% overall chondrocyte and proteoglycan loss in the zone if lesion is focal and deeper in some areas

4=marked degeneration, chondrocyte and proteoglycan loss with fibrillation extending through the mid zone into the lower 1/3 (75% of cartilage thickness) but without complete (to the tidemark) loss of chondrocytes or proteoglycan (50% or greater width of the zone), fibrillation/collagen damage generally extend through the mid zone (50% thickness) but deep zone collagen remains intact and chondrocyte and PG loss extend through 75% of cartilage thickness, or 50-75% overall chondrocyte and proteoglycan loss in the zone if lesion is focal and deeper in some areas

5=severe degeneration, matrix loss to tidemark (50% or greater width of the zone), or 75-
100% overall chondrocyte and proteoglycan loss in the zone A 3-zone sum for cartilage degeneration was also calculated. Femoral general cartilage degeneration was scored using the same criteria without attention to zones.

In addition to overall cartilage degeneration, collagen damage across the medial tibial plateau (the most severely affected section of the 2 halves) was quantified by measuring the total width of the following:

- Any damage (fibrillation ranging from superficial to full thickness loss).
- Severe damage (total or near total loss of collagen to tidemark, >90% thickness)
- Marked damage (extends through 61-90% of the cartilage thickness)
- Moderate damage (extends thru 31-60% of the cartilage thickness)
- Mild damage (extends through 11-30% of the cartilage thickness)
- Minimal damage (very superficial, affecting upper 10% only)

Two cartilage degeneration width measurements were also taken:

(1) The <u>total tibial cartilage degeneration width (um)</u> of the total extent of tibial plateau affected by any type of degeneration (cell loss, proteoglycan loss or collagen damage). This measurement extended from the origination of the osteophyte with adjacent cartilage degeneration (outside 1/3) across the surface to the point where tangential layer and underlying cartilage appeared histologically normal.

(2) The <u>significant cartilage degeneration width</u> (um) reflects areas of tibial cartilage degeneration in which both chondrocyte and proteoglycan loss extend through greater than 50% of the cartilage thickness. In general, the collagen damage is mild (25% depth) or greater for this

parameter but chondrocyte and proteoglycan loss extend to at least 50% or greater of the cartilage depth.

A micrometer depth of any type of lesion (both chondrocyte and proteoglycan loss, but may have good retention of collagenous matrix and no fibrillation), expressed as a ratio of depth of changed area vs. depth to tidemark, was taken in the area of greatest lesion severity in each of the 3 zones across the tibial surface at the midpoint of the zone. This measurement is the most critical analysis of any type of microscopic change present. The denominator can serve as an average measure of cartilage thickness in each of the 3 zones for comparison of anabolics when measures are taken at the midpoint of the zone. In this study, the cartilage thickness of zone 3 was evaluated in this manner.

Osteophytes and femoral cartilage degeneration were also scored. The femoral degeneration and the 3 zone tibial degeneration scores were summed to create the total cartilage degeneration score. The mean osteophyte score for each joint was added to this value to create a total joint score.

Scoring of the osteophytes and categorization into small, medium and large was done with an ocular micrometer. Marginal zone proliferative changes have to be ≥200 µm in order to be measured and designated as osteophytes. Scores are assigned to the largest osteophyte in each section (typically found in the tibia) according to the following criteria:

1=small up to 299 µm
2=moderate 300-399 µm
3=large 400-499 µm
4=very large 500-599
5=very large 600

The actual osteophyte measurement (tidemark to furthest distance point extending toward synovium) was also recorded. The femoral cartilage degeneration score and the 3-zone sum of the tibial cartilage degeneration scores (mean of 3 levels) were summed to create a total cartilage degeneration score. The mean osteophyte score for each joint was added to this value to create a **total joint score**.

Overall findings including synovial health were assessed and documented (e.g., Table 1), and representative images were saved for each animal (note that not all documentation is shown here). The most complete histopathological analyses were completed for the last MIA Trial # 3 and the MMT/MCLT Trials.

Table 1 and 2 below are examples of the method used to quantify histopathological changes for each animal, including individual animal joint histopathology and scoring of cartilage degeneration.

Table 1 – Example of a histopathology report for an individual animal.

Individual Animal Histopathology Report (ROA/MED-2)

Study Information

B

Species: Rat
Group: 4
Necropsy Day: 25

Sex: Male
Animal Number: 4
Disposition: Killed Terminal

Pathology

	Medial Tibia						Mean	Width (µm)	Medial Femur	Score	Osteophyte		Score
	Outside		Middle		Inside		by level	total/sub					
Level 1	5		2		0		7	1000	Minimal 1/3	1	Medium	300 µm	2
Relative Depth	150	150	20	180	0	280		800					
Relative Depth Ratio	1.00		0.11		0.00		0.37						
Level 2	5		3		0		8	1100	Mild 1/3	2	Medium	340 µm	2
Relative Depth	150	150	10	180	0	330		900					
Relative Depth Ratio	1.00		0.06		0.00		0.35						
Level 3	5		3		0		8	2000	Mild 1/3	2	Medium	300 µm	2
Relative Depth	150	150	60	230	10	380		900					
Relative Depth Ratio	1.00		0.26		0.03		0.43						
Means by Zone													
Score	5.00		2.67		0.00		7.666667	1366.67					
Relative Depth	150	150	30	197	3	330		866.67					
Relative Depth Ratio	1.00		0.14		0.01		0.38						

Comments	
Synovium	Hypocellular Fibrous Repair
Observations	Meniscal tear+marked bone marrow hypocellularity

Summary	
Bone Score	3
Mean Osteophyte Score	2.0
Mean Osteophyte Measurement	313
Mean CD Score Tibia	7.7
Mean CD Score Femur	1.7
Total CD Score	9.3
Total Joint Score	11.3

Table 2 – Example of calculated histopathological degeneration for an individual animal

Group 4, Animal 1	Normal	Total	Severe	Marked	Moderate	Mild	Minimal
L1	800	1200	500	0	200	200	300
L2	900	1100	700	0	200	100	100
L3	900	1100	800	100	100	100	0
Mean	866.67	1133.33	666.67	33.33	166.67	133.33	133.33

Group 4, Animal 2	Normal	Total	Severe	Marked	Moderate	Mild	Minimal
L1	900	1100	600	500	0	0	0
L2	700	1300	800	300	0	0	200
L3	900	1100	1000	0	100	0	0
Mean	833.33	1166.67	800.00	266.67	33.33	0.00	66.67

Group 4, Animal 3	Normal	Total	Severe	Marked	Moderate	Mild	Minimal
L1	1000	1000	300	100	0	0	600
L2	1000	1000	500	0	100	0	400
L3	1000	1000	600	100	100	0	200
Mean	1000.00	1000.00	466.67	66.67	66.67	0.00	400.00

Group 4, Animal 4	Normal	Total	Severe	Marked	Moderate	Mild	Minimal
L1	1000	1000	500	100	0	200	200
L2	1100	900	600	200	0	100	0
L3	800	1200	700	100	0	100	300
Mean	966.67	1033.33	600.00	133.33	0.00	133.33	166.67

Group 4, Animal 5	Normal	Total	Severe	Marked	Moderate	Mild	Minimal
L1	1000	1000	500	0	300	0	200
L2	900	1100	600	100	100	0	300
L3	1000	1000	800	0	0	100	100
Mean	966.67	1033.33	633.33	33.33	133.33	33.33	200.00

Micro CT

Knees were fixed in formalin and scanned using a Scanco µCT40 (Scanco MedicalAG, Brutisellen, Switzerland) set at 55kV x-ray tube energy, 145uA intensity with a 300ms image acquisition time. Slice thickness was 16µM, with isotropic voxels, and 511 projections were taken for each 360 degree rotation. To correct for beam hardening artifacts, a mu-scaling algorithm was applied at a level of 200 mg hydroxyapatite/cm3.

All µCT analysis was performed with the Scanco Medical Analysis software. Because the µCT scanner acquires images in a tranverse plane (along the z axis of the scan), and the region of interest

(ROI) for analysis is most easily identified in sagittal sections (along the y axis), scans were reformatted from transverse to sagittal planes. The tibial plateau was identified, and the slice numbers corresponding to the medial side were recorded. One hundred slices from the middle of the region were selected for analysis, and a contour applied to the subchondral bone that was 50 pixels (800μM) in diameter. The final size of the ROI was 800μM x 1600μM, pre-Gauss filtering. Care was taken to exclude cortical bone and only include trabecular bone in the ROI. A morphometric analysis on the ROI of each sample was completed, with the same size Gauss filter and threshold applied throughout the entire study. The output of this analysis included total volume analyzed, bone volume, trabecular bone measurements and a structural model index.

Parameters analyzed included:

1) Relative bone volume in the ROI (BoneVolume /Total Volume)
2) Trabecular spacing, calculated by measuring 3D distances directly in the trabecular network. This was the mean diameter of spheres filling the marrow space.
3) Trabecular thickness, calculated by measuring 3D distances directly in the trabecular network. This was the mean diameter of spheres filling the trabecular structure
4) Trabecular number, calculated by measuring 3D distances directly in the trabecular network. This was taken as the inverse of the mean diameter of spheres filling the skeletonized structure.
5) Connectivity density, based on the Conn-Euler number
6) Structural model index, which indicates the relative "rod-like" or "plate-like" structure where 3 is the theoretical value of a perfect cylinder and 0 the value of a perfect plate.

Statistics

General

Statistical analyses were conducted using Prism 5.01 (GraphPad, San Diego, CA, USA) or MS Excel with MedStat. For in vitro testing, one or two-way analysis of variance (ANOVA) was used to look for differences and if significant further testing with Dunnett's or pair-wise t-tests as indicated. In general, results of pairwise comparisons with controls using a standard two tailed T test are shown in Figure graphics where "*" indicates significance of $P \leq 0.05$. Samples were run at least in triplicate and tests repeated. Further details are included as needed in the text and figure legends.

μCT

A one-way ANOVA was used to look for differences between groups and if p was found to be <0.05, then Bonferroni's post-hoc test was performed to identify any significant differences between groups.

MIA and MMT/MCLT

Statistical significance was assigned as $p \leq 0.05$.

For the MIA WB studies, comparisons were made using repeated measures one-way ANOVA comparing WB scores at each experimental time point on a given testing day with that testing day's

pre-treatment measurement. Pain behavior in the vehicle group was estimated by comparing pre-treatment WB scores on each test day vs pre-MIA measurements using repeated measures one-way ANOVA. The acute effects of morphine, clonidine, and fluocinolone were analyzed by comparing WB scores at each test time point with the corresponding vehicle measurements using an unpaired t-test. The chronic effects of morphine, clonidine, and fluocinolone treatment were analyzed by comparing pre-treatment WB scoreson each testing day versus the appropriate Day 7 pre-treatment measurements using repeated measures one-way ANOVA and by comparing pre-treatment WB with vehicle pre-treatment WB on each testing day using an unpaired t-test. Pre-MIA and Day 7 pre-treatment WB between all groups was assessed using one-way ANOVA. The Dunnett's multiple comparison post hoc test was used when appropriate.

For MIA dRS studies, repeated measures one-way ANOVA comparing joint compression thresholds at each experimental time point on a given testing day was compared versus that testing day's pre-treatment value. Osteoarthritis-related pain in the vehicle group was estimated at each time point on each testing day by comparing ipsilateral (injured) to contralateral (normal) joint compression thresholds using paired t-tests. The progression of osteoarthritis-related pain for other treatments was similarly estimated by comparing pre-treatment joint compression thresholds on each testing day versus pre-MIA measurements using repeated measures one-way ANOVA and by comparing pre-treatment joint compression thresholds with vehicle treated animals on each testing day using an unpaired t-test or one-way ANOVA.

Similarly, for the MMT/MCLT WB studies, a repeated measures one-way ANOVA comparing pre and post treatment WB measure with the vehicle treated animal or pain behavior control treatment as indicated.

For histopathological comparisons, non-qualitative scales were used for scoring, and a treatment group Mean ± SEM for each score and measurement was determined as previously recommended by Gerwin et al., 2010 for the OARSI histopathology initiative.[1] Statistical analyses were then performed using parametric ANOVA methods. When several treatment groups were compared, multiple comparison procedures such as Bonferroni or Tukey correction were used. Dunnett's test was applied for comparisons to vehicle. Scored parameters were analyzed using a KruskaleWallis test with Dunn's post-test.

II. Supplementary Results

In Vitro NF-κB

NF-κB activity was assessed in HeLa cells that were transiently transfected with two reporter constructs as described in the manuscript. Agents were tested for their ability to block TNF-stimulated activity; results for those agents also tested in vivo were described in the manuscript. Expression of the luciferase from the Renilla construct was used as a measure of viability, which was confirmed with a cytotoxicity assay (see later section). Some compounds were found to inhibit Renilla expression at high concentrations indicating a toxic effect. For example, sulfasalazine was cytotoxic at 800 μM as measured by the Promega cytotoxicity assay and increasingly inhibited renilla expression from 50 to 800 μM. This may in part represent an effect on the CMV promoter (**Figure 3**).

Shown below are representative results for agents tested in the NF-κB assay (**Figure 4**). **Figure 4** includes results not reported in the manuscript. If inhibition of TNF-activated NF-κB was only detected in the range of toxicity, the agents were not advanced for in vivo testing. NF-κB inhibitor

VI was the most effective of those compounds shown here (**Figure 4 G, H**). This agent was used for control purposes only. Ascomycin was also very effective in the range tested (**Figure 4, B**). Further testing at lower concentrations was not completed by the time in vivo testing commenced. It was unexpected that BMS-345541, Inhibitor IV, and SC514 failed to inhibit NF-κB in this system at the concentrations tested, especially because, similar to Inhibitor IV, they are inhibitors of IKK-2. It is possible that the half-life or stability in the HeLa cell system, which was not measured, could have played a role. Only a very high concentration (680 uM) of Boswellia was found to be effective against NF-κB in this assay (not shown). IKK inhibitor V inhibited at higher concentrations (not shown).

Figure 3. Sulfasalazine effects on NFκB using the dual reporter assay

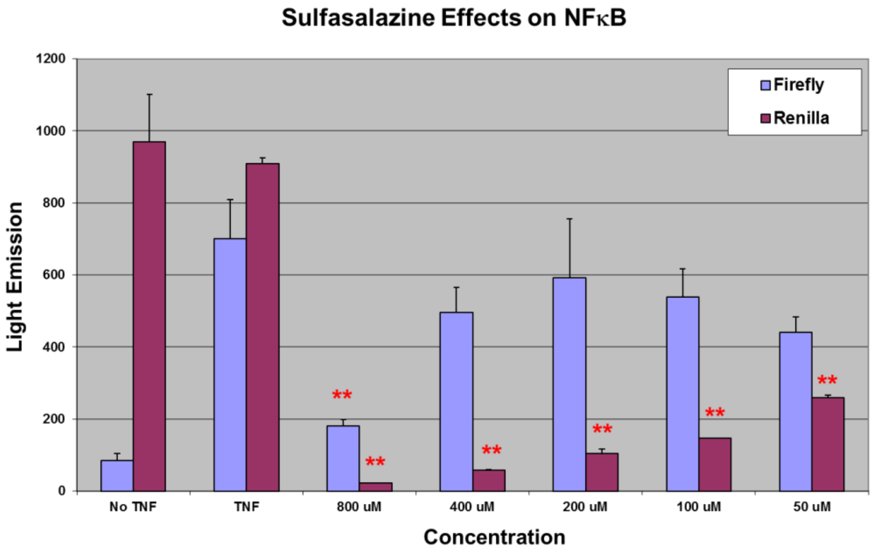

Figure 4. NF-κB activity with and without drug treatment. Controls: 'Media' = untreated HeLa cells; 'Media + TNF' = cells treated with TNF-α; 'Media + Enbrel' = cells treated with TNF-α in the presence of Enbrel, a known TNF inhibitor. Test: Cells treated with TNF-α in the presence of different concentrations of drug. Additional data is shown in the manuscript.

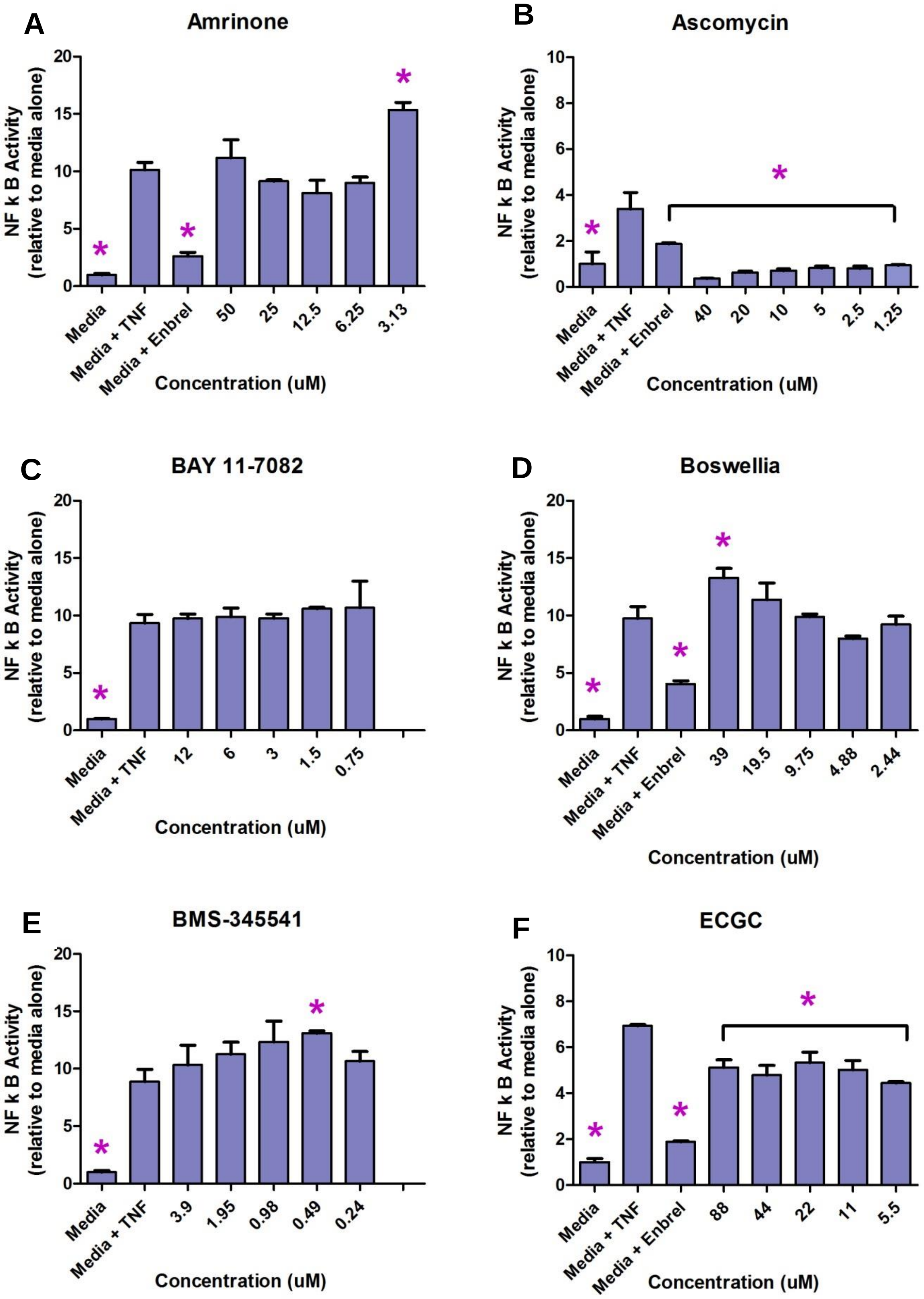

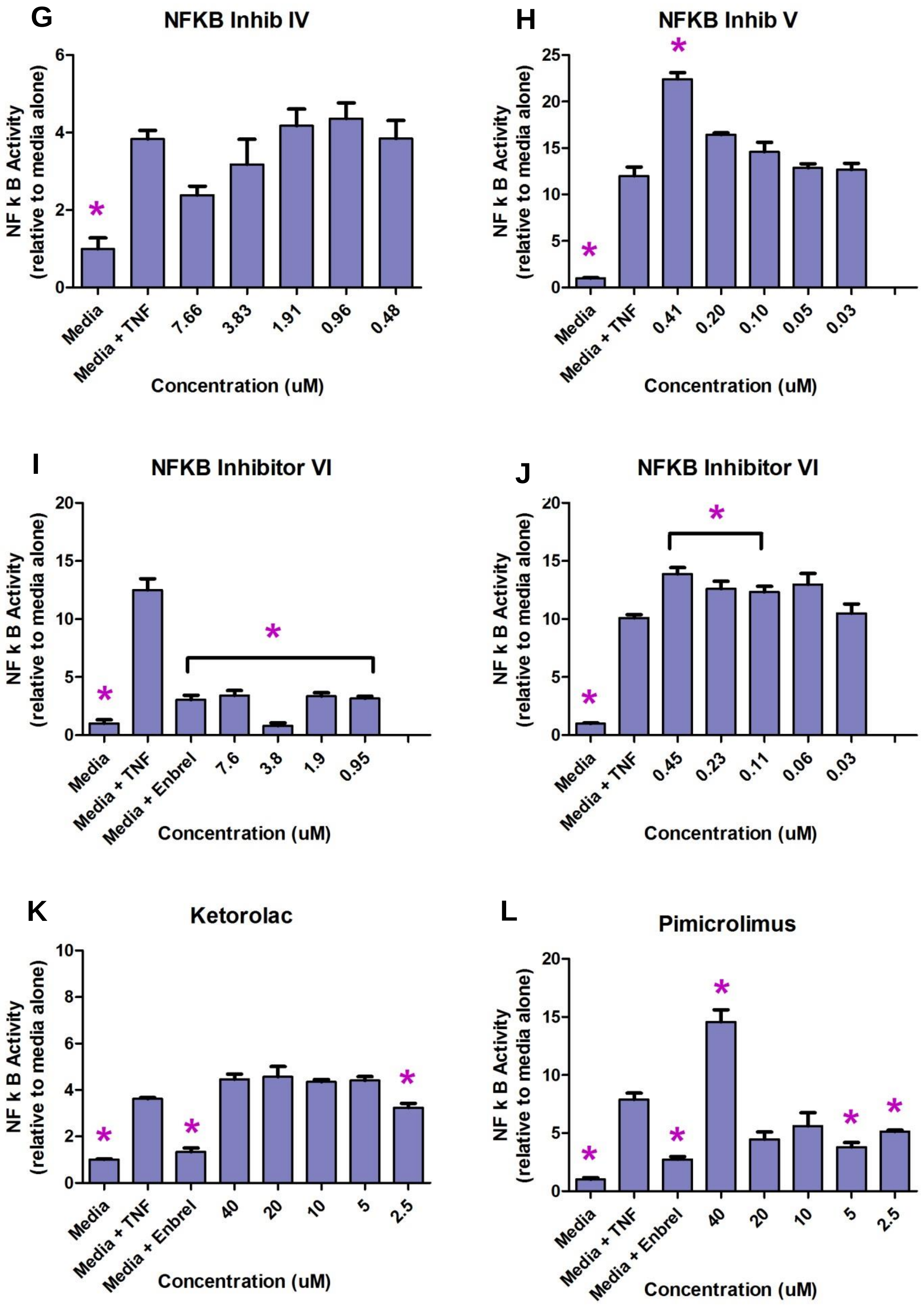
G
NFKB Inhib IV
NF k B Activity (relative to media alone)
0
2
4
6
*
Media
Media + TNF
7.66
3.83
1.91
0.96
0.48
Concentration (uM)
H
NFKB Inhib V
NF k B Activity (relative to media alone)
0
5
10
15
20
25
*
*
Media
Media + TNF
0.41
0.20
0.10
0.05
0.03
Concentration (uM)
I
NFKB Inhibitor VI
NF k B Activity (relative to media alone)
0
5
10
15
20
*
*
Media
Media + TNF
Media + Enbrel
7.6
3.8
1.9
0.95
Concentration (uM)
J
NFKB Inhibitor VI
NF k B Activity (relative to media alone)
0
5
10
15
20
*
*
Media
Media + TNF
0.45
0.23
0.11
0.06
0.03
Concentration (uM)
K
Ketorolac
NF k B Activity (relative to media alone)
0
2
4
6
8
10
*
*
*
Media
Media + TNF
Media + Enbrel
40
20
10
5
2.5
Concentration (uM)
L
Pimicrolimus
NF k B Activity (relative to media alone)
0
5
10
15
20
*
*
*
*
*
Media
Media + TNF
Media + Enbrel
40
20
10
5
2.5
Concentration (uM)

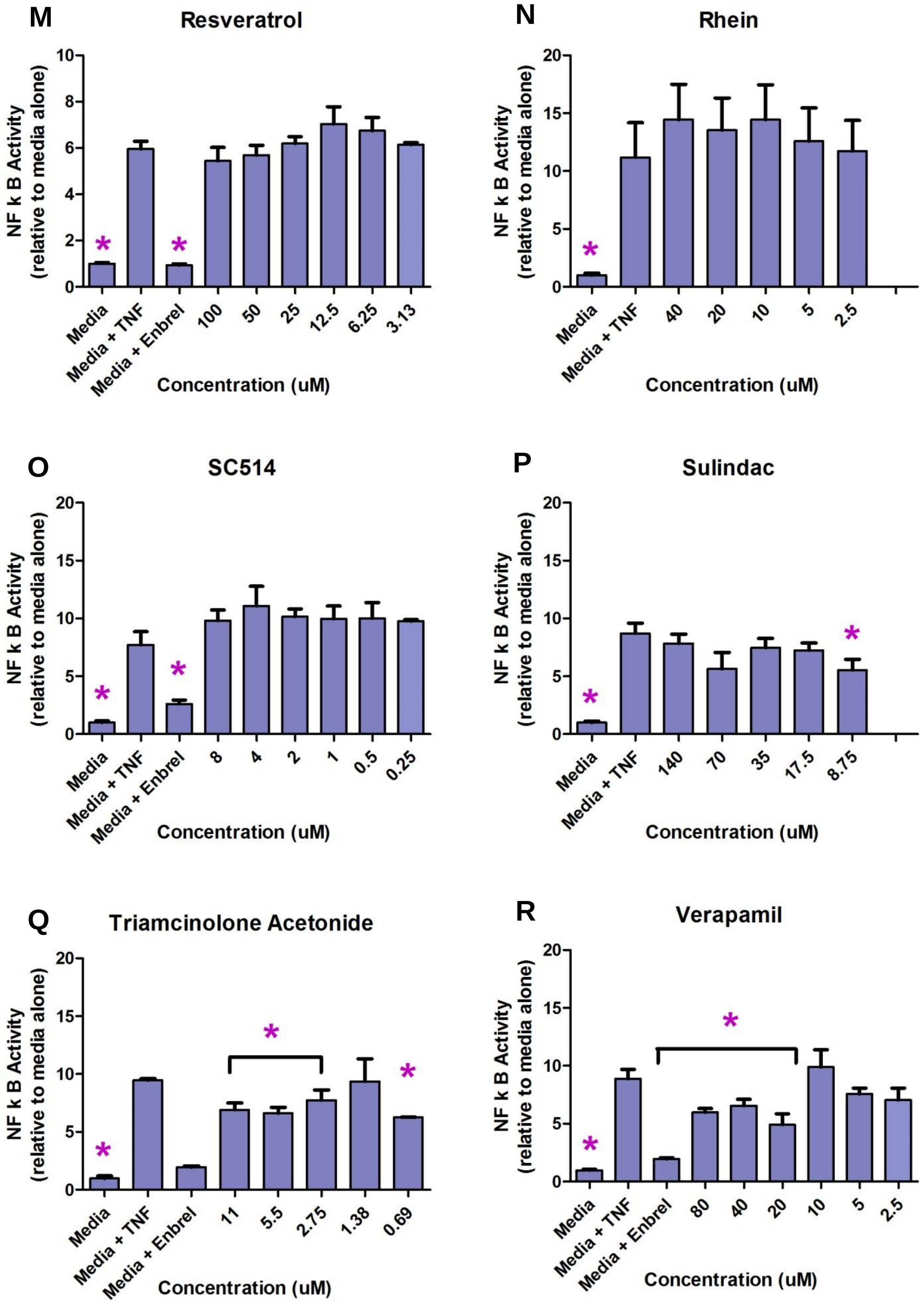
M
Resveratrol
NF k B Activity (relative to media alone)
0
2
4
6
8
10
Media
Media + TNF
Media + Enbrel
100
50
25
12.5
6.25
3.13
Concentration (uM)
N
Rhein
NF k B Activity (relative to media alone)
0
5
10
15
20
Media
Media + TNF
40
20
10
5
2.5
Concentration (uM)
O
SC514
NF k B Activity (relative to media alone)
0
5
10
15
20
Media
Media + TNF
Media + Enbrel
8
4
2
1
0.5
0.25
Concentration (uM)
P
Sulindac
NF k B Activity (relative to media alone)
0
5
10
15
20
Media
Media + TNF
140
70
35
17.5
8.75
Concentration (uM)
Q
Triamcinolone Acetonide
NF k B Activity (relative to media alone)
0
5
10
15
20
Media
Media + TNF
Media + Enbrel
11
5.5
2.75
1.38
0.69
Concentration (uM)
R
Verapamil
NF k B Activity (relative to media alone)
0
5
10
15
20
Media
Media + TNF
Media + Enbrel
80
40
20
10
5
2.5
Concentration (uM)

In Vitro Osteoclastogenesis

Osteoclastogenic activity was measured using BD osteologic plates and quantitation of the degree of bone matrix digestion on the plate surface. Images from the BD Osteologic assay used to measure drug effects on osteoclastogenesis and osteoclast activity are shown in **Figure 5.** The far left pane depicts an example of a control well after removal of cells from matrix. Pseudocoloring of matrix (blue) and pits (green) is shown in the far right pane. A transparent overlay of the two images is shown in the center.

Figure 5 - Measurement of drug effects on osteoclastogenesis and osteoclast activity.

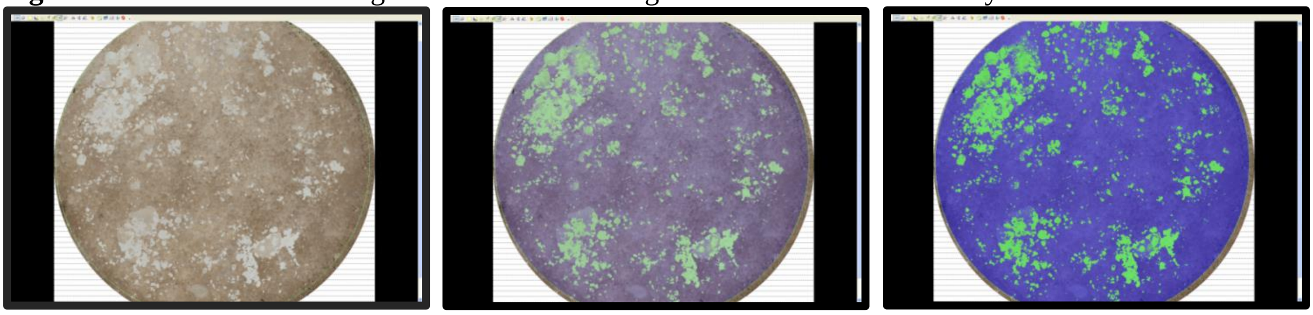

Comparisons were made between RANKL treated control wells and wells treated with both RANKL and drug.

Agents were initially tested either during differentiation or post differentiation in the bone assay (e.g., Figure 2, Sulfazalazine). It was found that this assay was more discerning if the drugs were present from initiation of differentiation with RANKL, likely because matrix degradation began before all cells had completely differentiated. Thus, this assay does not differentiate between effects on differentiation or effects on activity.

A number of agents were effective at inhibiting matrix dissolution. Representative results are shown in **Figures 6 through 9** and were expressed as the mean +/- SEM (n=4) at the concentrations indicated (*/**: $p < 0.05/0.01$, unpaired t-test versus RANKL control). See Figure 2 in the manuscript for additional data. Most effective were Tacrolimus (2.6 nM was the lowest inhibitory concentration), Pimicrolimus and Ascomycin (10 nM), and Fluocinolone (9.77 nM). Others that were effective, but not at such low concentrations, were Triamcinolone hexacetonide (297 nM), Tranilast (500 nM), IKK2 VIII inhibitor (500 nM), Withaferin A (530 nM), Alendronate (1.25 uM), Resveratrol (6.25 uM), clonidine (10 uM), CORM-2 (20 uM), and Triamcinolone acetonide (250 uM). Alendronate was included as a control. Both Triamcinolones were formulated for clinical use, and so it is possible that the different excipients influenced in vitro solubility and the significantly different results. Because Triamcinolone hexacetonide was most effective, this is the formulation used in vivo.

Figure 6. Effect of sulfasalazine on osteoclast differentiation vs. mature activity.

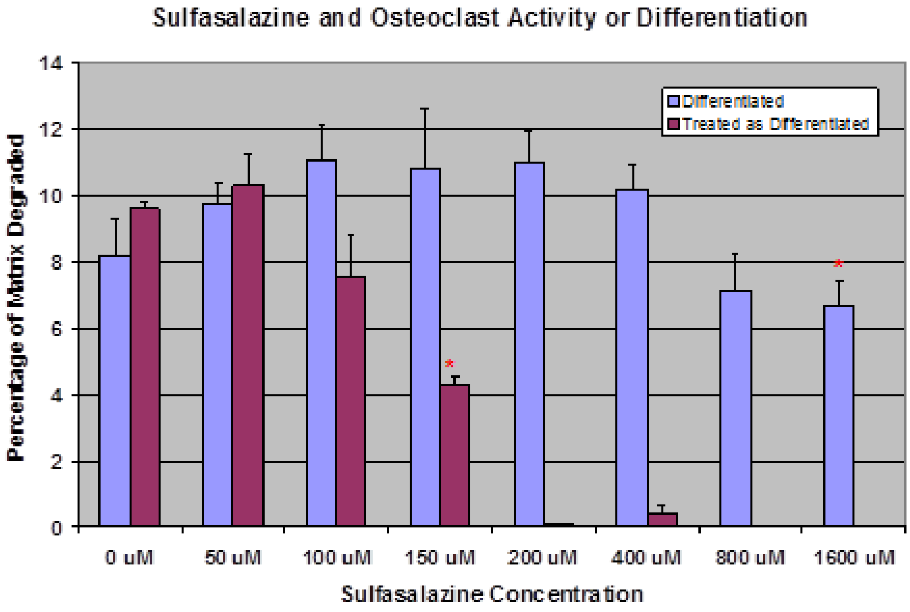

Figure 7 – Effects of different drugs on osteoclastogenesis and degradation of bone matrix.

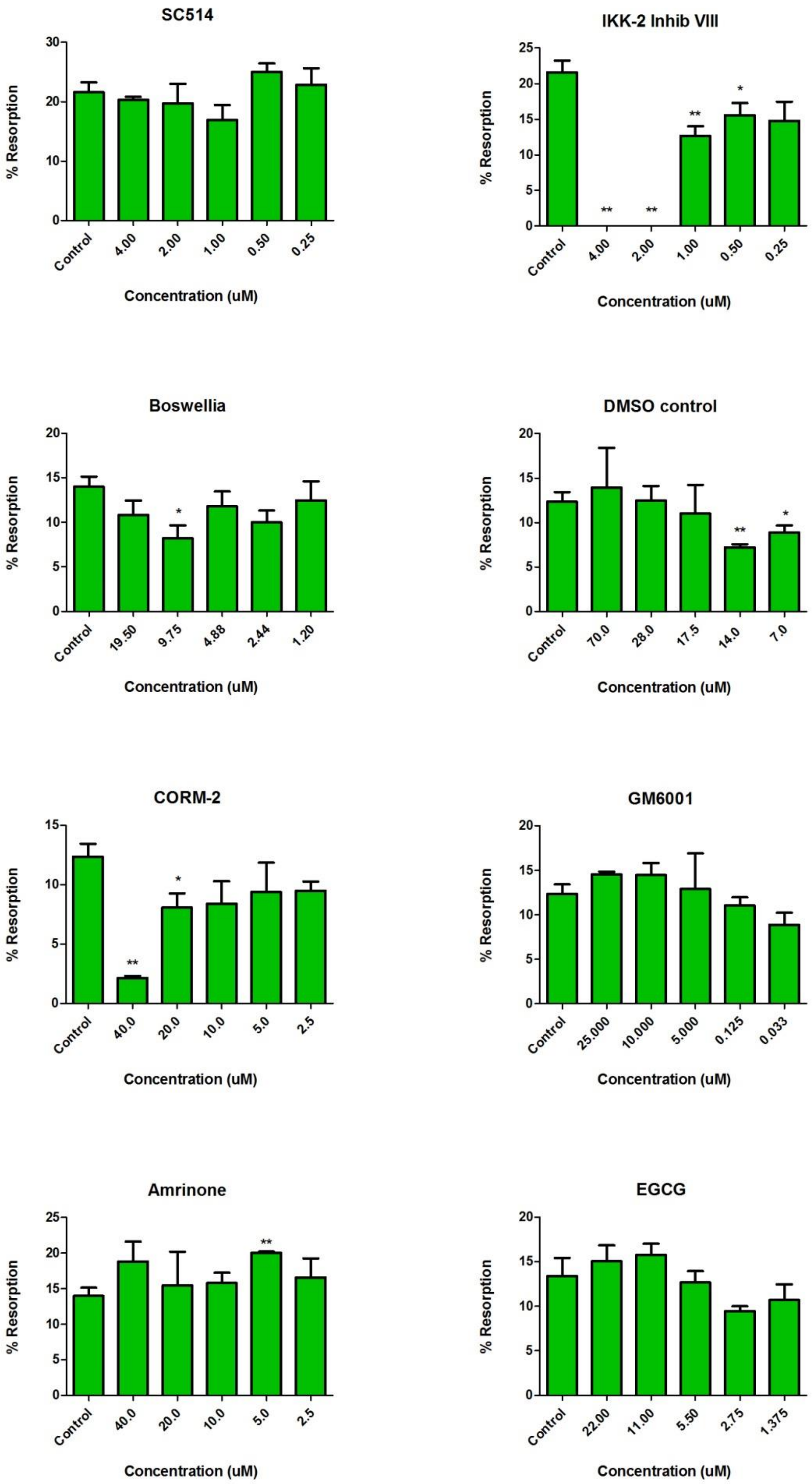

Figure 8 - Effects of different drugs on osteoclastogenesis and degradation of bone matrix.

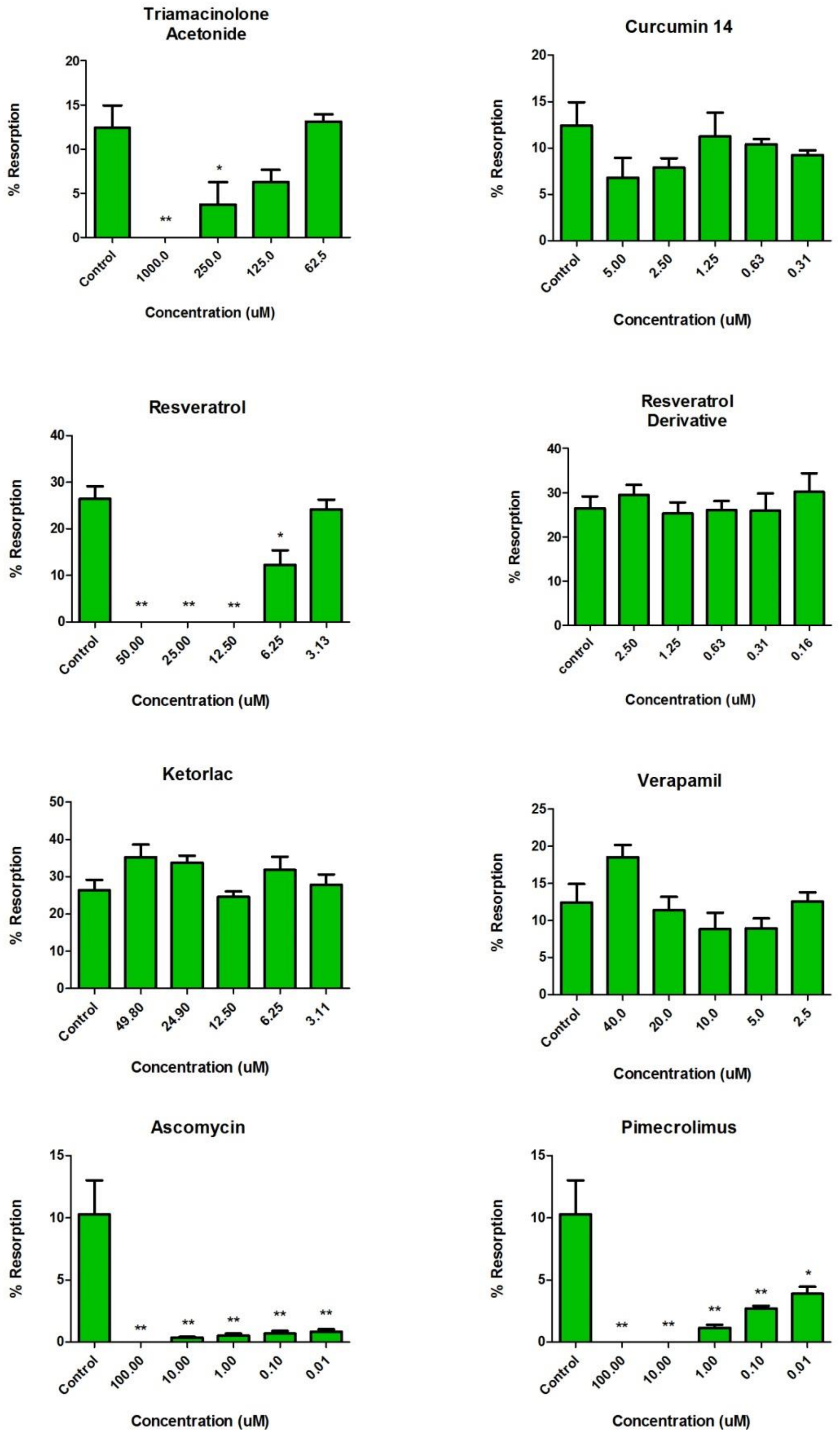

Figure 9 - Effects of different drugs on osteoclastogenesis and degradation of bone matrix.

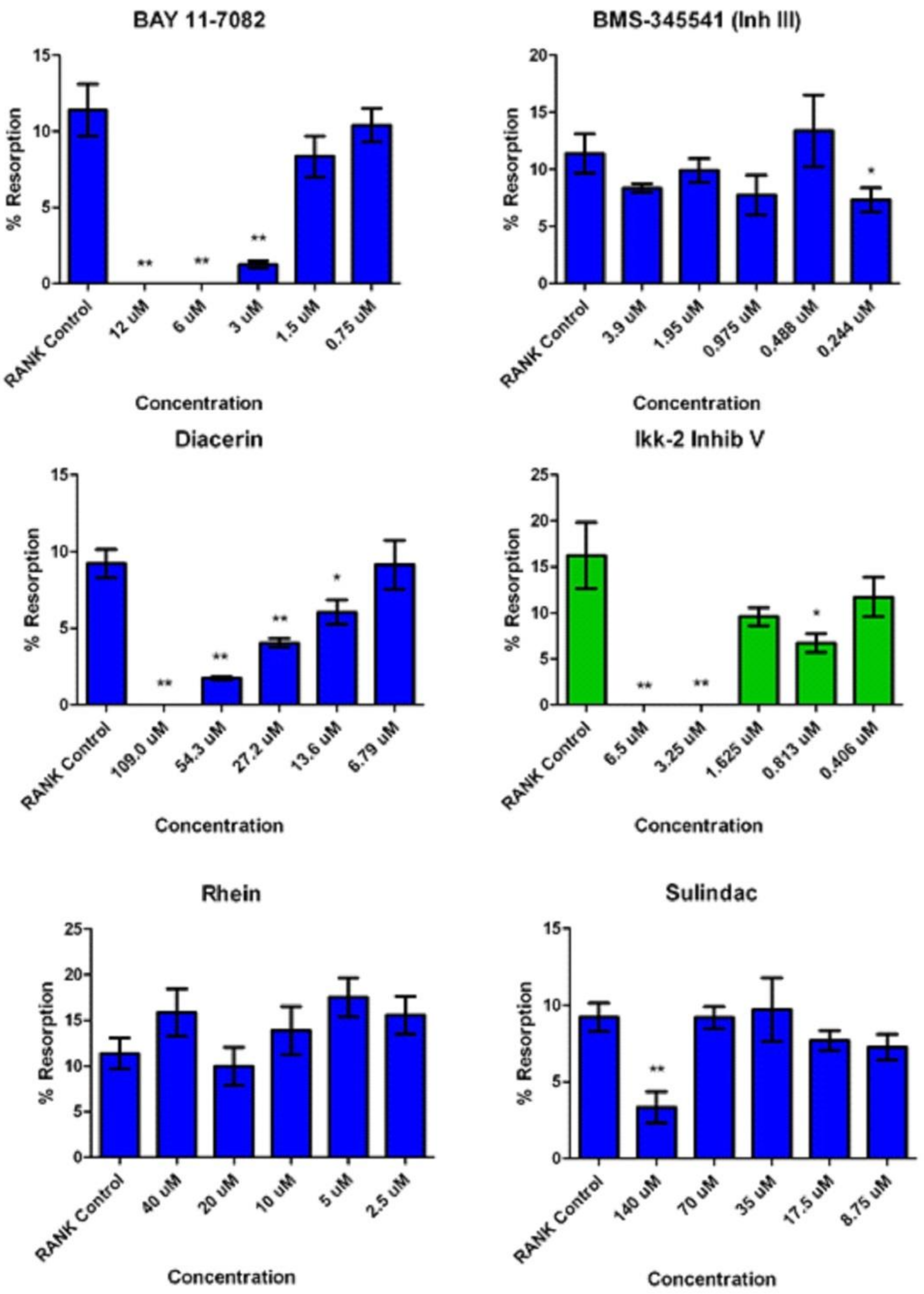

In Vitro MMP-13 Activity

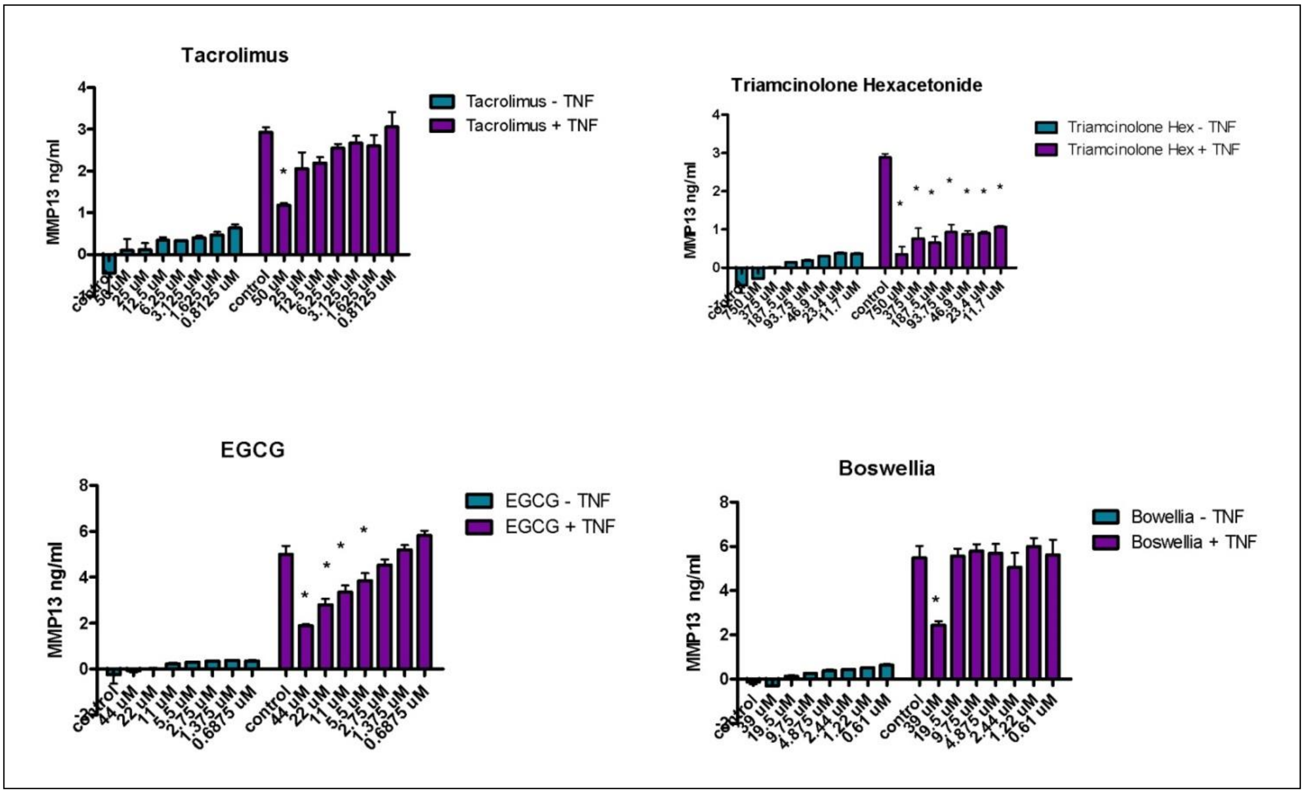

Figure 10 - Effects of Epigallocatechin Gallate (EGCG), Tacrolimus, Triamcinolone Hexacetonide and 3-O-Acetyl-11-keto-β-Boswellic Acid (Boswellia) on MMP-13 levels.

Effects of Epigallocatechin Gallate (EGCG), Tacrolimus, Triamcinolone Hexacetonide and 3-O-Acetyl-11-keto-β-Boswellic Acid (Boswellia) on MMP-13 levels in cell culture supernatants from chondrogenic micro-pellets in the presence and absence of TNF-α stimulation. Shown is the mean +/- SD (n=3) at the indicated concentrations. (*: $p<0.05$, one-way ANOVA with Dunnet's post-test compared to no drug controls with and without TNF-α).

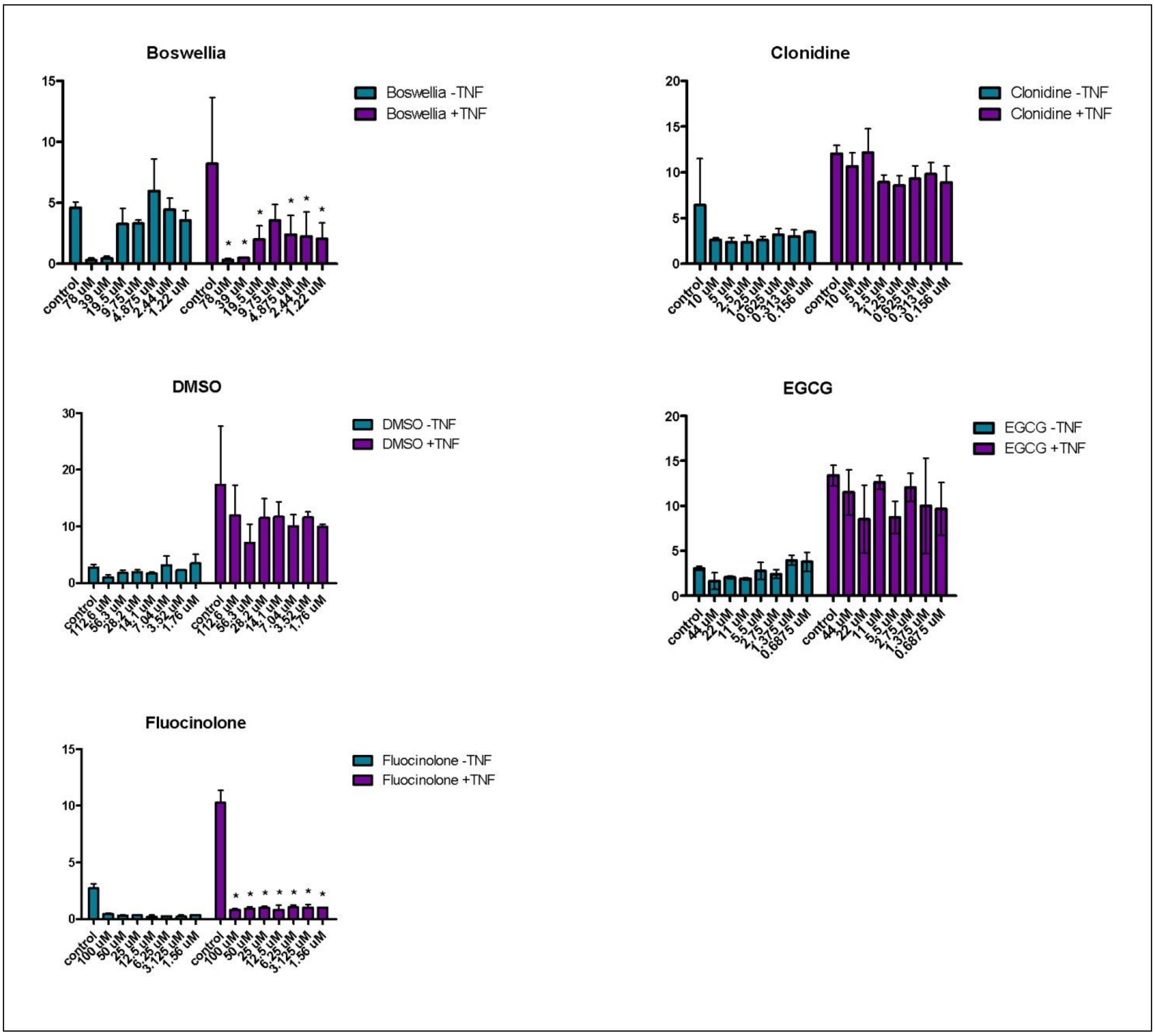

Figure 11 - Effects of Epigallocatechin Gallate (EGCG), Dimethyl Sulfoxide (DMSO), Clonidine, Fluocinolone and 3-O-Acetyl-11-keto-β-Boswellic Acid (Boswellia) on MMP-13 levels

Effects of Epigallocatechin Gallate (EGCG), Dimethyl Sulfoxide (DMSO), Clonidine, Fluocinolone and 3-O-Acetyl-11-keto-β-Boswellic Acid (Boswellia) on MMP-13 levels in cell culture supernatants from chondrogenic micro-pellets in the presence and absence of TNF-α stimulation. Shown is the mean +/- SD (n=3) at the indicated concentrations. (* : $p<0.05$, one-way ANOVA with Dunnet's post-test compared to no drug controls with and without TNF-α).

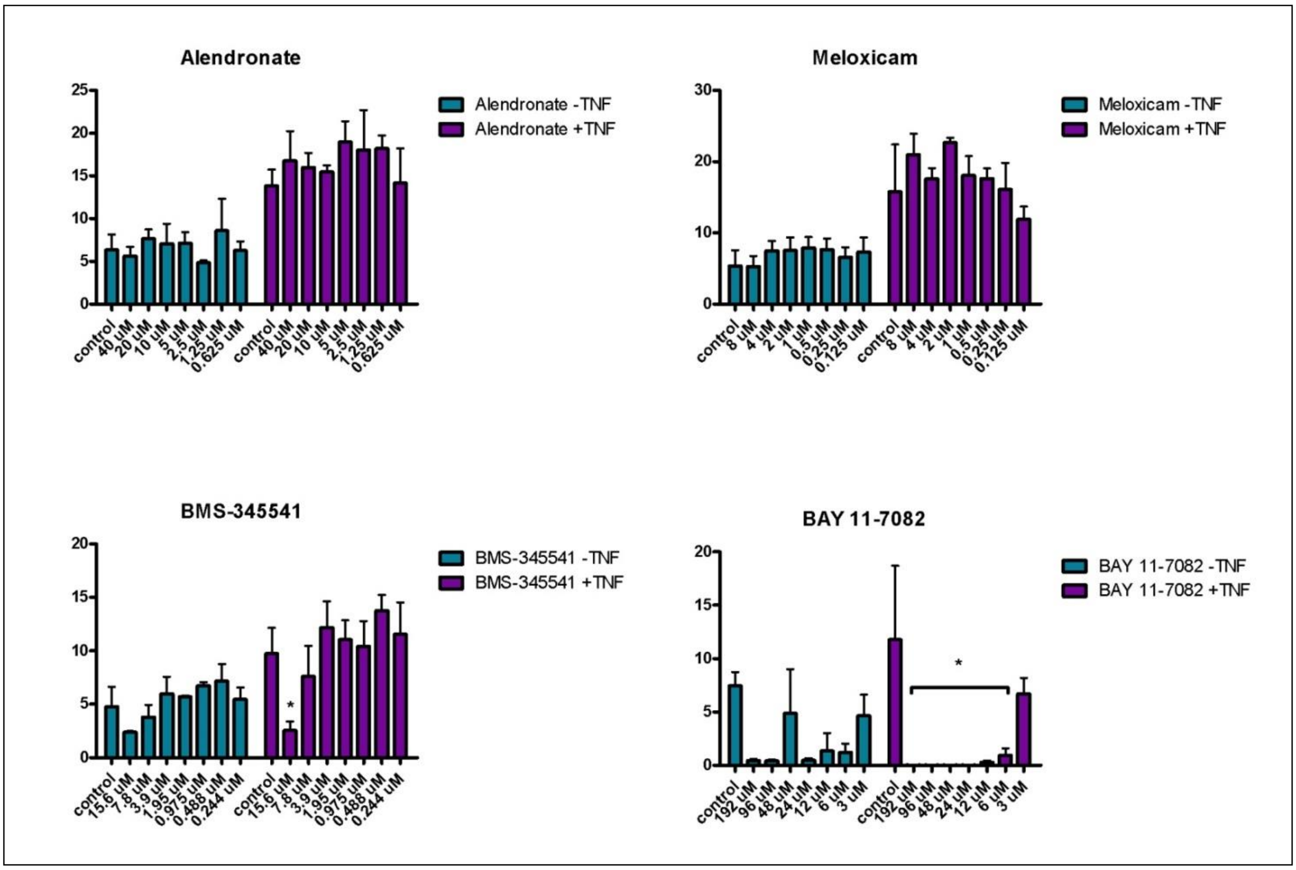

Figure 12 - Effects of BAY 11-7082, BMS-345541, Alendronate and Meloxicam on MMP-13 levels.

Effects of BAY 11-7082, BMS-345541, Alendronate and Meloxicam on MMP-13 levels in cell culture supernatants from chondrogenic micro-pellets in the presence and absence of TNF-α stimulation. Shown is the mean +/- SD (n=3) at the indicated concentrations. (* : $p<0.05$, one-way ANOVA with Dunnet's post-test compared to no drug controls with and without TNF-α). Note that Bay 11-7082 and BMS-345541 and were repeated (Figure 16 and 17, respectively).

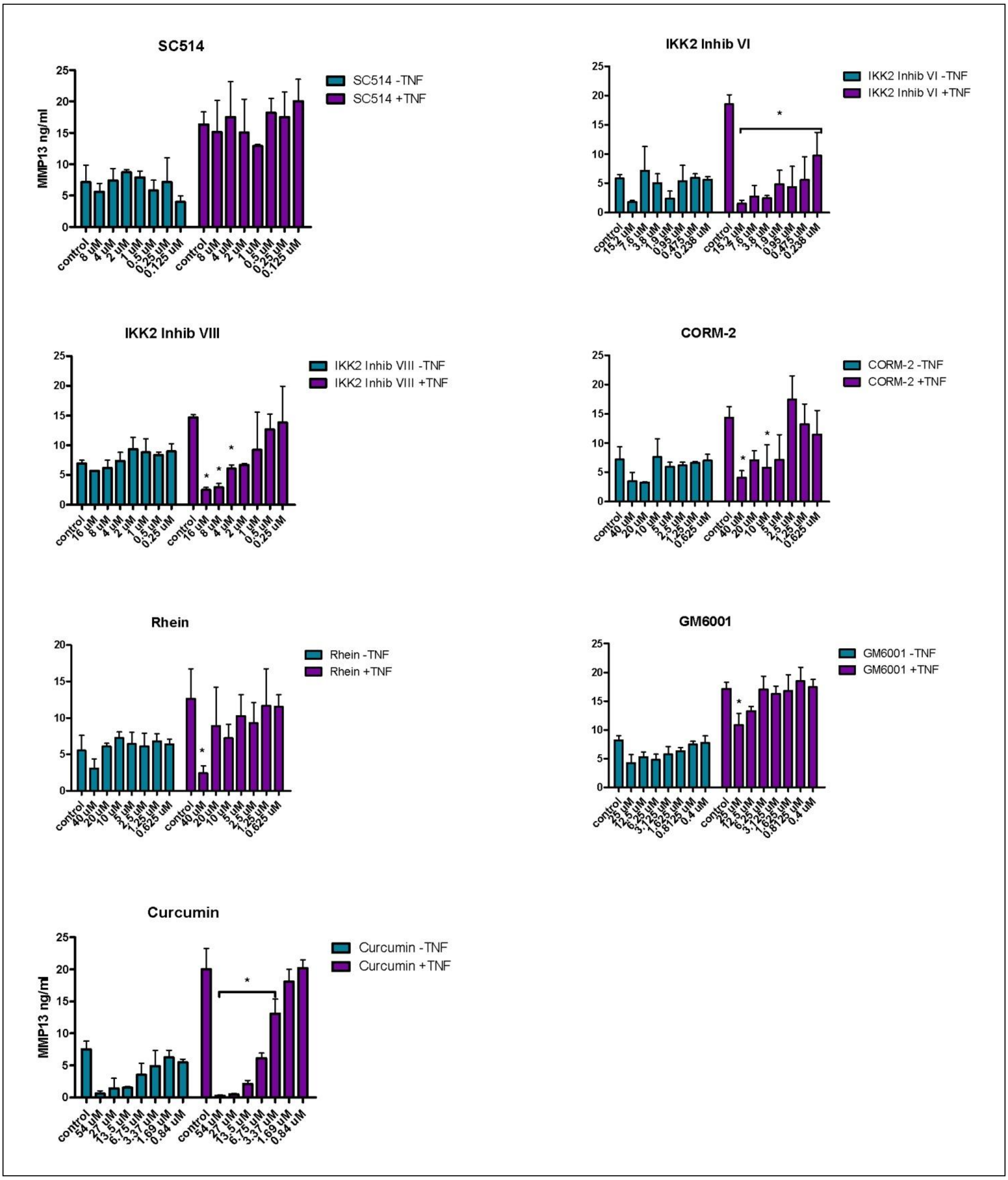

Figure 13 - Effects of IKK-2 Inhibitor VI, IKK-2 Inhibitor VIII, CORM-2, Rhein, GM6001, Curcumin and SC514 on MMP-13 levels.

Effects of IKK-2 Inhibitor VI, IKK-2 Inhibitor VIII, CORM-2, Rhein, GM6001, Curcumin and SC514 on MMP-13 levels in cell culture supernatants from chondrogenic micro-pellets in the presence and absence of TNF-α stimulation. Shown is the mean +/- SD (n=3) at the indicated concentrations. (* : $p<0.05$, one-way ANOVA with Dunnet's post-test compared to no drug controls with and without TNF-α).

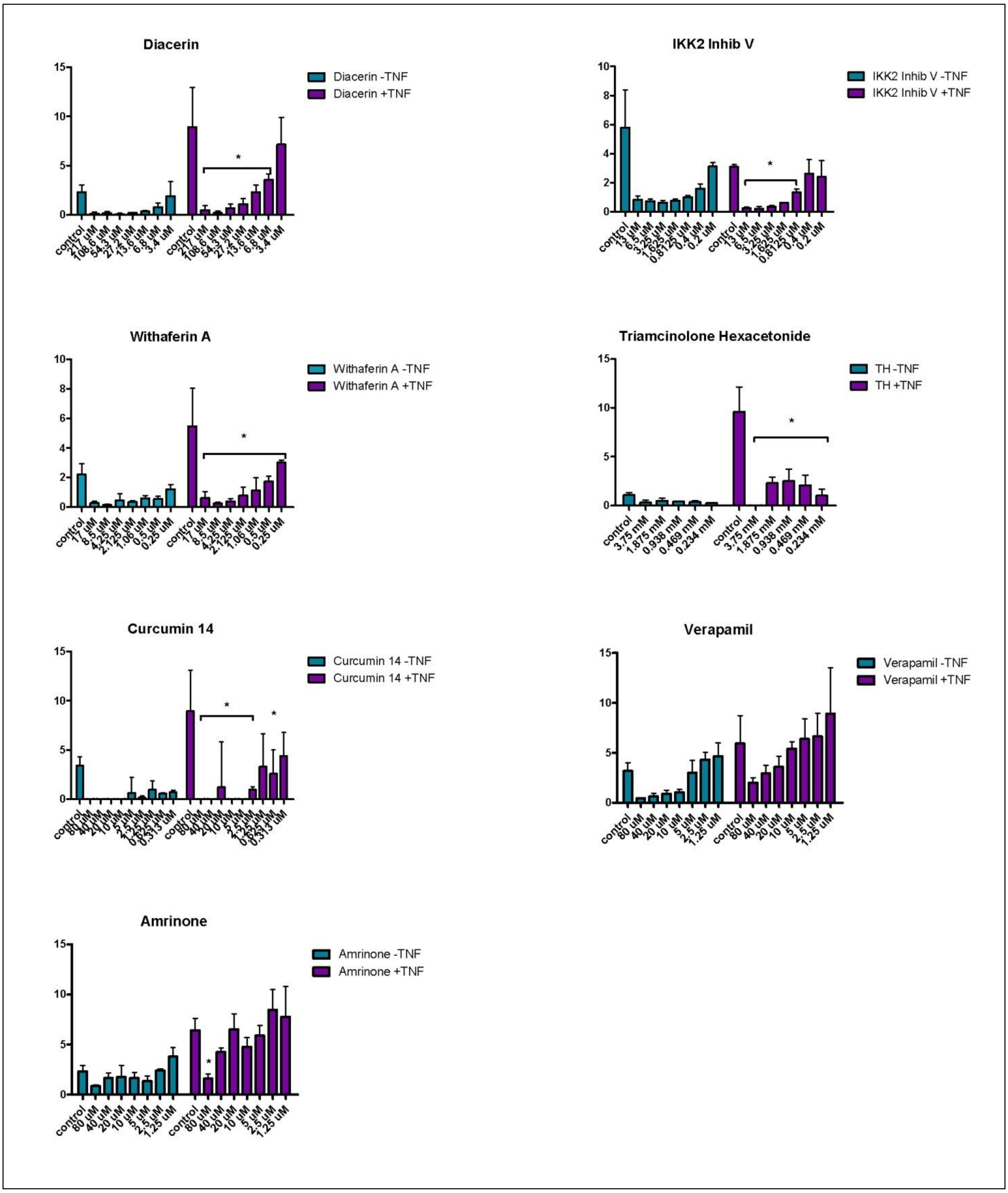

Figure 14 - Effects of Diacerin, IKK-2 Inhibitor V, Withaferin A, Triamcinolone Hexacetonide, Curcumin 14 and Amrinone on MMP-13 levels.

Effects of Diacerin, IKK-2 Inhibitor V, Withaferin A, Triamcinolone Hexacetonide, Curcumin 14 and Amrinone on MMP-13 levels in cell culture supernatants from chondrogenic micro-pellets in the presence and absence of TNF-α stimulation. Shown is the mean +/- SD (n=3) at the indicated concentrations. (* : $p<0.05$, one-way ANOVA with Dunnet's post-test compared to no drug controls with and without TNF-α).

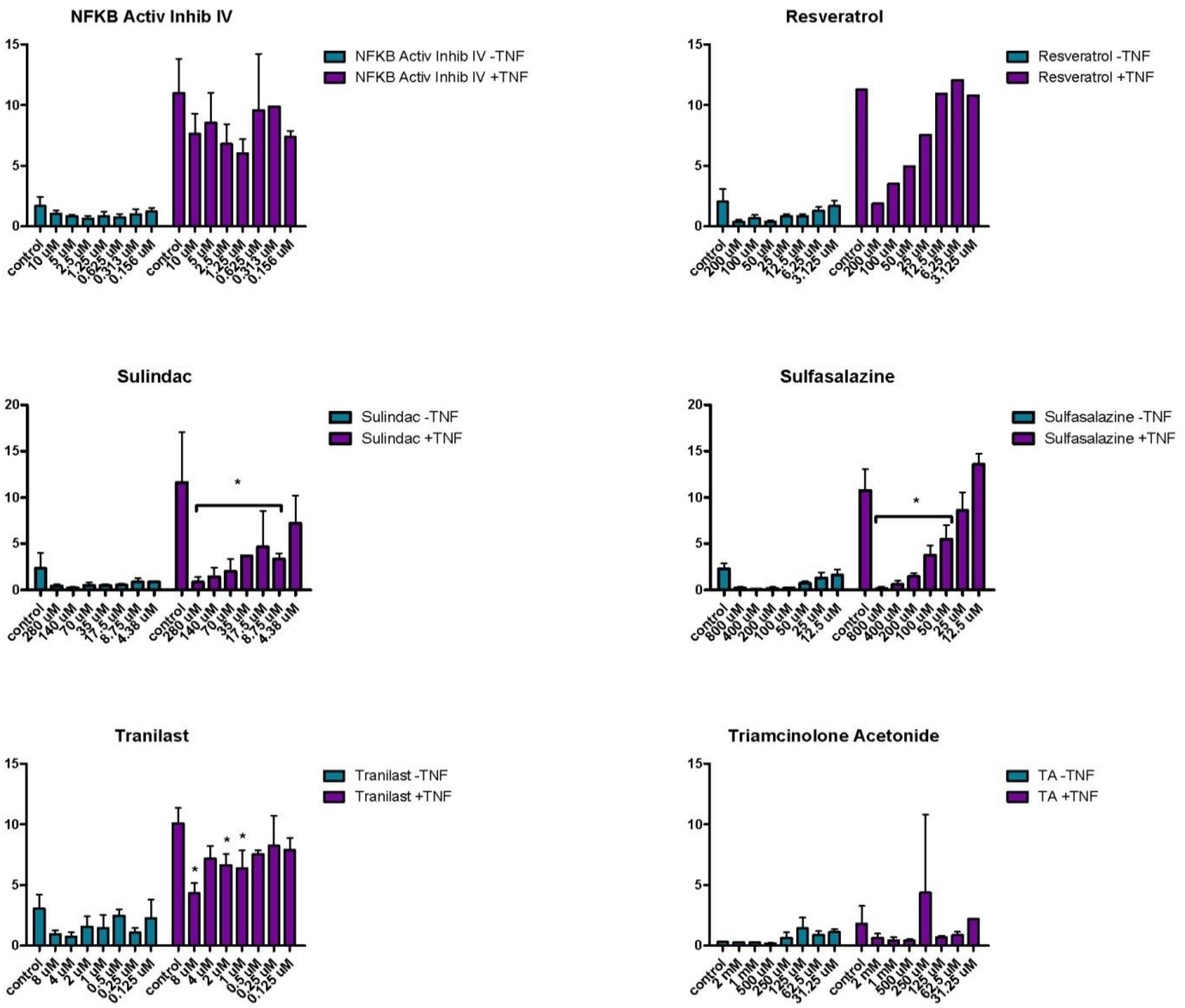

Figure 15 - Effects of Triamcinolone Acetonide, NFkB Activation Inhibitor IV, Sulindac, Sulfasalazine, Tranilast and Resveratrol on MMP-13 levels.

Effects of Triamcinolone Acetonide (TA), NFkB Activation Inhibitor IV, Sulindac, Sulfasalazine, Tranilast and Resveratrol on MMP-13 levels in cell culture supernatants from chondrogenic micro-pellets in the presence and absence of TNF-α stimulation. Shown is the mean +/- SD (n=3) at the indicated concentrations. (* : $p<0.05$, one-way ANOVA with Dunnet's post-test compared to no drug controls with and without TNF-α). The resveratrol + TNF-α group is only n=1 due to lack of viable pellets for the assay. Note that TA was repeated in the experiment of Figure 18.

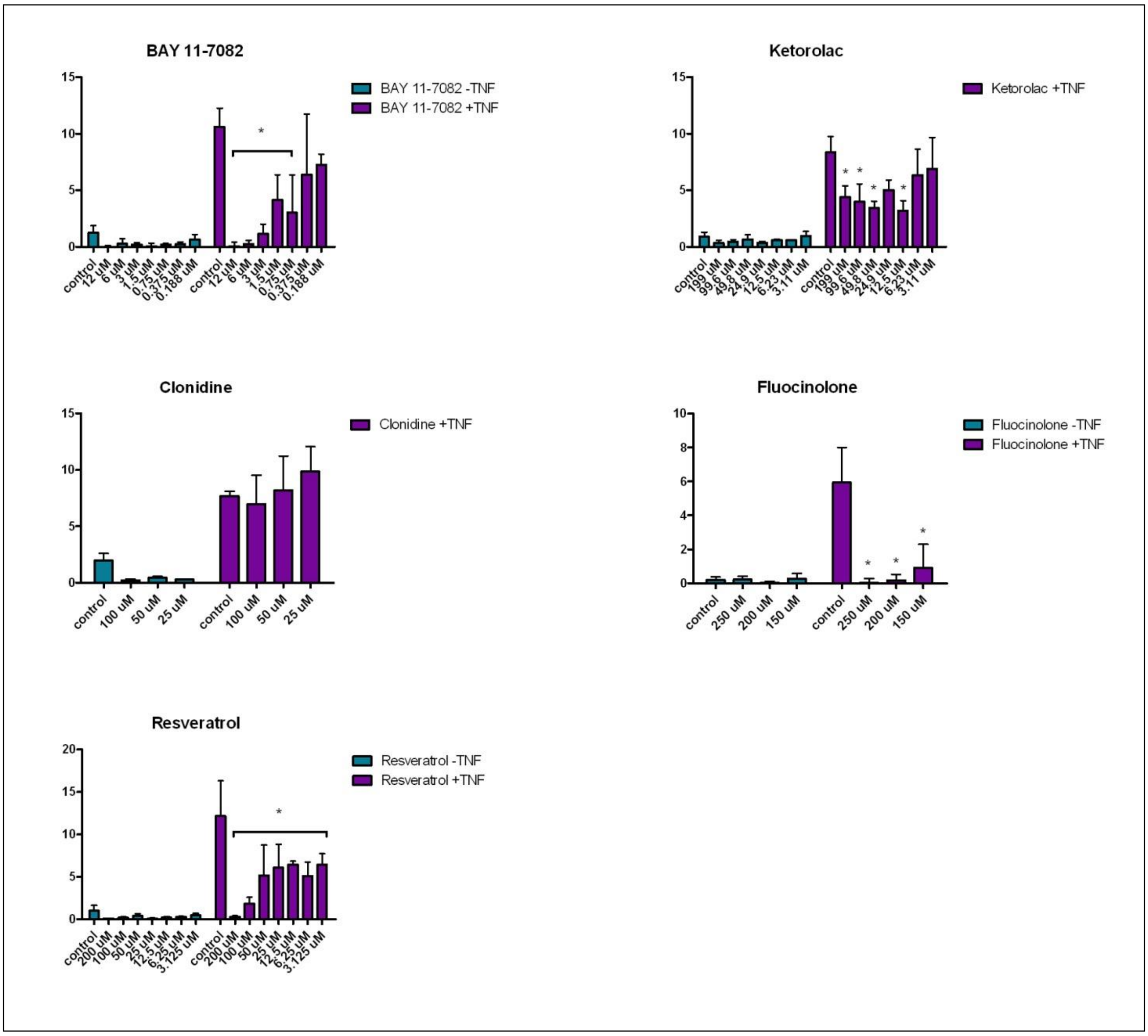

Figure 16 - Effects of BAY 11-7082, Ketorolac, Clonidine, Resveratrol, and Fluocinolone on MMP-13 levels.

Effects of BAY 11-7082, Ketorolac, Clonidine, Resveratrol, and Fluocinolone on MMP-13 levels in cell culture supernatants from chondrogenic micro-pellets in the presence and absence of TNF-α stimulation (experiment #9). Shown is the mean +/- SD (n=3) at the indicated concentrations. (* : $p<0.05$, one-way ANOVA with Dunnet's post-test compared to no drug controls with and without TNF-α).

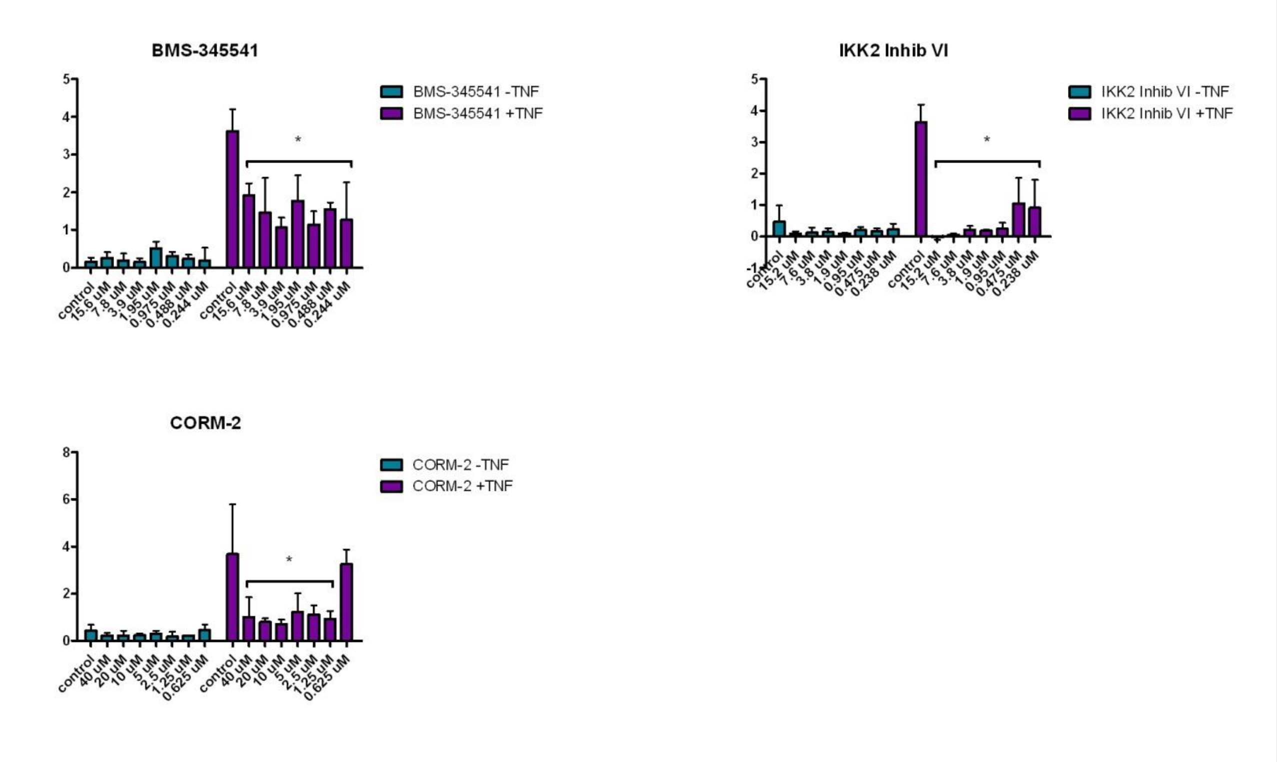

Figure 17 - Effects of BMS-345541 (IKK-2 Inhibitor III), IKK-2 Inhibitor VI and CORM-2 on MMP-13 levels.

Effects of BMS-345541 (IKK-2 Inhibitor III), IKK-2 Inhibitor VI and CORM-2 on MMP-13 levels in cell culture supernatants from chondrogenic micro-pellets in the presence and absence of TNF-α stimulation. Shown is the mean +/- SD (n=3) at the indicated concentrations. (* : $p<0.05$, one-way ANOVA with Dunnet's post-test compared to no drug controls with and without TNF-α).

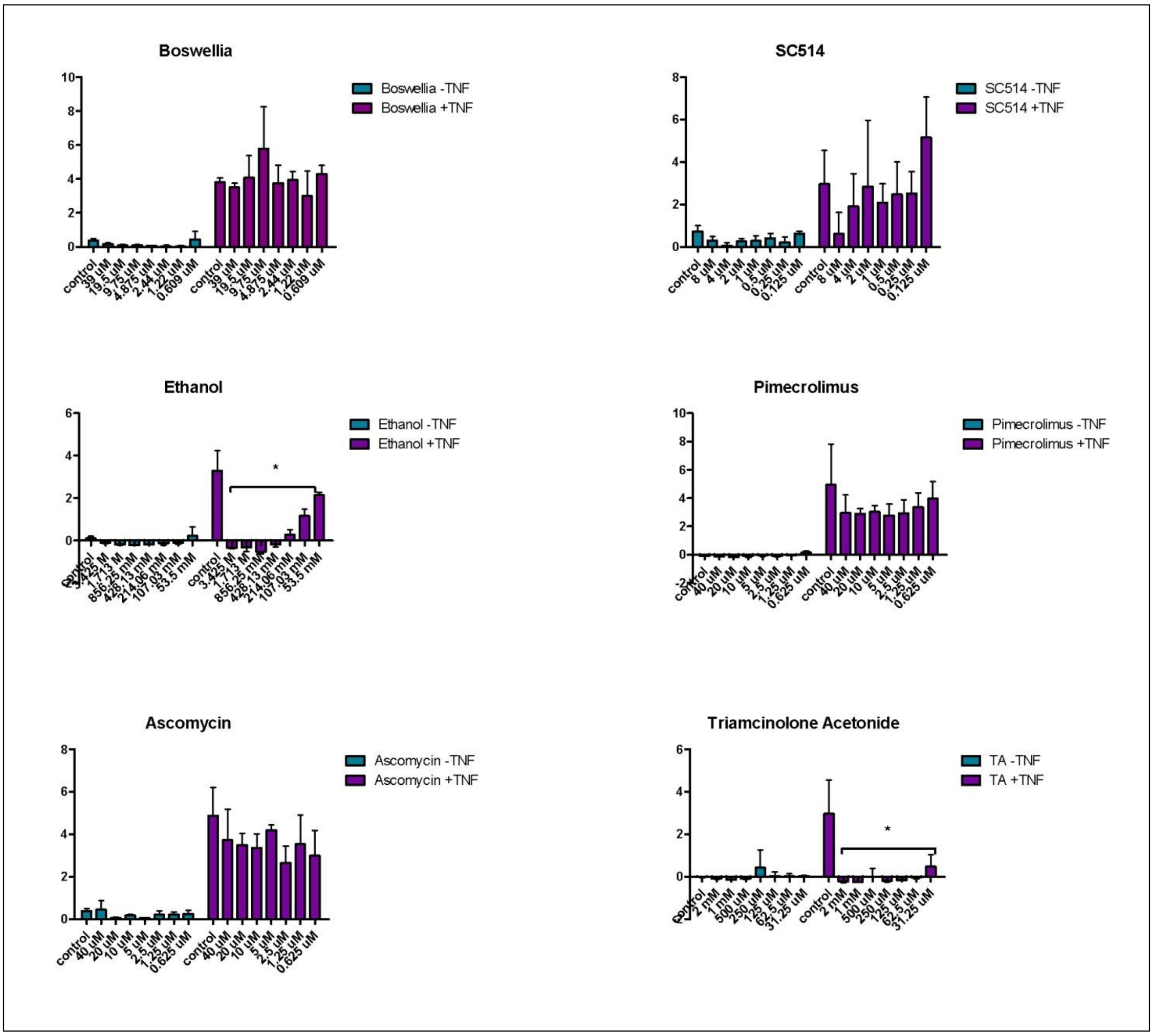

Figure 18 - Effects of Pimecrolimus, Ethanol, Ascomycin, Boswellia, SC514 and Triamcinolone Acetonide on MMP-13 levels.

Effects of Pimecrolimus, Ethanol, Ascomycin, Boswellia, SC514 and Triamcinolone Acetonide on MMP-13 levels in cell culture supernatants from chondrogenic micro-pellets in the presence and absence of TNF-α stimulation. Shown is the mean +/- SD (n=3) at the indicated concentrations. (*: $p<0.05$, one-way ANOVA with Dunnet's post-test compared to no drug controls with and without TNF-α). Triamcinolone Acetonide was repeated in this set of assays due to a problem with the control wells in Figure 15.

In Vitro Toxicity Testing

In Vitro Toxicity Testing with a Synovial Cell Line

Compounds were tested for synovial toxicity using SW982 cells alone or in the presence of TNF as shown in **Figures 19-27** below. Most agents were toxic only at very high concentrations, with Bay11-7082 demonstrating notable toxicity at 12 μM. This compound was much less toxic to HeLa cells, as shown in **Table 1.**

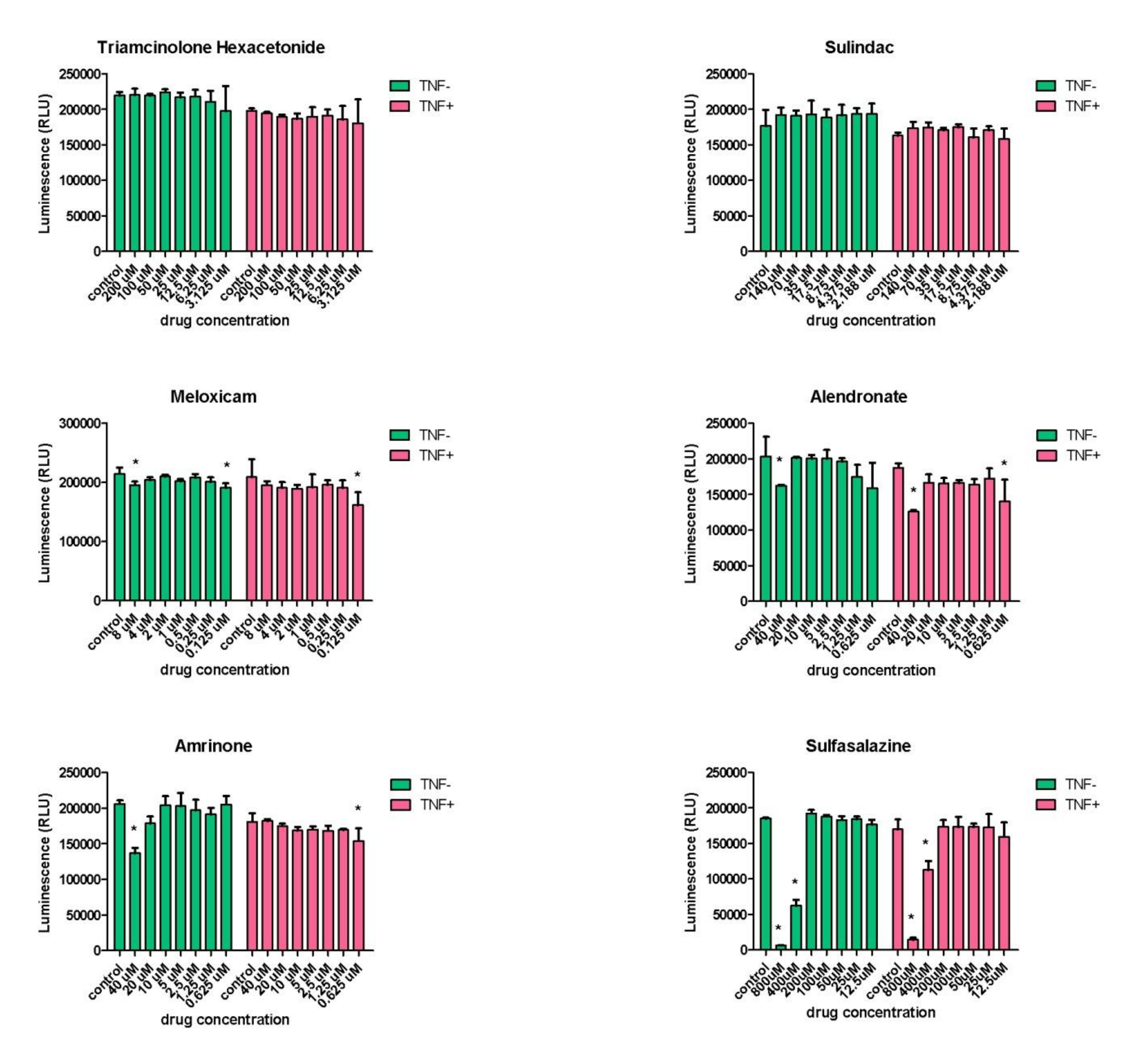

Figure 19 – Effects of Triamcinolone Hexacetonide, Sulindac, Meloxicam, Alendronate, Amrinone and Sulfasalazine on viability of synovial sarcoma cells (SW982) +/-TNF-alpha

The effects of Triamcinolone Hexacetonide, Sulindac, Meloxicam, Alendronate, Amrinone and Sulfasalazine on the viability of synovial sarcoma cells (SW982) in the presence and absence of TNF-alpha stimulation are shown in **Figure 19.** Shown is the mean +/- SD (n=3) at the indicated concentrations. ($p<0.05$, one-way ANOVA with Dunnet's post-test compared to no drug controls with and without TNF-alpha).

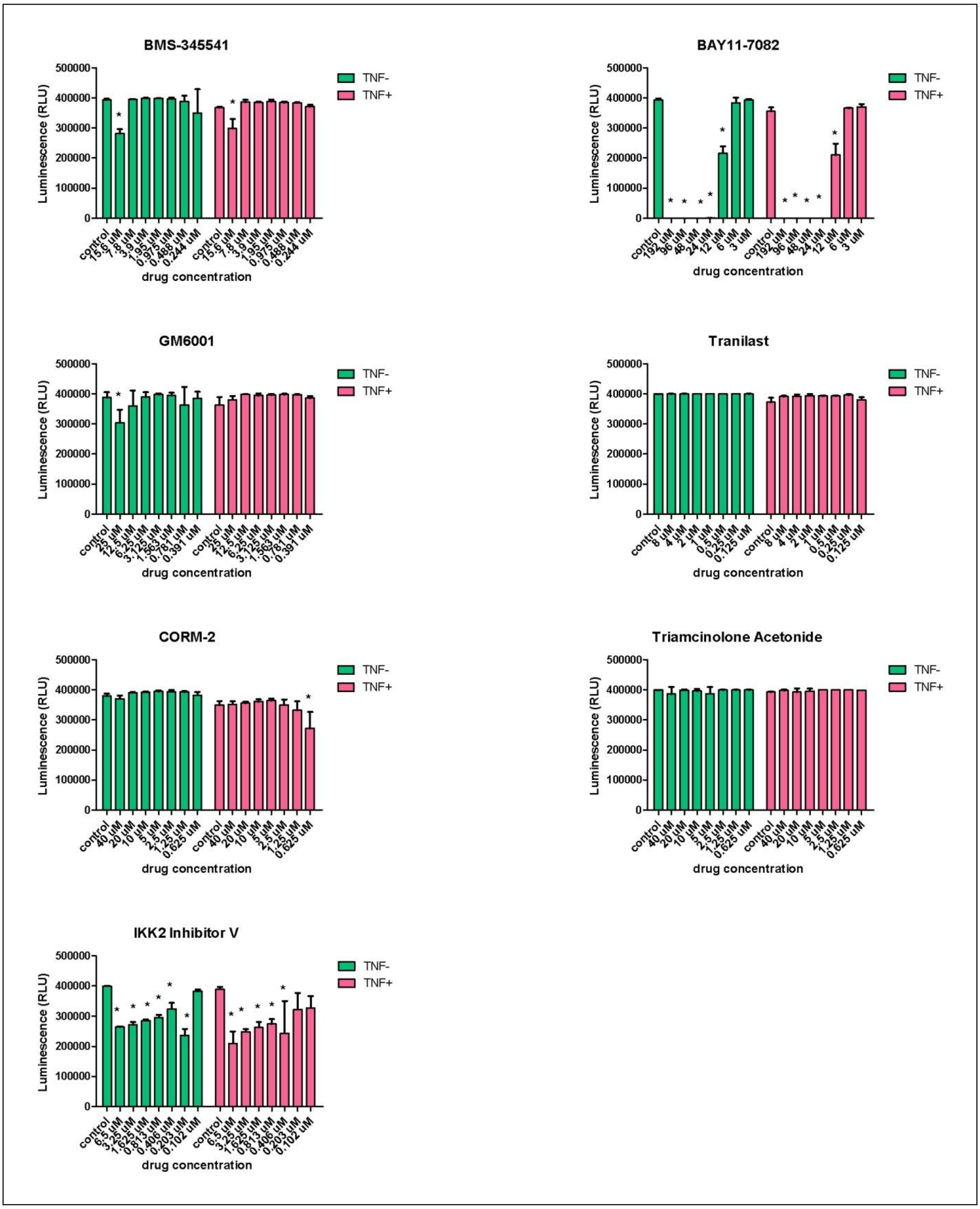

Figure 20 – Effects of BMS-345541, BAY 11-7082, GM6001, Tranilast, CORM-2, Triamcinolone Acetonide, and IKK2 Inhibitor V on the viability of synovial sarcoma cells (SW982) +/-TNF-alpha.

The effects of BMS-345541, BAY 11-7082, GM6001, Tranilast, CORM-2, Triamcinolone Acetonide, and IKK2 Inhibitor V on the viability of synovial sarcoma cells (SW982) +/-TNF-alpha are shown in **Figure 20** above. Shown is the mean +/- SD (n=3) at the indicated concentrations. (*: p<0.05, one-way ANOVA with Dunnet's post-test compared to no drug controls with and without TNF-alpha). The several assays that exceeded detection limits were repeated and results are shown in **Figures 21 and 22.**

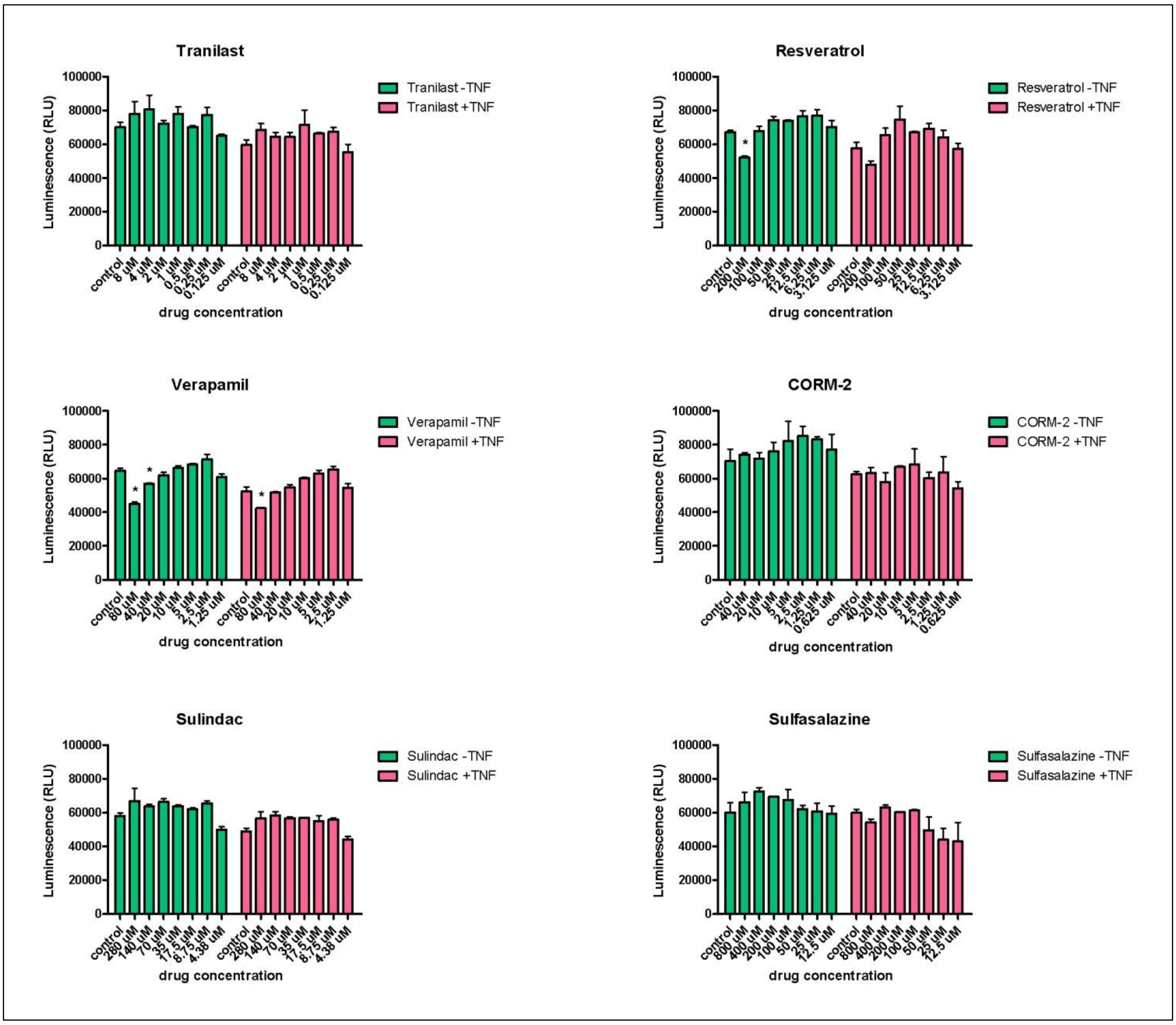

Figure 21 – Effects of Tranilast, Resveratrol, Verapamil, CORM-2, Sulindac, and Sulfasalazine on the viability of SW982 cells +/- TNF alpha.

The effects of Tranilast, Resveratrol, Verapamil, CORM-2, Sulindac, and Sulfasalazine on the viability of synovial sarcoma cells (SW982) in the presence and absence of TNF-alpha stimulation are shown above. Shown is the mean +/- SD (n=3) at the indicated concentrations. (*$p<0.05$, one-way ANOVA with Dunnet's post-test compared to no drug controls with and without TNF-alpha).

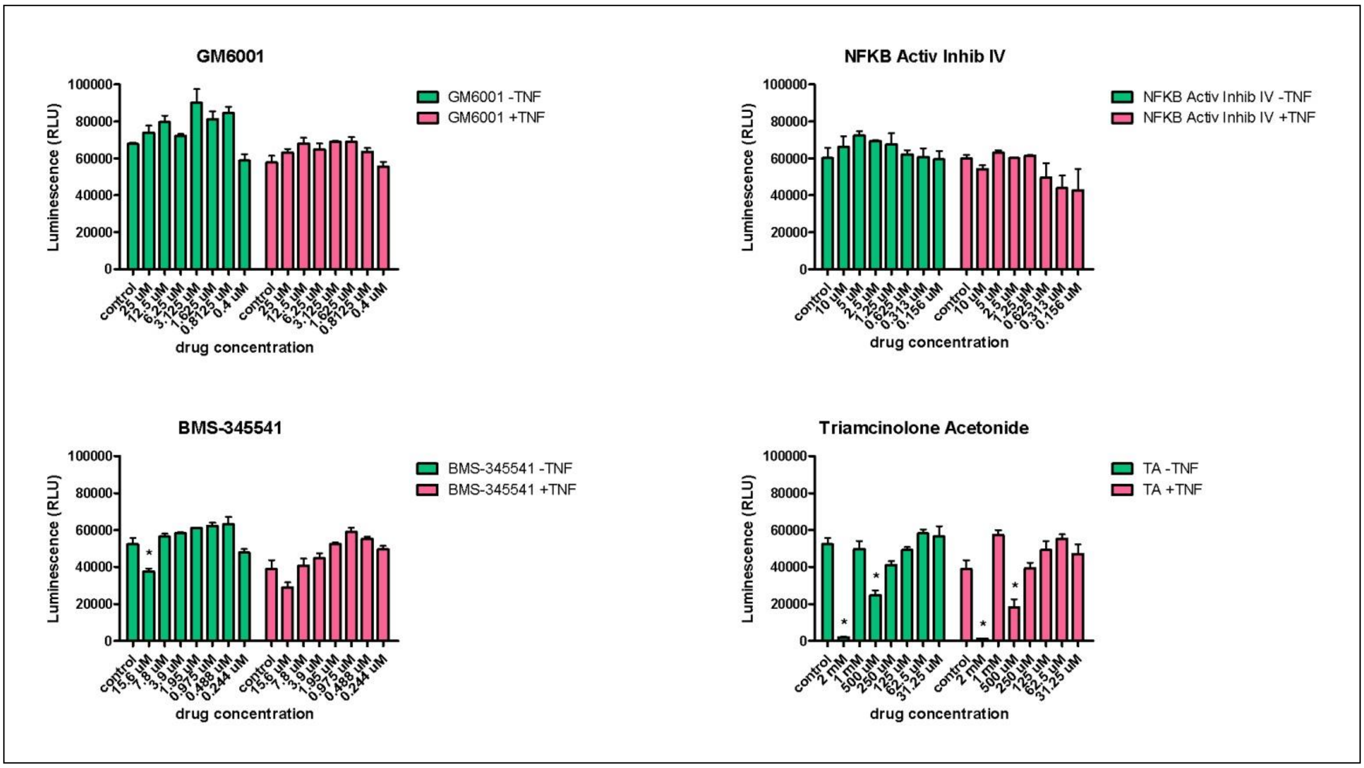

Figure 22 - Effects of GM6001, NFkB Activation Inhibitor IV, BMS-345541 and Triamcinolone Acetonide on the viability of SW982 cells +/- TNF-alpha.

The effects of GM6001, NFkB Activation Inhibitor IV, BMS-345541 and Triamcinolone Acetonide on the viability of synovial sarcoma cells (SW982) in the presence and absence of TNF-alpha stimulation are shown in **Figure 22** above. Shown is the mean +/- SD (n=3) at the indicated concentrations. (*$p<0.05$, one-way ANOVA with Dunnet's post-test compared to no drug controls with and without TNF-alpha).

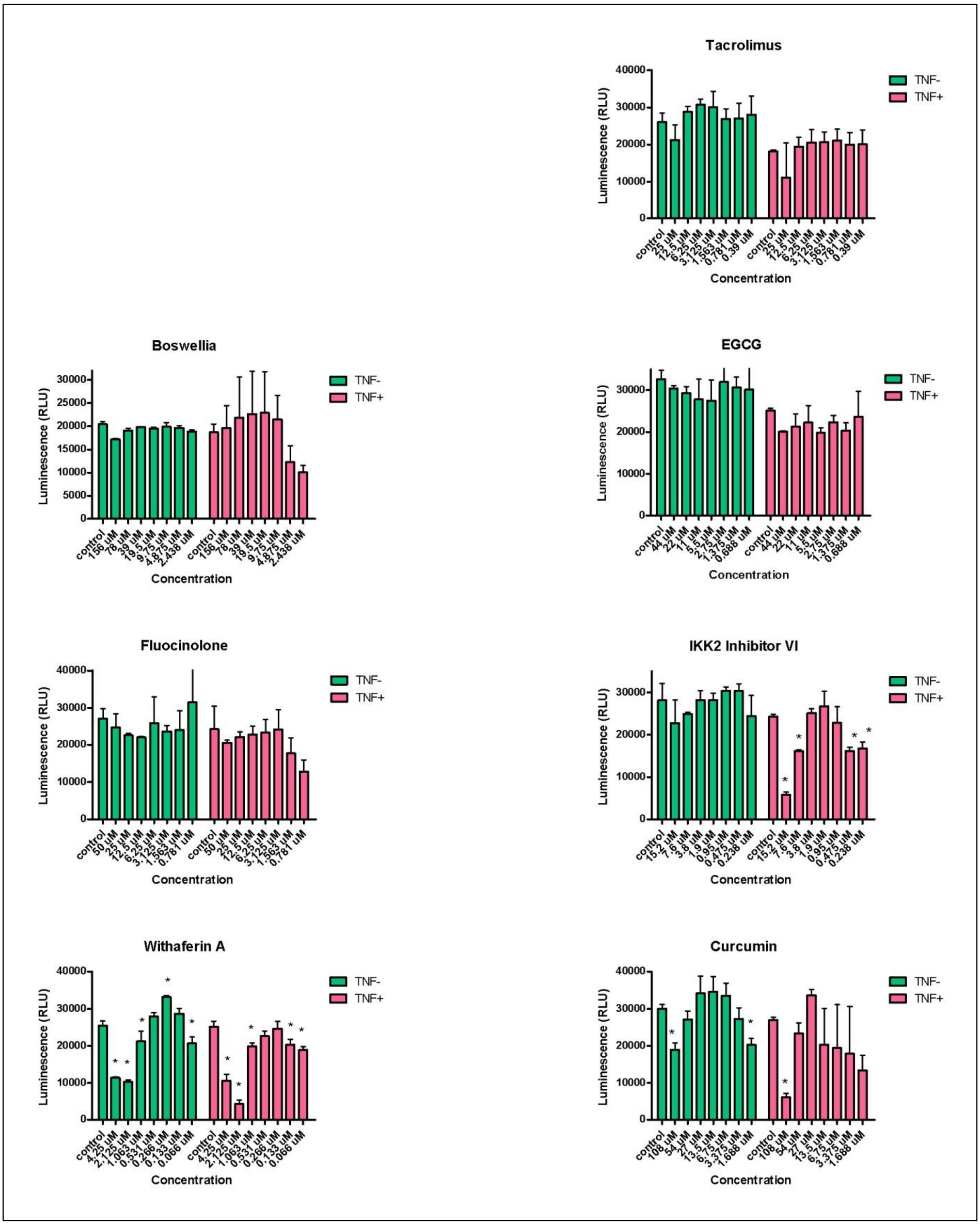

Figure 23 – The effects of Tacrolimus, Boswellia, EGCG, Fluocinolone, IKK2 Inhibitor VI, Withaferin A and Curcumin on the viability of SW982 cells +/- TNF-alpha.

The effects of Tacrolimus, Boswellia, EGCG, Fluocinolone, IKK2 Inhibitor VI, Withaferin A and Curcumin on the viability of synovial sarcoma cells (SW982) in the presence and absence of TNF-alpha stimulation. Shown in **Figure 23** is the mean +/- SD (n=3) at the indicated concentrations. (*$p<0.05$, one-way ANOVA with Dunnet's post-test compared to no drug controls with and without TNF-alpha).

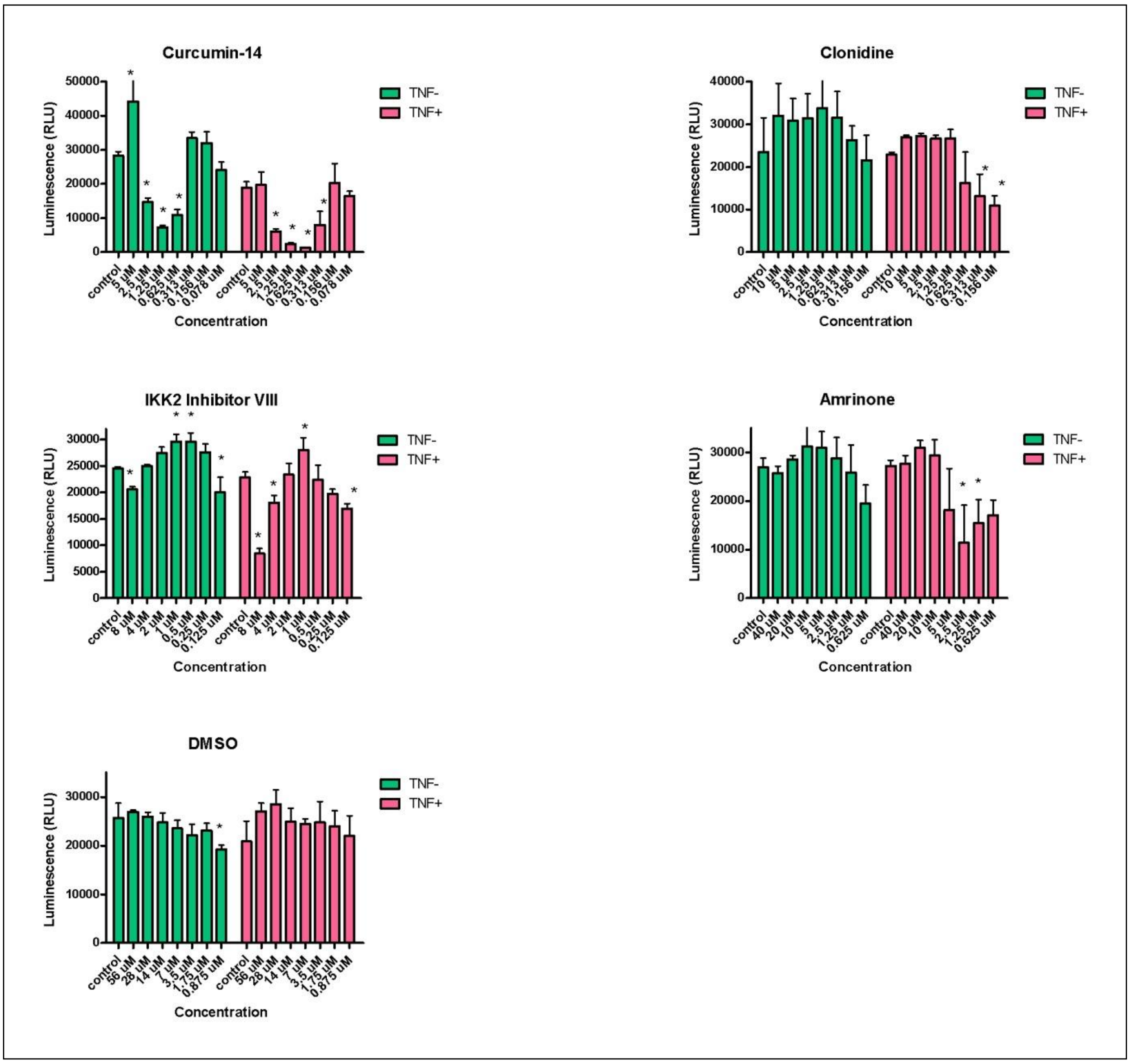

Figure 24 – The effects of Curcumin-14, Clonidine, IKK2 Inhibitor VIII, Amrinone and DMSO on the viability of SW982 cells +/- TNF alpha.

Shown in **Figure 24** above are the effects of Curcumin-14, Clonidine, IKK2 Inhibitor VIII, Amrinone and DMSO on the viability of synovial sarcoma cells (SW982) in the presence and absence of TNF-alpha stimulation. Shown is the mean +/- SD (n=3) at the indicated concentrations. (* $p<0.05$, one-way ANOVA with Dunnet's post-test compared to no drug controls with and without TNF-alpha).

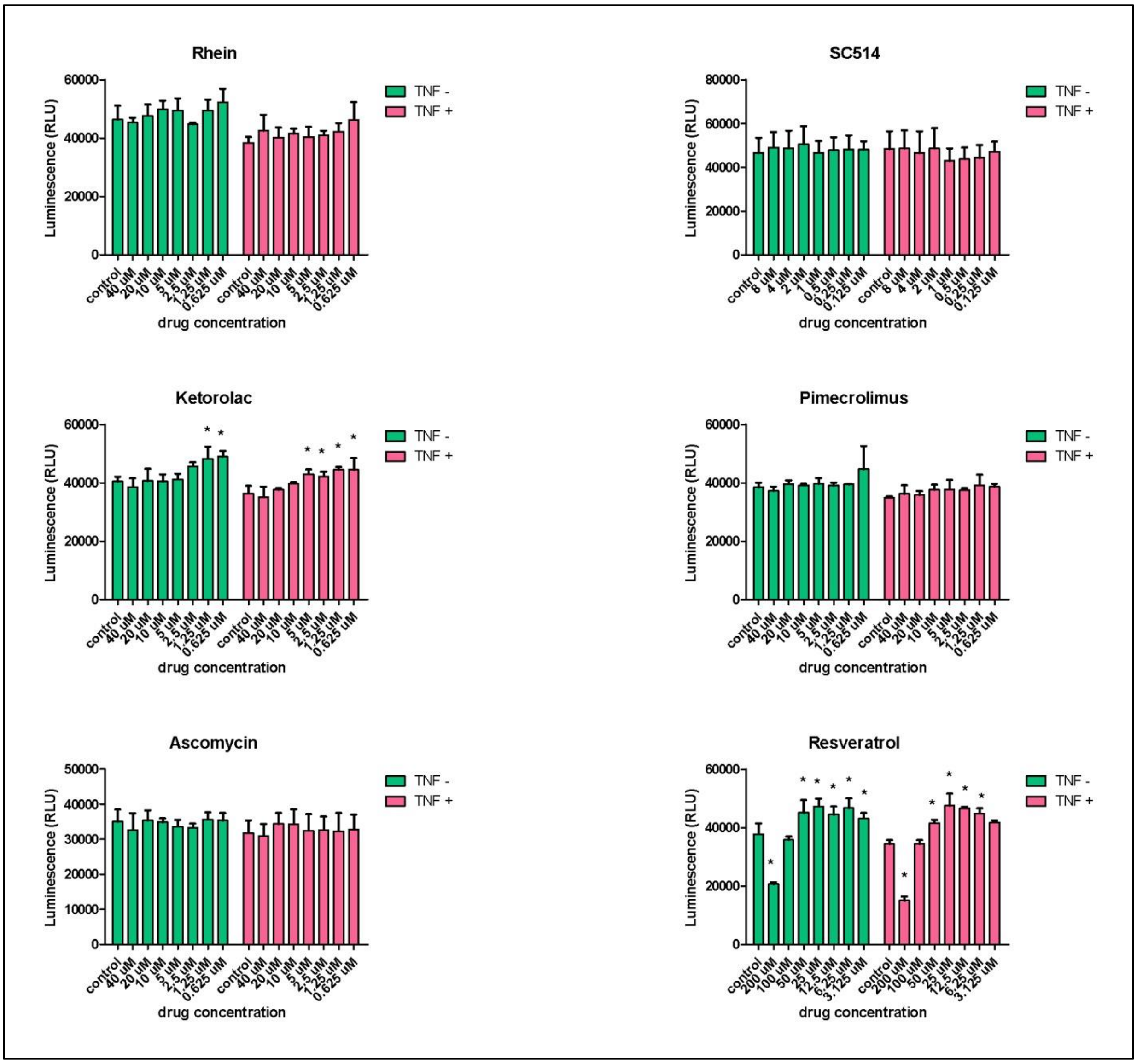

Figure 25 – The effects of Rhein, SC514, Ketorolac, Pimecrolimus, Resveratrol and Ascomycin on the viability of SW982 cells +/- TNF- alpha. Note that Resveratrol stimulated cell growth resulting in significantly higher luminescence readings.

The effects of Rhein, SC514, Ketorolac, Pimecrolimus, Resveratrol and Ascomycin on the viability of synovial sarcoma cells (SW982) in the presence and absence of TNF-alpha stimulation are shown above in **Figure 25**. Shown is the mean +/- SD (n=3) at the indicated concentrations. (*$p<0.05$, one-way ANOVA with Dunnet's post-test compared to no drug controls with and without TNF-alpha).

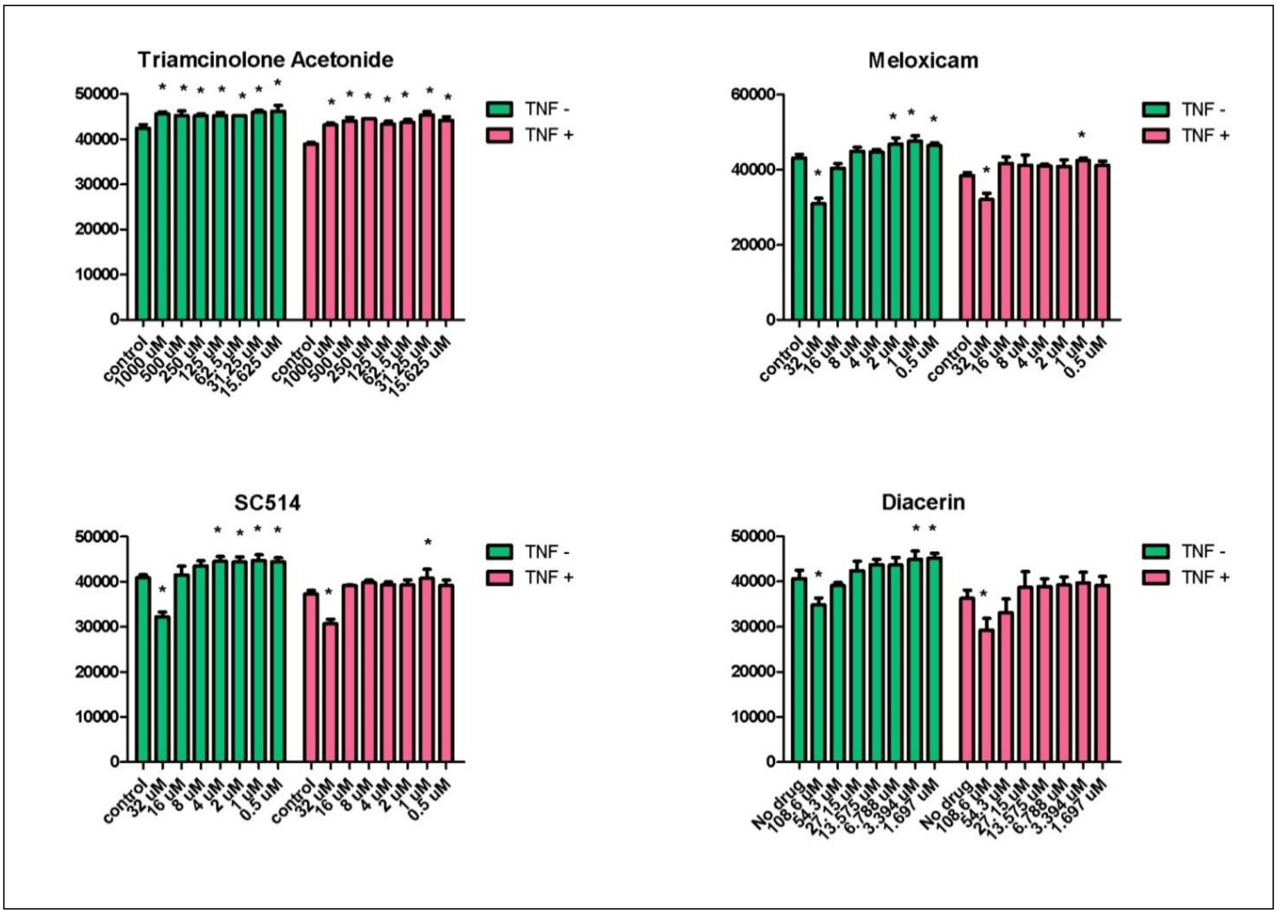

Figure 26 – The effects of Triamcinolone Acetonide, Meloxicam, SC514, and Diacerin on the viability of SW982 cells +/- TNF alpha.

The effects of Triamcinolone Acetonide, Meloxicam, SC514, and Diacerin on the viability of synovial sarcoma cells (SW982) in the presence and absence of TNF-alpha stimulation are shown above in **Figure 26**. Shown is the mean +/- SD (n=3) at the indicated concentrations. (*$p<0.05$, one-way ANOVA with Dunnet's post-test compared to no drug controls with and without TNF-alpha).

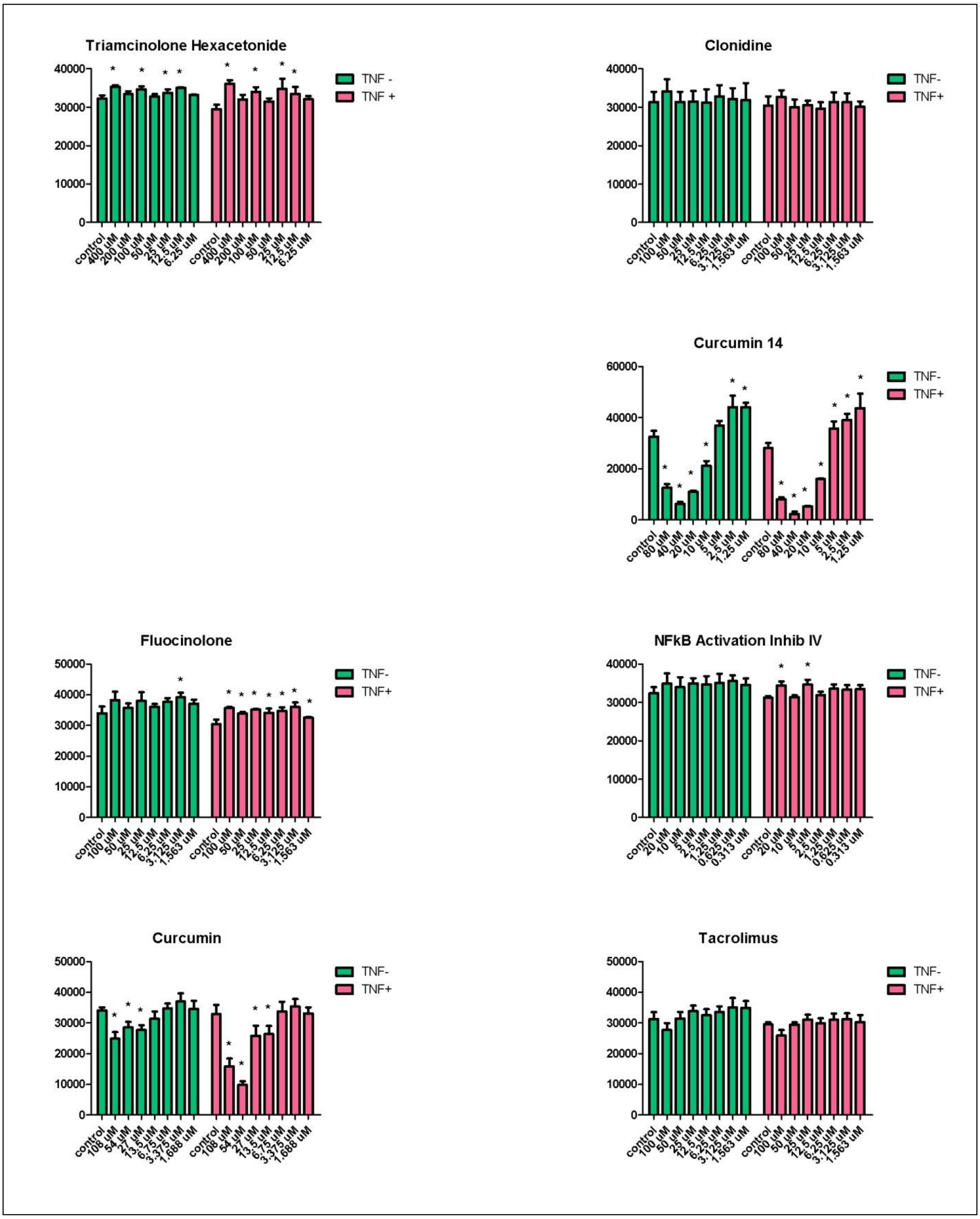

Figure 27 – The effects of Traimcinolone Hexacetonide, Clonidine, Curcumin-14, Fluocinolone, NFkB Activation Inhibitor, Curcumin and Tacrolimus on the viability of SW982 +/- TNF-alpha.

The effects of Traimcinolone Hexacetonide, Clonidine, Curcumin-14, Fluocinolone, NFkB Activation Inhibitor, Curcumin and Tacrolimus on the viability of SW982 in the presence and absence of TNF-alpha stimulation are shown above in **Figure 27**. Shown is the mean +/- SD (n=3) at the indicated concentrations. (*$p<0.05$, one-way ANOVA with Dunnet's post-test compared to no drug controls with and without TNF-alpha).

In Vitro Toxicity Testing with Differentiated Human Chondrocytes.

Agents were also tested for toxicity using differentiated human chondrocytes as described in the manuscript.

Figure 28. Drug toxicity testing with chondrogenic pellets for agents tested in vivo.

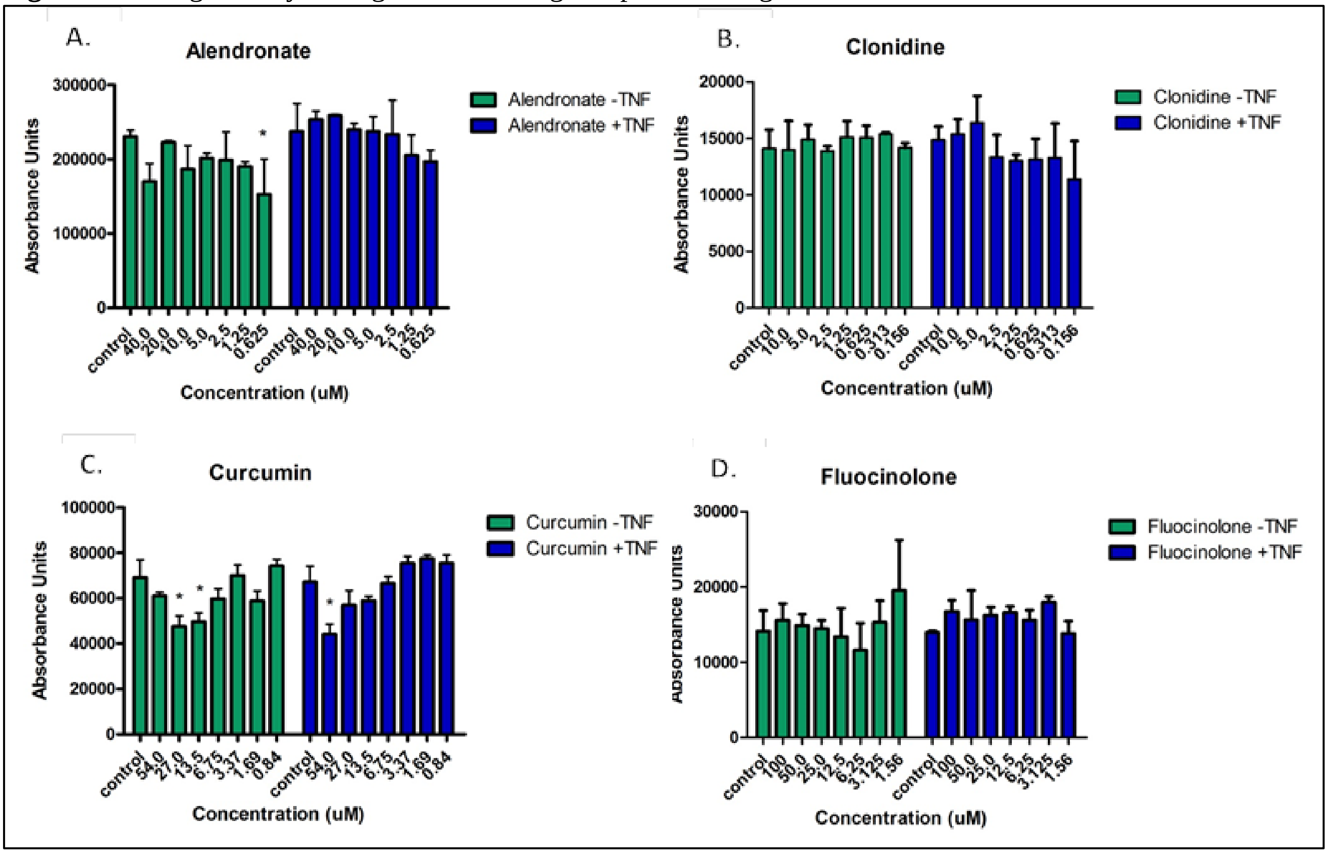

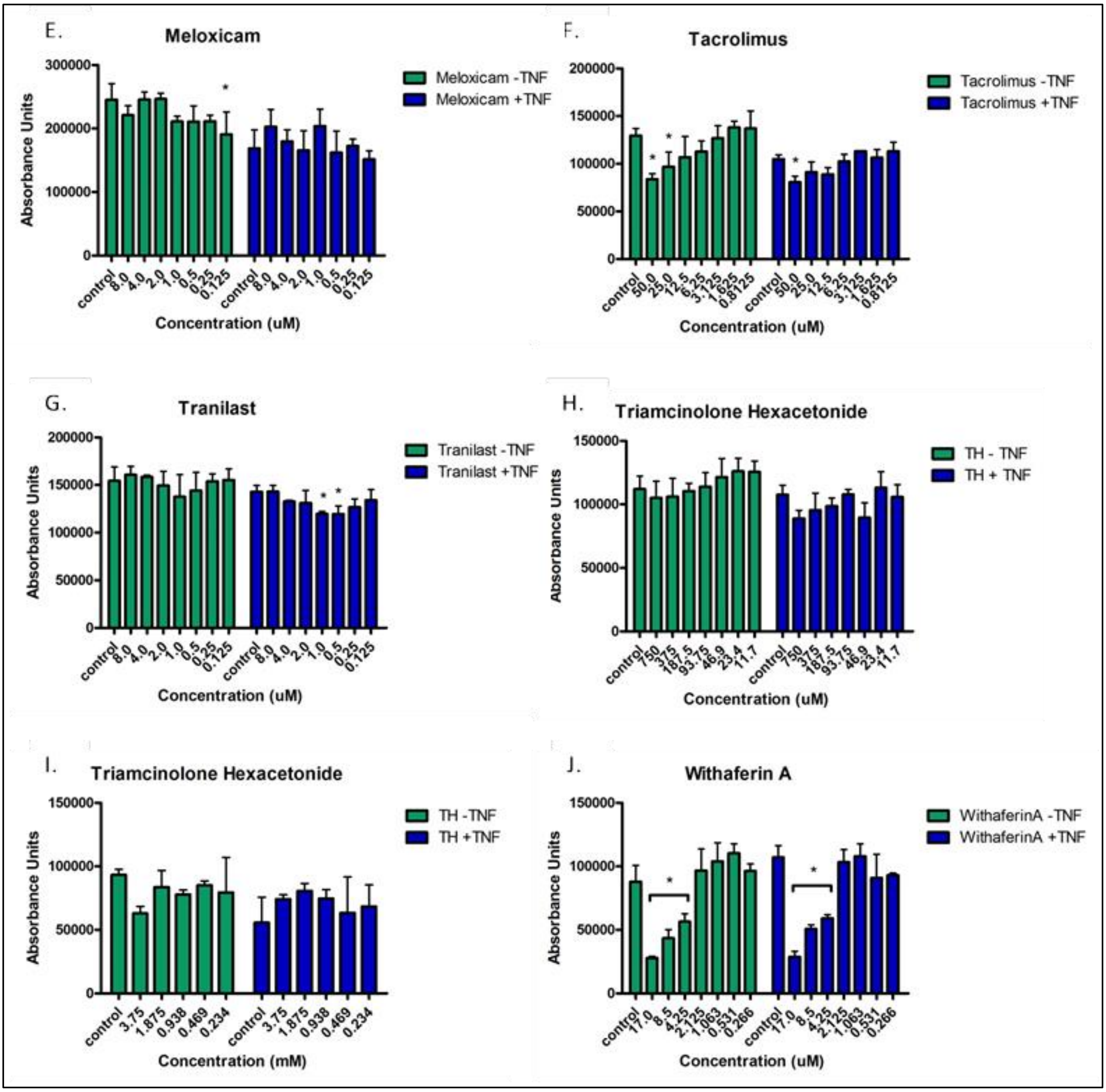

E.
Meloxicam
Absorbance Units
Concentration (uM)
Meloxicam -TNF
Meloxicam +TNF
F.
Tacrolimus
Absorbance Units
Concentration (uM)
Tacrolimus -TNF
Tacrolimus +TNF
G.
Tranilast
Absorbance Units
Concentration (uM)
Tranilast -TNF
Tranilast +TNF
H.
Triamcinolone Hexacetonide
Absorbance Units
Concentration (uM)
TH - TNF
TH + TNF
I.
Triamcinolone Hexacetonide
Absorbance Units
Concentration (mM)
TH -TNF
TH +TNF
J.
Withaferin A
Absorbance Units
Concentration (uM)
WithaferinA -TNF
WithaferinA +TNF

Figure 29. Drug toxicity testing with chondrogenic pellets for agents not tested in vivo.

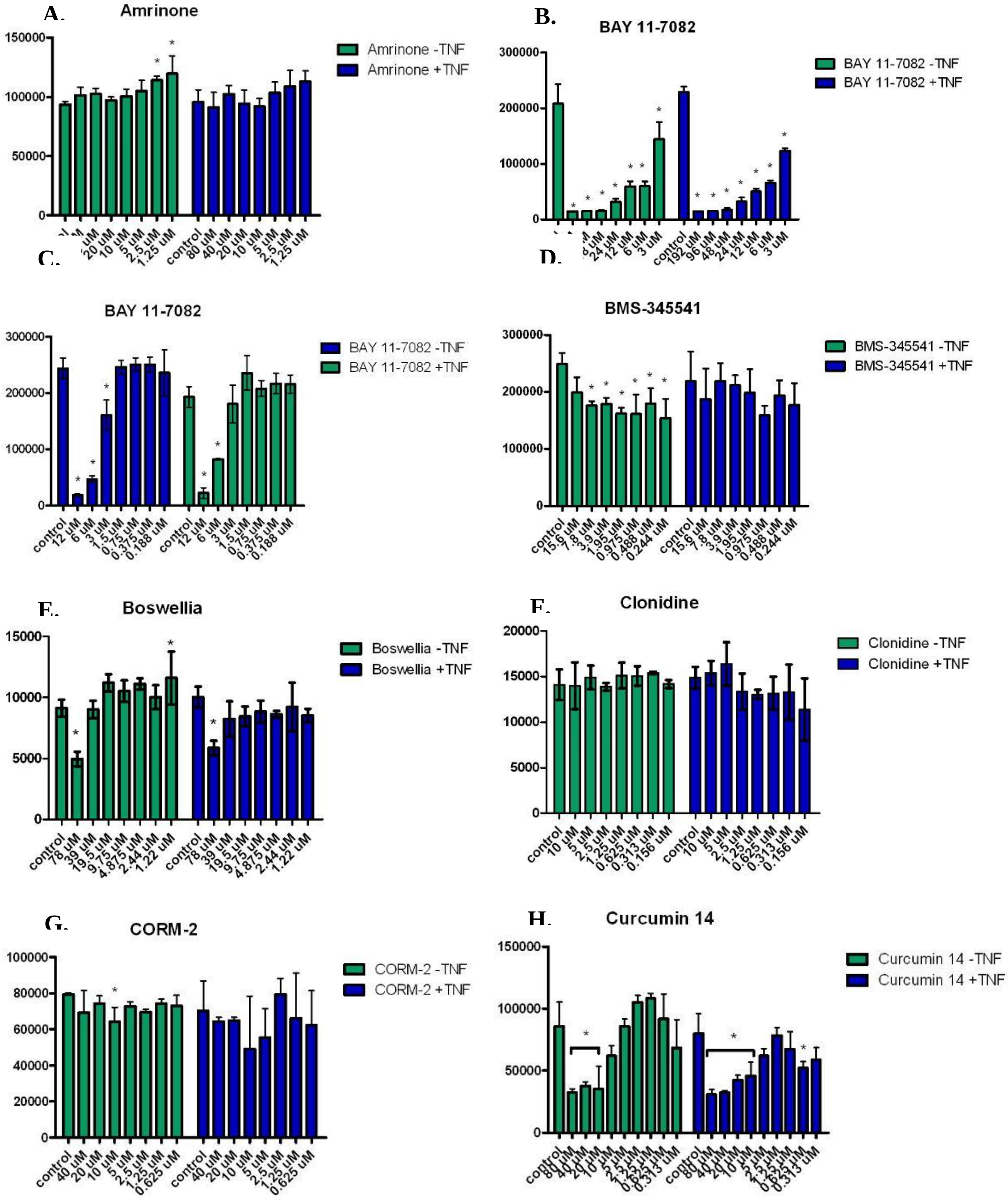

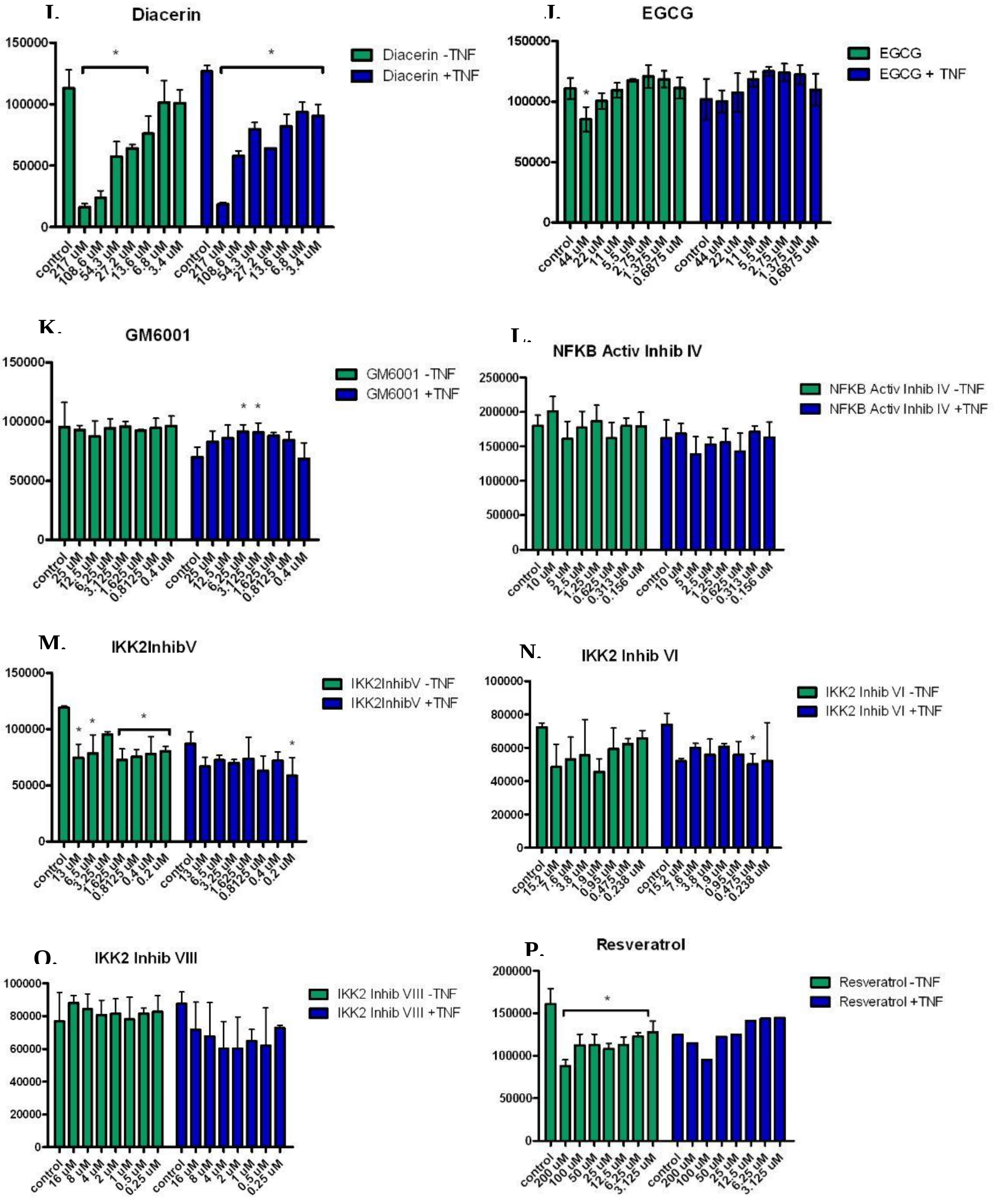
I.
Diacerin
Diacerin -TNF
Diacerin +TNF
control
217 uM
108.6 uM
54.3 uM
27.2 uM
13.6 uM
6.8 uM
3.4 uM
J.
EGCG
EGCG
EGCG + TNF
control
44 uM
22 uM
11 uM
5.5 uM
2.75 uM
1.375 uM
0.6875 uM
K.
GM6001
GM6001 -TNF
GM6001 +TNF
control
25 uM
12.5 uM
6.25 uM
3.125 uM
1.625 uM
0.8125 uM
0.4 uM
L.
NFKB Activ Inhib IV
NFKB Activ Inhib IV -TNF
NFKB Activ Inhib IV +TNF
control
10 uM
5 uM
2.5 uM
1.25 uM
0.625 uM
0.313 uM
0.156 uM
M.
IKK2InhibV
IKK2InhibV -TNF
IKK2InhibV +TNF
control
13 uM
6.5 uM
3.25 uM
1.625 uM
0.8125 uM
0.4 uM
0.2 uM
N.
IKK2 Inhib VI
IKK2 Inhib VI -TNF
IKK2 Inhib VI +TNF
control
15.2 uM
7.6 uM
3.8 uM
1.9 uM
0.95 uM
0.475 uM
0.238 uM
O.
IKK2 Inhib VIII
IKK2 Inhib VIII -TNF
IKK2 Inhib VIII +TNF
control
16 uM
8 uM
4 uM
2 uM
1 uM
0.5 uM
0.25 uM
P.
Resveratrol
Resveratrol -TNF
Resveratrol +TNF
control
200 uM
100 uM
50 uM
25 uM
12.5 uM
6.25 uM
3.125 uM

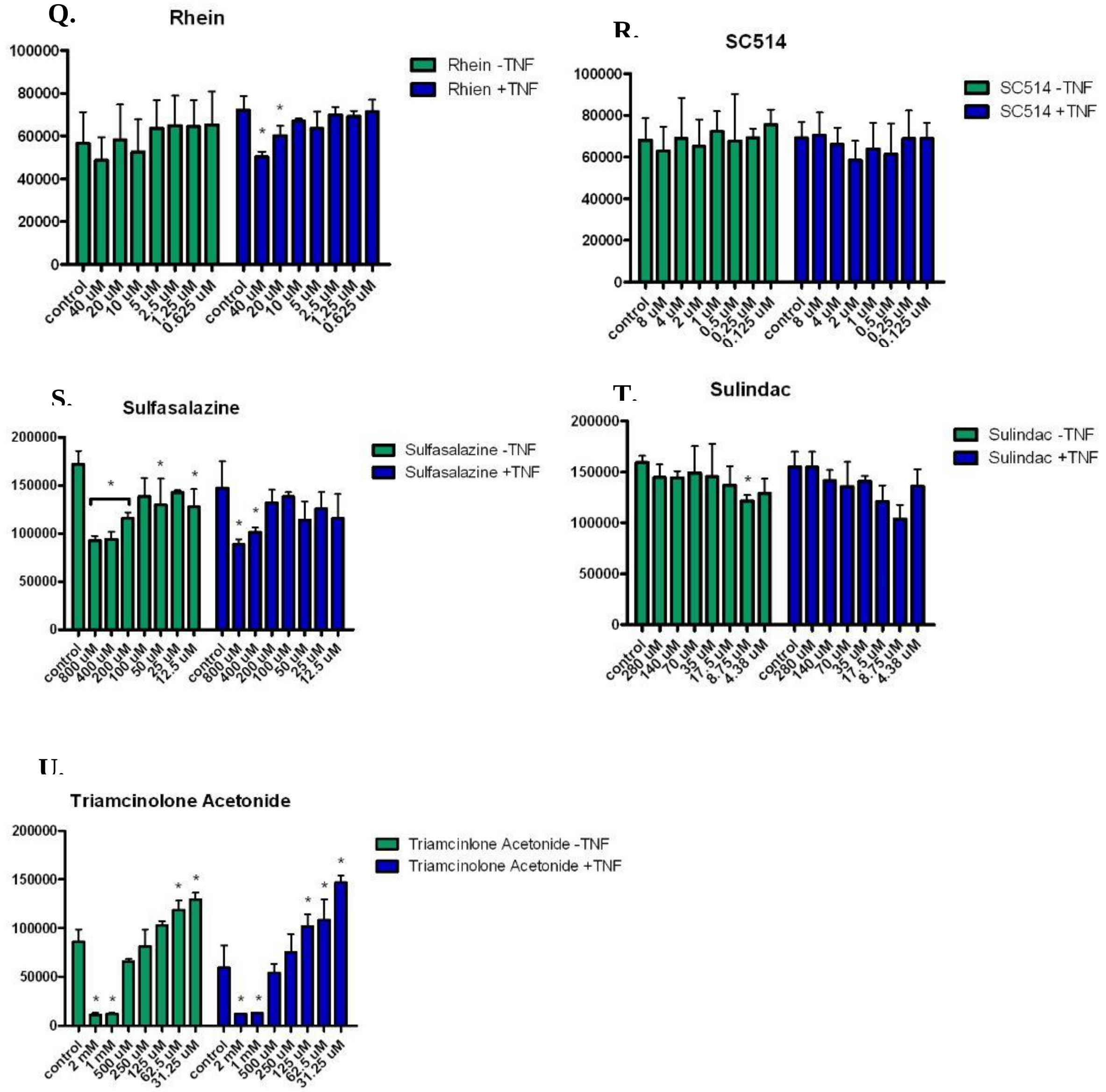
Q.
Rhein
Rhein -TNF
Rhien +TNF
100000
80000
60000
40000
20000
0
control
40 uM
20 uM
10 uM
5 uM
2.5 uM
1.25 uM
0.625 uM
R.
SC514
SC514 -TNF
SC514 +TNF
8 uM
4 uM
2 uM
1 uM
0.5 uM
0.25 uM
0.125 uM
S.
Sulfasalazine
Sulfasalazine -TNF
Sulfasalazine +TNF
200000
150000
100000
50000
800 uM
400 uM
200 uM
100 uM
50 uM
25 uM
12.5 uM
T.
Sulindac
Sulindac -TNF
Sulindac +TNF
280 uM
140 uM
70 uM
35 uM
17.5 uM
8.75 uM
4.38 uM
U.
Triamcinolone Acetonide
Triamcinlone Acetonide -TNF
Triamcinolone Acetonide +TNF
2 mM
1 mM
500 uM
250 uM
125 uM
62.5 uM
31.25 uM

In Vitro Hela Cell Drug Toxicity Testing

Potential drug toxicity was measured using a CTG assay and HeLa cells (Promega). Drugs not tested in this assay were tested in either of the in vitro toxicity assays already presented (synovial or chondrocyte toxicity assays).

Table 3- Drug toxicity measured using a CTG assay and HeLa cells.

Agent	Range Tested (µM)	Toxic Concentrations ($p<0.05$) (µM)	Non-toxic concentrations (µM)	Effective against NF κβ ($p<0.05$) (µM)
BAY-11-7082	6 – 96	-	6 – 96	12 – 96
BMS-345541	0.24 – 15.6	-	0.24 – 15.6	1-15.6
Boswellic Acid	42.5 – 680	85 – 680	42.5	Only at toxic concentrations
Curcumin	2.1 – 54	54	0 – 33.75	33.75 – 54
Diacerein	6.9 – 110	27.5 -110 without TNF<br>50 -110 with TNF	0 – 13.75 (TNF)<br>0 – 22.75 (+TNF)	110
IKK-2 Inhibitor IV	0.28 – 7.8	7.66	0 – 3.83	Not consistently effective (tested 3 times)
IKK-2 Inhibitor V (IMD-0354)	0.101 - 26	0.101 - 26 (without TNF 0.101 µM not toxic)	None	Only at toxic concentrations
IKK-2 Inhibitor VI	0.028 – 3.83	1.9 – 3.83 More toxicity with TNF	0 – 0.9	0.23 – 15.3
Rhein	2.5– 40	40	2.5 – 20	Only at toxic concentrations
Sulindac	8.75 – 70	-	No toxicity (+/- TNF) 8.75 – 70	70
Sulfasalazine	50 – 800	-	No toxicity (+/- TNF) 50 – 800	400 – 800
Tacrolimus	3.1 – 49.6	-	0 – 49.6	25 -49.6
Withaferin A	0.25 – 17	-	0 –17	2 – 17

Toxicity and Efficacy: Comparison of Test Results

Table 4. Comparison of screening results for various drugs.

**Agent**	**Range Tested (µM)**	**Non-toxic range (µM)** (Synovial)	**Non-toxic range (µM)** (Chondrocyte)	**Effective range against NFkB (µM)**	**Effective range against MMP-13 (µM)**	**Effective range against Osteoclasts (µM)**	**Non-toxic & predicted range of efficacy (uM)**
Alendronate (bone control)	0.625 - 40	1.25 – 20 (40 toxic)	0.625 - 40	Not tested	Not effective in range tested	1.25 and 20	1.25 -20^{+} **Bone control**
Amrinone	0.625 - 80	1.25 - 40	1.25 - 80	None; tested to 50 uM	80	Not effective in range tested	80 + for MMP only; don't use
Ascomycin	0.01 - 40	0.625 -40	0.625 - 40	1.25 - 40	Not effective in range tested	0.01^{+} (need to test lower)	1.25 – 40 **NFkB; OCs**
BAY-11-7082	0.75 – 12	3-6 (12^{+} toxic)	0.18 – 1.5 (3^{+} toxic)	12-96	0.75^{+}	3^{+}	None, don't use
BMS-345541	0.24 – 15.6	0.24 – 7.8 (15.6 toxic)	0.24-15.6	1 – 15.6	0.244^{+}	0.244	0.24 – 7.8
Boswellic Acid	0.61 – 680	2.4 – 156 (test higher)	0.6 – 78 (test higher)	680	39 $^{+}$	9.75^{+}	40-680 **NFkB; MMP-13; OCs**
Clonidine	0.156 - 100	1.56 - 100	0.156 - 100	Not effective in the range tested	Not effective in the range tested	10 and higher	**Pain control only**
CORM-2	0.625 - 40	2.5 - 40	0.625 - 40	Not tested – not expected to inhibit	10 – 40	20-40	20 – 40 (possibly higher) **MMP-13; OCs**

**Agent**	**Range Tested (µM)**	**Non-toxic range (µM)** (Synovial)	**Non-toxic range (µM)** (Chondrocyte)	**Effective range against NFkB (µM)**	**Effective range against MMP-13 (µM)**	**Effective range against Osteoclasts (µM)**	**Non-toxic & predicted range of efficacy (uM)**
Curcumin	0.84 – 108	1.7 – 54 (108+ toxic, but some variability)	0.84 – 27 (54+ toxic)	13.5 and higher	3.4 and higher	6.75+	13.5 -54<br><br>**NFkB; MMP-13; OCs**
Curcumin 14	1.25 - 80	0.313 – 5.0 (10+ toxic but variable)	0.3 – 10 uM (10 with minimal toxicity)	2.5 and higher	0.625 and higher	Not effective in range tested	2.5 – 10<br><br>**NFkB; MMP-13**
Diacerein	1.5 – 217	1.5 – 54.3 (108+ toxic)	All toxic (3.4 – 217 toxic)	110 uM (higher not tested)	6.8 - 217	13.6 - 110	None; don' t use
EGCG	0.69 - 44	0.69 -44	0.69 -44	5.5 - 88	5.5 and higher	None?	5.5 -44 (possibly higher)<br>**NFkB; MMP-13**
Fluocinolone	0.156 – 250	1.56 - 100	1.56 - 250	3.1 and higher (test lower)	1.56 and higher (test lower)	9.77 nM and higher (test lower)	3.1 – 250 (test lower)<br>**NFkB; MMP-13; OCs**
GM6001	0.33 - 25	0.33 - 25	0.4 - 25	Not tested	25	Not effective	25+ (test higher)
IKK-2 Inhibitor IV	0.28 – 7.8	0 – 20	Not tested	1.8 -7.7	Not tested	Not tested	Due to limited availability dropped
IKK-2 Inhibitor V (IMD-0354)	0.025 – 26	0.1 – 0.2 (test lower)	0.4 – 13 (test higher)	1.625 - 26	0.6125 and higher	0.813 and higher	1.625-26<br>**NFkB; MMP-13; OCs**

Agent	Range Tested (µM)	**Non-toxic range (µM)** (Synovial)	**Non-toxic range (µM)** (Chondrocyte)	**Effective range against NFkB (µM)**	**Effective range against MMP-13 (µM)**	**Effective range against Osteoclasts (µM)**	**Non-toxic & predicted range of efficacy (uM)**
IKK-2 Inhibitor VI	0.028 –15.2	0.028 – 0.25 not toxic	0.23 – 15.6	0.95 and higher	0.24 and higher	0.5 and higher (test lower)	0.95-4.0 **NFkB; MMP-13; OCs**
IKK inhibitor VIII	0.25 - 16	0.3 – 2.0 (4.0+ toxic)	0.25 - 16	Not tested.	4 - 16	2 and higher	2-4 (risk toxicity to synovium)
Keterolac	0.625 - 80	0.625 - 40	3.17 - 200	2.5 only; not 5-40	12.5 and higher	Not effective in range tested	12.5 and higher **MMP-13 only**
Meloxicam	0.125 - 32	0.25 – 16 (32 toxic)	0.125 -8 (need to test higher)	16 and higher	Not effective in range tested	Not effective in range tested	16-32 uM
Pimicrolimus	0.625 - 40	0.625 - 40	0.625 - 40	2.5 – 20 (not tested lower)	Not effective in range tested	0.01 - 100	2.5 – 40 possibly lower **NFkB; OCs**
Resveratrol	3.125 - 200	3.125 – 100 (200+ toxic)	3.125 - 200	Not effective in range tested	3.125 and higher	6.25 and higher	6.25 – 200 **MMP-13; OCs**
Rhein	0.625– 40	0.625 -40	0.625-40	40	40	Not effective in range tested	40 (test higher) **NFkB; MMP**
SC514	0.125 - 32	0.125 – 16 (32 toxic)	0.125 – 8 (test higher)	None (tested only to 8 uM)	Not effective (only tested to 8 uM)	Not effective tested (only tested to 4 uM)	Don't use
Sulindac	4.35 – 280	4.4 - 280	4.35 – 280	8.8 - 140 (not tested at higher)	8.75 and higher	100	8.8 – 280 **NFkB; MMP-13; OCs**

Agent	Range Tested (µM)	Non-toxic range (µM) (Synovial)	Non-toxic range (µM) (Chondrocyte)	Effective range against NFkB (µM)	Effective range against MMP-13 (µM)	Effective range against Osteoclasts (µM)	Non-toxic & predicted range of efficacy (uM)
Sulfasalazine	12.5 – 1600	12.5 – 400 (400+ toxic)	12.5 – 800	50 - 800	50 and higher	150 - 1600	50 -400 **NFkB; MMP-13; OCs**
Tacrolimus	0.8 – 100	1.5 - 100	0.8 - 50	25 and higher	50	0.011 and higher	25 -50; possibly higher **NFkB; MMP-13; OCs**
Tranilast	0.125 - 8	0.125 - 8	0.125 -8 (some minimal toxicity at mid values)	None; tested only to 8 uM	1 and higher	0.5 (but not 0.25)	1 – 8 **MMP-13; OCs**
Triamcinolone acetonide	0.7 – 1000	0.7 – 250 (500+ toxic)	32.25 - 500	None	31.25	1	31.25 -500 **MMP-13; OCs**
Triamcinolone hexacetonide	3.125 - 1000	3.125 - 400	11.7 uM – 3.75 mM	62.5+	0.06 and higher	9.5 and higher	62.5 uM – 3.75 mM **NFkB; MMP-13; OCs**
Verapamil	1.25 - 80	1.25 – 40 (80+ toxic)	1.25 -40 (80 barely toxic)	Only 20	20 – 80, but significant variability	Not effective concentration (to 40)	20 – 40 **NFkB and MMP-13**
Withaferin A	0.25 – 17	Toxic (or pro-apoptotic) down to 0.067	0.27 – 2.1	0.5– 17	0.25 and higher	0.5 and higher	synovial toxicity but effective for all targets 0.27 – 2.1 (nontoxic to cartilage)

In Vivo Testing of Osteoarthritic Animals

<u>MIA Model Functional Assessment</u> *<u>(see Appendix for individual animal data)</u>*

MIA Trial 1: Weight Bearing (WB)

Saline, morphine, clonidine, and fluocinolone were tested in the MIA model as shown below.

1) Effect of Saline alone

Intra-articular injection of MIA resulted in the development of significant and prolonged osteoarthritis(OA)-related pain as demonstrated by decreases in pre-treatment hind limb weight bearing in vehicle-treated animals compared to pre-MIA injection on all days tested. Administration of vehicle alone had no effect on WB score at any time point on any testing day when post-treatment WB score of vehicle-treated animals were compared to the corresponding pre-treatment WB score demonstrating the stability of the vehicle group (**Figure 30**). Shown is the mean ± SEM (n = 10) WB score for the experimental group indicated.

Figure 30 – Effect of vehicle on weight bearing on Days 7, 14, 21, and 28

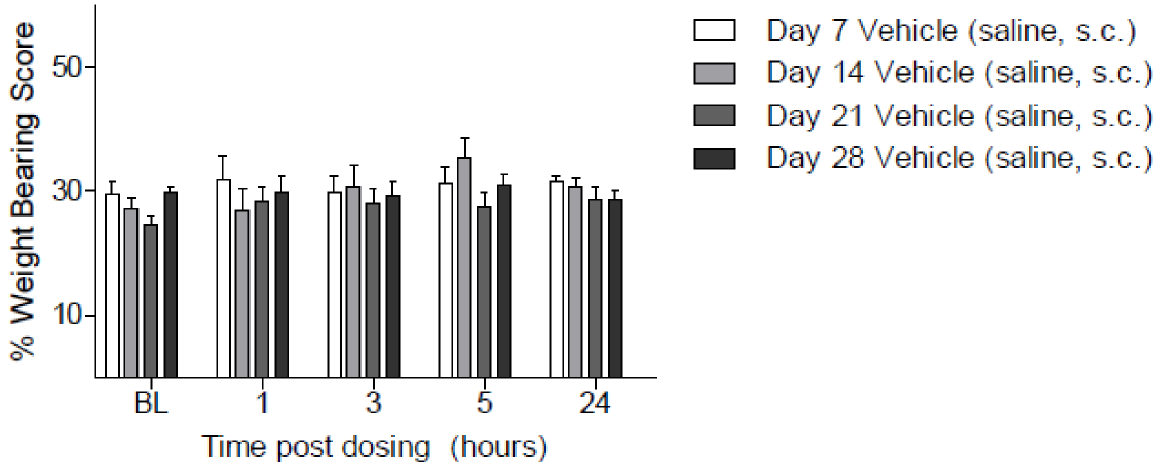

2) Effect of Morphine

Treatment with morphine (6 mg/kg, s.c.) significantly increased the WB score 1, 3, and 5 hours after administration on Day 7 (**Figure 31**), 1 hour after administration on Day 14 (**Figure 32**), and 1 and 3 hours after administration on Days 21 and 28 (**Figures 33 & 34**, respectively) compared to the vehicle-treated group on the respective testing days. Administration of morphine (6 mg/kg, 5 ml/kg, s.c.) on Days 7, 14, 21, and 28 had no effect on pre-dosing WB score on any day tested compared to Day 7 pre-treatment measurements, however there was a significant difference in WB score on Day 21 when compared to vehicle-treated rats not shown. Below are the mean ± SEM (n = 10) WB score for the experimental groups indicated (*/**/***: $p < 0.05/0.01/0.001$, unpaired t-test, versus vehicle).

Figure 31 – Effect of morphine (s.c.) weight bearing on Day 7

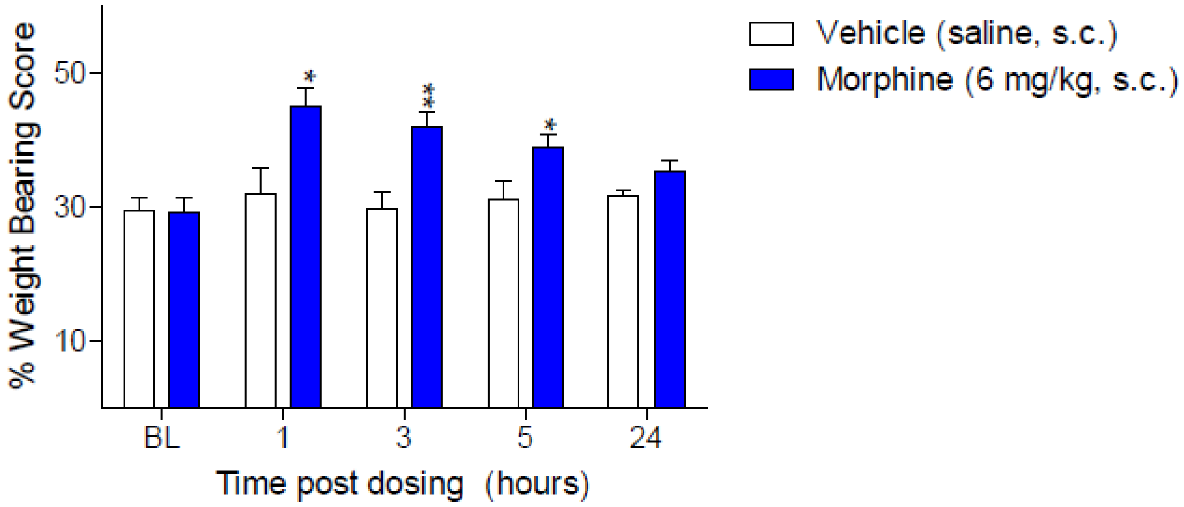

Figure 32 - Effect of morphine (s.c.) on weight bearing on Day 14

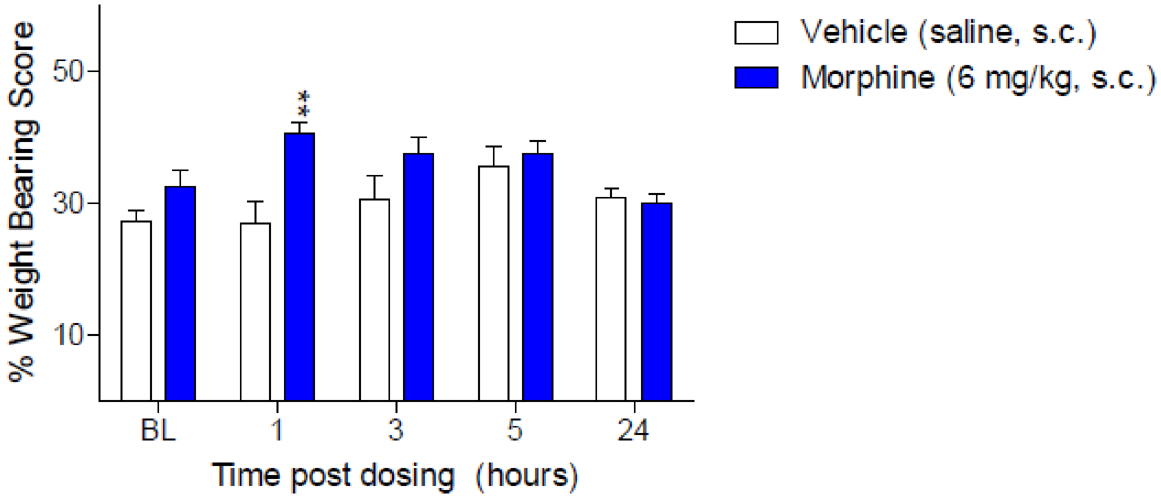

Figure 33 - Effect of morphine (s.c.) on weight bearing on Day 21

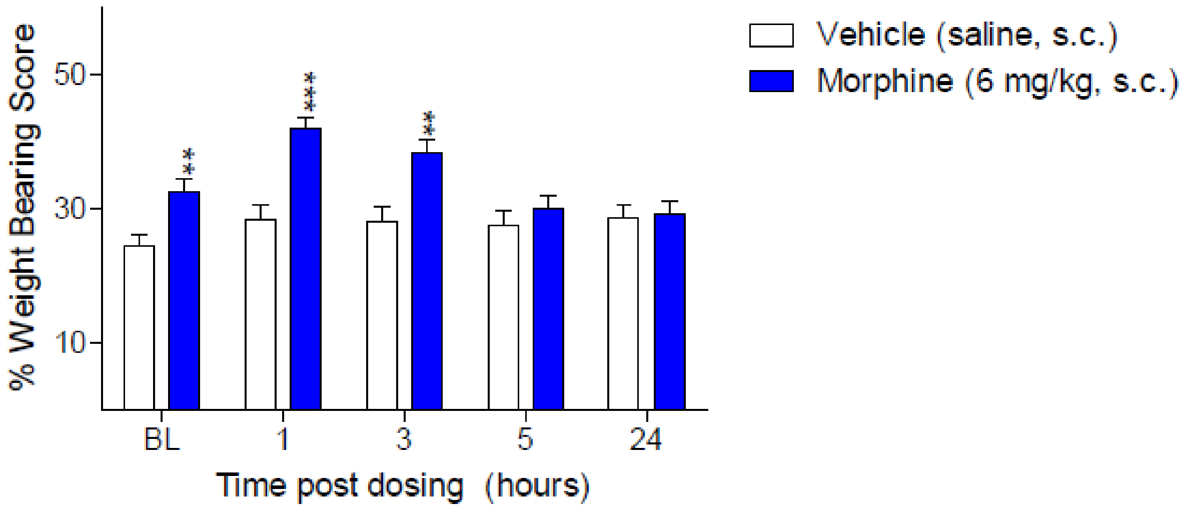

Figure 34 - Effect of morphine (s.c.) on weight bearing on Day 28

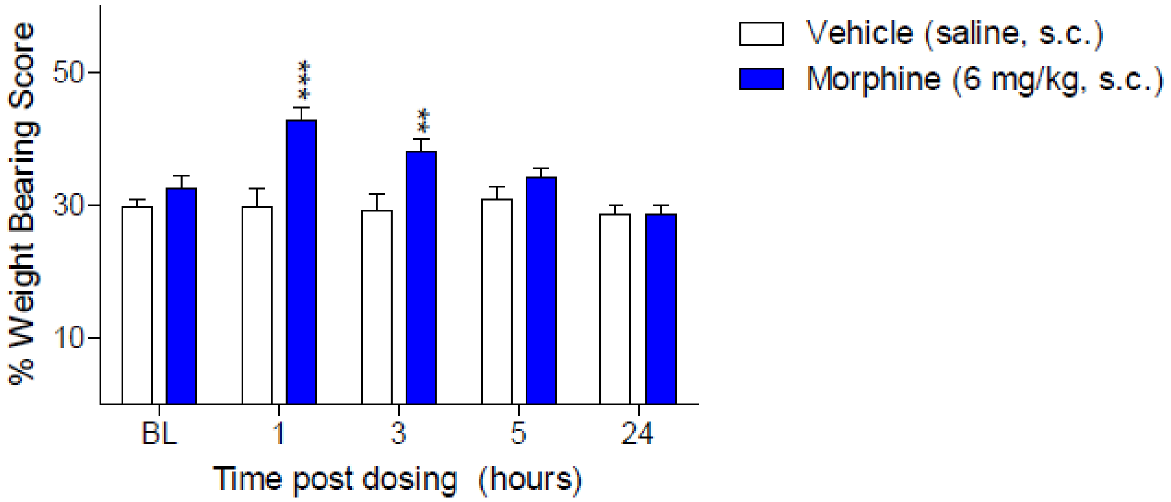

3) Effect of Clonidine

Daily administration (starting on Day 7) of clonidine (100 g/kg, 5 ml/kg, s.c.) had no effect on pre-treatment WB score on any day tested compared to Day 7 pre-treatment measurements or vehicle-treated rats (not shown). Clonidine treatment (100 g/kg, s.c.) increased the WB score 1, 5, and 24 hours after administration on Day 7 (**Figure 35**), 1 hour after administration on Days 14 and 21 (**Figures 36 & 37**, respectively), and 1 and 3 hours after administration on Day 28 (**Figure 38**) compared to the vehicle-treated group WB score on the respective testing days. Shown is the mean ± SEM (n = 10) WB score for the experimental groups indicated (*/**/***: $p < 0.05/0.01/0.001$, unpaired t-test, versus vehicle).

Figure 35 - Effect of clonidine (s.c.) on weight bearing on Day 7

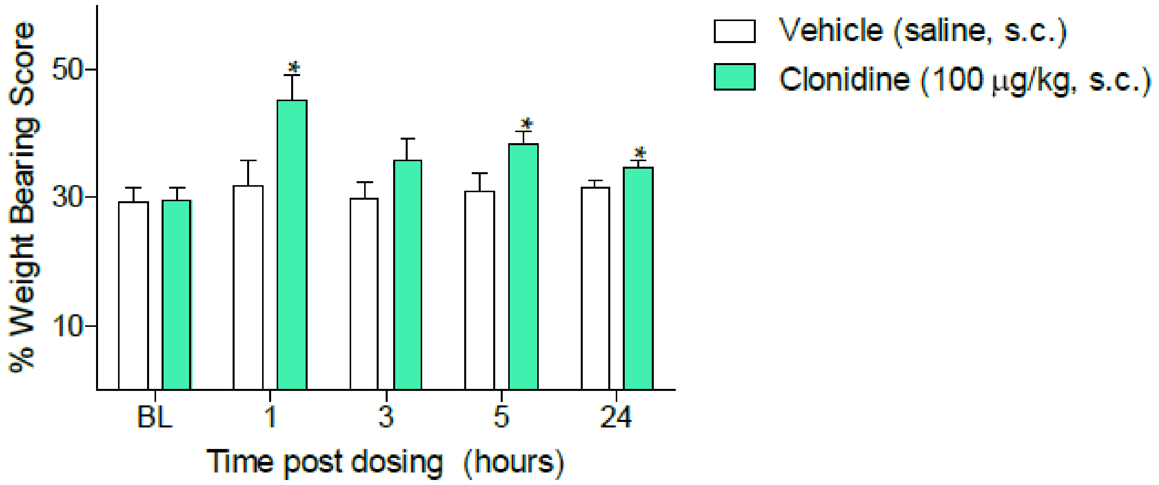

Figure 36 - Effect of clonidine (s.c.) on weight bearing on Day 14

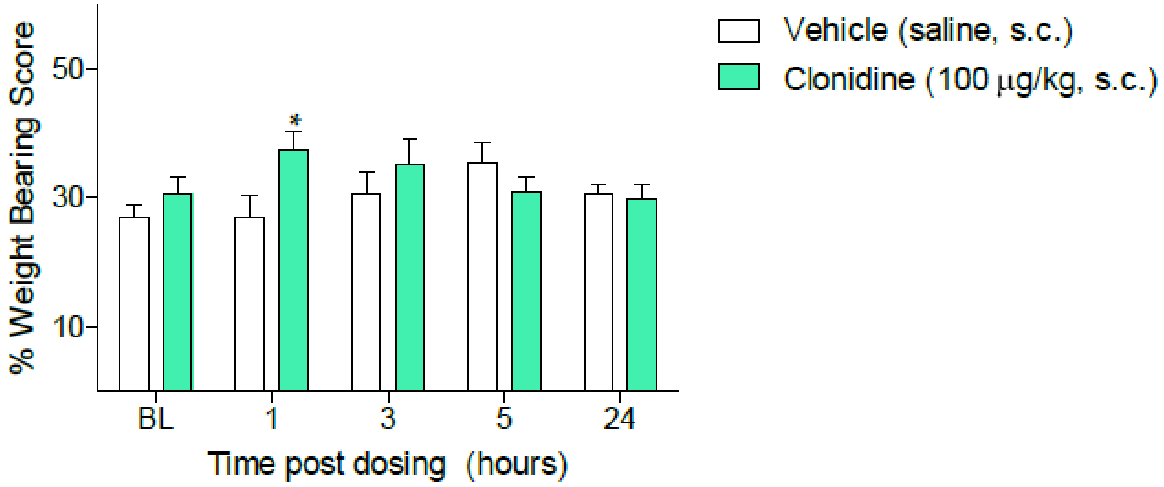

Figure 37- Effect of clonidine (s.c.) on weight bearing on Day 21

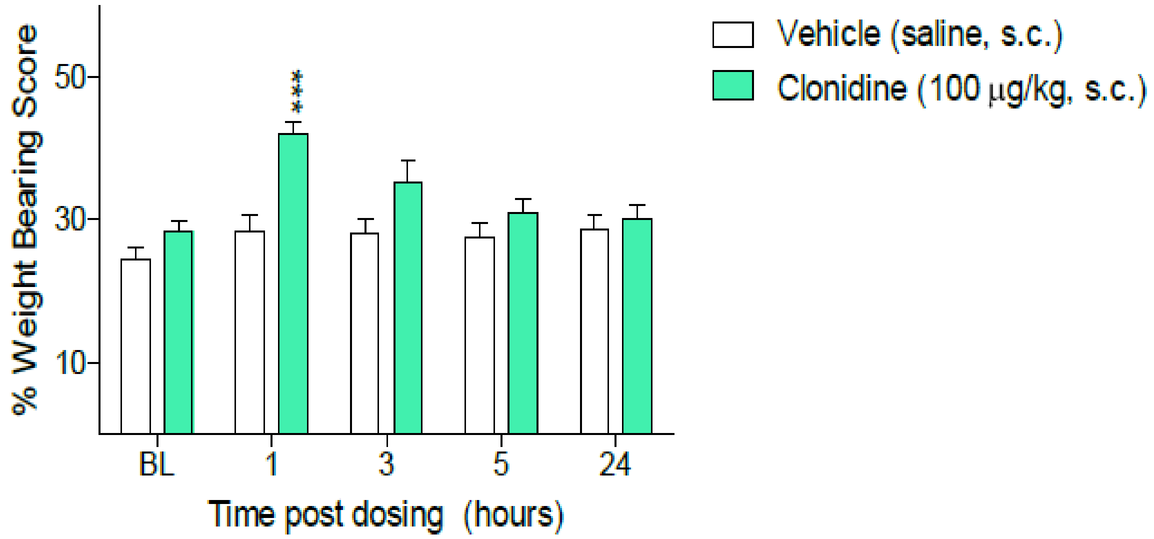

Figure 38 - Effect of clonidine (s.c.) on weight bearing on Day 28

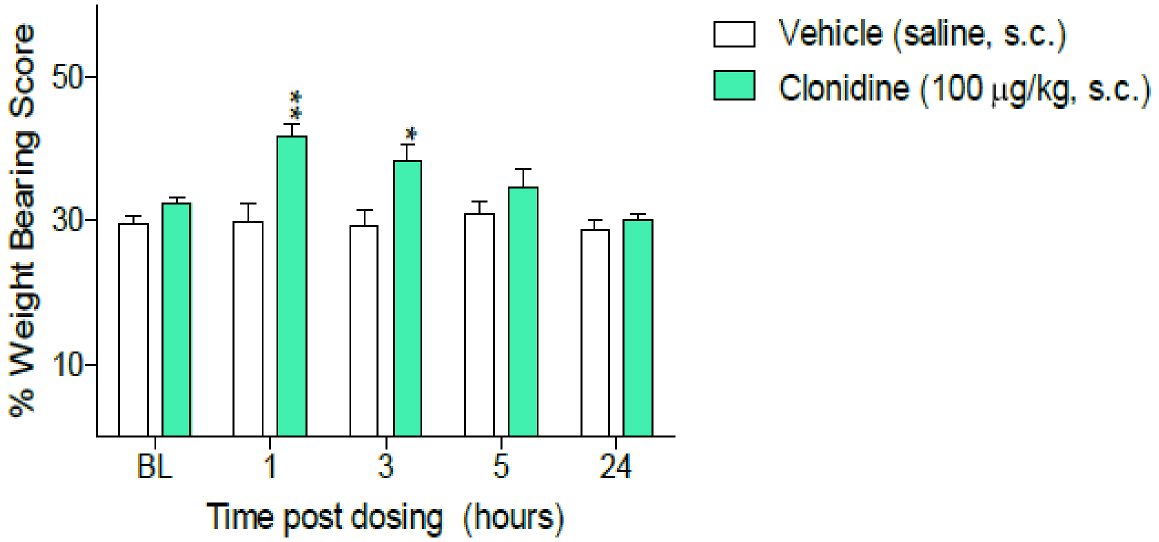

4) Effect of Fluocinolone

A significant difference in WB score prior to daily dosing was observed on Day 21 compared to vehicle-treated rats (**Figure 39**) following daily administration (starting on Day 7) of fluocinolone (2 g/kg, 5 ml/kg, s.c.). This was similar to that observed with morphine. Fluocinolone treatment also significantly increased WB score 1 hour after administration on Day 21 (**Figure 40**) but had no effect on WB score at any time point tested on Days 7, 14, or 28 compared to the vehicle-treated group WB score on the respective testing days. (*: $p < 0.05$, unpaired t-test, versus vehicle).

Figure 39 –Pre-dose effect of daily fluocinolone administration (s.c.) on weight bearing

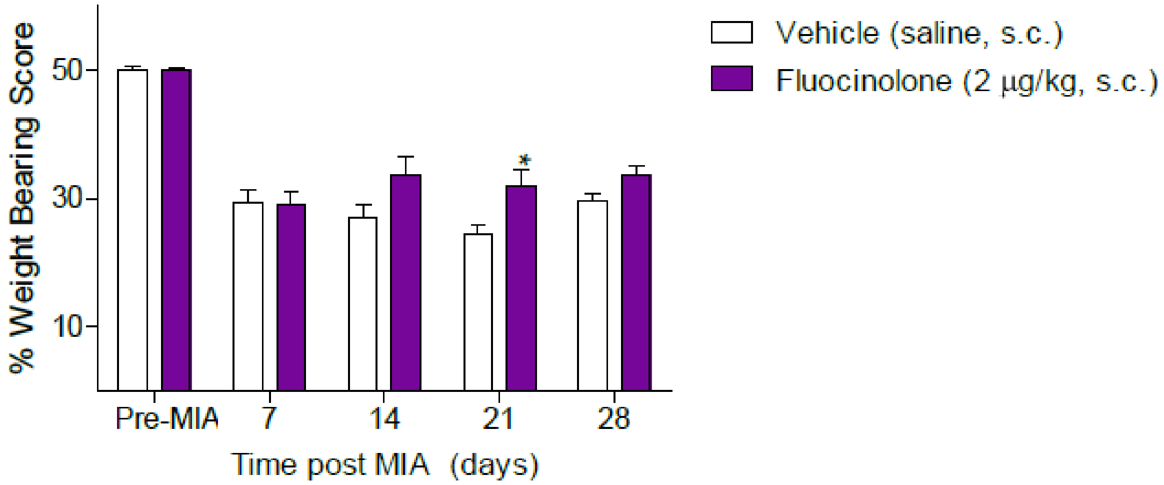

Figure 40 - Effect of fluocinolone (s.c.) on weight bearing on Day 21

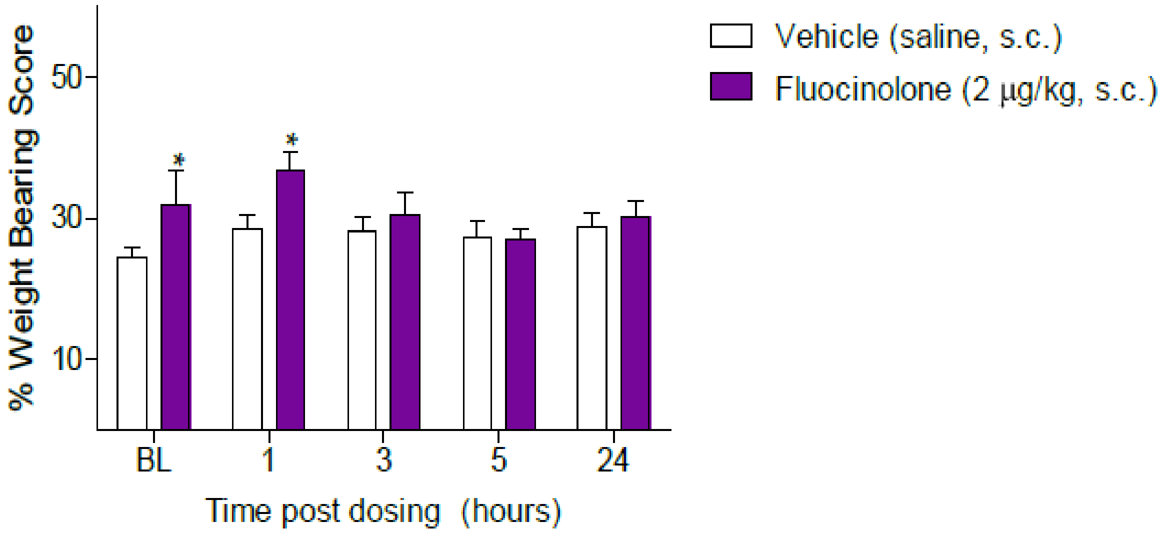

MIA Trial 2: Digital Randall-Selitto (dRS) Test

Because the ability to discern differences using weight bearing was limited in the MIA model, the digital Randall-Selitto test (dRS) was verified for efficacy (data not shown). A number of drugs were tested including Clonidine, Tacrolimus, Curcumin, and Fluocinolone. Shown is the mean ± SEM (n = 10) joint compression thresholds for the experimental groups indicated (+: $p < 0.05$, paired t-test versus vehicle; */***: $p < 0.05/0.001$, Dunnett's *post hoc* test for comparisons versus Day 7). Note that clonidine was delivered weekly, whereas the other compounds were delivered daily. Individual animal data is shown in the Appendix.

Effect of Vehicle

Differences between ipsilateral (injured) and contralateral (normal) joint compression thresholds within the vehicle group were statistically significant at all time points tested on each testing day following MIA injection. A significant increase in the ipsilateral joint compression threshold was observed 1 hour after administration of vehicle and a significant decrease in the contralateral joint compression threshold was observed 5 hours after administration of vehicle compared to baseline values on Day 21. Administration of vehicle had no effect on joint compression thresholds at any other time point when post-treatment joint compression thresholds of vehicle-treated animals were compared to the corresponding pre-treatment values (data not shown).

Effect of Clonidine

Effects on pre-treatment joint compression thresholds were observed following administration of clonidine (0.1 mg/kg, s.c.) on Days 7, 14, 21 and 28 (**Figure 41**). A significant decrease in pre-treatment joint compression thresholds was observed on Day 14 compared to Day 7 values but not to vehicle-treated animals. A significant increase in pre-treatment joint compression thresholds was observed on Day 21 compared to Day 7 values but not to vehicle-treated animals. A significant decrease in pre-treatment joint compression thresholds was observed on Day 28 compared to vehicle treated animals but not to Day 7 values.

Figure 41 – Pre-dose effect of weekly clonidine (s.c.) administration on MIA-induced mechanical hyperalgesia

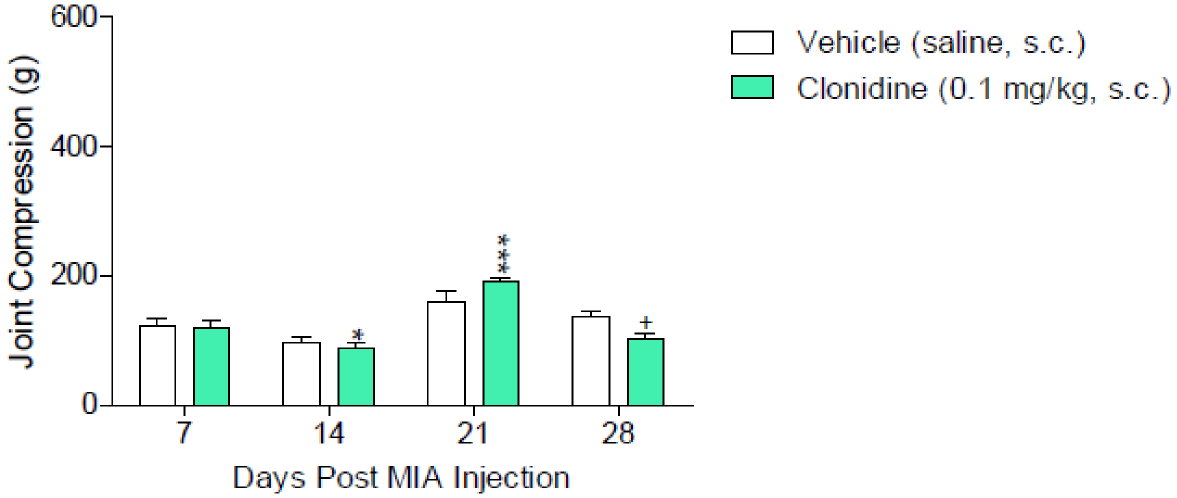

Subcutaneous administration of clonidine (0.1 mg/kg) once a week immediately prior to testing increased joint compression thresholds 1, 3 and 5 hours after administration on Days 7, 14 and 28 (**Figures 42, 43 & 45**, respectively) and 3 hours after administration on Day 21 (**Figure 44**) compared to vehicle treated animals.

Figure 42 - Effect of clonidine (s.c.) on MIA-induced mechanical hyperalgesia on Day 7

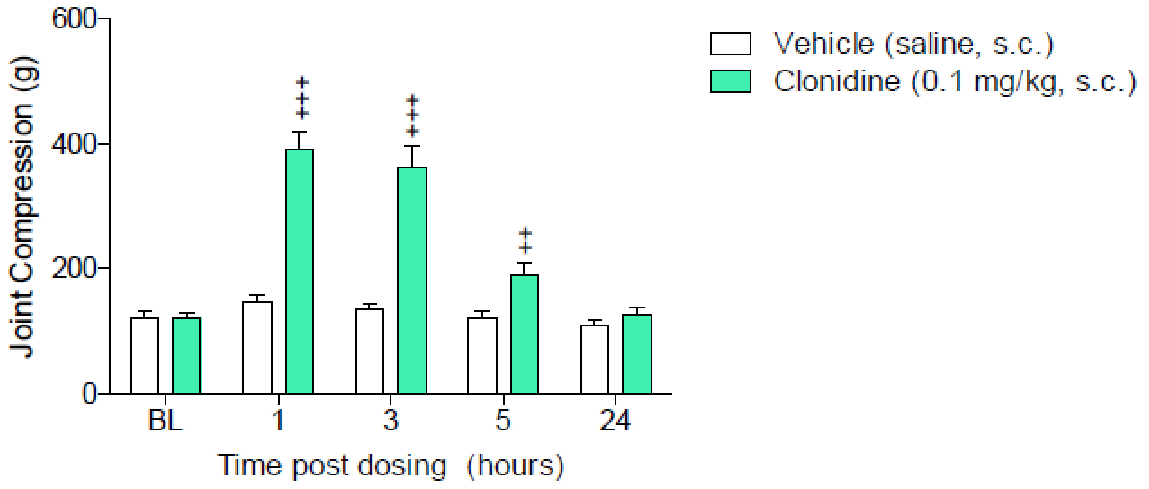

Figure 43 - Effect of clonidine (s.c.) on MIA-induced mechanical hyperalgesia on Day 14

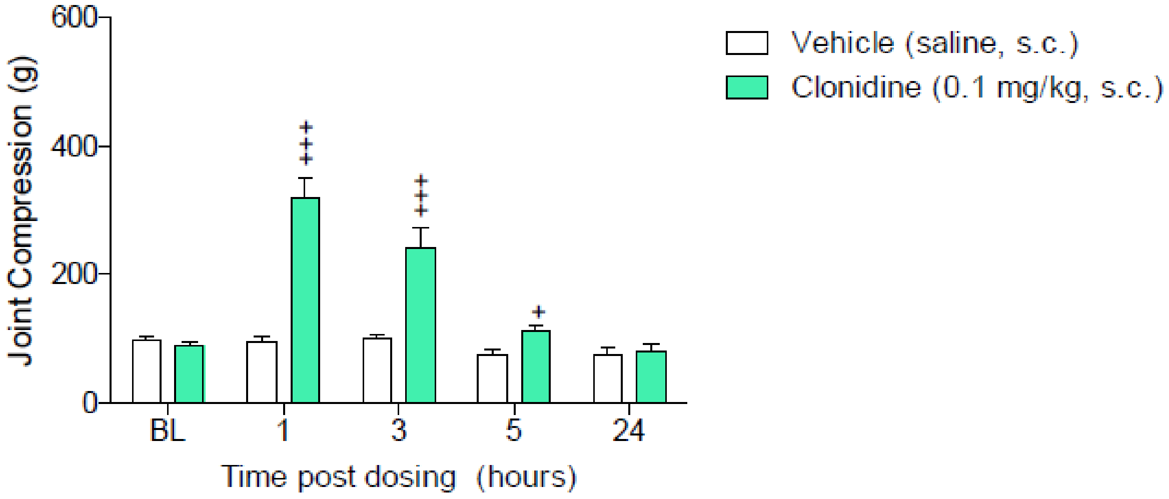

Figure 44 - Effect of clonidine (s.c.) on MIA-induced mechanical hyperalgesia on Day 21

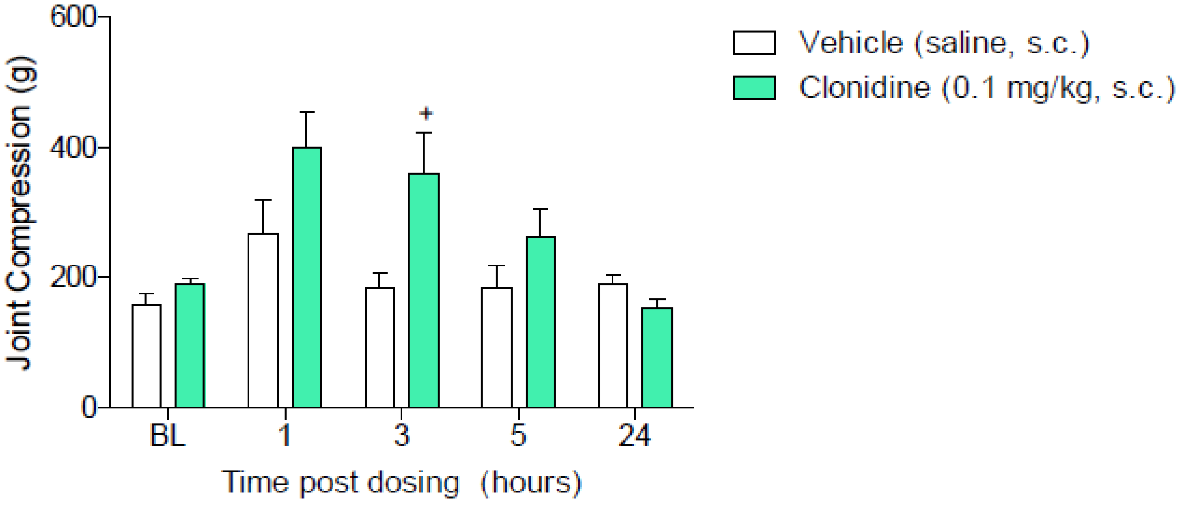

Figure 45 - Effect of clonidine (s.c.) on MIA-induced mechanical hyperalgesia on Day 28

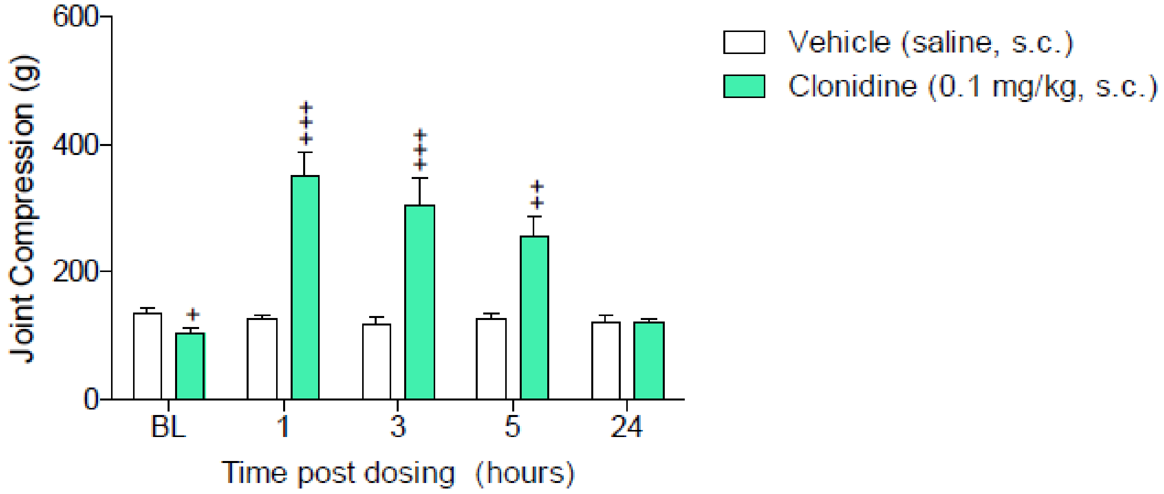

Effect of Tacrolimus

Daily administration (starting on Day 7) of Tacrolimus (0.3 mg/kg, i.p.) significantly increased pre-treatment joint compression thresholds on Day 21 following MIA injection compared to Day 7 values but not to vehicle-treated animals (**Figure 46**, below). Daily administration of Tacrolimus at the higher dose (0.6 mg/kg, i.p.) had no effect on pre-dosing joint compression thresholds at any time point tested compared to Day 7 values or to vehicle treated animals (not shown).

Figure 46 - Effect of daily Tacrolimus administration on pre-dosing MIA-induced mechanical hyperalgesia

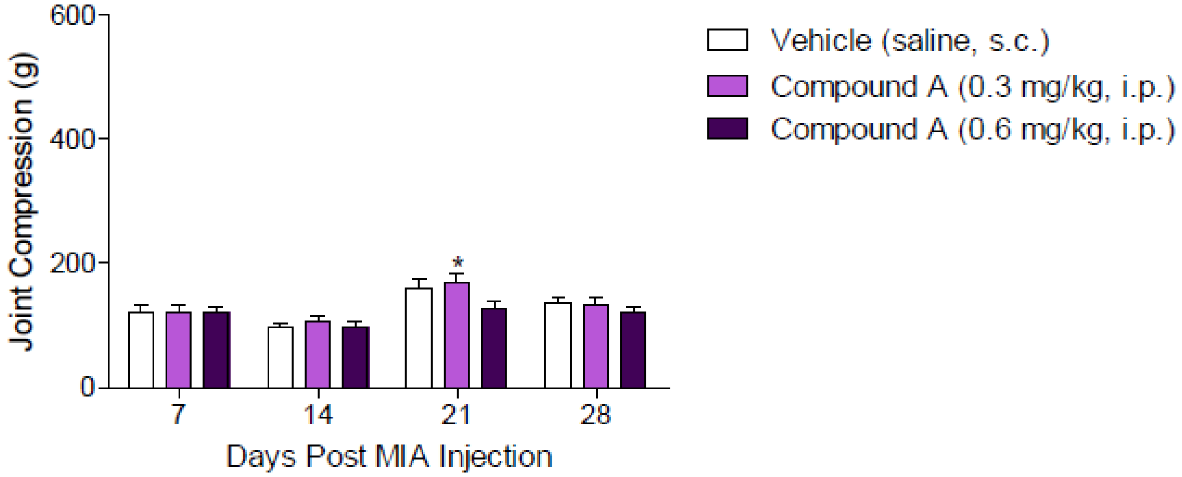

Administration of the higher dose Tacrolimus (0.6 mg/kg, i.p.) significantly increased joint compression thresholds 1 hour after administration on Day 21 (**Figure 47**) compared to pre-treatment values but not to vehicle treated animals. No effect of 0.6 mg/kg Tacrolimus was observed on Day 7, 14 or 28 (not shown) compared to pre-treatment values or to vehicle treated animals. Administration of the lower dose of Tacrolimus (0.3 mg/kg, i.p.) had no effect on joint compression thresholds at any time point tested compared to pre-treatment values or to vehicle treated animals.

Figure 47 - Effect of Tacrolimus on MIA-induced mechanical hyperalgesia on Day 21

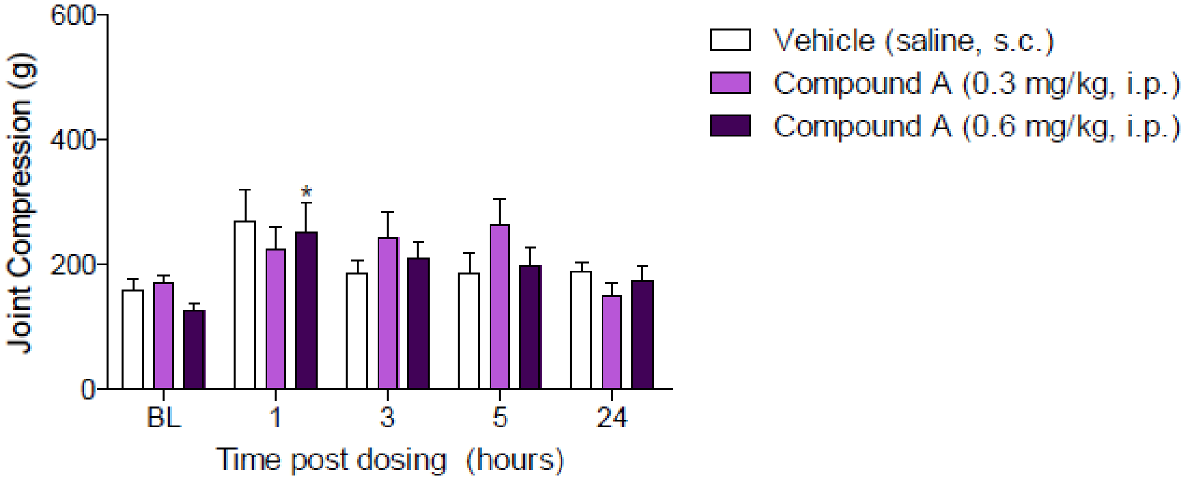

Effect of Curcumin

Oral daily administration of Curcumin significantly increased joint compression thresholds 1 hour after treatment on Day 7 compared to pre-treatment values (**Figure 48**). A significant increase in pre-dosing joint compression thresholds was also observed after daily administration of Curcumin on Day 21 when compared to Day 7 pre-treatment values (data not shown).

Figure 48 – Effect of Curcumin on MIA-induced mechanical hyperalgesia on Day 7

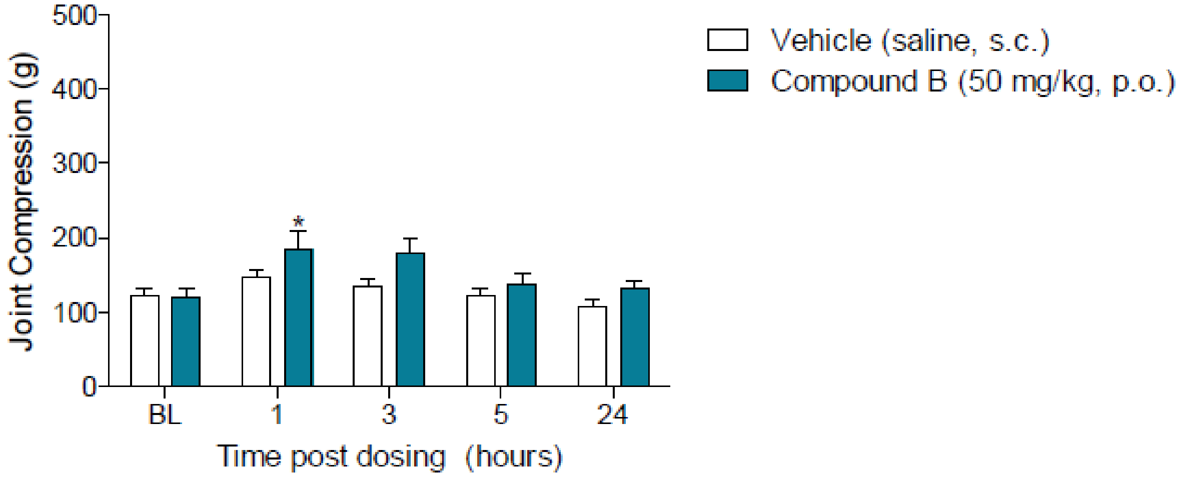

Effect of Fluocinolone

Subcutaneous daily administration of Fluocinolone (0.01 mg/kg) significantly increased joint compression thresholds 5 hours after treatment on Day 14 compared to vehicle treated animals (**Figure 49**). Daily administration of Fluocinolone had no effect on pre-treatment joint compression thresholds when compared to vehicle treated animals or to Day 7 pre-treatment values (data not shown).

Figure 49 - Effect of Fluocinolone on MIA-induced mechanical hyperalgesia on Day 14

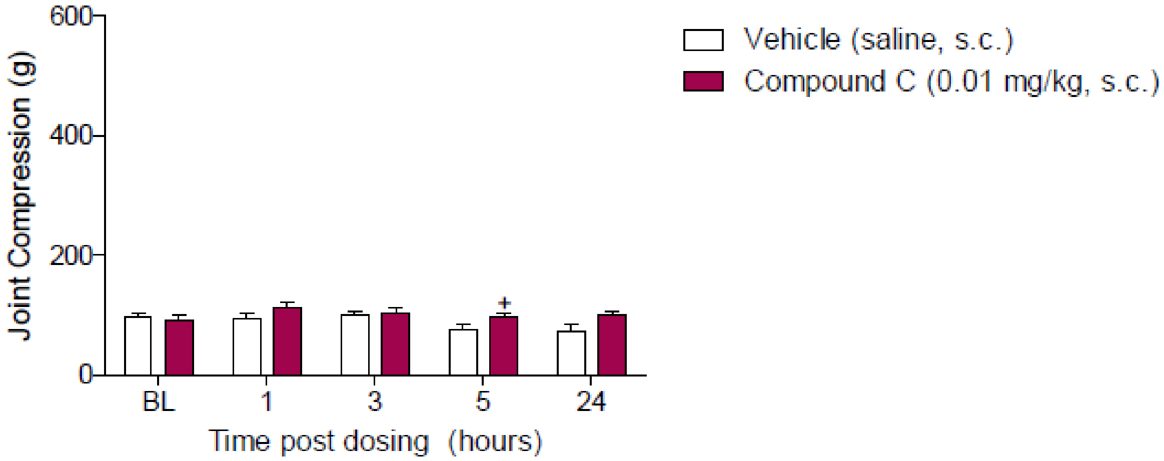

MIA Trial 3: Digital Randall-Selitto (dRS) Test

In Trial 3, the dRS test was used again to measure pain in the MIA model to test efficacy of clonidine, tacrolimus, fluocinolone, meloxicam, tranilast, and triamcinolone hexacetonide when delivered via intra-articular injection. Joint compression thresholds were measured 7, 14, 21 and 28 days following intra-articular injection of MIA (**Figures 50-54** below the descriptions). Joint compression thresholds were comparable among groups ($p = 1.0$, one-way ANOVA) on Day 7 after MIA injection. Differences between ipsilateral (injured) and contralateral (normal) joint compression thresholds within the vehicle group were statistically significant at all times post MIA injection, indicating the presence of mechanical hyperalgesia as a result of the MIA injection (see Appendix).

Excluded animals: Two animals were excluded from analysis in this study due to errors in dosing. Rat #98 received the wrong compound on Day 14, however, this rat received the correct compound on the other days. Rat #18 received the wrong compound on Day 28. The data from each animal prior to being dosed with the wrong compound was included in the analysis.

1) Saline (vehicle)

Administration of vehicle (saline, i.a.) had no significant effect on ipsilateral pre-dosing joint compression thresholds on any testing day compared to Day 7 values (**Figure 50**). Administration of saline (vehicle, i.a.) also had no significant effect on joint compression thresholds following dosing at any time point tested compared to the corresponding pre-treatment baseline values.

2) Clonidine

Systemic clonidine (0.1 mg/kg, s.c.) did not significantly increase ipsilateral pre-treatment joint compression thresholds on Days 14, 21 or 28 compared to Day 7 pre-treatment baseline values. However, a significant decrease in pre-treatment joint compression thresholds was observed on Day 28 compared to Day 7 pre-treatment baseline values (not shown). Similar to Trial 2 above, administration of systemic clonidine (0.1 mg/kg, s.c.) significantly increased joint compression thresholds 1, 3 and 5 hours after administration on Day 7 (**Figure 51**) and 1 and 3 hours after administration on Days 14, 21 and 28 compared to pre-treatment baseline values on the respective testing days (e.g. **Figure 52-54**).

3) IA clonidine (Compound A)

Weekly intra-articular administration (starting on Day 7) of Clonidine (4.5 μg) had no significant effect on pre-dosing joint compression thresholds on any testing day compared to Day 7 values or to vehicle treated animals **(Figure 50)**. Intra-articular administration of clonidine significantly increased joint compression thresholds 1 hour after treatment on Day 7 compared to vehicle treated animals (**Figure 51**). Administration of Clonidine had no significant effect on joint compression thresholds at any other time point tested when post-treatment joint compression thresholds were compared to vehicle treated animals

4) IA Tacrolimus (Compound B)

Weekly intra-articular administration (starting on Day 7) of Tacrolimus (30 ng, i.a.) had no significant effect on pre-treatment joint compression thresholds on any testing day compared to Day 7 values or to vehicle treated animals (**Figure 50**). Intra-articular administration of Tacrolimus also had no significant effect on joint compression thresholds at any time point after administration or day tested compared to vehicle treated animals **(Figure 51-54).**

5) IA Fluocinolone (Compound C)

Weekly intra-articular administration (starting on Day 7) of Fluocinolone (15 ng, i.a.) had no significant effect on pre-treatment joint compression thresholds on any testing day compared to Day 7 values or to vehicle treated animals **(Figure 50)**. However, acute intra-articular administration of Fluocinolone significantly increased joint compression thresholds 1 hour after treatment on Day 7 **(Figure 51)** compared to vehicle treated animals but not at any other time point or day tested. A significant decrease in joint compression threshold compared to vehicle treated animals was observed at the 24 hour time point 28 days after MIA injection **(Figure 54)**.

6) IA Meloxicam (Compound D)

Weekly intra-articular administration (starting on Day 7) of Meloxicam (0.1 mg) had no significant effect on pre-treatment joint compression thresholds on any testing day (**Figure 50**). However, acute intra-articular administration of Meloxicam significantly increased joint compression thresholds 1 hour after treatment on Day 7 **(Figure 51)** and 3 hours after treatment on Day 14 **(Figure 52)** compared to vehicle treated animals. Administration of Meloxicam had no effect on joint compression thresholds at any other time point tested when post-treatment joint compression thresholds were compared to vehicle treated animals.

7) IA Tranilast (Compound E)

Weekly intra-articular administration (starting on Day 7) of Tranilast (500 ng) had no significant effect on pre-dosing joint compression thresholds on any testing day compared to Day 7 values or to vehicle treated animals (**Figure 50**). However, acute intra-articular administration of Tranilast significantly increased joint compression thresholds 1 and 3 hours after treatment on Day 7 **(Figure 51)** and 1 hour after treatment on Days 14 and 21 **(Figures 52 and 53)** compared to vehicle treated animals.

8) IA Triamcinolone Hexacetonide (TH) (Compound F)

A significant decrease in pre-dosing joint compression threshold was observed on Day 21 after weekly intra-articular administration (starting on Day 7) of TH (0.15 mg, i.a.) compared to Day 7 pre-treatment baseline values (**Figure 50**). Weekly administration of TH did not significantly increase pre-treatment joint compression thresholds on any testing day compared to Day 7 values or to vehicle treated animals. Intra-articular administration of TH significantly increased joint compression thresholds 3 hours after treatment on Day 7 **(Figure 51)** and 1 hour after treatment on Day 14 **(Figure 52)** compared to vehicle treated animals. A significant decrease in joint compression threshold compared to vehicle treated animals was observed at the 24 hour time point 28 days after MIA injection **(Figure 54).**

Figure 50 – Pre-dosing effect of weekly intra-articular Test Compound administration on MIA-induced mechanical hyperalgesia in the rat measured prior to dosing and compared to day 7 pretreatment baseline values. Clonidine = A; Tacrolimus = B; Fluocinolone = C; Meloxicam = D; Tranilast = E; Triamcinolone hexacetonide =F.

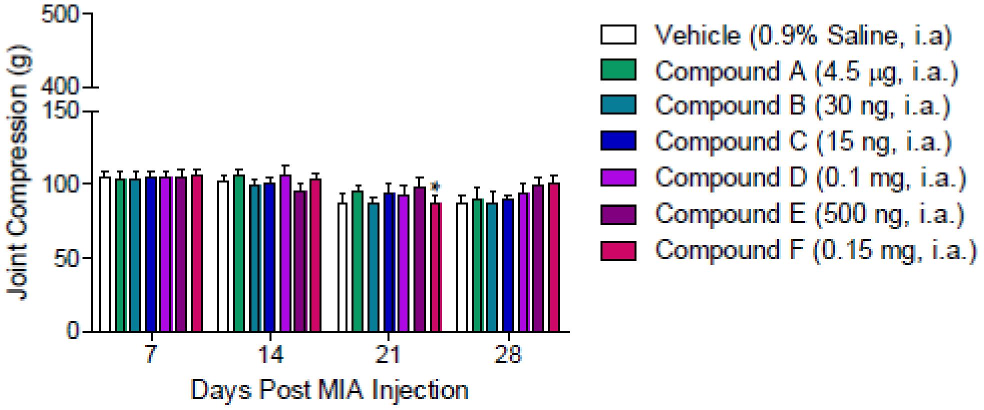

Figure 51 - Effect of the intra-articular Test Compounds on MIA-induced mechanical hyperalgesia on Day 7. Clonidine = A; Tacrolimus = B; Fluocinolone = C; Meloxicam = D; Tranilast = E; Triamcinolone hexacetonide =F.

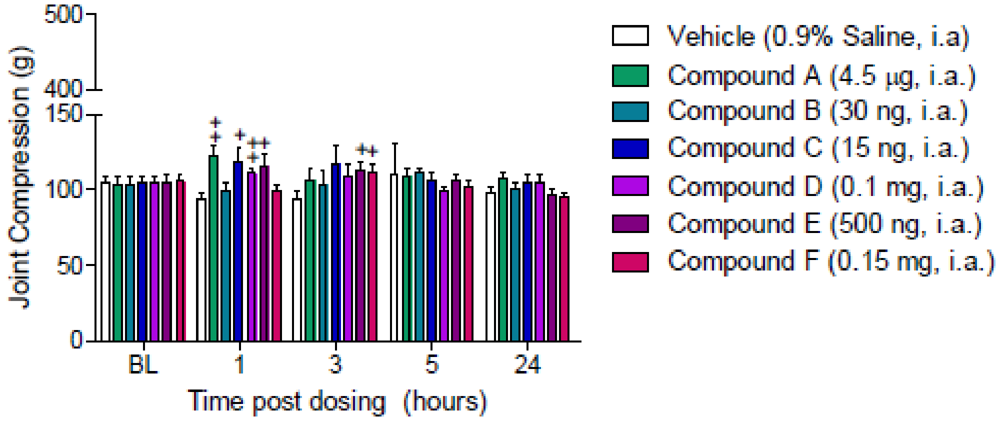

Figure 52 - Effect of the intra-articular Test Compounds on MIA-induced mechanical hyperalgesia on Day 14. Clonidine = A; Tacrolimus = B; Fluocinolone = C; Meloxicam = D; Tranilast = E; Triamcinolone hexacetonide =F.

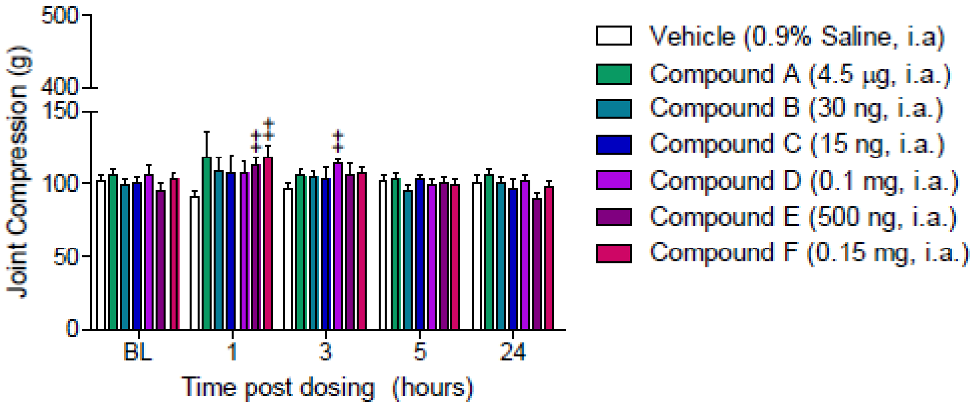

Figure 53 - Effect of the intra-articular Test Compounds on MIA-induced mechanical hyperalgesia on Day 21. Clonidine = A; Tacrolimus = B; Fluocinolone = C; Meloxicam = D; Tranilast = E; Triamcinolone hexacetonide =F.

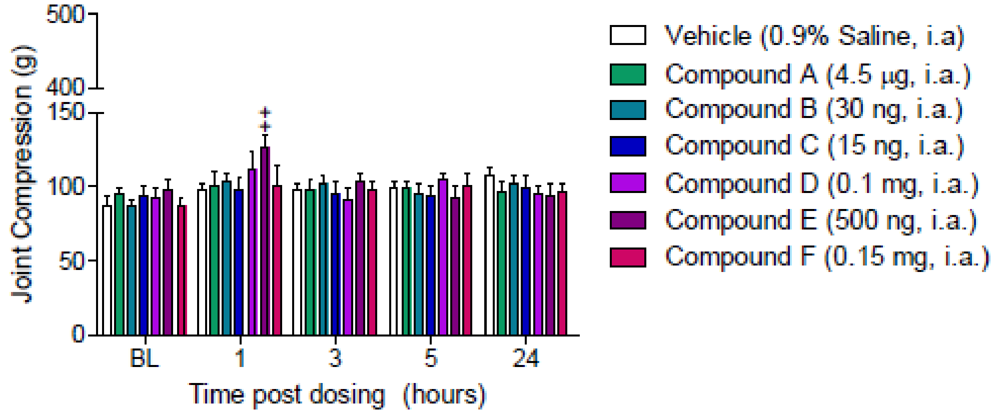

Figure 54 - Effect of the intra-articular Test Compounds on MIA-induced mechanical hyperalgesia on Day 28. Clonidine = A; Tacrolimus = B; Fluocinolone = C; Meloxicam = D; Tranilast = E; Triamcinolone hexacetonide =F.

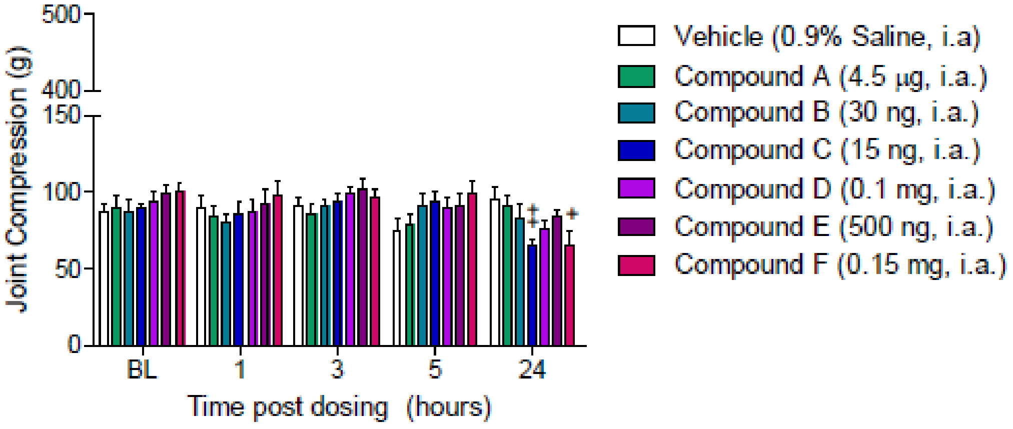

MIA Model Histopathology Results

MIA Trial 1: Histopathology

The average percentage chondrocyte degeneration for each group is shown in Table 5.

Table 5. Percent area chondrocyte necrosis / proteoglycan degeneration MIA Trial 1

Group	Necropsy Day	Treatment	Individual Animal Highest Score			Group Average
			IAD	IAD	IAD	
1	21	No Treatment	4	5	-	4.5
2	7	Vehicle (5 ml/kg)	5	4	5	4.7
3	14	Vehicle (5 ml/kg)	5	5	5	5.0
4	21	Vehicle (5 ml/kg)	5	5	4	4.7
5	29	Vehicle (5 ml/kg)	5	4	5	4.7
6	29	Clonidine (100 µg/kg)	5	4	5	4.7
7	29	Fluocinolone (2 µg/kg)	3	3	3	3.0

IAD = Individual animal data highest score of all sites examined for each animal.

Table 6. Average percent area chondrocyte necrosis / proteoglycan degeneration MIA Trial 1

Group	Day Necropsy	Treatment	Average across tibial and femoral cartilage			Average
			(Individual animal averages)			(all)
**1**	21	None	3	4.25	-	3.63
**2**	7	Vehicle	4.25	4.25	4.5	4.33
**3**	14	Vehicle	4.5	4.75	5.0	4.75
**4**	21	Vehicle	4.5	4.5	2.75	3.9
**5**	29	Vehicle	3.75	4.75	5.0	4.5
**6**	29	Clonidine	4.5	3.5	4.5	4.17
**7**	29	Fluocinolone	1	3	2.5	2.17

Semi-quantitative Analysis of Histological Sections MIA Trial 1

Table 7. Unmasked individual animal histopathological data for each treatment in MIA Trial 1

A) Group 1. Individual animal data after 21 days of no treatment

Group	1 - No Treatment - Necropsy Day 21							
Animal ID	3				21			
Site	MTP	MFC	LTP	LFC	MTP	MFC	LTP	LFC
**% Area of Chondrocyte Necrosis/ proteoglycan degeneration**	4	2	4	2	4	4	4	5
**Safranin - O**								
decreased proteoglycan	2	2	2	2	2	2	2	2
**Synovial membrane**								
fibrin/edema	0	0	0	0	0	0	0	0
lymphocytes	0	0	0	0	0	0	0	0
macrophages	0	0	0	0	0	0	0	0
neutrophils	0	0	0	0	0	0	0	0
hyperplasia	0	0	0	0	0	0	0	0
osteophytes	0	0	0	0	0	0	0	0
plasma cells	0	0	0	0	0	0	0	0
multinucleated cells	0	0	0	0	0	0	0	0
**Subchondral bone**								
increased osteoclasts	0	0	0	0	0	0	0	0
increased osteoblasts	0	0	0	0	0	0	0	0
increased fibrous stroma	+	+	+	+	+	+	+	+
decreased hematopoietic tissue	+	+	+	+	+	+	+	+
collapse of trabeculae	+	+	+	+	+	+	+	+
bone necrosis	0	0	0	0	0	0	0	0
bone regeneration	0	0	0	0	0	0	0	0
subchondral cyst	0	0	0	0	0	0	0	0
lymphocytes	0	0	0	0	0	0	0	0
macrophages	0	0	0	0	0	0	0	0
neutrophils	0	0	0	0	0	0	0	0
plasma cells	0	0	0	0	0	0	0	0
multinucleated cells	0	0	0	0	0	0	0	0
**Articular Cartilage**								
chondrocyte necrosis	+	+	+	+	+	+	+	+
erosion/fissues of articular surface	+	+	+	+	+	+	+	+
decreased thickness	+	+	+	+	0	0	0	0
cartilage matrix degeneration	+	+	+	+	+	+	+	+
clustering chondrocytes	0	+	0	+	+	+	+	0
chondrocyte proliferation	0	+	0	+	0	0	0	0
fibrocartilage	0	0	0	0	0	0	0	0

B) Group 2. Individual animal data after 7 days of treatment with vehicle alone

Group	2 - Vehicle (5 ml/kg) - Necropsy Day 7											
Animal ID	54				55				56			
Site	MTP	MFC	LTP	LFC	MTP	MFC	LTP	LFC	MTP	MFC	LTP	LFC
**% Area of Chondrocyte Necrosis/ proteoglycan degeneration**	4	5	4	4	4	4	4	4	5	4	4	5
**Safranin - O**												
decreased proteoglycan	3	2	3	2	3	3	3	4	3	3	3	4
**Synovial membrane**												
fibrin/edema	0	0	0	0	0	0	0	0	0	0	0	0
lymphocytes	0	0	0	0	0	0	0	0	0	0	0	0
macrophages	0	0	0	0	0	0	0	0	0	0	0	0
neutrophils	0	0	0	0	0	0	0	0	0	0	0	0
hyperplasia	0	0	0	0	0	0	0	0	0	0	0	0
osteophytes	0	0	0	0	0	0	0	0	0	0	0	0
plasma cells	0	0	0	0	0	0	0	0	0	0	0	0
multinucleated cells	0	0	0	0	0	0	0	0	0	0	0	0
**Subchondral bone**												
increased osteoclasts	+	0	+	0	+	+	+	+	+	0	+	0
increased osteoblasts	+	0	+	0	+	+	+	+	+	0	+	0
increased fibrous stroma	+	+	+	+	+	+	+	+	+	0	+	0
decreased hematopoietic tissue	0	0	0	0	0	0	0	0	0	0	0	0
collapse of trabeculae	+	+	+	+	+	+	+	+	+	+	+	+
bone necrosis	+	+	+	+	+	+	+	+	+	+	+	+
bone regeneration	0	0	0	0	0	0	0	0	+	0	+	0
subchondral cyst	0	0	0	0	0	0	0	0	0	0	0	0
lymphocytes	0	0	0	0	0	0	0	0	0	0	0	0
macrophages	0	0	0	0	0	0	0	0	0	0	0	0
neutrophils	0	0	0	0	0	0	0	0	0	0	0	0
plasma cells	0	0	0	0	0	0	0	0	0	0	0	0
multinucleated cells	0	0	0	0	0	0	0	0	0	0	0	0
**Articular Cartilage**												
chondrocyte necrosis	+	+	+	+	+	+	+	+	+	+	+	+
erosion/fissues of articular surface	+	+	+	+	0	0	+	0	+	+	+	+
decreased thickness	+	+	+	+	+	+	+	+	0	0	0	0
cartilage matrix degeneration	+	+	+	+	+	+	+	+	+	+	+	+
clustering chondrocytes	0	0	0	0	0	0	0	0	0	0	0	0
chondrocyte proliferation	0	0	0	0	0	0	0	0	0	0	0	0
fibrocartilage	0	0	0	0	0	0	0	0	0	0	0	0

C) Group 3. Individual animal data after 14 days of treatment with vehicle alone

Group	3 - Vehicle (5ml/kg) - Necropsy Day 14											
Animal ID	38				46				52			
Site	MTP	MFC	LTP	LFC	MTP	MFC	LTP	LFC	MTP	MFC	LTP	LFC
**% Area of Chondrocyte Necrosis/ proteoglycan degeneration**	4	5	4	5	4	5	5	5	5	5	5	5
**Safranin - O**												
decreased proteoglycan	3	3	3	3	3	3	3	3	3	3	4	4
**Synovial membrane**												
fibrin/edema	0	0	0	0	0	0	0	0	0	0	0	0
lymphocytes	0	0	0	0	0	0	0	0	0	0	0	0
macrophages	0	0	0	0	0	0	0	0	0	0	0	0
neutrophils	0	0	0	0	0	0	0	0	0	0	0	0
hyperplasia	0	0	0	0	0	0	0	0	0	0	0	0
osteophytes	0	0	0	0	0	0	0	0	0	0	0	0
plasma cells	0	0	0	0	0	0	0	0	0	0	0	0
multinucleated cells	0	0	0	0	0	0	0	0	0	0	0	0
**Subchondral bone**												
increased osteoclasts	+	+	+	+	+	+	+	+	+	+	+	+
increased osteoblasts	+	+	+	+	+	+	+	+	+	+	+	+
increased fibrous stroma	+	+	+	+	+	+	+	+	+	+	+	+
decreased hematopoietic tissue	+	+	+	+	+	+	+	+	+	+	+	+
collapse of trabeculae	0	0	0	0	+	0	+	0	+		+	
bone necrosis	+	+	+	+	+	+	+	+	+	+	+	+
bone regeneration	0	0	0	0	0	0	0	0	0	0	0	0
subchondral cyst	0	0	0	0	0	0	0	0	0	0	0	0
lymphocytes	0	0	0	0	0	0	0	0	0	0	0	0
macrophages	0	0	0	0	0	0	0	0	0	0	0	0
neutrophils	0	0	0	0	0	0	0	0	0	0	0	0
plasma cells	0	0	0	0	0	0	0	0	0	0	0	0
multinucleated cells	0	0	0	0	0	0	0	0	0	0	0	0
**Articular Cartilage**												
chondrocyte necrosis	+	+	+	+	+	+	+	+	+	+	+	+
erosion/fissues of articular surface	0	0	0	+	0	0	0	+	0	0	0	0
decreased thickness	0	0	0	0	0	0	0	0	0	0	0	0
cartilage matrix degeneration	+	+	+	+	+	+	+	+	+	+	+	+
clustering chondrocytes	0	0	0	0	0	0	0	0	0	0	0	0
chondrocyte proliferation	0	0	0	0	0	0	0	0	0	0	0	0
fibrocartilage	0	0	0	0	0	0	0	0	0	0	0	0

D) Group 4. Individual animal data after 21 days of treatment with vehicle alone

Group	4 - Vehicle (5ml/kg) - Necropsy Day 21											
Animal ID	12				22				29			
Site	**MTP**	**MFC**	**LTP**	**LFC**	**MTP**	**MFC**	**LTP**	**LFC**	**MTP**	**MFC**	**LTP**	**LFC**
**% Area of Chondrocyte Necrosis/ proteoglycan degeneration**	4	5	4	5	4	5	4	5	4	3	2	2
**Safranin - O**												
decreased proteoglycan	3	3	3	3	4	3	4	3	2	1	1	1
**Synovial membrane**												
fibrin/edema	0	0	0	0	0	0	0	0	0	0	0	0
lymphocytes	0	0	0	0	0	0	0	0	0	0	0	0
macrophages	0	0	0	0	0	0	0	0	0	0	0	0
neutrophils	0	0	0	0	0	0	0	0	0	0	0	0
hyperplasia	0	0	0	0	0	0	0	0	0	0	0	0
osteophytes	0	+	0	0	0	0	0	0	0	0	0	0
plasma cells	0	0	0	0	0	0	0	0	0	0	0	0
multinucleated cells	0	0	0	0	0	0	0	0	0	0	0	0
**Subchondral bone**												
increased osteoclasts	+	+	+	+	+	+	+	+	0	0	0	0
increased osteoblasts	+	+	+	+	+	+	+	+	0	0	0	0
increased fibrous stroma	+	+	+	+	+	+	+	+	0	0	0	0
decreased hematopoietic tissue	+	+	+	+	0	0	0	0	0	0	0	0
collapse of trabeculae	+	+	+	+	+	+	+	+	0	0	0	0
bone necrosis	+	+	+	+	0	0	0	0	0	0	0	0
bone regeneration	0	0	0	0	0	0	0	0	0	0	0	0
subchondral cyst	0	0	0	0	0	0	0	0	0	0	0	0
lymphocytes	0	0	0	0	0	0	0	0	0	0	0	0
macrophages	0	0	0	0	0	0	0	0	0	0	0	0
neutrophils	0	0	0	0	0	0	0	0	0	0	0	0
plasma cells	0	0	0	0	0	0	0	0	0	0	0	0
multinucleated cells	0	0	0	0	0	0	0	0	0	0	0	0
**Articular Cartilage**												
chondrocyte necrosis	+	+	+	+	+	+	+	+	+	+	+	+
erosion/fissues of articular surface	+	+	+	+	+	+	+	+	0	0	0	0
decreased thickness	0	0	0	0	+	+	+	+	0	0	0	0
cartilage matrix degeneration	+	+	+	+	+	+	+	+	+	+	+	+
clustering chondrocytes	0	0	0	0	0	0	0	0	+	+	+	+
chondrocyte proliferation	0	0	0	0	0	0	0	0	0	0	0	0
fibrocartilage	0	0	0	0	0	0	0	0	0	0	0	0

E) Group 5. Individual animal data after 29 days of treatment with vehicle alone

Group	5 - Vehicle (5ml/kg) - Necropsy Day 29											
Animal ID	23				31				41			
Site	MTP	MFC	LTP	LFC	MTP	MFC	LTP	LFC	MTP	MFC	LTP	LFC
**% Area of Chondrocyte Necrosis/ proteoglycan degeneration**	4	3	5	3	4	3	4	4	5	5	5	5
**Safranin - O**												
decreased proteoglycan	4	3	3	3	3	3	3	3	3	3	3	3
**Synovial membrane**												
fibrin/edema	0	0	0	0	0	0	0	0	0	0	0	0
lymphocytes	0	0	0	0	0	0	0	0	0	0	0	0
macrophages	0	0	0	0	0	0	0	0	0	0	0	0
neutrophils	0	0	0	0	0	0	0	0	0	0	0	0
hyperplasia	0	0	0	0	0	0	0	0	0	0	0	0
osteophytes	0	0	0	0	0	0	0	0	0	0	0	0
plasma cells	0	0	0	0	0	0	0	0	0	0	0	0
multinucleated cells	0	0	0	0	0	0	0	0	0	0	0	0
**Subchondral bone**												
increased osteoclasts	0	0	0	0	0	0	0	0	0	0	0	0
increased osteoblasts	0	0	0	0	0	0	0	0	0	0	0	0
increased fibrous stroma	0	0	0	0	0	0	0	0	+	+	+	+
decreased hematopoietic tissue	+	0	+	0	+	+	+	+	+	+	+	+
collapse of trabeculae	+	0	+	0	+	+	+	+	+	+	+	+
bone necrosis	0	0	0	+	0	0	0	0	+	+	+	+
bone regeneration	0	0	0	0	0	0	0	0	0	0	0	0
subchondral cyst	0	0	0	0	0	0	0	0	0	0	0	0
lymphocytes	0	0	0	0	0	0	0	0	0	0	0	0
macrophages	0	0	0	0	0	0	0	0	0	0	0	0
neutrophils	0	0	0	0	0	0	0	0	0	0	0	0
plasma cells	0	0	0	0	0	0	0	0	0	0	0	0
multinucleated cells	0	0	0	0	0	0	0	0	0	0	0	0
**Articular Cartilage**												
chondrocyte necrosis	+	+	+	+	+	+	+	+	+	+	+	+
erosion/fissues of articular surface	+	+	+	+	+	+	+	+	+	+	+	+
decreased thickness	+	+	+	+	+	+	+	+	+	+	+	+
cartilage matrix degeneration	+	+	+	+	+	+	+	+	+	+	+	+
clustering chondrocytes	0	+	0	0	+	+	+	+	0	0	0	0
chondrocyte proliferation	0	+	0	0	0	+	0	+	0	0	0	0
fibrocartilage	0	0	0	0	0	0	0	0	0	0	0	0

F) Group 6. Clonidine treated animals (100 ug/kg) necropsied at day 29

Group	6 - Clonidine (100 µg/kg) - Necropsy Day 29											
Animal ID	28				32				36			
Site	MTP	MFC	LTP	LFC	MTP	MFC	LTP	LFC	MTP	MFC	LTP	LFC
**% Area of Chondrocyte Necrosis/ proteoglycan degeneration**	5	4	5	4	3	3	4	4	5	4	4	5
**Safranin - O**												
decreased proteoglycan	3	3	3	3	3	3	3	3	4	3	3	3
**Synovial membrane**												
fibrin/edema	0	0	0	0	0	0	0	0	0	0	0	0
lymphocytes	0	0	0	0	0	0	0	0	0	0	0	0
macrophages	0	0	0	0	0	0	0	0	0	0	0	0
neutrophils	0	0	0	0	0	0	0	0	0	0	0	0
hyperplasia	0	0	0	0	0	0	0	0	0	0	0	0
osteophytes	0	0	0	0	0	0	0	0	0	0	0	0
plasma cells	0	0	0	0	0	0	0	0	0	0	0	0
multinucleated cells	0	0	0	0	0	0	0	0	0	0	0	0
**Subchondral bone**												
increased osteoclasts	+	+	0	0	0	0	0	0	0	0	0	0
increased osteoblasts	+	+	0	0	0	0	0	0	0	0	0	0
increased fibrous stroma	+	+	0	0	+	+	+	+	+	+	+	+
decreased hematopoietic tissue	+	+	+	+	+	+	+	+	+	+	+	+
collapse of trabeculae	+	+	+	+	+	+	+	+	+	+	+	+
bone necrosis	+	+	0	0	0	0	0	0	0	0	0	0
bone regeneration	0	0	0	0	+	+	0	0	0	0	0	0
subchondral cyst	0	0	0	0	0	0	0	0	0	0	0	0
lymphocytes	0	0	0	0	0	0	0	0	0	0	0	0
macrophages	0	0	0	0	0	0	0	0	0	0	0	0
neutrophils	0	0	0	0	0	0	0	0	0	0	0	0
plasma cells	0	0	0	0	0	0	0	0	0	0	0	0
multinucleated cells	0	0	0	0	0	0	0	0	0	0	0	0
**Articular Cartilage**												
chondrocyte necrosis	+	+	+	+	+	+	+	+	+	+	+	+
erosion/fissues of articular surface	+	+	+	+	+	+	+	+	+	+	+	+
decreased thickness	+	+	+	+	+	+	+	+	+	+	+	+
cartilage matrix degeneration	+	+	+	+	+	+	+	+	+	+	+	+
clustering chondrocytes	0	0	0	0	0	0	0	0	0	0	0	0
chondrocyte proliferation	0	0	0	0	0	0	0	0	0	0	0	0
fibrocartilage	0	0	0	0	0	0	0	0	0	0	0	0

G) Group 7. Fluocinolone treated animals (2 ug/kg) animals necropsied at day 29

Group	7 - Fluocinolone (2 µg/kg) - Necropsy Day 29											
Animal ID	4				15				25			
Site	MTP	MFC	LTP	LFC	MTP	MFC	LTP	LFC	MTP	MFC	LTP	LFC
**% Area of Chondrocyte Necrosis/ proteoglycan degeneration**	3	0	0	0	3	3	3	3	3	2	3	2
**Safranin - O**												
decreased proteoglycan	2	1	1	1	3	3	3	3	2	3	2	3
**Synovial membrane**												
fibrin/edema	0	0	0	0	0	0	0	0	0	0	0	0
lymphocytes	0	0	0	0	0	0	0	0	0	0	0	0
macrophages	0	0	0	0	0	0	0	0	0	0	0	0
neutrophils	0	0	0	0	0	0	0	0	0	0	0	0
hyperplasia	0	0	0	0	0	0	0	0	0	0	0	0
osteophytes	0	0	0	0	0	0	0	0	0	0	0	0
plasma cells	0	0	0	0	0	0	0	0	0	0	0	0
multinucleated cells	0	0	0	0	0	0	0	0	0	0	0	0
**Subchondral bone**												
increased osteoclasts	0	0	0	0	0	0	0	0	0	0	0	0
increased osteoblasts	0	0	0	0	0	0	0	0	0	0	0	0
increased fibrous stroma	0	0	0	0	+	+	+	+	0	0	0	0
decreased hematopoietic tissue	+	+	+	+	+	+	+	+	+	+	+	+
collapse of trabeculae	+	+	+	+	+	+	+	+	+	+	+	+
bone necrosis	0	0	0	0	0	0	0	0	0	0	0	0
bone regeneration	+	+	+	+	0	+	0	+	0	0	0	0
subchondral cyst	0	0	0	0	0	0	0	0	0	0	0	0
lymphocytes	0	0	0	0	0	0	0	0	0	0	0	0
macrophages	0	0	0	0	0	0	0	0	0	0	0	0
neutrophils	0	0	0	0	0	0	0	0	0	0	0	0
plasma cells	0	0	0	0	0	0	0	0	0	0	0	0
multinucleated cells	0	0	0	0	0	0	0	0	0	0	0	0
**Articular Cartilage**												
chondrocyte necrosis	+	+	+	+	+	+	+	+	+	+	+	+
erosion/fissues of articular surface	0	0	0	0	+	+	+	+	0	0	0	0
decreased thickness	0	0	0	0	0	0	0	0	0	0	0	0
cartilage matrix degeneration	+	+	+	+	+	+	+	+	+	+	+	+
clustering chondrocytes	+	+	+	+	+	+	+	+	+	+	+	+
chondrocyte proliferation	+	+	+	+	+	+	+	+	+	+	+	+
fibrocartilage	0	0	0	0	0	0	0	0	0	0	0	0

Moderate to severe degenerative changes were identified in all groups in both the lateral and medial femoral condyle and lateral and medial tibial plateau and in the articular cartilage and subchondral bone. The articular cartilage alterations were characterized by degeneration of matrix with loss of proteoglycans, necrosis of chondrocytes and erosion and fissures in the articular surface. The degenerative changes extended into the subchondral bone in many rats and were characterized by collapse of the trabeculae, proliferation of fibrous connective tissue and remodeling of the bone.

Alterations were scored according to the scale described in the methods. Differences among the individual rats could be broadly divided into 3 responses: 1) Rats in which the articular cartilage was present over the majority of the joint surface with focal to extensive degeneration of the matrix/necrosis of chondrocytes, 2) Rats with erosion/loss of the cartilage over the majority of the joint surfaces, with a minimal fibrous connective tissue response/collapse of trabeculae in the subchondral bone, and 3) Rats with erosion of the cartilage over the majority of the joint surfaces and with moderate fibrous connective tissue proliferation/collapse of trabeculae in the subchondral bone. In the first group, rats with articular cartilage present over the majority of the joint surface, the articular cartilage was pale and eosinophilic with few chondrocytes. The chondrocytes were mostly necrotic, i.e., exhibiting cartilage necrosis and degeneration. The thickness of the degenerate articular cartilage varied from near normal thickness to markedly thin. The subchondral bone had variable collapse of trabeculae and minimal fibrous connective tissue, osteoblasts and osteoclasts. In the second group, rats with almost no articular cartilage and exposed subchondral bone, there were some rats with minimal collapse of the underlying trabecular bone, accompanied by increased minimal increased fibrous connective tissue in the marrow adjacent to the articular surface. In the third group, there was marked collapse of the trabeculae, moderately decreased

hemopoietic tissue, increased fibrous connective tissue with abundant osteoclasts and osteoblasts lining the trabeculae.

In addition to the changes described above, some individual rats had necrosis of osteoid or regeneration of cartilage at the edge of the articular surface. Inflammatory cells (lymphocytes, macrophages, plasma cells, neutrophils) were not observed in either the bone or the soft tissues, and no changes were seen in the synovial membrane. The untreated control group, the 14, 21, and 29 day vehicle control groups, and the clonidine group were considered to be similarly affected, with the majority of sites scoring 4 or 5 based on the scale of percentage of area of articular degeneration/necrosis. The exception was rat #29 in the 21 day vehicle control group. In this rat, the femoral condyles and tibial plateaus were less severely affected than other rats: the cartilage degeneration was less extensive and there was minimal degeneration or remodeling of the subchondral bone. The articular cartilage also had some evidence of regeneration – clustering of chondrocytes, with focal invagination of the cartilage into the subchondral bone. The 7 day vehicle control group had severe degenerative changes but had greater numbers of necrotic chondrocytes and preservation of the architecture in necrotic articular cartilage, with minimal fibrous connective tissue response in the subchondral bone. The Fluocinolone group was considered the least severely affected, with overall lower scores on the percentage area of degenerative cartilage. Further, the rats in this group had minimal proliferation of fibrous connective tissue/ collapse of the trabeculae/subchondral bone remodeling.

Conclusions:
In this first trial of MIA treatment, moderate to severe degenerative changes were identified in all but the Fluocinolone group in both the lateral and medial femoral condyle and lateral and medial tibial plateau, in the articular cartilage, and in the subchondral bone. The articular cartilage alterations were characterized by degeneration of matrix, loss of proteoglycans, necrosis of chondrocytes and erosion of the articular surface. The degenerative changes extended into the subchondral bone in many rats, most notable in the untreated controls, 14-29 day groups, and the clonidine group: characterized by collapse of the trabeculae, proliferation of fibrous connective tissue and remodeling of the bone. Thus extension of degenerative changes into the sub-chondral bone was associated with time points of 14 days or greater, and was not observed at 7 days.

The vehicle alone and Clonidine (100 ug/kg) group were not significantly different from the rats that received no treatment. The group treated with Fluocinolone (2μg/kg) had moderate degenerative changes characterized by moderately extensive degeneration of the articular cartilage and minimal alterations in the subchondral bone. This group was less severely affected than other treatment groups.

MIA Trial 2: Histopathology

Semi-quantitative Analysis of Histological Sections MIA Trial 2

A comprehensive histopathological analysis was completed in Trial 2 with four animals from each group (below). The added detail provided in Figures 54-65 shows that some agents had significant effects on proteoglycan and matrix loss in specific regions but such significance may have been lost with calculation of the total joint score. For example, animals treated with fluocinolone (Tables 16 & 17; 0.01 mg/ml- a slightly lower concentration than was used in the previous study) showed a significant inhibition of the loss of cartilaginous matrix and bone resorption in both the medial tibial plateau and the medial femoral condyle and yet did not show a significant change in total joint scores. Significant t values are highlighted in red. Note also that in some cases the results trended toward improvement but were variable enough not to be significant.

1) Vehicle Control (Saline)

Table 8. Histopathological analysis of proteoglycan and matrix loss in Vehicle Controls in MIA Trial 2.

Group 1 Vehicle (saline)	MTP % loss of Cell	MTP % loss of PG	MTP % loss of Matrix	MTP Mean	MFC % loss of Cell	MFC % loss of PG	MFC % loss of Matrix	MFC Mean	LTP % loss of Cell	LTP % loss of PG	LTP % loss of Matrix	LTP Mean	LFC % loss of Cell	LFC % loss of PG	LFC % loss of Matrix	LFC Mean
9	100%	99%	80%	93%	100%	99%	70%	90%	100%	99%	80%	93%	100%	99%	70%	90%
14	100%	99%	70%	90%	100%	99%	50%	83%	100%	99%	80%	93%	100%	99%	80%	93%
19	100%	99%	80%	93%	90%	85%	50%	75%	100%	99%	80%	93%	100%	100%	100%	100%
34	95%	90%	5%	63%	65%	60%	5%	43%	65%	60%	30%	52%	80%	75%	30%	62%
**Mean**	**99%**	**97%**	**59%**	**85%**	**89%**	**86%**	**44%**	**73%**	**91%**	**89%**	**68%**	**83%**	**95%**	**93%**	**70%**	**86%**
**SE**	**#####**	**2%**	**18%**	**7%**	**8%**	**9%**	**14%**	**10%**	**9%**	**10%**	**13%**	**10%**	**5%**	**6%**	**15%**	**8%**
**% Inhibition**	**0%**	**0%**	**0%**	**0%**	**0%**	**0%**	**0%**	**0%**	**0%**	**0%**	**0%**	**0%**	**0%**	**0%**	**0%**	**0%**

Table 9. Histopathological analysis of cartilage, bone, and synovium in Vehicle Controls in MIA Trial 2.

Group 1 Vehicle (saline)	Mean of All Cartilage	Matrix Damage Score	Bone Resorption Score	Bone Resorption Percent of Surface MTP	MFC	LTP	LFC	Mean	Subchondral Bone Sclerosis Score/Surface	Osteophyte Score	Osteophyte Measure µm	Total Joint Score with Matrix	Total Joint Score w/o Matrix	Synovitis Comments
9	91%	5	5	100%	100%	100%	100%	100%	0	5	1000	15	10	Moderate fibrosis
14	90%	5	5	100%	100%	100%	100%	100%	1	5	1000	16	11	Moderate fibrosis with minimal subacute inflammation
19	90%	5	5	100%	100%	100%	100%	100%	0	5	1000	15	10	Moderate fibrosis
34	55%	3	5	90%	75%	100%	100%	91%	0	3	600	11	8	Mild fibrosis
**Mean**	**82%**	**4.5**	**5.0**	**98%**	**94%**	**100%**	**100%**	**98%**	**0.3**	**4.5**	**900.0**	**14.3**	**9.8**	
**SE**	**9%**	**0.5**	**0.0**	**3%**	**6%**	**0%**	**0%**	**2%**	**0.3**	**0.5**	**100.0**	**1.1**	**0.6**	
**% Inhibition**	**0%**	**0%**	**0%**	**0%**	**0%**	**0%**	**0%**	**0%**	**0%**	**0%**	**0%**	**0%**	**0%**	

2) Tacrolimus (0.3 mg/kg, i.p.)

Table 10. Histopathological analysis of proteoglycan and matrix loss in Tacrolimus-treated animals in MIA Trial 2.

Group 2 Tacrolimus 0.3	MTP % loss of Cell	MTP % loss of PG	MTP % loss of Matrix	MTP Mean	MFC % loss of Cell	MFC % loss of PG	MFC % loss of Matrix	MFC Mean	LTP % loss of Cell	LTP % loss of PG	LTP % loss of Matrix	LTP Mean	LFC % loss of Cell	LFC % loss of PG	LFC % loss of Matrix	LFC Mean
1	70%	65%	40%	58%	50%	45%	20%	38%	100%	99%	70%	90%	100%	99%	70%	90%
4	70%	65%	5%	47%	30%	25%	5%	20%	85%	80%	5%	57%	45%	40%	0%	28%
7	100%	99%	70%	90%	100%	99%	70%	90%	100%	99%	70%	90%	100%	99%	70%	90%
11	100%	99%	70%	90%	70%	70%	5%	48%	100%	99%	70%	90%	100%	99%	70%	90%
**Mean**	**85%**	**82%**	**46%**	**71%**	**63%**	**60%**	**25%**	**49%**	**96%**	**94%**	**54%**	**81%**	**86%**	**84%**	**53%**	**74%**
**SE**	**0.087**	**10%**	**15%**	**11%**	**15%**	**16%**	**15%**	**15%**	**4%**	**5%**	**16%**	**8%**	**14%**	**15%**	**18%**	**15%**
**t-test to grp 1**	**0.167**	**0.193**	**0.618**	**0.338**	**0.175**	**0.208**	**0.399**	**0.236**	**0.618**	**0.661**	**0.527**	**0.928**	**0.572**	**0.593**	**0.473**	**0.527**
**% Inhibition**	**14%**	**15%**	**21%**	**16%**	**30%**	**30%**	**43%**	**33%**	**-5%**	**-6%**	**20%**	**2%**	**9%**	**10%**	**25%**	**14%**

Table 11. Histopathological analysis of cartilage, bone, and synovium in Tacrolimus-treated animals in MIA Trial 2.

Group 2 Tacrolimus 0.3	Mean of All Cartilage	Matrix Damage Score	Bone Resorption						Subchondral Bone Sclerosis Score/Surface	Osteophyte		Total Joint Score		Synovitis Comments
				Percent of Surface										
			Score	MTP	MFC	LTP	LFC	Mean		Score	Measure µm	with Matrix	w/o Matrix	
1	69%	4	5	100%	100%	100%	100%	100%	0	5	1000	14	10	Moderate fibrosis
4	38%	2	3	75%	30%	50%	50%	51%	0	2	400	7	5	Minimal fibrosis
7	90%	5	5	100%	100%	100%	100%	100%	0	4	800	14	9	Moderate fibrosis
11	79%	4	4	100%	50%	100%	100%	88%	0	3	600	11	7	Mild fibrosis
**Mean**	**69%**	**3.8**	**4.3**	**94%**	**70%**	**88%**	**88%**	**85%**	**0.0**	**3.5**	**700.0**	**11.5**	**7.8**	
**SE**	**11%**	**0.6**	**0.5**	**6%**	**18%**	**13%**	**13%**	**12%**	**0.0**	**0.6**	**129.1**	**1.7**	**1.1**	
**t-test to grp 1**	**0.412**	**0.387**	**0.168**	**0.598**	**0.255**	**0.356**	**0.356**	**0.306**	**0.356**	**0.267**	**0.267**	**0.217**	**0.168**	
**% Inhibition**	**15%**	**17%**	**15%**	**4%**	**25%**	**13%**	**13%**	**13%**	**100%**	**22%**	**22%**	**19%**	**21%**	

3) Tacrolimus (0.6 mg/kg, i.p.)

Table 12. Histopathological analysis of proteoglycan and matrix loss in Tacrolimus-treated animals in MIA Trial 2.

Group 3 Tacrolimus 0.6	MTP				MFC				LTP				LFC			
	% loss of				% loss of				% loss of				% loss of			
	Cell	PG	Matrix	Mean	Cell	PG	Matrix	Mean	Cell	PG	Matrix	Mean	Cell	PG	Matrix	Mean
27	100%	99%	50%	83%	100%	99%	50%	83%	100%	99%	5%	68%	90%	85%	0%	58%
45	95%	95%	30%	73%	100%	99%	5%	68%	100%	99%	70%	90%	100%	99%	80%	93%
51	65%	60%	5%	43%	0%	0%	0%	0%	62%	60%	0%	41%	30%	25%	0%	18%
73	90%	85%	10%	62%	90%	85%	5%	60%	100%	99%	70%	90%	90%	85%	0%	58%
**Mean**	**88%**	**85%**	**24%**	**65%**	**73%**	**71%**	**15%**	**53%**	**91%**	**89%**	**36%**	**72%**	**78%**	**74%**	**20%**	**57%**
**SE**	**0.078**	**9%**	**10%**	**9%**	**24%**	**24%**	**12%**	**18%**	**9%**	**10%**	**20%**	**12%**	**16%**	**17%**	**20%**	**15%**
**t-test to grp 1**	**0.203**	**0.233**	**0.143**	**0.132**	**0.550**	**0.578**	**0.163**	**0.376**	**0.956**	**1.000**	**0.226**	**0.518**	**0.337**	**0.304**	**0.091**	**0.146**
**% Inhibition**	**11%**	**12%**	**60%**	**23%**	**18%**	**17%**	**66%**	**27%**	**1%**	**0%**	**46%**	**13%**	**18%**	**21%**	**71%**	**34%**

Table 13. Histopathological analysis of cartilage, bone, and synovium in Tacrolimus-treated animals in MIA Trial 2.

Group 3 Tacrolimus 0.6	Mean of All Cartilage	Matrix Damage Score	Bone Resorption						Subchondral Bone Sclerosis Score/Surface	Osteophyte		Total Joint Score		Synovitis Comments
				Percent of Surface										
			Score	MTP	MFC	LTP	LFC	Mean		Score	Measure µm	with Matrix	w/o Matrix	
27	73%	4	4	40%	100%	30%	100%	68%	0	4	800	12	8	Marked fibrosis
45	81%	5	4	100%	50%	100%	100%	88%	0	4	800	13	8	Marked fibrosis
51	26%	2	2	30%	0%	50%	20%	25%	0	0	0	4	2	Normal synovium
73	67%	4	5	100%	100%	100%	100%	100%	0	3	600	12	8	Moderate fibrosis
**Mean**	**62%**	**3.8**	**3.8**	**68%**	**63%**	**70%**	**80%**	**70%**	**0.0**	**2.8**	**550.0**	**10.3**	**6.5**	
**SE**	**12%**	**0.6**	**0.6**	**19%**	**24%**	**18%**	**20%**	**16%**	**0.0**	**0.9**	**189.3**	**2.1**	**1.5**	
**t-test to grp 1**	**0.241**	**0.387**	**0.094**	**0.166**	**0.253**	**0.143**	**0.356**	**0.144**	**0.356**	**0.153**	**0.153**	**0.143**	**0.093**	
**% Inhibition**	**24%**	**17%**	**25%**	**31%**	**33%**	**30%**	**20%**	**28%**	**100%**	**39%**	**39%**	**28%**	**33%**	

4) Curcumin (50 mg/kg, p.o.)

Table 14. Histopathological analysis of proteoglycan and matrix loss in Curcumin-treated animals in MIA Trial 2.

Group 4 Curcumin 50	MTP				MFC				LTP				LFC			
	% loss of				% loss of				% loss of				% loss of			
	Cell	PG	Matrix	Mean	Cell	PG	Matrix	Mean	Cell	PG	Matrix	Mean	Cell	PG	Matrix	Mean
5	100%	99%	70%	90%	100%	99%	70%	90%	100%	99%	80%	93%	100%	99%	70%	90%
21	100%	99%	70%	90%	100%	99%	50%	83%	100%	99%	80%	93%	100%	100%	100%	100%
30	100%	99%	50%	83%	100%	99%	50%	83%	100%	99%	70%	90%	100%	99%	80%	93%
35	100%	99%	30%	76%	100%	99%	50%	83%	100%	99%	80%	93%	100%	99%	80%	93%
**Mean**	**100%**	**99%**	**55%**	**85%**	**100%**	**99%**	**55%**	**85%**	**100%**	**99%**	**78%**	**92%**	**100%**	**99%**	**83%**	**94%**
**SE**	**0**	**0%**	**10%**	**3%**	**0%**	**0%**	**5%**	**2%**	**0%**	**0%**	**3%**	**1%**	**0%**	**0%**	**6%**	**2%**
**t-test to grp 1**	**0.356**	**0.356**	**0.861**	**0.992**	**0.222**	**0.200**	**0.471**	**0.295**	**0.356**	**0.356**	**0.463**	**0.395**	**0.356**	**0.363**	**0.465**	**0.402**
**% Inhibition**	**-1%**	**-2%**	**6%**	**0%**	**-13%**	**-15%**	**-26%**	**-16%**	**-10%**	**-11%**	**-15%**	**-11%**	**-5%**	**-6%**	**-18%**	**-9%**

Table 15. Histopathological analysis of cartilage, bone, and synovium in Curcumin-treated animals in MIA Trial 2.

Group 4 Curcumin 50	Mean of All Cartilage	Matrix Damage Score	Bone Resorption						Subchondral Bone Sclerosis Score/Surface	Osteophyte		Total Joint Score		Synovitis Comments
				Percent of Surface										
			Score	MTP	MFC	LTP	LFC	Mean		Score	Measure µm	with Matrix	w/o Matrix	
5	91%	5	5	100%	100%	100%	100%	100%	0	5	1000	15	10	Moderate fibrosis
21	91%	5	5	100%	100%	100%	100%	100%	0	5	1000	15	10	Moderate fibrosis
30	87%	5	4	60%	50%	100%	100%	78%	0	5	1200	14	9	Moderate fibrosis
35	86%	5	5	100%	100%	100%	100%	100%	0	4	800	14	9	Moderate fibrosis
**Mean**	**89%**	**5.0**	**4.8**	**90%**	**88%**	**100%**	**100%**	**94%**	**0.0**	**4.8**	**1000.0**	**14.5**	**9.5**	
**SE**	**1%**	**0.0**	**0.3**	**10%**	**13%**	**0%**	**0%**	**6%**	**0.0**	**0.3**	**81.6**	**0.3**	**0.3**	
**t-test to grp 1**	**0.446**	**0.356**	**0.356**	**0.494**	**0.670**	**#####**	**#####**	**0.590**	**0.356**	**0.670**	**0.468**	**0.834**	**0.730**	
**% Inhibition**	**-9%**	**-11%**	**5%**	**8%**	**7%**	**0%**	**0%**	**4%**	**100%**	**-6%**	**-11%**	**-2%**	**3%**	

5) Fluocinolone (0.01 mg/kg, s.c.)

Table 16. Histopathological analysis of proteoglycan and matrix loss in Fluocinolone-treated animals in MIA Trial 2.

Group 5 Fluocinolone 0.01	MTP				MFC				LTP				LFC			
	% loss of				% loss of				% loss of				% loss of			
	Cell	PG	Matrix	Mean	Cell	PG	Matrix	Mean	Cell	PG	Matrix	Mean	Cell	PG	Matrix	Mean
3	40%	35%	0%	25%	0%	0%	0%	0%	50%	45%	0%	32%	0%	0%	0%	0%
46	100%	99%	20%	73%	100%	99%	5%	68%	100%	99%	60%	86%	100%	99%	70%	90%
48	100%	99%	5%	68%	95%	90%	5%	63%	95%	90%	50%	78%	95%	90%	50%	78%
65	100%	99%	10%	70%	95%	90%	5%	63%	100%	99%	40%	80%	99%	95%	50%	81%
**Mean**	**85%**	**83%**	**9%**	**59%**	**73%**	**70%**	**4%**	**49%**	**86%**	**83%**	**38%**	**69%**	**74%**	**71%**	**43%**	**62%**
**SE**	**0.15**	**16%**	**4%**	**11%**	**24%**	**23%**	**1%**	**16%**	**12%**	**13%**	**13%**	**13%**	**25%**	**24%**	**15%**	**21%**
**t-test to grp 1**	**0.396**	**0.427**	**0.036**	**0.103**	**0.548**	**0.547**	**0.027**	**0.257**	**0.750**	**0.724**	**0.149**	**0.433**	**0.423**	**0.399**	**0.238**	**0.333**
**% Inhibition**	**14%**	**14%**	**85%**	**30%**	**18%**	**19%**	**91%**	**33%**	**5%**	**7%**	**44%**	**17%**	**23%**	**24%**	**39%**	**28%**

Table 17. Histopathological analysis of cartilage, bone, and synovium in Fluocinolone-treated animals in MIA Trial 2.

Group 5 Fluocinolone 0.01	Mean of All Cartilage	Matrix Damage Score	Bone Resorption						Subchondral Bone Sclerosis Score/Surface	Osteophyte		Total Joint Score		Synovitis Comments
				Percent of Surface										
			Score	MTP	MFC	LTP	LFC	Mean		Score	Measure µm	with Matrix	w/o Matrix	
3	14%	1	1	15%	0%	5%	0%	5%	0	0	0	2	1	Calcified cartilage retention GP Normal synovium
46	79%	4	3	15%	20%	75%	100%	53%	0	3	600	10	6	Mild fibrosis
48	72%	4	4	90%	50%	70%	60%	68%	0	4	800	12	8	Calcified cartilage retention GP Mild fibrosis
65	74%	4	4	60%	80%	80%	100%	80%	0	5	1000	13	9	Calcified cartilage retention GP Mild fibrosis
**Mean**	**60%**	**3.3**	**3.0**	**45%**	**38%**	**58%**	**65%**	**51%**	**0.0**	**3.0**	**600.0**	**9.3**	**6.0**	
**SE**	**15%**	**0.8**	**0.7**	**18%**	**18%**	**18%**	**24%**	**16%**	**0.0**	**1.1**	**216.0**	**2.5**	**1.8**	
**t-test to grp 1**	**0.262**	**0.215**	**0.030**	**0.030**	**0.023**	**0.052**	**0.189**	**0.031**	**0.356**	**0.254**	**0.254**	**0.117**	**0.094**	
**% Inhibition**	**27%**	**28%**	**40%**	**54%**	**60%**	**43%**	**35%**	**48%**	**100%**	**33%**	**33%**	**35%**	**38%**	

6) Clonidine (0.1 mg/kg, s.c.)

Table 18. Histopathological analysis of proteoglycan and matrix loss in Clonidine-treated animals in MIA Trial 2.

Group 6 Clonidine 0.1	MTP % loss of				MFC % loss of				LTP % loss of				LFC % loss of			
	Cell	PG	Matrix	Mean	Cell	PG	Matrix	Mean	Cell	PG	Matrix	Mean	Cell	PG	Matrix	Mean
8	100%	99%	20%	73%	100%	99%	70%	90%	90%	85%	0%	58%	100%	99%	50%	83%
26	80%	75%	5%	53%	50%	45%	20%	38%	65%	60%	30%	52%	60%	55%	10%	42%
40	95%	90%	10%	65%	95%	90%	50%	78%	100%	99%	70%	90%	100%	99%	50%	83%
**Mean**	**92%**	**88%**	**12%**	**64%**	**82%**	**78%**	**47%**	**69%**	**85%**	**81%**	**33%**	**67%**	**87%**	**84%**	**37%**	**69%**
**SE**	**0.06**	**7%**	**4%**	**6%**	**16%**	**17%**	**15%**	**16%**	**10%**	**11%**	**20%**	**12%**	**13%**	**15%**	**13%**	**14%**
**t-test to grp 1**	**0.234**	**0.232**	**0.082**	**0.084**	**0.686**	**0.680**	**0.891**	**0.832**	**0.663**	**0.620**	**0.189**	**0.351**	**0.538**	**0.558**	**0.168**	**0.319**
**% Inhibition**	**7%**	**9%**	**80%**	**25%**	**8%**	**9%**	**-7%**	**5%**	**7%**	**9%**	**51%**	**19%**	**9%**	**10%**	**48%**	**20%**

.

Table 19. Histopathological analysis of cartilage, bone, and synovium in Clonidine-treated animals in MIA Trial 2

Group 6 Clonidine 0.1	Mean of All Cartilage	Matrix Damage Score	Bone Resorption						Subchondral Bone Sclerosis	Osteophyte		Total Joint Score		Synovitis Comments
				Percent of Surface										
			Score	MTP	MFC	LTP	LFC	Mean	Score/Surface	Score	Measure µm	with Matrix	w/o Matrix	
8	76%	4	3	50%	50%	0%	100%	50%	1	5	1200	13	9	Marked fibrosis
26	46%	3	3	70%	40%	60%	60%	58%	0	4	800	10	7	Calcified cartilage retention GP, medial only<br>Mild fibrosis
40	79%	4	5	100%	100%	100%	100%	100%	0	3	600	12	8	Moderate fibrosis
**Mean**	**67%**	**3.7**	**3.7**	**73%**	**63%**	**53%**	**87%**	**69%**	**0.3**	**4.0**	**866.7**	**11.7**	**8.0**	
**SE**	**10%**	**0.3**	**0.7**	**15%**	**19%**	**29%**	**13%**	**16%**	**0.3**	**0.6**	**176.4**	**0.9**	**0.6**	
**t-test to grp 1**	**0.337**	**0.259**	**0.062**	**0.111**	**0.137**	**0.113**	**0.286**	**0.083**	**0.846**	**0.542**	**0.867**	**0.147**	**0.106**	
**% Inhibition**	**18%**	**19%**	**27%**	**25%**	**32%**	**47%**	**13%**	**29%**	**-33%**	**11%**	**4%**	**18%**	**18%**	

MIA Trial 3: Histopathology

Semi-quantitative Analysis of Histological Sections for MIA Trial 3

Histopathological analysis was completed by Bolder BioPATH for Trial 3 with three animals from each group treated with MIA (Figures 66-81 below). Intra-articular injection of saline alone was the vehicle control.

1) Vehicle Control (Saline, i.a.)

Table 20. Histopathological analysis of proteoglycan and matrix loss in MIA Trial 3 IA Vehicle Controls.

Group 1 Vehicle	MTP % loss of				MFC % loss of				LTP % loss of				LFC % loss of			
	Cell	PG	Matrix	Mean	Cell	PG	Matrix	Mean	Cell	PG	Matrix	Mean	Cell	PG	Matrix	Mean
3	100	100	50	83.333	100	100	50	83.333	100	100	50	83.333	100	100	50	83.333
15	0	0	0	0	0	0	0	0	0	0	0	0	0	0	0	0
27	100	75	10	61.667	90	75	10	58.333	75	50	5	43.333	5	5	0	3.3333
**Mean**	**66.7**	**58.3**	**20.0**	**48.3**	**63.3**	**58.3**	**20.0**	**47.2**	**58.3**	**50.0**	**18.3**	**42.2**	**35.0**	**35.0**	**16.7**	**28.9**
**SE**	**33.3**	**30.0**	**15.3**	**25.0**	**31.8**	**30.0**	**15.3**	**24.7**	**30.0**	**28.9**	**15.9**	**24.1**	**32.5**	**32.5**	**16.7**	**27.2**
**%**	**0%**	**0%**	**0%**	**0%**	**0%**	**0%**	**0%**	**0%**	**0%**	**0%**	**0%**	**0%**	**0%**	**0%**	**0%**	**0%**
**t-test**	**1.000**	**1.000**	**1.000**	**1.000**	**1.000**	**1.000**	**1.000**	**1.000**	**1.000**	**1.000**	**1.000**	**1.000**	**1.000**	**1.000**	**1.000**	**1.000**

Table 21. Histopathological analysis of cartilage, bone, and synovium in MIA Trial 3 IA Vehicle Controls

Group 1 Vehicle	Mean of All Cartilage	Matrix Damage Score	Bone Resorption Score	Bone Resorption Percent MTP	MFC	LTP	LFC	Mean	Subchond Bone Sclerosis	Osteophyte Score	Osteophyte Measure µm	Total Joint Score with Matrix	Total Joint Score w/o Matrix	Synovitis
3	83.3	5	**4**	75%	70%	90%	100%	84%	0	**4**	880 (MFC)	13	8	Mild subacute
15	0.0	0	**0**	0%	0%	0%	0%	0%	0	**0**	0	0	0	Minimal subacute
27	41.7	3	**1**	10%	5%	25%	0%	10%	0	**0**	0	4	1	Mild subacute
**Mean**	**41.7**	**2.7**	**1.7**	**28%**	**25%**	**38%**	**33%**	**31%**	**0.0**	**1.3**	**293.3**	**5.7**	**3.0**	
**SE**	**24.1**	**1.5**	**1.2**	**0.2**	**0.2**	**0.3**	**0.3**	**26%**	**0.0**	**1.3**	**293.3**	**3.8**	**2.5**	
**%**	**0%**	**0%**	**0%**	**0%**	**0%**	**0%**	**0%**	**0%**	**#DIV/0!**	**0%**	**0%**	**0%**	**0%**	
**t-test**	**1.000**	**1.000**	**1.000**	**1.000**	**1.000**	**1.000**	**1.000**	**1.000**	**#DIV/0!**	**1.000**	**1.000**	**1.000**	**1.000**	

2) IA Clonidine (4.5 µg)

Animals treated with intra-articular Clonidine (4.5 ug) were not significantly different from the vehicle controls. Lesion severity was increased compared to the vehicle controls, with a total joint score of 8.7. All animals had minimal to mild subacute synovitis.

Table 22. Histopathological analysis of proteoglycan and matrix loss in MIA Trial 3 IA Clonidine-treated animals.

Group 2 Cmpd A	MTP % loss of Cell	PG	Matrix	Mean	MFC % loss of Cell	PG	Matrix	Mean	LTP % loss of Cell	PG	Matrix	Mean	LFC % loss of Cell	PG	Matrix	Mean
8	60	40	10	36.667	0	0	0	0	0	0	0	0	0	0	0	0
10	100	100	25	75	100	100	25	75	100	75	5	60	90	75	0	55
20	100	100	10	70	100	90	10	66.667	100	75	20	65	100	100	10	70
**Mean**	**86.7**	**80.0**	**15.0**	**60.6**	**66.7**	**63.3**	**11.7**	**47.2**	**66.7**	**50.0**	**8.3**	**41.7**	**63.3**	**58.3**	**3.3**	**41.7**
**SE**	**13.3**	**20.0**	**5.0**	**12.0**	**33.3**	**31.8**	**7.3**	**23.7**	**33.3**	**25.0**	**6.0**	**20.9**	**31.8**	**30.0**	**3.3**	**21.3**
**%**	**-30%**	**-37%**	**25%**	**-25%**	**-5%**	**-9%**	**42%**	**0%**	**-14%**	**0%**	**55%**	**1%**	**-81%**	**-67%**	**80%**	**-44%**
**t-test**	**0.607**	**0.581**	**0.771**	**0.682**	**0.946**	**0.915**	**0.648**	**1.000**	**0.862**	**1.000**	**0.588**	**0.987**	**0.567**	**0.626**	**0.477**	**0.730**

Table 23. Histopathological analysis of cartilage, bone and synovium in MIA Trial 3 IA Clonidine-treated animals.

Group 2	Mean of	Matrix	Bone Resorption						Subchond	Osteophyte		Total Joint Score		Synovitis
Cmpd A	All	Damage		Percent					Bone		Measure	with	w/o	
	Cartilage	Score	Score	MTP	MFC	LTP	LFC	Mean	Sclerosis	Score	µm	Matrix	Matrix	
8	9.2	1	**1**	40%	0%	0%	0%	10%	0	**0**	0	2	1	Minimal subacute
10	66.3	4	**4**	70%	90%	92%	67%	80%	0	**4**	880 (MTP)	12	8	Mild subacute
20	67.9	4	**4**	85%	50%	92%	85%	78%	0	**4**	800 (MTP)	12	8	Mild subacute
**Mean**	**47.8**	**3.0**	**3.0**	**65%**	**47%**	**61%**	**51%**	**56%**	**0.0**	**2.7**	**560.0**	**8.7**	**5.7**	
**SE**	**19.3**	**1.0**	**1.0**	**0.1**	**0.3**	**0.3**	**0.3**	**23%**	**0.0**	**1.3**	**281.0**	**3.3**	**2.3**	
**%**	**-15%**	**-13%**	**-80%**	**-129%**	**-87%**	**-60%**	**-52%**	**-79%**	**#DIV/0!**	**-100%**	**-91%**	**-53%**	**-89%**	
**t-test**	**0.853**	**0.859**	**0.442**	**0.246**	**0.563**	**0.603**	**0.702**	**0.520**	**#DIV/0!**	**0.519**	**0.547**	**0.587**	**0.481**	

3) IA Tacrolimus (30 ng)

Animals treated with intra-articular Tacrolimus (30 ng) had significantly increased matrix loss in the MTP and LTP. Bone resorption was also significantly increased in the MTP and MFC, which resulted in a significant increase in the overall bone resorption score. All animals had subacute synovitis (mild to marked) with fibrosis. Significant t values are highlighted in red.

Table 24. Histopathological analysis of proteoglycan and matrix in MIA Trial 3 IA Tacrolimus-treated animals.

Group 3	MTP				MFC				LTP				LFC			
Cmpd B	% loss of				% loss of				% loss of				% loss of			
	Cell	PG	Matrix	Mean	Cell	PG	Matrix	Mean	Cell	PG	Matrix	Mean	Cell	PG	Matrix	Mean
13	100	100	75	91.667	100	100	50	83.333	100	100	75	91.667	100	100	100	100
17	100	100	100	100	100	100	100	100	100	100	100	100	100	100	100	100
23	100	100	90	96.667	100	100	25	75	100	100	75	91.667	100	100	40	80
**Mean**	**100.0**	**100.0**	**88.3**	**96.1**	**100.0**	**100.0**	**58.3**	**86.1**	**100.0**	**100.0**	**83.3**	**94.4**	**100.0**	**100.0**	**80.0**	**93.3**
**SE**	**0.0**	**0.0**	**7.3**	**2.4**	**0.0**	**0.0**	**22.0**	**7.3**	**0.0**	**0.0**	**8.3**	**2.8**	**0.0**	**0.0**	**20.0**	**6.7**
**%**	**-50%**	**-71%**	**-342%**	**-99%**	**-58%**	**-71%**	**-192%**	**-82%**	**-71%**	**-100%**	**-355%**	**-124%**	**-186%**	**-186%**	**-380%**	**-223%**
**t-test**	**0.374**	**0.238**	**0.016**	**0.129**	**0.313**	**0.238**	**0.226**	**0.206**	**0.238**	**0.158**	**0.022**	**0.097**	**0.116**	**0.116**	**0.072**	**0.083**

Table 25. Histopathology of cartilage, bone and synovium in MIA Trial 3 IA Tacrolimus-treated animals.

Group 3 Cmpd B	Mean of All Cartilage	Matrix Damage Score	Bone Resorption Score	Bone Resorption Percent MTP	MFC	LTP	LFC	Mean	Subchond Bone Sclerosis	Osteophyte Score	Osteophyte Measure µm	Total Joint Score with Matrix	Total Joint Score w/o Matrix	Synovitis
13	91.7	5	**5**	100%	100%	100%	100%	100%	0	**5**	1000 (MFC)	15	10	Marked subacute with fibrosis
17	100.0	5	**5**	100%	100%	100%	100%	100%	0	**0**	0	10	5	Mild subacute with fibrosis
23	85.8	5	**5**	100%	100%	100%	100%	100%	0	**5**	1000 (MTP)	15	10	Mild subacute with fibrosis
**Mean**	**92.5**	**5.0**	**5.0**	**100%**	**100%**	**100%**	**100%**	**100%**	**0.0**	**3.3**	**666.7**	**13.3**	**8.3**	
**SE**	**4.1**	**0.0**	**0.0**	**0.0**	**0.0**	**0.0**	**0.0**	**0%**	**0.0**	**1.7**	**333.3**	**1.7**	**1.7**	
**%**	**-122%**	**-88%**	**-200%**	**-253%**	**-300%**	**-161%**	**-200%**	**-220%**	**#DIV/0!**	**-150%**	**-127%**	**-135%**	**-178%**	
**t-test**	**0.106**	**0.184**	**0.050**	**0.038**	**0.029**	**0.083**	**0.116**	**0.060**	**#DIV/0!**	**0.402**	**0.448**	**0.141**	**0.152**	

4) IA Fluocinolone (15 ng)

Animals treated with intra-articular Fluocinolone (15 ng) were not significantly different from the vehicle controls. Lesion severity was increased compared to the vehicle controls, with a total joint score of 10.3. All animals had minimal to mild subacute synovitis.

Table 26. Histopathology of proteoglycan and matrix loss in MIA Trial 3 IA Fluocinolone-treated animals.

Group 4 Cmpd C	MTP % loss of Cell	PG	Matrix	Mean	MFC % loss of Cell	PG	Matrix	Mean	LTP % loss of Cell	PG	Matrix	Mean	LFC % loss of Cell	PG	Matrix	Mean
25	100	100	40	80	100	100	10	70	100	75	25	66.667	100	100	25	75
28	100	100	25	75	100	100	40	80	100	100	100	100	100	100	90	96.667
39	2	0	0	0.6667	0	0	0	0	0	0	0	0	2	0	0	0.6667
**Mean**	**67.3**	**66.7**	**21.7**	**51.9**	**66.7**	**66.7**	**16.7**	**50.0**	**66.7**	**58.3**	**41.7**	**55.6**	**67.3**	**66.7**	**38.3**	**57.4**
**SE**	**32.7**	**33.3**	**11.7**	**25.7**	**33.3**	**33.3**	**12.0**	**25.2**	**33.3**	**30.0**	**30.0**	**29.4**	**32.7**	**33.3**	**26.8**	**29.1**
**%**	**-1%**	**-14%**	**-8%**	**-7%**	**-5%**	**-14%**	**17%**	**-6%**	**-14%**	**-17%**	**-127%**	**-32%**	**-92%**	**-90%**	**-130%**	**-99%**
**t-test**	**0.989**	**0.862**	**0.935**	**0.926**	**0.946**	**0.862**	**0.872**	**0.941**	**0.862**	**0.851**	**0.530**	**0.743**	**0.522**	**0.534**	**0.530**	**0.513**

Table 27. Histopathology of cartilage, bone, & synovium in MIA Trial 3 IA Fluocinolone-treated animals.

Group 4 Cmpd C	Mean of All Cartilage	Matrix Damage Score	Bone Resorption Score	Bone Resorption Percent MTP	MFC	LTP	LFC	Mean	Subchond Bone Sclerosis	Osteophyte Score	Osteophyte Measure µm	Total Joint Score with Matrix	Total Joint Score w/o Matrix	Synovitis
25	72.9	4	**5**	100%	90%	100%	100%	98%	0	**4**	880 (MTP)	13	9	Mild subacute
28	87.9	5	**5**	100%	100%	100%	100%	100%	0	**3**	640 (MTP)	13	8	Mild subacute
39	0.3	1	**1**	1%	25%	0%	0%	7%	0	**3**	600 (MFC)	5	4	Minimal subacute
**Mean**	**53.7**	**3.3**	**3.7**	**67%**	**72%**	**67%**	**67%**	**68%**	**0.0**	**3.3**	**706.7**	**10.3**	**7.0**	
**SE**	**27.0**	**1.2**	**1.3**	**33%**	**24%**	**33%**	**33%**	**31%**	**0.0**	**0.3**	**87.4**	**2.7**	**1.5**	
**%**	**-29%**	**-25%**	**-120%**	**-136%**	**-187%**	**-74%**	**-100%**	**-118%**	**#DIV/0!**	**-150%**	**-141%**	**-82%**	**-133%**	
**t-test**	**0.756**	**0.742**	**0.328**	**0.394**	**0.225**	**0.544**	**0.519**	**0.416**	**#DIV/0!**	**0.219**	**0.248**	**0.375**	**0.246**	

5) IA Meloxicam (100 µg)

Animals treated with intra-articular Meloxicam (100 µg) were not significantly different from the vehicle controls. Lesion severity was decreased compared to the vehicle controls, with a total joint score of 3 (47% decrease). All animals had minimal to moderate subacute synovitis.

Table 28. Histopathology of proteoglycan and matrix loss in MIA Trial 3 IA Meloxicam-treated animals.

Group 5 Cmpd D	MTP % loss of Cell	MTP PG	MTP Matrix	MTP Mean	MFC % loss of Cell	MFC PG	MFC Matrix	MFC Mean	LTP % loss of Cell	LTP PG	LTP Matrix	LTP Mean	LFC % loss of Cell	LFC PG	LFC Matrix	LFC Mean
21	2	1	0	1	0	0	0	0	0	0	0	0	0	0	0	0
30	70	60	5	45	40	12.5	0	17.5	0	0	0	0	0	0	0	0
42	80	50	5	45	20	5	0	8.3333	0	0	0	0	0	0	0	0
**Mean**	**50.7**	**37.0**	**3.3**	**30.3**	**20.0**	**5.8**	**0.0**	**8.6**	**0.0**	**0.0**	**0.0**	**0.0**	**0.0**	**0.0**	**0.0**	**0.0**
**SE**	**24.5**	**18.2**	**1.7**	**14.7**	**11.5**	**3.6**	**0.0**	**5.1**	**0.0**	**0.0**	**0.0**	**0.0**	**0.0**	**0.0**	**0.0**	**0.0**
**%**	**24%**	**37%**	**83%**	**37%**	**68%**	**90%**	**100%**	**82%**	**100%**	**100%**	**100%**	**100%**	**100%**	**100%**	**100%**	**100%**
**t-test**	**0.719**	**0.577**	**0.339**	**0.568**	**0.269**	**0.158**	**0.261**	**0.200**	**0.124**	**0.158**	**0.313**	**0.154**	**0.343**	**0.343**	**0.374**	**0.349**

Table 29. Histopathology of cartilage, bone, and synovium in MIA Trial 3 IA Meloxicam-treated animals.

Group 5 Cmpd D	Mean of All Cartilage	Matrix Damage Score	Bone Resorption Score	Bone Resorption Percent MTP	MFC	LTP	LFC	Mean	Subchond Bone Sclerosis	Osteophyte Score	Osteophyte Measure µm	Total Joint Score with Matrix	Total Joint Score w/o Matrix	Synovitis
21	0.3	1	**0**	0%	0%	0%	0%	0%	0	**0**	0	1	0	Minimal subacute
30	15.6	1	**2**	25%	25%	0%	0%	13%	0	**0**	0	3	2	Moderate subacute
42	13.3	1	**1**	20%	20%	0%	0%	10%	1 (MTP)	**2**	440 (MFC)	5	4	Minimal subacute
**Mean**	**9.7**	**1.0**	**1.0**	**15%**	**15%**	**0%**	**0%**	**8%**	**0.3**	**0.7**	**146.7**	**3.0**	**2.0**	
**SE**	**4.8**	**0.0**	**0.6**	**8%**	**8%**	**0%**	**0%**	**4%**	**0.3**	**0.7**	**146.7**	**1.2**	**1.2**	
**%**	**77%**	**63%**	**40%**	**47%**	**40%**	**100%**	**100%**	**76%**	**#DIV/0!**	**50%**	**50%**	**47%**	**33%**	
**t-test**	**0.263**	**0.315**	**0.643**	**0.618**	**0.696**	**0.226**	**0.374**	**0.424**	**0.374**	**0.678**	**0.678**	**0.543**	**0.736**	

6) IA Tranilast (500 ng)

Animals treated with intra-articular Tranilast (500 ng) had significantly increased matrix loss in the MTP. Bone resorption was also significantly increased in the MTP and MFC, which resulted in a significant increase in the overall bone resorption score. All animals had mild subacute synovitis with fibrosis.

Table 30. Histopathological analysis of proteoglycan and matrix loss in MIA Trial 3 IA Tranilast-treated animals.

Group 6	MTP				MFC				LTP				LFC			
Cmpd E	% loss of				% loss of				% loss of				% loss of			
	Cell	PG	Matrix	Mean	Cell	PG	Matrix	Mean	Cell	PG	Matrix	Mean	Cell	PG	Matrix	Mean
7	100	100	90	96.667	100	100	40	80	100	100	50	83.333	100	100	40	80
11	100	100	90	96.667	100	100	80	93.333	100	100	75	91.667	100	100	75	91.667
22	100	100	60	86.667	100	100	80	93.333	100	100	75	91.667	100	100	75	91.667
Mean	100.0	100.0	80.0	93.3	100.0	100.0	66.7	88.9	100.0	100.0	66.7	88.9	100.0	100.0	63.3	87.8
SE	0.0	0.0	10.0	3.3	0.0	0.0	13.3	4.4	0.0	0.0	8.3	2.8	0.0	0.0	11.7	3.9
%	-50%	-71%	-300%	-93%	-58%	-71%	-233%	-88%	-71%	-100%	-264%	-111%	-186%	-186%	-280%	-204%
t-test	0.374	0.238	0.030	0.149	0.313	0.238	0.083	0.172	0.238	0.158	0.055	0.126	0.116	0.116	0.084	0.099

Table 31. Histopathological analysis of cartilage, bone, and synovium in MIA Trial 3 IA Tranilast-treated animals.

Group 6	Mean of	Matrix	Bone Resorption						Subchond	Osteophyte		Total Joint Score		Synovitis
Cmpd E	All	Damage		Percent					Bone		Measure	with	w/ o	
	Cartilage	Score	Score	MTP	MFC	LTP	LFC	Mean	Sclerosis	Score	μm	Matrix	Matrix	
7	85.0	5	**5**	100%	100%	100%	100%	100%	0	**5**	1000 (MTP)	15	10	Mild subacute with fibrosis
11	93.3	5	**5**	100%	100%	100%	100%	100%	0	**4**	800 (MFC)	14	9	Mild subacute with fibrosis
22	90.8	5	**5**	90%	100%	100%	100%	98%	0	**4**	880 (MFC)	14	9	Mild subacute with fibrosis
Mean	89.7	5.0	5.0	97%	100%	100%	100%	99%	0.0	4.3	893.3	14.3	9.3	
SE	2.5	0.0	0.0	3%	0%	0%	0%	1%	0.0	0.3	58.1	0.3	0.3	
%	-115%	-88%	-200%	-241%	-300%	-161%	-200%	-217%	#DIV/0!	-225%	-205%	-153%	-211%	
t-test	0.118	0.184	0.050	0.045	0.029	0.083	0.116	0.062	#DIV/0!	0.094	0.115	0.088	0.067	

7) IA Trimacinolone Hexacetonide (TH) (0.15 mg)

Animals treated with IA Trimacinolone Hexacetonide (0.15 mg) had significantly increased matrix loss in the MTP. Lesion severity was non-significantly increased, in general, with a total joint score of 8.3. All animals had subacute synovitis and hypocellular marrow. Lesion morphology suggests that this compound inhibited MIA lesions, but that the cartilage was ground away mechanically.

Table 32. Histopathology of proteoglycan and matrix in MIA Trial 3 IA TH-treated animals.

Group 7 Cmpd F	MTP % loss of				MFC % loss of				LTP % loss of				LFC % loss of			
	Cell	PG	Matrix	Mean	Cell	PG	Matrix	Mean	Cell	PG	Matrix	Mean	Cell	PG	Matrix	Mean
14	100	100	100	100	90	90	90	90	15	5	1	7	0	0	0	0
29	100	100	100	100	100	100	100	100	100	100	100	100	100	100	100	100
45	95	95	50	80	80	40	10	43.333	2	1	0	1	30	5	0	11.667
**Mean**	**98.3**	**98.3**	**83.3**	**93.3**	**90.0**	**76.7**	**66.7**	**77.8**	**39.0**	**35.3**	**33.7**	**36.0**	**43.3**	**35.0**	**33.3**	**37.2**
**SE**	**1.7**	**1.7**	**16.7**	**6.7**	**5.8**	**18.6**	**28.5**	**17.5**	**30.7**	**32.4**	**33.2**	**32.0**	**29.6**	**32.5**	**33.3**	**31.6**
**%**	**-48%**	**-69%**	**-317%**	**-93%**	**-42%**	**-31%**	**-233%**	**-65%**	**33%**	**29%**	**-84%**	**15%**	**-24%**	**0%**	**-100%**	**-29%**
**t-test**	**0.396**	**0.255**	**0.049**	**0.157**	**0.456**	**0.631**	**0.222**	**0.369**	**0.676**	**0.752**	**0.698**	**0.884**	**0.859**	**1.000**	**0.678**	**0.851**

Table 33. Histopathology of cartilage, bone, and synovium in MIA Trial 3 IA TH-treated animals.

Group 7 Cmpd F	Mean of All Cartilage	Matrix Damage Score	Bone Resorption Score	Bone Resorption Percent MTP	MFC	LTP	LFC	Mean	Subchond Bone Sclerosis	Osteophyte Score	Osteophyte Measure μm	Total Joint Score with Matrix	Total Joint Score w/o Matrix	Synovitis
14	49.3	3	**1**	0%	5%	1%	0%	2%	1	**3**	680	8	5	Minimal subacute
											(MFC)			Marrow hypocellular
29	100.0	5	**5**	90%	100%	100%	100%	98%	0	**3**	680	13	8	Severe subacute
											(MFC)			Marrow hypocellular
45	34.0	2	**1**	5%	0%	2%	5%	3%	1	**0**	0	4	2	Minimal subacute
														Marrow hypocellular
**Mean**	**61.1**	**3.3**	**2.3**	**32%**	**35%**	**34%**	**35%**	**34%**	**0.7**	**2.0**	**453.3**	**8.3**	**5.0**	
**SE**	**20.0**	**0.9**	**1.3**	**29%**	**33%**	**33%**	**33%**	**32%**	**0.3**	**1.0**	**226.7**	**2.6**	**1.7**	
**%**	**-47%**	**-25%**	**-40%**	**-12%**	**-40%**	**10%**	**-5%**	**-9%**	**#DIV/0!**	**-50%**	**-55%**	**-47%**	**-67%**	
**t-test**	**0.568**	**0.715**	**0.729**	**0.933**	**0.813**	**0.929**	**0.973**	**0.950**	**0.116**	**0.710**	**0.688**	**0.596**	**0.548**	

8) Systemic Clonidine (100 μg/kg)

Animals treated with Clonidine systemically (100 μg/kg) were not significantly different from the vehicle controls. Lesion severity was increased compared to the vehicle controls, with a total joint score of 10.7. All animals had minimal to mild subacute synovitis and one had fibrosis.

Table 34. Histopathological analysis of proteoglycan and matrix loss in MIA Trial 3 Clonidine-treated animals.

Group 8 Clonidine	MTP % loss of				MFC % loss of				LTP % loss of				LFC % loss of			
	Cell	PG	Matrix	Mean	Cell	PG	Matrix	Mean	Cell	PG	Matrix	Mean	Cell	PG	Matrix	Mean
1	100	100	50	83.333	100	75	30	68.333	100	100	75	91.667	100	75	25	66.667
12	100	100	90	96.667	100	100	75	91.667	100	100	75	91.667	100	100	75	91.667
18	65	35	5	35	10	5	0	5	60	30	5	31.667	50	25	0	25
**Mean**	**88.3**	**78.3**	**48.3**	**71.7**	**70.0**	**60.0**	**35.0**	**55.0**	**86.7**	**76.7**	**51.7**	**71.7**	**83.3**	**66.7**	**33.3**	**61.1**
**SE**	**11.7**	**21.7**	**24.6**	**18.7**	**30.0**	**28.4**	**21.8**	**25.9**	**13.3**	**23.3**	**23.3**	**20.0**	**16.7**	**22.0**	**22.0**	**19.4**
**%**	**-33%**	**-34%**	**-142%**	**-48%**	**-11%**	**-3%**	**-75%**	**-16%**	**-49%**	**-53%**	**-182%**	**-70%**	**-138%**	**-90%**	**-100%**	**-112%**
**t-test**	**0.573**	**0.618**	**0.383**	**0.496**	**0.886**	**0.970**	**0.603**	**0.839**	**0.437**	**0.512**	**0.303**	**0.400**	**0.257**	**0.466**	**0.579**	**0.390**

Table 35. Histopathological analysis of cartilage, bone, and synovium in MIA Trial 3 Clonidine-treated animals.

Group 8 Clonidine	Mean of All Cartilage	Matrix Damage Score	Bone Resorption Score	Bone Resorption Percent MTP	MFC	LTP	LFC	Mean	Subchond Bone Sclerosis	Osteophyte Score	Osteophyte Measure µm	Total Joint Score with Matrix	Total Joint Score w/o Matrix	Synovitis
1	77.5	4	**5**	100%	90%	100%	100%	98%	0	**5**	1000 (MTP)	14	10	Mild subacute with fibrosis
12	92.9	5	**5**	100%	100%	100%	100%	100%	0	**3**	680 (MFC)	13	8	Mild subacute
18	24.2	2	**3**	50%	10%	60%	15%	34%	0	**0**	0	5	3	Minimal subacute
**Mean**	**64.9**	**3.7**	**4.3**	**83%**	**67%**	**87%**	**72%**	**77%**	**0.0**	**2.7**	**560.0**	**10.7**	**7.0**	
**SE**	**20.8**	**0.9**	**0.7**	**17%**	**28%**	**13%**	**28%**	**22%**	**0.0**	**1.5**	**294.8**	**2.8**	**2.1**	
**%**	**-56%**	**-38%**	**-160%**	**-194%**	**-167%**	**-126%**	**-115%**	**-147%**	**#DIV/0!**	**-100%**	**-91%**	**-88%**	**-133%**	
**t-test**	**0.506**	**0.588**	**0.124**	**0.129**	**0.315**	**0.182**	**0.430**	**0.251**	**#DIV/0!**	**0.536**	**0.556**	**0.355**	**0.288**	

Conclusions

Results of this study indicated that Meloxicam had a somewhat beneficial effect on lesions of MIA-induced arthritis, but the effect was not statistically significant. All other treatments increased lesion severity; however, this was likely a result of lesion variability in the control group in conjunction with the extremely small group size. One control rat had no cartilage lesions (potentially due to mistargeting of the MIA), one was moderate, and one was severe. Variability was evident for all groups except those treated with Tacrolimus (all severe), Meloxicam (all less severe), and Tranilast (all severe). Treatment with Trimacinolone consistently resulted in marrow hypocellularity and lesions that were more suggestive of mechanical cartilage abrasion than purely MIA induced lesions, suggesting the animals continued to use their joint in spite of the injury. These results should be confirmed with histopathological analysis of larger group sizes.

MIA Trial 3: Representative Images

Figure 55. Three representative images from each group from MIA Trial 3. Group 1 was treated with intra-articular (IA) saline (vehicle), group 2 with IA clonidine (4.5 µg), group 3 with IA Tacrolimus (30 ng), group 4 with IA Fluocinolone (15 ng), group 5 with Meloxicam (100 µg), group 6 with Tranilast (500 ng), group 7 with Triamcinolone hexacetonide (150 µg), and group 8 with systemic clonidine (100 µg/kg). Note that in the manuscript group 8 was moved into the second position in Table 3 MIA Trial 3. Representative images from this figure are also found in the manuscript (Figure 4).

1. Grp 1, Vehicle, An 3, 16x.

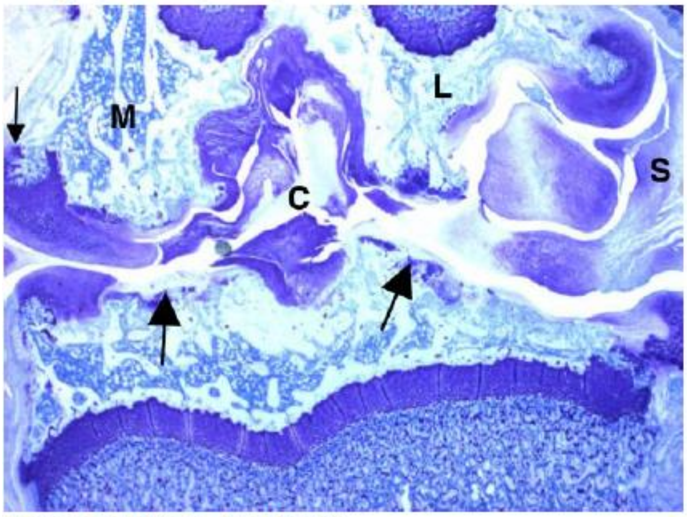

1. Knee from a vehicle treated animal has severe loss of cartilage on all four surfaces, mild subacute synovitis, and a large osteophyte. M and L identify medial and lateral aspects of the joint. C identifies cruciates. S identifies synovium. Large arrows identify representative affected cartilage surfaces; small arrow identifies osteophyte.

2. Grp 1, Vehicle, An 15, 16x.

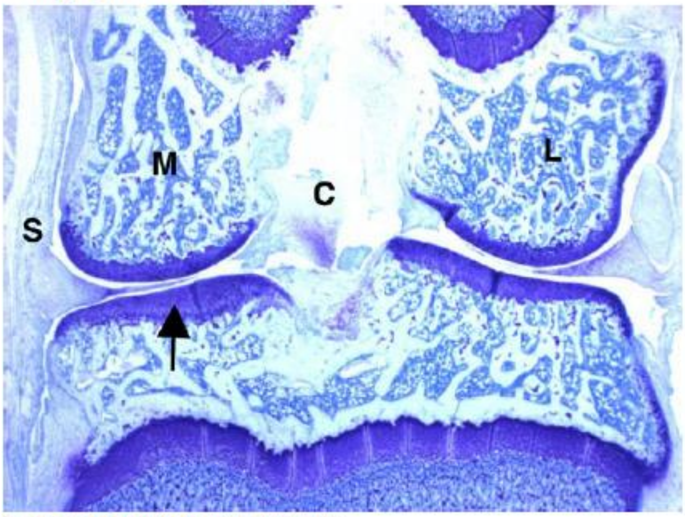

2. Knee from a vehicle treated animal has minimal subacute synovitis with no cartilage loss. M and L identify medial and lateral aspects of the joint. C identifies cruciates. S identifies synovium. Large arrow identifies normal cartilage.

3. Grp 1, Vehicle, An 27, 16x.

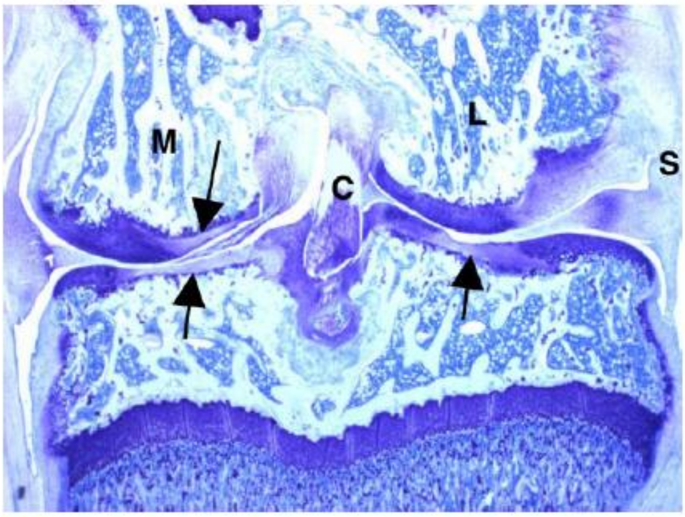

3. Knee from a vehicle treated animal has loss of cartilage on all four surfaces and mild subacute synovitis. M and L identify medial and lateral aspects of the joint. C identifies cruciates. S identifies synovium. Large arrows identify representative affected cartilage surfaces.

1. Grp 2, Cmpd A, An 8, 16x.

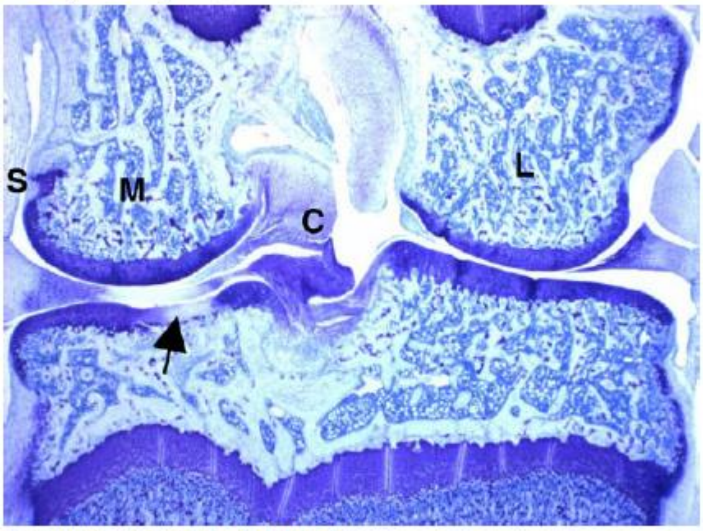

1. Knee from an animal treated with compound A has mild loss of cartilage on the medial tibial plateau and minimal subacute synovitis. M and L identify medial and lateral aspects of the joint. C identifies cruciates. S identifies synovium. Large arrow identifies representative affected cartilage surface.

2. Grp 2, Cmpd A, An 10, 16x.

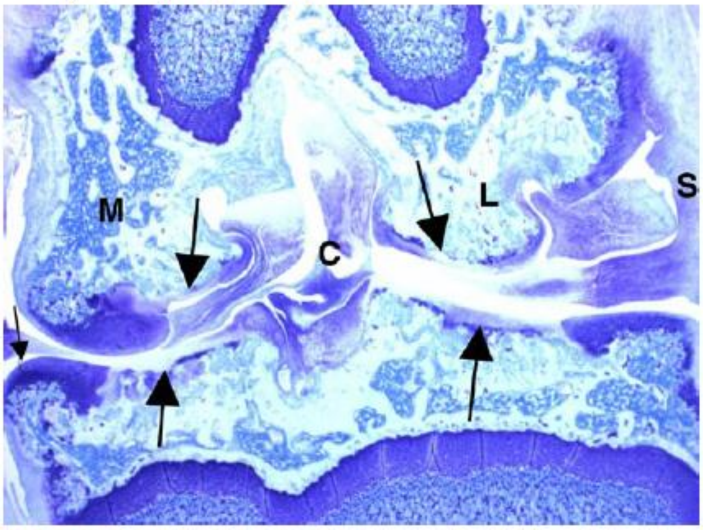

2. Knee from an animal treated with compound A has severe loss of cartilage on all four surfaces, mild subacute synovitis, and a large osteophyte. M and L identify medial and lateral aspects of the joint. C identifies cruciates. S identifies synovium. Large arrows identify representative affected cartilage surfaces; small arrow identifies osteophyte.

3. Grp 2, Cmpd A, An 20, 16x.

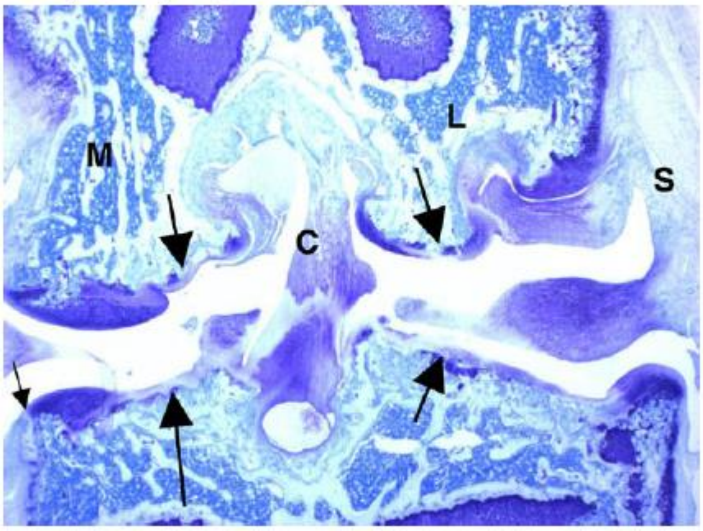

3. Knee from an animal treated with compound A has severe loss of cartilage on all four surfaces, mild subacute synovitis, and a large osteophyte. M and L identify medial and lateral aspects of the joint. C identifies cruciates. S identifies synovium. Large arrows identify representative affected cartilage surfaces; small arrow identifies osteophyte.

1. Grp 3, Cmpd B, An 13, 16x.

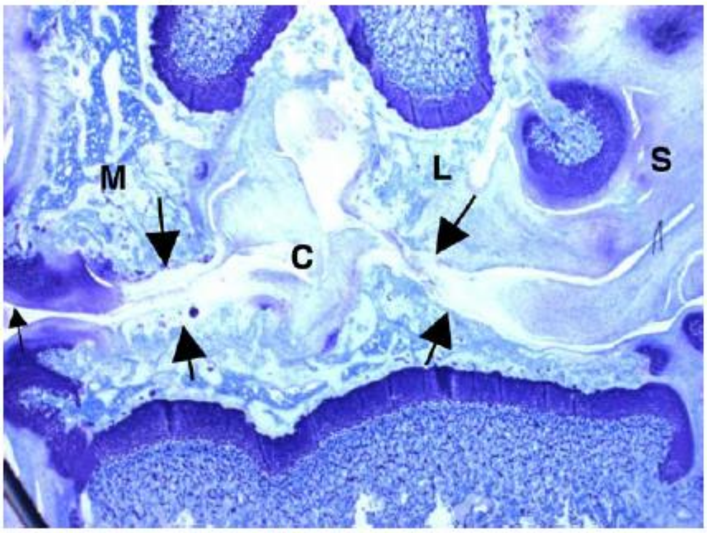

1. Knee from an animal treated with compound B has severe loss of cartilage on all four surfaces, marked subacute synovitis with fibrosis, and a very large osteophyte. M and L identify medial and lateral aspects of the joint. C identifies cruciates. S identifies synovium. Large arrows identify representative affected cartilage surfaces; small arrow identifies osteophyte.

2. Grp 3, Cmpd B, An 17, 16x.

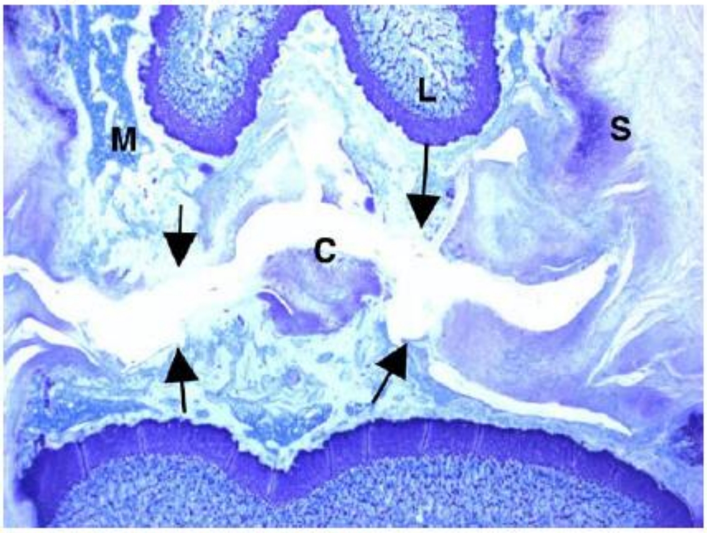

2. Knee from an animal treated with compound B has severe loss of cartilage on all four surfaces and mild subacute synovitis with fibrosis. M and L identify medial and lateral aspects of the joint. C identifies cruciates. S identifies synovium. Large arrows identify representative affected cartilage surfaces.

3. Grp 3, Cmpd B, An 23, 16x.

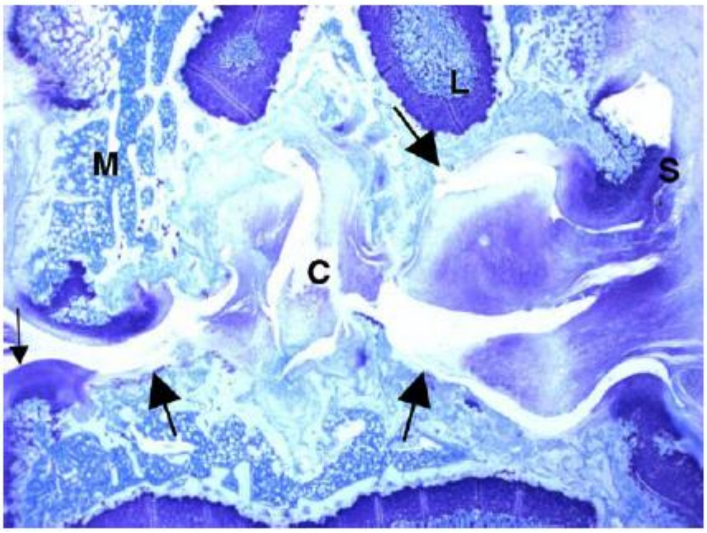

3. Knee from an animal treated with compound B has severe loss of cartilage on all four surfaces, mild subacute synovitis with fibrosis, and a very large osteophyte. M and L identify medial and lateral aspects of the joint. C identifies cruciates. S identifies synovium. Large arrows identify representative affected cartilage surfaces; small arrow identifies osteophyte.

1. Grp 4, Cmpd C, An 25, 16x.

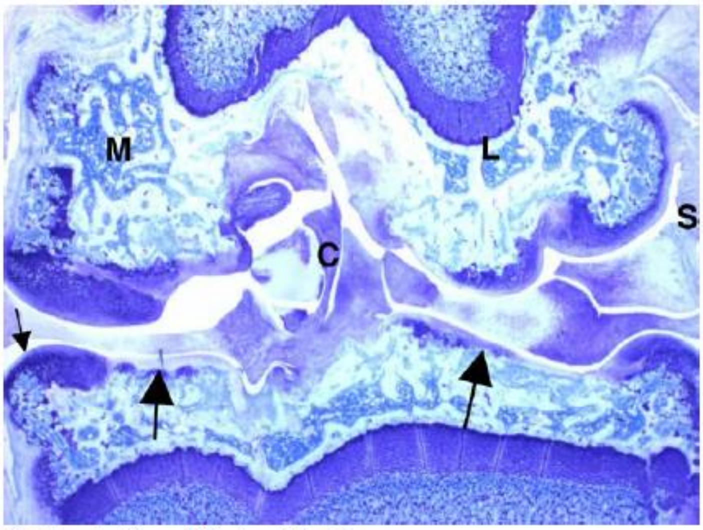

1. Knee from an animal treated with compound C has severe loss of cartilage on all four surfaces, mild subacute synovitis, and a large osteophyte. M and L identify medial and lateral aspects of the joint. C identifies cruciates. S identifies synovium. Large arrows identify representative affected cartilage surfaces; small arrow identifies osteophyte.

2. Grp 4, Cmpd C, An 28, 16x.

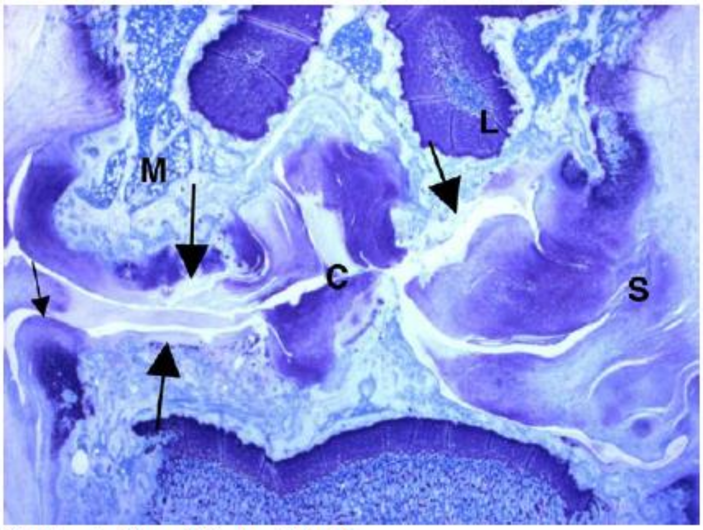

2. Knee from an animal treated with compound C has severe loss of cartilage on all four surfaces, mild subacute synovitis, and a large osteophyte. M and L identify medial and lateral aspects of the joint. C identifies cruciates. S identifies synovium. Large arrows identify representative affected cartilage surfaces; small arrow identifies osteophyte.

3. Grp 4, Cmpd C, An 39, 16x.

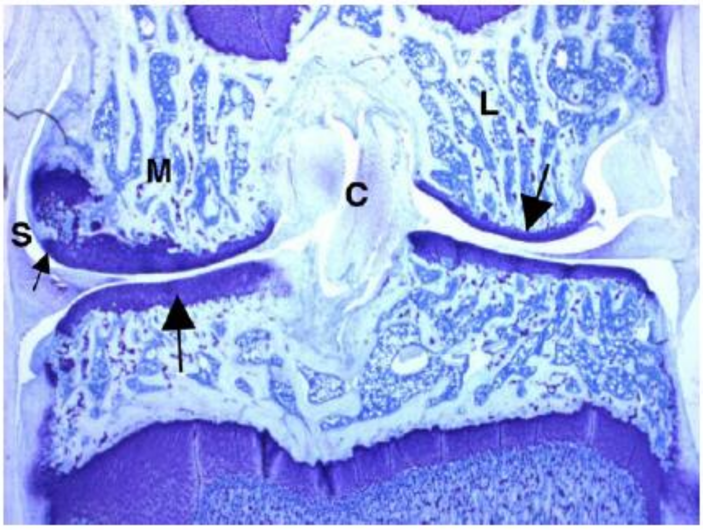

3. Knee from an animal treated with compound C has very slight loss of cartilage on the medial tibial plateau and lateral femoral condyle, minimal subacute synovitis, and a large osteophyte. M and L identify medial and lateral aspects of the joint. C identifies cruciates. S identifies synovium. Large arrows identify representative affected cartilage surfaces; small arrow identifies osteophyte.

1. Grp 5, Cmpd D, An 21, 16x.

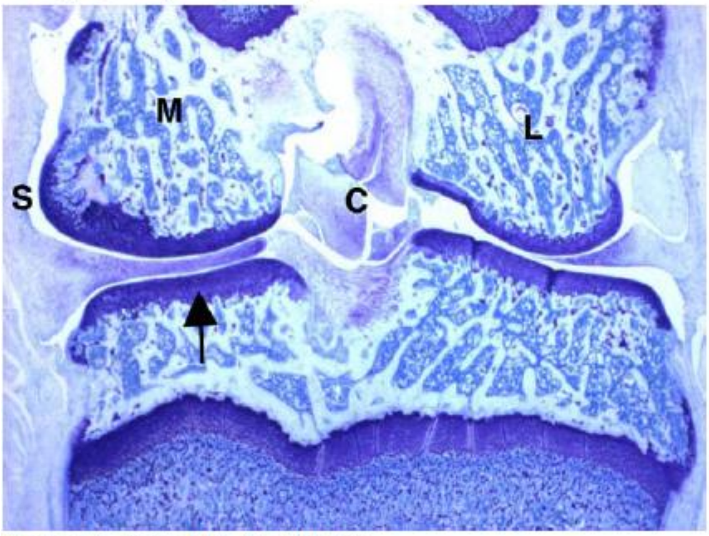

1. Knee from an animal treated with compound D has very slight loss of cartilage on the medial tibial plateau and minimal subacute synovitis. M and L identify medial and lateral aspects of the joint. C identifies cruciates. S identifies synovium. Large arrow identifies representative affected cartilage surface.

2. Grp 5, Cmpd D, An 30, 16x.

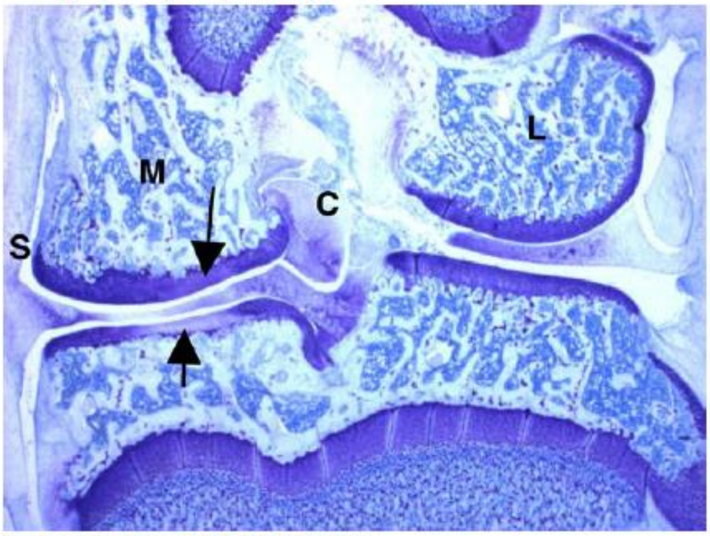

2. Knee from an animal treated with compound D has very slight loss of cartilage on the medial tibial plateau and medial femoral condyle with moderate subacute synovitis. M and L identify medial and lateral aspects of the joint. C identifies cruciates. S identifies synovium. Large arrow identifies representative affected cartilage surface.

3. Grp 5, Cmpd D, An 42, 16x.

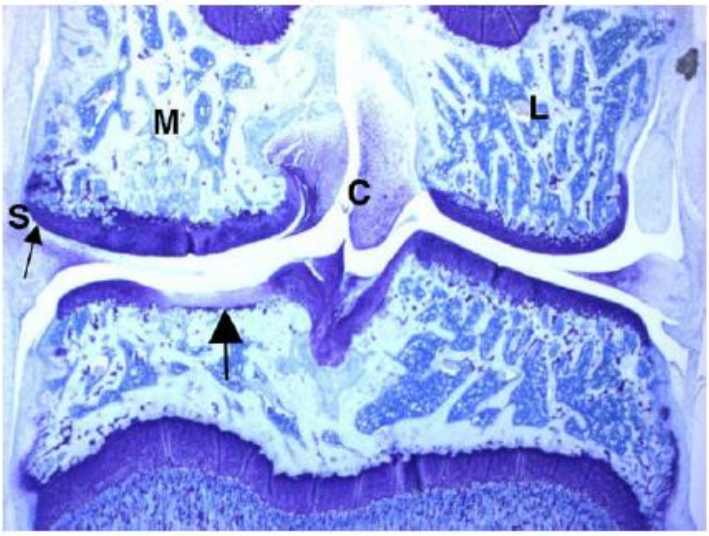

3. Knee from an animal treated with compound D has loss of cartilage on the medial tibial plateau and medial femoral condyle, minimal subacute synovitis, and a medium osteophyte. M and L identify medial and lateral aspects of the joint. C identifies cruciates. S identifies synovium. Large arrow identifies representative affected cartilage surface; small arrow identifies osteophyte.

1. Grp 6, Cmpd E, An 7, 16x.

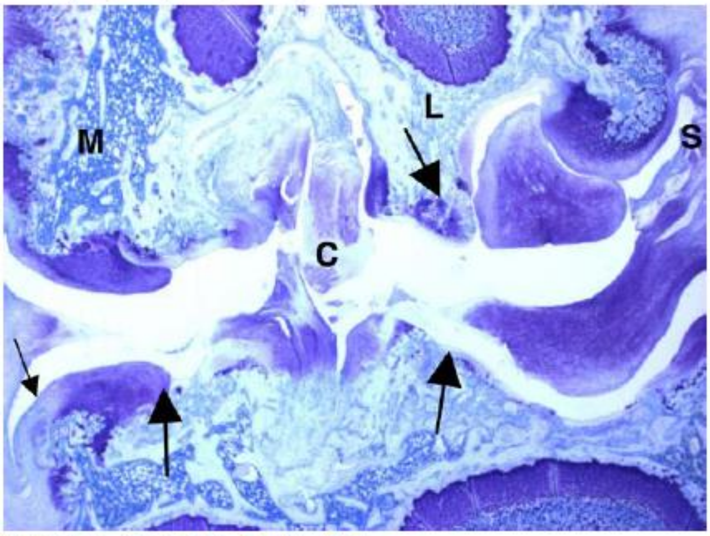

1. Knee from an animal treated with compound E has severe loss of cartilage on all four surfaces, mild subacute synovitis with fibrosis, and a very large osteophyte. M and L identify medial and lateral aspects of the joint. C identifies cruciates. S identifies synovium. Large arrows identify representative affected cartilage surfaces; small arrow identifies osteophyte.

2. Grp 6, Cmpd E, An 11, 16x.

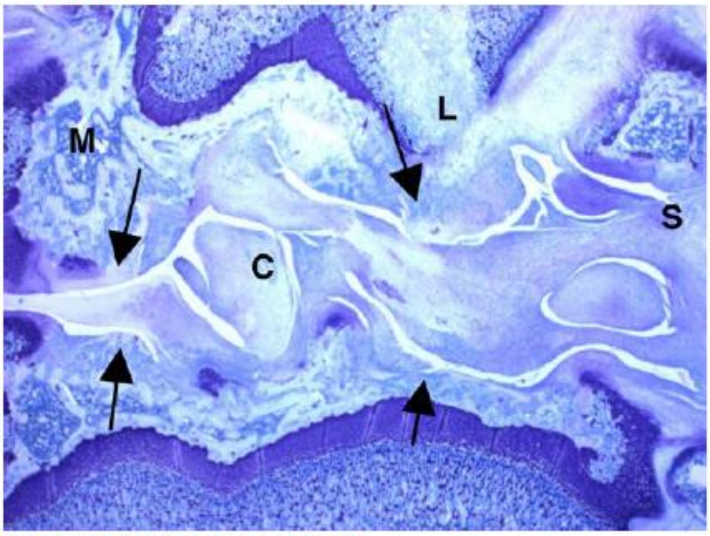

2. Knee from an animal treated with compound E has severe loss of cartilage on all four surfaces, mild subacute synovitis with fibrosis, and a large osteophyte. M and L identify medial and lateral aspects of the joint. C identifies cruciates. S identifies synovium. Large arrows identify representative affected cartilage surfaces.

3. Grp 6, Cmpd E, An 22, 16x.

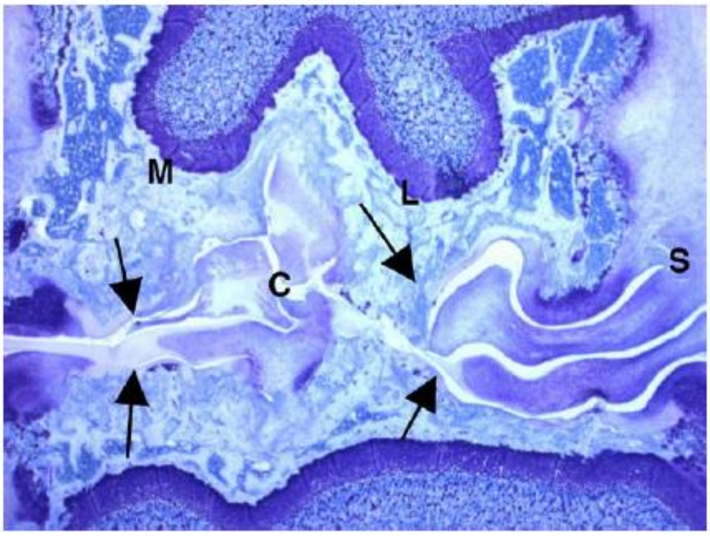

3. Knee from an animal treated with compound E has severe loss of cartilage on all four surfaces, mild subacute synovitis with fibrosis, and a large osteophyte. M and L identify medial and lateral aspects of the joint. C identifies cruciates. S identifies synovium. Large arrows identify representative affected cartilage surfaces.

1. Grp 7, Cmpd F, An 14, 16x.

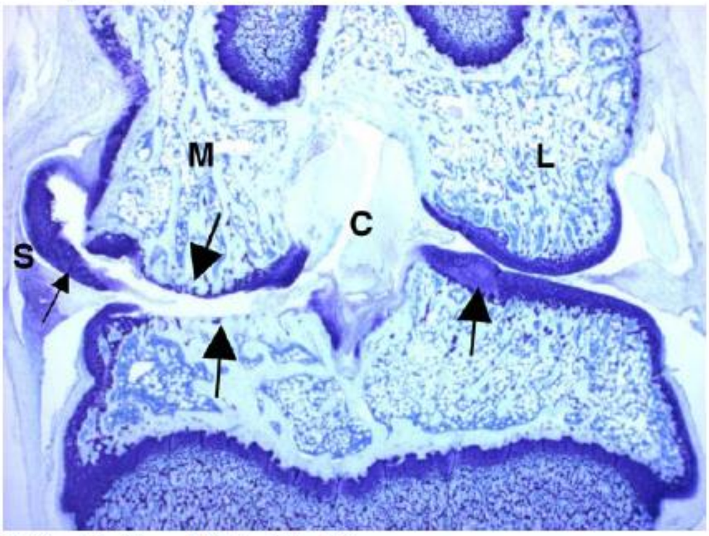

1. Knee from an animal treated with compound F has loss of cartilage on three surfaces, minimal subacute synovitis, and a large osteophyte. M and L identify medial and lateral aspects of the joint. C identifies cruciates. S identifies synovium. Large arrows identify representative affected cartilage surfaces; small arrow identifies osteophyte.

2. Grp 7, Cmpd F, An 29, 16x.

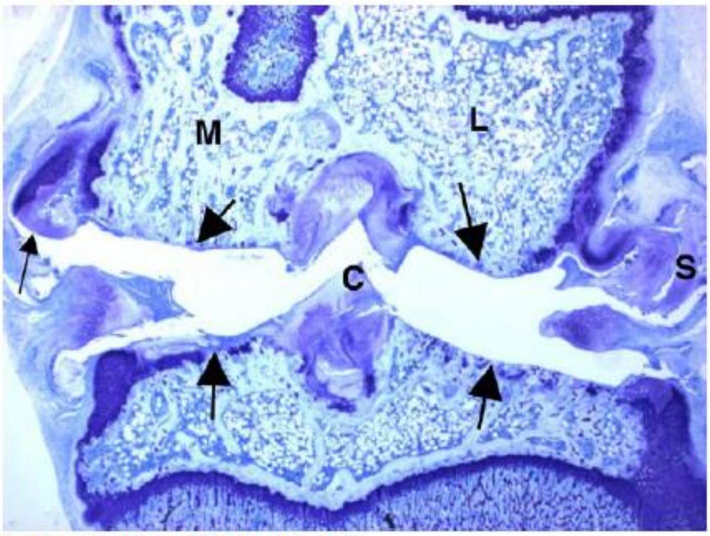

2. Knee from an animal treated with compound F has total loss of cartilage on all four surfaces, severe subacute synovitis, and a large osteophyte. M and L identify medial and lateral aspects of the joint. C identifies cruciates. S identifies synovium. Large arrows identify representative affected cartilage surfaces; small arrow identifies osteophyte.

3. Grp 7, Cmpd F, An 45, 16x.

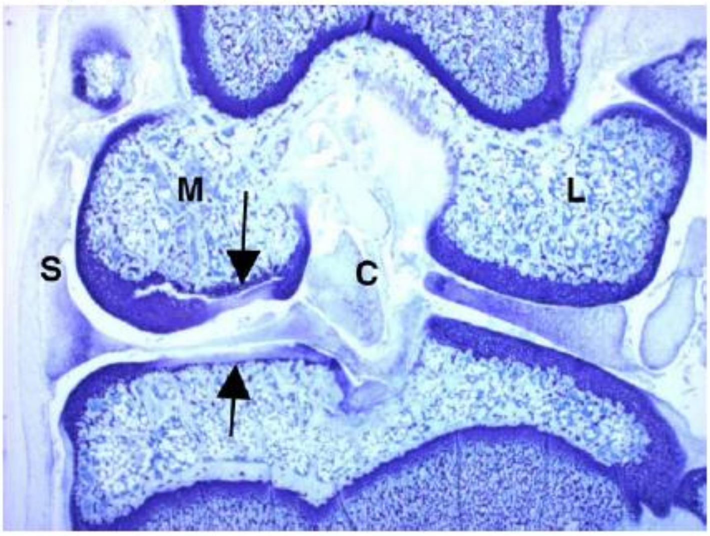

3. Knee from an animal treated with compound F has loss of cartilage on all four surfaces and minimal subacute synovitis. M and L identify medial and lateral aspects of the joint. C identifies cruciates. S identifies synovium. Large arrows identify representative affected cartilage surfaces.

1. Grp 8, Clonidine, An 1, 16x.

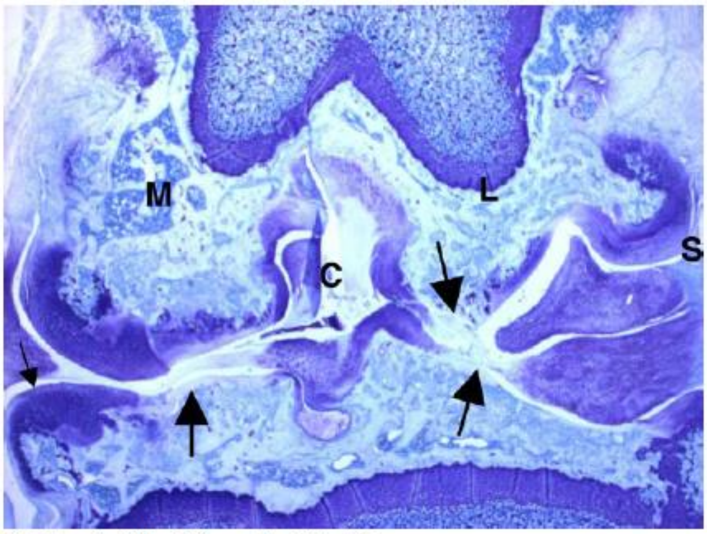

1. Knee from an animal treated with Clonidine has loss of cartilage on all four surfaces, mild subacute synovitis with fibrosis, and a very large osteophyte. M and L identify medial and lateral aspects of the joint. C identifies cruciates. S identifies synovium. Large arrows identify representative affected cartilage surfaces; small arrow identifies osteophyte.

2. Grp 8, Clonidine, An 12, 16x.

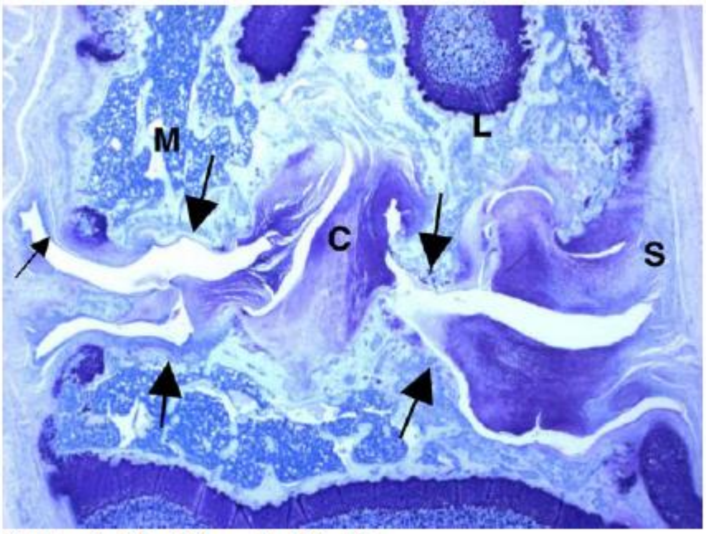

2. Knee from an animal treated with Clonidine has severe loss of cartilage on all four surfaces, mild subacute synovitis, and a large osteophyte. M and L identify medial and lateral aspects of the joint. C identifies cruciates. S identifies synovium. Large arrows identify representative affected cartilage surfaces; small arrow identifies osteophyte.

3. Grp 8, Clonidine, An 18, 16x.

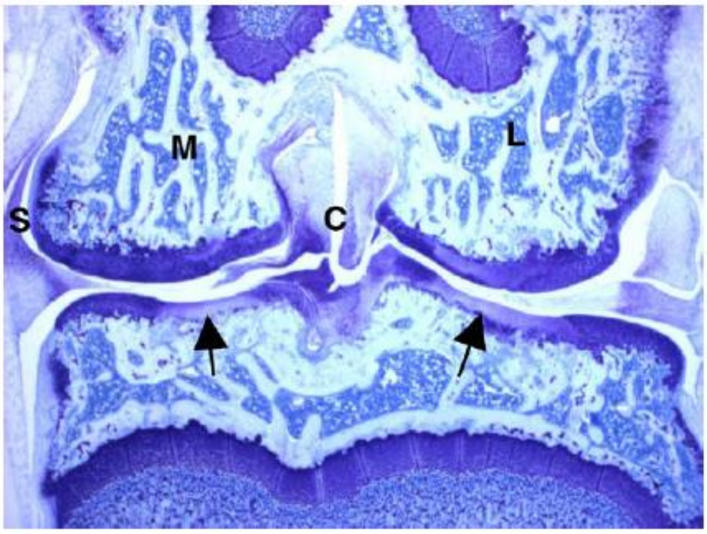

3. Knee from an animal treated with Clonidine has loss of cartilage on all four surfaces and minimal subacute synovitis. M and L identify medial and lateral aspects of the joint. C identifies cruciates. S identifies synovium. Large arrows identify representative affected cartilage surfaces.

MMT/MCLT Model Functional Assessment

Using the MMT/MCLT model, a number of the same agents were tested via systemic delivery and using incapacitance /weight bearing as a measure of pain.

MMT/MCLT Trial 1: Weight Bearing Results

In the first MMT/MCLT trial morphine, Tacrolimus, Fluocinolone, Clonidine, Alendronate, Curcumin, and Withaferin were tested. Alendronate was used as a control for bone degeneration, and Withaferin was added as a nutraceutical which showed promise in in vitro testing.

All animals resumed weight bearing upon recovery from anesthesia after surgery and there was no evidence of excessive post-operative swelling indicative of joint infection. Animals given Tacrolimus (both doses) had blood in the stool from day 7 to study termination. Animals in all IP-dosed compound treatment groups except for the Alendronate group gained significantly less weight over the course of the study than the Saline vehicle controls. Effects on body weight were dose responsive for Tacrolimus and Fluocinolone, with both high doses resulting in approximately 85% less weight gain and the low doses showing approximately 60% less weight gain. Effects on weight bearing scores are discussed below.

1) Morphine

At pre-treatment baseline on day 7, rats to be given Saline or Morphine had significant right to left weight bearing difference (favoring more weight on the left). Significance was also seen in the Saline group throughout the remainder of the study and in the Morphine group throughout the remainder of day 7. The baseline measure of right vs. left was significantly different for the Morphine group on day 21. Left/right weight distribution differences in Morphine-treated rats on days 7 and 14 decreased (toward the right) to the greatest degree at one hour post dose, and returned to baseline levels by twenty-four hours post dose. They were also observed to be sedated by treatment during the one-hour post dose testing. On day 21, Morphine-treated rats leaned progressively more toward the right leg from baseline to five hours post-dose, then returned to baseline levels at twenty-four hours. The one and three hour time points were also not significantly different between left and right.

2) Tacrolimus

At pre-treatment baseline on day 7, rats to be given Tacrolimus (0.3 or 0.6 mg/kg i.p.daily) had similar and significant right to left weight bearing differences (favoring more weight on the left) compared to the vehicle control rats. The difference in hind paw weight distribution was very close to the Saline vehicle controls at this time point. At one hour post treatment, rats given 0.3 mg/kg of Tacrolimus had similar weight bearing values to those observed at baseline, while rats given 0.6 mg/kg favored the left leg to a lesser degree, similar to the Morphine controls. At the remaining day 7 time points (3, 5, and 24 hours), rats given 0.3 mg/kg had left/right weight differences that dropped to less than half of the one hour value at three hours, then returned to the baseline level by twenty-four hours. Rats given 0.6 mg/kg Tacrolimus were similar to the Saline vehicle controls at all three time points. Effects of the 0.6 mg/kg dose were comparable to Morphine. The force exerted on the right leg expressed as a percentage of the total hind leg force showed similar results. At no time point was the data significantly different from the baseline.

At pre-treatment baseline and one hour post-dose on day 14, differences in force exerted on the left and right legs of rats to be given Tacrolimus were not significant, although they still leaned to the left. Left/right distribution was similar to Morphine controls and considerably reduced (toward the right) compared to the vehicle controls. The same was true for the percentage of the total hind leg force on the right leg. At three hours post dose, the 0.6 mg/kg group bore significantly less weight on the right leg than the left, but differences at five and twenty-four hours were not significant. Left/right distribution for both doses was similar to the Morphine controls at three, five, and twenty-four hours, with the exception of the high dose at three hours, which was increased (toward the left) even above the Saline controls. At no time point was the data significantly different from the baseline. At baseline on day 21, differences in force exerted on the left and right legs of rats to be given 0.6 mg/kg of Tacrolimus were significant. This was also true at five hours. Left/right distribution was similar to baseline at all time points except for the three hour, at which point the low (0.3 mg/kg) dose animals leaned slightly to the right. The percentage of total hind leg force on the right leg showed similar results. At no time point was the data significantly different from the baseline.

3) Fluocinolone

On day 7, rats given Fluocinolone (5 or 10 ug/kg i.p. daily) placed more weight on their left legs than their rights (with sporadic exceptions). This difference was significant for the low dose group at one hour. Left/right distribution and the percentage of weight on the right leg were generally improved (toward the right) compared to the Morphine and Saline controls, as well as the Tacrolimus groups. There was no discernable pattern to the change in weight bearing over the course of the day, and at no time point was the data significantly different from the baseline. On day 14, rats given Fluocinolone (5 or 10 ug/kg i.p. daily) placed more weight on their left legs than their rights (with sporadic exceptions). This difference was significant for the low dose group at five and twenty-four hours. Left/right distribution and the percentage of weight on the right leg were generally improved (toward the right) compared to the Saline controls, and were frequently comparable to the morphine controls. There was no discernable pattern to the change in weight bearing over the course of the day, and at no time point was the data significantly different from the baseline. On day 21, rats given 10 ug Fluocinolone placed significantly more weight on their left legs than their rights at all time points except for three hours. The 5 ug group leaned non-significantly to the left. Left/right distribution was very slightly improved (toward the right) compared to the Saline vehicle controls, with benefit in the high dose group peaking at three hours post-dose before returning to near baseline levels by twenty-four hours. The percentage of hind paw force on the right leg was comparable to that of the vehicle controls. At no time point was the data significantly different from the baseline.

4) Clonidine and Alendronate

On day 7, animals treated with Clonidine (100 ug/kg s.c. daily) or Alendronate (30 ug i.p. daily) bore significantly more weight on their left legs at all time points except for one hour post-dose. However, left/right distribution for was improved compared to the Saline controls at all time points except for three hours post-dose. Rats given Clonidine were improved compared to the Morphine controls as well at all time points, while rats given Alendronate were improved at all time points except for one hour post-dose. The percent of total hind leg force on the right leg was comparable to the vehicle controls at most time points, although improvement was seen at one hour (Clonidine) and five hours (both Clonidine and Alendronate). At no time point was the data significantly

different from the baseline. On day 14, animals treated with Clonidine bore significantly more weight on their left legs at baseline, and animals treated with Alendronate bore significantly more weight on the left at baseline, five hours, and twenty-four hours. Left/right weight distribution for Clonidine improved (toward right) from baseline to three hours (comparable to morphine), then increased (toward the left) to above the baseline and Saline control levels at twenty-four hours.

Animals treated with Alendronate followed a similar pattern, with peak benefit at one hour post-dose. A similar pattern was seen in the percentage of total hind leg force on the right leg. At no time point was the data significantly different from the baseline. On day 21, animals treated with Alendronate bore significantly more weight on their left legs at baseline, three, five, and twenty-four hours. Left/right weight distribution for Clonidine improved (toward right) from baseline to five hours (comparable to Morphine), then increased (toward the left) at twenty-four hours. Alendronate had comparable benefit at one hour, but was otherwise similar to the vehicle controls. A similar pattern was seen in the percentage of total hind leg force on the right leg, although the effect due to Clonidine peaked at three hours. At no time point was the data significantly different from the baseline.

5) Curcumin and Withaferin

On day 7, animals treated with orally dosed 50 mg/kg Curcumin or orally dosed 50 mg/kg Withaferin bore significantly more weight on their left legs at all time points except for one hour post-dose. Left/right weight distribution for animals treated with Curcumin was similar to the Saline controls at all time points except for five hours post-dose, at which time treated animals favored, to a greater degree, more weight on the left. Distribution for animals treated with Withaferin was comparable to that of the Morphine controls. The percentage of total hind leg force on the right leg was comparable to the Saline controls for the Curcumin group and slightly increased at all time points for the Withaferin group. At no time point was the data significantly different from the baseline.

On day 14, animals treated with Withaferin bore significantly more weight on their left legs at baseline and five hours post-dose. Curcumin treated animals bore slightly more weight on their right legs at three and five hours post-dose. Left/right weight distribution for Curcumin treated animals at all post-treatment time points was comparable to Morphine, while left/right distribution for Withaferin treated animals was similar to that of the Saline controls. A similar pattern was seen in the percentage of total hind leg force on the right leg. At no time point was the data significantly different from the baseline.

On day 21, animals treated with Withaferin bore significantly more weight on their left legs at three, five, and twenty-four hours post-dose. Left/right weight distribution at baseline and one hour was comparable to Morphine for both compounds. Animals treated with Curcumin remained similar to Morphine treated animals at three, five, and twenty-four hours, while Withaferin treated animals trended closer to the Saline controls. A similar pattern was seen in the percentage of total hind leg force on the right leg, although differences were less distinct. At no time point was the data significantly different from the baseline.

Below are shown results of incapacitance testing calculated as a ratio of injured to non-injured WB for days 7, 14, and 21 (**Figure 56 A, B, and C**). The average value for baseline is shown as a line (*:$p < .05$, 2 tailed, unpaired t-test, versus saline vehicle control).

Figure 56 - Day 7, 14, and 21 incapacitance results displayed as a ratio of injured to non-injured and compared with baseline control (shown as a line).

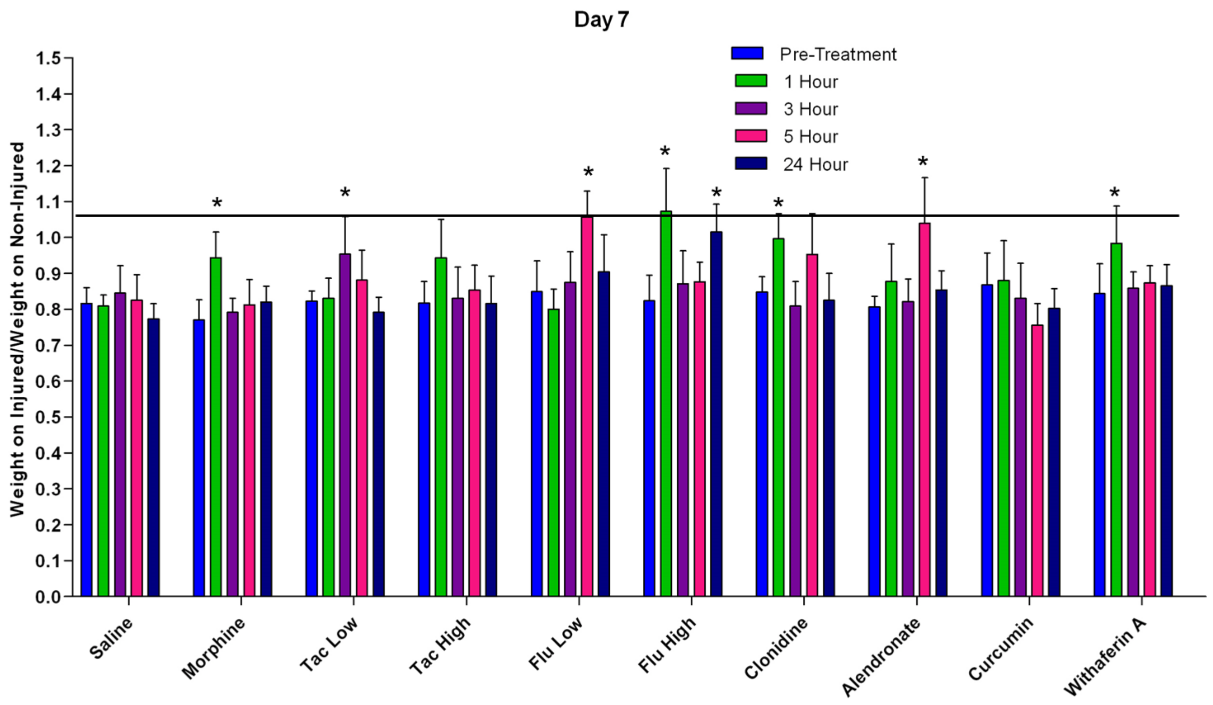

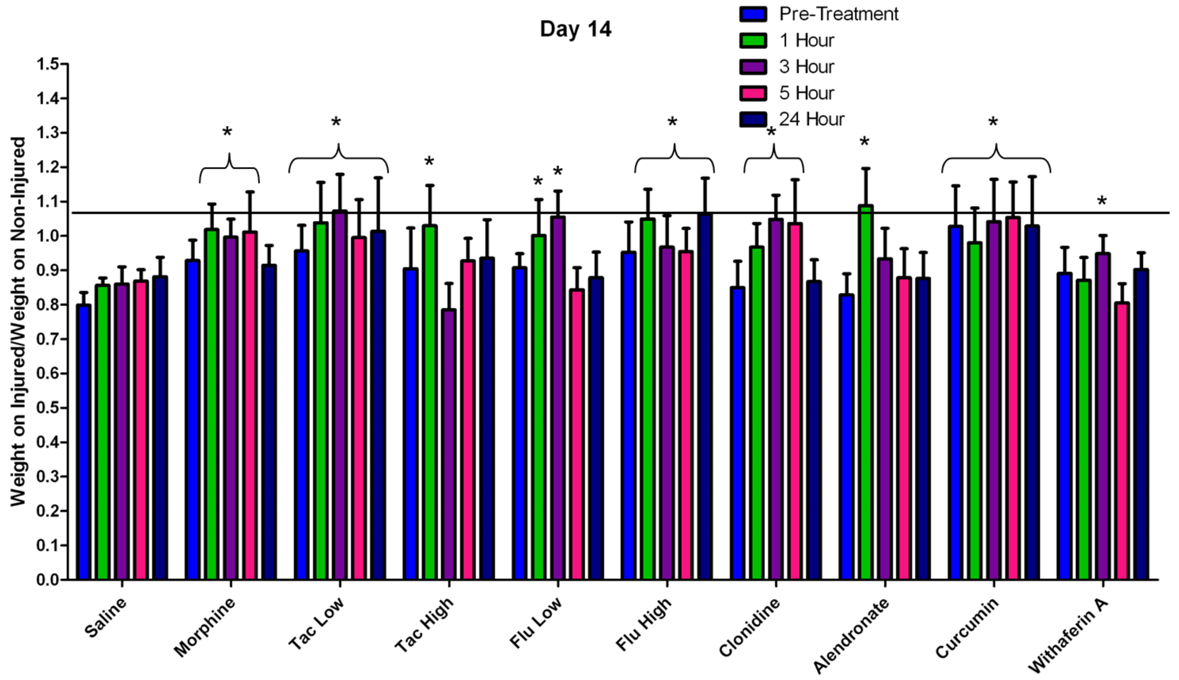

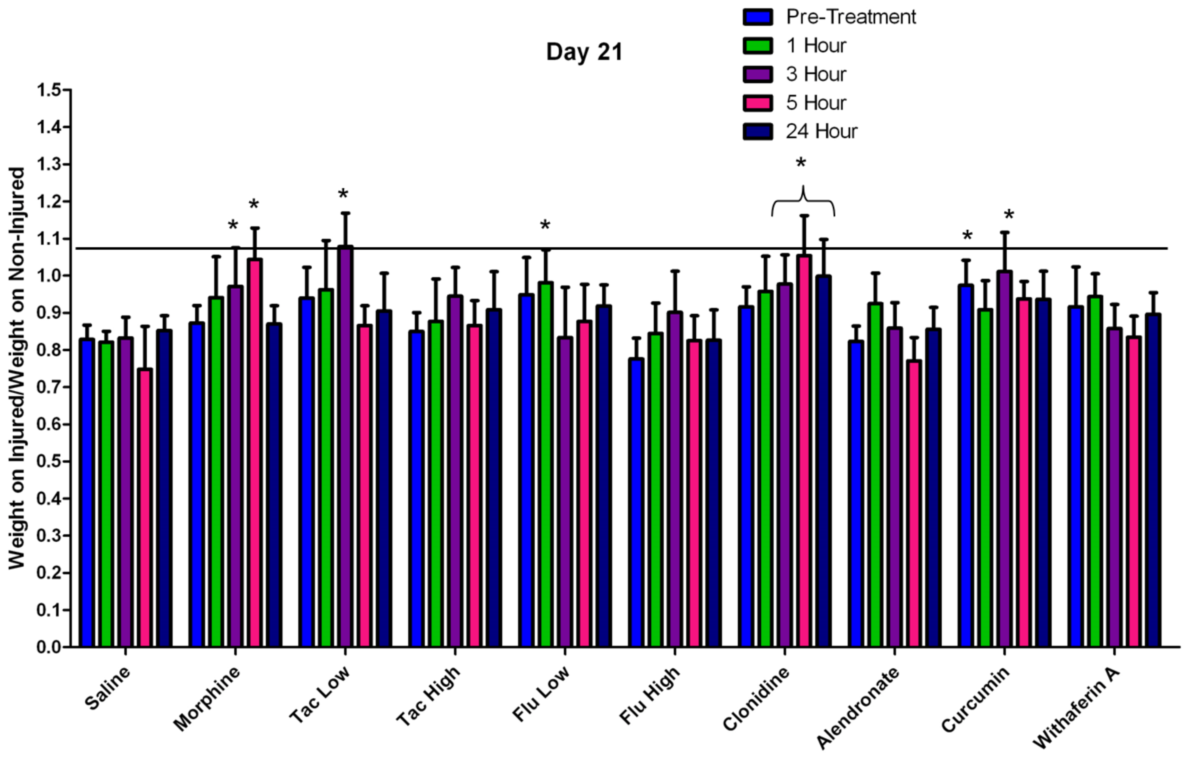

MMT/MCLT Trial 2: Weight bearing Results

Drugs of continuing interest were tested using intra-articular administration.

All animals resumed weight bearing post-surgery upon recovery from anesthesia and there was no evidence of excessive post-operative swelling indicative of joint infection. There was no significant variation in body weight gain over the course of the study. The animals of every group bore significantly less weight on the injuring right legs as compared to the left at all time points. Weight bearing data is included in the Appendix.

1) Saline (Group 1 Appendix)

The Vehicle control animals injected intra-articularly once a week with saline bore 43.8-46.4% of the total hind paw weight on their right legs over the course of the study. There was no pattern to the changes at later time points compared to pre-treatment on days 7 or 14, but on day 21, weight distribution was progressively worse.

2) Clonidine (Group 2 Appendix)

Control animals treated with subcutaneous weekly injections of 100 ug/kg Clonidine leaned significantly more on the left leg than the right (presumably due to increased pain) compared to the vehicle controls on day 21 at the pre-treatment time point. This was true of both the right/left ratio and the percent of hind paw weight on the right leg. There were no other significant differences in left/right weight bearing compared to the controls, but the percentage of the total body weight carried on the hind legs was significantly increased on days 7 (3 and 5 hr), 14 (5 hr), and 21 (3 and 5 hr). This parameter was significantly decreased at pre-treatment on day 21.

3) IA Clonidine (Group 3 Appendix)

Animals treated with intra-articular (IA) weekly injections of Clonidine (4.5 ug) showed significantly more discomfort as measured by weight bearing when compared with the vehicle controls at the 5 hour time point on days 7 and 14 and the 3 hour time point on day 21. This was evident in both the right/left ratio and the percent of hind paw weight on the right leg. There were no other significant differences in left/right weight bearing compared to the controls, but the percentage of the total body weight carried on the hind legs was significantly increased on days 7 (1 hr) and 21 (1 hr). This parameter was significantly decreased at pre-treatment on day 14 (1 hr). Weight bearing on the left leg at 1, 3, and 5 hours was generally greater than at pre-treatment.

4) IA Triamcinolone Hexacetonide (TH) (Group 4 Appendix)

Animals treated with TH IA injections once a week (0.15 mg, i.a.) bore significantly less weight on the surgical leg at the three hour time point on day 21. This was evident in both the right/left ratio and the percent of hind paw weight on the right leg. There were no other significant differences in left/right weight bearing compared to the controls, but the percentage of the total body weight carried on the hind legs was significantly increased on days 14 (1 and 5 hrs) and 21 (1 and 5 hrs). Weight bearing on the right leg at 1, 3, and 5 hours was generally less than at pre-treatment.

5) IA Tacrolimus (Group 5 & 6 Appendix)

Animals treated with 15 ng IA Tacrolimus once a week had significantly increased pain at the pre-treatment time point on day 21 based on the ratio of weight on the right leg vs. the left. This data was not significant when expressed as a percentage of the total hind leg force. Shifting of weight from the injured paw to the left leg tended to increase at the later time points after injection of the 15 ng Tacrolimus as compared to pre-treatment, except on day 14. In contrast, this was a significant shift in animals treated with 30 ng IA Tacrolimus once a week at the five hour time point on day 7 and the three hour time point on day 21. This was evident in both the right/left ratio and the percent of hind paw weight on the right leg. There were no other significant differences in left/right weight bearing compared to the controls. Presumed pain as indicated by this shift in weight bearing at 1, 3, and 5 hours was greater than at pre-treatment.

6) IA Fluocinolone (Group 7 Appendix)

Animals treated with 15 ng IA Fluocinolone had significantly increased favoring of the right paw at the three hour time point on day 14. This was evident in both the right/left ratio and the percent of hind paw weight on the right leg. There were no other significant differences in left/right weight bearing compared to the controls, but the percentage of the total body weight carried on the hind legs was significantly increased on day 21 (pre-treatment). The change in weight bearing toward greater use of the left paw at 1, 3, and 5 hours was greater than at pre-treatment.

7) IA Curcumin (Group 8 Appendix)

Animals treated with 30 μg IA Curcumin once a week had significantly increased changes in weight bearing indicating discomfort at the three and five hour time points on days 14 and 21. This was evident in both the right/left ratio and the percent of hind paw weight on the right leg. There were no other significant differences in left/right weight bearing compared to the controls, but the percentage of the total body weight carried on the hind legs was significantly increased on day 14 (3 and 5 hrs). Pain at 1, 3, and 5 hours was generally greater than at pre-treatment.

MMT/MCLT Model Histopathology Results

All studies completed at Bolder BioPATH included a complete analysis of the joint histopathology for each test animal including joint space and synovial tissue.

MMT/MCLT Trial 1: Representative Images

Shown in **Figure 57** below are representative images (50x) from animals of treatment groups in the first MMT/MCLT Trial with systemic delivery of therapeutic agents. Marrow is depicted by "M" and the region of cartilage damage is depicted with an arrow. In these higher power images the osteophytes are out of view. A. Saline, B. Morphine, C. Tacrolimus (0.3 mg/kg), D. Tacrolimus (0.6 mg/kg), E. Fluocinolone ((0.005 mg/kg), F. Fluocinolone (0.010 mg/kg), G. Clonidine (0.1 mg/kg), H. Alendronate (10 μg/kg), I. Curcumin (50 mg/kg), J. Withaferin (50 mg/kg).

Figure 57. Representative images from Trial 1 MMT/MCLT

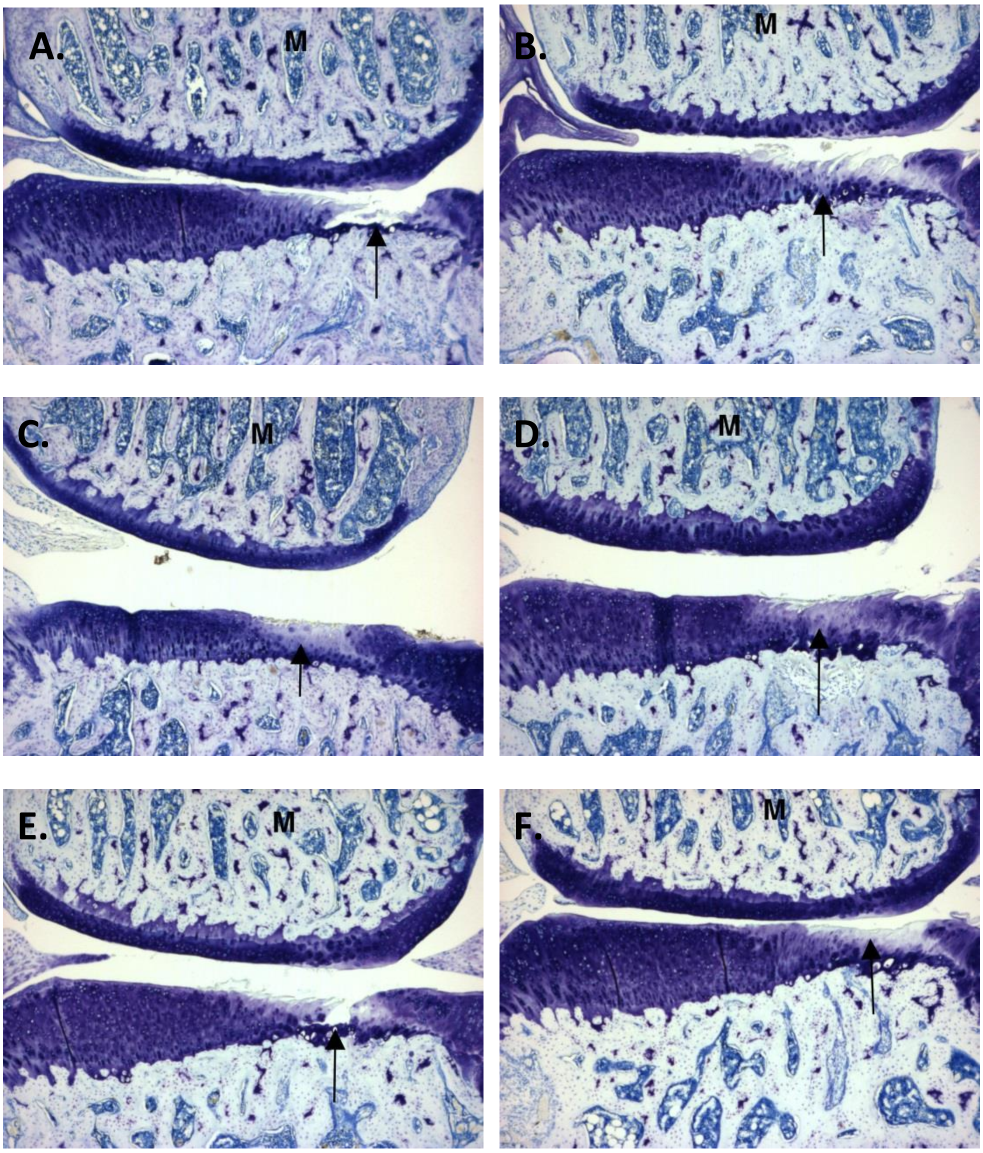

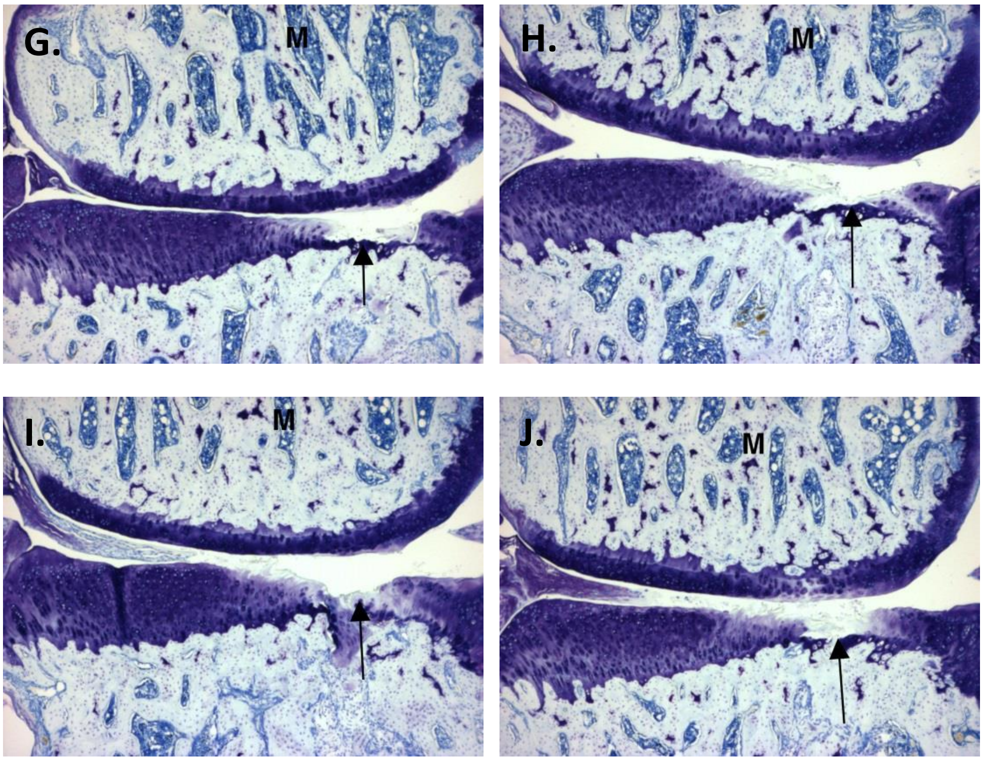

MMT/MCLT Trial 1: Semi-quantitative Analysis of Histological Sections

1) Vehicle (Saline)

Animals treated with vehicle alone had tibial cartilage degeneration that was most severe in the outer 2/3 of the tibial plateau and less severe in the inner 1/3. Osteophytes were present in all animals and ranged from large to very large, with a mean measurement of 513 um. Femoral cartilage degeneration was seen in all animals and ranged from minimal to marked when present. The mean total joint score for this group was 12.09.

Table 36. Histopathology of MMT/MCLT animals treated with vehicle alone (Group 1; Systemic delivery)

Treatment Group Animal #	Knee	Medial Tibial Cartilage Degeneration Score*				Tibial Cartilage Degeneration Width		Depth Ratio Any Matrix Change#		Medial Tibial Osteophyte		Medial Femur Cartilage Degeneration Score*	Bone Score	Total Joint Score w/ o Femur	Total Joint Score
		3 Zone total	Zone 1 (Outside)	Zone 2 (Middle)	Zone 3 (Inside)	Total (µm)**	Sig (µm)***	Mean	Zone 2	Score ##	Measure (µm)				
01 Vehicle															
1	R	7.00	4.67	2.33	0.00	1433	783	0.39	0.23	3.67	467	2.33	3.00	10.67	13.00
2	R	7.00	5.00	2.00	0.00	1333	767	0.42	0.27	4.00	553	2.67	3.00	11.00	13.67
3	R	7.00	4.67	2.33	0.00	1100	767	0.35	0.16	5.00	600	1.33	4.00	12.00	13.33
4	R	6.00	4.33	1.67	0.00	1100	667	0.30	0.06	3.67	480	1.00	3.00	9.67	10.67
5	R	5.67	3.67	2.00	0.00	1333	633	0.29	0.06	4.67	610	2.00	4.00	10.33	12.33
6	R	8.00	5.00	3.00	0.00	1367	900	0.45	0.34	3.00	440	3.33	3.00	11.00	14.33
7	R	6.67	3.67	2.67	0.33	1333	700	0.34	0.29	4.33	540	1.00	3.00	11.00	12.00
8	R	5.00	3.33	1.67	0.00	1500	600	0.32	0.06	3.00	413	1.00	3.00	8.00	9.00
Mean		6.54	4.29	2.21	0.04	1312.50	727.08	0.36	0.18	3.92	512.92	1.83	3.25	10.46	12.29
SE		0.33	0.23	0.17	0.04	50.76	34.21	0.02	0.04	0.26	25.99	0.31	0.16	0.42	0.62
%		0%	0%	0%	0%	0%	0%	0%	0%	0%	0%	0%	0%	0%	0%

2) Morphine

Animals treated with Morphine were similar to the controls for all general pathology parameters. Collagen degeneration widths were not significantly affected, nor were cartilage matrix areas. Mean growth plate and MCL thickness measurements were identical to the controls.

Table 37. Histopathology of MMT/MCLT animals treated with morphine (Group 2; Systemic delivery)

Treatment Group Animal #	Knee	Medial Tibial Cartilage Degeneration Score* 3 Zone total	Zone 1 (Outside)	Zone 2 (Middle)	Zone 3 (Inside)	Tibial Cartilage Degeneration Width Total (µm)**	Sig (µm)***	Depth Ratio Any Matrix Change# Mean	Zone 2	Medial Tibial Osteophyte Score ##	Measure (µm)	Medial Femur Cartilage Degeneration Score*	Bone Score	Total Joint Score w/o Femur	Total Joint Score
**02 Morphine**															
1	R	6.33	4.00	2.33	0.00	1233	733	0.38	0.17	4.33	587	3.00	3.00	10.67	13.67
2	R	5.33	3.67	1.67	0.00	1367	600	0.26	0.01	4.00	540	1.33	3.00	9.33	10.67
3	R	7.00	4.67	2.33	0.00	1333	767	0.43	0.29	5.00	663	1.33	4.00	12.00	13.33
4	R	6.00	4.00	2.00	0.00	1133	667	0.32	0.06	4.00	527	1.33	3.00	10.00	11.33
5	R	7.67	5.00	2.67	0.00	1300	767	0.40	0.24	5.00	600	2.33	4.00	12.67	15.00
6	R	6.67	4.67	2.00	0.00	1033	700	0.32	0.00	4.00	500	1.33	4.00	10.67	12.00
7	R	6.00	4.67	1.33	0.00	1200	600	0.35	0.06	5.00	647	6.67	4.00	11.00	17.67
8	R	4.67	3.33	1.00	0.33	1633	500	0.27	0.02	4.00	527	1.33	4.00	8.67	10.00
**Mean**		**6.21**	**4.25**	**1.92**	**0.04**	**1279.17**	**666.67**	**0.34**	**0.11**	**4.42**	**573.75**	**2.33**	**3.63**	**10.63**	**12.96**
**SE**		**0.33**	**0.21**	**0.20**	**0.04**	**63.60**	**33.33**	**0.02**	**0.04**	**0.18**	**21.22**	**0.66**	**0.18**	**0.46**	**0.89**
**ttest to G1**		**0.491**	**0.895**	**0.276**	**1.000**	**0.688**	**0.227**	**0.550**	**0.200**	**0.131**	**0.091**	**0.504**	**0.149**	**0.795**	**0.548**
**%**		**5%**	**1%**	**13%**	**0%**	**3%**	**8%**	**5%**	**42%**	**-13%**	**-12%**	**-27%**	**-12%**	**-2%**	**-5%**

*Cartilage degeneration score=depth (0-5) for each 1/3 then summed (mean of 3 step section)
**Width of any cartilage lesion (mean of 3 step section)
***Width of cartilage degeneration extending >50% of total thickness (mean of 3 step section)
#Mean Lesion depth in µm vs depth to tidemark in center of zone in the Tibial plateau (mean of 3 step section)
##Osteophyte scores 1=small up to 299 µm, 2=medium 300-399 µm, 3=large 400-499 µm, 4=very large 500-599 µm, 5=very large ≥600 µm

3) Tacrolimus (0.3 mg/kg, i.p.)

Animals treated IP with 0.3 mg/kg of Tacrolimus had significantly decreased cartilage degeneration scores (zones 1, 2, and the 3-zone total) in the medial tibia (35 %). The width of significant cartilage degeneration was significantly decreased (39%), as were the mean (29%) and zone 2 (59%) depth ratio of any matrix change, osteophyte scores (39%) and measurements (28%), femoral cartilage degeneration scores (73%), and the total joint score both with (42%) and without (37%) the femur. Subchondral bone scores were reduced non-significantly by 19%. The widths of severe (97%) and moderate (91%) collagen degeneration were significantly decreased, resulting in significant decreases to combined widths of severe+marked+moderate+mild (33%), severe+marked+moderate (88%), and severe+marked (86%) degeneration. The areas of viable (26%) and any (59%) cartilage matrix were significantly increased. Growth plate thickness was significantly decreased compared to the vehicle controls. Five animals had decreased metaphyseal/diaphyseal new bone.

Table 38. Histopathology of MMT/MCLT animals treated with Tacrolimus (Group 3; Systemic low dose)

Treatment Group Animal #	Knee	Medial Tibial Cartilage Degeneration Score* 3 Zone total	Zone 1 (Outside)	Zone 2 (Middle)	Zone 3 (Inside)	Tibial Cartilage Degeneration Width Total (µm)**	Sig (µm)***	Depth Ratio Any Matrix Change# Mean	Zone 2	Medial Tibial Osteophyte Score ##	Measure (µm)	Medial Femur Cartilage Degeneration Score*	Bone Score	Total Joint Score w/o Femur	Total Joint Score
**03 A1 0.3 mg/kg**															
1	R	4.00	2.33	1.67	0.00	1167	433	0.19	0.12	3.00	413	0.33	2.00	7.00	7.33
2	R	4.67	3.33	1.33	0.00	1300	567	0.30	0.02	3.00	413	1.00	2.00	7.67	8.67
3	R	3.33	2.33	1.00	0.00	1067	333	0.21	0.10	3.00	427	0.33	4.00	6.33	6.67
4	R	4.33	2.67	1.67	0.00	1400	500	0.28	0.07	1.00	280	0.00	3.00	5.33	5.33
5	R	6.67	4.00	2.67	0.00	1333	733	0.37	0.23	2.33	353	1.00	2.00	9.00	10.00
6	R	4.67	2.67	2.00	0.00	1033	567	0.26	0.00	3.00	450	0.33	4.00	7.67	8.00
7	R	3.67	3.00	0.67	0.00	867	300	0.33	0.01	2.67	390	1.00	2.00	6.33	7.33
8	R	2.67	1.67	1.00	0.00	933	100	0.12	0.06	1.00	240	0.00	2.00	3.67	3.67
**Mean**		**4.25**	**2.75**	**1.50**	**0.00**	**1137.50**	**441.67**	**0.26**	**0.07**	**2.38**	**370.83**	**0.50**	**2.63**	**6.63**	**7.13**
**SE**		**0.42**	**0.25**	**0.23**	**0.00**	**68.84**	**69.22**	**0.03**	**0.03**	**0.31**	**26.40**	**0.15**	**0.32**	**0.58**	**0.69**
**ttest to G1**		**0.001**	**0.000**	**0.025**	**0.334**	**0.060**	**0.002**	**0.011**	**0.042**	**0.002**	**0.002**	**0.002**	**0.107**	**0.000**	**0.000**
**%**		**35%**	**36%**	**32%**	**100%**	**13%**	**39%**	**29%**	**59%**	**39%**	**28%**	**73%**	**19%**	**37%**	**42%**

Animals treated IP with 0.6 mg/kg of Tacrolimus daily had significantly decreased cartilage degeneration scores (zones 1, 2, and the 3-zone total) in the medial tibia (45%). The widths of total (28%) and significant (55%) cartilage degeneration were significantly decreased as well, as were the mean (40%) and zone 2 (87%) depth ratio of any matrix change, osteophyte scores (39%) and measurements (28%), femoral cartilage degeneration scores (48%), and the total joint score both with (43%) and without (43%) the femur. The width of normal collagen was significantly increased by 19%. The widths of total (19%), severe (100%) and marked (97%) collagen degeneration were significantly decreased, resulting in significant decreases to combined widths of

severe+marked+moderate+mild (53%), severe+marked+moderate (96%), and severe+marked (99%) degeneration. The areas of viable (33%) and any (69%) cartilage matrix were significantly increased. Growth plate thickness was significantly decreased compared to the vehicle controls. All animals had decreased metaphyseal/diaphyseal new bone.

4) Tacrolimus (0.6 mg/kg, i.p.)

Table 39. Histopathology of MMT/MCLT animals treated with Tacrolimus (Group 4; Systemic high dose)

Treatment Group Animal #	Knee	Medial Tibial Cartilage Degeneration Score*				Tibial Cartilage Degeneration Width		Depth Ratio Any Matrix Change#		Medial Tibial Osteophyte		Medial Femur Cartilage Degeneration Score*	Bone Score	Total Joint Score w/o Femur	Total Joint Score
		3 Zone total	Zone 1 (Outside)	Zone 2 (Middle)	Zone 3 (Inside)	Total (µm)**	Sig (µm)***	Mean	Zone 2	Score ##	Measure (µm)				
04 A2 0.6 mg/kg															
1	R	2.33	2.00	0.33	0.00	833	100	0.19	0.01	2.33	353	0.00	4.00	4.67	4.67
2	R	4.00	2.33	1.67	0.00	1033	400	0.20	0.04	3.00	460	1.00	3.00	7.00	8.00
3	R	5.00	3.33	1.67	0.00	967	567	0.30	0.02	2.00	353	2.33	4.00	7.00	9.33
4	R	3.33	2.00	1.33	0.00	1000	267	0.14	0.09	2.67	393	1.00	3.00	6.00	7.00
5	R	4.00	2.67	1.33	0.00	933	400	0.23	0.00	3.00	400	1.33	3.00	7.00	8.33
6	R	5.00	3.33	1.67	0.00	967	600	0.28	0.04	2.00	313	1.33	3.00	7.00	8.33
7	R	3.00	2.00	1.00	0.00	833	167	0.24	0.00	2.00	320	0.00	2.00	5.00	5.00
8	R	2.33	1.67	0.67	0.00	1000	100	0.15	0.00	2.00	367	0.67	2.00	4.33	5.00
Mean		3.63	2.42	1.21	0.00	945.83	325.00	0.22	0.02	2.38	370.00	0.96	3.00	6.00	6.96
SE		0.38	0.22	0.18	0.00	26.68	70.08	0.02	0.01	0.16	16.81	0.27	0.27	0.41	0.65
ttest to G1		0.000	0.000	0.001	0.334	0.000	0.000	0.000	0.002	0.000	0.000	0.054	0.438	0.000	0.000
%		45%	44%	45%	100%	28%	55%	40%	87%	39%	28%	48%	8%	43%	43%
*Cartilage degeneration score=depth (0-5) for each 1/3 then summed (mean of 3 step section)															
**Width of any cartilage lesion (mean of 3 step section)															
***Width of cartilage degeneration extending >50% of total thickness (mean of 3 step section)															
#Mean Lesion depth in µm vs depth to tidemark in center of zone in theTibial plateau (mean of 3 step section)															
##Osteophyte scores 1=small up to 299 µm, 2=medium 300-399 µm, 3=large 400-499 µm, 4=very large 500-599 µm, 5=very large ≥600 µm															

5) Fluocinolone (5 µg/kg, i.p.)

Animals treated IP with 5 ug/kg daily of Fluocinolone had significantly reduced bone scores compared to the vehicle controls (27%). Other general pathology parameters were similar to the controls. Collagen degeneration widths were not significantly affected. The area of viable cartilage matrix was significantly increased by 17%. Growth plate thickness was significantly decreased compared to the vehicle controls.

Table 40. Histopathology of MMT/MCLT animals treated with fluocinolone (Group 5; Systemic low dose)

Treatment Group Animal #	Knee	Medial Tibial Cartilage Degeneration Score*				Tibial Cartilage Degeneration Width		Depth Ratio Any Matrix Change#		Medial Tibial Osteophyte		Medial Femur Cartilage Degeneration Score*	Bone Score	Total Joint Score w/o Femur	Total Joint Score
		3 Zone total	Zone 1 (Outside)	Zone 2 (Middle)	Zone 3 (Inside)	Total (µm)**	Sig (µm)***	Mean	Zone 2	Score ##	Measure (µm)				
05 B1 5 µg/kg															
1	R	6.33	5.00	1.33	0.00	933	667	0.34	0.02	3.00	400	1.00	2.00	9.33	10.33
2	R	6.33	4.33	2.00	0.00	1300	667	0.39	0.15	4.67	567	1.67	2.00	11.00	12.67
3	R	6.67	4.67	2.00	0.00	1067	700	0.41	0.22	2.33	387	0.67	2.00	9.00	9.67
4	R	5.67	4.00	1.67	0.00	1300	600	0.38	0.16	3.00	400	1.67	3.00	8.67	10.33
5	R	5.67	4.00	1.33	0.33	1100	533	0.33	0.03	3.33	467	1.67	2.00	9.00	10.67
6	R	3.33	2.33	1.00	0.00	1000	233	0.27	0.04	3.00	433	1.33	3.00	6.33	7.67
7	R	7.67	4.67	3.00	0.00	1433	733	0.37	0.21	3.67	493	1.67	3.00	11.33	13.00
8	R	7.00	4.33	2.67	0.00	1100	733	0.34	0.11	3.67	493	1.00	2.00	10.67	11.67
Mean		6.08	4.17	1.88	0.04	1154.17	608.33	0.35	0.12	3.33	455.00	1.33	2.38	9.42	10.75
SE		0.46	0.29	0.24	0.04	60.73	58.67	0.02	0.03	0.24	21.85	0.14	0.18	0.57	0.61
ttest to G1		0.432	0.740	0.277	1.000	0.065	0.102	0.773	0.214	0.123	0.110	0.169	0.003	0.164	0.096
%		7%	3%	15%	0%	12%	16%	2%	35%	15%	11%	27%	27%	10%	13%

Animals treated IP with 10 ug/kg of Fluocinolone had significantly reduced widths of significant cartilage degeneration (18%). Osteophyte scores (30%) and measurements (23%) were also significantly decreased, as was the total joint score without the femur (18%). Collagen degeneration widths were not significantly affected. The area of viable cartilage matrix was significantly increased by 20%. The mean growth plate thickness was significantly reduced.

6) Fluocinolone (10 µg/kg, i.p.)

Table 41. Histopathology of MMT/MCLT animals treated with fluocinolone (Group 6; Systemic high dose)

Treatment Group Animal #	Knee	Medial Tibial Cartilage Degeneration Score*				Tibial Cartilage Degeneration Width		Depth Ratio Any Matrix Change#		Medial Tibial Osteophyte		Medial Femur Cartilage Degeneration Score*	Bone Score	Total Joint Score w/o Femur	Total Joint Score
		3 Zone total	Zone 1 (Outside)	Zone 2 (Middle)	Zone 3 (Inside)	Total (µm)**	Sig (µm)***	Mean	Zone 2	Score ##	Measure (µm)				
06 B2 10 µg/kg															
1	R	6.33	4.33	2.00	0.00	1133	633	0.34	0.05	3.00	413	2.00	4.00	9.33	11.33
2	R	4.67	3.33	1.33	0.00	1000	433	0.28	0.06	1.33	280	3.00	1.00	6.00	9.00
3	R	7.00	4.67	2.33	0.00	1133	767	0.39	0.16	3.00	427	2.33	2.00	10.00	12.33
4	R	7.67	4.67	2.67	0.33	1600	800	0.42	0.31	4.67	587	3.00	3.00	12.33	15.33
5	R	5.33	4.00	1.33	0.00	1267	567	0.33	0.05	3.00	400	1.33	3.00	8.33	9.67
6	R	6.00	4.00	2.00	0.00	1100	667	0.35	0.10	2.67	380	2.00	3.00	8.67	10.67
7	R	4.67	3.00	1.67	0.00	1400	467	0.29	0.13	2.00	333	1.00	3.00	6.67	7.67
8	R	5.00	3.00	2.00	0.00	1067	433	0.32	0.03	2.33	333	1.00	2.00	7.33	8.33
**Mean**		**5.83**	**3.88**	**1.92**	**0.04**	**1212.50**	**595.83**	**0.34**	**0.11**	**2.75**	**394.17**	**1.96**	**2.63**	**8.58**	**10.54**
**SE**		**0.39**	**0.24**	**0.16**	**0.04**	**70.69**	**51.35**	**0.02**	**0.03**	**0.34**	**32.50**	**0.28**	**0.32**	**0.71**	**0.88**
**ttest to G1**		**0.191**	**0.235**	**0.231**	**1.000**	**0.270**	**0.052**	**0.464**	**0.177**	**0.017**	**0.013**	**0.773**	**0.107**	**0.040**	**0.125**
**%**		**11%**	**10%**	**13%**	**0%**	**8%**	**18%**	**6%**	**41%**	**30%**	**23%**	**-7%**	**19%**	**18%**	**14%**

*Cartilage degeneration score=depth (0-5) for each 1/3 then summed (mean of 3 step section)
**Width of any cartilage lesion (mean of 3 step section)
***Width of cartilage degeneration extending >50% of total thickness (mean of 3 step section)
#Mean Lesion depth in µm vs depth to tidemark in center of zone in the Tibial plateau (mean of 3 step section)
##Osteophyte scores 1=small up to 299 µm, 2=medium 300-399 µm, 3=large 400-499 µm, 4=very large 500-599 µm, 5=very large ≥600 µm

7) Clonidine (100 µg/kg, i.p.)

Animals treated IP with 100 ug/kg daily of Clonidine were similar to the controls for all general pathology parameters. Collagen degeneration widths, cartilage areas, and growth plate and MCL thickness were also not significantly affected.

Table 42. Histopathology of MMT/MCLT animals treated with clonidine (Group 7; Systemic delivery)

Treatment Group Animal #	Knee	Medial Tibial Cartilage Degeneration Score*				Tibial Cartilage Degeneration Width		Depth Ratio Any Matrix Change#		Medial Tibial Osteophyte		Medial Femur Cartilage Degeneration Score*	Bone Score	Total Joint Score w/o Femur	Total Joint Score
		3 Zone total	Zone 1 (Outside)	Zone 2 (Middle)	Zone 3 (Inside)	Total (µm)**	Sig (µm)***	Mean	Zone 2	Score ##	Measure (µm)				
07 C1 100 µg/kg															
1	R	5.67	4.00	1.67	0.00	1033	567	0.34	0.03	4.33	580	1.00	4.00	10.00	11.00
2	R	7.67	5.00	2.67	0.00	1233	833	0.35	0.06	3.00	447	2.67	2.00	10.67	13.33
3	R	5.33	3.67	1.67	0.00	1067	633	0.35	0.15	4.00	580	1.00	2.00	9.33	10.33
4	R	5.00	4.00	1.00	0.00	1200	467	0.32	0.26	4.67	587	1.33	2.00	9.67	11.00
5	R	7.00	4.00	3.00	0.00	1367	867	0.35	0.25	3.33	473	1.00	3.00	10.33	11.33
6	R	6.00	4.00	2.00	0.00	1267	733	0.33	0.07	3.00	433	1.00	4.00	9.00	10.00
7	R	7.67	4.67	3.00	0.00	1200	933	0.47	0.44	5.00	700	1.33	2.00	12.67	14.00
8	R	4.67	3.67	1.00	0.00	1033	500	0.30	0.00	4.00	520	1.00	4.00	8.67	9.67
**Mean**		**6.13**	**4.13**	**2.00**	**0.00**	**1175.00**	**691.67**	**0.35**	**0.16**	**3.92**	**540.00**	**1.29**	**2.88**	**10.04**	**11.33**
**SE**		**0.42**	**0.17**	**0.29**	**0.00**	**42.61**	**62.28**	**0.02**	**0.05**	**0.27**	**31.60**	**0.20**	**0.35**	**0.44**	**0.55**
**ttest to G1**		**0.448**	**0.567**	**0.542**	**0.334**	**0.057**	**0.626**	**0.791**	**0.712**	**1.000**	**0.519**	**0.171**	**0.349**	**0.507**	**0.265**
**%**		**6%**	**4%**	**9%**	**100%**	**10%**	**5%**	**2%**	**14%**	**0%**	**-5%**	**30%**	**12%**	**4%**	**8%**

8) Alendronate (10 µg/kg, i.p.)

Animals treated IP with 10 ug/kg of Alendronate had significantly decreased widths of total cartilage degeneration (10%). Other general pathology parameters were similar to the controls for all general pathology parameters. Collagen degeneration widths, cartilage areas, and growth plate and MCL thickness were also not significantly affected. All animals had retained calcified cartilage at the physis/metaphysis.

Table 43. Histopathology of MMT/MCLT animals treated with Alendronate (Group 8; Systemic delivery)

Treatment Group		Medial Tibial				Tibial Cartilage		Depth Ratio		Medial Tibial		Medial	Bone	Total	Total
Animal #	Knee	Cartilage Degeneration Score*				Degeneration		Any Matrix		Osteophyte		Femur	Score	Joint	Joint
						Width		Change#				Cartilage		Score	Score
		3 Zone	Zone 1	Zone 2	Zone 3	Total	Sig		Zone	Score	Measure	Degeneration		w/o	
		total	(Outside)	(Middle)	(Inside)	(µm)**	(µm)***	Mean	2	##	(µm)	Score*		Femur	
08 D1 10 µg/kg															
1	R	5.33	3.33	2.00	0.00	1167	533	0.30	0.15	4.00	500	1.33	4.00	9.33	10.67
2	R	6.33	4.00	2.33	0.00	1133	733	0.34	0.04	4.67	567	0.67	2.00	11.00	11.67
3	R	6.33	4.00	2.33	0.00	1200	767	0.38	0.23	3.00	413	2.00	2.00	9.33	11.33
4	R	7.67	5.00	2.67	0.00	1233	833	0.44	0.32	4.00	540	2.33	3.00	11.67	14.00
5	R	5.33	3.67	1.67	0.00	1067	600	0.30	0.13	3.33	433	1.00	4.00	8.67	9.67
6	R	5.00	3.33	1.67	0.00	1367	600	0.33	0.09	3.00	440	1.00	3.00	8.00	9.00
7	R	5.00	3.33	1.67	0.00	1100	533	0.34	0.10	4.00	540	0.33	3.00	9.00	9.33
8	R	5.67	3.67	2.00	0.00	1133	600	0.37	0.17	3.00	400	5.67	1.00	8.67	14.33
**Mean**		**5.83**	**3.79**	**2.04**	**0.00**	**1175.00**	**650.00**	**0.35**	**0.15**	**3.63**	**479.17**	**1.79**	**2.75**	**9.46**	**11.25**
**SE**		**0.32**	**0.20**	**0.13**	**0.00**	**33.18**	**39.84**	**0.02**	**0.03**	**0.22**	**23.04**	**0.60**	**0.37**	**0.44**	**0.72**
**ttest to G1**		**0.148**	**0.123**	**0.446**	**0.334**	**0.040**	**0.164**	**0.765**	**0.599**	**0.406**	**0.348**	**0.952**	**0.233**	**0.124**	**0.289**
**%**		**11%**	**12%**	**8%**	**100%**	**10%**	**11%**	**2%**	**15%**	**7%**	**7%**	**2%**	**15%**	**10%**	**8%**

*Cartilage degeneration score=depth (0-5) for each 1/3 then summed (mean of 3 step section)
**Width of any cartilage lesion (mean of 3 step section)
***Width of cartilage degeneration extending >50% of total thickness (mean of 3 step section)
#Mean Lesion depth in µm vs depth to tidemark in center of zone in theTibial plateau (mean of 3 step section)
##Osteophyte scores 1=small up to 299 µm, 2=medium 300-399 µm, 3=large 400-499 µm, 4=very large 500-599 µm, 5=very large ≥600 µm

9) Curcumin (50 mg/kg, p.o.)

Animals treated PO with 50 mg/kg of Curcumin were similar to the controls for all general pathology parameters. Collagen degeneration widths, cartilage areas, and growth plate and MCL thickness were also not significantly affected.

Table 44. Histopathology of MMT/MCLT animals treated with Curcumin (Group 9; Oral delivery)

Treatment Group		Medial Tibial				Tibial Cartilage		Depth Ratio		Medial Tibial		Medial	Bone	Total	Total
Animal #	Knee	Cartilage Degeneration Score*				Degeneration		Any Matrix		Osteophyte		Femur	Score	Joint	Joint
						Width		Change#				Cartilage		Score	Score
		3 Zone	Zone 1	Zone 2	Zone 3	Total	Sig		Zone	Score	Measure	Degeneration		w/o	
		total	(Outside)	(Middle)	(Inside)	(µm)**	(µm)***	Mean	2	##	(µm)	Score*		Femur	
09 E1 50 mg/kg															
1	R	7.33	4.67	2.67	0.00	1400	767	0.42	0.30	5.00	767	0.67	4.00	12.33	13.00
2	R	7.00	4.00	3.00	0.00	1333	833	0.51	0.54	5.00	767	2.33	3.00	12.00	14.33
3	R	6.00	4.00	2.00	0.00	1433	667	0.32	0.03	5.00	600	1.00	4.00	11.00	12.00
4	R	6.67	4.67	2.00	0.00	1100	700	0.45	0.38	4.33	577	2.00	3.00	11.00	13.00
5	R	5.00	3.00	2.00	0.00	1167	767	0.26	0.05	3.00	413	2.67	4.00	8.00	10.67
6	R	7.33	4.33	2.67	0.33	1333	767	0.40	0.54	3.00	447	6.00	2.00	10.33	16.33
7	R	5.67	3.67	2.00	0.00	1233	600	0.32	0.10	4.00	580	2.00	2.00	9.67	11.67
8	R	7.00	4.33	2.67	0.00	1500	833	0.42	0.26	4.67	593	1.00	3.00	11.67	12.67
**Mean**		**6.50**	**4.08**	**2.38**	**0.04**	**1312.50**	**741.67**	**0.39**	**0.27**	**4.25**	**592.92**	**2.21**	**3.13**	**10.75**	**12.96**
**SE**		**0.30**	**0.20**	**0.15**	**0.04**	**48.36**	**28.70**	**0.03**	**0.07**	**0.30**	**45.22**	**0.60**	**0.30**	**0.50**	**0.62**
**ttest to G1**		**0.927**	**0.503**	**0.464**	**1.000**	**1.000**	**0.749**	**0.452**	**0.288**	**0.414**	**0.147**	**0.587**	**0.717**	**0.662**	**0.456**
**%**		**1%**	**5%**	**-8%**	**0%**	**0%**	**-2%**	**-8%**	**-50%**	**-9%**	**-16%**	**-20%**	**4%**	**-3%**	**-5%**

10) Withaferin (50 mg/kg, p.o.)

Animals treated PO with 50 mg/kg of Withaferin were similar to the controls for all general pathology parameters. Collagen degeneration widths, cartilage areas, and growth plate and MCL thickness were also not significantly affected.

Table 45. Histopathology of MMT/MCLT animals treated with Withaferin (Group 10; Oral delivery)

Treatment Group Animal #	Knee	Medial Tibial Cartilage Degeneration Score* 3 Zone total	Zone 1 (Outside)	Zone 2 (Middle)	Zone 3 (Inside)	Tibial Cartilage Degeneration Width Total (µm)**	Sig (µm)***	Depth Ratio Any Matrix Change# Mean	Zone 2	Medial Tibial Osteophyte Score ##	Measure (µm)	Medial Femur Cartilage Degeneration Score*	Bone Score	Total Joint Score w/o Femur	Total Joint Score
10 F1 50 mg/kg															
1	R	5.00	3.33	1.67	0.00	1567	533	0.37	0.15	4.00	520	2.00	3.00	9.00	11.00
2	R	5.00	4.00	1.00	0.00	1000	533	0.33	0.00	3.33	460	5.33	4.00	8.33	13.67
3	R	7.00	4.67	2.33	0.00	1200	667	0.36	0.16	4.67	593	1.00	3.00	11.67	12.67
4	R	6.33	4.00	2.33	0.00	1367	600	0.44	0.35	4.67	567	1.00	3.00	11.00	12.00
5	R	8.00	5.00	2.67	0.33	1600	833	0.49	0.49	4.00	513	2.00	2.00	12.00	14.00
6	R	4.67	3.67	1.00	0.00	1000	567	0.29	0.02	4.00	527	1.33	3.00	8.67	10.00
7	R	6.67	4.33	2.33	0.00	1233	767	0.42	0.28	4.67	593	1.00	2.00	11.33	12.33
8	R	5.67	4.00	1.67	0.00	1133	600	0.32	0.01	2.33	350	1.00	3.00	8.00	9.00
**Mean**		**6.04**	**4.13**	**1.88**	**0.04**	**1262.50**	**637.50**	**0.38**	**0.18**	**3.96**	**515.42**	**1.83**	**2.88**	**10.00**	**11.83**
**SE**		**0.41**	**0.19**	**0.23**	**0.04**	**82.00**	**39.06**	**0.02**	**0.06**	**0.28**	**28.46**	**0.52**	**0.23**	**0.58**	**0.61**
**ttest to G1**		**0.360**	**0.585**	**0.255**	**1.000**	**0.612**	**0.106**	**0.590**	**0.997**	**0.915**	**0.949**	**1.000**	**0.201**	**0.535**	**0.606**
**%**		**8%**	**4%**	**15%**	**0%**	**4%**	**12%**	**-5%**	**0%**	**-1%**	**0%**	**0%**	**12%**	**4%**	**4%**

*Cartilage degeneration score=depth (0-5) for each 1/3 then summed (mean of 3 step section)
**Width of any cartilage lesion (mean of 3 step section)
***Width of cartilage degeneration extending >50% of total thickness (mean of 3 step section)
#Mean Lesion depth in µm vs depth to tidemark in center of zone in theTibial plateau (mean of 3 step section)
##Osteophyte scores 1=small up to 299 µm, 2=medium 300-399 µm, 3=large 400-499 µm, 4=very large 500-599 µm, 5=very large ≥600 µm

Conclusions

Results of the histopathological analysis indicate that IP treatment with Tacrolimus (0.3 or 0.6 mg/kg) had significant beneficial effects on most parameters of medial meniscal tear induced osteoarthritis in rats, although these effects came in conjunction with decreased weight gain, occasional blood in the stool, and decreased metaphyseal/diaphyseal new bone, as well as decreased growth plate thickness. These effects on growth plate and bone formation are likely a result of decreased growth associated with decreased food consumption. Since these animals had blood in the stool it is likely that they may have been lethargic, which could have contributed to the observed beneficial effects on cartilage pathology. Treatment with 5 or 10 ug of Fluocinolone had more sporadic significant benefit on cartilage parameters without the effect on new bone, although growth plate thickness was decreased, as were subchondral bone scores. Decreased general activity likely contributed to the sporadic efficacy on cartilage parameters. Treatment with Alendronate caused retention of calcified cartilage in the physis/metaphysis, but in general did not beneficially affect cartilage parameters, as was the case with compounds Clonidine, Curcumin, and Withaferin.

Evaluation of weight bearing (incapacitance meter) did not reveal a consistent pattern of effect for any treatment except Morphine. Morphine treatment was effective on day 7 (1 hour), day 14 (1, 3, and 5 hours), and day 21 (1, 3, and 5 hours) based on the absence of a significant difference between load bearing of right and left legs. Comparisons made to baseline (left-right differential) indicated decreased discomfort at these same time points, but changes were not significant. Evaluation of the data as a percentage of the force on the right leg showed the same pattern. Larger group sizes could have potentially revealed significant differences.

Evaluation of the weight bearing data for Tacrolimus and Fluocinolone was complicated by toxicity, as indicated by decreased weight gain and clinical signs such as blood in the stool with Tacrolimus. If malaise and decreased activity were associated with dosing, this could have influenced weight bearing at all time points. These agents need to be tested at doses that don’t induce clinical signs of toxicity in order to evaluate their true effect on cartilage degeneration and pain.

MMT/MCLT Trial 2: Representative Images

Representative images from MCLT/MMT animal joints of the different treatments in Trial 2 are shown below in **Figures 58 and 59**. Group 1 was treated with intra-articular (IA) saline weekly. Group 2 was treated with subcutaneous clonidine weekly (100 ug/kg). Group 3 was treated with IA clonidine weekly (4.5 ug). Group 4 was treated with IA triamcinolone hexacetonide weekly (150 ug). Group 5 was treated with IA low dose tacrolimus weekly (15 ng). Group 6 was treated with IA high dose tacrolimus weekly (30 ng). Group 7 was treated with IA fluocinolone weekly (15 ng). Group 8 was treated with intra-articular curcumin weekly (30 ug).

Figure 58. Representative cross-sectional images from vehicle-treated MCLT/MMT animals (Trial 2).

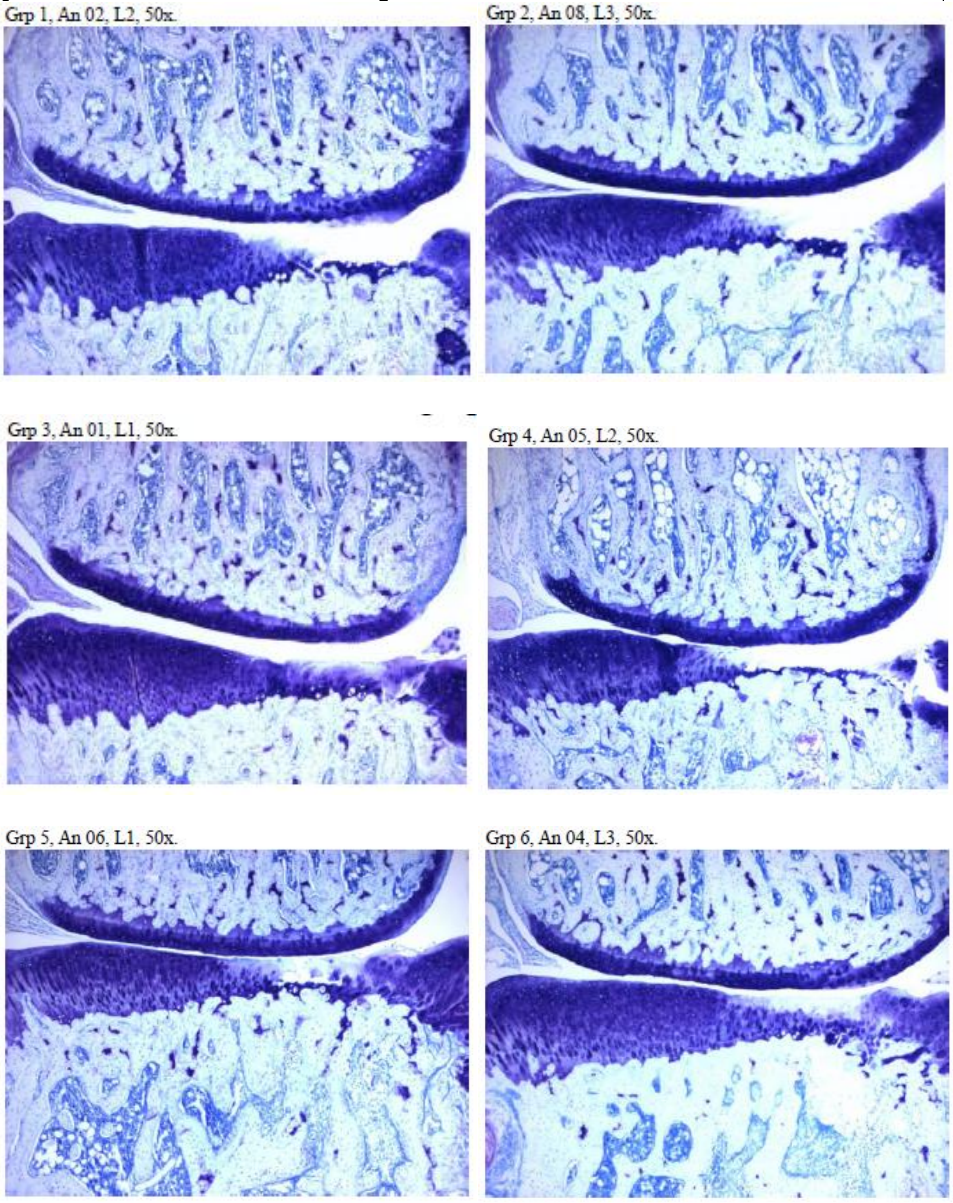

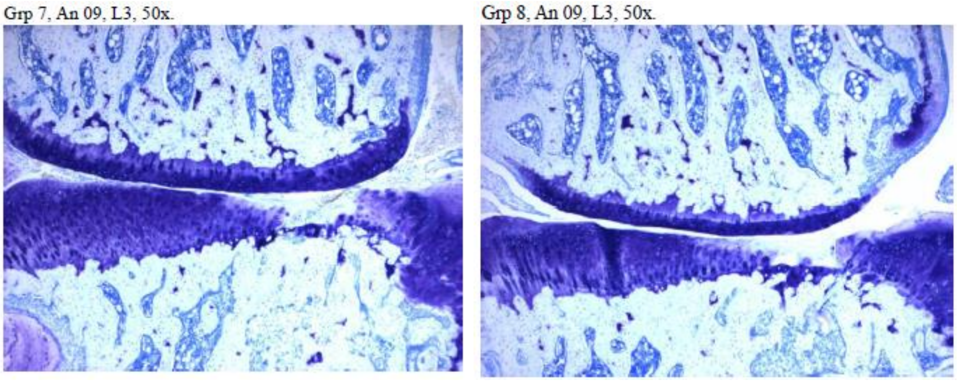

Shown below is a representative photomicrograph of a large osteophyte and synoviocytis seen in the synovial space in animal 2 of group 4 in Trial 2 (Triamcinolone hexacetonide treated animals). Analysis of the joint space was completed for each animal (discussed below but data not shown).

Figure 59 – Cross section of the joint space for Triamcinolone Hexacetonide treated rats (Trial 2) depicting a cartilage lesion (large arrow), an Osteophyte (small arrow), and a close up of the synovial membrane.

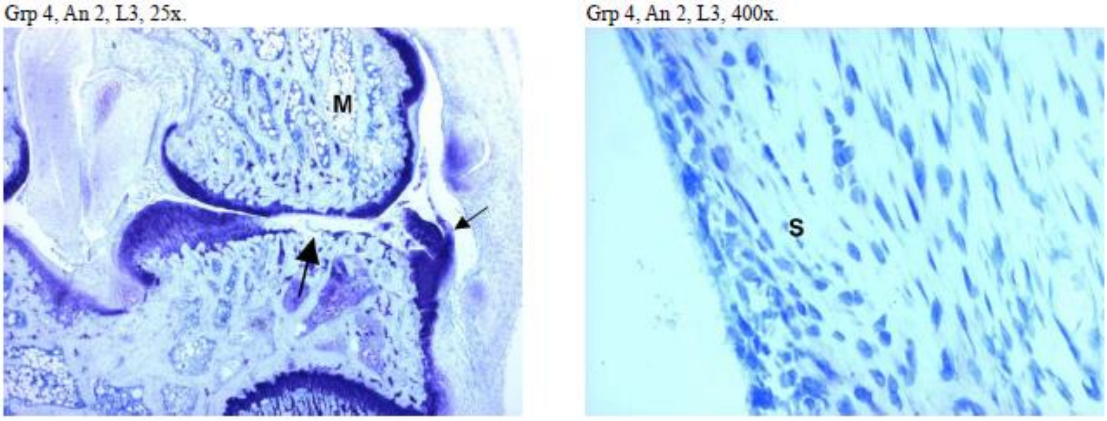

MMT/MCLT Trial 2: Semi-quantitative Analysis of Histological Sections

1) Saline (Vehicle)

Animals treated with IA saline alone had tibial cartilage degeneration that was most severe in the outer 2/3 of the tibial plateau and less severe in the inner 1/3 (see Figure 51 above). Osteophytes were present in all animals and ranged from medium to very large, with a mean measurement of 471 um. Femoral cartilage degeneration was seen in nine of ten animals and ranged from minimal to mild when present. The mean total joint score for this group was 10.23. Eight of ten animals had minimal to mild synovitis (e.g., Figure 69 above).

Table 46. Histopathology of MMT/MCLT animals treated with saline (Group 1; IA delivery)

Treatment Group Animal #	Knee	Medial Tibial Cartilage Degeneration Score* 3 Zone total	Zone 1 (Outside)	Zone 2 (Middle)	Zone 3 (Inside)	Tibial Cartilage Degeneration Width Total (µm)**	Sub (µm)***	Depth Ratio Any Matrix Change# Mean	Zone 2	Medial Tibial Osteophyte Score ##	Measure (µm)	Medial Femur Cartilage Degeneration Score*	Bone Score	Total Joint Score w/o Femur	Total Joint Score
01 Vehicle															
1	R	4.00	3.00	1.00	0.00	1000	400	0.27	0.00	2.00	327	0.67	4.00	6.00	6.67
2	R	8.00	5.00	3.00	0.00	1333	900	0.41	0.23	3.00	417	2.00	4.00	11.00	13.00
3	R	8.00	4.67	3.33	0.00	1500	900	0.43	0.29	4.67	567	0.67	3.00	12.67	13.33
4	R	4.33	3.33	1.00	0.00	967	533	0.26	0.03	2.33	333	1.00	3.00	6.67	7.67
5	R	5.33	3.67	1.67	0.00	1367	667	0.35	0.09	3.33	467	1.00	4.00	8.67	9.67
6	R	7.67	5.00	2.33	0.33	1400	833	0.39	0.20	4.33	567	2.00	3.00	12.00	14.00
7	R	5.67	4.33	1.33	0.00	967	733	0.37	0.10	3.67	467	0.00	4.00	9.33	9.33
8	R	6.33	4.00	2.33	0.00	1267	867	0.43	0.32	4.33	567	1.67	3.00	10.67	12.33
9	R	3.00	2.00	1.00	0.00	1267	333	0.17	0.01	4.00	500	1.67	2.00	7.00	8.67
10	R	3.67	2.33	1.33	0.00	1033	333	0.20	0.24	4.00	500	0.00	2.00	7.67	7.67
**Mean**		**5.60**	**3.73**	**1.83**	**0.03**	**1210.00**	**650.00**	**0.33**	**0.15**	**3.57**	**471.00**	**1.07**	**3.20**	**9.17**	**10.23**
**SE**		**0.59**	**0.34**	**0.27**	**0.03**	**63.26**	**73.74**	**0.03**	**0.04**	**0.28**	**28.27**	**0.24**	**0.25**	**0.74**	**0.85**
**%**		**0%**	**0%**	**0%**	**0%**	**0%**	**0%**	**0%**	**0%**	**0%**	**0%**	**0%**	**0%**	**0%**	**0%**

*Cartilage degeneration score=depth (0-5) for each 1/3 then summed (mean of 3 step section)
**Width of any cartilage lesion (mean of 3 step section)
***Width of cartilage degeneration extending >50% of total thickness (mean of 3 step section)
#Mean Lesion depth in µm vs depth to tidemark in center of zone in the Tibial plateau (mean of 3 step section)
##Osteophyte scores 1=small up to 299 µm, 2=medium 300-399 µm, 3=large 400-499 µm, 4=very large 500-599 µm, 5=very large ≥600 µm

2) Clonidine (100 µg/kg, s.c. 1x/wk)

Animals treated with subcutaneously delivered Clonidine (100 ug/kg) once a week had slightly elevated scores and measurements compared to the saline controls for all general pathology parameters. Osteophyte scores and measurements were significantly increased by 26 and 25%, respectively. The percent area of cartilage matrix was significantly decreased by 77%. Collagen degeneration and growth plate and MCL thicknesses were not significantly affected, although severe and marked collagen loss scores were slightly elevated. Three animals had minimal subacute synovitis.

Table 47. Histopathology of MMT/MCLT animals treated with clonidine (Group 2; SC delivery)

Treatment Group Animal #	Knee	Medial Tibial Cartilage Degeneration Score* 3 Zone total	Zone 1 (Outside)	Zone 2 (Middle)	Zone 3 (Inside)	Tibial Cartilage Degeneration Width Total (µm)**	Sub (µm)***	Depth Ratio Any Matrix Change# Mean	Zone 2	Medial Tibial Osteophyte Score ##	Measure (µm)	Medial Femur Cartilage Degeneration Score*	Bone Score	Total Joint Score w/o Femur	Total Joint Score
02 Clonidine															
1	R	6.67	5.00	1.67	0.00	1133	800	0.37	0.14	5.00	600	0.67	4.00	11.67	12.33
2	R	6.33	4.33	2.00	0.00	1133	800	0.45	0.44	4.00	550	0.00	3.00	10.33	10.33
3	R	5.33	4.33	1.00	0.00	933	667	0.34	0.05	4.33	563	0.00	3.00	9.67	9.67
4	R	5.00	4.00	1.00	0.00	1000	667	0.34	0.05	4.00	517	0.33	4.00	9.00	9.33
5	R	5.67	4.67	1.00	0.00	1033	683	0.34	0.03	4.67	600	0.00	3.00	10.33	10.33
6	R	6.00	4.00	2.00	0.00	1167	800	0.46	0.36	5.00	600	1.00	3.00	11.00	12.00
7	R	4.67	3.67	1.00	0.00	1033	567	0.30	0.04	4.67	600	2.33	2.00	9.33	11.67
8	R	7.33	5.00	2.33	0.00	1133	833	0.42	0.27	5.00	783	2.00	3.00	12.33	14.33
9	R	4.00	3.00	1.00	0.00	1067	400	0.27	0.06	3.67	480	0.00	3.00	7.67	7.67
10	R	6.67	4.67	2.00	0.00	1100	767	0.38	0.17	4.67	583	1.33	3.00	11.33	12.67
**Mean**		**5.77**	**4.27**	**1.50**	**0.00**	**1073.33**	**698.33**	**0.37**	**0.16**	**4.50**	**587.67**	**0.77**	**3.10**	**10.27**	**11.03**
**SE**		**0.33**	**0.20**	**0.17**	**0.00**	**23.20**	**42.49**	**0.02**	**0.05**	**0.15**	**25.29**	**0.28**	**0.18**	**0.44**	**0.61**
**ttest to G1**		**0.807**	**0.188**	**0.317**	**0.331**	**0.058**	**0.577**	**0.286**	**0.865**	**0.009**	**0.007**	**0.421**	**0.749**	**0.218**	**0.455**
**%**		**-3%**	**-14%**	**18%**	**100%**	**11%**	**-7%**	**-12%**	**-7%**	**-26%**	**-25%**	**28%**	**3%**	**-12%**	**-8%**

*Cartilage degeneration score=depth (0-5) for each 1/3 then summed (mean of 3 step section)
**Width of any cartilage lesion (mean of 3 step section)
***Width of cartilage degeneration extending >50% of total thickness (mean of 3 step section)
#Mean Lesion depth in µm vs depth to tidemark in center of zone in the Tibial plateau (mean of 3 step section)
##Osteophyte scores 1=small up to 299 µm, 2=medium 300-399 µm, 3=large 400-499 µm, 4=very large 500-599 µm, 5=very large ≥600 µm

3) IA Clonidine (4.5 µg)

Animals treated with IA clonidine (4.5 ug/weekly injection) had significantly decreased depth ratios of any matrix change in zone 2 (72%). Other general pathology parameters were not significantly affected. However, the total joint score was non-significantly decreased by 11% and bone scores were nonsignificantly decreased by 9%. Collagen degeneration, cartilage areas, and growth plate and MCL thicknesses were not significantly affected. All animals had mild subacute synovitis.

Table 48. Histopathology of MMT/MCLT animals treated with clonidine (Group 3; IA delivery)

Treatment Group Animal #	Knee	Medial Tibial Cartilage Degeneration Score* 3 Zone total	Zone 1 (Outside)	Zone 2 (Middle)	Zone 3 (Inside)	Tibial Cartilage Degeneration Width Total (µm)**	Sub (µm)***	Depth Ratio Any Matrix Change# Mean	Zone 2	Medial Tibial Osteophyte Score ##	Measure (µm)	Medial Femur Cartilage Degeneration Score*	Bone Score	Total Joint Score w/o Femur	Total Joint Score
03 A1															
1	R	4.33	3.33	1.00	0.00	1033	567	0.31	0.10	3.00	400	2.33	2.00	7.33	9.67
2	R	5.00	3.33	1.67	0.00	1333	667	0.24	0.01	4.00	530	1.00	3.00	9.00	10.00
3	R	4.33	3.33	1.00	0.00	1233	533	0.28	0.03	3.67	483	0.33	4.00	8.00	8.33
4	R	3.33	2.33	1.00	0.00	1000	400	0.18	0.00	3.33	463	0.00	2.00	6.67	6.67
5	R	5.67	4.00	1.67	0.00	1267	733	0.38	0.20	5.00	700	0.67	3.00	10.67	11.33
6	R	5.00	4.67	0.33	0.00	900	600	0.32	0.02	4.00	517	0.00	3.00	9.00	9.00
7	R	4.00	3.33	0.67	0.00	967	500	0.29	0.03	2.67	397	0.00	4.00	6.67	6.67
8	R	4.33	3.67	0.67	0.00	1100	500	0.30	0.00	3.00	413	1.00	2.00	7.33	8.33
9	R	6.33	4.67	1.67	0.00	967	767	0.34	0.00	3.00	447	0.33	3.00	9.33	9.67
10	R	6.00	4.00	2.00	0.00	1467	800	0.31	0.04	4.67	583	1.00	3.00	10.67	11.67
**Mean**		**4.83**	**3.67**	**1.17**	**0.00**	**1126.67**	**606.67**	**0.30**	**0.04**	**3.63**	**493.33**	**0.67**	**2.90**	**8.47**	**9.13**
**SE**		**0.30**	**0.22**	**0.17**	**0.00**	**59.38**	**41.51**	**0.02**	**0.02**	**0.25**	**29.88**	**0.23**	**0.23**	**0.47**	**0.54**
**ttest to G1**		**0.260**	**0.870**	**0.054**	**0.331**	**0.350**	**0.615**	**0.366**	**0.019**	**0.860**	**0.594**	**0.240**	**0.391**	**0.437**	**0.289**
**%**		**14%**	**2%**	**36%**	**100%**	**7%**	**7%**	**10%**	**72%**	**-2%**	**-5%**	**38%**	**9%**	**8%**	**11%**

*Cartilage degeneration score=depth (0-5) for each 1/3 then summed (mean of 3 step section)
**Width of any cartilage lesion (mean of 3 step section)
***Width of cartilage degeneration extending >50% of total thickness (mean of 3 step section)
#Mean Lesion depth in µm vs depth to tidemark in center of zone in the Tibial plateau (mean of 3 step section)
##Osteophyte scores 1=small up to 299 µm, 2=medium 300-399 µm, 3=large 400-499 µm, 4=very large 500-599 µm, 5=very large ≥600 µm

4) IA Triamcinolone Hexacetonide (TH; 0.15 mg)

Animals treated with 0.15 mg/week IA TH had significantly increased cartilage degeneration in zone 1 (28%), which contributed to a significant 35% increase in the 3-zone total. The widths of total (23%) and substantial (33%) cartilage degeneration were also significantly increased, as were the depth ratio of any matrix change, both the mean (38%) and in zone 2 (137%). Osteophyte scores (51%) and measurements (42%) were significantly decreased. The total joint score was non-significantly increased by 10%. The width of severe collagen degeneration was significantly increased by 176% and the widths of mild (44%) and minimal (53%) degeneration were significantly decreased. Corresponding significant increases were seen in the combined widths of severe+marked+moderate+mild (60%), severe+marked+moderate (114%), and severe+marked (153%) degeneration. Combined widths of mild+minimal (50%) and moderate+mild+minimal (46%) degeneration were significantly decreased. The percent area of viable cartilage matrix and the percent area of any matrix were significantly decreased by 37 and 199%, respectively. Growth plate and MCL thickness were both significantly decreased. All animals had hypocellular fibrous repair and marked bone marrow hypocellularity.

Table 49. Histopathology of MMT/MCLT animals treated with Triamcinolone Hexacetonide (Group 4; IA delivery)

Treatment Group Animal #	Knee	Medial Tibial Cartilage Degeneration Score* 3 Zone total	Zone 1 (Outside)	Zone 2 (Middle)	Zone 3 (Inside)	Tibial Cartilage Degeneration Width Total (µm)**	Sub (µm)***	Depth Ratio Any Matrix Change# Mean	Zone 2	Medial Tibial Osteophyte Score ##	Measure (µm)	Medial Femur Cartilage Degeneration Score*	Bone Score	Total Joint Score w/o Femur	Total Joint Score
04 B															
1	R	7.67	5.00	2.67	0.00	1933	933	0.48	0.45	2.00	333	1.33	4.00	9.67	11.00
2	R	10.00	5.00	4.00	1.00	2000	1100	0.71	1.00	2.67	383	5.67	4.00	12.67	18.33
3	R	7.00	4.67	2.00	0.33	1633	800	0.45	0.30	1.33	267	1.33	4.00	8.33	9.67
4	R	7.67	5.00	2.67	0.00	1367	867	0.38	0.14	2.00	313	1.67	3.00	9.67	11.33
5	R	7.67	5.00	2.67	0.00	1400	900	0.39	0.15	3.00	400	0.67	4.00	10.67	11.33
6	R	6.00	4.67	1.33	0.00	1333	700	0.34	0.02	0.00	0	1.00	3.00	6.00	7.00
7	R	8.00	5.00	3.00	0.00	1367	967	0.51	0.53	2.33	350	3.00	4.00	10.33	13.33
8	R	9.00	5.00	3.33	0.67	1600	933	0.52	0.54	2.33	343	2.33	4.00	11.33	13.67
9	R	8.00	5.00	3.00	0.00	1133	967	0.47	0.39	1.67	320	3.33	3.00	9.67	13.00
10	R	4.33	3.33	1.00	0.00	1133	467	0.25	0.07	0.00	0	0.00	2.00	4.33	4.33
**Mean**		**7.53**	**4.77**	**2.57**	**0.20**	**1490.00**	**863.33**	**0.45**	**0.36**	**1.73**	**271.00**	**2.03**	**3.50**	**9.27**	**11.30**
**SE**		**0.49**	**0.17**	**0.29**	**0.11**	**94.68**	**55.43**	**0.04**	**0.09**	**0.33**	**46.63**	**0.52**	**0.22**	**0.78**	**1.21**
**ttest to G1**		**0.021**	**0.013**	**0.080**	**0.175**	**0.024**	**0.033**	**0.022**	**0.053**	**0.000**	**0.002**	**0.107**	**0.382**	**0.927**	**0.480**
**%**		**-35%**	**-28%**	**-40%**	**-500%**	**-23%**	**-33%**	**-38%**	**-137%**	**51%**	**42%**	**-91%**	**-9%**	**-1%**	**-10%**

*Cartilage degeneration score=depth (0-5) for each 1/3 then summed (mean of 3 step section)
**Width of any cartilage lesion (mean of 3 step section)
***Width of cartilage degeneration extending >50% of total thickness (mean of 3 step section)
#Mean Lesion depth in µm vs depth to tidemark in center of zone in the Tibial plateau (mean of 3 step section)
##Osteophyte scores 1=small up to 299 µm, 2=medium 300-399 µm, 3=large 400-499 µm, 4=very large 500-599 µm, 5=very large ≥600 µm

5) IA Tacrolimus (15 ng)

Animals treated with 15 ng IA Tacrolimus once a week had significantly decreased cartilage degeneration scores in zone 2 of the medial tibia (42%). The width of total cartilage degeneration was also significantly decreased (12%). Other general pathology parameters were not significantly affected, although the total joint score was non-significantly reduced by 16%. The width of severe collagen degeneration was significantly decreased by 86%. Cartilage areas and growth plate and MCL thickness were not significantly affected. All animals had minimal to mild subacute synovitis.

Animals treated with 30 ng IA Tacrolimus once a week had significantly decreased cartilage degeneration scores in zone 2 of the medial tibia (35%). Other general pathology parameters were not significantly affected, although the total joint score was non-significantly reduced by 13%. The width of severe collagen degeneration was significantly decreased by 87%. Cartilage areas and growth plate and MCL thickness were not significantly affected. All animals had minimal to mild subacute synovitis.

Table 50. Histopathology of MMT/MCLT animals treated with low dose Tacrolimus (Group 5; IA delivery)

Treatment Group Animal #	Knee	Medial Tibial Cartilage Degeneration Score*				Tibial Cartilage Degeneration Width		Depth Ratio Any Matrix Change#		Medial Tibial Osteophyte		Medial Femur Cartilage Degeneration Score*	Bone Score	Total Joint Score w/o Femur	Total Joint Score
		3 Zone total	Zone 1 (Outside)	Zone 2 (Middle)	Zone 3 (Inside)	Total (µm)**	Sub (µm)***	Mean	Zone 2	Score ##	Measure (µm)				
05 C1															
1	R	2.67	2.00	0.67	0.00	1100	267	0.06	0.06	3.00	400	0.00	4.00	5.67	5.67
2	R	3.33	2.33	1.00	0.00	1067	333	0.14	0.02	3.67	467	0.67	2.00	7.00	7.67
3	R	3.33	3.00	0.33	0.00	900	467	0.24	0.00	3.00	443	0.00	2.00	6.33	6.33
4	R	4.33	3.33	1.00	0.00	1067	500	0.26	0.06	3.00	433	1.67	2.00	7.33	9.00
5	R	4.67	4.00	0.67	0.00	1000	533	0.29	0.04	3.33	447	3.67	2.00	8.00	11.67
6	R	5.67	4.00	1.67	0.00	1100	733	0.37	0.17	4.00	500	0.00	3.00	9.67	9.67
7	R	6.00	4.33	1.67	0.00	1133	733	0.31	0.07	1.33	267	0.33	2.00	7.33	7.67
8	R	4.33	3.33	1.00	0.00	1000	567	0.24	0.00	4.67	583	1.00	4.00	9.00	10.00
9	R	6.00	4.33	1.67	0.00	1233	767	0.40	0.22	4.00	500	1.00	3.00	10.00	11.00
10	R	4.00	3.00	1.00	0.00	1000	467	0.22	0.00	3.00	427	0.33	2.00	7.00	7.33
Mean		4.43	3.37	1.07	0.00	1060.00	536.67	0.25	0.06	3.30	446.67	0.87	2.60	7.73	8.60
SE		0.37	0.26	0.15	0.00	28.89	53.39	0.03	0.02	0.28	25.81	0.36	0.27	0.45	0.63
ttest to G1		0.110	0.397	0.024	0.331	0.045	0.229	0.102	0.059	0.512	0.533	0.645	0.118	0.115	0.140
%		21%	10%	42%	100%	12%	17%	23%	59%	7%	5%	19%	19%	16%	16%

*Cartilage degeneration score=depth (0-5) for each 1/3 then summed (mean of 3 step section)
**Width of any cartilage lesion (mean of 3 step section)
***Width of cartilage degeneration extending >50% of total thickness (mean of 3 step section)
#Mean Lesion depth in µm vs depth to tidemark in center of zone in the Tibial plateau (mean of 3 step section)
##Osteophyte scores 1=small up to 299 µm, 2=medium 300-399 µm, 3=large 400-499 µm, 4=very large 500-599 µm, 5=very large ≥600 µm

6) IA Tacrolimus (30 ng)

Table 51. Histopathology of MMT/MCLT animals treated with high dose Tacrolimus (Group 6; IA delivery)

Treatment Group Animal #	Knee	Medial Tibial Cartilage Degeneration Score*				Tibial Cartilage Degeneration Width		Depth Ratio Any Matrix Change#		Medial Tibial Osteophyte		Medial Femur Cartilage Degeneration Score*	Bone Score	Total Joint Score w/o Femur	Total Joint Score
		3 Zone total	Zone 1 (Outside)	Zone 2 (Middle)	Zone 3 (Inside)	Total (µm)**	Sub (µm)***	Mean	Zone 2	Score ##	Measure (µm)				
06 C2															
1	R	4.67	4.00	0.67	0.00	1067	600	0.33	0.11	4.33	550	0.00	3.00	9.00	9.00
2	R	4.67	3.67	1.00	0.00	1000	567	0.30	0.05	3.33	433	0.33	4.00	8.00	8.33
3	R	5.00	4.00	1.00	0.00	1067	700	0.27	0.11	3.00	400	1.00	2.00	8.00	9.00
4	R	4.00	3.00	1.00	0.00	900	433	0.20	0.01	2.67	383	1.00	2.00	6.67	7.67
5	R	4.33	2.67	1.67	0.00	1333	533	0.30	0.15	3.00	413	2.00	2.00	7.33	9.33
6	R	3.67	2.67	1.00	0.00	1067	367	0.19	0.02	2.00	307	0.00	1.00	5.67	5.67
7	R	6.00	4.00	1.67	0.33	1233	733	0.36	0.13	5.00	600	1.00	3.00	11.00	12.00
8	R	3.67	2.67	1.00	0.00	967	467	0.23	0.01	1.67	273	0.67	2.00	5.33	6.00
9	R	6.00	4.33	1.67	0.00	1133	667	0.27	0.05	3.67	497	1.00	4.00	9.67	10.67
10	R	5.33	4.00	1.33	0.00	1133	700	0.35	0.13	5.00	717	1.00	3.00	10.33	11.33
Mean		4.73	3.50	1.20	0.03	1090.00	576.67	0.28	0.08	3.37	457.33	0.80	2.60	8.10	8.90
SE		0.27	0.21	0.11	0.03	40.08	39.77	0.02	0.02	0.36	42.89	0.19	0.31	0.60	0.66
ttest to G1		0.197	0.565	0.046	1.000	0.126	0.393	0.176	0.080	0.669	0.793	0.389	0.146	0.279	0.233
%		15%	6%	35%	0%	10%	11%	15%	50%	6%	3%	25%	19%	12%	13%

*Cartilage degeneration score=depth (0-5) for each 1/3 then summed (mean of 3 step section)
**Width of any cartilage lesion (mean of 3 step section)
***Width of cartilage degeneration extending >50% of total thickness (mean of 3 step section)
#Mean Lesion depth in µm vs depth to tidemark in center of zone in the Tibial plateau (mean of 3 step section)
##Osteophyte scores 1=small up to 299 µm, 2=medium 300-399 µm, 3=large 400-499 µm, 4=very large 500-599 µm, 5=very large ≥600 µm

7) Fluocinolone (15 ng)

Animals treated with 15 ng IA Fluocinolone once a week were not significantly different from the controls for any parameter. All animals had minimal to moderate subacute synovitis.

Table 52. Histopathology of MMT/MCLT animals treated with Fluocinolone (Group 7; IA delivery)

Treatment Group Animal #	Knee	Medial Tibial Cartilage Degeneration Score*				Tibial Cartilage Degeneration Width		Depth Ratio Any Matrix Change#		Medial Tibial Osteophyte		Medial Femur Cartilage Degeneration Score*	Bone Score	Total Joint Score w/o Femur	Total Joint Score
		3 Zone total	Zone 1 (Outside)	Zone 2 (Middle)	Zone 3 (Inside)	Total (µm)**	Sub (µm)***	Mean	Zone 2	Score ##	Measure (µm)				
07 D															
1	R	6.00	5.00	1.00	0.00	1033	633	0.34	0.03	3.67	480	0.00	2.00	9.67	9.67
2	R	4.67	3.67	1.00	0.00	1033	500	0.33	0.02	2.67	367	1.00	2.00	7.33	8.33
3	R	5.33	4.33	1.00	0.00	933	533	0.33	0.00	2.33	390	0.00	3.00	7.67	7.67
4	R	4.67	3.67	1.00	0.00	1033	533	0.30	0.00	3.00	427	1.67	3.00	7.67	9.33
5	R	5.67	4.33	1.33	0.00	1200	667	0.35	0.04	4.33	550	0.00	2.00	10.00	10.00
6	R	5.33	4.00	1.33	0.00	1033	700	0.30	0.01	4.00	513	1.00	3.00	9.33	10.33
7	R	7.67	5.00	2.33	0.33	1100	867	0.40	0.18	3.00	453	2.33	4.00	10.67	13.00
8	R	3.33	2.33	1.00	0.00	1067	267	0.08	0.05	3.67	483	1.00	3.00	7.00	8.00
9	R	5.33	4.00	1.00	0.33	1367	633	0.37	0.11	5.00	600	0.00	3.00	10.33	10.33
10	R	6.33	4.67	1.67	0.00	1267	733	0.39	0.18	4.00	500	1.00	3.00	10.33	11.33
Mean		5.43	4.10	1.27	0.07	1106.67	606.67	0.32	0.06	3.57	476.33	0.80	2.80	9.00	9.80
SE		0.36	0.25	0.14	0.04	41.51	51.11	0.03	0.02	0.26	22.41	0.25	0.20	0.45	0.51
ttest to G1		0.812	0.392	0.081	0.556	0.189	0.635	0.809	0.056	1.000	0.884	0.453	0.227	0.850	0.668
%		3%	-10%	31%	-100%	9%	7%	3%	59%	0%	-1%	25%	13%	2%	4%

*Cartilage degeneration score=depth (0-5) for each 1/3 then summed (mean of 3 step section)
**Width of any cartilage lesion (mean of 3 step section)
***Width of cartilage degeneration extending >50% of total thickness (mean of 3 step section)
#Mean Lesion depth in µm vs depth to tidemark in center of zone in the Tibial plateau (mean of 3 step section)
##Osteophyte scores 1=small up to 299 µm, 2=medium 300-399 µm, 3=large 400-499 µm, 4=very large 500-599 µm, 5=very large ≥600 µm

8) Curcumin (30 µg)

Animals treated with 30 µg IA Curcumin once a week had significantly decreased cartilage degeneration scores in zone 2 of the medial tibia (40%). The depth ratio of any matrix change in zone 2 was also significantly decreased (69%). Other general pathology parameters were not significantly affected, although the total joint score was non-significantly reduced by 7%. Collagen degeneration, cartilage areas, and growth plate and MCL thicknesses were not significantly affected. All animals had minimal to moderate subacute synovitis.

Table 53. Histopathology of MMT/MCLT animals treated with Curcumin (Group 8; IA delivery)

Treatment Group Animal #	Knee	Medial Tibial Cartilage Degeneration Score*				Tibial Cartilage Degeneration Width		Depth Ratio Any Matrix Change#		Medial Tibial Osteophyte		Medial Femur Cartilage Degeneration Score*	Bone Score	Total Joint Score w/o Femur	Total Joint Score
		3 Zone total	Zone 1 (Outside)	Zone 2 (Middle)	Zone 3 (Inside)	Total (µm)**	Sub (µm)***	Mean	Zone 2	Score ##	Measure (µm)				
08 E															
1	R	4.00	3.67	0.33	0.00	900	533	0.24	0.00	3.00	427	1.00	3.00	7.00	8.00
2	R	5.33	4.00	1.00	0.33	1600	633	0.30	0.01	2.33	360	1.33	3.00	7.67	9.00
3	R	6.67	4.67	1.67	0.33	1333	700	0.34	0.02	4.00	517	0.67	4.00	10.67	11.33
4	R	5.67	4.33	1.33	0.00	1000	667	0.30	0.00	3.00	433	1.00	4.00	8.67	9.67
5	R	3.67	3.33	0.33	0.00	1000	400	0.31	0.02	3.00	417	6.00	3.00	6.67	12.67
6	R	4.00	3.00	1.00	0.00	967	467	0.20	0.02	2.33	340	0.33	4.00	6.33	6.67
7	R	5.33	4.00	1.33	0.00	1067	633	0.32	0.08	4.00	533	0.67	2.00	9.33	10.00
8	R	6.33	4.67	1.67	0.00	1067	733	0.36	0.09	4.33	550	4.33	4.00	10.67	15.00
9	R	4.67	3.67	1.00	0.00	967	500	0.27	0.03	3.33	447	0.00	2.00	8.00	8.00
10	R	3.33	2.00	1.33	0.00	1267	333	0.21	0.20	1.00	220	1.00	1.00	4.33	5.33
Mean		4.90	3.73	1.10	0.07	1116.67	560.00	0.29	0.05	3.03	424.33	1.63	3.00	7.93	9.57
SE		0.36	0.26	0.15	0.04	68.90	42.40	0.02	0.02	0.31	31.60	0.61	0.33	0.63	0.91
ttest to G1		0.324	1.000	0.030	0.556	0.332	0.304	0.233	0.023	0.220	0.286	0.400	0.637	0.220	0.599
%		13%	0%	40%	-100%	8%	14%	13%	69%	15%	10%	-53%	6%	13%	7%

*Cartilage degeneration score=depth (0-5) for each 1/3 then summed (mean of 3 step section)
**Width of any cartilage lesion (mean of 3 step section)
***Width of cartilage degeneration extending >50% of total thickness (mean of 3 step section)
#Mean Lesion depth in µm vs depth to tidemark in center of zone in the Tibial plateau (mean of 3 step section)
##Osteophyte scores 1=small up to 299 µm, 2=medium 300-399 µm, 3=large 400-499 µm, 4=very large 500-599 µm, 5=very large ≥600 µm

Conclusions

The histopathology results in this study were the most informative and indicated that treatment with IA Clonidine, Tacrolimus, or Curcumin had minor, sporadically significant beneficial effects on histopathology parameters of medial meniscal tear induced osteoarthritis in rats in conjunction with increased synovitis and evidence of decreased load bearing. Fluocinolone at this dose had no effect and Triamcinolone hexacetonide significantly increased lesion development, in conjunction with decreased growth plate width and MCL thickness, hypocellular fibrous repair, and bone marrow hypocellularity. It is possible that these observations were the result of increased use of the limb (less pain), although the weight bearing measure did not completely reflect this.

No treatment had consistently significant effects on load bearing, but this may have been due to the measuring weight bearing immediately following IA injections. All IA injections sporadically decreased weight bearing on the injured leg, and to a significant degree compared to the controls. (For this reason results from load bearing are not included here.) Systemic clonidine treatment had minimal, non-significant effects on load bearing at one hour only on days 7 and 21, but was generally unimpressive. Daily clonidine treatment may have given more positive results as the half-life of clonidine is between 6-24 hours. The increased lesion severity relative to the saline treated controls (IA treatment) is a result of IA treatment being compared to SC treatment. IA saline injections consistently decrease lesion severity in this model and the degree of decrease is directly related to frequency and timing of injections post-surgery.

Appendix

Experimental design / drug key

Table 54. Experimental Design and Key for MIA Trial 1.

Group (Termed)	Compound	Dose	Volume (ml/kg)	Route of Admin	Group size (n)
1 (Day 28)	Saline Vehicle Control	5 ml/kg	5	s.c.	10
2	Clonidine	0.1 mg/kg	5	s.c.	10
3	Fluocinolone	2 ug/kg	5	s.c.	10
4	Morphine	6 mg/kg	5	s.c.	10
(Day 07) Histology	Vehicle Saline	5 ml/kg	5	s.c.	3
(Day 14) Histology	Vehicle Saline	5 ml/kg	5	s.c.	3
(Day 21) Histology	Vehicle Saline	5 ml/kg	5	s.c.	3

Table 55. Experimental Design and Key for MIA Trial 2.

Group	Compound	Dose (mg/kg)	Volume (ml/kg)	Route of Admin	Group size (n)
1	Saline Vehicle Control	NA	5	s.c.	10
3	A1 Tacrolimus	0.3	1	i.p.	10
4	A2 Tacrolimus	0.6	1	i.p.	10
5	B Curcumin	50	5	p.o. gavage	10
6	C Fluocinolone	0.01	5	s.c.	10
2	Clonidine Control (systemic)	0.1	5	s.c.	10

Table 56. Experimental Design and Key for MIA Trial 3.

Group	Compound	Dose	Volume	Route of Admin	Group size (n)
1	Saline Vehicle Control	n.a.	30 µl	i.a.	10
2	Clonidine Control (systemic)	100 µg/kg	30 µl	s.c.	10
3	A Clonidine	4.5 µg	30 µl	i.a.	10
4	B Tacrolimus	30 ng	30 µl	i.a.	10
5	C Fluocinolone	15 ng	30 µl	i.a.	10
6	D Meloxicam	100 µg	30 µl	i.a.	10
7	E Tranilast	500 ng	30 µl	i.a.	10
8	F Triamcinolone Hex	150 ug	30 µl	i.a.	10

Table 57. Experimental Design and Key for MMT/MCLT Trial 1.

Group	Compound	Dose	Route of Admin	Group Size (n)
1	Vehicle (Saline)		i.p.	8
2	Morphine	10 mg/kg	i.p.	8
3	A1 Tacrolimus low	0.3 mg/kg	i.p.	8
4	A2 Tacrolimus high	0.6 mg/kg	i.p.	8
5	B1 Fluocinolone low	5 ug/kg	i.p.	8
6	B2 Fluocinolone high	10 ug/kg	i.p.	8
7	C1 Clonidine	100 ug/kg	i.p.	8
8	D1 Alendronate	10 ug/kg	i.p.	8
9	E1 Curcumin	50 mg/kg	p.o.	8
10	F1 Withaferin	50 mg/kg	p.o.	8

Table 58. Experimental Design and Key for MMT/MCLT Trial 2.

Group	Compound	Dose	Volume	Route of Admin	Group Size (n)
1	Saline Vehicle Control	n.a.	30 µl	i.a.	10
2	Clonidine	100 µg/kg	30 µl	s.c.	10
3	Drug A - Clonidine	4.5 µg	30 µl	i.a.	10
4	Drug B - Triamcinolone	0.15 mg	30 µl	i.a.	10
5	Drug C1 - Tacrolimus	15 ng	30 µl	i.a.	10
6	Drug C2 - Tacrolimus	30 ng	30 µl	i.a.	10
7	Drug D - Fluocinolone	15 ng	30 µl	i.a.	10
8	Drug E - Curcumin	30 µg	30 µl	i.a.	10

Weight Bearing Data for MIA Trial 1

Table 59. Individual animal WB score data for MIA Trial 1 Day 7. Scores calculated as indicated in the methods.

Rat #	Treatment	Weight Bearing Score (%)					
		Pre-MIA Baseline	Day 7 Baseline	Day 7 1 hour	Day 7 3 hour	Day 7 5 hour	Day 7 24 hour
14	Vehicle (5 ml/kg, s.c.)	49.5	34.7	33.7	23.5	40.6	28.7
17	Vehicle (5 ml/kg, s.c.)	51.7	15.7	29.4	40.9	32.2	28.8
23	Vehicle (5 ml/kg, s.c.)	48.2	34.9	19.7	16.7	21.5	27.6
30	Vehicle (5 ml/kg, s.c.)	53.0	27.9	43.2	38.4	36.8	32.0
31	Vehicle (5 ml/kg, s.c.)	47.7	36.2	36.1	34.9	42.3	28.2
40	Vehicle (5 ml/kg, s.c.)	50.1	33.2	12.3	29.2	22.4	35.6
41	Vehicle (5 ml/kg, s.c.)	51.7	23.3	18.3	33.2	20.7	31.6
43	Vehicle (5 ml/kg, s.c.)	50.9	30.2	51.5	22.4	40.9	36.4
49	Vehicle (5 ml/kg, s.c.)	50.5	28.8	30.9	35.9	23.1	32.3
57	Vehicle (5 ml/kg, s.c.)	47.9	29.4	43.7	23.6	30.9	34.8
10	Clonidine (100 μg/kg, s.c.)	51.5	36.5	62.3	41.3	41.2	37.2
11	Clonidine (100 μg/kg, s.c.)	49.8	16.7	24.5	49.4	42.1	36.1
18	Clonidine (100 μg/kg, s.c.)	49.6	29.6	44.0	36.7	42.3	36.4
28	Clonidine (100 μg/kg, s.c.)	48.6	21.4	42.8	18.7	37.0	34.6
32	Clonidine (100 μg/kg, s.c.)	49.6	32.2	55.3	38.1	45.0	29.5
35	Clonidine (100 μg/kg, s.c.)	49.2	28.1	54.2	39.4	34.8	30.1
36	Clonidine (100 μg/kg, s.c.)	50.5	30.9	25.6	24.8	24.0	32.6
37	Clonidine (100 μg/kg, s.c.)	48.9	34.7	51.1	50.7	39.4	37.8
44	Clonidine (100 μg/kg, s.c.)	47.1	35.5	54.2	23.5	41.4	38.9
45	Clonidine (100 μg/kg, s.c.)	48.7	29.0	36.7	35.9	36.9	34.2
4	Fluocinolone (2 μg/kg, s.c.)	50.7	24.6	39.8	40.5	32.5	27.9
13	Fluocinolone (2 μg/kg, s.c.)	49.9	29.0	28.5	29.3	16.7	33.5
15	Fluocinolone (2 μg/kg, s.c.)	48.9	32.2	24.8	39.4	50.3	42.9
16	Fluocinolone (2 μg/kg, s.c.)	50.4	36.5	52.6	52.3	47.7	28.4
25	Fluocinolone (2 μg/kg, s.c.)	50.5	33.6	33.5	48.3	24.7	32.8
34	Fluocinolone (2 μg/kg, s.c.)	47.3	16.9	26.5	22.1	29.5	30.6
39	Fluocinolone (2 μg/kg, s.c.)	51.3	21.5	48.8	14.5	14.9	17.6
42	Fluocinolone (2 μg/kg, s.c.)	49.9	35.9	46.2	24.4	43.6	40.6
47	Fluocinolone (2 μg/kg, s.c.)	50.6	31.5	59.0	58.2	44.3	39.9
58	Fluocinolone (2 μg/kg, s.c.)	50.8	29.7	57.5	55.9	31.5	33.6
1	Morphine (6 mg/kg, s.c.)	55.5	37.0	55.7	48.4	40.3	38.8
2	Morphine (6 mg/kg, s.c.)	50.2	17.1	43.3	48.4	44.7	34.3
6	Morphine (6 mg/kg, s.c.)	50.4	33.0	29.3	39.4	33.9	42.7
7	Morphine (6 mg/kg, s.c.)	51.5	34.3	53.1	40.2	33.6	25.9
8	Morphine (6 mg/kg, s.c.)	52.2	31.7	45.1	53.0	31.0	42.9
19	Morphine (6 mg/kg, s.c.)	53.4	29.9	53.0	36.1	42.1	34.9
20	Morphine (6 mg/kg, s.c.)	49.4	24.9	42.2	40.6	47.8	36.4
26	Morphine (6 mg/kg, s.c.)	47.9	28.2	41.0	43.6	46.8	26.1
48	Morphine (6 mg/kg, s.c.)	51.3	22.1	36.9	31.7	38.2	32.6
50	Morphine (6 mg/kg, s.c.)	52.3	34.9	51.5	40.0	30.9	37.5
12	Vehicle (5 ml/kg, s.c.)	52.0	29.3				
22	Vehicle (5 ml/kg, s.c.)	49.7	25.2				
29	Vehicle (5 ml/kg, s.c.)	51.0	32.1				
38	Vehicle (5 ml/kg, s.c.)	47.2	34.4				
46	Vehicle (5 ml/kg, s.c.)	47.1	19.9				
52	Vehicle (5 ml/kg, s.c.)	49.0	22.5				
54	Vehicle (5 ml/kg, s.c.)	48.0	28.8				
55	Vehicle (5 ml/kg, s.c.)	51.3	33.1				
56	Vehicle (5 ml/kg, s.c.)	51.3	34.9				
3	No Treatment	49.7	39.2				
21	No Treatment	50.8	37.6				

Table 60. Individual animal WB score data for MIA Trial 1 Day 14.

Rat #	Treatment	Weight Bearing Score (%) Day 14 Baseline	Day 14 1 hour	Day 14 3 hour	Day 14 5 hour	Day 14 24 hour
14	Vehicle (5 ml/kg, s.c.)	30.2	20.3	12.7	37.5	33.4
17	Vehicle (5 ml/kg, s.c.)	27.5	14.8	26.8	20.3	27.0
23	Vehicle (5 ml/kg, s.c.)	21.5	48.0	34.6	48.2	30.6
30	Vehicle (5 ml/kg, s.c.)	21.1	18.8	24.0	17.6	31.4
31	Vehicle (5 ml/kg, s.c.)	23.2	25.7	38.9	38.4	36.8
40	Vehicle (5 ml/kg, s.c.)	21.5	22.3	36.2	32.4	25.0
41	Vehicle (5 ml/kg, s.c.)	24.6	27.3	33.2	35.0	27.9
43	Vehicle (5 ml/kg, s.c.)	27.3	26.9	33.6	34.4	32.7
49	Vehicle (5 ml/kg, s.c.)	35.0	21.5	16.7	48.2	25.7
57	Vehicle (5 ml/kg, s.c.)	39.3	44.4	50.5	42.5	37.7
10	Clonidine (100 µg/kg, s.c.)	35.4	42.1	49.3	29.4	39.4
11	Clonidine (100 µg/kg, s.c.)	43.6	37.6	46.7	28.0	40.5
18	Clonidine (100 µg/kg, s.c.)	23.0	31.6	20.4	28.1	26.5
28	Clonidine (100 µg/kg, s.c.)	34.9	44.2	14.1	25.8	25.1
32	Clonidine (100 µg/kg, s.c.)	26.5	45.8	46.9	27.1	24.4
35	Clonidine (100 µg/kg, s.c.)	23.8	45.3	38.0	33.4	33.0
36	Clonidine (100 µg/kg, s.c.)	18.8	24.9	47.0	20.9	26.1
37	Clonidine (100 µg/kg, s.c.)	42.8	49.1	28.3	37.8	34.2
44	Clonidine (100 µg/kg, s.c.)	29.5	30.4	26.6	45.7	16.6
45	Clonidine (100 µg/kg, s.c.)	28.7	23.3	34.3	34.5	32.9
4	Fluocinolone (2 µg/kg, s.c.)	44.6	45.5	45.6	42.4	43.5
13	Fluocinolone (2 µg/kg, s.c.)	23.6	10.6	20.9	22.9	34.9
15	Fluocinolone (2 µg/kg, s.c.)	44.0	35.1	37.9	35.1	42.8
16	Fluocinolone (2 µg/kg, s.c.)	46.2	31.4	40.0	40.4	40.1
25	Fluocinolone (2 µg/kg, s.c.)	30.2	44.0	34.7	34.2	29.1
34	Fluocinolone (2 µg/kg, s.c.)	20.5	30.9	20.1	28.2	25.8
39	Fluocinolone (2 µg/kg, s.c.)	31.2	14.4	15.9	15.3	19.1
42	Fluocinolone (2 µg/kg, s.c.)	36.2	27.9	40.4	37.1	33.4
47	Fluocinolone (2 µg/kg, s.c.)	34.1	33.8	39.3	35.9	41.4
58	Fluocinolone (2 µg/kg, s.c.)	26.7	32.9	17.5	48.0	36.3
1	Morphine (6 mg/kg, s.c.)	37.6	40.9	46.8	38.2	32.8
2	Morphine (6 mg/kg, s.c.)	43.3	44.7	48.5	38.6	32.3
6	Morphine (6 mg/kg, s.c.)	38.0	37.3	42.3	47.3	28.9
7	Morphine (6 mg/kg, s.c.)	33.8	30.4	30.4	34.8	30.7
8	Morphine (6 mg/kg, s.c.)	41.5	44.7	43.4	44.4	35.2
19	Morphine (6 mg/kg, s.c.)	23.3	47.1	28.6	24.7	34.3
20	Morphine (6 mg/kg, s.c.)	32.2	35.2	35.2	32.0	23.7
26	Morphine (6 mg/kg, s.c.)	17.4	46.9	41.0	39.3	28.0
48	Morphine (6 mg/kg, s.c.)	30.4	36.8	32.7	42.9	25.9
50	Morphine (6 mg/kg, s.c.)	26.7	41.4	26.9	31.9	29.4
12	Vehicle (5 ml/kg, s.c.)	16.6				
22	Vehicle (5 ml/kg, s.c.)	24.2				
29	Vehicle (5 ml/kg, s.c.)	42.5				
38	Vehicle (5 ml/kg, s.c.)	46.4				
46	Vehicle (5 ml/kg, s.c.)	38.4				
52	Vehicle (5 ml/kg, s.c.)	32.4				
54	Vehicle (5 ml/kg, s.c.)					
55	Vehicle (5 ml/kg, s.c.)					
56	Vehicle (5 ml/kg, s.c.)					
3	No Treatment					
21	No Treatment					

Table 61. Individual animal WB score data for MIA Trial 1 Day 21.

Rat #	Treatment	Weight Bearing Score (%) Day 21 Baseline	Day 21 1 hour	Day 21 3 hour	Day 21 5 hour	Day 21 24 hour
14	Vehicle (5 ml/kg, s.c.)	28.8	30.3	20.6	27.5	36.5
17	Vehicle (5 ml/kg, s.c.)	26.1	35.0	26.8	39.3	26.1
23	Vehicle (5 ml/kg, s.c.)	21.8	19.2	30.7	22.9	24.9
30	Vehicle (5 ml/kg, s.c.)	22.7	27.6	17.6	16.4	24.8
31	Vehicle (5 ml/kg, s.c.)	27.4	40.9	34.7	31.8	26.2
40	Vehicle (5 ml/kg, s.c.)	15.5	23.4	25.4	27.8	38.5
41	Vehicle (5 ml/kg, s.c.)	20.8	29.3	24.7	21.2	32.4
43	Vehicle (5 ml/kg, s.c.)	25.4	27.7	37.0	26.1	32.1
49	Vehicle (5 ml/kg, s.c.)	22.3	20.6	25.0	24.8	17.1
57	Vehicle (5 ml/kg, s.c.)	33.4	30.5	38.2	37.0	28.7
10	Clonidine (100 μg/kg, s.c.)	30.0	40.2	39.9	25.1	32.7
11	Clonidine (100 μg/kg, s.c.)	22.9	33.4	28.8	31.9	36.1
18	Clonidine (100 μg/kg, s.c.)	27.5	35.7	29.3	21.0	31.1
28	Clonidine (100 μg/kg, s.c.)	28.2	42.1	39.3	34.0	27.2
32	Clonidine (100 μg/kg, s.c.)	26.8	35.8	30.7	32.6	30.9
35	Clonidine (100 μg/kg, s.c.)	32.6	47.5	44.5	34.6	23.0
36	Clonidine (100 μg/kg, s.c.)	24.2	42.4	18.7	27.1	23.2
37	Clonidine (100 μg/kg, s.c.)	36.6	48.3	46.1	38.9	40.6
44	Clonidine (100 μg/kg, s.c.)	26.2	49.0	46.5	38.4	35.5
45	Clonidine (100 μg/kg, s.c.)	29.5	45.8	28.5	27.0	21.0
4	Fluocinolone (2 μg/kg, s.c.)	38.8	45.9	47.5	31.7	36.3
13	Fluocinolone (2 μg/kg, s.c.)	29.7	31.0	27.8	23.8	23.4
15	Fluocinolone (2 μg/kg, s.c.)	22.6	33.4	24.6	25.4	33.1
16	Fluocinolone (2 μg/kg, s.c.)	43.0	41.6	46.4	30.8	43.8
25	Fluocinolone (2 μg/kg, s.c.)	41.8	27.9	31.5	22.2	30.5
34	Fluocinolone (2 μg/kg, s.c.)	23.4	31.4	21.2	25.7	26.2
39	Fluocinolone (2 μg/kg, s.c.)	21.6	26.9	18.5	19.9	27.3
42	Fluocinolone (2 μg/kg, s.c.)	33.2	41.6	31.2	29.0	20.3
47	Fluocinolone (2 μg/kg, s.c.)	29.2	45.6	29.3	28.2	36.8
58	Fluocinolone (2 μg/kg, s.c.)	35.7	43.6	26.0	34.0	24.1
1	Morphine (6 mg/kg, s.c.)	24.5	43.6	42.9	30.0	30.5
2	Morphine (6 mg/kg, s.c.)	37.2	45.1	42.6	43.3	29.3
6	Morphine (6 mg/kg, s.c.)	30.5	48.2	39.8	23.8	25.8
7	Morphine (6 mg/kg, s.c.)	32.2	48.2	40.1	21.6	32.9
8	Morphine (6 mg/kg, s.c.)	37.2	38.8	44.5	39.4	37.3
19	Morphine (6 mg/kg, s.c.)	29.1	36.8	34.2	28.2	23.3
20	Morphine (6 mg/kg, s.c.)	39.1	40.2	26.2	31.4	35.6
26	Morphine (6 mg/kg, s.c.)	37.3	35.1	32.2	25.0	27.4
48	Morphine (6 mg/kg, s.c.)	37.7	37.4	42.1	30.1	16.2
50	Morphine (6 mg/kg, s.c.)	21.1	47.3	40.1	26.7	34.2
12	Vehicle (5 ml/kg, s.c.)	41.0				
22	Vehicle (5 ml/kg, s.c.)	25.5				
29	Vehicle (5 ml/kg, s.c.)	44.6				
38	Vehicle (5 ml/kg, s.c.)					
46	Vehicle (5 ml/kg, s.c.)					
52	Vehicle (5 ml/kg, s.c.)					
54	Vehicle (5 ml/kg, s.c.)					
55	Vehicle (5 ml/kg, s.c.)					
56	Vehicle (5 ml/kg, s.c.)					
3	No Treatment	26.0				
21	No Treatment	19.5				

Table 62. Individual animal WB score data for MIA Trial 1 Day 28.

Rat #	Treatment	Weight Bearing Score (%)				
		Day 28 Baseline	Day 28 1 hour	Day 28 3 hour	Day 28 5 hour	Day 28 24 hour
14	Vehicle (5 ml/kg, s.c.)	26.7	31.6	24.6	23.2	31.0
17	Vehicle (5 ml/kg, s.c.)	31.2	19.7	25.6	29.4	29.9
23	Vehicle (5 ml/kg, s.c.)	26.1	28.4	40.3	23.2	21.9
30	Vehicle (5 ml/kg, s.c.)	26.5	25.9	21.8	30.4	35.0
31	Vehicle (5 ml/kg, s.c.)	32.2	47.1	26.3	34.5	30.1
40	Vehicle (5 ml/kg, s.c.)	29.1	27.3	18.9	24.9	24.5
41	Vehicle (5 ml/kg, s.c.)	26.3	20.4	31.4	32.6	28.6
43	Vehicle (5 ml/kg, s.c.)	35.8	30.0	30.2	35.9	28.3
49	Vehicle (5 ml/kg, s.c.)	29.1	28.1	36.3	35.4	22.8
57	Vehicle (5 ml/kg, s.c.)	34.1	40.0	38.0	40.2	34.1
10	Clonidine (100 μg/kg, s.c.)	30.2	48.7	41.1	39.4	34.1
11	Clonidine (100 μg/kg, s.c.)	32.4	34.5	35.5	31.5	31.9
18	Clonidine (100 μg/kg, s.c.)	34.0	43.1	27.1	30.0	29.1
28	Clonidine (100 μg/kg, s.c.)	34.1	33.9	28.5	17.4	26.6
32	Clonidine (100 μg/kg, s.c.)	30.2	44.5	42.5	40.7	31.5
35	Clonidine (100 μg/kg, s.c.)	27.9	43.8	40.4	30.2	31.1
36	Clonidine (100 μg/kg, s.c.)	30.4	41.8	49.6	35.1	26.8
37	Clonidine (100 μg/kg, s.c.)	31.5	42.8	47.5	43.3	35.6
44	Clonidine (100 μg/kg, s.c.)	32.6	34.6	37.3	40.9	26.9
45	Clonidine (100 μg/kg, s.c.)	40.0	49.0	34.7	39.1	27.4
4	Fluocinolone (2 μg/kg, s.c.)	41.1	47.5	35.3	42.2	25.5
13	Fluocinolone (2 μg/kg, s.c.)	34.3	29.3	29.7	26.1	31.9
15	Fluocinolone (2 μg/kg, s.c.)	24.4	30.8	19.8	26.4	23.3
16	Fluocinolone (2 μg/kg, s.c.)	35.9	43.6	43.9	34.2	31.7
25	Fluocinolone (2 μg/kg, s.c.)	38.8	33.6	38.7	37.4	36.7
34	Fluocinolone (2 μg/kg, s.c.)	32.9	29.9	30.0	20.9	16.9
39	Fluocinolone (2 μg/kg, s.c.)	35.0	19.8	27.3	30.8	24.4
42	Fluocinolone (2 μg/kg, s.c.)	34.4	30.8	44.1	43.8	37.8
47	Fluocinolone (2 μg/kg, s.c.)	32.8	35.9	24.5	37.8	27.2
58	Fluocinolone (2 μg/kg, s.c.)	26.1	29.9	26.2	36.7	30.2
1	Morphine (6 mg/kg, s.c.)	38.5	48.9	42.1	37.6	20.7
2	Morphine (6 mg/kg, s.c.)	34.8	47.8	41.7	36.3	27.0
6	Morphine (6 mg/kg, s.c.)	25.5	45.4	35.9	35.9	31.2
7	Morphine (6 mg/kg, s.c.)	23.8	44.5	39.7	33.7	31.6
8	Morphine (6 mg/kg, s.c.)	35.1	44.3	38.6	43.0	29.7
19	Morphine (6 mg/kg, s.c.)	31.4	43.9	41.8	30.9	31.5
20	Morphine (6 mg/kg, s.c.)	40.6	42.4	30.1	33.2	32.5
26	Morphine (6 mg/kg, s.c.)	38.4	43.1	45.7	35.0	28.1
48	Morphine (6 mg/kg, s.c.)	30.5	41.5	41.2	26.7	22.7
50	Morphine (6 mg/kg, s.c.)	28.1	26.5	25.5	30.8	32.3
12	Vehicle (5 ml/kg, s.c.)					
22	Vehicle (5 ml/kg, s.c.)					
29	Vehicle (5 ml/kg, s.c.)					
38	Vehicle (5 ml/kg, s.c.)					
46	Vehicle (5 ml/kg, s.c.)					
52	Vehicle (5 ml/kg, s.c.)					
54	Vehicle (5 ml/kg, s.c.)					
55	Vehicle (5 ml/kg, s.c.)					
56	Vehicle (5 ml/kg, s.c.)					
3	No Treatment					
21	No Treatment					

Digital Randall-Selitto Data for MIA Trial 2

Table 63. Individual animal joint compression data for MIA 2 Day 7.

		Day 7Joint Compression (g)									
		Baseline		1 hour		3 hour		5 hour		24 hour	
Rat #	Treatment	Ipsi.	Contra.	Ipsi.	Contra.	Ipsi.	Contra.	Ipsi.	Contra.	Ipsi.	Contra.
9	Vehicle (saline, 5 ml/kg, s.c.)	103.5	198.9	96.9	593.7	140.8	450.8	179.4	445.4	110.3	226.9
14	Vehicle (saline, 5 ml/kg, s.c.)	96.6	556.1	143.6	401.9	139.9	424.6	86.2	473.8	70.0	581.8
15	Vehicle (saline, 5 ml/kg, s.c.)	119.1	497.8	156.9	455.3	69.5	307.6	161.9	493.9	100.7	510.1
19	Vehicle (saline, 5 ml/kg, s.c.)	163.2	338.9	199.2	260.4	148.2	534.1	97.7	508.4	162.2	253.1
31	Vehicle (saline, 5 ml/kg, s.c.)	111.5	381.1	120.6	399.4	159.6	434.4	89.3	393.8	62.7	401.5
34	Vehicle (saline, 5 ml/kg, s.c.)	136.0	465.4	119.2	600.0	137.6	381.0	133.8	414.9	103.8	460.0
52	Vehicle (saline, 5 ml/kg, s.c.)	152.1	456.3	177.0	439.4	166.8	312.0	130.2	376.5	123.7	383.9
64	Vehicle (saline, 5 ml/kg, s.c.)	76.3	246.8	136.1	255.5	109.3	306.9	86.1	201.5	87.6	283.9
76	Vehicle (saline, 5 ml/kg, s.c.)	90.8	500.9	156.7	471.3	110.1	458.5	101.6	596.0	134.4	440.1
77	Vehicle (saline, 5 ml/kg, s.c.)	174.7	482.9	168.3	306.4	158.6	408.4	152.6	476.8	129.3	444.9
1	Compound A (0.3 mg/kg, i.p.)	109.1	377.3	170.2	498.6	108.5	333.5	183.9	355.8	162.2	241.0
4	Compound A (0.3 mg/kg, i.p.)	154.0	373.6	97.8	267.4	218.4	335.6	103.2	262.2	49.1	489.3
7	Compound A (0.3 mg/kg, i.p.)	61.6	288.7	132.0	225.2	153.1	266.9	133.7	292.6	156.6	460.1
11	Compound A (0.3 mg/kg, i.p.)	164.2	360.9	108.0	266.6	133.2	353.6	107.3	265.7	106.3	334.0
17	Compound A (0.3 mg/kg, i.p.)	128.8	370.6	173.7	293.1	159.2	315.4	126.0	426.4	110.1	378.3
20	Compound A (0.3 mg/kg, i.p.)	91.1	374.0	113.1	469.0	138.8	424.4	133.4	456.5	170.0	282.1
23	Compound A (0.3 mg/kg, i.p.)	121.1	329.0	95.9	431.4	165.8	398.7	208.2	376.6	86.3	409.4
28	Compound A (0.3 mg/kg, i.p.)	97.2	366.0	126.5	462.2	104.6	232.2	131.2	321.4	171.2	480.4
59	Compound A (0.3 mg/kg, i.p.)	177.3	403.2	110.0	333.5	116.2	465.3	135.6	405.2	165.9	470.6
60	Compound A (0.3 mg/kg, i.p.)	114.5	419.0	197.9	417.5	358.7	409.3	143.2	233.7	139.7	339.1
6	Compound A (0.6 mg/kg, i.p.)	102.2	482.7	232.5	548.8	149.9	600.0	207.6	317.7	126.7	404.9
27	Compound A (0.6 mg/kg, i.p.)	135.6	324.5	163.6	338.1	168.1	342.0	86.4	345.4	98.8	266.5
45	Compound A (0.6 mg/kg, i.p.)	172.5	416.6	134.7	446.5	118.9	289.1	74.8	510.0	110.0	378.0
49	Compound A (0.6 mg/kg, i.p.)	111.4	264.3	168.1	383.6	178.0	355.8	145.0	398.9	91.0	418.5
51	Compound A (0.6 mg/kg, i.p.)	126.6	429.4	135.2	405.1	184.3	381.9	200.6	322.9	104.1	225.3
56	Compound A (0.6 mg/kg, i.p.)	162.4	410.5	143.8	424.1	170.5	253.6	182.7	219.0	92.5	380.1
57	Compound A (0.6 mg/kg, i.p.)	143.8	410.5	208.5	376.9	146.5	515.3	73.5	400.4	159.9	428.4
71	Compound A (0.6 mg/kg, i.p.)	95.8	397.0	181.0	211.5	218.7	293.9	122.9	362.9	56.9	342.7
73	Compound A (0.6 mg/kg, i.p.)	87.2	437.4	166.7	447.5	130.6	529.2	85.4	233.9	194.2	265.1
75	Compound A (0.6 mg/kg, i.p.)	73.7	558.1	131.0	579.6	181.9	259.9	182.2	354.8	139.8	484.2
5	Compound B (50 mg/kg, p.o.)	169.4	264.7	157.6	566.8	147.9	295.6	62.2	416.3	73.8	284.9
10	Compound B (50 mg/kg, p.o.)	109.7	312.1	172.2	453.1	206.6	419.5	171.2	255.3	140.4	210.8
21	Compound B (50 mg/kg, p.o.)	177.7	457.8	346.4	455.5	103.3	194.9	141.7	320.9	177.6	452.7
30	Compound B (50 mg/kg, p.o.)	61.7	499.2	110.4	322.1	181.8	433.7	129.3	374.4	127.5	352.4
32	Compound B (50 mg/kg, p.o.)	129.3	323.6	127.7	374.6	120.8	312.6	105.3	450.5	186.7	421.4
35	Compound B (50 mg/kg, p.o.)	137.9	402.7	239.6	380.0	264.8	446.0	193.8	382.2	118.9	427.2
37	Compound B (50 mg/kg, p.o.)	123.2	508.4	240.0	443.7	264.2	371.7	156.2	380.9	110.2	486.6
53	Compound B (50 mg/kg, p.o.)	116.6	331.8	58.3	484.0	252.1	354.1	173.2	471.7	121.8	317.1
68	Compound B (50 mg/kg, p.o.)	77.7	338.4	170.9	412.7	74.8	369.1	171.6	264.0	149.1	298.0
74	Compound B (50 mg/kg, p.o.)	99.5	309.0	222.9	223.5	166.9	415.4	57.4	290.9	116.2	361.4
2	Compound C (0.01 mg/kg, s.c.)	131.1	392.8	128.9	600.0	323.6	351.2	161.5	361.4	174.8	275.4
3	Compound C (0.01 mg/kg, s.c.)	178.9	279.9	89.6	488.3	109.0	324.4	107.0	442.1	91.1	421.2
42	Compound C (0.01 mg/kg, s.c.)	110.9	246.6	104.3	514.7	79.5	298.9	134.5	277.5	68.5	328.4
46	Compound C (0.01 mg/kg, s.c.)	92.1	516.4	98.9	446.9	121.4	463.2	145.1	470.9	122.5	341.7
48	Compound C (0.01 mg/kg, s.c.)	139.1	464.1	66.4	307.1	95.9	437.8	193.7	274.6	93.9	522.7
58	Compound C (0.01 mg/kg, s.c.)	123.9	400.1	83.2	303.7	151.5	420.8	130.0	244.0	181.6	381.9
63	Compound C (0.01 mg/kg, s.c.)	63.7	320.4	104.7	292.1	105.3	332.1	223.8	153.3	140.2	337.9
65	Compound C (0.01 mg/kg, s.c.)	79.2	215.1	268.3	443.7	239.2	303.0	109.2	422.6	50.1	373.5
66	Compound C (0.01 mg/kg, s.c.)	117.4	459.4	176.5	307.0	165.2	325.6	132.0	367.4	116.9	310.8
69	Compound C (0.01 mg/kg, s.c.)	156.3	395.2	293.8	485.9	55.7	242.2	154.1	380.5	74.4	360.8
8	Clonidine (0.1 mg/kg, s.c.)	162.1	346.8	502.7	473.5	189.4	398.7	247.7	492.3	136.4	321.7
22	Clonidine (0.1 mg/kg, s.c.)	124.2	357.7	350.1	464.1	496.3	600.0	281.4	281.6	114.3	345.6
26	Clonidine (0.1 mg/kg, s.c.)	134.2	233.3	412.5	443.4	441.4	361.8	133.4	393.3	160.1	305.4
33	Clonidine (0.1 mg/kg, s.c.)	94.4	253.3	370.7	422.6	407.9	492.2	167.3	400.2	71.9	335.8
40	Clonidine (0.1 mg/kg, s.c.)	172.2	206.6	292.9	522.5	466.7	451.7	250.5	404.3	158.2	386.1
41	Clonidine (0.1 mg/kg, s.c.)	100.8	273.6	335.7	403.7	411.0	268.8	221.7	333.7	106.5	475.2
47	Clonidine (0.1 mg/kg, s.c.)	118.0	417.7	223.2	530.0	370.8	477.6	157.5	317.2	101.5	397.5
50	Clonidine (0.1 mg/kg, s.c.)	80.6	416.2	410.8	600.0	246.6	354.3	217.2	290.9	175.8	366.6
61	Clonidine (0.1 mg/kg, s.c.)	71.5	365.8	506.9	567.5	211.2	399.5	108.0	489.5	78.2	404.7
62	Clonidine (0.1 mg/kg, s.c.)	140.7	398.9	489.5	536.5	365.2	516.4	108.0	232.1	156.7	289.2

Table 64. Individual animal joint compression data for MIA 2 Day 14.

		Day 14 Joint Compression (g)									
		Baseline		1 hour		3 hour		5 hour		24 hour	
Rat #	Treatment	Ipsi.	Contra.	Ipsi.	Contra.	Ipsi.	Contra.	Ipsi.	Contra.	Ipsi.	Contra.
9	Vehicle (saline, 5 ml/kg, s.c.)	87.3	470.0	96.1	484.8	113.4	449.5	52.8	384.5	101.2	531.9
14	Vehicle (saline, 5 ml/kg, s.c.)	99.9	536.5	56.8	492.2	88.9	441.5	115.5	503.6	129.1	333.3
15	Vehicle (saline, 5 ml/kg, s.c.)	107.7	393.5	116.5	368.4	99.0	348.2	58.9	425.7	41.1	518.4
19	Vehicle (saline, 5 ml/kg, s.c.)	135.4	401.3	71.4	309.2	68.4	341.2	77.0	400.1	32.7	462.6
31	Vehicle (saline, 5 ml/kg, s.c.)	61.0	491.1	108.0	558.3	119.1	444.4	99.2	401.5	136.5	411.4
34	Vehicle (saline, 5 ml/kg, s.c.)	92.4	402.4	60.8	443.0	124.9	412.7	65.8	444.9	52.5	532.5
52	Vehicle (saline, 5 ml/kg, s.c.)	93.5	419.0	122.7	413.9	103.0	368.1	111.9	311.9	76.3	432.7
64	Vehicle (saline, 5 ml/kg, s.c.)	94.7	506.7	86.9	406.7	107.0	293.9	81.3	290.3	56.6	427.0
76	Vehicle (saline, 5 ml/kg, s.c.)	92.7	427.1	123.7	351.0	76.0	544.0	48.9	400.2	49.7	508.4
77	Vehicle (saline, 5 ml/kg, s.c.)	113.5	589.5	107.6	438.0	109.8	505.7	50.1	295.5	60.3	363.8
1	Compound A (0.3 mg/kg, i.p.)	176.6	469.1	60.4	450.1	155.7	455.7	127.6	292.9	104.6	405.0
4	Compound A (0.3 mg/kg, i.p.)	78.8	462.9	90.4	401.1	76.1	311.8	54.0	498.0	103.4	461.9
7	Compound A (0.3 mg/kg, i.p.)	67.3	358.1	128.1	363.1	82.0	393.6	56.6	340.0	66.3	415.4
11	Compound A (0.3 mg/kg, i.p.)	127.8	595.8	123.3	389.0	128.6	428.9	148.2	387.8	136.5	501.8
17	Compound A (0.3 mg/kg, i.p.)	49.3	391.4	72.9	517.1	216.4	282.1	195.7	422.2	150.9	450.0
20	Compound A (0.3 mg/kg, i.p.)	118.4	463.3	122.8	365.8	79.6	238.5	137.8	515.5	57.4	294.0
23	Compound A (0.3 mg/kg, i.p.)	110.7	364.5	126.8	366.9	99.5	423.6	89.1	411.5	43.7	362.1
28	Compound A (0.3 mg/kg, i.p.)	106.0	342.0	171.4	480.6	76.7	403.3	52.2	342.9	50.2	430.4
59	Compound A (0.3 mg/kg, i.p.)	113.3	361.9	102.7	305.4	90.9	257.0	75.0	243.9	120.6	299.6
60	Compound A (0.3 mg/kg, i.p.)	104.1	499.9	71.4	355.6	114.1	445.4	81.1	566.3	92.8	415.7
6	Compound A (0.6 mg/kg, i.p.)	115.8	569.2	73.9	519.0	71.8	289.2	108.5	201.8	39.1	420.5
27	Compound A (0.6 mg/kg, i.p.)	110.8	319.2	137.7	543.8	99.4	435.2	107.2	432.6	90.7	467.8
45	Compound A (0.6 mg/kg, i.p.)	91.3	436.6	69.2	509.5	141.9	329.4	90.5	436.0	155.5	408.5
49	Compound A (0.6 mg/kg, i.p.)	54.5	425.1	65.8	319.2	42.5	341.9	99.5	456.1	152.3	376.6
51	Compound A (0.6 mg/kg, i.p.)	92.5	436.2	87.9	560.0	87.0	347.6	75.5	288.2	52.0	443.2
56	Compound A (0.6 mg/kg, i.p.)	152.8	529.1	66.8	435.2	73.1	319.9	78.1	355.4	69.2	248.0
57	Compound A (0.6 mg/kg, i.p.)	73.6	571.7	62.3	456.1	53.5	240.7	108.7	397.7	133.3	330.6
71	Compound A (0.6 mg/kg, i.p.)	88.0	360.8	99.7	359.1	87.8	600.0	49.1	555.6	120.3	302.1
73	Compound A (0.6 mg/kg, i.p.)	103.2	534.1	104.5	346.5	119.9	342.0	57.6	320.2	87.5	417.2
75	Compound A (0.6 mg/kg, i.p.)	90.0	465.1	122.5	500.6	86.9	419.7	123.9	362.4	85.7	471.1
5	Compound B (50 mg/kg, p.o.)	48.8	439.4	126.4	392.5	94.7	344.9	53.7	457.7	49.8	453.9
10	Compound B (50 mg/kg, p.o.)	122.0	386.6	73.8	600.0	158.7	370.5	120.1	484.1	108.2	472.1
21	Compound B (50 mg/kg, p.o.)	55.4	300.8	74.0	420.2	94.4	412.4	72.6	331.6	89.8	354.3
30	Compound B (50 mg/kg, p.o.)	95.9	383.7	79.9	382.7	125.2	353.5	63.2	256.2	66.6	444.5
32	Compound B (50 mg/kg, p.o.)	97.1	411.7	112.9	470.6	74.4	347.9	76.7	401.6	116.2	483.8
35	Compound B (50 mg/kg, p.o.)	128.1	378.6	228.8	352.9	164.0	384.0	129.5	306.0	103.4	425.8
37	Compound B (50 mg/kg, p.o.)	119.4	264.7	109.4	394.4	57.8	336.9	123.2	408.1	51.4	450.7
53	Compound B (50 mg/kg, p.o.)	91.6	411.3	141.2	303.0	91.0	310.1	91.5	292.2	55.8	477.2
68	Compound B (50 mg/kg, p.o.)	91.7	505.0	84.7	332.6	123.0	285.3	51.3	344.6	59.2	474.0
74	Compound B (50 mg/kg, p.o.)	99.1	519.3	103.1	508.5	67.0	320.9	60.4	428.8	67.6	374.1
2	Compound C (0.01 mg/kg, s.c.)	70.0	395.1	132.2	421.8	73.3	372.4	110.1	309.8	89.3	309.5
3	Compound C (0.01 mg/kg, s.c.)	99.6	378.8	137.1	349.5	93.0	327.3	99.4	222.3	116.7	348.4
42	Compound C (0.01 mg/kg, s.c.)	87.0	288.9	138.3	397.3	78.9	414.2	74.8	353.3	123.0	351.5
46	Compound C (0.01 mg/kg, s.c.)	82.0	364.5	78.1	402.5	143.7	343.5	107.9	402.1	71.1	419.6
48	Compound C (0.01 mg/kg, s.c.)	134.3	320.2	105.3	333.2	105.0	511.1	110.2	330.1	85.7	427.4
58	Compound C (0.01 mg/kg, s.c.)	78.9	296.1	162.1	393.4	103.8	383.0	110.4	262.0	132.8	381.3
63	Compound C (0.01 mg/kg, s.c.)	68.9	250.3	97.7	325.3	69.6	236.7	104.4	265.6	90.4	412.1
65	Compound C (0.01 mg/kg, s.c.)	75.5	384.6	102.0	379.0	107.2	307.6	62.7	378.9	119.8	432.8
66	Compound C (0.01 mg/kg, s.c.)	98.3	426.5	67.8	250.4	151.7	310.2	100.2	372.2	60.6	444.9
69	Compound C (0.01 mg/kg, s.c.)	128.7	351.3	85.9	260.1	98.9	350.5	103.9	261.3	102.4	360.1
8	Clonidine (0.1 mg/kg, s.c.)	66.6	382.2	270.1	449.0	409.9	342.5	104.9	350.2	45.2	289.5
22	Clonidine (0.1 mg/kg, s.c.)	122.9	407.1	464.7	473.5	205.1	375.5	122.5	415.3	92.0	268.2
26	Clonidine (0.1 mg/kg, s.c.)	71.9	430.8	219.7	394.8	130.3	305.6	114.3	405.0	52.0	348.5
33	Clonidine (0.1 mg/kg, s.c.)	107.1	600.0	419.7	388.1	269.6	410.2	109.4	451.1	115.3	344.6
40	Clonidine (0.1 mg/kg, s.c.)	79.6	553.9	357.7	456.3	261.6	370.7	130.0	466.3	134.8	365.5
41	Clonidine (0.1 mg/kg, s.c.)	112.6	372.5	318.0	385.5	275.4	273.5	166.3	415.0	115.3	527.6
47	Clonidine (0.1 mg/kg, s.c.)	82.0	347.8	179.8	284.9	85.9	437.3	44.9	308.0	40.9	385.3
50	Clonidine (0.1 mg/kg, s.c.)	85.2	487.7	229.0	439.0	195.9	359.2	133.3	294.4	109.1	470.2
61	Clonidine (0.1 mg/kg, s.c.)	44.3	408.1	296.4	391.9	250.5	338.4	114.8	561.3	63.9	375.3
62	Clonidine (0.1 mg/kg, s.c.)	112.9	600.0	434.8	352.5	344.6	300.5	77.7	483.0	46.8	276.5

Table 65. Individual animal joint compression data for MIA 2 Day 21.

		Day 21 Joint Compression (g)									
		Baseline		1 hour		3 hour		5 hour		24 hour	
**Rat #**	**Treatment**	Ipsi.	Contra.	Ipsi.	Contra.	Ipsi.	Contra.	Ipsi.	Contra.	Ipsi.	Contra.
**9**	Vehicle (saline, 5 ml/kg, s.c.)	165.1	547.0	67.3	315.2	101.7	286.8	72.8	211.0	128.8	449.3
**14**	Vehicle (saline, 5 ml/kg, s.c.)	160.5	600.0	364.0	600.0	111.8	402.2	155.7	468.6	289.5	392.1
**15**	Vehicle (saline, 5 ml/kg, s.c.)	79.2	553.1	309.0	600.0	234.5	357.9	375.9	235.7	184.1	366.1
**19**	Vehicle (saline, 5 ml/kg, s.c.)	113.1	569.5	186.2	600.0	67.5	520.5	371.2	422.4	188.6	392.0
**31**	Vehicle (saline, 5 ml/kg, s.c.)	230.4	418.9	121.1	531.2	233.3	534.6	183.9	489.8	165.1	541.3
**34**	Vehicle (saline, 5 ml/kg, s.c.)	115.4	514.9	292.0	600.0	227.4	219.6	154.0	278.6	229.5	513.1
**52**	Vehicle (saline, 5 ml/kg, s.c.)	112.3	398.9	67.8	408.0	188.6	600.0	46.8	408.2	172.7	533.1
**64**	Vehicle (saline, 5 ml/kg, s.c.)	160.8	533.2	286.8	305.2	183.5	296.0	135.9	223.5	186.2	424.5
**76**	Vehicle (saline, 5 ml/kg, s.c.)	242.4	600.0	600.0	600.0	279.8	544.0	175.9	552.3	187.1	387.9
**77**	Vehicle (saline, 5 ml/kg, s.c.)	203.8	600.0	380.6	528.0	223.6	501.1	177.0	321.9	161.9	401.4
**1**	Compound A (0.3 mg/kg, i.p.)	118.6	324.3	261.6	374.4	204.2	471.6	53.7	284.4	188.1	331.9
**4**	Compound A (0.3 mg/kg, i.p.)	136.6	519.2	185.5	600.0	360.4	502.3	399.1	412.7	250.7	334.2
**7**	Compound A (0.3 mg/kg, i.p.)	123.5	564.6	427.7	597.4	489.5	566.3	316.1	338.4	171.0	328.8
**11**	Compound A (0.3 mg/kg, i.p.)	184.1	552.3	327.4	460.2	72.4	318.5	351.7	334.3	62.0	280.3
**17**	Compound A (0.3 mg/kg, i.p.)	254.4	422.3	224.1	600.0	317.5	395.7	294.7	334.6	169.6	405.4
**20**	Compound A (0.3 mg/kg, i.p.)	204.1	587.8	181.5	551.0	223.0	324.1	281.0	312.5	193.8	430.9
**23**	Compound A (0.3 mg/kg, i.p.)	205.0	414.2	118.9	455.6	98.0	286.3	78.4	225.6	152.7	376.6
**28**	Compound A (0.3 mg/kg, i.p.)	161.5	600.0	74.8	343.9	211.3	317.1	136.3	398.1	149.5	502.1
**59**	Compound A (0.3 mg/kg, i.p.)	126.7	600.0	336.8	538.8	146.6	403.8	298.6	194.8	111.2	211.3
**60**	Compound A (0.3 mg/kg, i.p.)	182.1	491.0	99.9	600.0	308.1	441.3	423.0	479.3	59.3	596.4
**6**	Compound A (0.6 mg/kg, i.p.)	215.1	522.6	542.6	574.4	260.5	600.0	373.5	460.0	298.1	390.4
**27**	Compound A (0.6 mg/kg, i.p.)	96.5	343.7	144.0	454.1	197.3	400.5	213.6	263.1	240.8	512.0
**45**	Compound A (0.6 mg/kg, i.p.)	125.9	435.8	127.8	468.3	290.8	525.6	107.8	474.0	189.7	461.7
**49**	Compound A (0.6 mg/kg, i.p.)	81.1	581.4	33.2	342.3	386.8	371.6	270.8	321.6	130.7	338.1
**51**	Compound A (0.6 mg/kg, i.p.)	89.3	600.0	311.4	491.9	117.4	450.3	316.6	411.9	143.6	547.0
**56**	Compound A (0.6 mg/kg, i.p.)	124.2	410.6	198.8	355.9	239.5	376.0	147.1	338.7	216.4	484.2
**57**	Compound A (0.6 mg/kg, i.p.)	98.6	510.1	159.8	582.5	137.0	264.9	109.3	480.8	221.5	388.7
**71**	Compound A (0.6 mg/kg, i.p.)	99.7	600.0	360.7	472.2	173.1	416.7	205.1	486.8	78.3	348.1
**73**	Compound A (0.6 mg/kg, i.p.)	179.1	600.0	307.6	454.6	118.4	319.5	86.8	408.0	111.4	420.9
**75**	Compound A (0.6 mg/kg, i.p.)	146.8	390.4	334.0	260.3	184.4	271.4	141.3	281.9	113.4	373.6
**5**	Compound B (50 mg/kg, p.o.)	133.7	600.0	198.8	408.1	269.4	405.5	347.4	399.9	269.3	420.4
**10**	Compound B (50 mg/kg, p.o.)	367.6	600.0	269.4	478.3	122.8	558.3	341.1	448.5	242.6	472.9
**21**	Compound B (50 mg/kg, p.o.)	272.2	590.0	285.7	541.1	318.9	600.0	303.4	442.3	226.0	419.1
**30**	Compound B (50 mg/kg, p.o.)	77.0	600.0	311.0	338.8	437.6	511.0	221.3	350.8	183.5	300.0
**32**	Compound B (50 mg/kg, p.o.)	169.4	455.8	193.8	461.5	192.8	395.3	68.0	335.0	190.3	434.6
**35**	Compound B (50 mg/kg, p.o.)	170.4	502.6	196.0	480.5	169.2	332.2	135.4	461.2	209.1	345.9
**37**	Compound B (50 mg/kg, p.o.)	113.0	411.3	217.1	600.0	149.4	436.0	146.7	281.0	188.7	458.5
**53**	Compound B (50 mg/kg, p.o.)	218.0	506.8	263.0	344.6	432.6	515.0	368.3	511.0	79.5	387.0
**68**	Compound B (50 mg/kg, p.o.)	301.7	559.6	359.5	421.6	187.1	390.2	234.2	600.0	211.2	382.9
**74**	Compound B (50 mg/kg, p.o.)	183.8	322.6	336.8	480.9	208.7	302.7	300.6	374.0	128.9	455.3
**2**	Compound C (0.01 mg/kg, s.c.)	91.4	289.6	266.0	600.0	142.0	516.3	111.5	275.3	98.8	258.6
**3**	Compound C (0.01 mg/kg, s.c.)	114.1	433.8	113.6	600.0	153.9	399.1	161.9	262.8	119.7	257.2
**42**	Compound C (0.01 mg/kg, s.c.)	110.0	422.4	477.5	600.0	192.1	323.1	112.6	271.9	188.1	413.1
**46**	Compound C (0.01 mg/kg, s.c.)	188.7	440.6	291.8	532.6	211.0	600.0	227.4	240.7	238.8	356.0
**48**	Compound C (0.01 mg/kg, s.c.)	135.3	600.0	228.9	424.5	145.1	414.4	161.8	365.3	167.3	306.1
**58**	Compound C (0.01 mg/kg, s.c.)	162.0	300.6	266.9	592.4	211.2	600.0	155.7	222.9	211.6	353.0
**63**	Compound C (0.01 mg/kg, s.c.)	137.0	276.3	178.5	166.9	96.5	228.3	198.6	215.8	124.1	379.4
**65**	Compound C (0.01 mg/kg, s.c.)	174.8	600.0	237.1	562.0	95.9	277.4	188.4	378.9	192.0	247.0
**66**	Compound C (0.01 mg/kg, s.c.)	55.4	299.4	123.7	362.7	86.2	242.9	139.9	353.1	160.5	238.1
**69**	Compound C (0.01 mg/kg, s.c.)	127.0	351.6	223.8	249.5	99.3	178.2	76.4	256.1	172.7	421.5
**8**	Clonidine (0.1 mg/kg, s.c.)	198.7	600.0	457.9	495.5	389.9	505.2	182.7	357.0	151.1	503.0
**22**	Clonidine (0.1 mg/kg, s.c.)	228.1	435.5	508.5	600.0	567.0	600.0	321.5	600.0	161.7	375.9
**26**	Clonidine (0.1 mg/kg, s.c.)	201.0	600.0	537.1	468.2	600.0	600.0	374.7	568.6	153.6	519.8
**33**	Clonidine (0.1 mg/kg, s.c.)	189.1	509.7	531.0	481.7	600.0	600.0	416.4	596.4	149.9	485.9
**40**	Clonidine (0.1 mg/kg, s.c.)	183.5	433.3	78.8	378.4	339.8	583.1	452.1	600.0	272.2	460.4
**41**	Clonidine (0.1 mg/kg, s.c.)	210.7	594.5	259.9	252.0	116.2	448.3	87.1	333.6	104.0	253.3
**47**	Clonidine (0.1 mg/kg, s.c.)	176.7	404.7	600.0	600.0	187.5	267.7	91.9	266.3	127.4	498.4
**50**	Clonidine (0.1 mg/kg, s.c.)	187.9	408.4	183.2	421.2	255.9	327.1	107.6	424.8	86.4	371.0
**61**	Clonidine (0.1 mg/kg, s.c.)	189.1	600.0	470.7	600.0	461.2	600.0	305.5	409.6	189.7	539.6
**62**	Clonidine (0.1 mg/kg, s.c.)	136.9	527.1	360.9	520.2	83.5	463.8	269.2	208.5	116.0	381.1

Table 66. Individual animal joint compression data for MIA 2 Day 28.

		Day 28 Joint Compression (g)									
		Baseline		1 hour		3 hour		5 hour		24 hour	
Rat #	Treatment	Ipsi.	Contra.	Ipsi.	Contra.	Ipsi.	Contra.	Ipsi.	Contra.	Ipsi.	Contra.
9	Vehicle (saline, 5 ml/kg, s.c.)	110.4	250.9	100.8	480.3	74.8	477.9	74.8	589.5	79.1	450.6
14	Vehicle (saline, 5 ml/kg, s.c.)	119.9	479.5	164.1	600.0	106.7	473.1	167.6	507.8	143.7	469.5
15	Vehicle (saline, 5 ml/kg, s.c.)	176.0	522.3	122.0	448.3	162.7	499.1	92.7	333.2	156.6	600.0
19	Vehicle (saline, 5 ml/kg, s.c.)	166.6	432.7	99.2	481.0	99.9	437.3	111.0	523.8	106.3	600.0
31	Vehicle (saline, 5 ml/kg, s.c.)	134.3	561.4	122.4	332.3	104.6	570.1	141.5	459.1	174.9	568.3
34	Vehicle (saline, 5 ml/kg, s.c.)	132.5	388.7	142.0	557.7	159.2	408.9	112.2	600.0	107.9	343.6
52	Vehicle (saline, 5 ml/kg, s.c.)	111.6	600.0	108.0	600.0	120.5	329.6	149.7	449.3	119.5	556.8
64	Vehicle (saline, 5 ml/kg, s.c.)	132.5	377.7	123.6	505.2	121.1	499.0	133.1	368.6	88.8	358.0
76	Vehicle (saline, 5 ml/kg, s.c.)	168.5	521.3	140.7	593.9	155.9	393.5	134.9	600.0	154.4	384.1
77	Vehicle (saline, 5 ml/kg, s.c.)	104.6	452.0	136.3	600.0	79.5	521.6	139.9	352.9	75.1	600.0
1	Compound A (0.3 mg/kg, i.p.)	116.7	507.3	117.0	482.7	109.4	436.2	162.2	461.1	127.2	386.7
4	Compound A (0.3 mg/kg, i.p.)	194.6	416.3	119.5	505.4	122.5	430.8	127.0	395.8	84.0	398.8
7	Compound A (0.3 mg/kg, i.p.)	165.2	307.5	123.0	408.6	189.3	518.5	73.8	347.1	123.2	432.2
11	Compound A (0.3 mg/kg, i.p.)	148.6	547.6	77.7	517.6	126.9	458.8	125.2	411.7	141.7	447.4
17	Compound A (0.3 mg/kg, i.p.)	110.4	265.7	175.5	381.2	114.8	176.6	179.2	535.3	158.1	531.9
20	Compound A (0.3 mg/kg, i.p.)	150.1	600.0	135.0	524.4	136.3	356.8	136.6	411.2	89.4	418.0
23	Compound A (0.3 mg/kg, i.p.)	89.4	463.6	136.6	438.0	123.1	548.7	154.0	311.0	91.9	456.8
28	Compound A (0.3 mg/kg, i.p.)	108.3	529.2	167.8	465.9	136.6	307.7	69.0	319.8	130.0	499.9
59	Compound A (0.3 mg/kg, i.p.)	104.2	521.8	241.2	490.8	126.4	218.9	120.5	457.1	120.1	448.6
60	Compound A (0.3 mg/kg, i.p.)	148.2	502.8	128.9	520.1	114.4	519.1	126.2	372.5	118.6	301.6
6	Compound A (0.6 mg/kg, i.p.)	164.8	348.5	188.6	600.0	176.5	594.6	97.6	573.8	168.9	555.9
27	Compound A (0.6 mg/kg, i.p.)	96.4	287.5	150.4	533.1	130.8	529.5	116.7	392.5	90.9	473.9
45	Compound A (0.6 mg/kg, i.p.)	135.6	544.4	197.6	466.2	230.1	535.1	179.5	452.9	102.3	484.0
49	Compound A (0.6 mg/kg, i.p.)	165.6	355.3	88.2	498.2	122.0	442.5	133.3	540.0	132.1	507.4
51	Compound A (0.6 mg/kg, i.p.)	96.9	501.9	216.4	419.5	117.3	517.2	181.8	491.4	117.9	418.0
56	Compound A (0.6 mg/kg, i.p.)	126.3	600.0	93.3	558.9	144.0	422.3	121.6	562.0	105.3	466.0
57	Compound A (0.6 mg/kg, i.p.)	133.9	386.6	166.6	560.8	141.9	373.0	186.7	387.7	135.1	336.8
71	Compound A (0.6 mg/kg, i.p.)	92.5	480.6	117.2	269.8	141.2	399.4	148.0	248.9	131.0	509.8
73	Compound A (0.6 mg/kg, i.p.)	100.7	524.4	81.3	386.3	179.2	363.0	125.9	329.2	102.9	600.0
75	Compound A (0.6 mg/kg, i.p.)	108.8	529.2	173.7	366.8	122.8	351.5	138.5	439.9	98.8	571.4
5	Compound B (50 mg/kg, p.o.)	124.3	580.5	112.1	519.7	159.5	554.4	148.2	568.2	136.7	573.9
10	Compound B (50 mg/kg, p.o.)	159.3	414.2	88.4	444.9	227.8	469.4	154.4	513.9	163.9	504.7
21	Compound B (50 mg/kg, p.o.)	130.0	554.9	120.3	576.0	92.9	479.6	166.9	495.1	151.4	600.0
30	Compound B (50 mg/kg, p.o.)	142.7	355.3	108.6	552.2	104.2	367.4	57.3	535.3	138.8	374.3
32	Compound B (50 mg/kg, p.o.)	100.0	334.3	108.0	540.7	124.1	452.0	91.2	269.3	126.2	473.2
35	Compound B (50 mg/kg, p.o.)	115.5	600.0	128.5	435.8	129.1	389.8	131.6	417.8	117.9	448.4
37	Compound B (50 mg/kg, p.o.)	92.8	475.5	171.3	428.3	177.5	308.7	102.7	332.3	132.7	499.5
53	Compound B (50 mg/kg, p.o.)	148.9	532.9	157.9	279.0	171.8	505.5	62.7	262.2	114.0	600.0
68	Compound B (50 mg/kg, p.o.)	132.5	581.3	199.9	495.1	132.7	469.3	149.2	270.8	140.9	471.5
74	Compound B (50 mg/kg, p.o.)	102.4	600.0	133.7	352.9	107.2	343.5	96.6	289.8	111.6	539.5
2	Compound C (0.01 mg/kg, s.c.)	111.0	411.3	149.4	324.1	130.7	285.3	132.6	350.2	124.9	495.0
3	Compound C (0.01 mg/kg, s.c.)	122.4	442.6	167.2	387.3	89.3	456.8	103.5	351.1	99.9	169.3
42	Compound C (0.01 mg/kg, s.c.)	84.7	600.0	155.0	336.3	146.3	418.4	105.6	427.7	117.1	493.4
46	Compound C (0.01 mg/kg, s.c.)	95.6	239.3	128.6	447.2	117.7	288.3	158.7	312.2	168.2	414.6
48	Compound C (0.01 mg/kg, s.c.)	146.4	527.3	175.8	413.2	149.9	427.1	157.8	330.6	136.0	434.7
58	Compound C (0.01 mg/kg, s.c.)	121.9	436.3	160.8	377.3	177.7	321.8	59.2	180.7	105.0	331.1
63	Compound C (0.01 mg/kg, s.c.)	91.2	312.2	105.3	215.4	117.7	252.4	104.4	394.5	136.8	299.6
65	Compound C (0.01 mg/kg, s.c.)	127.1	289.3	121.0	392.4	151.8	398.3	83.5	378.7	96.7	448.6
66	Compound C (0.01 mg/kg, s.c.)	93.0	426.5	114.3	388.0	111.1	361.4	171.8	490.7	113.9	456.8
69	Compound C (0.01 mg/kg, s.c.)	143.1	438.0	127.0	429.1	149.4	317.0	151.6	238.6	80.1	511.3
8	Clonidine (0.1 mg/kg, s.c.)	82.8	600.0	296.0	361.1	197.1	576.0	116.6	341.5	135.3	312.4
22	Clonidine (0.1 mg/kg, s.c.)	136.4	462.7	485.6	600.0	559.0	489.0	362.4	588.1	145.8	415.5
26	Clonidine (0.1 mg/kg, s.c.)	74.3	555.6	243.2	600.0	425.5	588.5	215.3	488.8	119.2	478.2
33	Clonidine (0.1 mg/kg, s.c.)	110.4	565.5	407.8	600.0	268.5	544.7	165.0	170.7	124.0	429.4
40	Clonidine (0.1 mg/kg, s.c.)	150.7	600.0	493.1	600.0	225.8	600.0	323.6	407.3	129.5	459.7
41	Clonidine (0.1 mg/kg, s.c.)	125.8	563.0	512.1	550.3	449.8	523.4	445.7	542.5	77.2	380.5
47	Clonidine (0.1 mg/kg, s.c.)	75.3	446.3	185.2	457.0	265.3	541.8	318.7	398.8	140.9	391.1
50	Clonidine (0.1 mg/kg, s.c.)	86.7	430.0	390.0	535.4	277.5	393.2	239.2	393.8	111.6	516.1
61	Clonidine (0.1 mg/kg, s.c.)	84.8	600.0	228.0	465.3	140.3	482.9	208.3	585.2	128.0	535.9
62	Clonidine (0.1 mg/kg, s.c.)	102.9	595.3	249.2	385.6	243.8	533.1	156.2	373.7	92.8	534.6

Digital Randall-Selitto Data for MIA Trial 3

Table 67. Individual joint compression data for MIA Trial 3 Day 7.

Joint Compression (g)											
		Day 7Joint Compression (g)									
		Baseline		1 hour		3 hour		5 hour		24 hour	
Rat #	Treatment	Ipsi.	Contra.	Ipsi.	Contra.	Ipsi.	Contra.	Ipsi.	Contra.	Ipsi.	Contra.
3	Vehicle (saline, 30 µl, i.a.)	91.4	482.7	119.9	412.4	81.3	486.9	97.3	443.8	101.9	433.6
15	Vehicle (saline, 30 µl, i.a.)	66.5	414.6	97.8	500.0	92.7	342.5	92.6	500.0	99.7	373.0
27	Vehicle (saline, 30 µl, i.a.)	116.4	375.3	82.8	433.1	117.9	395.2	89.8	391.5	103.9	460.4
32	Vehicle (saline, 30 µl, i.a.)	112.1	500.0	90.6	393.3	97.0	459.1	288.5	459.9	86.4	380.0
34	Vehicle (saline, 30 µl, i.a.)	109.8	366.0	88.4	352.4	83.6	456.6	83.3	305.9	97.5	379.4
56	Vehicle (saline, 30 µl, i.a.)	100.5	399.1	111.4	316.3	111.0	477.7	86.1	404.2	101.2	348.0
72	Vehicle (saline, 30 µl, i.a.)	118.6	402.8	82.4	411.4	101.4	310.0	107.3	335.2	113.0	424.7
78	Vehicle (saline, 30 µl, i.a.)	97.5	372.3	94.4	426.2	112.1	374.1	90.4	334.6	72.4	431.9
85	Vehicle (saline, 30 µl, i.a.)	123.2	337.1	88.2	433.2	70.3	343.9	78.3	404.1	114.5	452.9
88	Vehicle (saline, 30 µl, i.a.)	106.3	333.9	77.7	344.8	63.7	472.6	87.4	377.9	92.0	430.3
8	Compound A (4.5 µg, i.a.)	98.5	422.7	114.9	317.0	75.8	443.1	129.8	394.3	111.1	438.5
10	Compound A (4.5 µg, i.a.)	106.9	451.2	110.8	407.2	73.8	409.8	90.8	319.6	117.6	396.7
20	Compound A (4.5 µg, i.a.)	82.2	484.4	91.9	500.0	113.7	399.4	121.2	393.6	101.9	436.4
26	Compound A (4.5 µg, i.a.)	112.2	452.6	161.5	330.7	126.9	419.5	104.9	317.6	94.3	500.0
33	Compound A (4.5 µg, i.a.)	116.5	464.9	111.5	423.3	82.3	407.4	111.4	324.1	95.1	500.0
36	Compound A (4.5 µg, i.a.)	101.0	344.1	99.6	375.5	75.0	340.9	84.2	453.6	107.2	387.5
40	Compound A (4.5 µg, i.a.)	119.1	435.6	138.7	420.9	131.5	451.7	99.6	353.4	136.8	500.0
57	Compound A (4.5 µg, i.a.)	72.2	468.8	153.1	420.9	139.9	357.5	99.4	490.6	100.9	488.3
66	Compound A (4.5 µg, i.a.)	109.9	364.9	117.6	297.6	135.2	437.3	111.6	489.7	113.8	500.0
74	Compound A (4.5 µg, i.a.)	121.5	407.6	121.6	428.9	106.6	446.3	138.7	230.6	89.3	498.7
13	Compound B (30 ng, i.a.)	73.3	479.1	107.7	414.6	125.5	354.6	128.5	369.1	84.0	421.4
17	Compound B (30 ng, i.a.)	113.0	401.1	81.8	386.5	84.5	422.9	109.2	352.4	107.4	375.6
23	Compound B (30 ng, i.a.)	91.5	404.5	97.1	298.4	78.2	445.3	104.8	328.5	89.2	351.2
24	Compound B (30 ng, i.a.)	101.3	476.7	128.1	340.1	101.4	365.2	122.0	369.2	99.1	485.3
41	Compound B (30 ng, i.a.)	116.9	500.0	111.2	230.2	147.8	371.0	114.5	380.6	95.4	458.9
43	Compound B (30 ng, i.a.)	119.4	417.8	85.6	370.4	74.9	334.6	90.8	430.8	109.7	356.3
50	Compound B (30 ng, i.a.)	83.6	360.2	94.1	445.7	108.0	273.0	107.7	207.1	106.0	319.5
77	Compound B (30 ng, i.a.)	107.3	350.7	71.4	426.9	68.0	422.6	110.2	347.4	86.2	404.5
82	Compound B (30 ng, i.a.)	122.0	417.8	113.0	406.7	113.7	468.7	107.7	462.6	114.6	470.1
90	Compound B (30 ng, i.a.)	110.2	380.6	102.6	285.9	135.8	420.1	113.8	342.9	113.0	459.7
25	Compound C (15 ng, i.a.)	93.7	481.2	103.6	500.0	82.9	500.0	109.4	366.8	104.2	500.0
28	Compound C (15 ng, i.a.)	110.3	367.7	85.7	249.2	77.0	351.5	82.2	397.1	105.3	473.7
39	Compound C (15 ng, i.a.)	76.3	446.2	194.2	409.8	182.1	500.0	137.1	417.1	100.1	500.0
49	Compound C (15 ng, i.a.)	85.0	372.3	116.7	383.2	116.5	456.8	111.2	294.3	97.6	443.1
58	Compound C (15 ng, i.a.)	98.5	288.7	125.5	348.5	190.9	358.7	117.9	425.8	111.1	500.0
70	Compound C (15 ng, i.a.)	107.9	433.5	137.4	330.8	116.6	500.0	113.5	375.7	127.8	463.8
87	Compound C (15 ng, i.a.)	117.6	361.1	95.1	500.0	89.7	500.0	93.6	457.8	105.7	419.0
91	Compound C (15 ng, i.a.)	119.7	361.6	125.9	500.0	83.8	451.6	110.2	439.6	99.4	437.3
95	Compound C (15 ng, i.a.)	122.0	445.1	106.0	321.4	121.3	500.0	76.2	413.1	121.9	381.3
98	Compound C (15 ng, i.a.)	113.3	325.7	89.2	420.6	103.3	432.2	102.2	460.9	74.2	460.2
21	Compound D (0.1 mg, i.a.)	77.5	415.1	121.4	219.0	108.5	500.0	91.7	246.8	77.8	422.3
30	Compound D (0.1 mg, i.a.)	122.3	413.9	132.9	373.0	169.3	284.7	84.9	500.0	95.7	454.4
42	Compound D (0.1 mg, i.a.)	101.6	444.7	107.0	445.9	89.6	426.8	101.9	321.6	125.7	343.4
53	Compound D (0.1 mg, i.a.)	98.6	497.1	109.7	434.9	106.7	355.3	104.6	304.9	129.9	319.7
71	Compound D (0.1 mg, i.a.)	110.3	410.4	112.6	322.2	110.8	379.0	102.8	388.5	100.0	378.8
75	Compound D (0.1 mg, i.a.)	88.4	443.9	109.8	361.1	89.1	363.5	80.1	413.8	105.6	474.5
76	Compound D (0.1 mg, i.a.)	119.9	462.0	98.5	409.4	93.9	333.8	115.5	286.7	115.3	500.0
86	Compound D (0.1 mg, i.a.)	114.0	436.9	94.7	410.7	132.3	454.9	110.8	372.5	115.5	415.9
97	Compound D (0.1 mg, i.a.)	117.8	407.4	116.8	205.4	75.6	363.5	102.7	459.5	99.8	500.0
99	Compound D (0.1 mg, i.a.)	94.1	420.2	104.1	410.4	111.5	436.8	90.9	313.5	80.9	405.9
7	Compound E (500 ng, i.a.)	94.7	393.6	93.4	449.8	85.9	356.5	115.5	356.6	95.3	425.6
11	Compound E (500 ng, i.a.)	102.3	452.8	123.8	256.4	120.6	377.1	129.8	440.9	77.0	361.7
22	Compound E (500 ng, i.a.)	78.7	294.1	97.1	431.9	106.8	328.2	116.4	416.9	116.6	479.0
44	Compound E (500 ng, i.a.)	89.8	415.1	97.3	428.6	97.3	386.1	93.1	312.5	105.8	377.6
46	Compound E (500 ng, i.a.)	115.2	428.7	102.0	466.5	123.7	461.2	110.0	283.7	93.1	305.0
52	Compound E (500 ng, i.a.)	99.1	500.0	103.4	310.7	91.2	337.4	99.8	273.1	111.3	469.0
63	Compound E (500 ng, i.a.)	108.0	318.6	120.8	360.4	147.2	378.1	116.5	328.0	107.0	461.1
68	Compound E (500 ng, i.a.)	122.3	469.9	171.5	386.8	109.0	399.4	99.9	362.8	87.8	500.0
89	Compound E (500 ng, i.a.)	120.0	318.1	142.7	476.1	120.8	303.8	102.5	313.7	92.5	339.1
96	Compound E (500 ng, i.a.)	118.2	500.0	100.5	447.8	127.9	500.0	72.8	414.4	80.3	500.0
14	Compound F (0.15 mg, i.a.)	108.1	487.7	112.1	343.8	96.2	302.8	134.2	405.5	86.9	464.9
29	Compound F (0.15 mg, i.a.)	96.4	422.1	69.7	327.4	97.8	333.7	88.4	371.2	94.1	379.3
45	Compound F (0.15 mg, i.a.)	120.3	433.2	84.7	331.3	129.1	352.9	74.3	332.3	113.5	474.7
48	Compound F (0.15 mg, i.a.)	104.2	475.3	120.1	418.0	103.4	353.1	103.5	329.4	81.3	399.6
51	Compound F (0.15 mg, i.a.)	122.8	455.1	104.9	446.5	139.4	382.0	123.4	286.3	99.0	426.3
54	Compound F (0.15 mg, i.a.)	80.6	445.4	114.8	443.7	132.4	464.8	97.6	500.0	101.6	319.7
59	Compound F (0.15 mg, i.a.)	121.5	413.7	107.8	381.5	108.0	500.0	98.5	363.6	94.5	302.5
73	Compound F (0.15 mg, i.a.)	89.9	432.2	107.0	500.0	103.4	308.8	95.1	388.9	99.6	363.0
80	Compound F (0.15 mg, i.a.)	99.3	367.9	80.0	375.2	104.8	270.8	105.2	498.1	77.0	367.2
94	Compound F (0.15 mg, i.a.)	111.0	388.3	84.7	334.3	106.3	296.6	92.5	406.6	98.2	393.5
1	Clonidine (0.1 mg/kg, s.c.)	122.9	496.9	142.0	500.0	183.8	411.8	177.7	373.1	90.8	463.7
12	Clonidine (0.1 mg/kg, s.c.)	116.1	500.0	274.8	500.0	257.3	500.0	209.3	287.4	108.2	401.5
18	Clonidine (0.1 mg/kg, s.c.)	112.1	397.4	251.7	500.0	369.8	500.0	167.6	225.9	116.0	439.6
38	Clonidine (0.1 mg/kg, s.c.)	108.5	360.0	284.3	500.0	451.1	369.5	179.4	433.5	94.6	439.5
47	Clonidine (0.1 mg/kg, s.c.)	97.4	396.7	251.4	319.5	347.8	500.0	201.8	351.5	115.6	438.0
55	Clonidine (0.1 mg/kg, s.c.)	90.8	500.0	368.7	309.9	211.1	406.2	163.9	422.6	91.9	212.6
61	Clonidine (0.1 mg/kg, s.c.)	118.6	390.9	206.0	459.9	248.0	448.6	163.2	315.9	92.4	356.3
64	Clonidine (0.1 mg/kg, s.c.)	81.3	425.4	290.9	500.0	219.5	500.0	272.4	406.2	127.6	433.1
65	Clonidine (0.1 mg/kg, s.c.)	100.3	490.0	230.0	490.3	235.4	500.0	98.8	419.0	113.5	362.3
81	Clonidine (0.1 mg/kg, s.c.)	104.4	389.2	252.6	411.3	372.3	447.6	109.4	325.3	71.2	500.0

Table 68. Individual joint compression data for MIA Trial 3 Day 14.

Joint Compression (g)											
		Day 14 Joint Compression (g)									
		Baseline		1 hour		3 hour		5 hour		24 hour	
Rat #	Treatment	Ipsi.	Contra.	Ipsi.	Contra.	Ipsi.	Contra.	Ipsi.	Contra.	Ipsi.	Contra.
3	Vehicle (saline, 30 µl, i.a.)	116.5	500.0	79.9	500.0	83.5	316.2	106.8	471.1	101.3	486.8
15	Vehicle (saline, 30 µl, i.a.)	103.6	500.0	82.8	340.8	104.4	329.9	96.1	334.1	119.0	500.0
27	Vehicle (saline, 30 µl, i.a.)	88.4	499.8	85.9	368.0	95.1	471.4	85.0	420.5	77.8	500.0
32	Vehicle (saline, 30 µl, i.a.)	83.9	305.2	106.2	444.1	81.3	432.3	113.9	453.2	81.6	414.0
34	Vehicle (saline, 30 µl, i.a.)	92.0	500.0	74.4	464.5	85.6	429.2	98.6	500.0	78.4	500.0
56	Vehicle (saline, 30 µl, i.a.)	86.2	410.8	92.5	481.8	104.2	412.7	103.9	449.8	122.5	325.4
72	Vehicle (saline, 30 µl, i.a.)	110.9	399.1	100.7	500.0	126.2	324.4	84.2	422.4	106.3	500.0
78	Vehicle (saline, 30 µl, i.a.)	103.6	408.5	75.5	410.4	94.1	500.0	91.9	358.7	92.2	294.7
85	Vehicle (saline, 30 µl, i.a.)	114.0	450.7	108.2	436.4	109.1	458.0	109.8	500.0	115.8	464.4
88	Vehicle (saline, 30 µl, i.a.)	117.9	413.5	103.4	246.2	78.4	316.0	127.4	366.7	111.9	330.5
8	Compound A (4.5 µg, i.a.)	119.9	500.0	97.5	381.2	119.6	500.0	104.1	500.0	126.2	361.4
10	Compound A (4.5 µg, i.a.)	107.0	500.0	85.7	434.8	92.3	427.4	133.3	465.8	115.2	444.1
20	Compound A (4.5 µg, i.a.)	112.8	495.9	106.8	376.2	113.2	393.6	105.8	347.3	90.3	454.1
26	Compound A (4.5 µg, i.a.)	124.9	452.2	110.0	417.1	115.6	323.3	113.7	466.2	107.1	428.2
33	Compound A (4.5 µg, i.a.)	99.3	415.3	81.2	327.9	104.4	362.0	86.4	312.3	101.0	443.2
36	Compound A (4.5 µg, i.a.)	85.7	500.0	117.8	408.9	117.4	371.9	83.4	358.5	103.3	470.1
40	Compound A (4.5 µg, i.a.)	88.5	500.0	115.3	338.1	93.8	352.1	110.1	397.9	116.9	482.2
57	Compound A (4.5 µg, i.a.)	116.2	414.8	105.6	412.2	100.2	432.5	94.7	477.4	75.2	417.1
66	Compound A (4.5 µg, i.a.)	96.4	500.0	98.7	274.1	79.3	418.1	100.0	500.0	116.5	426.5
74	Compound A (4.5 µg, i.a.)	111.7	460.9	268.3	463.0	117.6	401.1	99.5	361.6	107.6	301.3
13	Compound B (30 ng, i.a.)	107.0	361.1	189.6	375.1	107.4	365.5	101.2	395.1	119.1	449.8
17	Compound B (30 ng, i.a.)	116.2	364.3	110.6	500.0	112.1	337.1	91.4	377.1	116.3	500.0
23	Compound B (30 ng, i.a.)	97.4	371.4	96.0	276.7	95.5	286.0	84.1	500.0	118.8	280.7
24	Compound B (30 ng, i.a.)	110.4	450.0	98.5	409.1	80.1	366.1	86.4	500.0	106.7	417.3
41	Compound B (30 ng, i.a.)	87.9	500.0	120.8	421.3	98.1	450.8	81.8	410.9	85.1	420.3
43	Compound B (30 ng, i.a.)	89.1	487.3	111.8	293.2	122.2	462.9	99.3	305.9	91.2	499.9
50	Compound B (30 ng, i.a.)	98.5	455.6	103.1	419.6	99.9	404.8	83.8	429.9	73.7	378.1
77	Compound B (30 ng, i.a.)	110.6	460.4	72.2	432.2	105.1	500.0	104.5	403.0	105.3	451.1
82	Compound B (30 ng, i.a.)	101.5	395.7	87.7	438.6	120.5	377.3	119.8	330.7	99.2	500.0
90	Compound B (30 ng, i.a.)	70.5	462.5	95.1	228.6	103.7	339.9	103.1	345.5	85.8	401.6
25	Compound C (15 ng, i.a.)	86.7	464.3	91.8	413.9	65.9	428.1	107.1	466.5	65.1	500.0
28	Compound C (15 ng, i.a.)	113.3	344.2	107.1	359.6	96.8	439.2	98.1	418.1	85.2	347.1
39	Compound C (15 ng, i.a.)	111.9	343.6	196.4	275.6	109.8	364.0	96.6	414.2	99.8	316.1
49	Compound C (15 ng, i.a.)	76.7	444.4	103.1	461.2	79.9	500.0	93.8	459.0	123.3	328.4
58	Compound C (15 ng, i.a.)	122.3	373.2	105.0	334.2	99.7	317.8	108.1	330.4	79.8	431.4
70	Compound C (15 ng, i.a.)	83.0	427.0	86.6	363.1	105.0	429.0	111.4	304.2	102.6	500.0
87	Compound C (15 ng, i.a.)	104.5	370.8	95.1	500.0	140.4	413.7	103.5	396.0	112.2	431.8
91	Compound C (15 ng, i.a.)	108.0	472.8	98.2	418.3	107.7	370.0	112.5	388.9	81.0	315.8
95	Compound C (15 ng, i.a.)	100.8	424.1	85.3	430.4	129.3	492.4	100.2	453.7	117.2	500.0
98	Compound C (15 ng, i.a.)	98.4	476.7	*82.1*	*464.5*	*97.1*	*376.5*	*121.1*	*442.5*	*93.3*	*275.6*
21	Compound D (0.1 mg, i.a.)	116.8	500.0	73.7	412.3	117.7	381.9	107.3	467.9	108.0	446.1
30	Compound D (0.1 mg, i.a.)	85.5	447.0	110.5	473.0	110.6	363.6	106.8	341.0	104.8	338.8
42	Compound D (0.1 mg, i.a.)	114.3	500.0	118.8	378.3	111.1	315.3	92.2	488.5	109.6	500.0
53	Compound D (0.1 mg, i.a.)	72.6	427.2	99.5	337.0	109.7	386.3	123.7	472.1	106.1	500.0
71	Compound D (0.1 mg, i.a.)	102.4	462.0	95.0	395.9	107.2	349.5	102.5	444.0	87.0	415.9
75	Compound D (0.1 mg, i.a.)	107.5	405.6	159.2	430.5	124.6	447.0	99.0	322.4	114.8	466.2
76	Compound D (0.1 mg, i.a.)	83.6	437.1	124.2	440.0	134.0	471.7	100.0	347.9	85.3	432.2
86	Compound D (0.1 mg, i.a.)	128.6	437.2	89.1	368.2	112.8	387.4	80.5	309.3	119.7	500.0
97	Compound D (0.1 mg, i.a.)	127.6	296.1	107.1	410.1	109.0	385.2	98.4	403.5	95.1	289.8
99	Compound D (0.1 mg, i.a.)	126.8	359.7	98.6	423.1	108.6	356.9	84.7	495.0	83.4	346.6
7	Compound E (500 ng, i.a.)	76.8	500.0	147.8	500.0	107.9	500.0	103.2	393.6	109.3	454.8
11	Compound E (500 ng, i.a.)	126.3	442.7	117.2	497.5	150.4	473.0	89.3	418.3	106.1	460.5
22	Compound E (500 ng, i.a.)	73.5	396.2	133.9	378.6	108.4	382.2	99.9	466.2	95.7	444.8
44	Compound E (500 ng, i.a.)	108.0	454.6	110.8	462.3	97.3	366.8	131.2	382.9	85.2	500.0
46	Compound E (500 ng, i.a.)	116.7	398.4	89.8	313.6	62.9	451.7	104.2	283.1	82.1	335.1
52	Compound E (500 ng, i.a.)	77.7	435.7	97.3	249.1	99.2	393.8	76.6	390.6	87.1	241.9
63	Compound E (500 ng, i.a.)	89.0	462.2	83.3	431.5	87.6	297.4	94.0	317.9	77.8	500.0
68	Compound E (500 ng, i.a.)	93.5	500.0	112.0	386.3	123.7	443.2	95.8	450.7	61.1	437.2
89	Compound E (500 ng, i.a.)	78.1	451.4	120.4	500.0	84.2	324.4	88.5	435.5	96.9	310.0
96	Compound E (500 ng, i.a.)	110.3	420.3	111.2	315.3	136.3	357.5	119.6	409.6	94.1	426.3
14	Compound F (0.15 mg, i.a.)	108.0	273.0	103.3	500.0	128.8	500.0	92.0	479.3	77.4	447.1
29	Compound F (0.15 mg, i.a.)	114.8	429.9	148.9	404.9	103.8	298.6	115.0	397.2	95.5	388.4
45	Compound F (0.15 mg, i.a.)	127.8	363.7	100.7	468.2	117.9	472.1	113.7	500.0	113.3	317.4
48	Compound F (0.15 mg, i.a.)	97.9	439.4	120.8	419.9	98.3	500.0	105.0	265.2	92.0	411.7
51	Compound F (0.15 mg, i.a.)	105.0	500.0	100.6	484.2	95.4	382.5	102.1	478.6	115.3	297.1
54	Compound F (0.15 mg, i.a.)	93.1	422.2	156.3	500.0	115.7	312.5	93.5	484.6	103.7	411.1
59	Compound F (0.15 mg, i.a.)	99.7	391.6	126.6	316.2	77.5	405.7	83.2	355.1	89.8	345.3
73	Compound F (0.15 mg, i.a.)	109.2	400.2	140.4	338.6	124.6	292.8	112.3	448.5	89.8	241.3
80	Compound F (0.15 mg, i.a.)	89.1	445.9	116.4	444.9	113.7	485.9	89.9	421.2	77.7	500.0
94	Compound F (0.15 mg, i.a.)	92.8	471.2	69.2	474.7	92.2	323.9	91.3	349.4	120.5	294.3
1	Clonidine (0.1 mg/kg, s.c.)	86.6	500.0	262.6	500.0	152.6	420.9	116.2	324.1	119.7	231.1
12	Clonidine (0.1 mg/kg, s.c.)	109.7	466.0	237.1	443.1	267.6	441.7	132.2	371.3	108.0	374.7
18	Clonidine (0.1 mg/kg, s.c.)	124.3	320.6	290.6	500.0	221.3	450.1	159.3	493.9	96.9	361.4
38	Clonidine (0.1 mg/kg, s.c.)	116.0	430.6	297.4	500.0	303.7	500.0	162.5	291.4	95.4	449.1
47	Clonidine (0.1 mg/kg, s.c.)	97.8	404.2	252.6	391.6	194.0	328.5	147.0	500.0	98.7	433.5
55	Clonidine (0.1 mg/kg, s.c.)	73.9	378.6	298.2	500.0	172.2	446.8	91.9	447.5	82.8	500.0
61	Clonidine (0.1 mg/kg, s.c.)	111.1	438.7	215.3	418.1	203.7	449.8	117.9	412.6	65.8	364.5
64	Clonidine (0.1 mg/kg, s.c.)	128.1	496.4	189.6	464.3	176.0	478.1	157.3	454.1	125.3	407.3
65	Clonidine (0.1 mg/kg, s.c.)	100.5	500.0	179.4	500.0	193.0	422.4	138.0	388.5	81.8	500.0
81	Clonidine (0.1 mg/kg, s.c.)	96.9	467.5	160.8	500.0	254.3	441.7	114.1	364.1	106.3	462.4

Table 69. Individual joint compression data for MIA Trial 3 Day 21.

Joint Compression (g)											
		Day 21 Joint Compression (g)									
		Baseline		1 hour		3 hour		5 hour		24 hour	
Rat #	Treatment	Ipsi.	Contra.	Ipsi.	Contra.	Ipsi.	Contra.	Ipsi.	Contra.	Ipsi.	Contra.
3	Vehicle (saline, 30 μl, i.a.)	117.2	477.3	85.6	500.0	106.8	345.1	93.0	459.9	94.9	368.1
15	Vehicle (saline, 30 μl, i.a.)	108.5	467.8	110.7	500.0	92.7	394.2	129.9	442.6	101.0	389.0
27	Vehicle (saline, 30 μl, i.a.)	85.9	335.8	121.9	386.9	101.6	245.1	102.3	341.1	112.1	483.1
32	Vehicle (saline, 30 μl, i.a.)	80.0	500.0	107.3	457.9	72.9	356.4	94.7	479.3	122.3	500.0
34	Vehicle (saline, 30 μl, i.a.)	84.5	408.4	74.3	500.0	91.5	336.2	104.8	367.1	107.0	399.9
56	Vehicle (saline, 30 μl, i.a.)	85.0	372.2	99.8	422.5	105.7	459.5	93.9	424.4	69.9	442.6
72	Vehicle (saline, 30 μl, i.a.)	100.5	477.9	75.0	426.5	94.4	453.4	76.6	490.5	117.2	404.1
78	Vehicle (saline, 30 μl, i.a.)	46.9	444.7	94.3	417.4	82.0	258.9	93.8	475.8	109.0	297.9
85	Vehicle (saline, 30 μl, i.a.)	70.2	500.0	95.6	359.7	123.0	390.4	101.4	486.0	126.6	492.2
88	Vehicle (saline, 30 μl, i.a.)	92.5	433.3	107.4	446.9	104.6	281.7	94.7	377.8	111.5	500.0
8	Compound A (4.5 μg, i.a.)	104.6	464.9	93.4	369.1	128.1	321.9	87.2	289.0	105.2	500.0
10	Compound A (4.5 μg, i.a.)	93.5	374.9	78.1	500.0	115.6	418.0	109.0	332.9	113.3	422.1
20	Compound A (4.5 μg, i.a.)	91.3	457.8	108.0	489.3	78.9	500.0	109.5	382.3	103.1	456.6
26	Compound A (4.5 μg, i.a.)	67.7	387.8	152.5	398.4	112.8	311.4	103.7	290.4	98.0	491.7
33	Compound A (4.5 μg, i.a.)	117.2	387.7	80.1	274.8	50.3	309.0	98.4	346.0	38.1	480.7
36	Compound A (4.5 μg, i.a.)	85.0	346.3	50.3	463.8	96.4	211.4	92.8	329.3	111.4	500.0
40	Compound A (4.5 μg, i.a.)	107.2	500.0	101.2	385.1	110.6	499.5	76.5	500.0	97.4	500.0
57	Compound A (4.5 μg, i.a.)	91.1	465.6	91.5	500.0	98.6	362.8	105.0	500.0	98.7	422.0
66	Compound A (4.5 μg, i.a.)	102.5	414.6	137.8	500.0	93.7	401.0	104.4	348.0	84.2	379.0
74	Compound A (4.5 μg, i.a.)	94.8	500.0	114.8	413.2	92.2	397.8	106.5	457.2	109.7	257.7
13	Compound B (30 ng, i.a.)	95.2	470.4	102.1	408.4	102.7	363.7	118.5	287.0	120.0	452.5
17	Compound B (30 ng, i.a.)	88.2	499.4	92.9	356.8	90.3	248.9	108.9	353.1	107.0	429.9
23	Compound B (30 ng, i.a.)	91.2	482.0	78.4	500.0	126.9	500.0	94.0	218.7	55.0	255.6
24	Compound B (30 ng, i.a.)	97.7	449.0	113.1	376.6	105.1	500.0	50.1	385.8	111.2	269.7
41	Compound B (30 ng, i.a.)	93.2	496.7	111.1	338.1	107.0	500.0	96.2	454.3	118.2	500.0
43	Compound B (30 ng, i.a.)	88.3	387.8	128.6	435.6	119.2	410.8	84.4	410.8	110.2	500.0
50	Compound B (30 ng, i.a.)	68.0	329.6	103.6	407.8	72.6	334.2	91.8	345.9	110.9	334.9
77	Compound B (30 ng, i.a.)	59.1	500.0	106.0	226.9	107.2	417.9	98.8	315.6	77.4	455.9
82	Compound B (30 ng, i.a.)	89.7	489.0	114.0	278.8	110.9	311.4	116.7	396.5	108.7	441.0
90	Compound B (30 ng, i.a.)	103.8	418.8	81.7	213.5	72.9	371.4	96.1	500.0	95.6	450.9
25	Compound C (15 ng, i.a.)	77.7	500.0	77.9	500.0	61.4	401.7	110.2	406.0	90.8	500.0
28	Compound C (15 ng, i.a.)	115.3	492.8	59.6	325.5	113.6	359.4	53.4	424.8	93.8	283.0
39	Compound C (15 ng, i.a.)	117.0	438.2	123.5	469.5	108.2	456.8	98.7	380.2	94.2	420.2
49	Compound C (15 ng, i.a.)	90.5	496.2	100.3	465.1	102.9	500.0	93.8	408.1	103.6	386.9
58	Compound C (15 ng, i.a.)	86.2	210.0	105.8	490.4	97.3	214.3	69.2	366.1	57.0	376.9
70	Compound C (15 ng, i.a.)	79.5	426.4	114.0	453.2	73.1	438.1	83.5	442.6	91.9	388.4
87	Compound C (15 ng, i.a.)	86.0	437.8	105.2	441.8	146.3	214.9	120.1	439.3	98.2	395.0
91	Compound C (15 ng, i.a.)	68.8	469.0	117.1	369.5	53.6	386.1	103.4	440.7	126.9	500.0
95	Compound C (15 ng, i.a.)	120.2	475.9	80.0	442.2	94.2	288.7	110.2	486.3	136.9	471.2
98	Compound C (15 ng, i.a.)	*57.7*	*433.0*	*120.5*	*361.0*	*55.4*	*425.1*	*96.7*	*357.2*	*110.3*	*400.8*
21	Compound D (0.1 mg, i.a.)	51.9	500.0	82.2	248.3	77.1	410.5	96.4	385.1	77.7	409.7
30	Compound D (0.1 mg, i.a.)	96.2	377.0	115.1	441.8	97.4	375.1	82.6	359.7	74.9	369.3
42	Compound D (0.1 mg, i.a.)	102.2	330.4	119.4	416.5	120.3	394.5	100.0	373.5	102.9	339.6
53	Compound D (0.1 mg, i.a.)	87.5	379.3	117.8	268.1	55.4	320.7	107.2	452.0	100.8	386.8
71	Compound D (0.1 mg, i.a.)	80.1	388.6	89.4	495.1	72.9	235.2	94.7	401.4	121.2	500.0
75	Compound D (0.1 mg, i.a.)	133.7	408.2	169.2	315.3	126.9	500.0	116.9	399.9	77.7	447.8
76	Compound D (0.1 mg, i.a.)	87.4	500.0	106.4	428.3	102.4	487.2	103.5	500.0	97.1	500.0
86	Compound D (0.1 mg, i.a.)	77.9	500.0	176.2	454.4	108.7	457.1	107.4	374.3	92.7	268.3
97	Compound D (0.1 mg, i.a.)	109.6	432.2	77.9	318.4	102.9	220.6	129.6	500.0	119.5	495.0
99	Compound D (0.1 mg, i.a.)	100.4	494.8	58.3	500.0	45.8	427.4	109.0	308.4	83.2	430.9
7	Compound E (500 ng, i.a.)	121.3	500.0	170.2	371.8	98.9	455.3	87.9	476.3	126.1	408.1
11	Compound E (500 ng, i.a.)	125.4	436.7	155.8	378.6	66.0	475.0	49.5	338.7	60.5	354.0
22	Compound E (500 ng, i.a.)	85.6	391.9	135.5	500.0	126.6	500.0	107.0	412.7	113.5	500.0
44	Compound E (500 ng, i.a.)	100.5	427.0	100.9	487.9	93.1	500.0	87.9	439.8	98.1	469.5
46	Compound E (500 ng, i.a.)	116.5	412.5	151.1	300.1	122.0	500.0	103.1	365.0	72.5	500.0
52	Compound E (500 ng, i.a.)	92.8	440.5	106.6	500.0	109.2	500.0	92.9	329.4	64.9	498.0
63	Compound E (500 ng, i.a.)	111.1	214.8	106.3	268.3	104.5	352.2	57.8	322.3	76.1	279.6
68	Compound E (500 ng, i.a.)	70.0	500.0	97.5	422.2	85.6	500.0	109.0	375.9	89.1	391.4
89	Compound E (500 ng, i.a.)	74.2	387.0	131.4	422.6	102.9	474.0	123.4	425.2	126.1	362.1
96	Compound E (500 ng, i.a.)	84.3	479.7	104.7	500.0	120.9	500.0	108.5	466.2	115.5	335.0
14	Compound F (0.15 mg, i.a.)	93.3	452.2	136.4	326.2	114.1	436.6	48.6	350.9	120.7	500.0
29	Compound F (0.15 mg, i.a.)	89.5	401.9	42.0	421.1	114.1	305.4	75.5	264.2	85.2	456.3
45	Compound F (0.15 mg, i.a.)	91.0	333.0	91.5	420.6	64.4	304.2	81.6	379.2	124.5	361.6
48	Compound F (0.15 mg, i.a.)	57.8	481.8	51.5	420.0	109.0	295.6	134.6	303.2	94.7	414.9
51	Compound F (0.15 mg, i.a.)	94.1	451.0	77.4	435.0	101.9	415.6	107.3	445.7	80.7	232.7
54	Compound F (0.15 mg, i.a.)	51.3	455.3	91.3	220.1	94.0	381.3	120.4	364.6	90.3	415.4
59	Compound F (0.15 mg, i.a.)	91.1	398.6	128.6	460.8	94.0	368.3	88.2	300.1	107.4	389.1
73	Compound F (0.15 mg, i.a.)	93.0	348.2	166.3	374.0	76.5	465.8	116.2	317.5	105.8	499.3
80	Compound F (0.15 mg, i.a.)	109.8	491.3	154.5	419.9	100.4	449.0	117.9	390.9	85.8	500.0
94	Compound F (0.15 mg, i.a.)	98.6	319.2	68.2	268.3	113.0	420.7	118.7	391.3	73.3	454.3
1	Clonidine (0.1 mg/kg, s.c.)	136.3	326.3	228.8	500.0	185.5	333.0	114.6	320.7	75.7	481.9
12	Clonidine (0.1 mg/kg, s.c.)	129.3	500.0	238.8	330.8	198.1	417.9	86.2	353.1	103.1	475.9
18	Clonidine (0.1 mg/kg, s.c.)	94.9	457.8	356.8	477.9	166.2	471.6	102.5	367.9	104.6	470.2
38	Clonidine (0.1 mg/kg, s.c.)	96.8	389.5	261.1	500.0	165.4	466.5	107.3	448.2	85.3	324.3
47	Clonidine (0.1 mg/kg, s.c.)	72.1	398.9	203.4	500.0	137.1	313.6	89.3	364.4	104.4	421.8
55	Clonidine (0.1 mg/kg, s.c.)	76.0	405.0	262.8	500.0	196.5	447.3	81.8	442.7	91.0	418.5
61	Clonidine (0.1 mg/kg, s.c.)	77.3	472.1	251.4	500.0	133.9	421.4	58.4	438.7	77.9	345.6
64	Clonidine (0.1 mg/kg, s.c.)	112.1	417.1	209.1	500.0	146.5	477.9	74.2	292.6	102.0	493.8
65	Clonidine (0.1 mg/kg, s.c.)	108.3	397.7	212.1	500.0	207.8	427.0	91.7	440.2	76.3	305.8
81	Clonidine (0.1 mg/kg, s.c.)	105.5	433.6	182.4	332.4	173.2	417.6	57.6	440.2	104.1	383.2

Table 70. Individual joint compression data for MIA Trial 3 Day 28.

Joint Compression (g)											
		Day 28 Joint Compression (g)									
		Baseline		1 hour		3 hour		5 hour		24 hour	
Rat #	Treatment	Ipsi.	Contra.	Ipsi.	Contra.	Ipsi.	Contra.	Ipsi.	Contra.	Ipsi.	Contra.
3	Vehicle (saline, 30 µl, i.a.)	71.4	321.8	102.9	500.0	92.2	357.0	91.2	449.4	104.2	500.0
15	Vehicle (saline, 30 µl, i.a.)	103.1	408.3	72.8	298.0	82.8	422.5	67.7	328.9	72.9	500.0
27	Vehicle (saline, 30 µl, i.a.)	96.6	484.5	82.3	385.6	88.7	435.0	40.9	365.2	66.2	426.6
32	Vehicle (saline, 30 µl, i.a.)	75.1	456.6	51.9	480.5	82.3	500.0	85.9	382.7	57.4	500.0
34	Vehicle (saline, 30 µl, i.a.)	95.4	381.0	49.1	500.0	116.9	476.9	87.4	310.5	101.7	373.0
56	Vehicle (saline, 30 µl, i.a.)	66.3	398.9	87.1	298.0	60.4	478.3	79.6	366.0	107.9	387.7
72	Vehicle (saline, 30 µl, i.a.)	101.0	388.0	128.3	496.3	100.2	255.5	40.7	415.7	129.9	487.9
78	Vehicle (saline, 30 µl, i.a.)	72.6	500.0	107.0	500.0	92.6	500.0	54.8	474.9	119.5	500.0
85	Vehicle (saline, 30 µl, i.a.)	111.3	370.1	116.3	352.4	119.1	500.0	124.8	318.3	111.4	368.3
88	Vehicle (saline, 30 µl, i.a.)	83.5	408.3	94.9	500.0	69.1	417.5	68.5	500.0	85.2	375.0
8	Compound A (4.5 µg, i.a.)	115.6	431.5	103.9	402.5	67.7	500.0	58.3	435.9	59.5	486.5
10	Compound A (4.5 µg, i.a.)	101.8	492.0	104.9	383.0	110.6	274.6	99.9	341.6	116.5	424.2
20	Compound A (4.5 µg, i.a.)	115.7	360.6	67.0	434.6	81.3	455.5	100.8	300.7	78.1	440.6
26	Compound A (4.5 µg, i.a.)	71.2	476.2	82.2	402.2	88.3	490.8	57.9	500.0	97.3	413.2
33	Compound A (4.5 µg, i.a.)	116.0	492.5	98.6	394.4	89.5	479.3	90.5	335.4	80.1	500.0
36	Compound A (4.5 µg, i.a.)	53.1	457.6	80.9	463.7	47.7	500.0	70.9	450.5	102.9	326.7
40	Compound A (4.5 µg, i.a.)	92.3	481.7	85.4	500.0	114.4	500.0	97.8	459.2	80.7	500.0
57	Compound A (4.5 µg, i.a.)	87.3	401.2	67.8	443.7	106.5	472.8	96.2	500.0	122.3	500.0
66	Compound A (4.5 µg, i.a.)	84.5	478.6	45.2	500.0	79.4	384.1	70.7	357.6	95.4	500.0
74	Compound A (4.5 µg, i.a.)	65.3	457.1	109.0	450.9	74.8	500.0	51.8	471.8	79.9	500.0
13	Compound B (30 ng, i.a.)	70.2	500.0	61.5	500.0	102.2	442.1	100.3	339.8	65.0	354.5
17	Compound B (30 ng, i.a.)	111.1	476.9	87.3	500.0	97.9	500.0	97.0	416.6	45.7	307.4
23	Compound B (30 ng, i.a.)	53.5	388.5	91.5	455.4	76.0	233.1	46.3	214.6	90.4	279.0
24	Compound B (30 ng, i.a.)	73.8	500.0	70.5	306.4	102.5	389.2	64.6	439.0	52.8	500.0
41	Compound B (30 ng, i.a.)	114.8	334.7	90.5	497.5	100.3	408.5	76.4	500.0	100.7	500.0
43	Compound B (30 ng, i.a.)	104.8	268.5	103.5	327.4	50.8	452.7	127.1	293.0	105.6	500.0
50	Compound B (30 ng, i.a.)	63.8	500.0	53.2	319.2	90.3	319.5	111.5	486.0	57.4	416.5
77	Compound B (30 ng, i.a.)	67.5	412.2	82.7	388.7	88.5	364.9	96.2	345.8	112.5	348.3
82	Compound B (30 ng, i.a.)	120.6	495.6	65.9	500.0	102.2	498.3	109.6	500.0	123.9	498.7
90	Compound B (30 ng, i.a.)	91.0	378.5	92.0	301.9	93.4	360.6	80.4	388.5	76.7	500.0
25	Compound C (15 ng, i.a.)	93.2	358.1	51.5	489.7	94.3	303.8	115.6	434.1	49.1	433.8
28	Compound C (15 ng, i.a.)	80.0	252.3	66.6	353.1	104.0	392.6	98.3	312.3	63.5	484.2
39	Compound C (15 ng, i.a.)	99.0	428.0	116.2	500.0	96.6	500.0	72.2	436.8	58.4	366.4
49	Compound C (15 ng, i.a.)	100.2	445.3	105.4	335.8	121.8	300.9	122.5	500.0	63.7	500.0
58	Compound C (15 ng, i.a.)	76.4	373.8	68.1	244.0	83.4	335.3	101.2	373.7	57.6	412.9
70	Compound C (15 ng, i.a.)	93.2	488.4	71.2	500.0	94.6	500.0	105.3	500.0	48.6	500.0
87	Compound C (15 ng, i.a.)	90.5	402.1	122.0	414.7	107.3	462.9	92.3	487.8	79.2	324.3
91	Compound C (15 ng, i.a.)	93.9	366.9	86.0	500.0	66.6	419.2	83.9	500.0	95.6	386.3
95	Compound C (15 ng, i.a.)	77.1	500.0	83.6	500.0	72.0	500.0	54.4	322.6	65.4	500.0
98	Compound C (15 ng, i.a.)	*66.6*	*486.3*	*81.9*	*416.8*	*85.4*	*420.7*	*92.2*	*399.1*	*93.7*	*362.4*
21	Compound D (0.1 mg, i.a.)	77.2	442.0	87.1	457.7	96.4	500.0	99.0	500.0	74.7	500.0
30	Compound D (0.1 mg, i.a.)	117.6	377.1	47.2	395.2	103.6	360.6	62.0	368.7	50.6	331.8
42	Compound D (0.1 mg, i.a.)	118.6	342.9	51.3	386.1	84.0	396.7	97.1	474.6	66.7	286.6
53	Compound D (0.1 mg, i.a.)	76.4	385.8	83.6	414.0	87.6	325.0	107.0	266.9	80.6	270.5
71	Compound D (0.1 mg, i.a.)	58.0	424.2	88.0	344.2	134.8	500.0	57.6	446.9	49.1	399.6
75	Compound D (0.1 mg, i.a.)	101.0	475.0	99.4	500.0	113.4	500.0	135.2	500.0	81.0	442.0
76	Compound D (0.1 mg, i.a.)	81.3	405.8	108.0	329.9	86.2	483.9	82.2	348.5	58.5	364.0
86	Compound D (0.1 mg, i.a.)	93.2	401.8	117.8	329.8	98.5	327.4	88.6	423.1	116.5	337.6
97	Compound D (0.1 mg, i.a.)	129.9	452.6	113.3	313.2	100.7	432.5	83.8	500.0	89.2	282.2
99	Compound D (0.1 mg, i.a.)	78.5	500.0	79.2	500.0	79.7	368.1	85.4	460.4	89.6	500.0
7	Compound E (500 ng, i.a.)	109.4	500.0	166.6	444.9	116.2	500.0	91.3	496.0	103.9	426.7
11	Compound E (500 ng, i.a.)	94.9	364.3	100.2	302.0	76.5	361.9	112.1	408.1	71.9	467.7
22	Compound E (500 ng, i.a.)	104.5	494.9	78.9	500.0	91.3	337.3	124.8	438.4	78.9	306.0
44	Compound E (500 ng, i.a.)	114.1	382.4	70.7	325.5	113.2	411.7	85.8	332.4	76.1	307.3
46	Compound E (500 ng, i.a.)	100.3	488.5	89.1	500.0	110.9	422.5	62.7	306.6	95.9	422.9
52	Compound E (500 ng, i.a.)	69.7	455.8	62.0	407.6	123.8	500.0	83.1	237.7	94.5	458.6
63	Compound E (500 ng, i.a.)	105.6	249.7	80.9	363.8	72.3	420.2	73.3	258.0	59.7	500.0
68	Compound E (500 ng, i.a.)	71.2	442.9	92.3	351.8	114.7	477.8	87.5	388.9	81.3	394.6
89	Compound E (500 ng, i.a.)	111.8	489.3	67.7	249.6	103.4	500.0	128.2	500.0	83.4	500.0
96	Compound E (500 ng, i.a.)	110.9	500.0	113.2	363.0	102.7	404.0	62.4	500.0	92.2	443.0
14	Compound F (0.15 mg, i.a.)	121.5	315.6	151.9	424.8	66.3	447.6	92.1	408.6	74.1	368.8
29	Compound F (0.15 mg, i.a.)	95.4	310.3	52.8	420.7	91.8	329.4	74.8	261.0	28.5	453.4
45	Compound F (0.15 mg, i.a.)	102.2	363.2	52.3	387.1	100.7	251.6	123.8	361.9	98.5	480.0
48	Compound F (0.15 mg, i.a.)	105.4	332.4	95.9	469.9	95.4	433.0	52.8	333.3	22.7	353.6
51	Compound F (0.15 mg, i.a.)	66.9	438.1	79.3	305.0	87.6	322.4	129.3	405.9	73.8	420.4
54	Compound F (0.15 mg, i.a.)	94.3	382.8	121.7	336.0	91.5	500.0	88.8	353.1	78.2	376.9
59	Compound F (0.15 mg, i.a.)	100.4	374.0	85.6	413.6	104.7	412.7	113.9	500.0	27.8	433.2
73	Compound F (0.15 mg, i.a.)	99.9	340.8	119.7	350.3	80.1	293.6	97.7	240.5	89.0	317.8
80	Compound F (0.15 mg, i.a.)	98.3	440.1	100.7	344.4	127.2	232.0	76.2	389.0	107.5	500.0
94	Compound F (0.15 mg, i.a.)	125.4	328.4	118.2	281.9	114.5	375.6	136.6	267.6	54.1	457.4
1	Clonidine (0.1 mg/kg, s.c.)	74.2	500.0	227.9	332.5	126.9	319.5	107.0	484.7	104.3	372.0
12	Clonidine (0.1 mg/kg, s.c.)	83.5	405.7	199.1	359.0	217.2	500.0	132.5	359.5	121.3	434.1
18	Clonidine (0.1 mg/kg, s.c.)	110.9	292.8	*92.1*	*371.6*	*89.9*	*327.4*	*132.0*	*500.0*	*95.9*	*282.8*
38	Clonidine (0.1 mg/kg, s.c.)	96.8	385.5	21.8	500.0	158.1	500.0	136.1	317.3	81.2	500.0
47	Clonidine (0.1 mg/kg, s.c.)	80.3	396.7	199.9	500.0	191.5	402.6	77.9	382.2	69.5	432.2
55	Clonidine (0.1 mg/kg, s.c.)	57.3	500.0	157.1	433.2	151.7	500.0	95.4	469.6	95.0	449.7
61	Clonidine (0.1 mg/kg, s.c.)	62.7	500.0	195.9	500.0	84.2	332.0	111.6	365.5	83.4	407.4
64	Clonidine (0.1 mg/kg, s.c.)	69.7	424.6	193.3	500.0	172.9	500.0	70.2	500.0	92.0	500.0
65	Clonidine (0.1 mg/kg, s.c.)	74.2	428.0	258.6	491.1	181.0	342.5	107.8	500.0	88.8	500.0
81	Clonidine (0.1 mg/kg, s.c.)	98.5	367.4	158.6	500.0	91.9	325.0	76.5	406.2	79.4	500.0

Weight Bearing Data for MMT/MCLT Trial 1

Table 71. Individual animal data for MMT/MCLT Trial 1 Baseline and Day 7 data

		Day -1 (Baseline)				Day 7 (Pre tx)				Day 7 (1 hr)				Day 7 (3 hr)				Day 7 (5 hr)				Day 7 (24 hr)			
Group 1<br>Vehicle (Saline)<br>ip, QD	Trial	Force (g)		Paw Wt Distribution (g)		Force (g)		Paw Wt Distribution (g)		Force (g)		Paw Wt Distribution (g)		Force (g)		Paw Wt Distribution (g)		Force (g)		Paw Wt Distribution (g)		Force (g)		Paw Wt Distribution (g)	
	#	Left	Right	Per Trial	Mean	Left	Right	Per Trial	Mean	Left	Right	Per Trial	Mean	Left	Right	Per Trial	Mean	Left	Right	Per Trial	Mean	Left	Right	Per Trial	Mean
Animal 1	1	73.01	92.99	-19.98		100.81	75.59	25.22		95.62	88.45	7.17		95.24	84.49	10.75		85.19	72.10	13.09		90.60	44.42	46.18	
	2	72.57	98.79	-26.22	**-11.90**	96.68	85.48	11.20	**19.61**	96.78	87.92	8.86	**6.56**	94.71	86.50	8.21	**8.22**	84.60	71.55	13.05	**11.71**	83.93	53.76	30.17	**34.96**
	3	88.15	77.66	10.49		96.27	73.85	22.42		85.28	81.62	3.66		94.31	88.61	5.70		81.36	72.36	9.00		81.96	53.42	28.54	
2	1	84.79	101.37	-16.58		108.75	75.71	33.04		85.58	76.91	8.67		87.76	58.55	29.21		94.53	89.58	4.95		81.04	70.97	10.07	
	2	64.84	97.60	-32.76	**-26.41**	105.58	87.56	18.02	**26.24**	105.88	76.79	29.09	**22.84**	79.68	57.71	21.97	**24.02**	99.18	88.63	10.55	**7.39**	81.67	72.22	9.45	**9.44**
	3	70.42	100.32	-29.90		102.35	74.68	27.67		108.60	77.83	30.77		77.84	56.96	20.88		89.59	82.92	6.67		81.32	72.51	8.81	
3	1	92.70	65.14	27.56		100.69	61.47	39.22		102.90	70.18	32.72		103.87	65.87	38.00		97.73	54.58	43.15		115.99	60.12	55.87	
	2	87.73	67.03	20.70	**21.53**	109.17	57.81	51.36	**46.42**	87.07	71.66	15.41	**24.98**	112.96	71.33	41.63	**45.84**	97.40	55.11	42.29	**43.00**	104.87	67.65	37.22	**35.22**
	3	84.39	68.05	16.34		110.08	61.39	48.69		92.50	65.69	26.81		115.86	57.98	57.88		100.04	56.47	43.57		87.67	75.11	12.56	
4	1	52.89	80.15	-27.26		105.91	97.63	8.28		106.39	82.29	24.10		88.14	74.71	13.43		88.09	102.49	-14.40		77.44	56.78	20.66	
	2	53.91	83.14	-29.23	**-30.45**	93.94	88.83	5.11	**4.53**	114.94	84.90	30.04	**27.84**	92.97	74.29	18.68	**16.78**	80.05	99.29	-19.24	**-18.70**	81.61	64.62	16.99	**18.05**
	3	54.88	89.75	-34.87		88.91	88.71	0.20		112.73	83.34	29.39		93.48	75.26	18.22		68.83	91.30	-22.47		84.77	68.27	16.50	
5	1	60.12	86.16	-26.04		80.93	65.08	15.85		94.90	75.74	19.16		85.91	96.56	-10.65		79.25	59.89	19.36		84.28	73.90	10.38	
	2	60.48	84.30	-23.82	**-29.45**	92.53	69.28	23.25	**17.29**	96.18	78.38	17.80	**17.71**	78.81	91.07	-12.26	**-13.21**	78.90	59.61	19.29	**20.45**	84.12	80.85	3.27	**5.16**
	3	47.11	85.59	-38.48		76.73	63.97	12.76		97.26	81.08	16.18		79.08	95.80	-16.72		81.26	58.56	22.70		81.80	79.96	1.84	
6	1	71.29	83.33	-12.04		104.08	89.22	14.86		93.89	88.20	5.69		116.60	79.30	37.30		69.85	68.26	1.59		106.73	75.11	31.62	
	2	74.80	77.50	-2.70	**-5.07**	97.60	89.63	7.97	**10.45**	86.46	79.09	7.37	**7.72**	107.65	85.52	22.13	**31.59**	91.89	50.46	41.43	**28.32**	109.68	74.25	35.43	**34.34**
	3	77.99	78.45	-0.46		96.23	87.70	8.53		85.15	75.05	10.10		108.42	73.08	35.34		89.56	47.62	41.94		111.04	75.07	35.97	
7	1	119.09	77.28	41.81		83.83	72.72	11.11		87.10	61.54	25.56		100.13	66.82	33.31		84.11	65.60	18.51		90.67	74.67	16.00	
	2	106.28	97.86	8.42	**21.75**	87.73	74.72	13.01	**11.45**	88.94	64.81	24.13	**28.27**	96.80	67.80	29.00	**30.21**	80.38	65.31	15.07	**16.49**	100.96	93.95	7.01	**12.06**
	3	108.04	93.02	15.02		84.13	73.90	10.23		98.27	63.14	35.13		95.84	67.53	28.31		80.96	65.08	15.88		98.56	85.39	13.17	
8	1	101.71	78.85	22.86		83.60	71.95	11.65		93.02	76.62	16.40		74.90	86.35	-11.45		83.79	69.69	14.10		91.61	71.09	20.52	
	2	93.05	82.81	10.24	**15.15**	79.10	74.07	5.03	**6.52**	91.69	80.76	10.93	**12.31**	73.38	84.76	-11.38	**-11.67**	85.32	69.70	15.62	**15.01**	90.57	70.64	19.93	**22.35**
	3	98.11	85.77	12.34		77.40	74.52	2.88		90.96	81.35	9.61		72.86	85.05	-12.19		83.61	68.29	15.32		97.93	71.33	26.60	
**Mean**		**79.10**	**84.70**	**-5.61**	**-5.61**	**94.29**	**76.48**	**17.82**	**17.82**	**95.75**	**77.22**	**18.53**	**18.53**	**92.80**	**76.33**	**16.47**	**16.47**	**85.64**	**70.19**	**15.46**	**15.46**	**91.70**	**70.25**	**21.45**	**21.45**
**SE**		**3.97**	**2.11**	**4.79**	**7.99**	**2.18**	**2.18**	**2.81**	**4.80**	**1.79**	**1.58**	**2.01**	**3.11**	**2.74**	**2.49**	**4.19**	**7.41**	**1.69**	**3.07**	**3.74**	**6.26**	**2.30**	**2.20**	**2.83**	**4.33**

Group 2<br>Morphine (10 mg/kg)<br>ip, QD	Trial	Force (g)		Paw Wt Distribution (g)		Force (g)		Paw Wt Distribution (g)		Force (g)		Paw Wt Distribution (g)		Force (g)		Paw Wt Distribution (g)		Force (g)		Paw Wt Distribution (g)		Force (g)		Paw Wt Distribution (g)	
	#	Left	Right	Per Trial	Mean	Left	Right	Per Trial	Mean	Left	Right	Per Trial	Mean	Left	Right	Per Trial	Mean	Left	Right	Per Trial	Mean	Left	Right	Per Trial	Mean
Animal 1	1	65.14	77.62	-12.48		94.85	46.39	48.46		77.14	68.48	8.66		102.36	90.08	12.28		83.88	81.29	2.59		84.54	73.27	11.27	
	2	49.05	54.04	-4.99	**-3.07**	80.37	74.70	5.67	**22.82**	80.10	64.70	15.40	**12.53**	100.37	79.85	20.52	**24.65**	84.89	68.73	16.16	**10.13**	85.35	68.25	17.10	**12.07**
	3	65.75	57.49	8.26		79.05	64.71	14.34		88.26	74.72	13.54		103.50	62.35	41.15		89.10	77.46	11.64		87.63	79.79	7.84	
2	1	76.12	80.23	-4.11		79.43	63.17	16.26		63.95	68.97	-5.02		71.06	58.80	12.26		82.31	78.24	4.07		73.43	62.39	11.04	
	2	72.98	79.14	-6.16	**-16.73**	93.33	56.37	36.96	**21.82**	70.71	78.64	-7.93	**-4.50**	67.71	55.78	11.93	**12.59**	79.49	75.43	4.06	**5.71**	74.52	55.82	18.70	**17.42**
	3	61.12	101.05	-39.93		81.00	68.77	12.23		74.29	74.83	-0.54		67.86	54.29	13.57		75.91	66.91	9.00		77.40	54.87	22.53	
3	1	94.37	63.32	31.05		92.23	65.13	27.10		74.18	91.89	-17.71		89.99	91.93	-1.94		84.45	66.39	18.06		110.83	92.64	18.19	
	2	88.03	71.09	16.94	**28.01**	90.57	63.29	27.28	**24.56**	75.42	92.92	-17.50	**-15.15**	90.46	94.60	-4.14	**-2.52**	87.22	68.77	18.45	**16.73**	109.31	93.35	15.96	**17.27**
	3	96.73	60.70	36.03		94.93	75.62	19.31		79.91	90.15	-10.24		83.99	85.48	-1.49		94.11	80.44	13.67		108.42	90.77	17.65	
4	1	74.59	85.48	-10.89		110.67	65.51	45.16		83.99	73.19	10.80		76.32	52.04	24.28		94.13	60.32	33.81		98.42	76.82	21.60	
	2	83.91	78.02	5.89	**-4.17**	101.59	57.00	44.59	**33.37**	99.23	55.25	43.98	**26.92**	83.00	57.41	25.59	**25.03**	105.45	62.44	43.01	**39.94**	98.20	77.81	20.39	**14.44**
	3	61.79	69.29	-7.50		85.81	75.46	10.35		89.56	63.58	25.98		74.90	49.69	25.21		103.33	60.33	43.00		93.73	92.40	1.33	
5	1	89.34	69.32	20.02		81.27	71.31	9.96		116.02	105.54	10.48		67.63	55.86	11.77		82.18	49.67	32.51		76.58	75.97	0.61	
	2	65.06	64.99	0.07	**1.69**	88.57	72.08	16.49	**21.17**	116.14	102.73	13.41	**13.77**	93.71	58.86	34.85	**27.42**	84.74	52.89	31.85	**33.34**	77.15	76.69	0.46	**3.71**
	3	61.25	76.27	-15.02		99.18	62.12	37.06		118.08	100.67	17.41		95.17	59.52	35.65		88.72	53.06	35.66		88.14	78.07	10.07	
6	1	78.78	92.96	-14.18		83.80	80.35	3.45		90.04	70.29	19.75		96.76	77.89	18.87		95.53	71.69	23.84		90.85	84.06	6.79	
	2	71.88	99.52	-27.64	**-21.91**	94.89	64.95	29.94	**18.96**	96.49	68.70	27.79	**26.48**	101.93	83.79	18.14	**21.63**	95.79	77.60	18.19	**21.24**	105.76	94.27	11.49	**8.29**
	3	74.78	98.70	-23.92		90.43	66.95	23.48		99.66	67.76	31.90		97.28	69.40	27.88		83.01	61.31	21.70		102.39	95.79	6.60	
7	1	81.47	78.60	2.87		84.16	84.37	-0.21		64.84	83.79	-18.95		73.71	64.66	9.05		95.50	65.92	29.58		96.98	51.74	45.24	
	2	86.93	78.24	8.69	**4.28**	63.27	84.71	-21.44	**-10.76**	65.82	85.05	-19.23	**-15.92**	77.83	65.30	12.53	**12.01**	98.81	68.10	30.71	**29.79**	98.03	51.42	46.61	**45.00**
	3	82.48	81.20	1.28		76.96	87.59	-10.63		72.74	82.33	-9.59		79.65	65.20	14.45		96.01	66.93	29.08		97.51	54.37	43.14	
8	1	63.97	68.12	-4.15		104.00	61.04	42.96		103.37	93.08	10.29		109.54	85.26	24.28		73.58	89.43	-15.85		90.19	73.71	16.48	
	2	60.59	74.89	-14.30	**-8.61**	102.70	63.35	39.35	**39.85**	93.01	87.21	5.80	**9.08**	107.31	79.33	27.98	**27.64**	73.90	88.80	-14.90	**-15.30**	88.95	73.90	15.05	**14.89**
	3	58.91	66.28	-7.37		101.71	64.48	37.23		93.90	82.75	11.15		108.16	77.49	30.67		73.40	88.54	-15.14		88.91	75.77	13.14	
**Mean**		**73.54**	**76.11**	**-2.56**	**-2.56**	**89.78**	**68.31**	**21.47**	**21.47**	**86.95**	**80.30**	**6.65**	**6.65**	**88.34**	**69.79**	**18.56**	**18.56**	**87.73**	**70.03**	**17.70**	**17.70**	**91.80**	**75.16**	**16.64**	**16.64**
**SE**		**2.56**	**2.61**	**3.57**	**5.37**	**2.21**	**1.99**	**3.77**	**5.24**	**3.31**	**2.73**	**3.52**	**5.98**	**2.87**	**2.85**	**2.42**	**3.71**	**1.88**	**2.29**	**3.50**	**6.25**	**2.28**	**2.90**	**2.58**	**4.37**

Group 3<br>Cmpd A1 (0.3 mg/kg)<br>ip, QD	Trial	Force (g)		Paw Wt Distribution (g)		Force (g)		Paw Wt Distribution (g)		Force (g)		Paw Wt Distribution (g)		Force (g)		Paw Wt Distribution (g)		Force (g)		Paw Wt Distribution (g)		Force (g)		Paw Wt Distribution (g)	
	#	Left	Right	Per Trial	Mean	Left	Right	Per Trial	Mean	Left	Right	Per Trial	Mean	Left	Right	Per Trial	Mean	Left	Right	Per Trial	Mean	Left	Right	Per Trial	Mean
Animal 1	1	67.10	78.03	-10.93		78.75	64.53	14.22		79.99	52.54	27.45		67.19	85.04	-17.85		79.65	52.07	27.58		64.47	49.92	14.55	
	2	65.79	80.46	-14.67	**-11.41**	78.47	57.65	20.82	**19.34**	87.60	66.38	21.22	**21.85**	67.04	80.58	-13.54	**-15.03**	84.84	52.28	32.56	**30.84**	63.25	55.54	7.71	**14.74**
	3	70.12	78.75	-8.63		79.78	56.79	22.99		78.93	62.04	16.89		66.03	79.73	-13.70		88.11	55.74	32.37		70.68	48.71	21.97	
2	1	102.70	69.59	33.11		74.41	57.10	17.31		67.27	65.40	1.87		65.02	91.51	-26.49		67.21	72.78	-5.57		74.80	72.36	2.44	
	2	101.00	68.00	33.00	**23.53**	76.90	56.37	20.53	**18.28**	69.74	66.35	3.39	**3.83**	59.81	92.28	-32.47	**-30.84**	70.16	72.35	-2.19	**-5.06**	81.27	66.82	14.45	**7.14**
	3	87.39	82.90	4.49		84.47	67.48	16.99		71.25	65.03	6.22		62.27	95.82	-33.55		65.11	72.53	-7.42		76.73	72.20	4.53	
3	1	68.48	74.28	-5.80		75.26	60.11	15.15		80.88	57.93	22.95		82.06	69.38	12.68		78.69	62.02	16.67		97.95	87.31	10.64	
	2	61.13	78.15	-17.02	**-12.50**	70.91	60.62	10.29	**10.23**	78.34	66.47	11.87	**13.19**	79.67	54.90	24.77	**20.99**	84.36	73.28	11.08	**14.53**	87.08	71.24	15.84	**19.06**
	3	62.48	77.15	-14.67		64.40	59.14	5.26		68.00	63.24	4.76		84.20	58.68	25.52		68.76	52.92	15.84		101.17	70.46	30.71	
4	1	78.38	78.36	0.02		72.30	64.73	7.57		98.27	80.82	17.45		103.93	61.91	42.02		111.49	65.78	45.71		82.16	71.56	10.60	
	2	79.44	73.29	6.15	**-4.89**	76.99	73.07	3.92	**5.01**	104.43	78.98	25.45	**23.14**	80.10	68.27	11.83	**20.71**	103.15	66.61	36.54	**30.51**	84.00	56.42	27.58	**17.17**
	3	67.85	88.70	-20.85		63.67	60.12	3.55		101.01	74.48	26.53		85.36	77.07	8.29		82.50	73.21	9.29		86.41	73.07	13.34	
5	1	76.91	103.52	-26.61		79.06	67.68	11.38		105.66	79.18	26.48		79.07	68.77	10.30		80.44	62.13	18.31		91.07	74.21	16.86	
	2	74.95	97.54	-22.59	**-29.21**	82.17	65.56	16.61	**12.44**	104.72	78.23	26.49	**26.82**	92.45	69.40	23.05	**22.20**	81.23	63.80	17.43	**17.18**	80.59	57.53	23.06	**21.81**
	3	75.01	113.44	-38.43		79.20	69.87	9.33		105.10	77.60	27.50		92.04	58.79	33.25		79.95	64.15	15.80		95.90	70.38	25.52	
6	1	54.99	72.38	-17.39		94.16	83.70	10.46		85.34	77.53	7.81		80.70	55.96	24.74		83.99	67.88	16.11		78.98	67.92	11.06	
	2	56.72	82.06	-25.34	**-24.04**	95.47	83.22	12.25	**12.55**	85.61	80.23	5.38	**8.09**	77.90	56.23	21.67	**22.79**	85.71	69.42	16.29	**15.78**	71.42	69.29	2.13	**4.61**
	3	50.71	80.09	-29.38		95.86	80.92	14.94		86.19	75.11	11.08		80.14	58.17	21.97		83.59	68.65	14.94		71.81	71.18	0.63	
7	1	77.16	59.16	18.00		95.17	68.79	26.38		88.89	56.90	31.99		74.26	88.45	-14.19		62.02	86.12	-24.10		99.76	51.02	48.74	
	2	82.59	55.01	27.58	**22.07**	99.22	66.28	32.94	**30.97**	88.99	52.84	36.15	**34.84**	72.48	83.95	-11.47	**-11.13**	61.54	84.04	-22.50	**-22.72**	101.12	54.84	46.28	**46.73**
	3	81.39	60.77	20.62		97.35	63.77	33.58		92.00	55.63	36.37		69.19	76.93	-7.74		63.19	84.76	-21.57		101.59	56.43	45.16	
8	1	79.62	84.78	-5.16		83.90	71.14	12.76		68.90	70.86	-1.96		74.51	63.37	11.14		60.64	57.42	3.22		71.36	59.35	12.01	
	2	69.35	81.18	-11.83	**-3.99**	86.05	69.91	16.14	**9.93**	63.76	72.93	-9.17	**-7.84**	80.51	59.97	20.54	**16.20**	64.09	49.23	14.86	**6.92**	71.97	54.08	17.89	**14.48**
	3	80.36	75.35	5.01		71.89	71.01	0.88		64.89	77.27	-12.38		74.51	57.59	16.92		50.89	48.21	2.68		72.21	58.68	13.53	
**Mean**		**73.82**	**78.87**	**-5.06**	**-5.06**	**81.49**	**66.65**	**14.84**	**14.84**	**84.41**	**68.92**	**15.49**	**15.49**	**77.10**	**71.36**	**5.74**	**5.74**	**76.72**	**65.72**	**11.00**	**11.00**	**82.41**	**64.19**	**18.22**	**18.22**
**SE**		**2.60**	**2.66**	**4.09**	**6.81**	**2.09**	**1.62**	**1.72**	**2.82**	**2.81**	**1.89**	**2.82**	**4.90**	**2.11**	**2.70**	**4.41**	**7.54**	**2.85**	**2.21**	**3.72**	**6.35**	**2.49**	**2.01**	**2.75**	**4.55**

Group 4 Cmpd A2 (0.6 mg/kg) ip, QD	Trial	Force (g)		Paw Wt Distribution (g)		Force (g)		Paw Wt Distribution (g)		Force (g)		Paw Wt Distribution (g)		Force (g)		Paw Wt Distribution (g)		Force (g)		Paw Wt Distribution (g)		Force (g)		Paw Wt Distribution (g)	
	#	Left	Right	Per Trial	Mean	Left	Right	Per Trial	Mean	Left	Right	Per Trial	Mean	Left	Right	Per Trial	Mean	Left	Right	Per Trial	Mean	Left	Right	Per Trial	Mean
Animal 1	1	97.31	88.95	8.36		94.51	74.38	20.13		84.40	74.50	9.90		98.72	66.32	32.40		92.80	78.95	13.85		70.02	95.02	-25.00	
	2	59.47	87.50	-28.03	-15.78	95.70	73.39	22.31	21.85	88.90	77.50	11.40	10.73	100.82	65.42	35.40	33.41	91.80	78.68	13.12	15.01	77.01	87.18	-10.17	-14.39
	3	69.95	97.63	-27.68		88.38	65.26	23.12		90.00	79.10	10.90		105.70	73.28	32.42		97.98	79.91	18.07		75.83	83.84	-8.01	
2	1	91.81	93.28	-1.47		77.65	63.25	14.40		97.93	67.94	29.99		91.25	80.89	10.36		76.87	78.22	-1.35		88.70	79.10	9.60	
	2	93.28	92.56	0.72	3.15	77.74	61.61	16.13	20.61	101.92	61.81	40.11	34.11	87.60	82.23	5.37	5.33	60.48	86.34	-25.86	-9.55	86.79	76.00	10.79	10.72
	3	84.39	74.18	10.21		89.80	58.49	31.31		96.99	64.76	32.23		85.38	85.12	0.26		76.96	78.40	-1.44		88.91	77.14	11.77	
3	1	81.54	100.79	-19.25		74.32	80.55	-6.23		53.96	90.07	-36.11		48.78	76.77	-27.99		61.54	77.90	-16.36		79.83	77.26	2.57	
	2	75.65	104.66	-29.01	-23.50	65.88	82.14	-16.26	-13.84	53.07	87.01	-33.94	-29.86	57.14	75.34	-18.20	-20.74	66.99	72.40	-5.41	-9.37	87.43	73.43	14.00	6.56
	3	76.28	98.52	-22.24		71.07	90.09	-19.02		59.31	78.83	-19.52		61.46	77.49	-16.03		69.01	75.34	-6.33		86.74	83.62	3.12	
4	1	88.73	83.59	5.14		91.81	58.63	33.18		75.32	70.47	4.85		103.32	69.89	33.43		99.21	72.04	27.17		94.85	40.33	54.52	
	2	80.55	77.56	2.99	6.77	86.28	57.19	29.09	28.95	78.68	53.15	25.53	20.27	94.48	82.82	11.66	24.95	97.39	70.89	26.50	25.50	77.53	49.21	28.32	37.42
	3	93.07	80.89	12.18		78.10	53.53	24.57		84.76	54.34	30.42		102.31	72.54	29.77		100.31	77.49	22.82		79.28	49.86	29.42	
5	1	91.21	71.97	19.24		102.04	73.26	28.78		78.46	56.36	22.10		95.33	75.82	19.51		82.02	65.17	16.85		75.76	43.10	32.66	
	2	77.91	61.87	16.04	32.46	100.30	63.18	37.12	22.58	72.74	69.45	3.29	8.96	90.61	79.17	11.44	15.21	69.18	70.63	-1.45	9.07	79.09	42.17	36.92	37.04
	3	108.78	46.69	62.09		91.78	89.94	1.84		72.20	70.71	1.49		93.67	78.99	14.68		80.93	69.11	11.82		82.05	40.51	41.54	
6	1	48.87	80.37	-31.50		77.30	70.90	6.40		82.54	69.35	13.19		96.04	46.45	49.59		89.27	58.74	30.53		85.74	75.04	10.70	
	2	52.06	77.88	-25.82	-29.68	80.11	62.12	17.99	12.93	82.68	66.77	15.91	16.50	89.45	57.00	32.45	38.87	90.53	59.11	31.42	32.92	75.63	73.97	1.66	5.27
	3	52.07	83.80	-31.73		79.44	65.04	14.40		84.73	64.32	20.41		93.23	58.65	34.58		93.65	56.84	36.81		78.09	74.65	3.44	
7	1	73.91	97.95	-24.04		82.02	63.06	18.96		81.61	82.95	-1.34		85.93	63.76	22.17		76.23	64.04	12.19		80.22	58.97	21.25	
	2	77.32	89.23	-11.91	-22.46	68.54	63.08	5.46	10.60	59.61	84.56	-24.95	-17.89	83.78	66.18	17.60	18.46	78.60	65.23	13.37	13.83	75.82	59.86	15.96	21.80
	3	58.91	90.33	-31.42		69.39	62.00	7.39		59.49	86.87	-27.38		86.20	70.60	15.60		81.18	65.26	15.92		89.56	61.36	28.20	
8	1	96.77	51.52	45.25		48.64	30.64	18.00		71.09	56.79	14.30		83.82	61.09	22.73		80.72	48.19	32.53		71.12	56.78	14.34	
	2	95.92	50.37	45.55	39.52	52.57	38.77	13.80	15.87	73.50	56.19	17.31	16.67	86.57	61.99	24.58	24.36	77.40	50.44	26.96	27.76	73.91	56.91	17.00	14.57
	3	80.76	53.01	27.75		55.87	40.06	15.81		74.12	55.73	18.39		90.04	64.28	25.76		75.89	52.11	23.78		72.46	60.09	12.37	
**Mean**		**79.44**	**80.63**	**-1.19**	**-1.19**	**79.14**	**64.19**	**14.95**	**14.95**	**77.42**	**69.98**	**7.44**	**7.44**	**87.98**	**70.50**	**17.48**	**17.48**	**81.96**	**68.81**	**13.15**	**13.15**	**80.52**	**65.64**	**14.87**	**14.87**
**SE**		**3.28**	**3.47**	**5.60**	**9.30**	**2.92**	**2.96**	**2.93**	**4.60**	**2.74**	**2.34**	**4.37**	**7.43**	**2.86**	**1.96**	**3.79**	**6.59**	**2.41**	**2.12**	**3.34**	**5.67**	**1.35**	**3.29**	**3.59**	**6.10**

Group 5 Cmpd B1 (5 µg/kg) ip, QD	Trial	Force (g)		Paw Wt Distribution (g)		Force (g)		Paw Wt Distribution (g)		Force (g)		Paw Wt Distribution (g)		Force (g)		Paw Wt Distribution (g)		Force (g)		Paw Wt Distribution (g)		Force (g)		Paw Wt Distribution (g)	
	#	Left	Right	Per Trial	Mean	Left	Right	Per Trial	Mean	Left	Right	Per Trial	Mean	Left	Right	Per Trial	Mean	Left	Right	Per Trial	Mean	Left	Right	Per Trial	Mean
Animal 1	1	69.19	78.17	-8.98		74.28	69.73	4.55		90.06	66.98	23.08		74.87	67.04	7.83		66.04	78.42	-12.38		74.21	94.16	-19.95	
	2	61.46	82.06	-20.60	-20.21	66.95	55.38	11.57	12.25	86.05	64.45	21.60	20.38	55.94	52.86	3.08	0.38	65.70	70.15	-4.45	-7.50	74.10	95.50	-21.40	-19.03
	3	58.56	89.62	-31.06		72.75	52.12	20.63		82.74	66.29	16.45		54.06	63.84	-9.78		66.97	72.65	-5.68		79.38	95.11	-15.73	
2	1	95.59	67.54	28.05		89.53	65.45	24.08		77.98	66.11	11.87		86.49	74.30	12.19		87.61	74.05	13.56		91.65	54.38	37.27	
	2	78.15	62.74	15.41	4.48	83.29	69.58	13.71	17.67	81.59	75.05	6.54	12.21	71.82	62.95	8.87	15.96	80.21	78.37	1.84	5.91	89.72	55.13	34.59	35.18
	3	58.63	88.65	-30.02		84.31	69.09	15.22		75.61	57.39	18.22		95.70	68.89	26.81		77.04	74.71	2.33		75.00	41.32	33.68	
3	1	94.76	89.74	5.02		59.68	84.40	-24.72		65.11	49.99	15.12		60.07	84.51	-24.44		59.41	75.31	-15.90		106.91	66.41	40.50	
	2	91.31	90.73	0.58	5.37	62.60	79.50	-16.90	-22.34	65.65	53.94	11.71	12.85	61.85	88.68	-26.83	-24.92	55.82	72.48	-16.66	-17.75	101.39	71.23	30.16	37.90
	3	97.57	87.07	10.50		58.56	83.97	-25.41		67.06	55.35	11.71		59.90	83.40	-23.50		54.37	75.06	-20.69		110.22	67.17	43.05	
4	1	70.89	85.45	-14.56		89.75	79.94	9.81		90.25	65.96	24.29		85.67	62.32	23.35		82.52	84.73	-2.21		76.17	86.82	-10.65	
	2	71.13	84.68	-13.55	-13.94	84.22	80.57	3.65	6.23	90.56	63.32	27.24	19.12	90.15	62.13	28.02	25.08	73.29	84.63	-11.34	-11.70	73.01	82.24	-9.23	-10.48
	3	70.05	83.77	-13.72		92.56	87.33	5.23		77.16	71.34	5.82		86.41	62.55	23.86		61.93	83.48	-21.55		68.51	80.06	-11.55	
5	1	89.73	70.41	19.32		68.66	41.54	27.12		95.22	77.10	18.12		100.81	90.43	10.38		91.71	68.97	22.74		78.51	65.57	12.94	
	2	77.16	79.47	-2.31	0.66	71.45	55.24	16.21	28.38	91.65	81.90	9.75	12.16	98.02	88.51	9.51	11.47	93.67	61.88	31.79	30.56	78.54	71.41	7.13	23.51
	3	60.79	75.83	-15.04		76.69	34.88	41.81		87.48	78.86	8.62		95.18	80.65	14.53		93.53	56.37	37.16		101.64	51.17	50.47	
6	1	91.00	88.60	2.40		95.27	87.87	7.40		93.48	66.25	27.23		100.97	87.47	13.50		91.25	86.79	4.46		81.84	74.98	6.86	
	2	97.70	83.09	14.61	4.78	94.83	84.01	10.82	10.05	91.28	59.96	31.32	30.73	103.04	82.70	20.34	18.16	96.66	94.76	1.90	3.52	80.97	75.16	5.81	6.42
	3	82.28	84.96	-2.68		91.00	79.07	11.93		91.83	58.18	33.65		97.83	77.18	20.65		91.07	86.87	4.20		81.56	74.96	6.60	
7	1	80.34	62.65	17.69		60.23	51.05	9.18		67.58	76.16	-8.58		81.79	53.76	28.03		64.56	73.87	-9.31		65.96	58.52	7.44	
	2	75.50	57.69	17.81	20.98	68.93	32.03	36.90	28.47	64.76	72.62	-7.86	-7.77	81.60	53.06	28.54	29.75	61.40	78.56	-17.16	-14.95	74.79	53.59	21.20	26.15
	3	82.84	55.39	27.45		76.32	37.00	39.32		74.30	81.18	-6.88		85.22	52.53	32.69		49.56	67.95	-18.39		82.74	32.92	49.82	
8	1	88.15	48.13	40.02		69.00	53.03	15.97		70.50	38.65	31.85		54.00	42.41	11.59		71.36	75.01	-3.65		67.80	80.94	-13.14	
	2	91.00	55.83	35.17	33.02	68.88	53.42	15.46	13.97	74.23	41.78	32.45	33.22	66.50	48.80	17.70	15.78	72.58	76.35	-3.77	-3.82	69.95	83.97	-14.02	-20.68
	3	88.11	64.25	23.86		65.14	54.66	10.48		80.37	45.01	35.36		65.12	47.06	18.06		72.43	76.48	-4.05		60.57	95.45	-34.88	
**Mean**		**80.08**	**75.69**	**4.39**	**4.39**	**76.04**	**64.20**	**11.83**	**11.83**	**80.52**	**63.91**	**16.61**	**16.61**	**79.71**	**68.25**	**11.46**	**11.46**	**74.20**	**76.16**	**-1.97**	**-1.97**	**81.05**	**71.17**	**9.87**	**9.87**
**SE**		**2.60**	**2.68**	**4.10**	**6.05**	**2.42**	**3.64**	**3.45**	**5.66**	**2.08**	**2.48**	**2.65**	**4.52**	**3.39**	**3.08**	**3.46**	**6.05**	**2.89**	**1.69**	**3.18**	**5.50**	**2.67**	**3.53**	**5.16**	**8.53**

Group 6 Cmpd B2 (10 µg/kg) ip, QD	Trial	Force (g)		Paw Wt Distribution (g)		Force (g)		Paw Wt Distribution (g)		Force (g)		Paw Wt Distribution (g)		Force (g)		Paw Wt Distribution (g)		Force (g)		Paw Wt Distribution (g)		Force (g)		Paw Wt Distribution (g)	
	#	Left	Right	Per Trial	Mean	Left	Right	Per Trial	Mean	Left	Right	Per Trial	Mean	Left	Right	Per Trial	Mean	Left	Right	Per Trial	Mean	Left	Right	Per Trial	Mean
Animal 1	1	58.92	90.38	-31.46		68.50	77.85	-9.35		66.30	77.12	-10.82		87.93	68.73	19.20		91.88	72.23	19.65		68.34	74.38	-6.04	
	2	64.71	90.71	-26.00	-32.47	68.59	83.21	-14.62	-12.63	61.61	76.40	-14.79	-17.87	90.00	79.44	10.56	14.83	94.69	76.39	18.30	15.62	72.26	81.72	-9.46	-8.95
	3	73.28	113.24	-39.96		68.59	82.50	-13.91		64.69	92.68	-27.99		92.22	77.49	14.73		86.30	77.38	8.92		68.47	79.83	-11.36	
2	1	49.01	68.37	-19.36		80.55	70.28	10.27		87.29	94.40	-7.11		98.73	78.42	20.31		94.08	70.07	24.01		71.48	70.40	1.08	
	2	50.29	69.03	-18.74	-23.24	87.13	66.28	20.85	13.15	85.97	96.03	-10.06	-9.18	93.00	75.02	17.98	22.52	88.52	79.02	9.50	16.24	73.56	73.17	0.39	1.96
	3	45.86	77.49	-31.63		76.91	68.59	8.32		83.55	93.91	-10.36		98.48	69.21	29.27		83.93	68.73	15.20		76.32	71.92	4.40	
3	1	79.86	99.90	-20.04		85.69	64.58	21.11		60.04	86.72	-26.68		82.83	70.30	12.53		77.94	57.20	20.74		83.07	77.74	5.33	
	2	80.92	97.30	-16.38	-2.44	79.36	53.49	25.87	22.85	65.82	92.41	-26.59	-24.85	82.65	70.71	11.94	12.36	77.71	59.76	17.95	21.17	85.48	74.70	10.78	8.44
	3	111.96	82.85	29.11		77.87	56.30	21.57		68.64	89.93	-21.29		83.33	70.72	12.61		81.91	57.09	24.82		85.56	76.35	9.21	
4	1	83.54	50.94	32.60		93.77	46.13	47.64		94.51	74.28	20.23		103.95	60.62	43.33		90.74	76.62	14.12		69.16	76.23	-7.07	
	2	93.34	40.77	52.57	40.03	92.78	38.46	54.32	49.78	84.66	77.74	6.92	11.33	105.65	63.21	42.44	42.83	85.05	78.29	6.76	9.68	67.76	76.14	-8.38	-10.97
	3	90.42	55.50	34.92		95.13	47.75	47.38		85.47	78.64	6.83		106.14	63.42	42.72		86.00	77.85	8.15		70.58	88.03	-17.45	
5	1	79.94	86.51	-6.57		56.00	38.01	17.99		63.70	90.72	-27.02		68.21	59.85	8.36		76.65	56.00	20.65		65.47	55.41	10.06	
	2	63.09	95.23	-32.14	-23.03	52.40	35.47	16.93	12.59	54.05	97.80	-43.75	-36.96	72.17	58.62	13.55	12.21	77.74	55.86	21.88	21.10	67.21	56.34	10.87	12.84
	3	61.35	91.74	-30.39		52.17	49.32	2.85		56.84	96.94	-40.10		72.52	57.79	14.73		76.76	56.00	20.76		72.30	54.72	17.58	
6	1	40.90	75.69	-34.79		92.17	83.82	8.35		88.76	72.31	16.45		74.65	96.79	-22.14		77.08	72.15	4.93		63.25	57.21	6.04	
	2	46.37	68.60	-22.23	-30.64	91.95	78.80	13.15	12.15	92.07	62.89	29.18	20.49	74.96	96.79	-21.83	-18.94	81.10	78.20	2.90	4.88	74.39	49.65	24.74	23.28
	3	45.42	80.33	-34.91		94.84	79.89	14.95		63.89	48.05	15.84		78.60	91.45	-12.85		79.52	72.72	6.80		79.97	40.90	39.07	
7	1	98.71	62.31	36.40		61.63	60.23	1.40		65.71	58.44	7.27		56.40	71.28	-14.88		49.10	61.40	-12.30		47.46	70.57	-23.11	
	2	107.85	61.20	46.65	33.08	62.01	58.29	3.72	3.44	68.97	59.58	9.39	9.47	57.50	73.57	-16.07	-14.76	50.65	61.55	-10.90	-11.05	51.63	75.13	-23.50	-19.48
	3	79.96	63.76	16.20		63.87	58.68	5.19		69.91	58.16	11.75		56.71	70.03	-13.32		52.37	62.33	-9.96		59.63	71.46	-11.83	
8	1	75.15	90.32	-15.17		80.70	69.76	10.94		93.36	61.63	31.73		99.92	56.66	43.26		92.54	69.63	22.91		76.51	82.92	-6.41	
	2	79.69	49.08	30.61	17.38	78.23	61.89	16.34	17.10	97.19	60.31	36.88	28.57	97.83	58.00	39.83	40.97	86.42	76.89	9.53	13.61	78.56	82.85	-4.29	-7.68
	3	78.80	42.10	36.70		91.77	67.74	24.03		83.72	66.63	17.09		97.78	57.96	39.82		85.65	77.25	8.40		73.33	85.66	-12.33	
**Mean**		**72.47**	**75.14**	**-2.67**	**-2.67**	**77.19**	**62.39**	**14.80**	**14.80**	**75.28**	**77.66**	**-2.38**	**-2.38**	**84.67**	**70.67**	**14.00**	**14.00**	**80.18**	**68.78**	**11.41**	**11.41**	**70.91**	**70.98**	**-0.07**	**-0.07**
**SE**		**4.11**	**3.97**	**6.40**	**10.36**	**2.88**	**3.01**	**3.56**	**6.25**	**2.76**	**3.13**	**4.70**	**8.23**	**3.17**	**2.40**	**4.43**	**7.96**	**2.60**	**1.77**	**2.22**	**3.74**	**1.89**	**2.49**	**3.03**	**5.04**

Group 7 Cmpd C1 (100 µg/kg) ip, QD	Trial	Force (g)		Paw Wt Distribution (g)		Force (g)		Paw Wt Distribution (g)		Force (g)		Paw Wt Distribution (g)		Force (g)		Paw Wt Distribution (g)		Force (g)		Paw Wt Distribution (g)		Force (g)		Paw Wt Distribution (g)	
	#	Left	Right	Per Trial	Mean	Left	Right	Per Trial	Mean	Left	Right	Per Trial	Mean	Left	Right	Per Trial	Mean	Left	Right	Per Trial	Mean	Left	Right	Per Trial	Mean
Animal 1	1	70.67	80.80	-10.13		90.35	76.66	13.69		81.08	77.14	3.94		96.59	69.16	27.43		96.60	75.13	21.47		88.95	54.42	34.53	
	2	73.33	79.87	-6.54	-8.43	82.50	72.38	10.12	15.61	80.08	77.12	2.96	3.06	96.19	68.35	27.84	27.45	95.43	74.25	21.18	20.81	95.41	55.00	40.41	38.75
	3	79.03	87.66	-8.63		94.75	71.73	23.02		79.23	76.95	2.28		95.00	67.91	27.09		93.17	73.40	19.77		97.72	56.42	41.30	
2	1	75.67	65.49	10.18		85.19	56.13	29.06		73.06	86.03	-12.97		83.34	72.45	10.89		81.33	100.57	-19.24		80.02	72.94	7.08	
	2	82.79	64.68	18.11	10.89	89.52	54.61	34.91	23.22	70.72	84.16	-13.44	-13.07	84.19	72.98	11.21	10.42	81.81	101.58	-19.77	-20.98	80.79	74.48	6.31	5.58
	3	75.03	70.65	4.38		71.12	65.44	5.68		69.91	82.71	-12.80		83.52	74.36	9.16		76.52	100.45	-23.93		80.92	77.58	3.34	
3	1	90.63	83.41	7.22		90.95	79.57	11.38		67.11	77.00	-9.89		98.25	83.90	14.35		60.00	90.54	-30.54		69.39	73.32	-3.93	
	2	82.64	94.68	-12.04	-13.44	80.73	71.45	9.28	9.06	79.76	86.33	-6.57	-7.23	96.89	84.03	12.86	12.14	64.90	102.71	-37.81	-36.95	58.87	79.81	-20.94	-10.92
	3	71.35	106.84	-35.49		85.89	79.37	6.52		73.34	78.57	-5.23		94.53	85.33	9.20		61.06	103.55	-42.49		66.90	74.80	-7.90	
4	1	82.61	64.86	17.75		82.34	74.21	8.13		72.31	91.17	-18.86		75.61	96.90	-21.29		89.45	65.04	24.41		75.94	57.48	18.46	
	2	72.89	75.83	-2.94	13.11	77.19	51.13	26.06	14.95	70.51	91.14	-20.63	-20.10	77.69	95.75	-18.06	-18.48	83.28	63.94	19.34	20.51	88.64	48.84	39.80	32.28
	3	84.42	59.91	24.51		64.48	53.83	10.65		70.31	91.11	-20.80		76.98	93.07	-16.09		84.31	66.52	17.79		83.54	44.95	38.59	
5	1	69.47	92.97	-23.50		85.39	59.85	25.54		103.51	70.30	33.21		107.06	72.87	34.19		93.38	53.76	39.62		76.70	68.60	8.10	
	2	70.27	97.09	-26.82	-24.17	86.91	69.78	17.13	20.75	101.96	70.33	31.63	32.34	105.61	72.61	33.00	33.04	91.30	53.99	37.31	36.61	77.27	71.17	6.10	6.08
	3	65.27	87.47	-22.20		87.88	68.29	19.59		102.03	69.84	32.19		105.55	73.61	31.94		88.49	55.59	32.90		84.22	80.17	4.05	
6	1	71.43	94.11	-22.68		75.69	57.84	17.85		80.58	74.85	5.73		74.59	51.01	23.58		98.71	79.52	19.19		79.09	67.73	11.36	
	2	67.68	86.71	-19.03	-24.78	79.43	62.55	16.88	17.15	79.11	73.47	5.64	12.04	72.78	48.10	24.68	23.58	91.26	82.33	8.93	9.70	75.00	54.42	20.58	18.11
	3	66.75	99.38	-32.63		78.61	61.88	16.73		90.23	65.49	24.74		72.12	49.63	22.49		85.70	84.71	0.99		78.04	55.65	22.39	
7	1	76.70	84.38	-7.68		57.63	67.34	-9.71		71.77	77.49	-5.72		71.67	57.31	14.36		85.56	72.47	13.09		95.92	64.24	31.68	
	2	77.32	86.71	-9.39	-8.54	67.81	68.40	-0.59	-6.46	71.34	73.79	-2.45	-4.71	77.39	59.93	17.46	19.81	85.64	67.05	18.59	15.25	101.48	61.23	40.25	37.98
	3	79.60	88.16	-8.56		58.49	67.56	-9.07		73.52	79.49	-5.97		84.52	56.90	27.62		85.37	71.30	14.07		103.09	61.09	42.00	
8	1	71.71	68.49	3.22		93.34	84.98	8.36		81.67	68.40	13.27		90.86	57.29	33.57		76.95	69.75	7.20		71.37	72.15	-0.78	
	2	79.73	75.24	4.49	10.80	89.93	79.92	10.01	10.12	81.64	69.10	12.54	12.95	91.96	59.28	32.68	32.55	75.69	68.64	7.05	7.26	73.01	72.21	0.80	0.98
	3	87.63	62.93	24.70		89.43	77.45	11.98		81.96	68.93	13.03		93.24	61.84	31.40		76.89	69.35	7.54		74.46	71.55	2.91	
**Mean**		**76.03**	**81.60**	**-5.57**	**-5.57**	**81.06**	**68.01**	**13.05**	**13.05**	**79.45**	**77.54**	**1.91**	**1.91**	**87.76**	**70.19**	**17.57**	**17.57**	**83.45**	**76.92**	**6.53**	**6.53**	**81.53**	**65.43**	**16.10**	**16.10**
**SE**		**1.38**	**2.64**	**3.53**	**5.48**	**2.15**	**1.90**	**2.19**	**3.26**	**2.13**	**1.57**	**3.36**	**5.96**	**2.31**	**2.90**	**3.32**	**5.95**	**2.15**	**3.20**	**4.75**	**8.49**	**2.28**	**2.07**	**3.79**	**6.58**

Group 8 Cmpd D1 (10 µg/kg) ip, QD	Trial	Force (g)		Paw Wt Distribution (g)		Force (g)		Paw Wt Distribution (g)		Force (g)		Paw Wt Distribution (g)		Force (g)		Paw Wt Distribution (g)		Force (g)		Paw Wt Distribution (g)		Force (g)		Paw Wt Distribution (g)	
	#	Left	Right	Per Trial	Mean	Left	Right	Per Trial	Mean	Left	Right	Per Trial	Mean	Left	Right	Per Trial	Mean	Left	Right	Per Trial	Mean	Left	Right	Per Trial	Mean
Animal 1	1	72.90	64.34	8.56		87.08	82.24	4.84		98.65	65.87	32.78		82.09	57.82	24.27		67.27	89.92	-22.65		88.63	65.24	23.39	
	2	85.34	63.98	21.36	**16.64**	100.40	66.27	34.13	**26.97**	107.88	67.58	40.30	**34.39**	82.89	60.82	22.07	**22.08**	60.13	82.62	-22.49	**-30.59**	73.18	59.16	14.02	**19.89**
	3	82.70	62.69	20.01		112.39	70.44	41.95		99.70	69.60	30.10		84.74	64.83	19.91		58.28	104.90	-46.62		87.09	64.82	22.27	
2	1	69.06	108.72	-39.66		85.77	80.70	5.07		88.79	77.50	11.29		73.63	63.98	9.65		53.76	76.81	-23.05		93.65	67.78	25.87	
	2	71.14	104.82	-33.68	**-43.34**	93.26	71.48	21.78	**23.66**	91.78	77.54	14.24	**17.37**	85.74	64.94	20.80	**10.83**	56.62	77.74	-21.12	**-15.87**	94.18	67.42	26.76	**23.73**
	3	61.14	117.83	-56.69		107.38	63.24	44.14		101.30	74.73	26.57		74.15	72.10	2.05		59.69	63.14	-3.45		85.87	67.32	18.55	
3	1	83.19	78.00	5.19		98.33	82.18	16.15		81.18	86.46	-5.28		103.14	94.78	8.36		112.88	72.36	40.52		97.87	77.42	20.45	
	2	99.37	82.84	16.53	**16.08**	103.38	85.41	17.97	**19.73**	84.99	90.61	-5.62	**-7.33**	104.66	93.45	11.21	**9.25**	113.03	74.03	39.00	**39.13**	99.63	68.11	31.52	**26.82**
	3	104.88	78.36	26.52		102.56	77.48	25.08		81.45	92.53	-11.08		102.83	94.66	8.17		113.53	75.67	37.86		114.21	85.73	28.48	
4	1	69.45	72.66	-3.21		72.57	69.18	3.39		83.60	76.18	7.42		82.58	70.77	11.81		92.99	78.26	14.73		80.36	71.96	8.40	
	2	73.52	61.62	11.90	**4.06**	75.05	65.77	9.28	**5.66**	84.96	76.18	8.78	**7.05**	82.00	70.03	11.97	**12.82**	89.13	77.30	11.83	**13.43**	81.41	71.37	10.04	**11.91**
	3	69.81	66.31	3.50		73.88	69.57	4.31		80.37	75.43	4.94		83.70	69.02	14.68		91.38	77.65	13.73		87.34	70.05	17.29	
5	1	70.77	59.95	10.82		79.30	69.69	9.61		111.24	58.62	52.62		95.26	51.25	44.01		93.92	60.49	33.43		74.81	64.73	10.08	
	2	59.48	66.82	-7.34	**6.92**	79.97	68.43	11.54	**10.02**	111.47	74.53	36.94	**44.15**	85.60	56.75	28.85	**36.14**	93.60	58.41	35.19	**35.28**	85.00	66.89	18.11	**13.90**
	3	65.60	48.32	17.28		78.37	69.47	8.90		109.11	66.21	42.90		90.22	54.67	35.55		94.89	57.67	37.22		83.84	70.33	13.51	
6	1	59.94	66.67	-6.73		78.43	57.14	21.29		101.25	84.50	16.75		110.38	71.74	38.64		66.44	92.84	-26.40		78.91	66.50	12.41	
	2	75.14	65.56	9.58	**-5.01**	85.92	49.46	36.46	**23.78**	104.85	80.85	24.00	**16.67**	112.08	78.57	33.51	**32.85**	66.47	93.79	-27.32	**-28.85**	82.30	66.05	16.25	**12.71**
	3	63.80	81.69	-17.89		75.73	62.14	13.59		100.88	91.61	9.27		107.86	81.47	26.39		64.95	97.78	-32.83		83.83	74.37	9.46	
7	1	79.74	66.62	13.12		79.91	75.15	4.76		52.98	81.49	-28.51		60.19	70.41	-10.22		73.65	82.26	-8.61		69.24	82.40	-13.16	
	2	79.47	67.59	11.88	**12.90**	80.31	76.60	3.71	**7.58**	48.67	80.32	-31.65	**-25.98**	61.39	72.69	-11.30	**-11.30**	59.32	76.41	-17.09	**-14.66**	68.72	80.79	-12.07	**-14.08**
	3	79.94	66.25	13.69		89.99	75.73	14.26		59.56	77.33	-17.77		61.38	73.77	-12.39		61.58	79.86	-18.28		67.73	84.75	-17.02	
8	1	61.11	79.61	-18.50		81.89	62.74	19.15		86.74	49.33	37.41		87.65	64.22	23.43		91.49	61.68	29.81		70.85	66.27	4.58	
	2	64.65	56.83	7.82	**6.41**	77.05	53.88	23.17	**18.72**	78.61	53.56	25.05	**32.84**	90.23	64.78	25.45	**24.14**	82.73	64.44	18.29	**21.73**	72.61	67.43	5.18	**11.85**
	3	81.55	51.65	29.90		75.18	61.33	13.85		85.43	49.36	36.07		90.72	67.17	23.55		84.14	67.05	17.09		91.23	65.43	25.80	
**Mean**		**74.32**	**72.49**	**1.83**	**1.83**	**86.42**	**69.41**	**17.02**	**17.02**	**88.98**	**74.08**	**14.90**	**14.90**	**87.30**	**70.20**	**17.10**	**17.10**	**79.24**	**76.80**	**2.45**	**2.45**	**83.85**	**70.51**	**13.34**	**13.34**
**SE**		**2.38**	**3.50**	**4.40**	**6.92**	**2.40**	**1.87**	**2.48**	**2.89**	**3.52**	**2.50**	**4.67**	**8.23**	**3.01**	**2.39**	**3.08**	**5.36**	**3.92**	**2.58**	**5.66**	**10.01**	**2.30**	**1.41**	**2.63**	**4.41**

Group 9 Cmpd E1 (50 mg/kg) po, QD	Trial	Force (g)		Paw Wt Distribution (g)		Force (g)		Paw Wt Distribution (g)		Force (g)		Paw Wt Distribution (g)		Force (g)		Paw Wt Distribution (g)		Force (g)		Paw Wt Distribution (g)		Force (g)		Paw Wt Distribution (g)	
	#	Left	Right	Per Trial	Mean	Left	Right	Per Trial	Mean	Left	Right	Per Trial	Mean	Left	Right	Per Trial	Mean	Left	Right	Per Trial	Mean	Left	Right	Per Trial	Mean
Animal 1	1	122.06	91.62	30.44		81.66	59.74	21.92		99.70	66.41	33.29		95.24	58.18	37.06		90.83	62.25	28.58		87.85	67.86	19.99	
	2	80.50	61.05	19.45	**28.40**	90.59	55.94	34.65	**29.47**	102.31	69.14	33.17	**32.50**	95.63	63.66	31.97	**33.08**	93.99	62.41	31.58	**30.17**	82.82	73.69	9.13	**16.73**
	3	102.80	67.50	35.30		91.67	59.84	31.83		102.30	71.25	31.05		96.23	66.02	30.21		95.36	65.00	30.36		91.99	70.93	21.06	
2	1	87.37	77.70	9.67		95.95	66.68	29.27		101.88	93.37	8.51		90.96	64.67	26.29		111.43	60.49	50.94		93.31	75.23	18.08	
	2	86.72	79.07	7.65	**-1.21**	92.22	74.51	17.71	**20.48**	98.32	89.60	8.72	**9.05**	97.53	60.77	36.76	**29.71**	93.02	71.09	21.93	**30.78**	106.89	78.37	28.52	**25.65**
	3	68.84	89.80	-20.96		92.72	78.25	14.47		99.96	90.05	9.91		89.38	63.31	26.07		85.13	65.65	19.48		110.47	80.12	30.35	
3	1	80.10	96.87	-16.77		67.60	65.81	1.79		97.46	61.20	36.26		91.53	60.21	31.32		109.47	62.72	46.75		101.98	60.06	41.92	
	2	64.00	83.89	-19.89	**-21.42**	87.30	59.23	28.07	**19.13**	96.91	65.34	31.57	**36.87**	95.59	52.27	43.32	**34.56**	79.54	65.02	14.52	**29.83**	90.10	58.29	31.81	**34.98**
	3	73.11	100.70	-27.59		85.28	57.76	27.52		98.73	55.95	42.78		90.21	61.16	29.05		87.64	59.41	28.23		92.68	61.47	31.21	
4	1	87.13	99.06	-11.93		100.02	68.18	31.84		117.01	64.30	52.71		79.26	55.67	23.59		79.83	72.98	6.85		68.83	69.86	-1.03	
	2	99.55	83.25	16.30	**-3.23**	106.80	75.05	31.75	**30.87**	115.77	67.06	48.71	**53.75**	90.97	61.82	29.15	**25.62**	82.75	74.08	8.67	**11.22**	73.72	74.46	-0.74	**-1.17**
	3	78.24	92.30	-14.06		104.47	75.44	29.03		125.99	66.17	59.82		92.97	68.86	24.11		88.34	70.19	18.15		71.39	73.14	-1.75	
5	1	55.21	82.76	-27.55		93.99	55.24	38.75		98.14	68.11	30.03		94.35	75.31	19.04		129.74	63.85	65.89		106.11	74.72	31.39	
	2	53.86	85.51	-31.65	**-27.99**	91.05	57.62	33.43	**34.37**	97.48	67.45	30.03	**29.88**	98.34	76.32	22.02	**21.89**	124.33	69.11	55.22	**60.16**	107.76	71.99	35.77	**35.96**
	3	58.67	83.45	-24.78		91.52	60.58	30.94		96.75	67.18	29.57		93.56	68.95	24.61		127.10	67.74	59.36		109.94	69.21	40.73	
6	1	101.66	78.04	23.62		77.90	90.69	-12.79		100.16	91.29	8.87		89.30	80.35	8.95		83.66	55.09	28.57		83.34	78.17	5.17	
	2	88.53	76.11	12.42	**19.63**	86.15	87.29	-1.14	**-7.07**	97.56	90.49	7.07	**7.48**	109.67	86.75	22.92	**11.61**	80.95	62.96	17.99	**22.00**	97.17	72.04	25.13	**15.48**
	3	95.26	72.40	22.86		76.48	83.77	-7.29		95.63	89.12	6.51		82.83	79.88	2.95		85.99	66.54	19.45		86.14	69.99	16.15	
7	1	49.34	79.76	-30.42		93.09	85.40	7.69		81.14	91.39	-10.25		87.10	72.65	14.45		85.19	62.82	22.37		73.22	73.59	-0.37	
	2	51.09	78.91	-27.82	**-28.86**	89.60	85.22	4.38	**5.52**	77.19	95.76	-18.57	**-18.38**	83.29	68.33	14.96	**11.98**	85.56	65.75	19.81	**19.81**	71.83	72.37	-0.54	**-2.81**
	3	49.49	77.82	-28.33		88.91	84.41	4.50		74.13	100.44	-26.31		82.25	75.72	6.53		84.15	66.89	17.26		66.97	74.48	-7.51	
8	1	97.72	66.19	31.53		71.82	86.66	-14.84		69.53	93.61	-24.08		66.34	93.65	-27.31		79.69	84.98	-5.29		81.88	62.26	19.62	
	2	98.54	68.37	30.17	**24.00**	57.92	79.90	-21.98	**-22.41**	69.72	98.93	-29.21	**-28.86**	60.56	95.11	-34.55	**-29.32**	77.23	86.74	-9.51	**-7.89**	78.49	48.76	29.73	**27.19**
	3	86.40	76.09	10.31		52.79	83.20	-30.41		64.38	97.68	-33.30		62.18	88.29	-26.11		77.62	86.49	-8.87		85.97	53.75	32.22	
**Mean**		**79.84**	**81.18**	**-1.33**	**-1.33**	**86.15**	**72.35**	**13.80**	**13.80**	**94.92**	**79.64**	**15.29**	**15.29**	**88.14**	**70.75**	**17.39**	**17.39**	**92.44**	**67.93**	**24.51**	**24.51**	**88.37**	**69.37**	**19.00**	**19.00**
**SE**		**4.08**	**2.11**	**4.90**	**8.28**	**2.69**	**2.45**	**4.14**	**7.13**	**3.11**	**2.99**	**5.58**	**10.03**	**2.36**	**2.42**	**4.18**	**7.35**	**3.25**	**1.68**	**4.09**	**6.85**	**2.81**	**1.63**	**3.09**	**5.27**

Group 10 Cmpd F1 (50 mg/kg) po, QD	Trial	Force (g)		Paw Wt Distribution (g)		Force (g)		Paw Wt Distribution (g)		Force (g)		Paw Wt Distribution (g)		Force (g)		Paw Wt Distribution (g)		Force (g)		Paw Wt Distribution (g)		Force (g)		Paw Wt Distribution (g)	
	#	Left	Right	Per Trial	Mean	Left	Right	Per Trial	Mean	Left	Right	Per Trial	Mean	Left	Right	Per Trial	Mean	Left	Right	Per Trial	Mean	Left	Right	Per Trial	Mean
Animal 1	1	73.88	111.20	-37.32		109.44	66.62	42.82		73.89	102.65	-28.76		86.31	73.38	12.93		88.13	84.66	3.47		94.50	69.71	24.79	
	2	63.56	119.49	-55.93	**-50.74**	98.27	68.33	29.94	**30.06**	74.53	104.61	-30.08	**-29.44**	92.37	65.04	27.33	**19.54**	89.15	87.51	1.64	**2.80**	95.95	71.85	24.10	**25.07**
	3	60.37	119.33	-58.96		91.58	74.17	17.41		75.21	104.69	-29.48		88.08	69.71	18.37		85.42	82.14	3.28		97.23	70.91	26.32	
2	1	87.42	72.08	15.34		107.67	68.72	38.95		69.24	79.96	-10.72		79.47	84.03	-4.56		89.48	78.53	10.95		84.21	81.28	2.93	
	2	84.74	73.50	11.24	**13.80**	100.26	76.88	23.38	**26.57**	68.52	84.88	-16.36	**-21.56**	77.39	81.88	-4.49	**-4.96**	87.24	81.38	5.86	**9.10**	86.50	83.21	3.29	**5.05**
	3	85.54	70.72	14.82		95.63	78.24	17.39		64.37	101.97	-37.60		77.59	83.41	-5.82		93.34	82.85	10.49		91.81	82.88	8.93	
3	1	124.83	81.61	43.22		95.90	65.31	30.59		97.48	67.24	30.24		92.58	78.72	13.86		76.26	87.99	-11.73		88.67	65.96	22.71	
	2	129.41	85.21	44.20	**28.20**	96.79	69.44	27.35	**30.13**	99.39	67.62	31.77	**32.66**	93.75	78.95	14.80	**14.49**	92.40	93.66	-1.26	**-4.63**	84.48	68.36	16.12	**19.27**
	3	99.91	102.72	-2.81		102.09	69.65	32.44		102.56	66.60	35.96		90.71	75.91	14.80		90.02	90.92	-0.90		86.58	67.61	18.97	
4	1	81.67	81.38	0.29		96.74	71.74	25.00		90.88	74.04	16.84		94.52	55.54	38.98		74.66	73.19	1.47		83.62	97.37	-13.75	
	2	59.68	84.21	-24.53	**-10.74**	89.63	81.12	8.51	**17.51**	75.95	70.15	5.80	**7.58**	90.04	85.04	5.00	**19.90**	73.43	70.94	2.49	**6.23**	80.42	95.33	-14.91	**-14.63**
	3	76.09	84.08	-7.99		99.17	80.14	19.03		83.19	83.08	0.11		92.68	76.97	15.71		84.30	69.57	14.73		81.70	96.94	-15.24	
5	1	78.46	83.00	-4.54		65.68	79.21	-13.53		117.68	74.50	43.18		68.49	57.52	10.97		77.29	66.81	10.48		85.04	79.54	5.50	
	2	77.99	82.99	-5.00	**-2.35**	67.94	76.35	-8.41	**-12.03**	98.33	77.31	21.02	**32.17**	89.77	66.75	23.02	**14.75**	78.34	69.87	8.47	**7.86**	87.77	79.04	8.73	**6.49**
	3	90.85	88.36	2.49		64.35	78.50	-14.15		103.21	70.90	32.31		82.79	72.52	10.27		78.74	74.11	4.63		86.80	81.56	5.24	
6	1	94.74	61.03	33.71		79.81	40.06	39.75		104.95	73.41	31.54		90.63	63.86	26.77		88.42	75.34	13.08		88.35	87.32	1.03	
	2	89.64	64.43	25.21	**24.45**	85.39	42.26	43.13	**39.48**	95.98	78.10	17.88	**19.12**	90.81	63.86	26.95	**22.54**	91.92	76.82	15.10	**14.59**	90.14	74.01	16.13	**13.23**
	3	85.99	71.57	14.42		85.28	49.73	35.55		93.15	85.20	7.95		89.79	75.89	13.90		79.70	64.10	15.60		93.74	71.22	22.52	
7	1	66.89	81.43	-14.54		74.99	91.96	-16.97		74.58	88.23	-13.65		92.30	74.70	17.60		87.01	77.42	9.59		93.40	88.23	5.17	
	2	65.64	75.26	-9.62	**-12.13**	78.39	92.44	-14.05	**-13.78**	79.02	102.34	-23.32	**-20.37**	91.72	71.04	20.68	**20.14**	86.54	75.39	11.15	**15.59**	76.39	62.23	14.16	**9.32**
	3	64.67	76.89	-12.22		82.67	92.99	-10.32		81.76	105.90	-24.14		94.33	72.20	22.13		95.47	69.45	26.02		92.85	84.21	8.64	
8	1	63.22	80.28	-17.06		90.10	86.10	4.00		95.71	73.56	22.15		80.53	88.60	-8.07		118.04	64.19	53.85		101.75	59.05	42.70	
	2	89.45	53.14	36.31	**15.09**	89.88	80.75	9.13	**8.55**	96.97	77.46	19.51	**16.61**	79.75	84.88	-5.13	**-4.76**	108.97	67.08	41.89	**45.31**	100.02	58.36	41.66	**38.77**
	3	81.71	55.70	26.01		91.07	78.55	12.52		90.75	82.57	8.18		83.51	84.59	-1.08		107.49	67.30	40.19		95.39	63.43	31.96	
**Mean**		**82.35**	**81.65**	**0.70**	**0.70**	**89.11**	**73.30**	**15.81**	**15.81**	**87.80**	**83.21**	**4.60**	**4.60**	**87.08**	**74.37**	**12.71**	**12.71**	**88.41**	**76.30**	**12.11**	**12.11**	**89.47**	**76.65**	**12.82**	**12.82**
**SE**		**3.66**	**3.53**	**5.68**	**9.11**	**2.53**	**2.82**	**4.08**	**7.06**	**2.85**	**2.73**	**5.11**	**8.84**	**1.39**	**1.84**	**2.55**	**3.95**	**2.24**	**1.75**	**3.05**	**5.26**	**1.29**	**2.36**	**3.19**	**5.56**

Table 72. Individual animal data for MMT/MCLT Trial 1 Day 14 data

Group 1 Vehicle (Saline) ip, QD	Trial	Day 14 (Pre tx)				Day 14 (1 hr)				Day 14 (3 hr)				Day 14 (5 hr)				Day 14 (24 hr)			
		Force (g)		Paw Wt Distribution (g)		Force (g)		Paw Wt Distribution (g)		Force (g)		Paw Wt Distribution (g)		Force (g)		Paw Wt Distribution (g)		Force (g)		Paw Wt Distribution (g)	
	#	Left	Right	Per Trial	Mean	Left	Right	Per Trial	Mean	Left	Right	Per Trial	Mean	Left	Right	Per Trial	Mean	Left	Right	Per Trial	Mean
Animal 1	1	111.52	79.81	31.71		110.87	80.78	30.09		105.04	82.74	22.30		88.47	84.72	3.75		87.47	83.51	3.96	
	2	107.46	77.10	30.36	**30.98**	103.91	80.38	23.53	**26.86**	115.35	80.39	34.96	**29.52**	88.70	84.69	4.01	**4.51**	84.98	82.23	2.75	**3.19**
	3	106.12	75.26	30.86		108.91	81.96	26.95		113.48	82.18	31.30		89.69	83.93	5.76		87.20	84.33	2.87	
2	1	97.14	91.51	5.63		86.05	78.41	7.64		73.62	62.31	11.31		88.25	73.97	14.28		96.01	89.29	6.72	
	2	115.29	79.17	36.12	**22.17**	91.09	87.08	4.01	**6.02**	75.25	63.66	11.59	**12.88**	92.46	82.28	10.18	**12.52**	97.89	84.77	13.12	**12.55**
	3	106.05	81.29	24.76		92.57	86.15	6.42		80.12	64.38	15.74		93.63	80.54	13.09		98.44	80.63	17.81	
3	1	92.91	87.41	5.50		92.39	82.61	9.78		92.36	79.27	13.09		96.30	91.72	4.58		85.72	71.84	13.88	
	2	106.24	82.34	23.90	**19.51**	93.90	83.75	10.15	**9.25**	107.78	78.10	29.68	**17.12**	95.78	91.24	4.54	**4.05**	83.44	73.75	9.69	**10.52**
	3	108.96	79.82	29.14		93.42	85.59	7.83		94.51	85.93	8.58		92.46	89.42	3.04		86.78	78.80	7.98	
4	1	96.13	79.07	17.06		114.70	97.70	17.00		97.38	79.32	18.06		118.07	76.02	42.05		110.75	100.52	10.23	
	2	102.32	76.68	25.64	**22.46**	111.26	82.35	28.91	**24.49**	96.75	82.75	14.00	**16.06**	113.42	80.47	32.95	**34.84**	108.62	104.17	4.45	**6.40**
	3	101.25	76.57	24.68		105.84	78.28	27.56		98.71	82.59	16.12		111.99	82.47	29.52		108.27	103.74	4.53	
5	1	90.72	88.71	2.01		93.83	79.06	14.77		77.78	92.45	-14.67		98.19	84.45	13.74		94.26	91.62	2.64	
	2	92.37	89.46	2.91	**2.19**	92.35	81.80	10.55	**10.85**	78.35	93.05	-14.70	**-14.15**	103.52	84.93	18.59	**14.42**	92.51	91.55	0.96	**2.27**
	3	90.57	88.91	1.66		91.37	84.15	7.22		78.80	91.88	-13.08		97.84	86.91	10.93		92.55	89.34	3.21	
6	1	94.70	63.06	31.64		91.84	83.05	8.79		86.37	66.49	19.88		95.25	87.33	7.92		89.30	57.95	31.35	
	2	94.37	62.02	32.35	**30.93**	90.62	81.35	9.27	**9.70**	86.74	65.87	20.87	**21.06**	88.78	78.60	10.18	**9.20**	103.10	55.81	47.29	**42.64**
	3	93.91	65.11	28.80		88.31	77.27	11.04		87.50	65.06	22.44		90.05	80.56	9.49		105.92	56.65	49.27	
7	1	103.79	76.38	27.41		87.50	77.05	10.45		94.93	87.96	6.97		93.97	90.00	3.97		109.50	79.44	30.06	
	2	102.05	74.79	27.26	**28.36**	87.68	76.21	11.47	**12.15**	96.59	88.07	8.52	**7.23**	96.22	91.30	4.92	**4.78**	106.44	80.85	25.59	**26.86**
	3	104.59	74.18	30.41		89.85	75.33	14.52		95.46	89.26	6.20		96.69	91.23	5.46		106.54	81.62	24.92	
8	1	97.65	89.13	8.52		95.23	85.04	10.19		103.93	78.22	25.71		93.71	68.04	25.67		72.91	78.38	-5.47	
	2	97.15	88.29	8.86	**8.15**	103.24	85.88	17.36	**14.13**	101.45	82.26	19.19	**21.91**	88.84	71.15	17.69	**20.44**	72.58	80.37	-7.79	**-7.31**
	3	99.72	92.64	7.08		104.14	89.29	14.85		101.85	81.02	20.83		89.44	71.48	17.96		73.96	82.62	-8.66	
**Mean**		**100.54**	**79.95**	**20.59**	**20.59**	**96.70**	**82.52**	**14.18**	**14.18**	**93.34**	**79.38**	**13.95**	**13.95**	**95.91**	**82.81**	**13.09**	**13.09**	**93.96**	**81.82**	**12.14**	**12.14**
**SE**		**1.39**	**1.76**	**2.40**	**3.72**	**1.77**	**1.00**	**1.57**	**2.65**	**2.43**	**1.99**	**2.69**	**4.64**	**1.67**	**1.40**	**2.12**	**3.70**	**2.43**	**2.60**	**3.17**	**5.57**

Group 2 Morphine (10 mg/kg) ip, QD	Trial	Force (g)		Paw Wt Distribution (g)		Force (g)		Paw Wt Distribution (g)		Force (g)		Paw Wt Distribution (g)		Force (g)		Paw Wt Distribution (g)		Force (g)		Paw Wt Distribution (g)	
	#	Left	Right	Per Trial	Mean	Left	Right	Per Trial	Mean	Left	Right	Per Trial	Mean	Left	Right	Per Trial	Mean	Left	Right	Per Trial	Mean
Animal 1	1	83.29	84.86	-1.57		84.36	81.81	2.55		84.64	94.86	-10.22		68.97	106.13	-37.16		98.93	85.84	13.09	
	2	73.22	85.93	-12.71	**-6.08**	87.86	86.07	1.79	**2.48**	87.23	98.56	-11.33	**-11.98**	73.29	102.49	-29.20	**-30.71**	93.71	86.04	7.67	**9.08**
	3	81.03	84.98	-3.95		89.54	86.44	3.10		85.17	99.57	-14.40		77.01	102.77	-25.76		91.59	85.11	6.48	
2	1	82.82	77.78	5.04		81.58	108.49	-26.91		82.48	63.77	18.71		83.42	70.44	12.98		76.28	71.50	4.78	
	2	87.96	84.90	3.06	**9.60**	82.70	106.53	-23.83	**-25.45**	88.42	57.54	30.88	**22.31**	77.38	73.46	3.92	**8.49**	83.42	74.24	9.18	**6.32**
	3	100.79	80.09	20.70		82.87	108.49	-25.62		86.32	68.97	17.35		85.14	76.57	8.57		86.17	81.18	4.99	
3	1	108.33	87.44	20.89		101.31	85.93	15.38		90.56	98.72	-8.16		68.56	112.49	-43.93		79.65	102.03	-22.38	
	2	107.16	92.07	15.09	**14.39**	100.08	85.85	14.23	**12.96**	95.80	97.60	-1.80	**-4.21**	67.94	110.43	-42.49	**-43.42**	82.86	103.47	-20.61	**-18.55**
	3	104.37	97.18	7.19		97.93	88.66	9.27		95.95	98.62	-2.67		71.20	115.03	-43.83		83.71	96.38	-12.67	
4	1	90.55	56.83	33.72		108.34	94.63	13.71		71.79	93.63	-21.84		79.55	67.74	11.81		95.03	80.93	14.10	
	2	97.13	56.80	40.33	**35.05**	90.79	83.05	7.74	**9.03**	84.17	88.34	-4.17	**-10.88**	82.61	67.60	15.01	**14.55**	94.41	82.06	12.35	**11.47**
	3	94.56	63.46	31.10		87.51	81.87	5.64		78.66	85.29	-6.63		88.12	71.28	16.84		87.45	79.48	7.97	
5	1	80.63	82.00	-1.37		85.08	75.14	9.94		77.20	68.97	8.23		81.47	75.22	6.25		90.58	63.59	26.99	
	2	67.37	82.34	-14.97	**-6.35**	88.33	78.64	9.69	**8.48**	79.61	72.88	6.73	**6.70**	84.95	75.12	9.83	**10.74**	82.90	61.33	21.57	**25.56**
	3	66.71	69.43	-2.72		84.75	78.95	5.80		79.79	74.65	5.14		90.88	74.75	16.13		89.54	61.42	28.12	
6	1	98.34	82.27	16.07		86.83	115.49	-28.66		87.40	89.61	-2.21		77.98	53.16	24.82		98.26	76.52	21.74	
	2	94.06	83.00	11.06	**12.95**	88.79	111.68	-22.89	**-23.38**	88.75	91.11	-2.36	**-7.17**	71.10	67.12	3.98	**13.65**	87.25	80.75	6.50	**13.14**
	3	93.38	81.65	11.73		87.10	105.68	-18.58		82.99	99.94	-16.95		77.17	65.03	12.14		91.25	80.08	11.17	
7	1	96.89	84.21	12.68		105.98	74.72	31.26		107.46	85.11	22.35		113.47	82.04	31.43		82.03	64.79	17.24	
	2	103.33	81.44	21.89	**12.69**	101.25	84.76	16.49	**28.17**	103.39	87.14	16.25	**17.49**	116.18	76.74	39.44	**37.07**	90.55	68.48	22.07	**21.24**
	3	87.66	84.17	3.49		109.55	72.78	36.77		89.53	75.67	13.86		122.59	82.25	40.34		99.19	74.78	24.41	
8	1	66.09	79.35	-13.26		74.75	84.17	-9.42		93.81	98.95	-5.14		79.81	72.89	6.92		75.94	81.20	-5.26	
	2	66.58	76.54	-9.96	**-9.28**	73.18	88.15	-14.97	**-14.09**	93.22	99.82	-6.60	**-7.31**	77.73	74.24	3.49	**4.91**	76.03	78.66	-2.63	**-3.84**
	3	71.13	75.76	-4.63		76.27	94.15	-17.88		92.51	102.71	-10.20		78.56	74.24	4.32		76.30	79.94	-3.64	
**Mean**		**87.64**	**79.77**	**7.87**	**7.87**	**89.86**	**90.09**	**-0.22**	**-0.22**	**87.79**	**87.17**	**0.62**	**0.62**	**83.13**	**81.22**	**1.91**	**1.91**	**87.21**	**79.16**	**8.05**	**8.05**
**SE**		**2.79**	**1.98**	**3.10**	**5.22**	**2.08**	**2.58**	**3.79**	**6.70**	**1.66**	**2.72**	**2.76**	**4.69**	**2.98**	**3.49**	**5.18**	**9.24**	**1.51**	**2.28**	**2.82**	**4.95**

Group 3 Cmpd A1 (0.3 mg/kg) ip, QD	Trial	Force (g)		Paw Wt Distribution (g)		Force (g)		Paw Wt Distribution (g)		Force (g)		Paw Wt Distribution (g)		Force (g)		Paw Wt Distribution (g)		Force (g)		Paw Wt Distribution (g)	
	#	Left	Right	Per Trial	Mean	Left	Right	Per Trial	Mean	Left	Right	Per Trial	Mean	Left	Right	Per Trial	Mean	Left	Right	Per Trial	Mean
Animal 1	1	84.75	60.67	24.08		51.50	64.93	-13.43		62.58	79.56	-16.98		71.31	49.42	21.89		92.57	56.36	36.21	
	2	85.28	67.20	18.08	**15.07**	54.38	69.15	-14.77	**-14.85**	62.42	80.93	-18.51	**-19.65**	84.34	54.13	30.21	**24.36**	98.31	61.58	36.73	**35.79**
	3	77.84	74.78	3.06		55.63	71.98	-16.35		65.06	88.51	-23.45		74.68	53.70	20.98		94.68	60.26	34.42	
2	1	76.12	70.11	6.01		50.70	85.95	-35.25		62.51	73.56	-11.05		66.76	82.45	-15.69		42.54	85.16	-42.62	
	2	72.36	68.58	3.78	**3.48**	52.39	89.55	-37.16	**-35.94**	62.27	73.10	-10.83	**-10.01**	66.94	82.22	-15.28	**-10.68**	42.71	87.50	-44.79	**-44.30**
	3	74.75	74.11	0.64		51.93	87.35	-35.42		62.07	70.21	-8.14		68.89	69.96	-1.07		44.40	89.90	-45.50	
3	1	97.46	62.84	34.62		76.64	86.26	-9.62		77.83	84.17	-6.34		103.91	80.72	23.19		69.03	65.00	4.03	
	2	94.11	65.88	28.23	**27.16**	71.76	94.54	-22.78	**-13.05**	79.16	87.52	-8.36	**-7.62**	97.45	67.91	29.54	**26.32**	70.49	65.23	5.26	**4.87**
	3	89.41	70.79	18.62		86.45	93.21	-6.76		79.55	87.70	-8.15		100.29	74.07	26.22		71.45	66.12	5.33	
4	1	77.25	69.51	7.74		85.77	65.70	20.07		89.32	91.33	-2.01		103.32	75.69	27.63		79.65	75.62	4.03	
	2	87.81	67.37	20.44	**12.88**	98.50	74.86	23.64	**22.61**	92.51	96.09	-3.58	**-2.99**	86.46	74.97	11.49	**15.50**	83.55	79.04	4.51	**8.22**
	3	79.08	68.63	10.45		86.11	61.98	24.13		83.75	87.14	-3.39		89.23	81.84	7.39		83.01	66.89	16.12	
5	1	76.69	100.13	-23.44		89.70	83.86	5.84		71.64	98.32	-26.68		75.25	100.65	-25.40		87.37	89.44	-2.07	
	2	79.01	98.39	-19.38	**-21.98**	89.59	84.98	4.61	**5.04**	73.03	100.69	-27.66	**-28.78**	74.18	98.35	-24.17	**-24.16**	86.74	88.58	-1.84	**-2.13**
	3	80.46	103.59	-23.13		89.23	84.56	4.67		70.76	102.77	-32.01		75.52	98.42	-22.90		87.77	90.25	-2.48	
6	1	69.43	91.13	-21.70		96.81	85.57	11.24		106.63	73.94	32.69		88.14	66.85	21.29		88.64	69.27	19.37	
	2	72.36	91.14	-18.78	**-19.53**	93.11	82.31	10.80	**11.05**	105.31	74.90	30.41	**33.52**	91.57	72.65	18.92	**20.25**	91.86	63.39	28.47	**21.34**
	3	72.69	90.80	-18.11		96.44	85.34	11.10		111.86	74.40	37.46		90.60	70.06	20.54		86.27	70.10	16.17	
7	1	70.43	72.31	-1.88		86.86	71.33	15.53		75.56	103.23	-27.67		77.77	66.30	11.47		75.49	87.85	-12.36	
	2	65.59	62.76	2.83	**3.85**	82.29	71.02	11.27	**10.60**	79.38	102.88	-23.50	**-24.64**	84.45	74.05	10.40	**7.96**	74.99	80.33	-5.34	**-7.76**
	3	78.63	68.04	10.59		89.00	84.01	4.99		77.19	99.93	-22.74		82.97	80.95	2.02		75.64	81.22	-5.58	
8	1	75.38	51.13	24.25		81.17	57.15	24.02		96.41	42.71	53.70		52.57	55.40	-2.83		78.74	65.46	13.28	
	2	67.81	61.20	6.61	**12.16**	84.04	55.74	28.30	**23.66**	90.28	51.79	38.49	**38.72**	43.21	83.36	-40.15	**-26.16**	79.80	57.00	22.80	**19.70**
	3	68.60	62.99	5.61		76.01	57.35	18.66		84.66	60.69	23.97		46.05	81.55	-35.50		78.40	55.37	23.03	
**Mean**		**78.05**	**73.92**	**4.13**	**4.13**	**78.17**	**77.03**	**1.14**	**1.14**	**80.07**	**82.75**	**-2.68**	**-2.68**	**78.99**	**74.82**	**4.17**	**4.17**	**77.67**	**73.21**	**4.47**	**4.47**
**SE**		**1.68**	**2.87**	**3.53**	**6.03**	**3.32**	**2.44**	**4.10**	**7.32**	**3.02**	**3.31**	**5.07**	**9.02**	**3.35**	**2.78**	**4.47**	**7.61**	**3.14**	**2.47**	**4.76**	**8.54**

Group 4 Cmpd A2 (0.6 mg/kg) ip, QD	Trial	Force (g)		Paw Wt Distribution (g)		Force (g)		Paw Wt Distribution (g)		Force (g)		Paw Wt Distribution (g)		Force (g)		Paw Wt Distribution (g)		Force (g)		Paw Wt Distribution (g)	
	#	Left	Right	Per Trial	Mean	Left	Right	Per Trial	Mean	Left	Right	Per Trial	Mean	Left	Right	Per Trial	Mean	Left	Right	Per Trial	Mean
Animal 1	1	87.64	64.49	23.15		106.99	68.87	38.12		67.79	93.67	-25.88		73.08	69.53	3.55		73.51	71.52	1.99	
	2	83.41	70.81	12.60	15.95	107.68	64.72	42.96	41.53	72.08	89.80	-17.72	-20.22	75.58	68.77	6.81	3.68	74.61	70.30	4.31	3.46
	3	86.57	74.47	12.10		108.10	64.60	43.50		71.68	88.75	-17.07		73.40	72.72	0.68		73.60	69.53	4.07	
2	1	76.94	67.52	9.42		85.30	70.24	15.06		103.79	65.56	38.23		71.74	87.61	-15.87		62.49	95.65	-33.16	
	2	77.12	67.42	9.70	9.01	86.75	68.70	18.05	16.40	107.10	64.49	42.61	40.55	78.48	79.31	-0.83	-10.67	62.72	88.84	-26.12	-30.05
	3	78.38	70.47	7.91		84.46	68.37	16.09		106.99	66.18	40.81		69.04	84.34	-15.30		61.44	92.32	-30.88	
3	1	92.89	74.17	18.72		56.14	87.46	-31.32		81.03	59.66	21.37		78.92	65.48	13.44		85.53	65.34	20.19	
	2	90.33	69.08	21.25	17.83	57.70	94.70	-37.00	-35.12	86.37	60.53	25.84	22.24	81.92	65.76	16.16	17.14	90.94	67.85	23.09	22.02
	3	86.61	73.10	13.51		58.05	95.08	-37.03		85.52	66.00	19.52		87.22	65.39	21.83		94.27	71.50	22.77	
4	1	91.29	56.80	34.49		96.45	89.99	6.46		85.48	48.76	36.72		76.19	57.32	18.87		82.32	52.45	29.87	
	2	89.27	56.97	32.30	33.75	100.29	71.40	28.89	24.50	85.88	62.18	23.70	29.03	77.58	57.58	20.00	19.51	84.60	54.28	30.32	31.04
	3	90.92	56.46	34.46		90.81	52.65	38.16		94.84	68.18	26.66		78.28	58.61	19.67		89.43	56.51	32.92	
5	1	70.33	89.36	-19.03		78.18	79.05	-0.87		89.96	72.46	17.50		76.68	80.56	-3.88		79.90	71.18	8.72	
	2	71.29	89.05	-17.76	-16.71	80.82	81.92	-1.10	-0.89	91.34	70.30	21.04	20.23	73.39	81.00	-7.61	-6.82	81.22	68.89	12.33	11.77
	3	75.68	89.01	-13.33		80.73	81.44	-0.71		91.97	69.83	22.14		72.91	81.88	-8.97		83.14	68.87	14.27	
6	1	81.26	70.65	10.61		60.50	66.49	-5.99		71.51	60.36	11.15		79.37	69.44	9.93		79.37	61.27	18.10	
	2	84.59	71.87	12.72	14.05	66.36	76.83	-10.47	-11.19	70.18	60.41	9.77	10.62	74.64	66.06	8.58	11.14	79.06	60.08	18.98	17.44
	3	85.91	67.10	18.81		57.19	74.31	-17.12		75.05	64.10	10.95		73.54	58.63	14.91		78.11	62.87	15.24	
7	1	56.65	85.99	-29.34		68.20	64.70	3.50		87.22	67.37	19.85		74.31	84.72	-10.41		65.48	89.22	-23.74	
	2	55.79	85.98	-30.19	-30.51	77.49	72.20	5.29	3.88	99.69	47.77	51.92	30.17	73.75	88.59	-14.84	-11.37	68.54	93.14	-24.60	-24.53
	3	55.18	87.18	-32.00		69.20	66.35	2.85		88.33	69.59	18.74		73.88	82.75	-8.87		69.16	94.41	-25.25	
8	1	98.68	48.78	49.90		58.87	77.90	-19.03		99.77	72.93	26.84		92.38	62.21	30.17		94.48	62.09	32.39	
	2	99.42	49.45	49.97	47.97	60.84	80.28	-19.44	-20.30	111.43	72.09	39.34	36.50	88.93	62.84	26.09	18.75	92.94	61.31	31.63	32.60
	3	98.21	54.17	44.04		62.36	84.78	-22.42		113.59	70.27	43.32				0.00		94.14	60.37	33.77	
Mean		81.85	70.43	11.42	11.42	77.48	75.13	2.35	2.35	89.11	67.97	21.14	21.14	77.18	71.79	5.17	5.17	79.21	71.24	7.97	7.97
SE		2.61	2.57	4.96	8.92	3.57	2.15	4.97	8.80	2.78	2.23	3.99	6.79	1.19	2.18	2.86	4.71	2.16	2.76	4.65	8.41

Group 5 Cmpd B1 (5 µg/kg) ip, QD	Trial	Force (g)		Paw Wt Distribution (g)		Force (g)		Paw Wt Distribution (g)		Force (g)		Paw Wt Distribution (g)		Force (g)		Paw Wt Distribution (g)		Force (g)		Paw Wt Distribution (g)	
	#	Left	Right	Per Trial	Mean	Left	Right	Per Trial	Mean	Left	Right	Per Trial	Mean	Left	Right	Per Trial	Mean	Left	Right	Per Trial	Mean
Animal 1	1	94.44	70.15	24.29		76.47	65.68	10.79		76.15	65.81	10.34		89.00	71.01	17.99		68.21	91.50	-23.29	
	2	90.56	71.90	18.66	25.08	78.28	71.09	7.19	7.55	80.22	68.24	11.98	11.59	91.80	65.76	26.04	17.87	67.33	91.28	-23.95	-24.99
	3	104.69	72.41	32.28		78.47	73.79	4.68		84.49	72.04	12.45		92.10	82.51	9.59		69.42	97.16	-27.74	
2	1	94.35	65.38	28.97		74.80	98.49	-23.69		81.24	93.71	-12.47		90.67	64.88	25.79		78.60	77.60	1.00	
	2	86.65	72.57	14.08	15.77	68.53	99.49	-30.96	-27.05	76.58	92.75	-16.17	-15.94	101.50	62.14	39.36	34.71	89.25	56.43	32.82	19.57
	3	84.04	79.78	4.26		69.15	95.64	-26.49		78.75	97.93	-19.18		103.00	64.02	38.98		91.51	66.61	24.90	
3	1	84.80	79.02	5.78		84.68	61.82	22.86		75.98	87.43	-11.45		89.10	85.89	3.21		91.65	73.06	18.59	
	2	89.77	80.04	9.73	7.34	87.97	65.64	22.33	20.76	79.70	89.32	-9.62	-10.22	87.98	87.18	0.80	2.21	92.90	73.61	19.29	19.43
	3	88.66	82.16	6.50		89.45	72.36	17.09		82.90	92.49	-9.59		88.53	85.90	2.63		94.02	73.60	20.42	
4	1	71.29	75.48	-4.19		76.19	68.20	7.99		79.95	91.65	-11.70		99.34	67.21	32.13		77.79	62.90	14.89	
	2	67.65	75.62	-7.97	-3.88	75.91	68.00	7.91	8.07	76.73	91.45	-14.72	-14.90	97.98	68.33	29.65	30.36	79.81	63.59	16.22	14.98
	3	73.78	73.27	0.51		76.33	68.01	8.32		73.39	91.66	-18.27		99.21	69.91	29.30		79.38	65.54	13.84	
5	1	79.31	58.09	21.22		106.65	74.78	31.87		75.59	76.87	-1.28		77.01	79.84	-2.83		89.71	66.02	23.69	
	2	72.48	55.70	16.78	16.41	96.12	78.79	17.33	24.94	78.93	83.45	-4.52	-4.02	83.58	68.89	14.69	11.77	94.62	68.69	25.93	21.28
	3	68.55	57.33	11.22		92.35	66.72	25.63		77.69	83.95	-6.26		92.54	69.10	23.44		86.33	72.11	14.22	
6	1	75.71	82.79	-7.08		89.48	69.34	20.14		76.32	82.84	-6.52		82.70	57.48	25.22		73.88	64.29	9.59	
	2	75.72	79.00	-3.28	-3.40	84.40	69.36	15.04	16.54	77.08	83.27	-6.19	-6.04	83.08	54.15	28.93	30.72	83.45	61.24	22.21	18.20
	3	77.26	77.10	0.16		84.57	70.13	14.44		77.69	83.10	-5.41		86.55	48.53	38.02		86.95	64.14	22.81	
7	1	78.55	72.86	5.69		93.31	92.72	0.59		76.67	98.66	-21.99		72.17	81.48	-9.31		97.53	73.47	24.06	
	2	81.66	74.66	7.00	5.78	93.74	90.47	3.27	1.47	74.92	96.34	-21.42	-22.48	75.31	82.06	-6.75	-7.62	99.69	74.50	25.19	21.11
	3	84.36	79.72	4.64		92.99	92.43	0.56		75.59	99.62	-24.03		74.60	81.40	-6.80		92.67	78.59	14.08	
8	1	70.85	66.53	4.32		47.29	79.31	-32.02		92.12	55.89	36.23		79.68	81.97	-2.29		79.60	77.31	2.29	
	2	67.04	60.42	6.62	1.13	59.19	86.33	-27.14	-29.39	91.83	57.48	34.35	34.20	81.75	84.77	-3.02	-3.07	80.43	77.69	2.74	2.74
	3	64.55	72.09	-7.54		57.24	86.26	-29.02		90.91	58.88	32.03		82.91	86.81	-3.90		80.50	77.31	3.19	
Mean		80.28	72.25	8.03	8.03	80.57	77.70	2.86	2.86	79.64	83.12	-3.48	-3.48	87.59	72.97	14.62	14.62	84.38	72.84	11.54	11.54
SE		2.09	1.62	2.28	3.65	2.80	2.42	4.07	7.30	1.08	2.74	3.59	6.47	1.77	2.30	3.40	5.81	1.89	2.04	3.34	5.64

Group 6 Cmpd B2 (10 µg/kg) ip, QD	Trial	Force (g)		Paw Wt Distribution (g)		Force (g)		Paw Wt Distribution (g)		Force (g)		Paw Wt Distribution (g)		Force (g)		Paw Wt Distribution (g)		Force (g)		Paw Wt Distribution (g)	
	#	Left	Right	Per Trial	Mean	Left	Right	Per Trial	Mean	Left	Right	Per Trial	Mean	Left	Right	Per Trial	Mean	Left	Right	Per Trial	Mean
Animal 1	1	69.17	63.90	5.27		85.41	53.62	31.79		73.78	95.09	-21.31		68.99	75.76	-6.77		77.33	98.50	-21.17	
	2	69.12	61.85	7.27	6.75	87.78	56.11	31.67	31.54	76.19	92.22	-16.03	-17.49	68.40	74.47	-6.07	-5.72	73.13	95.62	-22.49	-25.76
	3	72.79	65.08	7.71		88.07	56.91	31.16		77.48	92.61	-15.13		68.29	72.60	-4.31		72.07	105.70	-33.63	
2	1	80.32	61.48	18.84		73.46	90.59	-17.13		83.17	95.23	-12.06		68.14	81.14	-13.00		72.90	99.11	-26.21	
	2	67.41	66.30	1.11	12.28	73.86	90.93	-17.07	-17.41	82.34	94.06	-11.72	-11.34	70.00	85.39	-15.39	-11.50	70.04	103.30	-33.26	-30.87
	3	84.93	68.04	16.89		74.24	92.26	-18.02		83.50	93.74	-10.24		74.18	80.28	-6.10		72.00	105.13	-33.13	
3	1	76.61	54.10	22.51		58.08	76.28	-18.20		89.72	61.07	28.65		78.77	84.04	-5.27		71.46	50.88	20.58	
	2	75.67	58.06	17.61	19.19	59.42	79.44	-20.02	-20.42	90.47	67.04	23.43	25.92	81.60	88.17	-6.57	-4.57	68.27	57.55	10.72	19.58
	3	75.68	58.24	17.44		58.61	81.66	-23.05		92.37	66.70	25.67		86.35	88.22	-1.87		79.92	52.49	27.43	
4	1	77.80	92.94	-15.14		80.36	85.83	-5.47		94.62	75.12	19.50		101.60	62.55	39.05		75.99	88.38	-12.39	
	2	77.18	94.10	-16.92	-12.73	78.85	83.90	-5.05	-5.26	97.22	71.66	25.56	23.52	102.06	63.17	38.89	40.26	71.46	87.52	-16.06	-14.08
	3	77.96	84.10	-6.14		78.04	83.29	-5.25		95.75	70.24	25.51		106.55	63.72	42.83		73.18	86.97	-13.79	
5	1	75.31	93.14	-17.83		80.71	84.90	-4.19		86.43	61.46	24.97		86.43	73.24	13.19		83.21	67.02	16.19	
	2	61.88	94.49	-32.61	-27.96	67.18	86.11	-18.93	-9.33	84.64	66.51	18.13	20.03	84.46	76.83	7.63	9.57	86.80	61.99	24.81	19.01
	3	66.14	99.57	-33.43		81.14	86.02	-4.88		83.93	66.95	16.98		84.87	76.99	7.88		80.00	63.97	16.03	
6	1	86.30	60.84	25.46		71.18	82.97	-11.79		70.96	61.22	9.74		66.73	62.31	4.42		101.45	69.30	32.15	
	2	79.27	67.57	11.70	20.34	60.13	83.90	-23.77	-12.58	70.72	66.03	4.69	8.41	75.75	70.17	5.58	4.10	85.66	71.41	14.25	19.74
	3	83.69	59.82	23.87		74.97	77.16	-2.19		76.10	65.30	10.80		70.64	68.34	2.30		83.07	70.24	12.83	
7	1	67.85	75.37	-7.52		84.15	60.80	23.35		81.17	70.25	10.92		69.93	53.33	16.60		55.85	51.06	4.79	
	2	67.15	77.28	-10.13	-7.19	73.87	56.33	17.54	20.43	82.72	71.34	11.38	11.78	70.11	57.53	12.58	15.76	54.65	50.68	3.97	4.50
	3	65.05	68.97	-3.92		74.81	54.40	20.41		84.38	71.34	13.04		77.71	59.62	18.09		56.07	51.33	4.74	
8	1	79.98	54.48	25.50		87.39	92.69	-5.30		38.82	57.50	-18.68		81.75	84.77	-3.02		66.55	74.12	-7.57	
	2	81.50	55.48	26.02	24.98	79.63	84.42	-4.79	-4.99	40.81	56.79	-15.98	-16.72	62.64	70.57	-7.93	-8.36	53.05	83.75	-30.70	-20.80
	3	83.54	60.12	23.42		80.44	85.32	-4.88		41.86	57.36	-15.50		60.43	74.56	-14.13		61.96	86.10	-24.14	
Mean		75.10	70.64	4.46	4.46	75.49	77.74	-2.25	-2.25	78.30	72.78	5.51	5.51	77.77	72.82	4.94	4.94	72.75	76.34	-3.59	-3.59
SE		1.42	2.97	3.78	6.60	1.88	2.71	3.70	6.53	3.33	2.71	3.57	6.42	2.52	2.03	3.37	6.03	2.33	3.90	4.41	7.68

Group 7 Cmpd C1 (100 µg/kg) ip, QD	Trial	Force (g)		Paw Wt Distribution (g)		Force (g)		Paw Wt Distribution (g)		Force (g)		Paw Wt Distribution (g)		Force (g)		Paw Wt Distribution (g)		Force (g)		Paw Wt Distribution (g)	
	#	Left	Right	Per Trial	Mean	Left	Right	Per Trial	Mean	Left	Right	Per Trial	Mean	Left	Right	Per Trial	Mean	Left	Right	Per Trial	Mean
**Animal 1**	1	92.87	80.30	12.57		83.35	93.05	-9.70		78.77	98.50	-19.73		66.88	89.80	-22.92		89.54	80.80	8.74	
	2	90.99	84.17	6.82	**8.79**	82.70	92.57	-9.87	**-9.94**	77.34	96.06	-18.72	**-18.78**	67.28	85.63	-18.35	**-19.55**	90.33	85.05	5.28	**7.84**
	3	93.67	86.69	6.98		82.19	92.45	-10.26		77.38	95.27	-17.89		69.75	87.13	-17.38		93.66	84.17	9.49	
**2**	1	97.69	72.86	24.83		87.95	65.68	22.27		74.49	83.66	-9.17		76.21	93.71	-17.50		80.54	70.07	10.47	
	2	91.57	72.41	19.16	**26.34**	86.93	65.49	21.44	**21.51**	76.53	84.87	-8.34	**-8.54**	76.22	93.80	-17.58	**-17.75**	82.79	69.38	13.41	**12.73**
	3	93.48	58.45	35.03		86.25	65.43	20.82		75.29	83.40	-8.11		75.40	93.58	-18.18		85.14	70.82	14.32	
**3**	1	85.85	76.72	9.13		68.07	86.76	-18.69		91.06	81.80	9.26		85.78	105.71	-19.93		113.30	101.90	11.40	
	2	90.76	81.56	9.20	**8.95**	67.77	85.94	-18.17	**-17.88**	90.91	79.92	10.99	**7.93**	83.53	102.91	-19.38	**-17.85**	118.03	102.66	15.37	**14.31**
	3	90.96	82.44	8.52		67.25	84.02	-16.77		88.31	84.78	3.53		79.63	93.86	-14.23		118.94	102.77	16.17	
**4**	1	88.44	66.32	22.12		77.36	56.17	21.19		81.88	102.17	-20.29		76.62	90.75	-14.13		81.02	68.13	12.89	
	2	78.09	76.86	1.23	**13.68**	73.00	58.37	14.63	**16.60**	79.17	102.37	-23.20	**-22.16**	77.68	91.80	-14.12	**-13.62**	94.56	63.40	31.16	**22.56**
	3	81.37	63.67	17.70		72.83	58.84	13.99		79.62	102.60	-22.98		76.69	89.30	-12.61		91.11	67.47	23.64	
**5**	1	86.91	75.35	11.56		78.54	67.63	10.91		120.73	61.33	59.40		105.57	66.24	39.33		110.98	66.78	44.20	
	2	85.17	56.97	28.20	**24.40**	77.22	66.00	11.22	**10.86**	118.62	96.91	21.71	**34.39**	102.97	67.17	35.80	**36.99**	112.55	66.85	45.70	**44.28**
	3	89.38	55.95	33.43		77.30	66.84	10.46		115.30	93.23	22.07		103.27	67.43	35.84		110.17	67.23	42.94	
**6**	1	66.45	77.25	-10.80		66.72	77.53	-10.81		90.14	78.71	11.43		108.53	73.39	35.14		56.97	71.85	-14.88	
	2	68.11	79.68	-11.57	**-20.05**	65.26	77.10	-11.84	**-11.76**	91.24	79.54	11.70	**10.28**	109.50	73.71	35.79	**35.48**	63.81	90.70	-26.89	**-14.29**
	3	62.92	100.70	-37.78		65.05	77.68	-12.63		90.08	82.38	7.70		109.38	73.87	35.51		69.38	70.49	-1.11	
**7**	1	87.46	76.00	11.46		107.67	92.14	15.53		86.46	103.98	-17.52		114.10	59.88	54.22		90.47	69.52	20.95	
	2	83.69	69.36	14.33	**10.71**	105.54	87.81	17.73	**17.88**	84.71	99.78	-15.07	**-16.52**	111.00	62.09	48.91	**51.12**	95.30	83.32	11.98	**19.49**
	3	84.71	78.38	6.33		105.02	84.63	20.39		84.30	101.27	-16.97		115.58	65.36	50.22		100.85	75.32	25.53	
**8**	1	82.42	68.15	14.27		84.79	81.13	3.66		81.66	86.30	-4.64		58.22	85.18	-26.96		76.12	69.42	6.70	
	2	77.41	69.48	7.93	**11.52**	81.45	78.60	2.85	**3.26**	80.92	85.81	-4.89	**-4.77**	59.68	89.51	-29.83	**-29.92**	77.58	75.41	2.17	**6.20**
	3	85.88	73.53	12.35		81.97	78.71	3.26		81.65	86.42	-4.77		59.06	92.03	-32.97		83.53	73.79	9.74	
**Mean**		**84.84**	**74.30**	**10.54**	**10.54**	**80.51**	**76.69**	**3.82**	**3.82**	**87.36**	**89.63**	**-2.27**	**-2.27**	**86.19**	**83.08**	**3.11**	**3.11**	**91.11**	**76.97**	**14.14**	**14.14**
**SE**		**1.81**	**2.05**	**3.12**	**4.99**	**2.50**	**2.41**	**2.99**	**5.39**	**2.66**	**2.14**	**3.94**	**6.71**	**3.95**	**2.71**	**6.29**	**11.39**	**3.43**	**2.45**	**3.41**	**5.86**

Group 8 Cmpd D1 (10 µg/kg) ip, QD	Trial	Force (g)		Paw Wt Distribution (g)		Force (g)		Paw Wt Distribution (g)		Force (g)		Paw Wt Distribution (g)		Force (g)		Paw Wt Distribution (g)		Force (g)		Paw Wt Distribution (g)	
	#	Left	Right	Per Trial	Mean	Left	Right	Per Trial	Mean	Left	Right	Per Trial	Mean	Left	Right	Per Trial	Mean	Left	Right	Per Trial	Mean
**Animal 1**	1	97.56	87.32	10.24		93.89	82.74	11.15		72.69	82.52	-9.83		90.37	100.17	-9.80		89.00	90.00	-1.00	
	2	97.30	85.53	11.77	**11.20**	95.23	81.64	13.59	**12.44**	73.68	82.92	-9.24	**-9.21**	87.56	99.57	-12.01	**-12.08**	87.84	90.45	-2.61	**-5.29**
	3	99.76	88.18	11.58		94.56	81.99	12.57		75.20	83.75	-8.55		84.32	98.75	-14.43		81.93	94.19	-12.26	
**2**	1	82.33	60.26	22.07		64.32	109.15	-44.83		93.99	80.32	13.67		105.23	95.80	9.43		96.69	65.10	31.59	
	2	93.82	57.71	36.11	**32.48**	62.37	101.08	-38.71	**-41.75**	93.32	79.84	13.48	**10.43**	95.80	85.56	10.24	**12.87**	102.38	65.14	37.24	**33.85**
	3	97.86	58.59	39.27		61.19	102.89	-41.70		86.76	82.61	4.15		97.01	78.08	18.93		94.44	61.71	32.73	
**3**	1	96.15	74.85	21.30		102.77	100.39	2.38		61.64	74.45	-12.81		93.09	89.52	3.57		110.02	84.01	26.01	
	2	94.97	77.08	17.89	**18.59**	102.98	100.47	2.51	**2.28**	59.07	87.57	-28.50	**-21.78**	94.54	87.85	6.69	**5.48**	108.83	83.57	25.26	**24.00**
	3	94.94	78.36	16.58		101.42	99.47	1.95		62.97	87.01	-24.04		94.54	88.35	6.19		107.32	86.59	20.73	
**4**	1	105.48	70.17	35.31		83.91	82.75	1.16		96.41	77.29	19.12		89.20	69.21	19.99		110.71	78.98	31.73	
	2	104.22	68.35	35.87	**35.10**	83.20	86.70	-3.50	**-2.23**	101.77	78.94	22.83	**21.85**	92.95	71.35	21.60	**20.97**	110.93	79.56	31.37	**31.62**
	3	103.24	69.13	34.11		83.25	87.60	-4.35		101.71	78.12	23.59		92.59	71.26	21.33		111.81	80.04	31.77	
**5**	1	93.42	64.87	28.55		85.00	87.19	-2.19		72.37	96.09	-23.72		88.50	72.72	15.78		76.93	71.63	5.30	
	2	94.65	68.16	26.49	**26.84**	86.51	88.64	-2.13	**-1.68**	87.34	94.47	-7.13	**-11.71**	102.40	74.98	27.42	**28.02**	84.27	76.84	7.43	**8.00**
	3	90.25	64.78	25.47		87.71	88.43	-0.72		94.31	98.59	-4.28		109.87	69.00	40.87		92.06	80.79	11.27	
**6**	1	92.23	87.42	4.81		116.09	74.84	41.25		98.54	63.39	35.15		110.80	71.69	39.11		77.92	83.96	-6.04	
	2	94.01	88.48	5.53	**5.34**	114.92	76.09	38.83	**39.61**	97.70	63.26	34.44	**34.08**	111.81	71.39	40.42	**40.30**	79.11	84.14	-5.03	**-5.11**
	3	94.32	88.65	5.67		113.79	75.03	38.76		97.97	65.33	32.64		112.30	70.93	41.37		86.32	90.57	-4.25	
**7**	1	71.50	80.56	-9.06		75.68	102.72	-27.04		90.80	60.72	30.08		70.64	89.98	-19.34		73.31	89.33	-16.02	
	2	73.23	82.48	-9.25	**-11.93**	74.57	100.50	-25.93	**-25.06**	91.64	62.65	28.99	**29.96**	69.82	88.52	-18.70	**-21.18**	73.40	87.97	-14.57	**-15.13**
	3	71.05	88.53	-17.48		78.48	100.70	-22.22		93.52	62.70	30.82		70.63	96.13	-25.50		75.33	90.12	-14.79	
**8**	1	86.81	65.30	21.51		83.13	89.85	-6.72		85.98	80.06	5.92		92.53	59.18	33.35		93.10	61.74	31.36	
	2	79.43	73.51	5.92	**16.15**	84.12	90.37	-6.25	**-11.76**	98.09	79.81	18.28	**12.77**	94.07	61.96	32.11	**33.11**	95.61	57.55	38.06	**35.51**
	3	89.42	68.39	21.03		71.94	94.25	-22.31		98.57	84.45	14.12		95.21	61.35	33.86		95.46	58.35	37.11	
**Mean**		**91.58**	**74.86**	**16.72**	**16.72**	**87.54**	**91.06**	**-3.52**	**-3.52**	**86.92**	**78.62**	**8.30**	**8.30**	**93.57**	**80.14**	**13.44**	**13.44**	**92.28**	**78.85**	**13.43**	**13.43**
**SE**		**1.98**	**2.14**	**3.13**	**5.47**	**3.20**	**2.02**	**4.79**	**8.61**	**2.73**	**2.22**	**4.12**	**7.26**	**2.44**	**2.65**	**4.32**	**7.67**	**2.65**	**2.34**	**4.00**	**7.18**

Group 9 Cmpd E1 (50 mg/kg) po, QD	Trial	Force (g)		Paw Wt Distribution (g)		Force (g)		Paw Wt Distribution (g)		Force (g)		Paw Wt Distribution (g)		Force (g)		Paw Wt Distribution (g)		Force (g)		Paw Wt Distribution (g)	
	#	Left	Right	Per Trial	Mean	Left	Right	Per Trial	Mean	Left	Right	Per Trial	Mean	Left	Right	Per Trial	Mean	Left	Right	Per Trial	Mean
**Animal 1**	1	82.05	115.01	-32.96		77.67	94.52	-16.85		70.75	112.65	-41.90		97.94	95.87	2.07		70.51	114.89	-44.38	
	2	73.87	109.37	-35.50	**-33.81**	80.40	93.54	-13.14	**-15.59**	72.65	113.31	-40.66	**-40.17**	100.97	92.87	8.10	**7.00**	69.53	117.07	-47.54	**-46.93**
	3	74.52	107.48	-32.96		77.96	94.74	-16.78		76.17	114.13	-37.96		103.56	92.74	10.82		69.86	118.74	-48.88	
**2**	1	95.80	76.00	19.80		118.31	67.49	50.82		92.58	89.42	3.16		73.42	99.41	-25.99		96.67	88.75	7.92	
	2	92.78	77.63	15.15	**16.21**	113.73	69.21	44.52	**46.38**	100.35	85.02	15.33	**9.30**	77.27	101.11	-23.84	**-25.55**	102.74	93.46	9.28	**8.52**
	3	92.19	78.51	13.68		111.00	67.20	43.80		97.43	88.01	9.42		76.73	103.55	-26.82		98.22	89.85	8.37	
**3**	1	85.79	66.99	18.80		84.47	76.84	7.63		89.72	74.35	15.37		60.69	88.95	-28.26		112.29	62.40	49.89	
	2	90.31	67.90	22.41	**21.18**	95.50	59.53	35.97	**18.21**	91.54	86.58	4.96	**24.23**	77.44	81.67	-4.23	**-12.01**	105.88	65.05	40.83	**45.12**
	3	90.99	68.67	22.32		81.17	70.14	11.03		100.36	48.01	52.35		81.78	85.33	-3.55		111.50	66.87	44.63	
**4**	1	84.45	80.34	4.11		87.10	78.79	8.31		83.96	69.33	14.63		78.92	66.36	12.56		94.14	68.04	26.10	
	2	85.48	80.37	5.11	**4.32**	86.64	75.37	11.27	**9.81**	88.00	74.44	13.56	**14.00**	75.54	64.99	10.55	**11.57**	89.39	74.73	14.66	**21.10**
	3	84.53	80.79	3.74		77.63	67.79	9.84		89.29	75.48	13.81		76.32	64.72	11.60		93.76	71.22	22.54	
**5**	1	100.79	67.60	33.19		91.59	65.68	25.91		97.72	63.60	34.12		105.54	53.77	51.77		104.73	64.30	40.43	
	2	105.16	67.25	37.91	**40.24**	85.01	69.17	15.84	**19.93**	97.06	64.81	32.25	**36.24**	112.08	54.90	57.18	**53.73**	103.51	68.80	34.71	**34.01**
	3	110.87	61.24	49.63		86.08	68.04	18.04		106.63	64.27	42.36		111.61	59.36	52.25		99.80	72.92	26.88	
**6**	1	80.15	70.30	9.85		76.01	96.53	-20.52		87.11	90.79	-3.68		71.76	70.36	1.40		86.53	79.74	6.79	
	2	83.83	71.38	12.45	**14.44**	55.93	94.09	-38.16	**-30.69**	92.72	81.79	10.93	**6.95**	65.27	71.08	-5.81	**-4.57**	82.86	82.39	0.47	**3.27**
	3	92.34	71.31	21.03		60.27	93.66	-33.39		94.09	80.48	13.61		64.95	74.25	-9.30		81.88	79.33	2.55	
**7**	1	66.07	102.55	-36.48		91.08	85.04	6.04		70.05	85.95	-15.90		60.05	100.91	-40.86		75.52	81.76	-6.24	
	2	65.42	93.30	-27.88	**-32.00**	92.56	86.68	5.88	**3.30**	72.46	86.33	-13.87	**-15.36**	89.06	114.49	-25.43	**-31.97**	78.79	83.35	-4.56	**-3.83**
	3	62.12	93.77	-31.65		91.94	93.97	-2.03		73.12	89.43	-16.31		74.36	103.99	-29.63		78.27	78.95	-0.68	
**8**	1	71.60	83.71	-12.11		74.13	87.65	-13.52		70.08	103.34	-33.26		71.42	80.73	-9.31		63.74	101.33	-37.59	
	2	70.21	83.37	-13.16	**-19.47**	76.13	87.09	-10.96	**-13.21**	70.95	110.55	-39.60	**-37.69**	96.98	95.33	1.65	**-11.17**	67.90	101.62	-33.72	**-38.14**
	3	60.84	93.98	-33.14		77.80	92.96	-15.16		72.35	112.56	-40.21		77.74	103.59	-25.85		55.90	99.02	-43.12	
**Mean**		**83.42**	**82.03**	**1.39**	**1.39**	**85.42**	**80.66**	**4.77**	**4.77**	**85.71**	**86.03**	**-0.31**	**-0.31**	**82.56**	**84.18**	**-1.62**	**-1.62**	**87.25**	**84.36**	**2.89**	**2.89**
**SE**		**2.73**	**3.08**	**5.36**	**9.53**	**2.98**	**2.48**	**4.91**	**8.62**	**2.42**	**3.68**	**5.72**	**9.91**	**3.22**	**3.62**	**5.40**	**9.46**	**3.29**	**3.48**	**6.35**	**11.44**

Group 10 Cmpd F1 (50 mg/kg) po, QD	Trial	Force (g)		Paw Wt Distribution (g)		Force (g)		Paw Wt Distribution (g)		Force (g)		Paw Wt Distribution (g)		Force (g)		Paw Wt Distribution (g)		Force (g)		Paw Wt Distribution (g)	
	#	Left	Right	Per Trial	Mean	Left	Right	Per Trial	Mean	Left	Right	Per Trial	Mean	Left	Right	Per Trial	Mean	Left	Right	Per Trial	Mean
Animal 1	1	88.49	94.01	-5.52		95.27	80.24	15.03		84.82	98.20	-13.38		115.59	63.81	51.78		94.08	79.75	14.33	
	2	89.51	93.67	-4.16	**-6.36**	94.64	85.62	9.02	**9.59**	90.64	94.40	-3.76	**-9.76**	114.41	61.80	52.61	**47.95**	91.65	83.55	8.10	**10.17**
	3	89.03	98.44	-9.41		93.17	88.44	4.73		85.92	98.07	-12.15		114.13	74.67	39.46		91.95	83.86	8.09	
2	1	106.15	75.73	30.42		103.50	76.12	27.38		111.14	88.13	23.01		99.72	85.80	13.92		97.01	73.02	23.99	
	2	110.36	74.66	35.70	**32.94**	104.04	75.04	29.00	**32.23**	114.45	78.03	36.42	**28.80**	90.63	78.50	12.13	**12.45**	89.09	75.68	13.41	**20.87**
	3	110.40	77.71	32.69		107.65	67.35	40.30		105.61	78.64	26.97		94.99	83.68	11.31		93.96	68.74	25.22	
3	1	84.70	74.15	10.55		114.05	75.82	38.23		75.63	88.21	-12.58		95.89	77.12	18.77		104.16	87.48	16.68	
	2	88.16	73.28	14.88	**14.33**	113.13	77.99	35.14	**33.91**	73.15	91.02	-17.87	**-15.43**	104.47	78.38	26.09	**24.39**	105.40	86.12	19.28	**19.52**
	3	91.24	73.69	17.55		108.51	80.14	28.37		72.48	88.33	-15.85		106.42	78.11	28.31		106.05	83.44	22.61	
4	1	104.18	75.16	29.02		84.79	98.24	-13.45		101.26	76.02	25.24		88.49	97.25	-8.76		85.91	104.65	-18.74	
	2	110.76	68.12	42.64	**41.31**	84.72	98.33	-13.61	**-13.70**	93.16	82.62	10.54	**14.40**	87.17	96.87	-9.70	**-9.67**	90.11	97.10	-6.99	**-15.42**
	3	117.03	64.77	52.26		85.56	99.60	-14.04		95.25	87.84	7.41		87.89	98.45	-10.56		85.32	105.85	-20.53	
5	1	89.40	79.48	9.92		102.72	62.88	39.84		79.52	72.23	7.29		92.49	80.79	11.70		90.02	67.19	22.83	
	2	91.82	73.86	17.96	**14.91**	106.38	67.38	39.00	**38.08**	84.92	72.26	12.66	**11.52**	89.14	76.57	12.57	**9.03**	79.91	68.51	11.40	**14.74**
	3	93.09	76.23	16.86		101.61	66.22	35.39		89.00	74.39	14.61		90.23	87.42	2.81		89.58	79.58	10.00	
6	1	112.23	87.64	24.59		99.99	90.98	9.01		98.21	89.60	8.61		86.18	75.01	11.17		97.04	78.68	18.36	
	2	103.01	66.96	36.05	**29.42**	94.04	84.24	9.80	**11.45**	99.12	90.12	9.00	**8.30**	96.77	68.82	27.95	**22.43**	96.59	77.37	19.22	**19.45**
	3	101.89	74.26	27.63		99.96	84.42	15.54		99.55	92.27	7.28		100.79	72.61	28.18		99.21	78.44	20.77	
7	1	81.06	101.06	-20.00		80.03	77.35	2.68		85.60	82.36	3.24		93.52	91.27	2.25		75.48	75.04	0.44	
	2	83.48	95.73	-12.25	**-15.31**	70.99	68.12	2.87	**3.86**	86.04	74.64	11.40	**5.06**	94.96	71.05	23.91	**20.62**	71.43	74.24	-2.81	**-2.22**
	3	86.16	99.85	-13.69		80.43	74.39	6.04		80.17	79.63	0.54		101.51	65.81	35.70		72.20	76.48	-4.28	
8	1	73.70	86.91	-13.21		67.96	70.32	-2.36		92.76	89.65	3.11		119.90	70.02	49.88		78.54	67.33	11.21	
	2	75.01	85.02	-10.01	**-10.97**	76.27	77.39	-1.12	**-4.27**	95.24	94.16	1.08	**3.53**	113.02	64.57	48.45	**35.98**	77.84	67.54	10.30	**7.85**
	3	75.85	85.53	-9.68		75.02	84.34	-9.32		93.34	86.93	6.41		90.16	80.55	9.61		78.68	76.65	2.03	
**Mean**		**94.03**	**81.50**	**12.53**	**12.53**	**93.52**	**79.62**	**13.89**	**13.89**	**91.12**	**85.32**	**5.80**	**5.80**	**98.69**	**78.29**	**20.40**	**20.40**	**89.22**	**79.85**	**9.37**	**9.37**
**SE**		**2.61**	**2.23**	**4.29**	**7.59**	**2.76**	**2.12**	**3.78**	**6.73**	**2.26**	**1.63**	**2.85**	**4.89**	**2.11**	**2.15**	**3.90**	**6.15**	**2.07**	**2.17**	**2.61**	**4.47**

Table 73. Individual animal data for MMT/MCLT Trial 1 Day 21 data

		Day 21 (Pre tx)				Day 21 (1 hr)				Day 21 (3 hr)				Day 21 (5 hr)				Day 21 (24 hr)			
Group 1 Vehicle (Saline) ip, QD	Trial	Force (g)		Paw Wt Distribution (g)		Force (g)		Paw Wt Distribution (g)		Force (g)		Paw Wt Distribution (g)		Force (g)		Paw Wt Distribution (g)		Force (g)		Paw Wt Distribution (g)	
	#	Left	Right	Per Trial	Mean	Left	Right	Per Trial	Mean	Left	Right	Per Trial	Mean	Left	Right	Per Trial	Mean	Left	Right	Per Trial	Mean
Animal 1	1	106.96	77.99	28.97		110.33	88.22	22.11		98.06	89.03	9.03		99.07	84.37	14.70		107.94	107.74	0.20	
	2	94.76	86.41	8.35	**12.95**	112.98	86.57	26.41	**22.98**	103.68	86.78	16.90	**13.90**	96.05	88.71	7.34	**9.13**	90.17	89.11	1.06	**1.53**
	3	98.37	96.83	1.54		109.22	88.81	20.41		112.70	96.94	15.76		96.02	90.67	5.35		91.79	88.47	3.32	
2	1	115.38	85.60	29.78		105.59	86.24	19.35		105.48	74.49	30.99		77.80	85.94	-8.14		97.22	83.75	13.47	
	2	122.94	84.22	38.72	**28.79**	104.39	89.41	14.98	**16.83**	98.20	92.13	6.07	**20.92**	76.36	85.36	-9.00	**-11.45**	100.44	84.49	15.95	**10.85**
	3	106.31	88.45	17.86		106.45	90.29	16.16		105.45	79.74	25.71		85.17	102.38	-17.21		96.34	93.21	3.13	
3	1	93.80	82.33	11.47		94.83	87.80	7.03		85.61	97.16	-11.55		94.35	104.27	-9.92		93.89	71.61	22.28	
	2	101.92	84.59	17.33	**10.63**	102.20	80.85	21.35	**11.87**	90.80	104.50	-13.70	**-14.50**	101.40	96.90	4.50	**-3.71**	111.10	68.99	42.11	**32.91**
	3	98.26	95.18	3.08		98.30	91.08	7.22		86.69	104.94	-18.25		88.97	94.67	-5.70		104.51	70.17	34.34	
4	1	104.56	108.72	-4.16		99.76	90.57	9.19		112.77	75.36	37.41		133.73	83.66	50.07		96.30	95.43	0.87	
	2	118.26	111.08	7.18	**2.29**	103.08	90.57	12.51	**13.11**	110.88	73.50	37.38	**37.99**	136.00	79.46	56.54	**53.76**	98.11	93.09	5.02	**8.13**
	3	117.29	113.43	3.86		107.62	89.99	17.63		112.35	73.18	39.17		133.60	78.92	54.68		106.76	88.26	18.50	
5	1	110.15	85.96	24.19		104.09	92.40	11.69		87.43	79.05	8.38		83.22	83.38	-0.16		107.62	91.39	16.23	
	2	108.36	85.79	22.57	**22.78**	104.70	94.73	9.97	**10.43**	91.49	82.04	9.45	**8.01**	80.97	84.29	-3.32	**-4.30**	107.07	92.75	14.32	**14.57**
	3	107.63	86.06	21.57		107.93	98.30	9.63		91.36	85.15	6.21		79.79	89.21	-9.42		107.03	93.88	13.15	
6	1	94.48	70.94	23.54		90.62	74.13	16.49		104.46	74.66	29.80		95.69	55.54	40.15		85.28	80.56	4.72	
	2	100.00	57.74	42.26	**38.33**	93.02	80.72	12.30	**14.39**	105.40	76.38	29.02	**28.92**	104.57	60.00	44.57	**44.47**	94.08	82.54	11.54	**5.52**
	3	105.32	56.14	49.18		90.68	76.29	14.39		104.52	76.59	27.93		107.89	59.20	48.69		93.77	93.48	0.29	
7	1	103.30	89.47	13.83		122.85	78.07	44.78		111.87	77.56	34.31		91.25	70.37	20.88		105.17	88.59	16.58	
	2	102.77	88.03	14.74	**14.23**	123.64	78.08	45.56	**44.92**	110.29	84.32	25.97	**27.73**	80.34	74.90	5.44	**13.06**	103.76	90.63	13.13	**13.50**
	3	102.53	88.40	14.13		123.66	79.25	44.41		108.10	85.20	22.90		87.09	74.23	12.86		105.37	94.59	10.78	
8	1	94.78	83.31	11.47		114.70	87.35	27.35		101.31	76.88	24.43		105.97	80.97	25.00		104.19	67.89	36.30	
	2	93.13	84.20	8.93	**12.44**	111.83	94.13	17.70	**21.29**	99.79	78.56	21.23	**22.53**	105.73	80.25	25.48	**25.38**	102.49	70.59	31.90	**32.87**
	3	96.84	79.92	16.92		111.77	92.94	18.83		100.50	78.57	21.93		105.11	79.44	25.67		101.55	71.15	30.40	
**Mean**		**104.09**	**86.28**	**17.80**	**17.80**	**106.43**	**86.95**	**19.48**	**19.48**	**101.63**	**83.45**	**18.19**	**18.19**	**97.76**	**81.96**	**15.79**	**15.79**	**100.50**	**85.52**	**14.98**	**14.98**
**SE**		**1.69**	**2.75**	**2.68**	**4.06**	**1.91**	**1.31**	**2.29**	**3.95**	**1.79**	**1.94**	**3.28**	**5.69**	**3.50**	**2.51**	**4.71**	**8.38**	**1.37**	**2.15**	**2.52**	**4.18**

Group 2 Morphine (10 mg/kg) ip, QD	Trial	Force (g)		Paw Wt Distribution (g)		Force (g)		Paw Wt Distribution (g)		Force (g)		Paw Wt Distribution (g)		Force (g)		Paw Wt Distribution (g)		Force (g)		Paw Wt Distribution (g)	
	#	Left	Right	Per Trial	Mean	Left	Right	Per Trial	Mean	Left	Right	Per Trial	Mean	Left	Right	Per Trial	Mean	Left	Right	Per Trial	Mean
Animal 1	1	108.54	99.35	9.19		113.77	106.30	7.47		93.79	97.46	-3.67		90.07	110.99	-20.92		79.81	73.86	5.95	
	2	110.28	102.92	7.36	**8.90**	116.57	104.74	11.83	**9.97**	88.74	101.35	-12.61	**-7.30**	90.40	110.20	-19.80	**-20.71**	81.99	84.86	-2.87	**0.38**
	3	108.27	98.11	10.16		116.60	105.98	10.62		97.57	103.18	-5.61		89.17	110.58	-21.41		86.10	88.05	-1.95	
2	1	92.31	86.41	5.90		90.91	100.72	-9.81		73.64	88.10	-14.46		86.67	91.04	-4.37		88.28	101.85	-13.57	
	2	95.41	87.37	8.04	**10.86**	83.55	111.97	-28.42	**-16.89**	71.33	97.39	-26.06	**-21.00**	78.74	94.45	-15.71	**-7.48**	102.25	103.49	-1.24	**-8.98**
	3	103.43	84.79	18.64		92.28	104.72	-12.44		74.39	96.87	-22.48		84.15	86.51	-2.36		95.48	107.62	-12.14	
3	1	101.91	91.52	10.39		75.89	98.71	-22.82		91.53	101.58	-10.05		100.84	104.70	-3.86		103.74	80.30	23.44	
	2	87.01	86.04	0.97	**5.05**	70.79	115.63	-44.84	**-32.78**	92.05	102.05	-10.00	**-8.25**	99.35	100.32	-0.97	**-2.43**	103.81	81.56	22.25	**19.89**
	3	117.24	113.45	3.79		78.16	108.85	-30.69		96.57	101.28	-4.71		95.90	98.37	-2.47		104.11	90.12	13.99	
4	1	88.67	71.96	16.71		96.48	45.41	51.07		110.22	67.55	42.67		79.40	104.60	-25.20		104.35	89.35	15.00	
	2	89.81	79.37	10.44	**13.27**	89.51	49.61	39.90	**46.32**	109.83	68.96	40.87	**41.52**	71.14	105.26	-34.12	**-28.01**	94.61	88.11	6.50	**14.84**
	3	101.49	88.84	12.65		90.34	42.36	47.98		108.43	67.41	41.02		76.48	101.20	-24.72		104.18	81.16	23.02	
5	1	87.48	72.56	14.92		88.58	73.79	14.79		101.16	76.73	24.43		74.56	89.61	-15.05		91.98	84.92	7.06	
	2	104.33	86.86	17.47	**14.82**	96.20	68.13	28.07	**21.76**	100.22	79.49	20.73	**19.49**	78.41	86.01	-7.60	**-10.80**	92.42	84.08	8.34	**8.13**
	3	102.26	90.19	12.07		92.94	70.52	22.42		98.37	85.05	13.32		80.76	90.51	-9.75		94.22	85.24	8.98	
6	1	105.70	76.31	29.39		128.36	111.60	16.76		108.34	72.64	35.70		106.81	97.56	9.25		117.15	91.12	26.03	
	2	106.14	75.63	30.51	**30.41**	120.02	108.72	11.30	**20.28**	115.97	76.31	39.66	**37.91**	107.82	97.14	10.68	**10.75**	118.17	89.07	29.10	**28.38**
	3	106.56	75.24	31.32		128.62	95.84	32.78		114.62	76.26	38.36		108.78	96.46	12.32		119.50	89.50	30.00	
7	1	93.73	69.48	24.25		102.05	74.99	27.06		103.21	83.94	19.27		114.18	67.98	46.20		106.23	71.64	34.59	
	2	98.10	67.51	30.59	**31.04**	111.48	68.90	42.58	**31.87**	112.28	90.97	21.31	**19.82**	119.15	65.17	53.98	**50.92**	116.62	69.37	47.25	**39.80**
	3	107.52	69.24	38.28		97.60	71.63	25.97		104.76	85.87	18.89		118.33	65.75	52.58		109.70	72.14	37.56	
8	1	85.72	91.95	-6.23		92.74	113.61	-20.87		75.88	103.14	-27.26		91.05	93.12	-2.07		93.75	81.65	12.10	
	2	82.63	93.31	-10.68	**-9.53**	97.62	117.40	-19.78	**-19.73**	72.85	105.46	-32.61	**-31.15**	90.88	94.57	-3.69	**-2.88**	91.30	82.14	9.16	**8.73**
	3	81.47	93.16	-11.69		101.05	119.60	-18.55		73.18	106.77	-33.59		92.00	94.89	-2.89		86.96	82.03	4.93	
**Mean**		**98.58**	**85.48**	**13.10**	**13.10**	**98.84**	**91.24**	**7.60**	**7.60**	**95.37**	**88.99**	**6.38**	**6.38**	**92.71**	**94.04**	**-1.33**	**-1.33**	**99.45**	**85.55**	**13.90**	**13.90**
**SE**		**2.01**	**2.41**	**2.68**	**4.68**	**3.20**	**4.97**	**5.60**	**9.85**	**3.01**	**2.71**	**5.33**	**9.58**	**2.87**	**2.62**	**4.76**	**8.55**	**2.35**	**1.93**	**3.16**	**5.48**

Group 3 Cmpd A1 (0.3 mg/kg ip, QD)	Trial	Force (g)		Paw Wt Distribution (g)		Force (g)		Paw Wt Distribution (g)		Force (g)		Paw Wt Distribution (g)		Force (g)		Paw Wt Distribution (g)		Force (g)		Paw Wt Distribution (g)	
	#	Left	Right	Per Trial	Mean	Left	Right	Per Trial	Mean	Left	Right	Per Trial	Mean	Left	Right	Per Trial	Mean	Left	Right	Per Trial	Mean
Animal 1	1	90.34	73.43	16.91		80.43	78.72	1.71		88.20	64.01	24.19		95.99	67.40	28.59		103.14	52.06	51.08	
	2	85.33	74.81	10.52	**12.73**	85.61	77.16	8.45	**5.27**	68.59	61.82	6.77	**25.46**	99.96	45.09	54.87	**47.50**	101.71	48.29	53.42	**48.47**
	3	86.37	75.61	10.76		84.91	79.27	5.64		95.88	50.45	45.43		98.39	39.35	59.04		100.11	59.20	40.91	
2	1	80.93	74.97	5.96		78.82	101.30	-22.48		70.06	100.30	-30.24		93.63	73.46	20.17		69.26	93.04	-23.78	
	2	83.35	75.76	7.59	**7.74**	79.59	93.71	-14.12	**-17.96**	73.43	96.32	-22.89	**-24.22**	103.04	75.99	27.05	**19.74**	69.70	92.10	-22.40	**-21.95**
	3	83.83	74.16	9.67		67.73	85.01	-17.28		77.35	96.89	-19.54		93.92	81.91	12.01		59.20	78.87	-19.67	
3	1	93.23	82.93	10.30		106.74	58.85	47.89		71.89	87.90	-16.01		96.83	84.44	12.39		69.59	74.81	-5.22	
	2	95.58	88.62	6.96	**8.17**	107.71	57.15	50.56	**49.00**	75.79	95.24	-19.45	**-15.12**	97.08	92.17	4.91	**7.21**	69.37	74.92	-5.55	**-5.19**
	3	92.68	85.43	7.25		110.84	62.30	48.54		82.18	92.09	-9.91		97.27	92.95	4.32		69.48	74.28	-4.80	
4	1	99.37	63.98	35.39		73.59	54.13	19.46		63.29	70.86	-7.57		90.51	85.35	5.16		69.39	102.30	-32.91	
	2	104.62	62.04	42.58	**26.16**	91.68	43.59	48.09	**31.02**	66.61	70.24	-3.63	**-5.24**	94.35	83.56	10.79	**8.34**	85.14	97.62	-12.48	**-18.89**
	3	80.04	79.53	0.51		80.41	54.91	25.50		76.48	80.99	-4.51		92.17	83.09	9.08		78.75	90.02	-11.27	
5	1	88.54	89.78	-1.24		92.52	79.17	13.35		84.14	97.07	-12.93		79.10	73.37	5.73		102.46	82.15	20.31	
	2	89.16	122.89	-33.73	**-20.63**	113.25	79.04	34.21	**21.91**	86.81	96.54	-9.73	**-9.77**	82.26	79.35	2.91	**5.10**	104.39	83.72	20.67	**19.72**
	3	89.05	115.98	-26.93		105.30	87.14	18.16		84.52	91.18	-6.66		75.28	68.61	6.67		106.82	88.63	18.19	
6	1	74.80	59.55	15.25		95.01	71.60	23.41		80.63	64.80	15.83		89.53	88.01	1.52		84.14	64.79	19.35	
	2	78.30	64.27	14.03	**14.10**	93.36	68.68	24.68	**23.89**	73.08	68.53	4.55	**8.79**	91.16	89.50	1.66	**0.74**	82.19	72.15	10.04	**12.84**
	3	81.86	68.84	13.02		92.22	68.65	23.57		76.50	70.52	5.98		88.29	89.25	-0.96		81.49	72.35	9.14	
7	1	97.01	72.82	24.19		82.03	93.87	-11.84		80.94	91.54	-10.60		87.44	83.89	3.55		116.02	70.56	45.46	
	2	99.15	72.07	27.08	**27.58**	82.27	97.43	-15.16	**-12.59**	66.45	110.26	-43.81	**-33.18**	87.42	78.73	8.69	**7.23**	117.15	70.55	46.60	**45.27**
	3	103.36	71.89	31.47		93.28	104.04	-10.76		67.73	112.85	-45.12		92.99	83.54	9.45		117.90	74.14	43.76	
8	1	56.19	84.30	-28.11		53.94	98.11	-44.17		92.32	79.47	12.85		60.39	56.98	3.41		73.29	64.51	8.78	
	2	62.71	78.86	-16.15	**-21.52**	53.14	101.79	-48.65	**-38.42**	92.18	77.23	14.95	**12.79**	65.30	59.81	5.49	**5.25**	73.84	65.28	8.56	**14.12**
	3	61.11	81.41	-20.30		64.67	87.10	-22.43		90.96	80.39	10.57		68.22	61.38	6.84		86.40	61.38	25.02	
**Mean**		**85.70**	**78.91**	**6.79**	**6.79**	**86.21**	**78.45**	**7.76**	**7.76**	**78.58**	**83.65**	**-5.06**	**-5.06**	**88.36**	**75.72**	**12.64**	**12.64**	**87.12**	**75.32**	**11.80**	**11.80**
**SE**		**2.58**	**3.03**	**4.06**	**6.62**	**3.34**	**3.52**	**5.85**	**10.28**	**1.91**	**3.30**	**4.24**	**6.97**	**2.29**	**2.94**	**3.17**	**5.34**	**3.66**	**2.84**	**5.27**	**9.36**

Group 4 Cmpd A2 (0.6 mg/kg ip, QD)	Trial	Force (g)		Paw Wt Distribution (g)		Force (g)		Paw Wt Distribution (g)		Force (g)		Paw Wt Distribution (g)		Force (g)		Paw Wt Distribution (g)		Force (g)		Paw Wt Distribution (g)	
	#	Left	Right	Per Trial	Mean	Left	Right	Per Trial	Mean	Left	Right	Per Trial	Mean	Left	Right	Per Trial	Mean	Left	Right	Per Trial	Mean
Animal 1	1	99.46	65.80	33.66		97.60	64.94	32.66		94.99	78.47	16.52		72.72	88.20	-15.48		67.67	94.04	-26.37	
	2	86.88	75.40	11.48	**19.45**	97.08	63.67	33.41	**34.99**	95.94	77.22	18.72	**14.65**	68.36	88.38	-20.02	**-20.16**	71.89	98.68	-26.79	**-27.54**
	3	86.34	73.12	13.22		102.25	63.35	38.90		85.18	76.48	8.70		67.35	92.33	-24.98		65.96	95.41	-29.45	
2	1	88.98	61.31	27.67		67.88	90.66	-22.78		82.81	69.01	13.80		86.73	71.49	15.24		63.62	98.22	-34.60	
	2	80.73	74.44	6.29	**12.94**	65.53	98.32	-32.79	**-29.28**	90.63	82.83	7.80	**6.12**	83.63	82.21	1.42	**17.28**	61.33	69.23	-7.90	**-17.78**
	3	80.75	75.90	4.85		65.74	98.01	-32.27		81.32	84.56	-3.24		102.75	67.57	35.18		68.89	79.73	-10.84	
3	1	86.46	74.82	11.64		90.97	68.83	22.14		80.51	82.00	-1.49		101.97	84.43	17.54		90.29	70.28	20.01	
	2	85.36	71.81	13.55	**9.73**	93.95	65.12	28.83	**20.87**	80.22	86.36	-6.14	**-5.18**	102.13	85.68	16.45	**13.09**	96.22	75.28	20.94	**22.38**
	3	87.00	83.01	3.99		88.46	76.82	11.64		78.39	86.30	-7.91		97.28	92.01	5.27		101.79	75.60	26.19	
4	1	91.71	79.32	12.39		98.81	74.56	24.25		109.12	73.73	35.39		105.85	68.64	37.21		80.38	74.41	5.97	
	2	99.58	78.41	21.17	**17.86**	95.15	74.41	20.74	**21.85**	106.82	72.19	34.63	**34.46**	107.19	70.30	36.89	**37.35**	80.34	74.71	5.63	**5.96**
	3	101.66	81.64	20.02		94.24	73.69	20.55		106.08	72.71	33.37		109.01	71.07	37.94		81.75	75.48	6.27	
5	1	98.78	73.60	25.18		82.16	59.29	22.87		89.11	65.42	23.69		79.10	58.06	21.04		106.73	88.62	18.11	
	2	98.02	73.67	24.35	**25.05**	85.13	57.83	27.30	**23.44**	90.48	66.97	23.51	**24.18**	78.63	59.08	19.55	**19.62**	76.43	48.94	27.49	**25.04**
	3	98.41	72.79	25.62		81.63	61.48	20.15		91.31	65.96	25.35		79.94	61.66	18.28		78.23	48.71	29.52	
6	1	85.61	74.67	10.94		72.45	41.68	30.77		71.84	71.43	0.41		80.69	64.16	16.53		78.03	64.85	13.18	
	2	89.95	70.64	19.31	**17.77**	80.24	50.32	29.92	**30.44**	78.48	76.78	1.70	**-0.05**	85.52	70.16	15.36	**15.00**	74.14	63.69	10.45	**11.41**
	3	89.00	65.93	23.07		76.88	46.26	30.62		74.48	76.73	-2.25		79.33	66.21	13.12		74.78	64.17	10.61	
7	1	79.86	88.42	-8.56		67.24	99.62	-32.38		75.89	70.40	5.49		73.24	68.86	4.38		104.99	74.97	30.02	
	2	70.17	92.38	-22.21	**-14.05**	72.79	96.43	-23.64	**-24.49**	80.69	76.35	4.34	**4.63**	82.21	69.07	13.14	**5.95**	92.65	74.83	17.82	**22.24**
	3	78.68	90.07	-11.39		76.19	93.65	-17.46		83.41	79.35	4.06		69.38	69.04	0.34		98.90	80.02	18.88	
8	1	108.47	63.67	44.80		87.44	66.67	20.77		52.87	72.11	-19.24		76.17	58.73	17.44		99.64	62.10	37.54	
	2	104.73	77.73	27.00	**28.57**	89.19	62.56	26.63	**23.90**	54.84	74.41	-19.57	**-20.59**	77.02	62.53	14.49	**14.19**	104.55	52.35	52.20	**46.12**
	3	94.03	80.12	13.91		92.03	67.72	24.31		55.17	78.12	-22.95		77.71	67.07	10.64		100.13	51.50	48.63	
**Mean**		**90.44**	**75.78**	**14.66**	**14.66**	**84.21**	**71.50**	**12.71**	**12.71**	**82.94**	**75.66**	**7.28**	**7.28**	**85.16**	**72.37**	**12.79**	**12.79**	**84.14**	**73.16**	**10.98**	**10.98**
**SE**		**1.90**	**1.59**	**3.00**	**4.62**	**2.34**	**3.43**	**4.94**	**8.81**	**3.04**	**1.23**	**3.42**	**6.08**	**2.70**	**2.21**	**3.37**	**5.68**	**2.99**	**3.02**	**4.82**	**8.48**

Group 5 Cmpd B1 (5 µg/kg) ip, QD	Trial	Force (g)		Paw Wt Distribution (g)		Force (g)		Paw Wt Distribution (g)		Force (g)		Paw Wt Distribution (g)		Force (g)		Paw Wt Distribution (g)		Force (g)		Paw Wt Distribution (g)	
	#	Left	Right	Per Trial	Mean	Left	Right	Per Trial	Mean	Left	Right	Per Trial	Mean	Left	Right	Per Trial	Mean	Left	Right	Per Trial	Mean
Animal 1	1	82.70	77.95	4.75		103.90	77.72	26.18		93.72	77.28	16.44		79.42	109.35	-29.93		86.72	61.88	24.84	
	2	101.26	70.99	30.27	**19.94**	95.01	82.59	12.42	**24.41**	91.15	86.03	5.12	**17.50**	74.00	104.43	-30.43	**-26.40**	79.31	72.22	7.09	**13.25**
	3	98.89	74.10	24.79		106.73	72.11	34.62		101.45	70.50	30.95		67.23	86.07	-18.84		80.34	72.53	7.81	
2	1	114.14	88.24	25.90		73.99	64.34	9.65		107.37	75.09	32.28		59.49	50.82	8.67		75.31	59.87	15.44	
	2	85.75	78.63	7.12	**25.17**	61.72	62.79	-1.07	**9.80**	116.92	66.90	50.02	**38.18**	57.36	48.63	8.73	**14.90**	87.76	54.69	33.07	**28.93**
	3	116.22	73.72	42.50		76.10	55.28	20.82		110.83	78.58	32.25		80.53	53.24	27.29		93.95	55.66	38.29	
3	1	62.29	64.41	-2.12		92.22	67.29	24.93		66.18	92.00	-25.82		84.12	92.28	-8.16		76.90	74.29	2.61	
	2	61.28	65.33	-4.05	**-7.17**	92.77	68.49	24.28	**24.63**	63.77	97.36	-33.59	**-31.20**	82.76	92.27	-9.51	**-10.66**	83.57	77.65	5.92	**5.04**
	3	52.23	67.56	-15.33		94.47	69.80	24.67		63.74	97.94	-34.20		78.84	93.16	-14.32		80.91	74.33	6.58	
4	1	67.34	81.38	-14.04		70.19	94.12	-23.93		79.67	80.06	-0.39		69.01	75.40	-6.39		73.72	59.27	14.45	
	2	68.91	90.00	-21.09	**-19.96**	71.63	97.53	-25.90	**-24.81**	80.88	81.54	-0.66	**-3.56**	69.49	78.22	-8.73	**-5.30**	69.84	64.75	5.09	**11.14**
	3	68.73	93.49	-24.76		71.90	96.49	-24.59		78.20	87.83	-9.63		69.86	70.63	-0.77		74.04	60.15	13.89	
5	1	101.91	46.99	54.92		80.79	84.49	-3.70		109.68	73.42	36.26		78.19	60.82	17.37		77.58	102.62	-25.04	
	2	91.76	50.10	41.66	**46.01**	82.73	87.48	-4.75	**-2.91**	109.21	72.73	36.48	**33.08**	71.10	60.05	11.05	**13.04**	84.85	88.66	-3.81	**-10.80**
	3	97.63	56.19	41.44		83.39	83.68	-0.29		94.18	67.69	26.49		68.37	57.68	10.69		85.01	88.57	-3.56	
6	1	72.88	86.00	-13.12		68.35	86.04	-17.69		69.94	20.24	49.70		83.52	55.94	27.58		88.69	75.91	12.78	
	2	57.75	85.10	-27.35	**-22.96**	72.23	83.14	-10.91	**-14.57**	72.45	20.56	51.89	**50.11**	91.67	58.77	32.90	**30.46**	92.17	73.14	19.03	**17.42**
	3	61.11	89.51	-28.40		74.44	89.54	-15.10		74.70	25.95	48.75		89.87	58.97	30.90		94.68	74.23	20.45	
7	1	92.91	79.35	13.56		79.95	96.19	-16.24		79.19	41.93	37.26		101.91	62.29	39.62		77.20	84.26	-7.06	
	2	97.00	82.00	15.00	**12.51**	82.66	98.31	-15.65	**-16.27**	89.28	41.43	47.85	**42.48**	103.41	61.71	41.70	**41.87**	77.80	84.94	-7.14	**-7.25**
	3	93.48	84.52	8.96		84.02	100.94	-16.92		86.86	44.52	42.34		108.63	64.33	44.30		78.46	86.00	-7.54	
8	1	78.03	75.65	2.38		112.44	66.97	45.47		76.69	92.41	-15.72		102.32	63.49	38.83		77.72	83.37	-5.65	
	2	87.27	73.64	13.63	**10.77**	85.95	69.73	16.22	**28.69**	78.67	92.89	-14.22	**-14.14**	100.93	59.54	41.39	**40.40**	75.73	95.46	-19.73	**-2.36**
	3	83.62	67.32	16.30		87.84	63.46	24.38		81.05	93.54	-12.49		103.14	62.15	40.99		88.71	70.40	18.31	
**Mean**		**83.13**	**75.09**	**8.04**	**8.04**	**83.56**	**79.94**	**3.62**	**3.62**	**86.49**	**69.93**	**16.56**	**16.56**	**82.30**	**70.01**	**12.29**	**12.29**	**81.71**	**74.79**	**6.92**	**6.92**
**SE**		**3.68**	**2.52**	**4.89**	**8.32**	**2.61**	**2.76**	**4.33**	**7.46**	**3.25**	**4.98**	**5.91**	**10.49**	**3.05**	**3.53**	**4.93**	**8.80**	**1.39**	**2.60**	**3.13**	**4.74**

Group 6 Cmpd B2 (10 µg/kg) ip, QD	Trial #	Force (g)		Paw Wt Distribution (g)		Force (g)		Paw Wt Distribution (g)		Force (g)		Paw Wt Distribution (g)		Force (g)		Paw Wt Distribution (g)		Force (g)		Paw Wt Distribution (g)	
		Left	Right	Per Trial	Mean	Left	Right	Per Trial	Mean	Left	Right	Per Trial	Mean	Left	Right	Per Trial	Mean	Left	Right	Per Trial	Mean
Animal 1	1	102.04	63.85	38.19		79.57	53.29	26.28		85.62	71.57	14.05		80.70	69.96	10.74		98.09	73.60	24.49	
	2	100.14	76.85	23.29	**34.88**	80.76	54.74	26.02	**26.28**	82.31	76.30	6.01	**11.96**	84.60	71.87	12.73	**15.86**	99.46	75.57	23.89	**28.07**
	3	101.86	58.70	43.16		81.74	55.19	26.55		86.98	71.16	15.82		93.31	69.20	24.11		108.46	72.63	35.83	
2	1	82.59	81.80	0.79		76.01	89.32	-13.31		79.98	90.12	-10.14		76.17	72.26	3.91		89.47	75.65	13.82	
	2	82.23	72.43	9.80	**12.31**	76.82	90.48	-13.66	**-15.17**	77.41	84.35	-6.94	**-7.64**	78.03	73.49	4.54	**18.02**	91.13	74.16	16.97	**16.90**
	3	88.76	62.43	26.33		73.49	92.04	-18.55		78.52	84.35	-5.83		96.76	51.16	45.60		94.44	74.54	19.90	
3	1	95.95	51.85	44.10		94.06	57.36	36.70		63.57	53.23	10.34		77.10	72.23	4.87		97.05	54.05	43.00	
	2	97.93	51.05	46.88	**45.76**	94.49	57.77	36.72	**38.16**	71.70	61.39	10.31	**7.30**	82.02	74.87	7.15	**6.76**	102.29	60.13	42.16	**39.03**
	3	101.13	54.84	46.29		102.44	61.39	41.05		65.81	64.55	1.26		84.13	75.87	8.26		91.95	60.02	31.93	
4	1	95.70	61.20	34.50		92.54	89.21	3.33		107.42	63.70	43.72		78.73	89.40	-10.67		73.66	77.58	-3.92	
	2	96.10	71.83	24.27	**32.13**	91.62	88.18	3.44	**4.92**	100.27	62.17	38.10	**34.40**	74.11	88.27	-14.16	**-13.24**	74.37	79.85	-5.48	**-5.05**
	3	103.10	65.49	37.61		93.59	85.59	8.00		103.27	81.90	21.37		74.90	89.78	-14.88		75.26	81.02	-5.76	
5	1	69.65	58.81	10.84		80.42	72.70	7.72		59.16	93.02	-33.86		94.21	52.44	41.77		106.67	78.62	28.05	
	2	69.34	50.51	18.83	**16.61**	80.31	80.23	0.08	**4.91**	59.33	93.64	-34.31	**-34.61**	75.69	54.40	21.29	**30.13**	108.89	76.28	32.61	**30.27**
	3	81.55	61.38	20.17		78.88	71.96	6.92		60.68	96.35	-35.67		79.65	52.31	27.34		108.02	77.87	30.15	
6	1	58.67	58.73	-0.06		69.76	74.90	-5.14		93.56	67.51	26.05		94.62	68.82	25.80		66.33	85.46	-19.13	
	2	58.24	60.17	-1.93	**-2.85**	66.71	76.90	-10.19	**-6.41**	101.91	68.36	33.55	**34.72**	85.98	66.59	19.39	**18.73**	65.43	87.71	-22.28	**-18.59**
	3	60.85	67.41	-6.56		70.22	74.11	-3.89		108.55	63.99	44.56		77.44	66.43	11.01		70.74	85.10	-14.36	
7	1	71.00	68.14	2.86		101.39	66.08	35.31		86.93	68.49	18.44		77.37	41.17	36.20		64.46	50.46	14.00	
	2	69.27	65.60	3.67	**10.71**	104.55	62.70	41.85	**39.72**	85.14	67.88	17.26	**18.37**	77.63	40.28	37.35	**33.58**	90.33	48.01	42.32	**25.22**
	3	84.97	59.36	25.61		104.76	62.76	42.00		84.56	65.16	19.40		69.96	42.77	27.19		79.76	60.41	19.35	
8	1	59.52	44.55	14.97		69.28	58.59	10.69		108.90	43.56	65.34		78.55	76.68	1.87		73.19	58.14	15.05	
	2	65.59	55.16	10.43	**11.09**	84.51	51.96	32.55	**26.36**	75.91	62.53	13.38	**29.43**	83.84	73.15	10.69	**4.51**	89.57	47.88	41.69	**20.86**
	3	64.16	56.30	7.86		90.38	54.55	35.83		73.03	63.45	9.58		74.38	73.40	0.98		61.41	55.58	5.83	
**Mean**		**81.68**	**61.60**	**20.08**	**20.08**	**84.93**	**70.08**	**14.85**	**14.85**	**83.36**	**71.61**	**11.74**	**11.74**	**81.25**	**66.95**	**14.30**	**14.30**	**86.68**	**69.60**	**17.09**	**17.09**
**SE**		**3.32**	**1.78**	**3.42**	**5.65**	**2.40**	**2.81**	**4.13**	**7.29**	**3.24**	**2.72**	**5.10**	**8.39**	**1.47**	**2.93**	**3.41**	**5.29**	**3.17**	**2.55**	**4.09**	**6.84**

Group 7 Cmpd C1 (100 µg/kg) ip, QD	Trial #	Force (g)		Paw Wt Distribution (g)		Force (g)		Paw Wt Distribution (g)		Force (g)		Paw Wt Distribution (g)		Force (g)		Paw Wt Distribution (g)		Force (g)		Paw Wt Distribution (g)	
		Left	Right	Per Trial	Mean	Left	Right	Per Trial	Mean	Left	Right	Per Trial	Mean	Left	Right	Per Trial	Mean	Left	Right	Per Trial	Mean
Animal 1	1	106.91	93.09	13.82		77.78	73.27	4.51		102.02	100.86	1.16		82.42	105.88	-23.46		82.50	75.35	7.15	
	2	97.10	74.02	23.08	**16.83**	78.00	73.36	4.64	**4.15**	99.19	99.02	0.17	**0.42**	82.32	104.56	-22.24	**-21.83**	78.22	71.57	6.65	**7.70**
	3	100.83	87.24	13.59		77.15	73.85	3.30		98.28	98.35	-0.07		83.91	103.70	-19.79		78.46	69.17	9.29	
2	1	88.86	75.90	12.96		88.44	93.33	-4.89		90.18	84.55	5.63		116.01	78.87	37.14		106.44	77.68	28.76	
	2	91.36	80.79	10.57	**9.10**	87.70	92.99	-5.29	**-5.29**	89.82	83.62	6.20	**5.52**	114.37	77.39	36.98	**38.07**	95.46	84.67	10.79	**17.84**
	3	92.10	88.34	3.76		87.69	93.37	-5.68		93.01	88.29	4.72		123.39	83.29	40.10		97.12	83.15	13.97	
3	1	82.82	74.83	7.99		89.55	68.00	21.55		83.49	86.94	-3.45		107.37	96.60	10.77		99.34	89.50	9.84	
	2	80.78	77.29	3.49	**6.17**	89.15	68.38	20.77	**20.89**	85.51	86.68	-1.17	**-2.14**	106.37	92.48	13.89	**12.33**	100.53	91.65	8.88	**9.81**
	3	83.73	76.71	7.02		89.20	68.86	20.34		85.47	87.27	-1.80		105.53	93.19	12.34		103.66	92.95	10.71	
4	1	75.68	81.72	-6.04		92.45	86.28	6.17		84.93	99.23	-14.30		72.31	120.37	-48.06		84.60	66.56	18.04	
	2	76.83	83.41	-6.58	**-8.24**	92.35	86.75	5.60	**5.26**	83.82	98.79	-14.97	**-14.77**	84.53	113.80	-29.27	**-35.72**	78.67	64.72	13.95	**14.86**
	3	75.39	87.49	-12.10		91.32	87.30	4.02		83.36	98.39	-15.03		83.50	113.32	-29.82		87.10	74.51	12.59	
5	1	87.67	85.58	2.09		98.08	68.66	29.42		97.82	70.62	27.20		107.47	63.18	44.29		94.54	80.12	14.42	
	2	89.63	86.09	3.54	**2.15**	96.27	68.93	27.34	**27.37**	96.66	70.85	25.81	**26.13**	107.10	63.54	43.56	**43.90**	99.96	81.62	18.34	**11.19**
	3	90.83	90.00	0.83		93.87	68.52	25.35		96.77	71.38	25.39		108.15	64.30	43.85		91.58	90.78	0.80	
6	1	73.27	78.85	-5.58		82.57	63.19	19.38		91.29	81.56	9.73		76.28	94.86	-18.58		58.72	96.52	-37.80	
	2	65.38	76.56	-11.18	**-8.21**	83.16	63.82	19.34	**18.37**	91.18	81.25	9.93	**9.36**	73.67	91.18	-17.51	**-17.70**	58.51	97.17	-38.66	**-37.84**
	3	66.80	74.66	-7.86		80.03	63.64	16.39		90.76	82.34	8.42		72.98	90.00	-17.02		56.71	93.77	-37.06	
7	1	110.31	68.97	41.34		92.09	81.91	10.18		93.05	63.26	29.79		88.93	99.70	-10.77		92.49	87.76	4.73	
	2	107.50	78.53	28.97	**31.16**	89.38	79.27	10.11	**10.42**	92.05	64.91	27.14	**30.01**	79.04	100.85	-21.81	**-18.36**	88.62	82.70	5.92	**9.06**
	3	97.59	74.43	23.16		89.10	78.13	10.97		94.00	60.91	33.09		73.31	95.81	-22.50		91.69	75.16	16.53	
8	1	104.13	71.29	32.84		58.69	91.15	-32.46		74.87	104.22	-29.35		80.65	85.25	-4.60		79.54	95.17	-15.63	
	2	105.75	74.77	30.98	**23.60**	58.69	91.29	-32.60	**-32.65**	76.29	103.87	-27.58	**-27.45**	77.40	86.52	-9.12	**-7.65**	83.12	89.34	-6.22	**-9.88**
	3	89.14	82.15	6.99		58.78	91.67	-32.89		76.98	102.39	-25.41		77.47	86.69	-9.22		77.90	85.68	-7.78	
**Mean**		**89.18**	**80.11**	**9.07**	**9.07**	**84.23**	**78.16**	**6.07**	**6.07**	**89.62**	**86.23**	**3.39**	**3.39**	**91.02**	**91.89**	**-0.87**	**-0.87**	**86.06**	**83.22**	**2.84**	**2.84**
**SE**		**2.65**	**1.30**	**3.01**	**5.02**	**2.31**	**2.21**	**3.67**	**6.65**	**1.51**	**2.79**	**3.76**	**6.80**	**3.35**	**3.12**	**5.76**	**10.34**	**2.81**	**1.97**	**3.71**	**6.50**

Group 8 Cmpd D1 (10 µg/kg) ip, QD	Trial #	Force (g)		Paw Wt Distribution (g)		Force (g)		Paw Wt Distribution (g)		Force (g)		Paw Wt Distribution (g)		Force (g)		Paw Wt Distribution (g)		Force (g)		Paw Wt Distribution (g)	
		Left	Right	Per Trial	Mean	Left	Right	Per Trial	Mean	Left	Right	Per Trial	Mean	Left	Right	Per Trial	Mean	Left	Right	Per Trial	Mean
Animal 1	1	106.72	92.31	14.41		101.52	88.48	13.04		105.33	71.33	34.00		91.06	89.85	1.21		100.10	84.32	15.78	
	2	109.35	91.83	17.52	**16.54**	99.64	90.43	9.21	**9.89**	99.59	73.27	26.32	**27.44**	93.22	90.36	2.86	**3.34**	103.86	86.56	17.30	**17.00**
	3	110.00	92.30	17.70		103.56	96.13	7.43		98.77	76.78	21.99		94.38	88.42	5.96		105.00	87.07	17.93	
2	1	107.31	80.18	27.13		90.41	91.84	-1.43		93.75	109.13	-15.38		118.76	79.52	39.24		91.12	102.45	-11.33	
	2	101.74	88.91	12.83	**17.55**	96.24	98.13	-1.89	**-3.71**	87.37	109.26	-21.89	**-16.62**	119.63	81.63	38.00	**26.10**	82.87	85.52	-2.65	**-5.56**
	3	98.93	86.23	12.70		92.11	99.92	-7.81		87.78	100.38	-12.60		90.48	89.43	1.05		89.98	92.69	-2.71	
3	1	122.84	80.05	42.79		115.25	80.61	34.64		120.83	80.11	40.72		120.73	81.28	39.45		122.69	87.17	35.52	
	2	125.74	80.93	44.81	**43.84**	116.29	79.98	36.31	**34.52**	120.12	77.87	42.25	**41.09**	123.09	76.89	46.20	**43.35**	106.59	88.62	17.97	**27.74**
	3	125.86	81.95	43.91		114.28	81.66	32.62		117.02	76.72	40.30		122.41	78.02	44.39		120.46	90.72	29.74	
4	1	101.93	98.49	3.44		108.27	73.66	34.61		86.69	78.09	8.60		130.80	76.91	53.89		98.57	95.31	3.26	
	2	102.09	95.37	6.72	**5.12**	109.81	75.28	34.53	**34.14**	98.59	74.14	24.45	**18.05**	123.57	78.71	44.86	**49.48**	102.20	98.26	3.94	**4.05**
	3	101.91	96.71	5.20		108.93	75.64	33.29		96.69	75.59	21.10		125.37	75.68	49.69		103.74	98.80	4.94	
5	1	96.87	77.81	19.06		95.51	85.13	10.38		106.76	68.47	38.29		101.51	76.60	24.91		109.24	76.40	32.84	
	2	95.81	82.30	13.51	**13.40**	107.78	85.46	22.32	**19.67**	101.31	71.11	30.20	**32.61**	104.55	73.07	31.48	**32.78**	121.72	77.20	44.52	**37.75**
	3	88.92	81.28	7.64		112.81	86.51	26.30		100.11	70.76	29.35		113.53	71.59	41.94		115.14	79.25	35.89	
6	1	87.43	85.64	1.79		110.67	106.68	3.99		71.25	94.49	-23.24		110.85	65.95	44.90		94.09	97.20	-3.11	
	2	85.67	84.69	0.98	**2.59**	109.64	108.30	1.34	**2.39**	88.36	100.68	-12.32	**-9.43**	117.67	74.10	43.57	**40.41**	80.96	95.93	-14.97	**-8.46**
	3	88.70	83.70	5.00		111.39	109.54	1.85		95.36	88.09	7.27		107.04	74.28	32.76		86.93	94.23	-7.30	
7	1	107.49	70.77	36.72		72.57	111.85	-39.28		92.24	79.41	12.83		76.68	87.41	-10.73		91.50	68.90	22.60	
	2	110.35	72.59	37.76	**35.79**	78.46	101.79	-23.33	**-30.09**	75.39	70.35	5.04	**10.05**	81.22	93.11	-11.89	**-9.13**	92.20	72.00	20.20	**20.65**
	3	107.47	74.57	32.90		71.49	99.14	-27.65		78.78	66.51	12.27		88.08	92.84	-4.76		92.28	73.13	19.15	
8	1	87.36	67.76	19.60		92.46	78.64	13.82		98.47	79.77	18.70		84.00	67.01	16.99		90.81	64.73	26.08	
	2	74.28	70.39	3.89	**16.20**	95.27	82.87	12.40	**12.71**	95.59	74.77	20.82	**18.48**	86.03	60.94	25.09	**20.62**	96.39	67.11	29.28	**30.29**
	3	91.75	66.65	25.10		99.73	87.82	11.91		99.02	83.11	15.91		87.96	68.18	19.78		102.47	66.95	35.52	
**Mean**		**101.52**	**82.64**	**18.88**	**18.88**	**100.59**	**90.65**	**9.94**	**9.94**	**96.47**	**81.26**	**15.21**	**15.21**	**104.69**	**78.82**	**25.87**	**25.87**	**100.04**	**84.61**	**15.43**	**15.43**
**SE**		**2.64**	**1.87**	**2.92**	**5.01**	**2.63**	**2.36**	**4.20**	**7.49**	**2.54**	**2.55**	**4.08**	**7.06**	**3.40**	**1.82**	**4.25**	**7.17**	**2.39**	**2.35**	**3.44**	**6.04**

Group 9 Cmpd E1 (50 mg/kg) po, QD	Trial	Force (g)		Paw Wt Distribution (g)		Force (g)		Paw Wt Distribution (g)		Force (g)		Paw Wt Distribution (g)		Force (g)		Paw Wt Distribution (g)		Force (g)		Paw Wt Distribution (g)	
	#	Left	Right	Per Trial	Mean	Left	Right	Per Trial	Mean	Left	Right	Per Trial	Mean	Left	Right	Per Trial	Mean	Left	Right	Per Trial	Mean
Animal 1	1	111.49	94.25	17.24		93.19	117.51	-24.32		80.80	101.81	-21.01		99.64	82.33	17.31		103.86	77.04	26.82	
	2	111.26	97.25	14.01	**16.69**	94.73	118.90	-24.17	**-25.08**	81.27	96.37	-15.10	**-17.18**	105.93	89.83	16.10	**17.30**	102.70	78.81	23.89	**23.06**
	3	116.73	97.90	18.83		92.64	119.40	-26.76		84.49	99.91	-15.42		106.09	87.60	18.49		98.21	79.73	18.48	
2	1	104.60	77.51	27.09		101.49	97.88	3.61		90.54	91.59	-1.05		85.94	108.01	-22.07		76.29	113.15	-36.86	
	2	107.29	79.56	27.73	**19.09**	108.95	93.96	14.99	**12.47**	83.01	107.91	-24.90	**-21.87**	81.52	104.62	-23.10	**-19.07**	84.77	110.49	-25.72	**-35.19**
	3	97.34	94.89	2.45		110.99	92.19	18.80		72.82	112.47	-39.65		92.33	104.38	-12.05		72.63	115.63	-43.00	
3	1	91.64	97.06	-5.42		82.33	89.12	-6.79		100.09	69.82	30.27		82.54	63.24	19.30		92.17	71.04	21.13	
	2	83.27	86.88	-3.61	**-6.86**	78.71	84.20	-5.49	**-5.01**	108.71	64.31	44.40	**29.61**	82.60	75.87	6.73	**11.95**	116.35	106.92	9.43	**16.62**
	3	86.02	97.57	-11.55		85.20	87.94	-2.74		97.06	82.89	14.17		85.57	75.75	9.82		89.12	69.82	19.30	
4	1	71.85	92.35	-20.50		91.10	69.65	21.45		126.65	70.62	56.03		86.79	83.50	3.29		107.93	95.40	12.53	
	2	71.73	94.91	-23.18	**-22.86**	91.77	70.50	21.27	**21.87**	118.91	70.01	48.90	**50.28**	87.74	83.36	4.38	**3.69**	104.73	90.55	14.18	**14.17**
	3	73.72	98.62	-24.90		94.02	71.14	22.88		118.43	72.52	45.91		88.01	84.62	3.39		105.13	89.32	15.81	
5	1	105.57	95.20	10.37		95.16	80.27	14.89		80.37	100.81	-20.44		100.11	82.04	18.07		94.16	85.02	9.14	
	2	105.46	94.81	10.65	**7.63**	95.24	81.12	14.12	**14.69**	81.98	97.56	-15.58	**-8.60**	97.52	82.30	15.22	**18.65**	86.07	83.70	2.37	**5.84**
	3	100.00	98.14	1.86		95.91	80.86	15.05		97.85	87.64	10.21		103.50	80.84	22.66		92.72	86.70	6.02	
6	1	101.76	86.20	15.56		111.96	75.03	36.93		121.55	83.06	38.49		82.38	77.67	4.71		99.23	91.61	7.62	
	2	87.46	84.98	2.48	**8.30**	113.27	72.60	40.67	**36.85**	117.74	84.00	33.74	**32.85**	83.80	78.60	5.20	**5.05**	121.59	98.45	23.14	**18.73**
	3	95.65	88.80	6.85		111.26	78.32	32.94		112.74	86.43	26.31		83.67	78.43	5.24		122.72	97.30	25.42	
7	1	79.95	99.17	-19.22		92.93	103.37	-10.44		83.75	96.80	-13.05		77.18	76.99	0.19		90.99	84.85	6.14	
	2	81.31	88.66	-7.35	**-11.76**	94.31	103.17	-8.86	**-11.33**	86.02	96.73	-10.71	**-8.94**	77.49	78.00	-0.51	**-0.53**	91.39	88.50	2.89	**6.99**
	3	83.65	92.35	-8.70		97.88	112.56	-14.68		92.61	95.68	-3.07		77.26	78.54	-1.28		96.50	84.56	11.94	
8	1	100.17	75.06	25.11		133.30	74.56	58.74		70.61	107.54	-36.93		97.98	83.11	14.87		113.01	94.98	18.03	
	2	120.62	90.15	30.47	**26.99**	112.26	80.17	32.09	**38.96**	77.81	112.61	-34.80	**-30.62**	104.30	92.70	11.60	**13.27**	106.45	94.68	11.77	**14.34**
	3	106.26	80.87	25.39		99.56	73.52	26.04		84.70	104.84	-20.14		98.04	84.69	13.35		103.96	90.75	13.21	
**Mean**		**95.62**	**90.96**	**4.65**	**4.65**	**99.09**	**88.66**	**10.43**	**10.43**	**94.60**	**91.41**	**3.19**	**3.19**	**90.33**	**84.04**	**6.29**	**6.29**	**98.86**	**90.79**	**8.07**	**8.07**
**SE**		**2.92**	**1.46**	**3.51**	**6.03**	**2.46**	**3.32**	**4.60**	**8.06**	**3.47**	**2.92**	**6.16**	**10.58**	**1.97**	**2.06**	**2.47**	**4.34**	**2.61**	**2.48**	**3.72**	**6.50**

Group 10 Cmpd F1 (50 mg/kg) po, QD	Trial	Force (g)		Paw Wt Distribution (g)		Force (g)		Paw Wt Distribution (g)		Force (g)		Paw Wt Distribution (g)		Force (g)		Paw Wt Distribution (g)		Force (g)		Paw Wt Distribution (g)	
	#	Left	Right	Per Trial	Mean	Left	Right	Per Trial	Mean	Left	Right	Per Trial	Mean	Left	Right	Per Trial	Mean	Left	Right	Per Trial	Mean
Animal 1	1	89.35	80.58	8.77		118.41	81.07	37.34		103.24	101.30	1.94		107.10	78.51	28.59		100.04	86.82	13.22	
	2	84.93	94.15	-9.22	**1.67**	118.48	79.49	38.99	**37.96**	104.60	98.84	5.76	**4.82**	127.40	84.21	43.19	**37.46**	101.29	91.03	10.26	**10.37**
	3	91.50	86.03	5.47		119.65	82.10	37.55		104.28	97.52	6.76		123.23	82.62	40.61		100.56	92.92	7.64	
2	1	108.29	76.39	31.90		92.15	100.78	-8.63		108.76	90.53	18.23		96.37	74.44	21.93		90.66	106.15	-15.49	
	2	108.21	78.51	29.70	**36.64**	88.24	94.49	-6.25	**-6.79**	118.11	71.87	46.24	**36.41**	105.48	71.76	33.72	**26.74**	93.39	117.37	-23.98	**-16.88**
	3	120.50	72.19	48.31		95.68	101.18	-5.50		119.80	75.05	44.75		102.34	77.76	24.58		89.85	101.03	-11.18	
3	1	105.22	83.33	21.89		90.31	111.64	-21.33		87.45	100.72	-13.27		105.90	81.51	24.39		95.58	83.05	12.53	
	2	113.66	78.15	35.51	**26.18**	96.44	108.22	-11.78	**-14.90**	86.73	97.98	-11.25	**-9.15**	113.14	83.35	29.79	**23.02**	96.06	81.51	14.55	**14.07**
	3	108.84	87.69	21.15		95.01	106.61	-11.60		88.21	91.13	-2.92		99.42	84.54	14.88		98.43	83.31	15.12	
4	1	102.76	79.26	23.50		103.13	84.63	18.50		111.70	88.66	23.04		95.84	97.90	-2.06		111.03	75.10	35.93	
	2	103.65	80.28	23.37	**24.50**	108.63	82.79	25.84	**22.64**	106.74	99.67	7.07	**12.40**	93.55	95.73	-2.18	**-2.80**	106.19	73.51	32.68	**34.23**
	3	105.91	79.29	26.62		109.15	85.57	23.58		100.69	93.59	7.10		92.32	96.48	-4.16		111.64	77.55	34.09	
5	1	92.81	115.67	-22.86		97.17	97.47	-0.30		91.04	71.32	19.72		95.47	59.85	35.62		95.98	82.71	13.27	
	2	75.80	119.60	-43.80	**-38.63**	94.85	96.53	-1.68	**-2.41**	92.17	78.03	14.14	**17.32**	83.12	72.63	10.49	**30.15**	106.13	80.38	25.75	**17.86**
	3	79.42	128.65	-49.23		90.93	96.19	-5.26		91.40	73.30	18.10		105.74	61.39	44.35		94.36	79.80	14.56	
6	1	101.49	78.93	22.56		102.65	110.05	-7.40		117.71	78.50	39.21		100.14	63.07	37.07		86.05	94.08	-8.03	
	2	99.62	84.55	15.07	**16.30**	103.37	107.66	-4.29	**-11.21**	116.37	88.87	27.50	**31.17**	95.71	73.26	22.45	**26.16**	92.97	100.84	-7.87	**-8.83**
	3	100.46	89.19	11.27		85.82	107.75	-21.93		115.49	88.68	26.81		102.35	83.40	18.95		88.23	98.82	-10.59	
7	1	72.83	96.19	-23.36		93.75	82.86	10.89		74.41	87.50	-13.09		90.63	88.39	2.24		93.40	77.36	16.04	
	2	74.28	91.34	-17.06	**-19.49**	96.46	83.28	13.18	**12.03**	81.09	96.17	-15.08	**-8.04**	89.31	81.02	8.29	**5.80**	97.52	73.27	24.25	**14.70**
	3	78.52	96.57	-18.05		96.25	84.23	12.02		79.55	75.51	4.04		83.42	76.55	6.87		97.83	94.01	3.82	
8	1	86.75	61.69	25.06		106.52	86.21	20.31		102.26	65.13	37.13		89.59	92.19	-2.60		97.87	75.37	22.50	
	2	84.83	61.61	23.22	**38.76**	97.24	80.51	16.73	**17.51**	103.03	64.65	38.38	**41.08**	85.21	92.10	-6.89	**-7.46**	96.60	75.57	21.03	**20.73**
	3	111.83	43.82	68.01		103.23	87.73	15.50		109.48	61.76	47.72		83.53	96.43	-12.90		90.80	72.14	18.66	
**Mean**		**95.89**	**85.15**	**10.74**	**10.74**	**100.15**	**93.29**	**6.85**	**6.85**	**100.60**	**84.85**	**15.75**	**15.75**	**98.60**	**81.21**	**17.38**	**17.38**	**97.19**	**86.40**	**10.78**	**10.78**
**SE**		**2.82**	**3.74**	**5.76**	**9.77**	**1.93**	**2.27**	**3.71**	**6.59**	**2.71**	**2.58**	**4.04**	**6.85**	**2.37**	**2.24**	**3.55**	**5.86**	**1.35**	**2.45**	**3.31**	**5.79**

Weight Bearing Data for MMT/MCLT Trial 2

Table 74. Individual animal weight bearing data for the MMT/MCLT Trial 2 Day 7 Data

		Day 7 (Pre Tx)				Day 7 (1hr)				Day 7 (3hr)				Day 7 (5hr)			
Group 1 Vehicle (Saline) IA, 1x/week	Trial #	Force (g)		Paw Wt Distribution (g)		Force (g)		Paw Wt Distribution (g)		Force (g)		Paw Wt Distribution (g)		Force (g)		Paw Wt Distribution (g)	
		Left	Right	Per Trial	Mean	Left	Right	Per Trial	Mean	Left	Right	Per Trial	Mean	Left	Right	Per Trial	Mean
Animal 1	1	96	56	40		74	56	18		73	70	3		74	54	20	
	2	98	60	38	39.67	75	53	22	20.33	73	65	8	6.00	74	54	20	20.33
	3	99	58	41		76	55	21		74	67	7		78	57	21	
2	1	82	68	14		78	70	8		76	73	3		74	62	12	
	2	82	69	13	11.33	74	68	6	7.33	75	61	14	7.33	76	63	13	13.00
	3	80	73	7		79	71	8		71	66	5		74	60	14	
3	1	80	67	13		72	61	11		74	67	7		70	56	14	
	2	81	66	15	16.00	75	63	12	10.33	72	67	5	6.33	67	53	14	14.00
	3	87	67	20		72	64	8		74	67	7		66	52	14	
4	1	81	62	19		83	50	33		75	60	15		62	57	5	
	2	81	59	22	21.00	85	54	31	32.33	73	61	12	13.33	62	56	6	5.67
	3	79	57	22		83	50	33		74	61	13		61	55	6	
5	1	82	76	6		81	64	17		86	63	23		70	57	13	
	2	83	75	8	7.67	82	66	16	16.67	87	63	24	24.33	70	57	13	12.67
	3	84	75	9		82	65	17		90	64	26		71	59	12	
6	1	84	72	12		86	55	31		76	60	16		83	73	10	
	2	82	67	15	14.67	86	56	30	29.67	76	58	18	17.67	80	72	8	8.00
	3	86	69	17		85	57	28		79	60	19		80	74	6	
7	1	95	84	11		91	87	4		84	74	10		82	76	6	
	2	92	83	9	10.33	87	82	5	3.67	83	73	10	11.00	82	80	2	4.00
	3	96	85	11		87	85	2		84	71	13		83	79	4	
8	1	85	55	30		94	61	33		82	51	31		81	42	39	
	2	82	55	27	29.00	92	61	31	32.33	82	50	32	32.33	81	43	38	38.00
	3	85	55	30		95	62	33		87	53	34		81	44	37	
9	1	86	61	25		77	58	19		79	54	25		75	62	13	
	2	87	59	28	26.33	76	59	17	17.33	77	53	24	24.33	73	62	11	12.67
	3	88	62	26		75	59	16		76	52	24		76	62	14	
10	1	71	65	6		81	77	4		69	52	17		77	47	30	
	2	72	64	8	7.67	81	77	4	3.00	69	51	18	18.00	76	46	30	30.00
	3	73	64	9		80	79	1		69	50	19		77	47	30	
Mean		84.63	66.27	18.37		81.47	64.17	17.30	17.30	77.30	61.23	16.07	16.07	74.53	58.70	15.83	15.83
SE		1.30	1.57	1.89		1.17	1.87	2.01	3.58	1.07	1.37	1.61	2.81	1.16	1.91	1.91	3.41

Group 2 Clonidine (d7, d14, d18-22) 00μg/kg (d7,18-22) or 200μg/kg (	Trial #	Force (g)		Paw Wt Distribution (g)		Force (g)		Paw Wt Distribution (g)		Force (g)		Paw Wt Distribution (g)		Force (g)		Paw Wt Distribution (g)	
		Left	Right	Per Trial	Mean	Left	Right	Per Trial	Mean	Left	Right	Per Trial	Mean	Left	Right	Per Trial	Mean
Animal 1	1	82	68	14		76	67	9		80	63	17		74	61	13	
	2	84	65	19	18.67	76	67	9	9.00	80	63	17	17.67	73	61	12	10.00
	3	80	57	23		77	68	9		81	62	19		71	66	5	
2	1	88	59	29		95	71	24		77	71	6		81	74	7	
	2	86	56	30	29.67	95	71	24	23.67	77	71	6	6.00	81	72	9	7.33
	3	84	54	30		94	71	23		77	71	6		80	74	6	
3	1	79	73	6		81	77	4		87	71	16		80	64	16	
	2	82	74	8	6.67	81	77	4	4.33	89	76	13	15.33	81	64	17	15.33
	3	79	73	6		82	77	5		91	74	17		81	68	13	
4	1	80	54	26		87	79	8		83	72	11		72	59	13	
	2	83	54	29	27.67	87	78	9	8.67	84	71	13	12.67	72	58	14	14.00
	3	83	55	28		88	79	9		85	71	14		75	60	15	
5	1	89	66	23		92	80	12		83	72	11		92	72	20	
	2	89	66	23	23.33	92	80	12	12.33	84	71	13	12.67	92	72	20	20.00
	3	89	65	24		91	78	13		85	71	14		91	71	20	
6	1	70	64	6		79	68	11		80	64	16		94	66	28	
	2	70	64	6	5.00	78	68	10	10.67	81	69	12	12.33	95	65	30	30.33
	3	69	66	3		79	68	11		82	73	9		99	66	33	
7	1	87	67	20		99	93	6		81	64	17		87	82	5	
	2	87	66	21	22.00	99	93	6	5.67	81	66	15	15.33	86	81	5	6.00
	3	91	66	25		98	93	5		79	65	14		87	79	8	
8	1	71	62	9		71	63	8		90	71	19		94	77	17	
	2	73	69	4	5.67	70	63	7	7.00	90	71	19	19.00	93	76	17	16.33
	3	72	68	4		71	65	6		90	71	19		94	79	15	
9	1	70	53	17		75	71	4		80	61	19		99	76	23	
	2	71	54	17	17.00	76	71	5	8.00	79	63	16	17.00	100	77	23	21.67
	3	70	53	17		76	61	15		80	64	16		97	78	19	
10	1	83	67	16		92	74	18		97	70	27		83	69	14	
	2	81	67	14	14.67	93	73	20	20.33	99	71	28	29.00	85	62	23	19.00
	3	80	66	14		93	70	23		97	65	32		83	63	20	
Mean		80.07	63.03	17.03		84.77	73.80	10.97	10.97	84.30	68.60	15.70	15.70	85.73	69.73	16.00	16.00
SE		1.28	1.18	1.61		1.67	1.54	1.16	1.99	1.11	0.75	1.08	1.88	1.63	1.31	1.34	2.31

Group 3<br>Cmpd A1<br>IA, 1x/week	Trial #	Force (g)		Paw Wt Distribution (g)		Force (g)		Paw Wt Distribution (g)		Force (g)		Paw Wt Distribution (g)		Force (g)		Paw Wt Distribution (g)	
		Left	Right	Per Trial	Mean	Left	Right	Per Trial	Mean	Left	Right	Per Trial	Mean	Left	Right	Per Trial	Mean
Animal 1	1	92	72	20		90	79	11		94	60	34		83	56	27	
	2	95	68	27	24.33	90	79	11	12.00	94	60	34	33.67	83	58	25	25.33
	3	94	68	26		98	84	14		91	58	33		83	59	24	
2	1	90	69	21		97	80	17		86	66	20		85	52	33	
	2	87	67	20	20.67	96	83	13	13.00	80	58	22	24.33	83	50	33	34.33
	3	84	63	21		96	87	9		84	53	31		86	49	37	
3	1	83	73	10		81	56	25		66	49	17		68	43	25	
	2	85	75	10	8.00	81	57	24	24.33	65	49	16	16.00	67	45	22	22.67
	3	83	79	4		82	58	24		65	50	15		67	46	21	
4	1	92	63	29		86	71	15		84	45	39		65	51	14	
	2	95	64	31	30.33	85	71	14	14.33	84	47	37	39.00	66	52	14	13.67
	3	94	63	31		85	71	14		88	47	41		66	53	13	
5	1	90	62	28		82	65	17		89	51	38		89	46	43	
	2	90	61	29	30.00	82	66	16	15.67	89	51	38	38.00	88	48	40	40.00
	3	90	57	33		80	66	14		89	51	38		88	51	37	
6	1	84	57	27		81	69	12		63	50	13		74	52	22	
	2	80	59	21	24.67	80	69	11	10.33	63	50	13	13.00	74	52	22	21.67
	3	82	56	26		80	72	8		64	51	13		74	53	21	
7	1	84	73	11		95	77	18		78	54	24		97	53	44	
	2	83	75	8	10.33	75	76	-1	12.00	80	55	25	24.33	97	55	42	43.67
	3	86	74	12		94	75	19		79	55	24		99	54	45	
8	1	85	70	15		94	77	17		72	64	8		73	44	29	
	2	82	69	13	14.33	94	77	17	17.00	71	65	6	6.67	72	42	30	29.67
	3	86	71	15		93	76	17		71	65	6		75	45	30	
9	1	80	72	8		95	88	7		64	49	15		83	52	31	
	2	83	73	10	9.00	95	89	6	6.67	65	48	17	16.00	81	51	30	29.33
	3	80	71	9		95	88	7		65	49	16		78	51	27	
10	1	94	69	25		83	72	11		76	55	21		74	47	27	
	2	94	73	21	24.00	83	72	11	11.00	75	56	19	20.00	73	43	30	28.33
	3	94	68	26		84	73	11		74	54	20		71	43	28	
Mean		87.37	67.80	19.57		87.73	74.10	13.63	13.63	76.93	53.83	23.10	23.10	78.73	49.87	28.87	28.87
SE		0.93	1.10	1.54		1.24	1.61	1.03	1.50	1.89	1.07	1.95	3.45	1.77	0.84	1.58	2.80

Group 4<br>Cmpd B<br>IA, 1x/week	Trial #	Force (g)		Paw Wt Distribution (g)		Force (g)		Paw Wt Distribution (g)		Force (g)		Paw Wt Distribution (g)		Force (g)		Paw Wt Distribution (g)	
		Left	Right	Per Trial	Mean	Left	Right	Per Trial	Mean	Left	Right	Per Trial	Mean	Left	Right	Per Trial	Mean
Animal 1	1	81	74	7		92	70	22		81	53	28		89	61	28	
	2	85	72	13	10.67	92	79	13	16.33	82	57	25	26.33	88	61	27	26.33
	3	84	72	12		90	76	14		84	58	26		87	63	24	
2	1	99	71	28		82	57	25		79	63	16		73	59	14	
	2	98	72	26	20.33	80	58	22	22.67	79	62	17	16.67	72	60	12	13.33
	3	79	72	7		78	57	21		79	62	17		74	60	14	
3	1	81	61	20		92	63	29		93	65	28		71	51	20	
	2	81	68	13	14.33	92	64	28	29.00	96	62	34	27.67	70	53	17	18.00
	3	80	70	10		91	61	30		93	72	21		71	54	17	
4	1	74	56	18		85	59	26		84	58	26		74	66	8	
	2	74	57	17	17.67	85	63	22	22.67	81	57	24	24.67	74	66	8	8.00
	3	74	56	18		85	65	20		82	58	24		73	65	8	
5	1	79	69	10		70	61	9		71	61	10		75	52	23	
	2	78	69	9	8.67	70	62	8	8.33	71	62	9	8.67	75	55	20	21.67
	3	76	69	7		69	61	8		71	64	7		76	54	22	
6	1	80	69	11		62	52	10		64	53	11		71	51	20	
	2	79	70	9	9.33	62	51	11	10.00	64	59	5	7.33	72	50	22	20.00
	3	76	68	8		61	52	9		65	59	6		70	52	18	
7	1	71	67	4		83	55	28		85	58	27		73	69	4	
	2	73	68	5	3.67	84	55	29	28.00	87	60	27	27.00	72	68	4	4.33
	3	73	71	2		83	56	27		87	60	27		72	67	5	
8	1	76	58	18		81	66	15		65	59	6		75	55	20	
	2	80	64	16	18.00	82	69	13	13.67	69	61	8	7.00	71	64	7	11.33
	3	83	63	20		79	66	13		69	62	7		74	67	7	
9	1	90	82	8		87	67	20		73	68	5		82	65	17	
	2	89	83	6	7.33	84	69	15	17.67	73	67	6	5.67	82	67	15	16.00
	3	92	84	8		85	67	18		72	66	6		80	64	16	
10	1	91	68	23		86	58	28		89	84	5		86	54	32	
	2	89	70	19	20.67	87	58	29	28.00	88	83	5	6.00	85	54	31	31.33
	3	87	67	20		86	59	27		87	79	8		84	53	31	
Mean		81.73	68.67	13.07		81.50	61.87	19.63	19.63	78.77	63.07	15.70	15.70	76.37	59.33	17.03	17.03
SE		1.34	1.27	1.24		1.66	1.23	1.38	2.40	1.70	1.39	1.76	3.08	1.10	1.14	1.51	2.61

Group 5 Cmpd C1 IA, 1x/week	Trial	Force (g)		Paw Wt Distribution (g)		Force (g)		Paw Wt Distribution (g)		Force (g)		Paw Wt Distribution (g)		Force (g)		Paw Wt Distribution (g)	
	#	Left	Right	Per Trial	Mean	Left	Right	Per Trial	Mean	Left	Right	Per Trial	Mean	Left	Right	Per Trial	Mean
Animal 1	1	63	51	12		74	64	10		84	75	9		82	61	21	
	2	62	51	11	10.00	74	64	10	10.00	83	72	11	8.33	82	62	20	19.67
	3	62	55	7		72	62	10		85	80	5		81	63	18	
2	1	92	83	9		100	87	13		93	68	25		87	63	24	
	2	92	78	14	12.67	100	87	13	13.00	90	67	23	24.00	86	64	22	20.67
	3	97	82	15		101	88	13		93	69	24		83	67	16	
3	1	89	63	26		85	66	19		82	69	13		82	68	14	
	2	90	62	28	27.33	84	65	19	19.00	82	69	13	11.67	81	67	14	13.67
	3	92	64	28		84	65	19		80	71	9		81	68	13	
4	1	70	55	15		70	54	16		57	42	15		63	45	18	
	2	70	54	16	15.33	70	54	16	16.33	57	42	15	14.00	63	47	16	17.00
	3	70	55	15		71	54	17		57	45	12		63	46	17	
5	1	73	62	11		70	54	16		72	63	9		61	55	6	
	2	71	64	7	9.33	70	54	16	16.33	72	65	7	7.33	61	56	5	5.67
	3	74	64	10		74	57	17		74	68	6		58	52	6	
6	1	91	82	9		88	77	11		83	43	40		86	44	42	
	2	91	80	11	9.67	86	77	9	10.00	84	43	41	40.33	86	45	41	43.00
	3	90	81	9		86	76	10		84	44	40		90	44	46	
7	1	86	56	30		73	63	10		65	51	14		60	51	9	
	2	86	53	33	32.67	70	64	6	7.67	62	51	11	11.33	60	52	8	8.00
	3	88	53	35		71	64	7		59	50	9		58	51	7	
8	1	96	77	19		79	71	8		77	40	37		75	44	31	
	2	96	79	17	18.33	78	71	7	7.67	77	41	36	35.67	75	40	35	33.67
	3	96	77	19		79	71	8		76	42	34		77	42	35	
9	1	96	66	30		85	68	17		61	53	8		66	54	12	
	2	96	60	36	30.67	84	66	18	18.33	63	51	12	10.00	66	54	12	10.33
	3	92	66	26		85	65	20		63	53	10		64	57	7	
10	1	76	59	17		89	74	15		74	51	23		61	33	28	
	2	73	60	13	12.67	80	74	6	10.00	77	52	25	24.33	60	34	26	24.67
	3	74	66	8		83	74	9		78	53	25		56	36	20	
Mean		83.13	65.27	17.87		80.50	67.67	12.83	12.83	74.80	56.10	18.70	18.70	71.80	52.17	19.63	19.63
SE		2.15	1.96	1.65		1.69	1.77	0.81	1.38	2.00	2.23	2.10	3.73	2.05	1.89	2.09	3.69

Group 6 Cmpd C2 IA, 1x/week	Trial	Force (g)		Paw Wt Distribution (g)		Force (g)		Paw Wt Distribution (g)		Force (g)		Paw Wt Distribution (g)		Force (g)		Paw Wt Distribution (g)	
	#	Left	Right	Per Trial	Mean	Left	Right	Per Trial	Mean	Left	Right	Per Trial	Mean	Left	Right	Per Trial	Mean
Animal 1	1	98	67	31		77	54	23		78	33	45		75	49	26	
	2	95	71	24	27.33	75	52	23	24.33	77	33	44	42.67	82	49	33	28.33
	3	99	72	27		78	51	27		75	36	39		77	51	26	
2	1	74	58	16		75	59	16		54	42	12		67	35	32	
	2	79	59	20	18.33	79	62	17	15.67	55	43	12	13.33	68	36	32	31.33
	3	76	57	19		74	60	14		59	43	16		65	35	30	
3	1	67	52	15		76	59	17		67	42	25		46	32	14	
	2	70	56	14	14.33	77	60	17	17.00	66	45	21	22.67	44	35	9	11.00
	3	70	56	14		73	56	17		67	45	22		46	36	10	
4	1	73	61	12		89	67	22		84	76	8		93	67	26	
	2	73	64	9	10.00	89	70	19	20.67	84	73	11	10.00	94	64	30	27.00
	3	72	63	9		88	67	21		84	73	11		90	65	25	
5	1	85	71	14		72	62	10		72	66	6		85	65	20	
	2	84	75	9	10.00	69	63	6	8.67	72	68	4	4.33	83	66	17	19.00
	3	85	78	7		76	66	10		70	67	3		86	66	20	
6	1	82	71	11		89	56	33		62	60	2		80	53	27	
	2	81	71	10	8.67	86	53	33	32.33	62	60	2	4.00	80	53	27	27.67
	3	82	77	5		88	57	31		68	60	8		82	53	29	
	1	80	51	29		75	67	8		80	63	17		71	48	23	
	2	82	57	25	26.00	78	67	11	9.67	80	64	16	15.00	72	45	27	23.33
	3	83	59	24		77	67	10		80	68	12		70	50	20	
8	1	86	69	17		87	55	32		82	43	39		62	30	32	
	2	87	70	17	17.33	87	54	33	31.67	83	44	39	39.33	63	30	33	32.00
	3	86	68	18		84	54	30		84	44	40		62	31	31	
9	1	84	73	11		70	61	9		84	49	35		88	58	30	
	2	88	74	14	10.67	80	71	9	8.00	85	51	34	34.67	86	57	29	29.33
	3	83	76	7		73	67	6		86	51	35		84	55	29	
10	1	86	53	33		81	58	23		77	40	37		73	42	31	
	2	85	58	27	29.00	84	57	27	25.00	79	39	40	36.33	72	43	29	31.00
	3	83	56	27		79	54	25		74	42	32		76	43	33	
Mean		81.93	64.77	17.17		79.50	60.20	19.30	19.30	74.33	52.10	22.23	22.23	74.07	48.07	26.00	26.00
SE		1.42	1.51	1.44		1.12	1.07	1.61	2.86	1.72	2.38	2.65	4.71	2.42	2.21	1.21	2.08

Group 7<br>Cmpd D<br>IA, 1x/week	Trial	Force (g)		Paw Wt Distribution (g)		Force (g)		Paw Wt Distribution (g)		Force (g)		Paw Wt Distribution (g)		Force (g)		Paw Wt Distribution (g)	
	#	Left	Right	Per Trial	Mean	Left	Right	Per Trial	Mean	Left	Right	Per Trial	Mean	Left	Right	Per Trial	Mean
Animal 1	1	82	71	11		82	63	19		71	64	7		70	65	5	
	2	83	72	11	11.33	83	63	20	21.00	71	64	7	7.67	68	64	4	5.67
	3	83	71	12		87	63	24		71	62	9		70	62	8	
2	1	96	82	14		83	75	8		83	76	7		78	62	16	
	2	94	80	14	12.33	80	75	5	5.00	87	75	12	9.00	78	66	12	12.33
	3	89	80	9		77	75	2		84	76	8		76	67	9	
3	1	91	69	22		77	47	30		73	59	14		76	53	23	
	2	91	70	21	21.67	76	46	30	30.00	74	59	15	14.33	76	53	23	22.33
	3	91	69	22		77	47	30		73	59	14		75	54	21	
4	1	82	71	11		83	73	10		83	67	16		73	62	11	
	2	86	71	15	12.00	84	73	11	12.00	82	65	17	17.00	73	65	8	9.00
	3	82	72	10		84	69	15		82	64	18		72	64	8	
5	1	86	51	35		84	63	21		87	59	28		72	60	12	
	2	85	51	34	33.33	84	63	21	22.00	87	60	27	26.67	72	60	12	12.67
	3	84	53	31		84	60	24		86	61	25		74	60	14	
6	1	76	51	25		86	52	34		83	50	33		94	54	40	
	2	70	57	13	19.33	86	54	32	32.00	84	50	34	33.00	94	54	40	39.67
	3	72	52	20		84	54	30		82	50	32		92	53	39	
7	1	82	63	19		96	73	23		83	70	13		79	55	24	
	2	85	71	14	17.00	95	71	24	22.67	85	70	15	15.00	78	58	20	21.67
	3	86	68	18		92	71	21		84	67	17		78	57	21	
8	1	71	66	5		57	50	7		78	66	12		86	67	19	
	2	72	66	6	5.67	57	51	6	6.00	78	66	12	12.00	84	66	18	18.00
	3	72	66	6		57	52	5		79	67	12		81	64	17	
9	1	85	80	5		80	70	10		81	45	36		80	59	21	
	2	83	77	6	5.67	82	68	14	12.67	83	46	37	37.33	80	59	21	21.67
	3	82	76	6		81	67	14		84	45	39		81	58	23	
10	1	84	60	24		90	61	29		93	80	13		92	52	40	
	2	85	63	22	22.33	87	61	26	27.33	91	81	10	10.67	91	49	42	40.67
	3	83	62	21		90	63	27		90	81	9		89	49	40	
Mean		83.10	67.03	16.07		81.50	62.43	19.07	19.07	81.73	63.47	18.27	18.27	79.40	59.03	20.37	20.37
SE		1.22	1.68	1.55		1.76	1.70	1.73	3.05	1.08	1.89	1.85	3.29	1.39	0.99	2.11	3.75

Group 8<br>Cmpd E<br>IA, 1x/week	Trial	Force (g)		Paw Wt Distribution (g)		Force (g)		Paw Wt Distribution (g)		Force (g)		Paw Wt Distribution (g)		Force (g)		Paw Wt Distribution (g)	
	#	Left	Right	Per Trial	Mean	Left	Right	Per Trial	Mean	Left	Right	Per Trial	Mean	Left	Right	Per Trial	Mean
Animal 1	1	79	62	17		79	63	16		77	64	13		79	50	29	
	2	79	64	15	15.33	78	67	11	13.67	75	67	8	12.67	81	50	31	31.00
	3	76	62	14		78	64	14		79	62	17		83	50	33	
2	1	78	58	20		83	50	33		70	59	11		77	59	18	
	2	76	58	18	19.00	82	54	28	30.00	72	59	13	12.00	77	55	22	20.33
	3	82	63	19		83	54	29		71	59	12		78	57	21	
3	1	86	69	17		82	61	21		78	69	9		72	50	22	
	2	86	70	16	16.00	80	59	21	21.00	78	69	9	9.00	72	50	22	21.33
	3	86	71	15		80	59	21		78	69	9		71	51	20	
4	1	74	57	17		74	60	14		86	73	13		79	66	13	
	2	73	58	15	18.00	76	64	12	13.00	87	71	16	15.00	79	67	12	12.33
	3	77	55	22		74	61	13		88	72	16		79	67	12	
5	1	84	60	24		81	66	15		78	64	14		75	52	23	
	2	83	60	23	23.33	81	68	13	14.00	79	63	16	14.00	75	54	21	22.33
	3	82	59	23		80	66	14		76	64	12		77	54	23	
6	1	69	63	6		72	65	7		73	51	22		68	51	17	
	2	68	64	4	5.00	72	65	7	6.33	74	53	21	21.67	68	52	16	16.33
	3	68	63	5		72	67	5		74	52	22		69	53	16	
7	1	85	66	19		82	61	21		69	49	20		72	53	19	
	2	84	61	23	21.33	82	61	21	20.67	69	49	20	19.67	71	51	20	20.67
	3	83	61	22		82	62	20		69	50	19		75	52	23	
8	1	81	74	7		96	53	43		85	53	32		88	45	43	
	2	80	71	9	7.67	96	53	43	42.33	85	53	32	33.00	87	44	43	44.33
	3	79	72	7		96	55	41		86	51	35		89	42	47	
9	1	87	63	24		77	67	10		64	55	9		69	42	27	
	2	89	63	26	24.00	76	71	5	6.33	60	54	6	6.33	71	45	26	24.33
	3	84	62	22		74	70	4		61	57	4		67	47	20	
10	1	74	52	22		78	49	29		62	44	18		78	50	28	
	2	75	51	24	23.00	80	53	27	25.67	64	41	23	19.67	79	50	29	28.67
	3	75	52	23		78	57	21		62	44	18		79	50	29	
Mean		79.40	62.13	17.27		80.13	60.83	19.30	19.30	74.30	58.00	16.30	16.30	76.13	51.97	24.17	24.17
SE		1.05	1.08	1.18		1.16	1.10	2.00	3.54	1.51	1.64	1.38	2.41	1.08	1.16	1.59	2.82

Table 75. Individual animal data for MMT/MCLT IA Trial 2 Day 14 data

		Day 14 (Pre Tx)				Day 14 (1hr)				Day 14 (3hr)				Day 14 (5hr)			
Group 1 Vehicle (Saline) IA, 1x/week	Trial	Force (g)		Paw Wt Distribution (g)		Force (g)		Paw Wt Distribution (g)		Force (g)		Paw Wt Distribution (g)		Force (g)		Paw Wt Distribution (g)	
	#	Left	Right	Per Trial	Mean	Left	Right	Per Trial	Mean	Left	Right	Per Trial	Mean	Left	Right	Per Trial	Mean
Animal 1	1	79	61	18		72	63	9		81	64	17		90	75	15	
	2	83	62	21	20.67	73	67	6	7.67	83	63	20	17.33	90	74	16	16.00
	3	85	62	23		72	64	8		83	68	15		91	74	17	
2	1	78	73	5		85	69	16		75	67	8		76	67	9	
	2	75	72	3	4.33	81	68	13	14.67	76	69	7	6.33	77	68	9	10.00
	3	77	72	5		84	69	15		78	74	4		81	69	12	
3	1	98	76	22		79	66	13		77	70	7		74	65	9	
	2	97	73	24	25.00	78	65	13	14.00	75	69	6	6.00	73	63	10	8.00
	3	96	67	29		79	63	16		75	70	5		75	70	5	
4	1	71	56	15		71	51	20		68	50	18		71	64	7	
	2	71	57	14	14.67	72	48	24	23.00	69	50	19	18.00	72	65	7	7.33
	3	74	59	15		73	48	25		69	52	17		71	63	8	
5	1	85	77	8		82	73	9		81	71	10		73	65	8	
	2	84	77	7	8.33	82	75	7	7.33	81	71	10	9.67	75	65	10	8.67
	3	85	75	10		82	76	6		80	71	9		74	66	8	
6	1	96	82	14		97	76	21		85	77	8		88	73	15	
	2	96	85	11	13.67	96	76	20	20.00	84	77	7	7.33	89	68	21	17.33
	3	98	82	16		95	76	19		84	77	7		87	71	16	
7	1	94	79	15		97	74	23		89	75	14		97	66	31	
	2	94	78	16	16.33	95	73	22	23.00	88	73	15	14.33	96	68	28	28.33
	3	95	77	18		96	72	24		88	74	14		93	67	26	
8	1	109	90	19		75	68	7		76	53	23		84	59	25	
	2	108	92	16	16.33	77	68	9	8.67	80	54	26	24.67	83	59	24	25.00
	3	108	94	14		76	66	10		78	53	25		85	59	26	
9	1	102	80	22		104	90	14		95	81	14		86	73	13	
	2	102	81	21	20.67	110	97	13	13.00	98	82	16	15.00	82	75	7	8.33
	3	102	83	19		106	94	12		96	81	15		83	78	5	
10	1	99	72	27		94	69	25		84	69	15		76	69	7	
	2	100	73	27	26.00	94	70	24	24.67	85	71	14	14.67	76	71	5	5.67
	3	98	74	24		93	68	25		86	71	15		76	71	5	
Mean		91.30	74.70	16.60	16.60	85.67	70.07	15.60	15.60	81.57	68.23	13.33	13.33	81.47	68.00	13.47	13.47
SE		2.08	1.80	1.25	2.17	2.08	1.99	1.20	2.11	1.36	1.72	1.08	1.90	1.43	0.90	1.44	2.51

Group 2 Clonidine (d7, d14, d18-22) 00µg/kg (d7,18-22) or 200µg/kg (	Trial	Force (g)		Paw Wt Distribution (g)		Force (g)		Paw Wt Distribution (g)		Force (g)		Paw Wt Distribution (g)		Force (g)		Paw Wt Distribution (g)	
	#	Left	Right	Per Trial	Mean	Left	Right	Per Trial	Mean	Left	Right	Per Trial	Mean	Left	Right	Per Trial	Mean
Animal 1	1	100	76	24		76	66	10		71	56	15		93	66	27	
	2	100	77	23	23.00	77	66	11	11.00	70	56	14	14.00	93	66	27	26.67
	3	100	78	22		77	65	12		70	57	13		93	67	26	
2	1	80	69	11		97	74	23		90	65	25		83	61	22	
	2	81	69	12	13.33	97	74	23	23.33	90	66	24	24.33	84	61	23	22.00
	3	81	64	17		98	74	24		90	66	24		83	62	21	
3	1	94	87	7		77	67	10		93	75	18		83	77	6	
	2	91	84	7	7.00	78	68	10	10.67	94	78	16	15.33	83	77	6	5.67
	3	91	84	7		78	66	12		92	80	12		82	77	5	
4	1	74	65	9		82	59	23		89	79	10		87	80	7	
	2	75	67	8	8.00	82	60	22	22.67	89	80	9	9.33	89	80	9	8.00
	3	73	66	7		83	60	23		89	80	9		88	80	8	
5	1	91	78	13		96	89	7		100	89	11		113	80	33	
	2	93	78	15	15.00	95	88	7	7.33	101	89	12	11.00	113	81	32	32.67
	3	94	77	17		96	88	8		98	88	10		113	80	33	
6	1	97	77	20		96	81	15		84	69	15		102	79	23	
	2	96	72	24	21.00	96	80	16	14.00	85	70	15	15.67	102	79	23	24.33
	3	98	79	19		96	85	11		87	70	17		105	78	27	
7	1	84	66	18		84	79	5		89	84	5		93	88	5	
	2	84	65	19	18.33	84	79	5	5.00	88	84	4	4.33	93	87	6	5.67
	3	82	64	18		85	80	5		90	86	4		95	89	6	
8	1	101	92	9		84	65	19		87	74	13		99	60	39	
	2	100	84	16	12.67	83	65	18	18.00	87	74	13	13.00	99	60	39	39.00
	3	107	94	13		83	66	17		86	73	13		99	60	39	
9	1	98	90	8		73	59	14		113	102	11		100	94	6	
	2	98	84	14	13.67	72	59	13	13.33	111	102	9	8.33	100	94	6	6.00
	3	103	84	19		72	59	13		107	102	5		100	94	6	
10	1	81	70	11		97	79	18		90	74	16		92	67	25	
	2	83	69	14	13.67	97	80	17	17.00	90	74	16	16.00	91	67	24	23.67
	3	83	67	16		97	81	16		90	74	16		89	67	22	
Mean		90.43	75.87	14.57	14.57	86.27	72.03	14.23	14.23	90.33	77.20	13.13	13.13	94.63	75.27	19.37	19.37
SE		1.74	1.64	0.99	1.61	1.67	1.79	1.08	1.92	1.81	2.25	0.98	1.72	1.66	1.99	2.16	3.87

Group 3<br>Cmpd A1<br>IA, 1x/week	Trial<br>#	Force (g)		Paw Wt Distribution (g)		Force (g)		Paw Wt Distribution (g)		Force (g)		Paw Wt Distribution (g)		Force (g)		Paw Wt Distribution (g)	
		Left	Right	Per Trial	Mean	Left	Right	Per Trial	Mean	Left	Right	Per Trial	Mean	Left	Right	Per Trial	Mean
Animal 1	1	97	92	5		87	79	8		89	71	18		88	66	22	
	2	96	89	7	6.00	89	81	8	8.00	89	70	19	17.67	89	66	23	22.33
	3	95	89	6		88	80	8		88	72	16		87	65	22	
2	1	93	80	13		101	79	22		82	71	11		70	57	13	
	2	95	85	10	15.00	101	78	23	22.00	81	72	9	8.67	70	57	13	13.33
	3	99	77	22		100	79	21		80	74	6		72	58	14	
3	1	97	77	20		102	74	28		83	59	24		73	53	20	
	2	99	77	22	22.33	102	75	27	27.67	82	59	23	23.33	72	54	18	19.33
	3	98	73	25		103	75	28		84	61	23		73	53	20	
4	1	71	63	8		106	77	29		93	50	43		78	65	13	
	2	71	64	7	7.67	107	77	30	30.00	93	50	43	43.00	78	65	13	12.67
	3	76	68	8		107	76	31		94	51	43		78	66	12	
5	1	94	74	20		105	82	23		84	71	13		89	63	26	
	2	94	75	19	19.67	107	79	28	26.33	84	71	13	12.00	90	63	27	24.33
	3	94	74	20		108	80	28		83	73	10		87	67	20	
6	1	90	62	28		92	68	24		82	54	28		68	59	9	
	2	93	63	30	26.67	97	67	30	27.00	85	56	29	27.67	70	61	9	10.33
	3	89	67	22		99	72	27		82	56	26		72	59	13	
7	1	88	79	9		109	79	30		94	76	18		82	50	32	
	2	88	80	8	8.67	105	79	26	27.33	92	75	17	18.67	82	50	32	31.67
	3	87	78	9		105	79	26		96	75	21		82	51	31	
8	1	94	85	9		98	82	16		84	69	15		81	49	32	
	2	93	84	9	9.33	99	83	16	15.33	85	68	17	16.00	81	49	32	31.67
	3	94	84	10		98	84	14		84	68	16		80	49	31	
9	1	84	69	15		73	60	13		87	62	25		74	56	18	
	2	84	70	14	14.33	73	60	13	12.67	86	63	23	23.67	73	56	17	18.00
	3	83	69	14		73	61	12		90	67	23		75	56	19	
10	1	75	66	9		87	79	8		78	61	17		64	52	12	
	2	75	63	12	10.00	88	79	9	9.00	79	61	18	17.67	64	53	11	11.67
	3	75	66	9		89	79	10		79	61	18		66	54	12	
Mean		88.70	74.73	13.97	13.97	96.60	76.07	20.53	20.53	85.73	64.90	20.83	20.83	76.93	57.40	19.53	19.53
SE		1.59	1.60	1.29	2.19	1.92	1.19	1.51	2.67	0.91	1.46	1.70	3.03	1.41	1.10	1.41	2.49

Group 4<br>Cmpd B<br>IA, 1x/week	Trial<br>#	Force (g)		Paw Wt Distribution (g)		Force (g)		Paw Wt Distribution (g)		Force (g)		Paw Wt Distribution (g)		Force (g)		Paw Wt Distribution (g)	
		Left	Right	Per Trial	Mean	Left	Right	Per Trial	Mean	Left	Right	Per Trial	Mean	Left	Right	Per Trial	Mean
Animal 1	1	98	74	24		87	77	10		81	50	31		86	64	22	
	2	97	75	22	21.67	86	79	7	7.67	84	52	32	28.67	88	65	23	21.33
	3	95	76	19		84	78	6		75	52	23		81	62	19	
2	1	77	65	12		83	61	22		68	56	12		78	55	23	
	2	77	65	12	12.67	85	63	22	21.33	68	56	12	14.00	78	55	23	22.67
	3	77	63	14		87	67	20		74	56	18		76	54	22	
3	1	95	74	21		70	62	8		76	64	12		73	67	6	
	2	95	75	20	20.00	70	62	8	7.00	73	66	7	8.67	74	68	6	6.33
	3	94	75	19		69	64	5		73	66	7		74	67	7	
4	1	85	56	29		83	55	28		75	69	6		71	53	18	
	2	85	57	28	28.67	83	55	28	28.00	74	69	5	5.67	71	52	19	18.67
	3	86	57	29		85	57	28		74	68	6		71	52	19	
5	1	86	82	4		83	68	15		79	62	17		72	67	5	
	2	86	82	4	3.67	82	69	13	13.00	77	60	17	16.33	73	66	7	6.00
	3	85	82	3		83	72	11		76	61	15		72	66	6	
6	1	78	66	12		77	76	1		90	82	8		82	70	12	
	2	79	65	14	11.33	78	77	1	0.67	89	82	7	7.67	82	69	13	16.00
	3	78	70	8		78	78	0		89	81	8		87	64	23	
7	1	87	73	14		82	75	7		90	61	29		82	64	18	
	2	83	75	8	12.67	82	79	3	7.67	87	61	26	28.33	82	63	19	18.33
	3	89	73	16		86	73	13		91	61	30		82	64	18	
8	1	86	79	7		89	80	9		86	67	19		88	73	15	
	2	85	80	5	5.67	93	70	23	15.00	85	68	17	17.67	88	73	15	15.67
	3	86	81	5		93	80	13		85	68	17		88	71	17	
9	1	97	81	16		108	97	11		89	73	16		84	76	8	
	2	98	83	15	14.00	108	96	12	11.67	89	73	16	14.00	84	76	8	8.00
	3	95	84	11		107	95	12		83	73	10		85	77	8	
10	1	95	74	21		98	88	10		83	70	13		84	72	12	
	2	96	77	19	19.67	95	88	7	8.67	83	70	13	13.67	84	73	11	10.00
	3	91	72	19		96	87	9		86	71	15		86	79	7	
Mean		88.03	73.03	15.00	15.00	86.33	74.27	12.07	12.07	81.07	65.60	15.47	15.47	80.20	65.90	14.30	14.30
SE		1.29	1.47	1.38	2.40	1.84	2.15	1.46	2.48	1.27	1.54	1.44	2.49	1.10	1.41	1.15	1.97

Group 5 Cmpd C1 IA, 1x/week	Trial #	Force (g)		Paw Wt Distribution (g)		Force (g)		Paw Wt Distribution (g)		Force (g)		Paw Wt Distribution (g)		Force (g)		Paw Wt Distribution (g)	
		Left	Right	Per Trial	Mean	Left	Right	Per Trial	Mean	Left	Right	Per Trial	Mean	Left	Right	Per Trial	Mean
Animal 1	1	90	79	11		81	67	14		78	54	24		64	52	12	
	2	91	79	12	11.00	79	68	11	11.00	80	53	27	24.67	65	53	12	11.33
	3	90	80	10		80	72	8		79	56	23		64	54	10	
2	1	90	77	13		96	82	14		99	75	24		80	72	8	
	2	90	78	12	11.33	97	83	14	13.67	99	74	25	24.67	80	73	7	6.67
	3	89	80	9		94	81	13		97	72	25		83	78	5	
3	1	98	69	29		94	74	20		84	58	26		94	64	30	
	2	97	69	28	28.00	95	71	24	23.00	83	58	25	25.33	94	64	30	29.67
	3	97	70	27		96	71	25		83	58	25		93	64	29	
4	1	96	67	29		82	59	23		75	62	13		69	60	9	
	2	95	67	28	27.67	81	59	22	22.67	75	62	13	13.00	68	60	8	9.00
	3	94	68	26		82	59	23		75	62	13		69	59	10	
5	1	96	62	34		97	71	26		98	66	32		88	65	23	
	2	94	62	32	33.33	95	70	25	27.33	96	69	27	28.33	87	63	24	22.33
	3	96	62	34		97	66	31		96	70	26		86	66	20	
6	1	92	64	28		83	74	9		60	53	7		71	68	3	
	2	92	66	26	27.00	82	73	9	9.00	61	53	8	8.00	72	69	3	2.67
	3	92	65	27		83	74	9		60	51	9		71	69	2	
7	1	86	58	28		74	68	6		84	66	18		91	81	10	
	2	86	59	27	26.67	74	68	6	5.67	86	68	18	17.67	90	81	9	9.00
	3	88	63	25		73	68	5		84	67	17		89	81	8	
8	1	85	61	24		85	74	11		82	75	7		82	78	4	
	2	86	62	24	23.67	83	71	12	11.67	82	75	7	6.33	81	78	3	4.00
	3	87	64	23		86	74	12		81	76	5		83	78	5	
9	1	93	71	22		92	79	13		77	69	8		92	68	24	
	2	92	72	20	22.00	93	76	17	15.33	76	68	8	8.00	91	67	24	23.33
	3	97	73	24		93	77	16		78	70	8		90	68	22	
10	1	90	81	9		105	68	37		79	68	11		76	66	10	
	2	90	81	9	6.67	105	72	33	34.67	76	66	10	11.00	74	66	8	7.33
	3	89	87	2		108	74	34		80	68	12		74	70	4	
Mean		91.60	69.87	21.73	21.73	88.83	71.43	17.40	17.40	81.43	64.73	16.70	16.70	80.37	67.83	12.53	12.53
SE		0.69	1.44	1.60	2.82	1.74	1.11	1.64	2.90	1.91	1.39	1.50	2.67	1.80	1.49	1.64	2.91

Group 6 Cmpd C2 IA, 1x/week	Trial #	Force (g)		Paw Wt Distribution (g)		Force (g)		Paw Wt Distribution (g)		Force (g)		Paw Wt Distribution (g)		Force (g)		Paw Wt Distribution (g)	
		Left	Right	Per Trial	Mean	Left	Right	Per Trial	Mean	Left	Right	Per Trial	Mean	Left	Right	Per Trial	Mean
Animal 1	1	90	82	8		83	54	29		82	57	25		74	67	7	
	2	90	83	7	7.33	82	56	26	27.67	81	58	23	24.00	74	66	8	6.67
	3	89	82	7		84	56	28		80	56	24		74	69	5	
2	1	78	61	17		84	65	19		73	57	16		83	74	9	
	2	76	62	14	14.67	88	69	19	16.33	76	58	18	17.33	83	69	14	11.33
	3	75	62	13		86	75	11		75	57	18		83	72	11	
3	1	91	83	8		71	51	20		67	45	22		73	38	35	
	2	92	84	8	8.00	73	51	22	22.67	67	46	21	21.00	73	38	35	34.33
	3	94	86	8		74	48	26		67	47	20		71	38	33	
4	1	91	61	30		93	87	6		86	72	14		87	71	16	
	2	92	64	28	31.00	92	86	6	7.33	86	71	15	14.67	85	71	14	13.67
	3	94	59	35		98	88	10		88	73	15		83	72	11	
5	1	87	64	23		84	66	18		80	71	9		83	70	13	
	2	89	68	21	22.33	84	68	16	17.00	78	72	6	7.00	81	70	11	11.67
	3	95	72	23		84	67	17		77	71	6		82	71	11	
6	1	76	65	11		89	75	14		71	65	6		74	63	11	
	2	75	70	5	9.67	90	75	15	13.00	72	65	7	6.33	71	62	9	9.33
	3	80	67	13		86	76	10		73	67	6		72	64	8	
	1	87	72	15		91	77	14		89	60	29		87	75	12	
	2	91	72	19	17.33	88	75	13	14.00	83	60	23	25.33	87	77	10	11.00
	3	90	72	18		93	78	15		86	62	24		88	77	11	
8	1	81	71	10		72	58	14		87	77	10		73	55	18	
	2	80	70	10	10.00	70	64	6	10.33	84	78	6	7.00	74	54	20	18.33
	3	79	69	10		71	60	11		83	78	5		74	57	17	
9	1	98	69	29		96	75	21		112	77	35		91	71	20	
	2	98	69	29	29.00	98	74	24	22.67	117	77	40	38.00	93	69	24	22.67
	3	99	70	29		99	76	23		115	76	39		95	71	24	
10	1	86	83	3		82	55	27		61	46	15		85	53	32	
	2	90	83	7	9.67	83	56	27	24.33	61	46	15	14.33	83	53	30	30.33
	3	97	78	19		79	60	19		62	49	13		84	55	29	
Mean		87.67	71.77	15.90	15.90	84.90	67.37	17.53	17.53	80.63	63.13	17.50	17.50	80.67	63.73	16.93	16.93
SE		1.35	1.49	1.63	2.77	1.53	2.06	1.25	2.09	2.58	2.02	1.78	3.16	1.28	2.06	1.67	2.95

Group 7<br>Cmpd D<br>IA, 1x/week	Trial #	Force (g) Left	Force (g) Right	Paw Wt Distribution (g) Per Trial	Paw Wt Distribution (g) Mean	Force (g) Left	Force (g) Right	Paw Wt Distribution (g) Per Trial	Paw Wt Distribution (g) Mean	Force (g) Left	Force (g) Right	Paw Wt Distribution (g) Per Trial	Paw Wt Distribution (g) Mean	Force (g) Left	Force (g) Right	Paw Wt Distribution (g) Per Trial	Paw Wt Distribution (g) Mean
Animal 1	1	103	75	28		87	77	10		85	62	23		85	70	15	
	2	102	77	25	26.00	87	75	12	11.33	87	64	23	23.00	85	70	15	15.00
	3	102	77	25		88	76	12		88	65	23		84	69	15	
2	1	92	86	6		70	54	16		84	56	28		75	67	8	
	2	89	84	5	5.33	70	53	17	15.67	84	56	28	27.67	75	69	6	5.00
	3	92	87	5		69	55	14		85	58	27		73	72	1	
3	1	85	71	14		89	69	20		64	50	14		87	55	32	
	2	85	73	12	11.00	88	69	19	19.33	65	51	14	13.33	83	56	27	31.00
	3	80	73	7		87	68	19		65	53	12		86	52	34	
4	1	82	65	17		80	72	8		96	68	28		77	58	19	
	2	79	65	14	14.67	80	73	7	8.67	95	66	29	29.00	76	57	19	19.00
	3	81	68	13		82	71	11		95	65	30		77	58	19	
5	1	90	65	25		87	80	7		80	63	17		79	63	16	
	2	90	65	25	25.00	87	78	9	9.00	84	62	22	19.33	78	62	16	15.00
	3	92	67	25		89	78	11		82	63	19		79	66	13	
6	1	93	87	6		92	78	14		95	64	31		80	34	46	
	2	90	77	13	12.33	94	79	15	14.67	97	65	32	31.67	80	34	46	46.00
	3	91	73	18		95	80	15		96	64	32		80	34	46	
7	1	85	66	19		76	62	14		84	52	32		86	55	31	
	2	85	62	23	23.33	77	64	13	13.33	86	54	32	31.00	91	55	36	36.00
	3	89	61	28		77	64	13		84	55	29		94	53	41	
8	1	99	83	16		93	69	24		88	61	27		68	54	14	
	2	98	83	15	15.67	92	69	23	23.00	88	62	26	25.67	67	55	12	12.67
	3	98	82	16		93	71	22		86	62	24		68	56	12	
9	1	87	79	8		87	77	10		81	64	17		90	66	24	
	2	85	76	9	7.67	88	76	12	12.33	81	61	20	19.33	93	70	23	25.33
	3	83	77	6		88	73	15		84	63	21		96	67	29	
10	1	101	75	26		94	66	28		82	62	20		77	64	13	
	2	100	79	21	23.00	95	65	30	29.67	82	62	20	19.67	76	64	12	13.33
	3	100	78	22		95	64	31		83	64	19		79	64	15	
Mean		90.93	74.53	16.40	16.40	85.87	70.17	15.70	15.70	84.53	60.57	23.97	23.97	80.80	58.97	21.83	21.83
SE		1.33	1.40	1.39	2.37	1.41	1.39	1.19	2.09	1.53	0.89	1.08	1.90	1.37	1.90	2.25	3.97

Group 8<br>Cmpd E<br>IA, 1x/week	Trial #	Force (g) Left	Force (g) Right	Paw Wt Distribution (g) Per Trial	Paw Wt Distribution (g) Mean	Force (g) Left	Force (g) Right	Paw Wt Distribution (g) Per Trial	Paw Wt Distribution (g) Mean	Force (g) Left	Force (g) Right	Paw Wt Distribution (g) Per Trial	Paw Wt Distribution (g) Mean	Force (g) Left	Force (g) Right	Paw Wt Distribution (g) Per Trial	Paw Wt Distribution (g) Mean
Animal 1	1	100	95	5		73	57	16		83	56	27		83	59	24	
	2	102	95	7	6.00	74	57	17	16.33	82	56	26	27.33	83	59	24	24.67
	3	102	96	6		74	58	16		84	55	29		84	58	26	
2	1	95	68	27		81	70	11		71	53	18		76	49	27	
	2	96	67	29	28.67	79	71	8	10.33	71	55	16	17.33	76	50	26	27.00
	3	97	67	30		80	68	12		74	56	18		76	48	28	
3	1	91	65	26		85	61	24		76	52	24		76	42	34	
	2	91	61	30	29.00	85	63	22	21.67	78	54	24	24.00	74	43	31	32.67
	3	92	61	31		82	63	19		77	53	24		76	43	33	
4	1	102	98	4		78	71	7		76	62	14		83	68	15	
	2	101	97	4	4.00	77	71	6	6.33	75	62	13	12.33	84	69	15	14.67
	3	103	99	4		78	72	6		73	63	10		84	70	14	
5	1	88	63	25		95	77	18		84	66	18		87	74	13	
	2	90	64	26	26.33	94	76	18	18.33	84	66	18	18.67	88	74	14	14.33
	3	91	63	28		94	75	19		87	67	20		89	73	16	
6	1	89	78	11		75	62	13		81	53	28		73	42	31	
	2	88	78	10	10.67	73	62	11	11.00	82	54	28	28.33	71	42	29	29.67
	3	85	74	11		74	65	9		84	55	29		73	44	29	
7	1	77	64	13		94	63	31		78	49	29		66	54	12	
	2	76	67	9	11.00	95	64	31	29.67	77	50	27	27.00	69	55	14	10.33
	3	75	64	11		93	66	27		74	49	25		61	56	5	
8	1	90	65	25		76	62	14		62	48	14		69	52	17	
	2	91	67	24	23.67	76	65	11	11.67	61	48	13	13.67	70	53	17	17.00
	3	91	69	22		75	65	10		62	48	14		69	52	17	
9	1	107	75	32		93	64	29		71	46	25		80	57	23	
	2	105	70	35	31.33	93	64	29	28.67	72	45	27	27.00	80	58	22	22.00
	3	100	73	27		90	62	28		76	47	29		79	58	21	
10	1	95	74	21		76	68	8		72	58	14		81	57	24	
	2	95	75	20	19.00	77	68	9	9.00	73	56	17	16.33	78	56	22	24.33
	3	92	76	16		77	67	10		73	55	18		81	54	27	
Mean		93.23	74.27	18.97	18.97	82.20	65.90	16.30	16.30	75.77	54.57	21.20	21.20	77.30	55.63	21.67	21.67
SE		1.49	2.26	1.84	3.25	1.48	0.96	1.46	2.59	1.22	1.11	1.11	1.96	1.26	1.78	1.32	2.32

Table 76. Individual animal data for MMT/MCLT IA Trial 2 Day 21 data

		Day 21 (Pre Tx)				Day 21 (1hr)				Day 21 (3hr)				Day 21 (5hr)			
Group 1 Vehicle (Saline) IA, 1x/week	Trial #	Force (g) Left	Force (g) Right	Paw Wt Distribution (g) Per Trial	Paw Wt Distribution (g) Mean	Force (g) Left	Force (g) Right	Paw Wt Distribution (g) Per Trial	Paw Wt Distribution (g) Mean	Force (g) Left	Force (g) Right	Paw Wt Distribution (g) Per Trial	Paw Wt Distribution (g) Mean	Force (g) Left	Force (g) Right	Paw Wt Distribution (g) Per Trial	Paw Wt Distribution (g) Mean
Animal 1	1	86	75	11		94	66	28		86	60	26		76	60	16	
	2	85	76	9	10.33	98	69	29	29.33	85	61	24	25.00	78	61	17	16.33
	3	86	75	11		99	68	31		86	61	25		77	61	16	
2	1	91	86	5		94	76	18		83	75	8		81	69	12	
	2	89	86	3	5.00	95	76	19	17.00	83	75	8	8.33	80	69	11	11.33
	3	90	83	7		95	81	14		83	74	9		81	70	11	
3	1	95	86	9		97	67	30		66	56	10		91	70	21	
	2	93	86	7	7.67	98	73	25	26.67	67	59	8	9.00	91	71	20	20.33
	3	94	87	7		98	73	25		70	61	9		91	71	20	
4	1	77	56	21		70	62	8		65	57	8		83	57	26	
	2	80	58	22	21.33	72	65	7	7.33	68	59	9	8.67	82	56	26	26.67
	3	78	57	21		72	65	7		67	58	9		83	55	28	
5	1	93	84	9		81	77	4		73	66	7		83	71	12	
	2	93	88	5	7.00	84	77	7	6.67	77	68	9	7.00	83	73	10	10.67
	3	94	87	7		84	75	9		74	69	5		84	74	10	
6	1	90	83	7		103	70	33		86	63	23		75	57	18	
	2	92	82	10	10.00	102	69	33	33.33	85	64	21	22.00	75	57	18	18.33
	3	92	79	13		103	69	34		86	64	22		78	59	19	
7	1	104	77	27		86	74	12		90	78	12		92	80	12	
	2	105	77	28	27.33	82	73	9	10.00	90	78	12	11.67	91	80	11	11.67
	3	104	77	27		85	76	9		90	79	11		92	80	12	
8	1	95	78	17		77	70	7		87	67	20		79	58	21	
	2	94	80	14	15.00	75	70	5	5.67	87	62	25	21.67	80	57	23	21.33
	3	91	77	14		76	71	5		87	67	20		76	56	20	
9	1	89	78	11		88	80	8		76	67	9		76	67	9	
	2	87	77	10	10.33	91	82	9	8.00	76	67	9	8.67	76	70	6	7.00
	3	86	76	10		87	80	7		75	67	8		75	69	6	
10	1	72	68	4		74	72	2		77	64	13		78	67	11	
	2	74	70	4	4.00	76	73	3	3.00	77	64	13	13.00	78	68	10	10.67
	3	74	70	4		77	73	4		77	64	13		79	68	11	
Mean		89.10	77.30	11.80	11.80	87.10	72.40	14.70	14.70	79.30	65.80	13.50	13.50	81.47	66.03	15.43	15.43
SE		1.54	1.59	1.33	2.35	1.91	0.92	1.98	3.52	1.45	1.17	1.21	2.14	1.03	1.40	1.10	1.95

Group 2 Clonidine (d7, d14, d18-22) 00µg/kg (d7,18-22) or 200µg/kg (	Trial #	Force (g) Left	Force (g) Right	Paw Wt Distribution (g) Per Trial	Paw Wt Distribution (g) Mean	Force (g) Left	Force (g) Right	Paw Wt Distribution (g) Per Trial	Paw Wt Distribution (g) Mean	Force (g) Left	Force (g) Right	Paw Wt Distribution (g) Per Trial	Paw Wt Distribution (g) Mean	Force (g) Left	Force (g) Right	Paw Wt Distribution (g) Per Trial	Paw Wt Distribution (g) Mean
Animal 1	1	81	63	18		83	64	19		76	69	7		92	68	24	
	2	80	63	17	17.33	83	64	19	19.00	76	69	7	6.67	94	69	25	25.33
	3	81	64	17		83	64	19		78	72	6		94	67	27	
						*acting very calm starting at 1hr											
2	1	97	68	29		103	84	19		98	72	26		83	71	12	
	2	100	69	31	30.33	102	84	18	18.00	99	72	27	26.33	83	71	12	11.67
	3	100	69	31		100	83	17		98	72	26		83	72	11	
						*acting very calm but seems agitated											
3	1	86	67	19		94	67	27		84	70	14		96	65	31	
	2	84	65	19	17.67	92	65	27	27.33	85	70	15	13.33	97	67	30	29.33
	3	86	71	15		94	66	28		83	72	11		98	71	27	
						*acting very calm starting at 1hr											
4	1	81	62	19		87	63	24		80	72	8		92	87	5	
	2	78	61	17	18.67	86	63	23	23.00	79	72	7	7.33	95	86	9	9.67
	3	83	63	20		86	64	22		80	73	7		98	83	15	
						*acting very calm starting at 1hr											
5	1	89	60	29		107	88	19		98	85	13		106	90	16	
	2	89	62	27	27.67	105	88	17	17.67	98	85	13	12.33	105	91	14	14.33
	3	88	61	27		105	88	17		97	86	11		103	90	13	
						*acting very calm starting at 1hr											
6	1	82	75	7		98	83	15		87	69	18		99	76	23	
	2	80	72	8	7.33	96	84	12	13.33	87	69	18	18.33	98	74	24	23.67
	3	79	72	7		97	84	13		87	68	19		97	73	24	
						*acting very calm starting at 1hr											
7	1	90	77	13		91	81	10		104	89	15		87	70	17	
	2	93	80	13	13.67	92	83	9	9.00	104	86	18	16.33	88	73	15	15.33
	3	93	78	15		91	83	8		103	87	16		86	72	14	
						*acting very calm starting at 1hr											
8	1	83	70	13		80	72	8		72	58	14		78	60	18	
	2	84	73	11	11.33	80	72	8	8.00	73	58	15	14.33	77	61	16	17.00
	3	84	74	10		80	72	8		73	59	14		78	61	17	
9	1	81	63	18		98	79	19		94	72	22		85	68	17	
	2	79	64	15	18.33	98	80	18	18.33	94	71	23	21.67	85	68	17	17.00
	3	82	60	22		97	79	18		91	71	20		85	68	17	
10	1	86	66	20		81	66	15		99	74	25		94	67	27	
	2	82	68	14	15.67	80	66	14	15.00	98	73	25	25.00	92	65	27	27.67
	3	82	69	13		81	65	16		99	74	25		95	66	29	
Mean		85.43	67.63	17.80	17.80	91.67	74.80	16.87	16.87	89.13	72.97	16.17	16.17	91.43	72.33	19.10	19.10
SE		1.11	1.03	1.25	2.18	1.59	1.69	1.05	1.86	1.88	1.46	1.20	2.14	1.44	1.61	1.25	2.18

Group 3 Cmpd A1 IA, 1x/week	Trial #	Force (g)		Paw Wt Distribution (g)		Force (g)		Paw Wt Distribution (g)		Force (g)		Paw Wt Distribution (g)		Force (g)		Paw Wt Distribution (g)	
		Left	Right	Per Trial	Mean	Left	Right	Per Trial	Mean	Left	Right	Per Trial	Mean	Left	Right	Per Trial	Mean
Animal 1	1	100	88	12		112	91	21		92	66	26		84	59	25	
	2	99	86	13	12.00	112	94	18	20.00	89	64	25	24.67	83	59	24	24.33
	3	97	86	11		115	94	21		90	67	23		85	61	24	
2	1	95	63	32		100	84	16		90	72	18		94	70	24	
	2	95	64	31	31.67	101	83	18	16.33	94	73	21	17.67	96	70	26	25.67
	3	97	65	32		100	85	15		89	75	14		96	69	27	
3	1	94	83	11		109	93	16		67	50	17		63	55	8	
	2	90	82	8	9.00	106	90	16	15.33	66	51	15	15.33	64	54	10	8.67
	3	92	84	8		105	91	14		67	53	14		63	55	8	
4	1	90	83	7		101	72	29		77	55	22		78	70	8	
	2	95	86	9	9.00	102	72	30	29.67	78	55	23	22.33	77	70	7	7.67
	3	94	83	11		103	73	30		78	56	22		79	71	8	
5	1	88	77	11		103	79	24		95	62	33		82	69	13	
	2	91	80	11	13.67	104	77	27	27.67	95	63	32	33.67	82	69	13	13.00
	3	91	72	19		106	74	32		98	62	36		83	70	13	
6	1	103	92	11		97	74	23		89	60	29		89	59	30	
	2	106	97	9	12.33	99	79	20	21.33	95	63	32	29.67	88	60	28	28.67
	3	106	89	17		97	76	21		90	62	28		87	59	28	
7	1	86	79	7		97	81	16		77	71	6		96	79	17	
	2	86	79	7	7.00	97	82	15	14.67	79	72	7	7.33	94	80	14	15.00
	3	86	79	7		92	79	13		80	71	9		94	80	14	
8	1	81	61	20		87	70	17		73	58	15		83	50	33	
	2	80	60	20	21.00	87	72	15	16.00	72	58	14	15.67	83	51	32	32.00
	3	85	62	23		87	71	16		75	57	18		82	51	31	
9	1	81	73	8		94	80	14		89	58	31		88	53	35	
	2	81	72	9	8.00	92	81	11	11.67	88	59	29	29.00	88	53	35	34.00
	3	81	74	7		92	82	10		87	60	27		85	53	32	
10	1	96	82	14		92	77	15		91	70	21		87	59	28	
	2	96	82	14	14.67	93	78	15	15.00	91	71	20	20.67	86	60	26	26.33
	3	93	77	16		94	79	15		90	69	21		87	62	25	
Mean		91.83	78.00	13.83	13.83	99.20	80.43	18.77	18.77	84.37	62.77	21.60	21.60	84.20	62.67	21.53	21.53
SE		1.34	1.76	1.36	2.36	1.37	1.31	1.07	1.86	1.71	1.29	1.43	2.52	1.61	1.63	1.71	3.05

Group 4 Cmpd B IA, 1x/week	Trial #	Force (g)		Paw Wt Distribution (g)		Force (g)		Paw Wt Distribution (g)		Force (g)		Paw Wt Distribution (g)		Force (g)		Paw Wt Distribution (g)	
		Left	Right	Per Trial	Mean	Left	Right	Per Trial	Mean	Left	Right	Per Trial	Mean	Left	Right	Per Trial	Mean
Animal 1	1	76	70	6		91	68	23		81	69	12		74	43	31	
	2	76	70	6	6.33	91	69	22	22.67	80	67	13	11.67	72	47	25	27.33
	3	77	70	7		91	68	23		76	66	10		73	47	26	
2	1	83	67	16		83	76	7		75	51	24		95	68	27	
	2	86	70	16	15.33	84	78	6	5.33	74	51	23	23.67	95	68	27	27.00
	3	83	69	14		80	77	3		75	51	24		96	69	27	
3	1	81	54	27		92	72	20		74	59	15		79	50	29	
	2	81	51	30	28.33	91	71	20	20.33	70	62	8	10.00	80	50	30	29.67
	3	81	53	28		91	70	21		74	67	7		79	49	30	
4	1	86	71	15		82	55	27		83	49	34		78	47	31	
	2	87	71	16	15.67	84	56	28	26.67	83	48	35	35.67	78	49	29	29.33
	3	87	71	16		82	57	25		85	47	38		78	50	28	
5	1	65	51	14		87	75	12		64	52	12		74	65	9	
	2	67	55	12	10.33	88	76	12	12.33	64	52	12	11.00	74	65	9	9.33
	3	63	58	5		87	74	13		62	53	9		74	64	10	
6	1	90	72	18		83	55	28		73	66	7		78	70	8	
	2	91	72	19	18.67	83	57	26	27.33	72	57	15	12.00	76	68	8	9.00
	3	92	73	19		86	58	28		74	60	14		78	67	11	
7	1	84	65	19		94	86	8		84	69	15		73	54	19	
	2	83	64	19	19.67	93	86	7	7.33	86	69	17	16.33	73	54	19	19.00
	3	85	64	21		94	87	7		84	67	17		74	55	19	
8	1	57	56	1		77	68	9		71	48	23		75	60	15	
	2	58	57	1	0.67	78	69	9	6.67	68	46	22	24.67	75	60	15	15.00
	3	59	59	0		78	76	2		78	49	29		76	61	15	
9	1	74	56	18		82	73	9		76	44	32		76	69	7	
	2	74	58	16	16.00	83	75	8	8.67	73	45	28	27.33	75	71	4	5.67
	3	73	59	14		82	73	9		72	50	22		75	69	6	
10	1	73	71	2		65	52	13		92	65	27		76	66	10	
	2	75	68	7	10.67	66	50	16	15.67	91	66	25	25.67	78	66	12	13.67
	3	88	65	23		69	51	18		89	64	25		81	62	19	
Mean		77.83	63.67	14.17	14.17	83.90	68.60	15.30	15.30	76.77	56.97	19.80	19.80	77.93	59.43	18.50	18.50
SE		1.83	1.33	1.47	2.42	1.40	1.94	1.51	2.67	1.42	1.58	1.61	2.78	1.15	1.62	1.66	2.92

Group 5<br>Cmpd C1<br>IA, 1x/week	Trial #	Force (g)		Paw Wt Distribution (g)		Force (g)		Paw Wt Distribution (g)		Force (g)		Paw Wt Distribution (g)		Force (g)		Paw Wt Distribution (g)	
		Left	Right	Per Trial	Mean	Left	Right	Per Trial	Mean	Left	Right	Per Trial	Mean	Left	Right	Per Trial	Mean
Animal 1	1	85	61	24		85	65	20		65	55	10		72	52	20	
	2	81	68	13	17.00	84	66	18	18.33	66	56	10	9.33	70	52	18	18.67
	3	83	69	14		86	69	17		66	58	8		72	54	18	
														*Seeing slight pain in this group			
2	1	106	89	17		96	82	14		89	75	14		106	86	20	
	2	105	89	16	16.33	90	84	6	10.00	85	76	9	12.67	107	89	18	20.00
	3	106	90	16		95	85	10		91	76	15		109	87	22	
3	1	92	80	12		94	73	21		86	52	34		87	59	28	
	2	91	80	11	11.00	93	73	20	21.33	83	54	29	32.33	88	56	32	29.33
	3	89	79	10		93	70	23		85	51	34		86	58	28	
										*Seeing slight pain in Sx leg							
4	1	97	74	23		92	71	21		90	68	22		66	57	9	
	2	93	76	17	18.67	92	71	21	21.67	89	69	20	23.67	66	56	10	10.00
	3	94	78	16		95	72	23		94	65	29		68	57	11	
										*Seeing slight pain in Sx leg							
5	1	94	76	18		82	69	13		81	67	14		85	62	23	
	2	95	76	19	20.33	80	67	13	13.33	80	67	13	14.33	83	66	17	19.00
	3	95	71	24		81	67	14		82	66	16		83	66	17	
										*Seeing slight pain in Sx leg				*Increased pain in Sx leg			
6	1	99	87	12		90	68	22		81	58	23		94	67	27	
	2	97	87	10	10.00	90	67	23	22.33	81	59	22	22.33	93	67	26	26.00
	3	97	89	8		90	68	22		82	60	22		92	67	25	
										*Seeing slight pain in Sx leg				*Increased pain in Sx leg			
7	1	105	80	25		89	74	15		79	64	15		89	58	31	
	2	103	81	22	22.33	89	76	13	14.33	78	64	14	14.67	89	59	30	30.33
	3	102	82	20		89	74	15		77	62	15		89	59	30	
										*Seeing slight pain in Sx leg				*Increased pain in Sx leg			
8	1	107	81	26		73	61	12		78	66	12		66	61	5	
	2	108	83	25	24.00	73	62	11	11.33	78	67	11	11.33	65	61	4	4.00
	3	106	85	21		73	62	11		79	68	11		66	63	3	
9	1	104	80	24		86	74	12		99	72	27		76	61	15	
	2	105	79	26	26.33	87	74	13	11.67	98	75	23	23.00	79	61	18	16.67
	3	108	79	29		85	75	10		94	75	19		78	61	17	
10	1	89	68	21		79	67	12		87	57	30		78	67	11	
	2	87	60	27	24.67	78	69	9	11.00	86	56	30	29.67	78	68	10	11.00
	3	89	63	26		81	69	12		86	57	29		79	67	12	
**Mean**		**97.07**	**78.00**	**19.07**	**19.07**	**86.33**	**70.80**	**15.53**	**15.53**	**83.17**	**63.83**	**19.33**	**19.33**	**81.97**	**63.47**	**18.50**	**18.50**
**SE**		**1.47**	**1.51**	**1.09**	**1.76**	**1.23**	**1.07**	**0.90**	**1.55**	**1.53**	**1.37**	**1.45**	**2.52**	**2.27**	**1.70**	**1.53**	**2.70**

Group 6<br>Cmpd C2<br>IA, 1x/week	Trial #	Force (g)		Paw Wt Distribution (g)		Force (g)		Paw Wt Distribution (g)		Force (g)		Paw Wt Distribution (g)		Force (g)		Paw Wt Distribution (g)	
		Left	Right	Per Trial	Mean	Left	Right	Per Trial	Mean	Left	Right	Per Trial	Mean	Left	Right	Per Trial	Mean
Animal 1	1	91	76	15		89	75	14		73	58	15		78	50	28	
	2	91	76	15	15.33	89	79	10	13.00	71	58	13	14.00	78	51	27	27.00
	3	92	76	16		90	75	15		73	59	14		77	51	26	
														*Showing slight pain in Sx leg			
2	1	97	89	8		90	65	25		94	66	28		91	63	28	
	2	95	88	7	7.33	90	64	26	26.33	92	64	28	29.00	90	64	26	25.33
	3	98	91	7		91	63	28		95	64	31		88	66	22	
3	1	87	64	23		74	66	8		72	57	15		64	54	10	
	2	86	67	19	21.33	74	66	8	7.67	71	58	13	14.33	62	55	7	8.33
	3	87	65	22		74	67	7		73	58	15		63	55	8	
4	1	93	81	12		86	75	11		75	54	21		83	73	10	
	2	94	81	13	12.67	84	73	11	10.67	73	53	20	20.67	82	72	10	10.33
	3	92	79	13		88	78	10		74	53	21		83	72	11	
5	1	95	82	13		92	74	18		87	58	29		83	59	24	
	2	90	83	7	10.33	89	75	14	15.67	88	60	28	28.00	82	58	24	24.00
	3	98	87	11		90	75	15		88	61	27		83	59	24	
6	1	74	50	24		74	68	6		81	62	19		84	63	21	
	2	74	55	19	21.67	74	66	8	7.67	82	59	23	20.00	85	65	20	20.33
	3	76	54	22		74	65	9		81	63	18		82	62	20	
										*Showing slight pain in Sx leg							
	1	86	77	9		81	71	10		78	54	24		88	62	26	
	2	87	79	8	8.33	79	71	8	8.33	78	55	23	23.67	88	62	26	25.67
	3	86	78	8		79	72	7		79	55	24		87	62	25	
										*Showing slight pain in Sx leg							
8	1	68	65	3		79	68	11		66	45	21		51	42	9	
	2	71	63	8	5.67	77	70	7	8.67	69	46	23	22.00	51	41	10	7.67
	3	72	66	6		79	71	8		69	47	22		50	46	4	
9	1	105	67	38		104	72	32		94	68	26		71	61	10	
	2	102	66	36	37.67	104	73	31	31.00	98	69	29	27.67	73	63	10	10.00
	3	107	68	39		104	74	30		98	70	28		72	62	10	
10	1	90	80	10		76	54	22		77	66	11		79	58	21	
	2	88	80	8	7.00	78	56	22	21.67	74	69	5	8.67	79	59	20	20.67
	3	87	84	3		78	57	21		78	68	10		79	58	21	
**Mean**		**88.63**	**73.90**	**14.73**	**14.73**	**84.33**	**69.27**	**15.07**	**15.07**	**80.03**	**59.23**	**20.80**	**20.80**	**76.87**	**58.93**	**17.93**	**17.93**
**SE**		**1.80**	**1.97**	**1.77**	**3.13**	**1.68**	**1.14**	**1.51**	**2.68**	**1.72**	**1.24**	**1.22**	**2.14**	**2.12**	**1.44**	**1.42**	**2.51**

Group 7 Cmpd D IA, 1x/week	Trial	Force (g)		Paw Wt Distribution (g)		Force (g)		Paw Wt Distribution (g)		Force (g)		Paw Wt Distribution (g)		Force (g)		Paw Wt Distribution (g)	
	#	Left	Right	Per Trial	Mean	Left	Right	Per Trial	Mean	Left	Right	Per Trial	Mean	Left	Right	Per Trial	Mean
**Animal 1**	1	98	81	17		103	81	22		93	66	27		79	64	15	
	2	98	82	16	**15.33**	101	80	21	**21.00**	96	68	28	**27.67**	78	63	15	**14.33**
	3	95	82	13		101	81	20		93	65	28		78	65	13	
										*showing slight pain in Sx leg							
**2**	1	101	95	6		100	93	7		88	84	4		81	70	11	
	2	102	96	6	**5.00**	100	93	7	**6.67**	90	86	4	**3.33**	80	70	10	**9.67**
	3	100	97	3		99	93	6		88	86	2		80	72	8	
**3**	1	97	86	11		80	61	19		90	74	16		83	61	22	
	2	96	87	9	**10.33**	81	61	20	**20.00**	92	71	21	**20.67**	84	61	23	**21.67**
	3	95	84	11		83	62	21		95	70	25		82	62	20	
**4**	1	88	73	15		88	79	9		84	72	12		70	67	3	
	2	88	75	13	**12.67**	95	80	15	**8.67**	83	75	8	**10.33**	70	67	3	**3.33**
	3	89	79	10		88	86	2		82	71	11		69	65	4	
**5**	1	102	81	21		87	73	14		82	61	21		87	69	18	
	2	104	82	22	**22.67**	87	74	13	**13.00**	84	60	24	**22.00**	89	70	19	**18.33**
	3	105	80	25		87	75	12		84	63	21		88	70	18	
										*showing slight pain in Sx leg							
**6**	1	104	89	15		82	61	21		84	54	30		76	54	22	
	2	106	89	17	**19.00**	89	66	23	**23.00**	87	54	33	**31.00**	77	57	20	**20.67**
	3	107	82	25		87	62	25		84	54	30		75	55	20	
										*showing slight pain in Sx leg							
**7**	1	100	82	18		97	78	19		80	65	15		77	57	20	
	2	99	86	13	**16.33**	96	75	21	**19.67**	80	67	13	**14.00**	76	58	18	**18.67**
	3	102	84	18		96	77	19		82	68	14		77	59	18	
														*showing slight pain in Sx leg			
**8**	1	88	77	11		78	64	14		56	50	6		63	54	9	
	2	89	76	13	**11.67**	77	64	13	**13.67**	56	50	6	**5.33**	62	55	7	**8.00**
	3	88	77	11		77	63	14		55	51	4		63	55	8	
**9**	1	107	81	26		87	59	28		72	65	7		76	56	20	
	2	107	81	26	**26.00**	90	58	32	**30.33**	72	64	8	**7.67**	78	58	20	**20.67**
	3	105	79	26		90	59	31		72	64	8		76	54	22	
**10**	1	90	73	17		91	71	20		69	55	14		78	64	14	
	2	85	73	12	**14.00**	93	72	21	**20.33**	69	56	13	**16.00**	78	65	13	**13.67**
	3	87	74	13		90	70	20		74	53	21		77	63	14	
**Mean**		**97.40**	**82.10**	**15.30**	**15.30**	**90.00**	**72.37**	**17.63**	**17.63**	**80.53**	**64.73**	**15.80**	**15.80**	**76.90**	**62.00**	**14.90**	**14.90**
**SE**		**1.30**	**1.18**	**1.14**	**1.93**	**1.39**	**1.94**	**1.30**	**2.25**	**2.06**	**1.85**	**1.70**	**2.98**	**1.22**	**1.05**	**1.11**	**1.97**

Group 8 Cmpd E IA, 1x/week	Trial	Force (g)		Paw Wt Distribution (g)		Force (g)		Paw Wt Distribution (g)		Force (g)		Paw Wt Distribution (g)		Force (g)		Paw Wt Distribution (g)	
	#	Left	Right	Per Trial	Mean	Left	Right	Per Trial	Mean	Left	Right	Per Trial	Mean	Left	Right	Per Trial	Mean
**Animal 1**	1	109	91	18		94	69	25		77	52	25		71	41	30	
	2	106	90	16	**15.00**	94	69	25	**25.33**	75	56	19	**21.33**	69	43	26	**27.00**
	3	103	92	11		95	69	26		77	57	20		70	45	25	
						*showing slight pain in Sx leg				*pain inreasing				*Pain increasing			
**2**	1	99	69	30		95	66	29		70	62	8		84	52	32	
	2	101	71	30	**31.00**	96	69	27	**28.00**	75	63	12	**9.33**	85	55	30	**29.33**
	3	101	68	33		96	68	28		71	63	8		84	58	26	
														*showing slight pain in Sx leg			
**3**	1	86	75	11		76	62	14		84	61	23		81	56	25	
	2	86	77	9	**9.67**	76	64	12	**12.00**	84	64	20	**21.00**	82	55	27	**26.67**
	3	85	76	9		76	66	10		83	63	20		83	55	28	
										*showing slight pain in Sx leg							
**4**	1	104	95	9		93	82	11		94	68	26		77	46	31	
	2	100	93	7	**8.67**	92	78	14	**14.00**	93	66	27	**26.33**	72	46	26	**27.33**
	3	102	92	10		97	80	17		93	67	26		73	48	25	
										*showing slight pain in Sx leg				*Pain increasing			
**5**	1	109	90	19		92	72	20		86	62	24		73	55	18	
	2	110	90	20	**20.67**	93	72	21	**21.00**	85	64	21	**21.67**	72	55	17	**17.33**
	3	114	91	23		93	71	22		84	64	20		74	57	17	
										*showing slight pain in Sx leg							
**6**	1	83	62	21		78	68	10		84	54	30		67	52	15	
	2	84	62	22	**21.00**	78	69	9	**8.00**	83	53	30	**29.00**	67	53	14	**14.00**
	3	82	62	20		76	71	5		82	55	27		66	53	13	
										*showing slight pain in Sx leg							
**7**	1	86	82	4		91	77	14		78	67	11		83	69	14	
	2	84	80	4	**3.33**	86	77	9	**10.33**	79	70	9	**10.00**	82	68	14	**14.67**
	3	85	83	2		85	77	8		78	68	10		83	67	16	
**8**	1	74	58	16		69	61	8		78	53	25		83	66	17	
	2	72	58	14	**14.00**	70	63	7	**7.67**	74	52	22	**22.33**	83	66	17	**18.00**
	3	71	59	12		71	63	8		74	54	20		82	62	20	
**9**	1	71	65	6		84	55	29		72	51	21		88	69	19	
	2	75	69	6	**6.33**	84	55	29	**30.67**	74	50	24	**22.33**	89	69	20	**20.00**
	3	77	70	7		87	53	34		72	50	22		89	68	21	
**10**	1	90	68	22		83	73	10		73	45	28		66	45	21	
	2	92	65	27	**24.33**	83	67	16	**13.00**	78	43	35	**31.33**	66	44	22	**21.67**
	3	90	66	24		87	74	13		75	44	31		68	46	22	
**Mean**		**91.03**	**75.63**	**15.40**	**15.40**	**85.67**	**68.67**	**17.00**	**17.00**	**79.50**	**58.03**	**21.47**	**21.47**	**77.07**	**55.47**	**21.60**	**21.60**
**SE**		**2.36**	**2.26**	**1.57**	**2.77**	**1.58**	**1.32**	**1.54**	**2.70**	**1.20**	**1.41**	**1.30**	**2.26**	**1.41**	**1.64**	**1.04**	**1.79**

1. Gerwin N, Bendele AM, Glasson S, Carlson CS. The OARSI histopathology initiative - recommendations for histological assessments of osteoarthritis in the rat. Osteoarthritis Cartilage 2010;18 Suppl 3(S24-34.
